# A comprehensive overview of directing groups applied in metal-catalysed C–H functionalisation chemistry†
†Dedicated to the memory of Prof. Walther Schmidt.
‡
‡Electronic supplementary information (ESI) available. See DOI: 10.1039/c8cs00201k


**DOI:** 10.1039/c8cs00201k

**Published:** 2018-07-23

**Authors:** Carlo Sambiagio, David Schönbauer, Remi Blieck, Toan Dao-Huy, Gerit Pototschnig, Patricia Schaaf, Thomas Wiesinger, Muhammad Farooq Zia, Joanna Wencel-Delord, Tatiana Besset, Bert U. W. Maes, Michael Schnürch

**Affiliations:** a Organic Synthesis (ORSY) , Department of Chemistry , University of Antwerp , Groenenborgerlaan 171 , 2020 Antwerp , Belgium; b Institute of Applied Synthetic Chemistry , TU Wien , Getreidemarkt 9/163 , A-1060 Vienna , Austria . Email: michael.schnuerch@tuwien.ac.at; c Normandie Univ , INSA Rouen , UNIROUEN , CNRS , COBRA (UMR 6014) , 76000 Rouen , France; d Laboratoire de Chimie Moléculaire (UMR CNRS 7509) , Université de Strasbourg , ECPM 25 Rue Becquerel , 67087 Strasbourg , France

## Abstract

The present review is devoted to summarizing the recent advances (2015–2017) in the field of metal-catalysed group-directed C–H functionalisation. In order to clearly showcase the molecular diversity that can now be accessed by means of directed C–H functionalisation, the whole is organized following the directing groups installed on a substrate. Its aim is to be a comprehensive reference work, where a specific directing group can be easily found, together with the transformations which have been carried out with it. Hence, the primary format of this review is schemes accompanied with a concise explanatory text, in which the directing groups are ordered in sections according to their chemical structure. The schemes feature typical substrates used, the products obtained as well as the required reaction conditions. Importantly, each example is commented on with respect to the most important positive features and drawbacks, on aspects such as selectivity, substrate scope, reaction conditions, directing group removal, and greenness. The targeted readership are both experts in the field of C–H functionalisation chemistry (to provide a comprehensive overview of the progress made in the last years) and, even more so, all organic chemists who want to introduce the C–H functionalisation way of thinking for a design of straightforward, efficient and step-economic synthetic routes towards molecules of interest to them. Accordingly, this review should be of particular interest also for scientists from industrial R&D sector. Hence, the overall goal of this review is to promote the application of C–H functionalisation reactions outside the research groups dedicated to method development and establishing it as a valuable reaction archetype in contemporary R&D, comparable to the role cross-coupling reactions play to date.

## Abstract 2

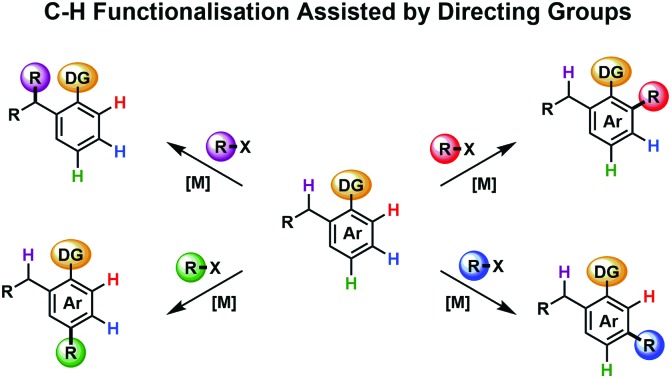
The present review is devoted to summarizing the recent advances (2015–2017) in the field of metal-catalysed group-directed C–H functionalisation.

## 1. Introduction

**Fig. 1:**
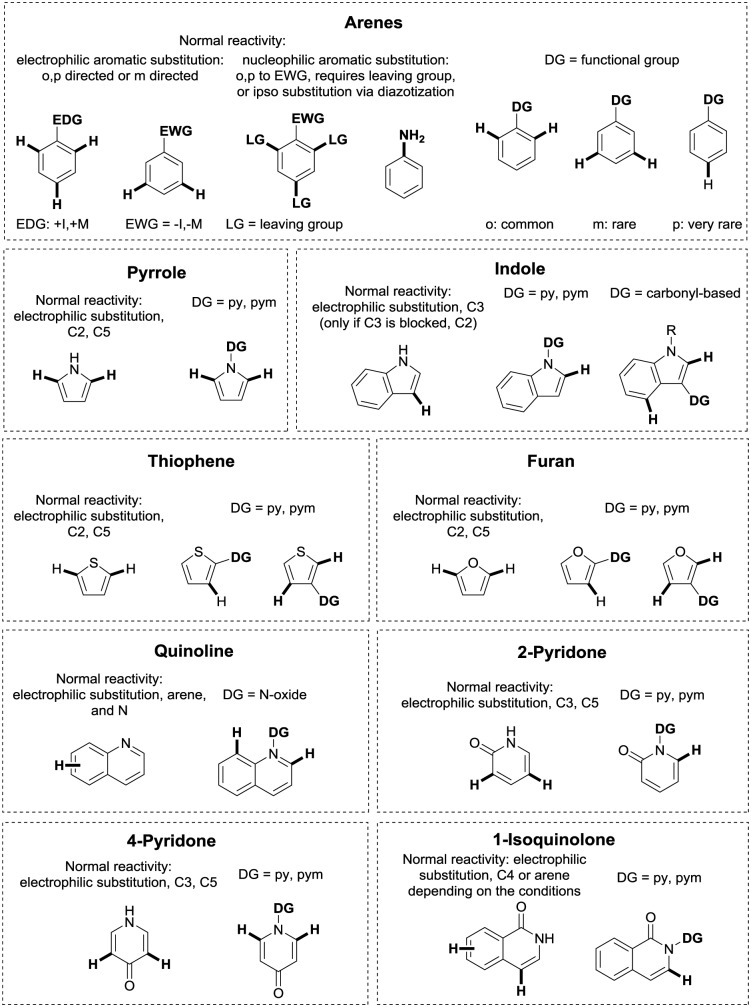
Examples of classical reactivity *versus* DG approach.

Metal catalysed C–H functionalisation chemistry is a rapidly expanding field with research going into many different directions.^1^–^21^ Due to the ubiquity of C–H bonds in organic molecules, selective functionalisation of a specific C–H bond is highly challenging. In aromatic heterocycles, the heteroatoms usually guide the regioselectivity of C–H functionalisation reactions due to their influence on the electron density of the different C–H positions.^22^–^26^ On arenes or aliphatic compounds, such intrinsic reactivity differences are significantly less pronounced, and selective cleavage of a specific C–H bond is more difficult, even though substituents in arenes can obviously have a significant effect. One way to overcome this problem is the assistance of directing groups (DGs), consisting of a coordinating moiety (an “internal ligand”), which directs a metal catalyst into the proximity of a certain C–H bond in the molecule, leading to its selective cleavage and subsequent functionalisation.^17^,^27^–^33^ This strategy allows to overrule innate reactivity in (hetero)arenes. A schematic example of this is shown in Fig. 1 for a number of common unsaturated heterocycles, where a comparison with classical electrophilic substitution is given.^34^

DG-assisted C–H functionalisation started to gain momentum in the mid-1990s after a landmark contribution by the group of Murai.^35^ In this paper, a ketone DG enabled the Ru(ii)-catalysed *ortho*-alkylation of arenes with alkenes, clearly illustrating that the regioselectivity issued might be selectively tackled if formation of a metallacyclic intermediate occurs. Since then, a huge number of papers on the topic have been published, expanding the scope of potential DGs and of chemical transformations which can be carried out.

### 1.1. Limitations to the use of C–H functionalisation in molecules for R&D or in total synthesis

Most of the published contributions can be classified as methodological papers. Typically, substrates are designed which align themselves nicely in order to allow a selective C–H cleavage. The complexity of these substrates is typically limited, since they are usually from the pool of readily available small molecules featuring a standard functional group (FG) compatibility. Applications in the field of R&D^36^–^38^ and total synthesis of complex molecules^39^–^45^ have been reported, however those remain the exception. For the former it is of course difficult to get an objective view on the situation as most results obtained in fine chemicals industry are never published. However, when comparing the number of cross coupling reactions and direct C–H functionalisations disclosed in the scientific literature by industry, one can safely conclude that the area is still in its infancy. This is somewhat surprising, considering the amount of transformations, which have been reported so far from academia and the wide variety of DGs which can potentially promote them. You might think that in a retrosynthesis, you frequently run into situations, in which transition metal-catalysed C–H functionalisation would be a potential and even the preferred option. So what are the reasons why C–H functionalisation has not yet reached the level of general applicability which related transformations, *e.g.* cross-coupling reactions, have achieved already quite some time ago? Considering cross-coupling reactions, it can be noticed that the majority of coupling partners organometals and (pseudo)halides (Suzuki–Miyaura, Stille, Negishi, Hiyama), or (pseudo)halides and olefins (Heck) or alkynes (Sonogashira) in order to create new C–C bonds without regioselectivity issues. Hence, in retrosynthetic analysis, cross-coupling reactions are quickly identified and nowadays these are standard transformations frequently applied in all contexts of synthesis, also including the most complex area of total synthesis of natural products. Additionally, the metals applied are also only a few with Pd being the single most important one dominating the field, followed by Cu and Ni (recently). All other metals play only relative minor roles at the moment, although research in this field is also increasing.

In case of C–H functionalisation, the situation is much more complex due to the higher diversity in coupling partners which often react *via* distinctively different reaction mechanisms, and a large variety of specific additives which these reactions require (often complex mixtures of reagents and additives are required for each transformation). To facilitate the understanding and the choice of a specific procedure, below are listed the different types of additives and their roles in C–H functionalisation reactions. It is important to stress that, although the main classes of additives and roles are relatively clear, many compounds can have more than one distinct role in the reaction, therefore their choice and combinations is not straightforward.

• Oxidants: most often Cu salts (commonly Cu(OAc)_2_), and Ag salts (sometimes Mn salts) are used in stoichiometric or superstoichiometric amounts in oxidative reactions. Other oxidants, used alone or in combination with Cu or Ag are benzoquinones, peroxides, O_2_/air, K_2_S_2_O_8_ or hypervalent iodine compounds.

• Catalytic Ag salts: catalytic amounts of Ag salts are often used in combination with groups 8 or 9 metal halide dimers, commonly used as catalysts (*e.g.* [RhCp*Cl_2_]_2_). In these cases the Ag acts as a halide scavenger, and the counteranion (usually OTf, NTf_2_, or SbF_6_) promotes the *in situ* formation of cationic metal catalysts in solution. When a suitable preformed cationic metal catalyst is used, the addition of such additives is generally not required.

• Carboxylates: carboxylates (acetates, benzoates, pivalates, adamantanecarboxylates, trifluoroacetates and others) are very common additives in many C–H functionalisation protocols. Their role is mostly related to their ability to deprotonate the desired C–H bond, often *via* a concerted metallation–deprotonation (CMD) pathway (also termed ambiphilic metal ligand activation (AMLA)).^2^,^46^,^47^ These additives are generally added as acids in stoichiometric or catalytic amounts, often in combination with bases, or can be added as their Cu, Ag, Zn, Na salts.

• Ligands: although typically no external ligands are required in C–H functionalisation reactions (the DG acts as an “internal ligand”), in some cases an external auxiliary (catalytic amounts) is required to facilitate the reaction. These can be phosphines, carbenes, mono-protected aminoacids (MPAA) or other bidentate ligands. Noteworthy, the choice of the ligand will strongly depend on the mechanism of a targeted transformation (for instance Pd(ii)/Pd(0) catalysis *vs.* Pd(ii)/Pd(iv) catalytic cycle).

• Lewis acids: in this category many compounds can fall, including some of the above. Although most often simple compounds are used, such as Zn salts, the applied Lewis acids span over a wide range of reagents also including more exotic In/Gd salts or BPh_3_, as can be seen in the examples in this review. These can display different roles, including the activation of coupling partners (*e.g.* activation of ketones/aldehydes in alkylation reactions), and can be added in stoichiometric or catalytic amounts.

• Bases: a number of different bases, usually inorganic ones, *e.g.* carbonates are used to neutralize acids formed during the reaction, or to facilitate deprotonation of the starting materials/reactants/additives used. It is worth noting that common bases used for cross coupling reactions, such as Cs_2_CO_3_, are instead not often encountered in C–H functionalisation, while Na, K, or Li counterparts are generally more effective (Ag_2_CO_3_ is often used as both oxidant and base).

The variety of additives and reaction conditions encountered in this field is also reflected by the range of metals which are applied as catalysts. There is no such thing as a single dominant metal species, but Pd,^48^,^49^ Rh,^50^,^51^ and Ru^52^–^54^ share together the top spot. Moreover, increasing examples using Ni,^55^–^58^ Co,^55^,^59^–^64^ Ir,^64^–^66^ Cu,^67^–^69^ Fe,^3^,^55^,^63^,^70^,^71^ and Mn^72^–^74^ are appearing in the literature.

Naturally, such a manifold of metals with often distinctively different properties adds a lot of complexity to the field. This is of course an advantage for method development, since it is only logical that a larger pool of catalyst candidates allows also for a larger variety of possible transformations. On the other hand, the complexity has reached a level where it is very difficult to keep track of the developments and to get an overview of what is actually possible in C–H functionalisations.

What does this mean for a synthetic chemist planning a multistep synthesis of a molecule? The retrosynthetic disconnections might frequently lead to situations in which C–H functionalisation chemistry could be applied. However, because of the diversity of the field and the lack of a general and easy to consult overview, its application is unfortunately not even considered in many cases. This is the shortcoming which the authors attempted to address by this review. So overall, this review aims at giving a comprehensive overview on the types of FGs available for transition metal-catalysed C–H cleavage reactions and the types of transformation they allow, and the associated required “reaction cocktail”. This should be very useful for taking this research field to the next step, the more frequent application in syntheses, also at late stages of prolonged synthetic sequences.

### 1.2. Structure and scope of the review

Our aim in this review was to present the DGs and possible transformations in a concise way so that all readers can easily find the specific transformation looked for. Hence, the review is divided in sections dedicated to specific types of DGs: (i) monodentate carbonyl-containing DGs, (ii) bidentate DGs, (iii) heterocyclic DGs, and (iv) heteroatom-based DGs (N, S, P, Si…). These main sections are further subdivided into specific functional groups (*e.g.* ketones, amides, *etc.*), and then into alkylations, alkenylations, arylations, *etc.* In turns, for each of these subsections, the different procedures reported in the literature have been summarised in schemes, making it easier to compare them with each other. To facilitate this task, a list of advantages/disadvantages has been added for each procedure, concerning issues such as selectivity, substrate scope, reaction conditions, DG removal, and overall greenness. We realise that said advantages/disadvantages can be somewhat subjective, and what is good for one purpose may not be so good for another. For example, the use of halogenated solvents has been considered as a drawback in this review. This has not the aim of criticising the research methodology, neither reducing the value of the results reported, but the use of such solvents is a no-go in chemical manufacturing, and therefore is something that necessarily has disadvantages in terms of that procedure being used for such a purpose. The same reasoning applies to the other advantages/disadvantages considered in the review. In particular, among the drawbacks, the following have been specifically listed: toxic solvents; reaction temperatures > 120 °C; reaction time > 24 h; (most) yields < 70%; superstoichiometric amounts of (metal) oxidants in oxidative couplings; regio- or stereoisomeric mixtures; specific limitations in scope or FG tolerance (*e.g.* sensitivity to steric hindrance); use of additional ligands, unless in stereoselective reactions (the DG is already a ligand, and most non-stereoselective procedures do not require additional ligands). As positive aspects, the following are instead considered: green(er) solvents; reaction temperature < 50 °C; reaction times < 10 h; (most) yields > 80–90%; particularly good FG tolerance (halide, methoxy, alkyl, ester and cyanide are considered generally standard substituents, if something more advanced, such as alkene, alkyne, boryl, silyl, or free NH/OH groups are also tolerated, this has been mentioned as an advantage); other substrates and other DGs available for the reaction; DG removal (when specifically mentioned); *in situ*, one-pot, or cascade processes; scalability. Other aspects have not been mentioned as specific advantages/disadvantages, mostly because of the lack of a real distinction between the two; a catalyst loading up to 10 mol% metal has been considered standard, as most protocols use these amounts, though it does not mean this is what is acceptable for chemical development purposes (higher than 10 mol% metal for expensive catalyst has been considered specifically as a disadvantage); characteristic of reactants have also not been generally considered (*e.g.* availability or cost), as a large variety exist and price is heavily depending on the scale required.

Another important aspect of the review is the stress that has been placed on the DG removal. The removal of DGs is an important drawback in C–H functionalisation chemistry, as the DG might not be required after the directed step, and its removal is therefore necessary (which is an extra synthetic step). Several DGs are actually not removable (depending on the substrate as well) which is no problem when they anyway constitute a (precursor) part of the target molecule, while others are easily removed or modified. We tried to incorporate all the possible protocols for the removal or modification of the DGs in each section, so that the reader will be immediately aware of what can currently be done with the DG in use. This information is often not reported in reviews dealing with C–H functionalisation. The removal of the DG has also been listed as a specific advantage in each procedure, in case this is specifically investigated by the authors. So not being mentioned does not mean it could not be done, it is just not reported in the original publication.

It has to be mentioned that sometimes different publications use different terms for the very same transformation. For the sake of simplicity, in this review each transformation has the same name in all entries. For example, the coupling of olefins with arenes to give alkylated arenes is either classified as alkylation or hydroarylation in literature. It was decided for this review to look at the reaction product and see what happened to the part of the molecule which carried the DG. Hence, in case the arene carried the DG, the reaction is always classified as an alkylation reaction.

This review aims at being comprehensive in the regard that all DGs and potential transformations with the DG are listed, however, reporting every single variant of a given transformation would just be beyond the scope of this review. In such cases a selection had to be made by critically looking at the reported protocols and choosing representative examples to give a clear flavour on chemical diversity searched for by the synthetic chemist. It is clear that such a selection will always be biased and readers might find that one or the other example should have been selected differently.

The speed in which new contributions are brought forward in the field is amazing. This shows the high relevance of this area of research and the potential of the field. For writers of a review it brings certain problems as well, most importantly which time frame to cover and which examples to include? As a starting point, we used the very comprehensive review by Zhanxiang Liu and Yuhong Zhang published in early 2015, which covers literature until the end of 2014.^17^ Hence, our review starts at the beginning of 2015 until the end of 2017.

## 2. Ketone DGs

**Fig. 2:**
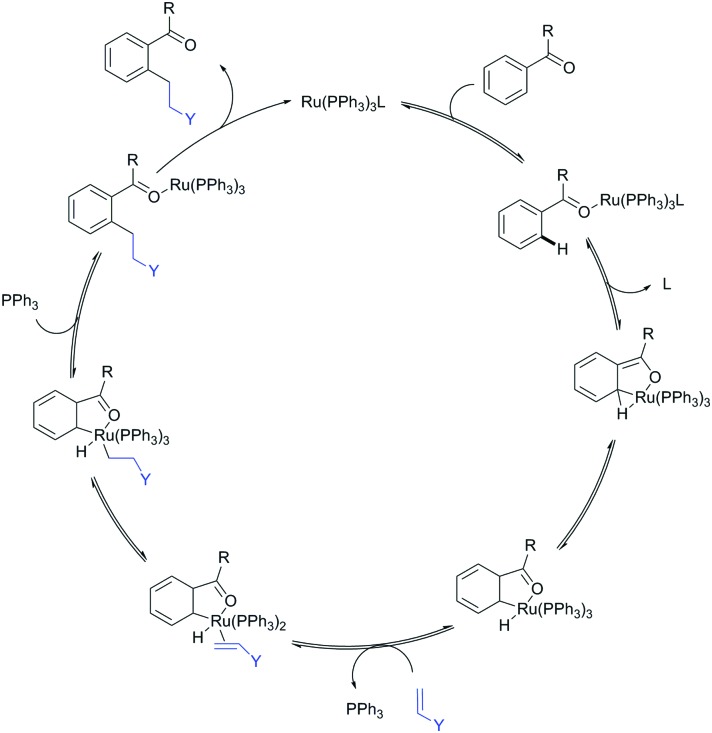
Murai's ketone-directed alkylation.

The carbonyl group in ketones was historically amongst the first DGs to be used in C–H functionalisation chemistry. Already in 1993, a pioneering work for ketone-directed alkylations was published by Murai *et al.*^35^ In this landmark contribution, the first highly efficient and selective C–H cleavage with a simultaneous C–C bond formation mediated by a Ru complex on different aromatic ketones (*e.g.* naphtyl, furan, thiophene) with mono- and di-substituted olefins was shown. The mechanism proposed in this paper served as blueprint for many more contributions to come, and can be considered as one of the most important starting points for the field of C–H functionalisation chemistry, as we experience it today. It was proposed that the carbonyl function first pre-coordinates the metal catalyst, which brings it into a position in close proximity to the C–H bond *ortho* to the ketone function (Fig. 2). This allows C–H insertion of Ru into the C–H bond. Then, one PPh_3_ ligand is replaced by the olefin, first *via* π- then *via* σ-bonding. Finally, reductive elimination forms the new C–C bond and the catalyst dissociates again.

25 years later, the field is still very active and new contributions are brought forward continuously and even though the field has been recently reviewed,^75^ a significant number of new contributions has been published, which will be treated in this section. This is certainly due to the manifold of opportunities of further manipulation a ketone allows, and also the ease of introduction *via* various methods, *e.g.* Friedel–Crafts acylation. From the very beginning, Ru proved to be especially well suited for ketone-directed C–H activation reactions followed by Rh, with other metals playing only a minor role.

### 2.1. Arylation

**Scheme 1:**
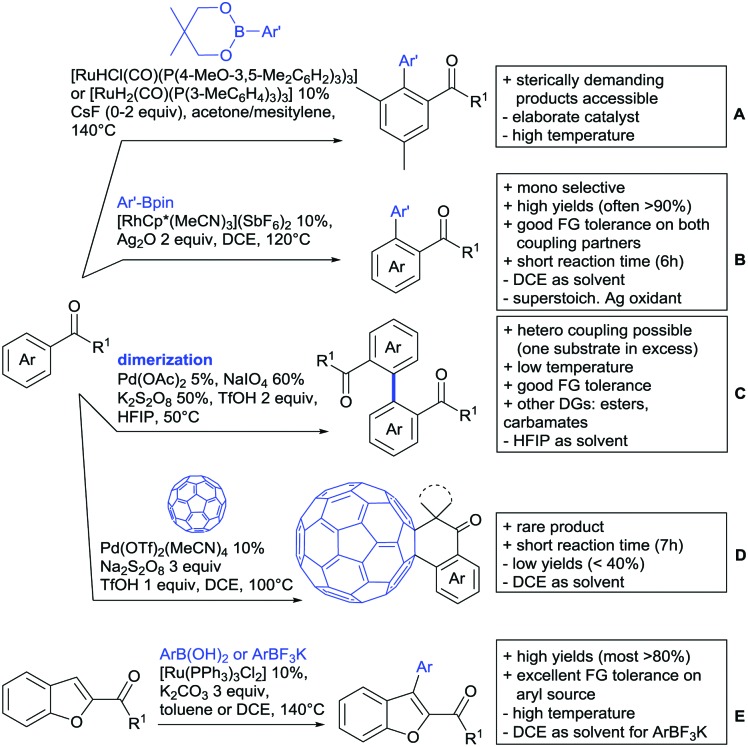
Ketone-directed arylations.

Kakiuchi and co-workers tackled the problem of arylating sterically demanding diaryl and aryl–alkyl ketones by designing Ru catalysts of the general structures [RuHCl(CO)(PAr_3_)_3_] and [RuH_2_(CO)(PAr_3_)_3_].^76^ 3,3′,5,5′-Tetramethylbenzophenone served as model substrate and a boronic acid ester as the aryl source. For this substrate, [RuHCl(CO)(P(4-MeO-3,5-Me_2_C_6_H_2_)_3_)_3_] turned out to be the best performing catalyst, whereas for the pivalophenone derivative 1-(3,5-dimethylphenyl)-2,2-dimethylpropan-1-one [RuH_2_(CO)(P(3-MeC_6_H_4_)_3_)_3_] performed best (Scheme 1A).

Again using boronic esters as aryl sources, Lu and Sun developed a Rh catalysed protocol for arylating alkyl–aryl ketones exclusively at the C(sp^2^)–H bond.^77^ Di-arylated products were not observed, even in the absence of steric bulk. Additionally, yields were generally excellent and frequently >90% (Scheme 1B).

Rao reported on a dimerization of aromatic ketones leading to 2,2′-dicarbonylated biaryls *via* a Pd(ii)–Pd(iv) catalytic cycle (Scheme 1C).^78^ Substituted benzophenone derivatives and also alkyl–aryl ketones worked well towards the homodimers. More importantly, the authors showed that also two different ketone substrates can be applied leading to heterocoupling products in yields between 61–82%. In these cases, the more electron-rich ketone had to be used in excess. This is a significant improvement to traditional routes towards the same type of products.^79^,^80^ Furthermore, the method was not limited to ketones as DG but also esters and carbamates were applied.

An example which can be counted to arylation reactions as well is the synthesis of [60]fullerene-fused tetralones *via* a Pd catalysed protocol.^81^ [60]Fullerene reacted with a series of alkyl–aryl ketones. Even though the yields were low (10–31%), this is an interesting example showing the diversity of possible coupling partners (Scheme 1D).

Ramana investigated the C3-arylation of 2-arylbenzofurans using either arylboronic acids or the corresponding potassium trifluoroborates as the aryl source.^82^ Almost the same conditions as for their alkylation protocol could be used (see Section 2.2) but interestingly AgOAc had a detrimental effect and only in its absence, high yields were obtained and also excellent FG tolerance was observed (Scheme 1E).

### 2.2. Alkylation

**Scheme 2:**
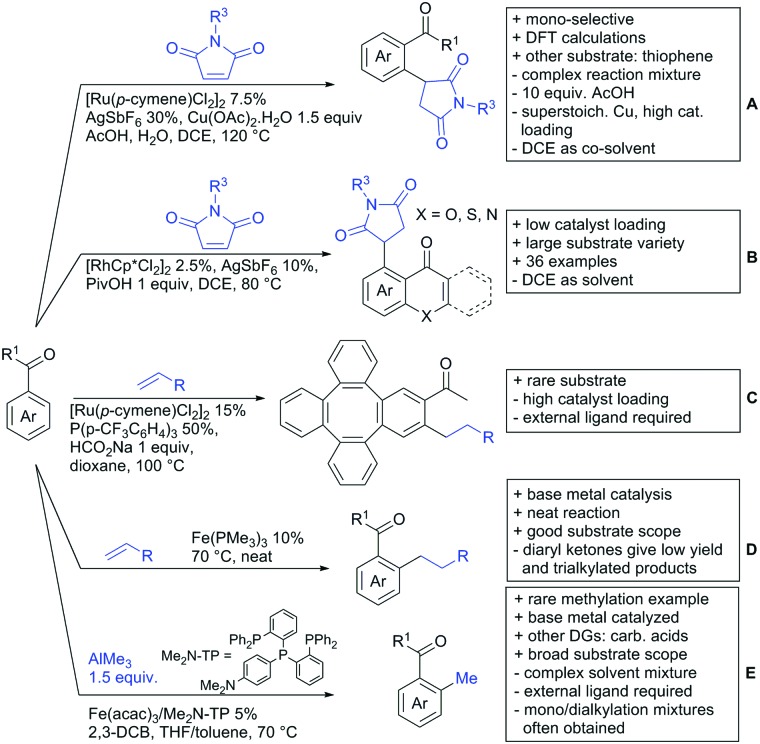
Ketone-directed alkylations.

In 2015, the group of Prabhu reported on the addition of maleimides mainly to acetophenones, which is formally an alkylation reaction (Scheme 2A).^83^ Also 2-acetylthiophene was a suitable starting material, however with a decreased yield of 54%, whereas two benzophenone derivatives were as efficient as the acetophenones. Noteworthy are the DFT studies in this contribution, which strongly favour a CMD mechanism over an electrophilic aromatic substitution or a mechanism involving an oxidative addition. One year later, Kim and Kim reported a similar protocol applied to chromones and xanthones.^84^ By switching to a Rh catalyst, they could reduce the catalyst loading significantly (Scheme 2B). Acetyl-directed alkylation (as well as amidation and alkenylation) was also reported by Zhang (Scheme 2C).^85^ Relatively high catalyst and ligand loadings were required but on the upside, an uncommon substrate, tetraphenylene, was used in this transformation.

Recently, an Fe catalysed alkylation protocol was published by Kakiuchi and co-workers (Scheme 2D).^86^ Using simple Fe(PMe_3_)_4_ as catalyst without any additional ligand or other additives, alkyl–aryl ketones were alkylated with substituted olefins (mainly alkenyl silanes) in excellent yields under neat conditions. Also cyclic alkyl–aryl ketones (*e.g.* 2,2-dimethyltetralone) could be used as substrates. Diaryl ketones did not react as efficiently and required higher catalyst loading and trialkylation was observed as the major product. The proposed mechanism followed the initial suggestion by Murai.^35^

One rare example of *ortho* methylation was recently disclosed by Ilies and Nakamura (Scheme 2E).^87^ AlMe_3_ served as alkyl source and simple Fe(acac)_3_ as catalyst. However, the addition of a less common ligand, Me_2_N-TP (see Scheme 2E) was necessary. As usual in most of such alkylation reactions, mixtures of mono- and di-alkylated products were obtained if permitted by the substrate structure. In the same paper also carboxylic acids were used as DGs (see Section 4).

**Scheme 3:**
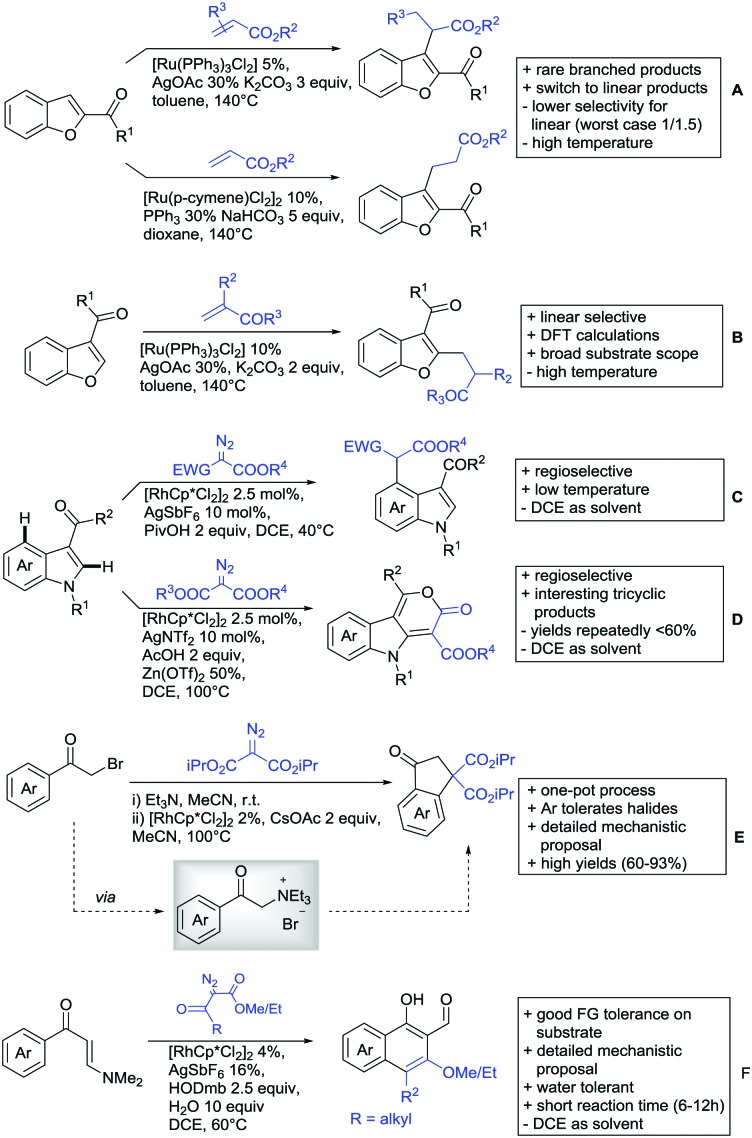
Ketone-directed alkylation on carbo- and heterocycles.

All examples so far gave exclusively the synthesis of linear products. However, Ramana and co-workers showed that tuning the catalytic system allowed to switch between linear and branched products in the C3-alkylation of 2-aroylbenzofurans with acrylates (Scheme 3A).^88^ Whereas the selectivity for branched products was generally in the 9/1 (branched/linear) range, for linear products it was often significantly lower. Recently, the group published a follow-up paper trying to elucidate *via* DFT calculations how the linear/branched selectivity was controlled.^89^ They calculated transition states leading either to the linear or to the branched products, which are both feasible pathways. The transition state towards the branched product turned out to be significantly lower in energy, which goes in line with the experimental observations. Furthermore, the method was extended to the C2-alkylation of 3-aryloxybenzofurans.^89^ On this second type of substrates, the linear product was preferred with high selectivity (20/1) and no protocol for the branched products was disclosed (Scheme 3B).

The very same reagent was also used for another functionalisation/cyclisation sequence using 3-ketoindoles as substrates (Scheme 3C and D).^90^ Initially, the authors found two products, the one alkylated in C4, and one derived from alkylation and subsequent cyclisation in C2. At the beginning, they could successfully optimise the reaction towards selective formation of the C4 alkylated products with good to excellent yields (36–98%). Subsequently, also the second type of products could be obtained selectively, however, generally in lower yields (∼10% lower) for similar examples. In both cases, [RhCp*Cl_2_]_2_ was used as the catalyst, but the additives differed, as can be seen in Scheme 3C and D.

An interesting example has been reported by Wan and Li.^91^ Using phenylacyl ammonium salts as substrates (which can also be produced *in situ*) in combination with α-diazo esters led to benzocyclanone products (Scheme 3E). Key was the unprecedented presence of an oxidizing C–N bond in the substrates which allowed this overall redox-neutral process. Detailed mechanistic studies were carried out, including DFT calculations.

**Scheme 4:**
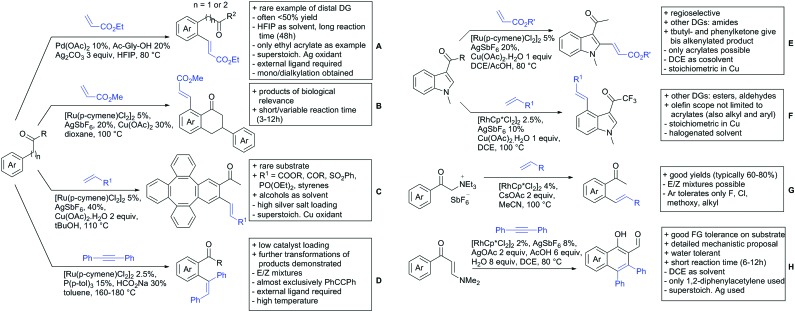
Ketone-directed olefinations on various substrates.

The group of Zhu used enaminone starting materials for the synthesis of naphthalene derivatives using either α-diazo-β-ketoesters (Scheme 3F) or alkynes (Scheme 4H) as coupling partners.^92^ In case of α-diazo-β-ketoesters as coupling partners the initial step is an alkylation and good FG tolerance on the substrate was observed. Synthetically useful yields between 50–85% were reported and various alkyl residues including cyclopropyl can be in position R^2^.

### 2.3. Alkenylation

Yu explored the application of distal weakly coordinating DGs, including ketones (Scheme 4A).^93^ In this report, the ketone functionality is not directly attached to the aromatic ring but separated by a –CH_2_–, or in one case a –CH_2_CH_2_– spacer. Yields for the reaction with acrylates are moderate and selectivity between mono- and di-alkenylation is an issue.

Bakthadoss *et al.* showed that basically the same reaction conditions could be applied for the alkenylation of chromanones as well, in this case in dioxane as solvent (Scheme 4B).^94^ Yields were commonly good (60–81%) and the typical range of FGs was tolerated.

Again on tetraphenylenes, Zhang also reported alkenylation reactions (Scheme 4C).^85^ In this case, the catalyst loading could be significantly reduced and no ligand was required, although 2 equiv. of Cu had to be used. Besides acrylates, also alkenyl sulfoxides and -phosphates as well as styrene derivatives could be used.

Szostak's group showed the ketone-directed hydroarylation of internal alkynes leading to alkenylated arenes (Scheme 4D).^95^ A low Ru loading could be applied; however the *E*/*Z* ratio left room for improvement. In this contribution, the DG showed significant variation as opposed to many contributions which relied primarily on the acetyl group. Further elaboration of the obtained alkenylated products to a series of useful compounds was also demonstrated.

Prabhu and co-workers showed that a ketone DG in position 3 of indoles leads to alkenylated products either at the C2- or C4-position.^96^ The source of this regioselectivity is primarily the electronic nature of the DG. For R = Me/Et, alkenylation took place in C2-position under Ru catalysis (Scheme 4E).^96^ Also an amide DG gave the same regioselectivity. In case of R = *t*-butyl, di-alkenylation in C2- and C4-position was observed and for R = phenyl di-alkenylation in C2-position and on the DG occurred. For R = CF_3_ and using a Rh catalyst, exclusive C4 alkenylation was observed (Scheme 4F).^96^ Trying to elucidate the origin of this regioselectivity, the authors have reason to believe that COCF_3_ is the weaker coordinating DG, which eventually does not direct C–H activation by precoordination but rather stabilizes a metal–arene σ complex that forms due to electrophilic metallation. An ester or an aldehyde as DG also gave C4 alkenylation. Such examples, giving guidelines for achieving different regioselectivity, are of significant importance for further promoting the application of C–H functionalisation in complex syntheses.

Another peculiar ketone DG was developed by Lan and Li, namely phenylacyl ammonium salts (Scheme 4G).^91^ Using a Rh catalyst, *ortho* alkenylation was obtained in good yields with styrene derivatives. However, also alkyl substituted olefins could be applied with the drawback that sometimes, mixtures of *E* and *Z* isomers were obtained. It is noteworthy that the ammonium group was cleaved during the reaction, overall leading to *ortho* alkenylated acetophenone derivatives. Noteworthy, the reaction does not require an external oxidant since the ammonium salt of the DG takes over this role.

The group of Zhu used enaminone starting materials for the synthesis of naphthalene derivatives using either alkynes (Scheme 4H) or α-diazo-β-ketoesters (Scheme 3F) as coupling partners.^92^ In the case of alkynes, only 1,2-diphenylacetylene was used as reaction partner but good FG tolerance on the substrate was observed and synthetically useful yields between 50–85% were reported. According to the proposed mechanism, the initial step is a Rh-catalysed alkenylation.

### 2.4. Alkynylation and acylation

**Scheme 5:**
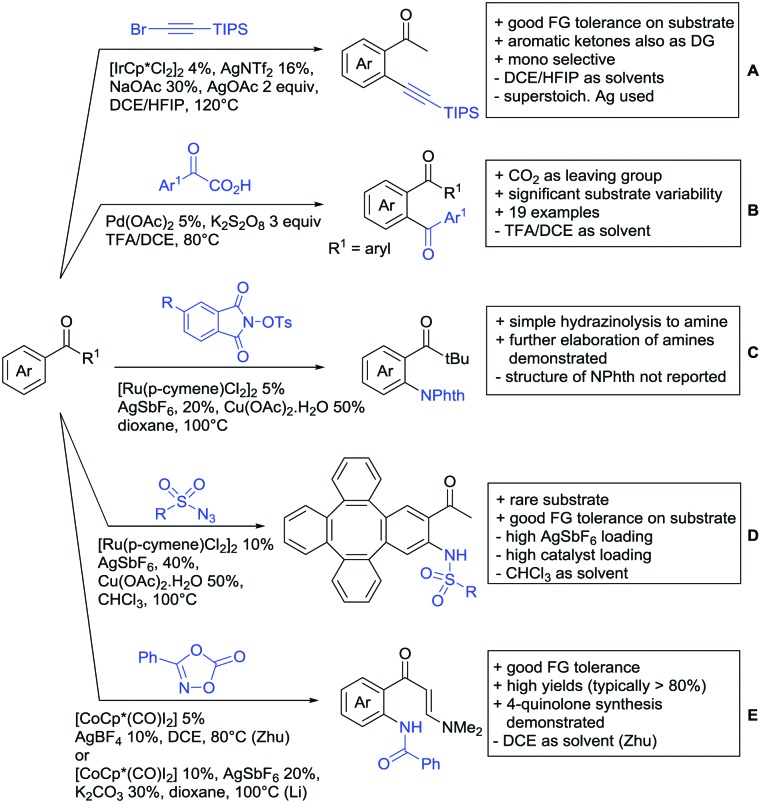
Ketone-directed alkynylation, acylation and C–N bond formation.

One example of an alkynylation reaction was reported by Jiang and Huo (Scheme 5A).^97^ TIPS protected bromo acetylene was used as alkyne source under Ir(iii) catalysis. Aromatic and heteroaromatic ketones worked equally well and a range of substituents were tolerated with good yields (generally ∼70%). On the downside was the large amount of a mixture of three different Ag salts and the requirement for halogenated solvents. Esters can be used as alternative DGs, although the ketone showed a better directing effect, whereas an amide or pyrazole DG were superior to the ketone. Such studies, allowing the ranking of directing ability of different DGs, are unfortunately rare but extremely important for the application of C–H functionalisation to the synthesis/modification of complex molecules, carrying many FGs.

Decarboxylative acylation was reported by Yu and co-workers (Scheme 5B).^98^ As acyl source, α-oxocarboxylic acids were used under Pd catalysis. The authors suggested an acyl radical as important intermediate, which was supported by a radical trapping experiment with TEMPO.

### 2.5. C–N bond formation

Ackermann disclosed Ru catalysed imidation of aryl–*t*butyl ketones using *N*-tosyloxyphthalimides (Scheme 5C).^99^ The obtained products were easily transformed to the corresponding amines using standard conditions (N_2_H_4_), which gave valuable 2-amino-phenones. Those can be converted to a series of nitrogen containing heterocycles.

Using aromatic or aliphatic sulfonyl azides, it was again Zhang who showed amidation of terpenylenes with high FG tolerance (Scheme 5D).^85^

Dioxazolones became popular amidating agents in recent years in the field of C–H activation. Interestingly, two groups almost simultaneously exploited this reagent on enaminones. Zhu and co-workers developed a Co(iii)-catalysed protocol,^100^ whereas Li developed Co(iii)-and Rh(iii)-catalysed reactions.^101^ The two Co(iii)-catalysed protocols used both [Cp*Co(CO)I_2_] as catalyst (Scheme 5E). The substrate scope and yield (typically >80%) were comparable and all common FGs seemed to be tolerated. The amidated product was then transformed to 4-quinolones under acidic conditions (not shown).

## 3. Aldehydes as DGs

Aldehydes are frequently applied in C–H functionalisation chemistry in case of the formation of so-called “transient” DGs, where a DG is formed and cleaved *in situ*. A prominent example is the formation of imines from aldehydes (or ketones) and amines. Since this subject has been reviewed very recently,^102^,^103^ this type of chemistry is not included in this chapter and only examples where the aldehyde function is the actual DG are discussed. Hence, in the timeframe covered by this review, only three examples have been reported and only in one example the aldehyde function remains in the final products, whereas in the other two examples it acts as traceless DG.

**Scheme 6:**
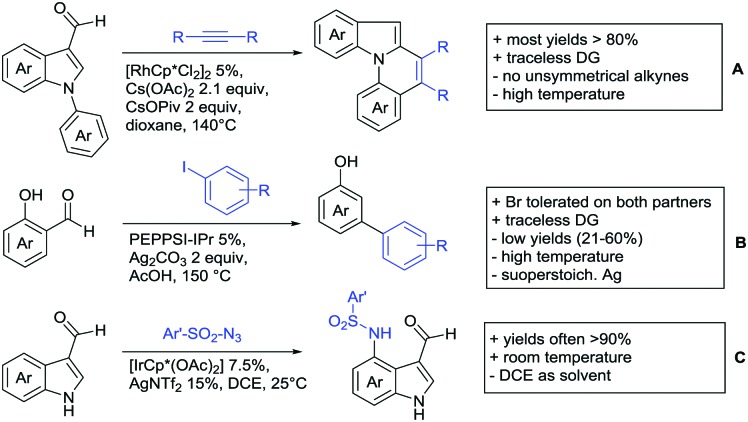
Aldehyde-directed C–H functionalisation.

The first example used the aldehyde function as a traceless DG in a Rh catalysed reaction with alkynes (Scheme 6A).^104^ 2-Aryl-3-formylindoles were used as starting materials. In the first step (according to the proposed mechanism), the aldehyde-directed an alkenylation at C2-position of the indole core. Rh was then suggested to be involved in the decarbonylation process and further activation of a C–H bond of the *N*-aryl group as well leading overall to indolo[1,2-*a*]quinolones in high yields (up to 94%, frequently >80%), which is overall an elegant access to this system.

Also in the second example, the aldehyde DG was removed under the applied reaction conditions, this time in an arylation protocol (Scheme 6B).^105^ Initially, salicylaldehyde was used as the starting material and aryl iodides as coupling partner. Arylation took place *ortho* to the formyl group, which was cleaved under the applied reaction conditions leading overall to *meta* substituted phenols in yields around 50%. Regarding the mechanism, experiments actually pointed towards a reaction sequence in which the first step is the oxidation of the salicylaldehyde to salicyclic acid (which would mean that the actual DG is the acid function), which is then removed *via* decarboxylation. The advantage of starting from the aldehyde function is that salicylic aldehyde derivatives could be prepared much more easily from the corresponding phenols than salicylic acids.

The one example keeping the aldehyde function reported sulfamidation of indoles in position 4 (Scheme 6C).^106^ This Ir catalysed method could be run at room temperature and delivered typically high yields of the desired products. Noteworthy, the application of the method to a gram scale synthesis was reported showing the scalability of this transformation.

## 4. Carboxylic acids as DG

Even though carboxylic acids are a frequently occurring FG, it has been added rather late to the repertoire of DGs. The majority of contributions regarding carboxylic acid-directed *ortho* C–H functionalisation were only made since the late 2000s and the field has been recently reviewed.^107^,^108^

The benefits of carboxylic acids as DGs are quite striking as they are naturally abundant and relatively easy to introduce. They can either be cleaved or further transformed *via* decarboxylative coupling reactions, often possible to perform *in situ* or in one-pot fashion.^109^,^110^ Furthermore, the possibility to remove the group catalytically led to the development of its use as a traceless DG.^111^ However, carboxylates are usually weakly coordinating to the catalyst and are often disfavoured in comparison to stronger DGs (*e.g.* pyridines, bidentate amides). They can also undergo undesired side reactions such as hydrodecarboxylation as these are also catalysed by transition metals,^112^ which is an additional difficulty in developing carboxylic acid-directed C–H functionalisation processes.

### 4.1. C(sp^2^)–H activation

#### 4.1.1. Arylation

Within this section, the recent advances in directed *ortho* arylation reactions are divided into: (i) arylations keeping the carboxylic acid moiety intact, (ii) arylations with carboxylates as traceless DGs, and (iii) methods in which the arylated product undergoes a consecutive ring-closing reaction with the carboxylate DG. Before 2015 arylations were widely catalysed by Pd, with other metals applied only scarcely.^17^ Recent years showed more and more other transition metals such as Rh, Ir or Ru becoming of greater importance.

**Scheme 7:**
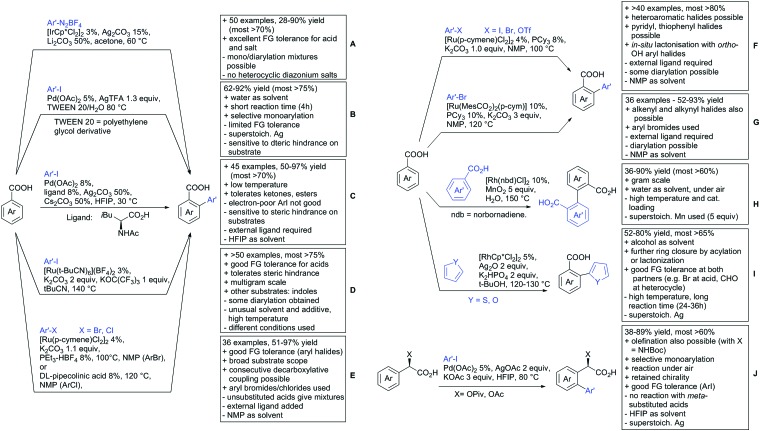
Arylation with carboxylic acids as DG.

For example, the group of Gooßen developed a protocol using aryldiazonium salts as aryl sources, which was catalysed by Ir and proceeded at a mild temperature of 60 °C (Scheme 7A).^113^ Additionally, only a sub-stoichiometric amount of base and Ag salt was needed and acetone was used as solvent. Notably the weakly coordinating carboxylic acid controlled the regioselectivity even if “stronger” DGs are present (*e.g. o*-acetamide). The regioselectivity was greatly influenced by steric factors. If *meta* substituted acids were the substrates, the easier accessible position was arylated exclusively and if both *meta* positions were occupied, no conversion took place. The parent benzoic acid gave a 1 : 1 mixture of mono- and di-arylated product. The transformation converted a wide range of different diazonium salts. However, heterocyclic salts did not react.^113^

Coupling with aryl halides was achieved with both Pd (Scheme 7B and C) and Ru (Scheme 7D–G). Ren (Scheme 7B) reported a selective mono-arylation with aryl iodides in water (with the use of TWEEN 20 as a surfactant).^114^ The yields were generally good to excellent. However, some sensitivity to steric hindrance in *ortho* substituted benzoic acids was observed. For instance, in comparison to 4-chlorobenzoic acid (88% yield), the 2-chloro acid reacted more slowly, and only 73% yield was reported.

Weiping Su reported another Pd catalysed arylation^115^ (Scheme 7C). The reaction used a mono-protected amino acid (MPAA) as a ligand, proceeded at room temperature and tolerated a broad range of substrates. However, this reaction was more sensitive to substitution in the *ortho* position to the acid moiety. Even the relative small methyl group reduced the yield by 20% compared to its *para* and *meta* regioisomers. The authors stated that the formation of the palladacycle might be prevented due to steric reasons, which also explains the exclusive monoarylation of this transformation.

Ru-Catalysed approaches were investigated by several groups (Scheme 7D–F). For example, Larrosa reported an arylation using aryl iodides in *t*-BuCN as a solvent (Scheme 7D).^116^ Several different aryl iodides were used as reactants in this protocol. Generally, *para* and *meta* substituted iodides gave better yields and electronic properties seemed to have a minor influence on the reactivity. Acids substituted in *ortho* position gave usually excellent yields. Remarkably, 3,6-disubstituted benzoic acids provided the arylated products as well, despite the considerable steric hindrance. However, if *meta* substituted acids were used 3.0 equiv. of water had to be added and di-arylation became an issue. Its extent depended mainly on steric congestion of the substituents. *para* substituted acids gave the di-arylated products exclusively. The protocol was also applicable to indole carboxylic acids in various positions and even in certain cases without any protection of the indole nitrogen.

**Scheme 8:**
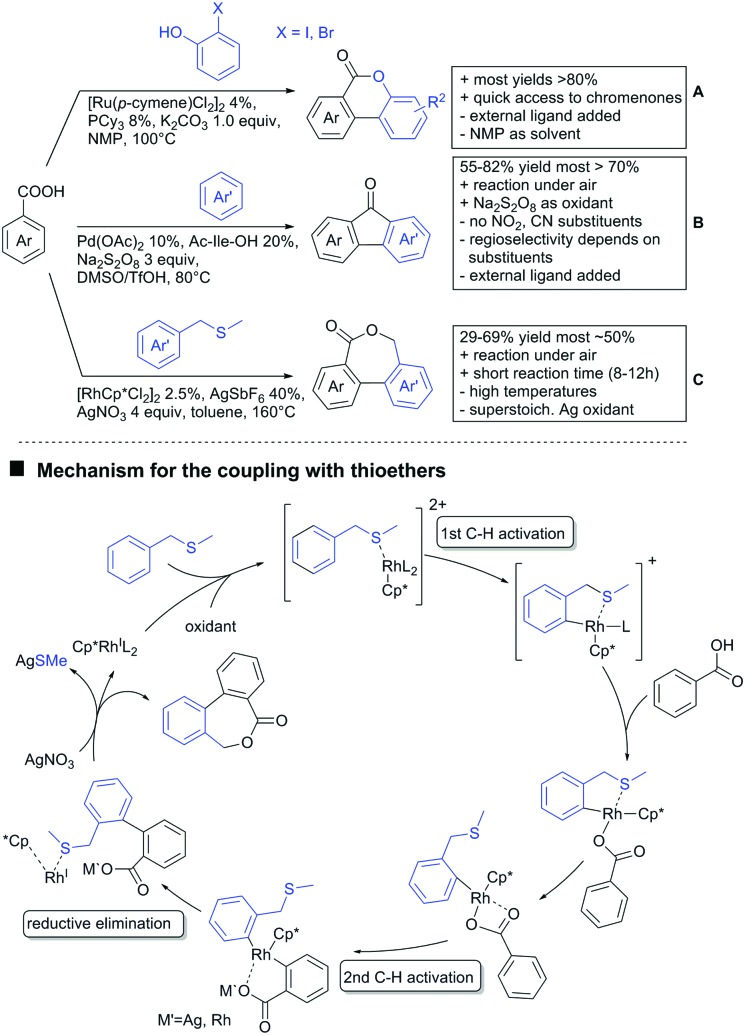
Annulation reactions.

With the help of Ru catalysis, significant improvement was made in making aryl bromides and chlorides applicable for this kind of chemistry: Gooßen developed a catalytic system with [Ru(*p*-cymene)Cl_2_]_2_ and PEt_3_·HBF_4_, which gave good yields with aryl bromides as substrates (Scheme 7E).^117^ The corresponding chlorides could be transformed as well, when pipecolinic acid was used as ligand instead of triethyl phosphine. Both protocols converted a broad range of different substrates. Synthetically useful was as well the possibility to protodecarboxylate the product *in situ* using a Cu catalyst. A very similar system by Weix (Scheme 7F) used aryl iodides (and bromides) as substrates.^118^ Even a broad scope of heteroaryl halides was reported for this transformation. Furthermore, when 2-iodophenols were used as substrates, an additional lactone formation offered an access to benzochromenones (see also Scheme 8A). Interestingly in both protocols, ketones or amides did not act as competing DGs, which opened possibilities for consecutive C–H functionalisation.

Ackermann reported a protocol with PCy_3_ as ligand and ([Ru(MesCO_2_)_2_(*p*-cymene)]) as precatalyst (Scheme 7G).^119^ Arylation with the corresponding aryl bromides proceeded smoothly. Additionally, alkenes and alkynes were suitable substrates to achieve alkenylations/alkynylations similar to the transformations shown in the previous section. Both methods used NMP as solvent and K_2_CO_3_ as base.

All mentioned Ru catalysed protocols did not need any halogen scavenger (*e.g.* Ag salt) to facilitate the reaction (Scheme 7D–G). However, di-arylation could not be suppressed by neither of the protocols, which made the use of *ortho* substituted acids mandatory. This is in contrast to the Pd catalysed reactions (Scheme 7B and C). In such cases if a *meta* substituent at the carboxylic acid was used, the reactions proceeded regioselectively due to steric reasons.

In the field of oxidative C–H activations, transformations with the retention of the DG and also annulations were reported (Schemes 7H, I and 8A–C). Chao-Jun Li reported an cross-dehydrogenative Rh catalysed homocoupling of benzoic acid derivatives.^120^ The protocol was later improved to achieve heterocoupling between electron-rich and electron-poor acids (Scheme 7H).^121^ Another cross-dehydrogenative C–H activation developed by You^122^ used thiophene and furan derivatives as coupling partners (see Scheme 7I). The reaction was again catalysed by a Rh species and showed good FG tolerance. Notably, a subsequent intramolecular Pd catalysed oxidative coupling between the acid and the C3 position of the heterocycle gave the corresponding lactones.

Arylation could also be achieved using phenylacetic acid derivatives under Pd catalysis *via* 6-membered metallacyles. One contribution used –OH protected mandelic acid to functionalise in *ortho* position to the residue (Scheme 7J).^123^ As expected under Pd catalysis, mono-arylation was observed and substrates with *ortho* substituents to the DG failed to react. It is noteworthy that the stereochemistry in case of a chiral substrate is completely retained.

Regarding arylations that are followed by a ring closing reaction, the already mentioned protocol developed in the group of Weix (see Scheme 7F) yielded chromenones if a hydroxy group was present *ortho* to the halide (Scheme 8A).^124^ In the group of Jingsong You an oxidative Pd catalysed arylation, followed by *in situ* ring closure *via* acylation (Scheme 8B) was developed.^125^

A rather unusual double C–H activation reaction also formed polycycles upon lactonization (Scheme 8C).^126^ Quite interestingly, the first C–H activation is actually directed by the sulfur atom of a thioether and only at a later stage of the catalytic cycle coordination to the carboxylic acid occurred.

**Scheme 9:**
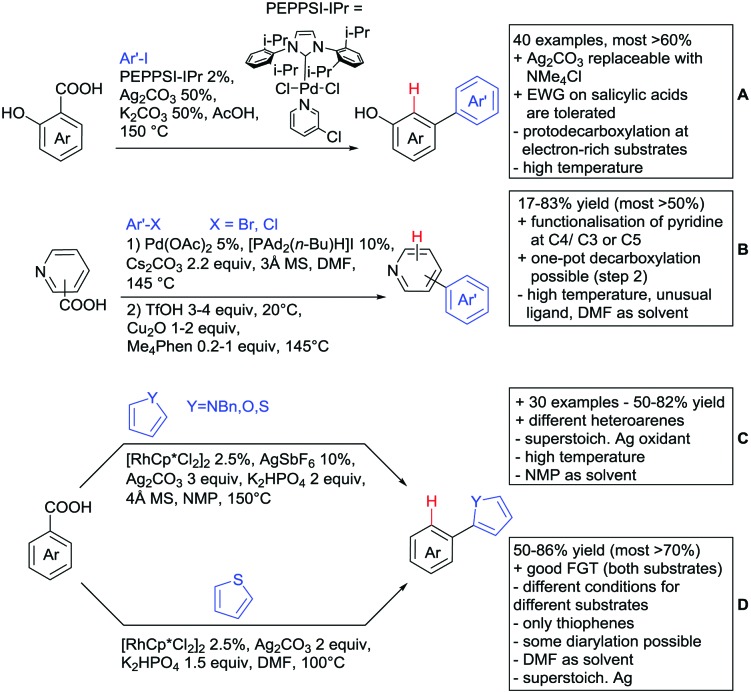
Arylation with carboxylic acids as traceless DG.

As previously mentioned, carboxylic acids could also be applied as traceless DGs. The first (although in a separate reaction after workup) protocol using this idea was developed by Daugulis in 2007.^127^ The development towards a one-pot protocol in 2011 could be seen as the start of the quest towards the application of carboxylic acids as traceless DGs.^128^ This approach is very convenient and enhances the utility of directed C–H functionalisation, since the removal of DGs can be difficult. Recent progress in the traceless carboxylic acid-directed arylations over the last years are summarized in Scheme 9A–D.

The coupling of aryl iodides with salicylic acid derivatives was achieved by the use of PdPEPPSI-IPr as catalyst (Scheme 9A).^129^ Both electron-poor and -rich aryl iodides were good coupling partners in this regioselective protocol, although *meta* substituted iodides were not well tolerated, possibly due to steric reasons. Different salicylic acid derivatives could be converted with good yields. However, very electron-rich substrates gave lower yield, due to protodecarboxylation. Industrially appealing is the option to substitute the sub-stoichiometric amount of Ag salt with environmentally more benign tetramethylammonium chloride. In a previous study the same group showed that the typically required Ag salts, which are regenerating the catalyst after the catalytic cycle, can be replaced with a quaternary ammonium salt.^130^

Notably, pyridine carboxylic acids could also be functionalised (Scheme 9B), which is remarkable since C–H functionalisation on electron-poor heterocycles is rarely reported.^131^ The protocol was applicable with retention of the carboxylic acid. However, a decarboxylative protocol (Cu catalysis) was also reported in the same contribution. A competing experiment with 6-phenylpyridine-3-carboxylic acid showed that the carboxylic acid overrules the DG ability of pyridine. The compound is arylated in 4-position (43%) and only small amounts (4%) of arylation at the phenyl moiety were observed.

Oxidative heteroarylation (mainly with thiophenes) was established with [RhCp*Cl_2_]_2_ as catalyst and Ag_2_CO_3_ as oxidant (Scheme 9C and D).^132^,^133^ Both methods showed a good substrate scope, including nitriles, acetamides or halides, but differed in their use of additives and solvent.

#### 4.1.2. Alkylation and allylation

**Scheme 10:**
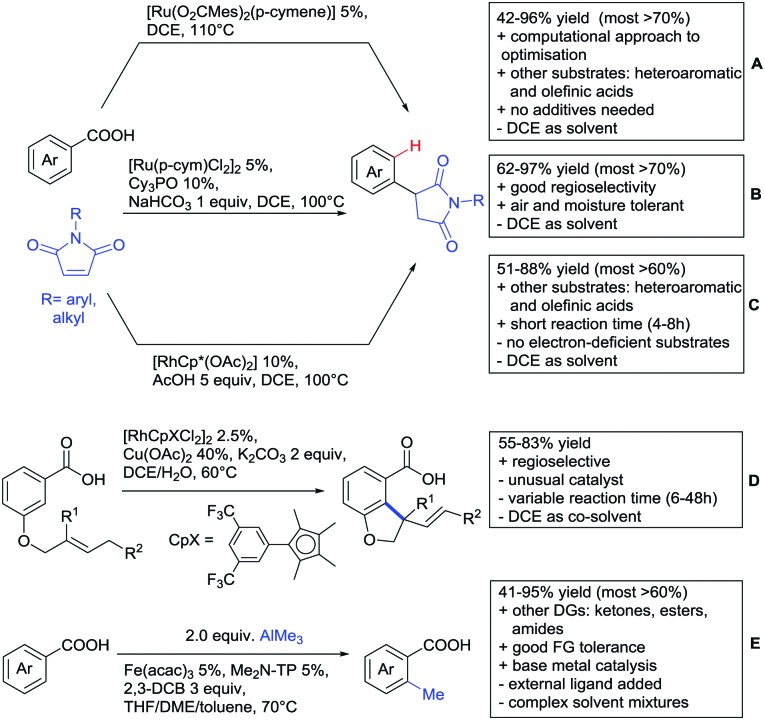
Alkylation and allylation reactions directed by carboxylic acids.

The addition to maleimides with the carboxylate as traceless DG has been reported rather recently. Ackermann and co-workers used a Ru carboxylate catalyst for this transformation (Scheme 10A).^134^ The reaction conditions were optimised with the support of DFT calculations. They commenced their theoretical studies with the Ru catalysed alkenylation with alkynes (Scheme 13B–D). In contrast to the already mentioned computational study by the group of Hong^135^,^136^ their calculations showed no significant solvent effect of this reaction. They continued the study towards the alkylation with maleimides for which they calculated also a negligible solvent effect. The reaction tolerated several FGs (CN, F, Br, I, NO_2_) and also heteroaromatic acids were converted. The scope included several *ortho*- and *para*-substituted acids, which gave only one product. Additionally, one example with a phenyl residue *meta* to the acid was given. In this case, functionalisation in C6-position occurred exclusively. Additionally, also cyclohexenecarboxylic acid and different acrylic acids were convertible by this transformation (61–81% yield, Scheme 10A).^134^ Baidya also used a Ru catalyst for the same transformation, obtaining very similar results regarding yields, scope and regioselectivity (Scheme 10B).^137^ Interestingly with [Ru(*p*-cymene)Cl_2_]_2_ as precatalyst, the addition of base is necessary. A Rh catalysed protocol was also reported (Scheme 10C).^138^ This rather fast reaction also worked on substituted acrylic and heteroaromatic acids as substrates. However, no electron-deficient acids were reported. This time the addition of acetic acid to the reaction mixture was necessary. Interestingly, the three mentioned protocols differed in their need of acid or bases. Whereas in the first transformation (Scheme 10A) neither acid nor base was needed. The other two needed either base (Scheme 10B) or acid (Scheme 10C) in the reaction mixtures.

Quite interesting was an intramolecular allylation (Fujiwara–Moritani reaction) catalysed by an electron poor Rh complex (Scheme 10D).^139^ Presumably, the carboxylate-directed the *ortho* C–H functionalisation.

The ketone-directed methylation reported in Scheme 2 was also effective using carboxylic acids (Scheme 10E).^140^ This rare example of Fe catalysis proceeded under mild conditions with good FG tolerance. Furthermore, this transformation was also applicable with esters and amides.

#### 4.1.3. Alkenylation

**Scheme 11:**
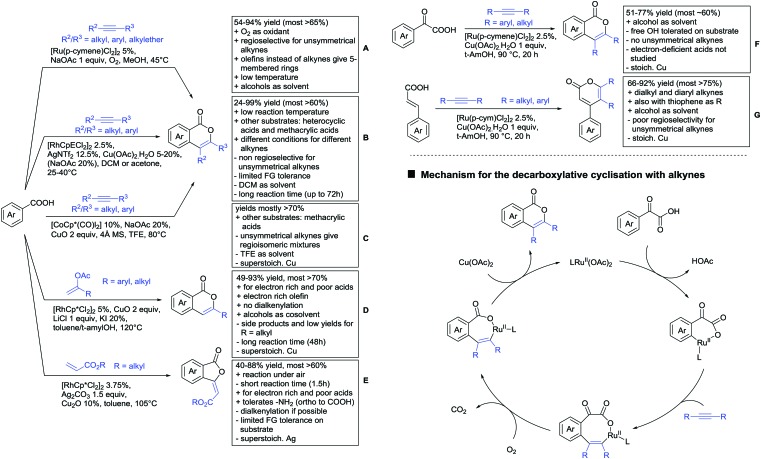
Alkenylations directed by carboxylic acids to access lactones.

In the area of alkenylation reactions, two main reaction types can be distinguished: (i) oxidative reactions with olefins or alkynes followed by annulation to yield the corresponding lactones, typically employing Ag or Cu as oxidants (Scheme 11)^141^ (ii) secondly, oxidative couplings with olefins without annulation (Scheme 13A and E–G), and (iii) hydroarylation reactions with alkynes under redox-neutral conditions (Scheme 13B–D).^142^

Major advances were made in the use of Ru catalysis and also a reaction with Co was published. Ackermann and co-workers reported a quite interesting example of a mild reaction, that used only oxygen as oxidant (Scheme 11A).^143^ The transformation gave good to excellent yields with good FG tolerance at 45 °C in methanol. Importantly, unsymmetrical alkyl–aryl alkynes gave a single regioisomer placing the alkyl group in the C4 position of the final product.

A Rh catalysed annulation developed by Tanaka also proceeded under very mild conditions (Scheme 11B).^144^ However, the use of DCM made this protocol environmentally less attractive. The authors classified the used alkynes into three groups depending on their coordination ability to the catalyst and developed three different protocols accordingly. They differ in the amount of Cu salt and the use of different additives and solvent. These variations enlarge the scope of applicable alkynes and might make the protocol applicable in the synthesis of more complex molecules and can be used as a guideline for a development of specific reaction conditions. Despite the low temperature, the reaction showed limited FG tolerance on the substrates and gave regioisomeric mixtures if unsymmetrical alkynes were used. Notably, though, the reaction also worked with heteroaromatic acids and methacrylic acids as substrates.^144^

The first carboxylic acid-directed C–H functionalisation with (mainly) diaryl alkynes catalysed by Co was reported by Sundararaju (Scheme 11C).^145^ Different acids could be converted with good to excellent yields (61–92%). Unsymmetrical alkyl–aryl alkynes gave regioisomeric mixtures with a preference for the isomer with the alkyl residue in C4-position and the aryl moiety in C3-position of the products. Quite interestingly, if a strong chelating group such as an azaindoyl moiety was installed at the *para* position to the carboxylic acid, the directing effect was still ensured by the acid. Similar to the Rh catalysed annulation by Tanaka (Scheme 11B), the protocol also converted methacrylic acids with good yields. Notably, unsubstituted acrylic acid could also be converted, albeit with low yield (33%).

One example for the formation of isocoumarines without the use of alkynes, was reported by Ting-Bin Wen 2016 (Scheme 11D).^146^ This protocol utilises vinyl acetates and is a rare example of a C–H functionalisation with electron-rich olefins. Both electron-rich and -poor carboxylic acids were tolerated and no di-alkenylated product was observed. The reaction proceeded smoothly with arylvinyl acetates as reactants. However, if alkylvinyl acetates were used, a five membered lactone with an exocyclic double bond (*Z* selective) was observed as a side product in considerable amounts (not shown).

The same Rh species gave the aforementioned 5-membered lactones as the main products, if electron-deficient olefins were used instead. Acrylic esters were transformed regioselectively with moderate to good yields into 5 membered lactones with exocyclic olefins with *E* selectivity (Scheme 11E).^147^ Although dialkenylation did not occur, *ortho* unsubstituted acids gave usually lower conversions.

Both mentioned protocols used stoichiometric amounts of Cu or Ag salts as oxidants, which is clearly a disadvantage regarding environmental sustainability. An interesting alternative to several transformations regarding the oxidant was reported by the group of Wang.^148^ N–O containing molecules were used as an external oxidant and might be an alternative approach to certain transformations.

A special decarboxylative annulation to six-membered lactones of α-ketoacids with Cu(OAc)_2_ as oxidant was also reported under Ru catalysis (Scheme 11F).^149^ The reported substrate scope included chloro and free hydroxy substituents on the acid moiety. However, only internal symmetric alkynes were reported (alkyl as well as aryl residues).

This reaction proceeds *via* the formation of an initial eight-membered ruthenacycle. After uptake of oxygen, incorporated in the carbonyl group of the product, carbon dioxide is released, and the catalytic Rh(ii) species is reformed by oxidation (Scheme 11, bottom right).

As an example of an activation at a non-aromatic C(sp^2^)–H bond, the formation of α-pyranones was reported by Gogoi and co-workers (Scheme 11G).^150^ The reaction proceeded under Ru catalysis and the β carbon of a cinnamic acid derivative was activated. Subsequent insertion of an alkyne and reductive elimination furnished the six-membered ring. This transformation converted heteroaromatic cinnamic acids as well with good yields and accepted both diaryl as well as dialkyl alkynes. However, the use of unsymmetrical alkynes gave mixtures.

**Scheme 12:**
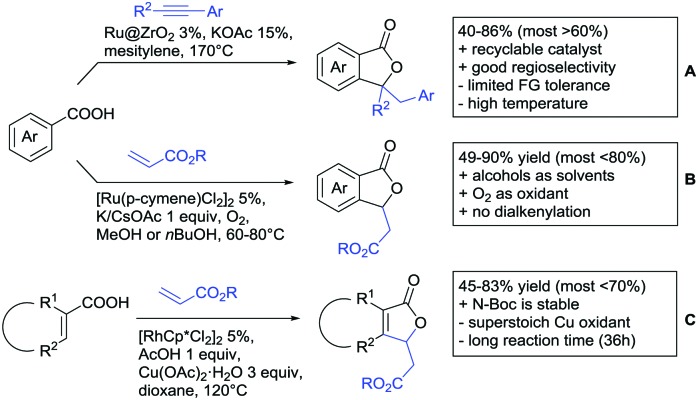
Alkenylation directed by carboxylic acids and subsequent annulation to alkylated lactones.

Under different reaction conditions, saturated lactones can be formed instead of unsaturated ones, depending on the use of oxidant and/or the used coupling partners (alkynes, olefins) of the protocols. As can be seen in Scheme 12, *ortho*-directed alkenylations and subsequent nucleophilic attack of the carboxylate group, followed by protonation, resulted in saturated 5-membered lactones. Shishido reported a transformation with unsymmetrical internal alkynes under ZrO_2_ supported Ru catalysis (Scheme 12A).^151^ Notably, the catalyst could be recycled for at least five runs and the reaction required only a catalytic amount of base. The regioselectivity was generally good. However, not a broad FG tolerance was reported.

Substantially milder reaction conditions were sufficient for the oxidative coupling with acrylates (Scheme 12B).^152^ Under O_2_ atmosphere and a reaction temperature of 60–80 °C, bromo and iodo substituents were tolerated and no di-alkenylated product was observed. A similar cyclisation was also reported by Zhu for cyclic acrylic acids (Scheme 12C).^153^ The reaction was catalysed by a Rh complex and Cu(OAc)_2_ was used as oxidant. Quite interestingly, under the reaction conditions (acidic, metal salts and 120 °C) *N*-Boc protected substrates were converted with moderate yields without any cleavage of the carbamate.

**Scheme 13:**
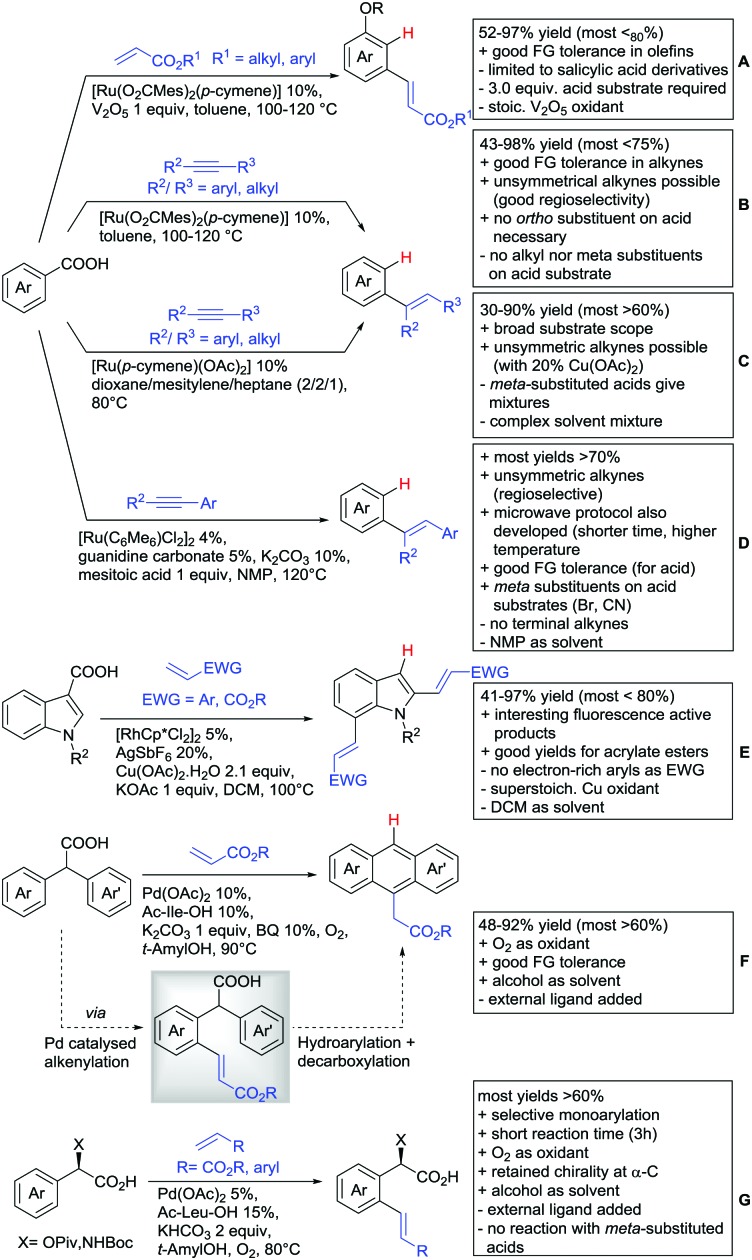
Alkenylation directed by carboxylic acids.

Alkenylation without annulation and subsequent decarboxylation could be achieved with alkenes under oxidative conditions or with alkynes under redox-neutral conditions. These transformations make *meta* substituted aryls more easily accessible if an *ortho* substituted acid is used as substrate and *in situ* protodecarboxylated. In recent literature, alkenylation protocols by the Ackermann^154^ (Scheme 13B), Hartwig/Zhao^155^ (Scheme 13C) and Gooßen^156^,^157^ groups (Scheme 13D) have appeared.

All three protocols used Ru complexes that catalysed the alkenylation as well as the *in situ* decarboxylation. Ackermann also included a report of an oxidative coupling between salicylic acids and electron-poor olefins, using V_2_O_5_ as oxidant (Scheme 13A).^154^ For this transformation, the FG tolerance regarding the olefin was quite good. However, the alkenylation with internal alkynes (Scheme 13B) converted also benzoic acid derivatives and was not restricted to salicylic acids.^154^ Furthermore, examples with aryl and alkyl (also unsymmetrical) alkynes were provided. Interestingly, nitro-, fluoro- and bromo groups *ortho* to the acid were tolerated and gave moderate to good yields. Both *para* and *ortho* residues to the acid naturally gave the corresponding *meta* products. However no example of *meta* substituted acids (to potentially generate the *para* alkenylated product) was reported.

A very similar protocol by Hartwig and Zhao (Scheme 13C) demonstrated a broad substrate scope.^155^ The same substitution pattern regarding the acid was reported with comparable yields (*ortho* and *para* substituents to COOH). As expected, *meta* substituted substrates delivered mixtures of regioisomers. However, if sterically more demanding substituents (*e.g.* –NMe_2_, –iPr, –*t*Bu) were used, the *para* substituted product (after decarboxylation) was formed regioselectively.

Interestingly, in comparison to Ackermann's protocol, nitro groups on the acid were not tolerated, but the unsubstituted benzoic acid gave a much higher yield (80% *vs.* 43% in Ackermann's protocol). Upon the addition of 20 mol% Cu(OAc)_2_, good regioselectivity of aryl–alkyl alkynes was achieved (placing the aryl residue on the sterically less demanding position of the olefin product).

Gooßen developed a similar protocol for methyl aryl alkynes (not shown)^157^ that was tailored towards a larger substrate scope (see Scheme 13D).^156^ Additionally, a considerably faster version under microwave irradiation was also reported. The scope for both protocols was broad and no additional additives were necessary to convert asymmetric alkynes.

Regarding the mechanism of the reported Ru catalysed alkenylation reactions, a computational study by Hong gave possible insights to the selectivity of these transformations.^135^ It was shown, that the chemoselectivity (*i.e.* consecutive annulation *versus* decarboxylation) was strongly depending on the size of the ruthenacylce prior to the decarboxylation and the polarity of the solvent used.

The alkenylation of indoles was achieved *via* Rh catalysis under relatively harsh conditions, since DCM had to be heated to 100 °C (Scheme 13E).^158^ This transformation made fluorescence-active indoles easily accessible as it gave the di-alkenylated compounds exclusively.

One example of a decarboxylative annulation was the formation of polyarenes by C–H functionalisation of diphenyl acetic acids (Scheme 13F).^159^ The transformation was catalysed by Pd(OAc)_2_ with a MPAA as ligand. First the substrate was alkenylated *via* typical carboxyl-directed C–H activation. Subsequently, the *ortho* C–H bond of the second ring was activated and an intramolecular annulation occurred. The oxidation was mediated by benzoquinone under O_2_ atmosphere. Notably, this method constructed different (hetero)anthracene derivatives in a direct way.

The previously described mono-arylation of OH protected mandelic acids (see Scheme 7J) was not only accepting aryl halides but also electron poor olefins as substrates (among them one example with phosphonates) (see Scheme 13G).^123^ More importantly, the reaction proceeded also with *N*-protected α-phenylglycine instead of mandelic acid, which enabled a fast route to unnatural amino acids. The yield of the corresponding alkenylated amino acids was generally good to excellent (62–88%).

#### 4.1.4. Alkynylation

**Scheme 14:**
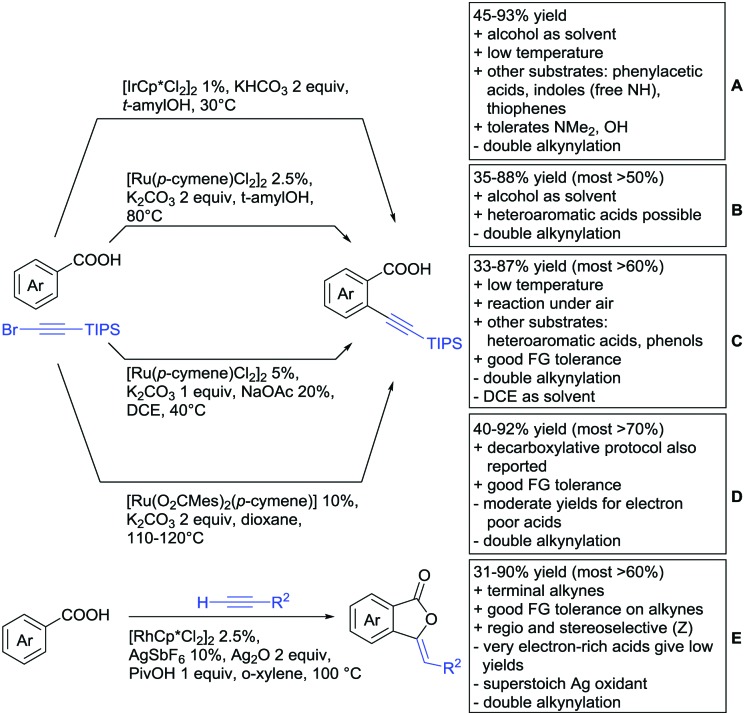
Alkynylation directed by carboxylic acids.

Alkynylation directed by carboxylates has not been extensively studied and is mostly realized with (bromoethynyl)triisopropylsilane. Zen reported two protocols (Scheme 14A and B). Protocol A involved Ir catalysis^160^ and tolerated FGs such as OH, NMe_2_ or halides. Generally, the conversion of electron-rich acids was high (76–93%). For electron-poor substrates, the yield was significantly lower (35–50%). Additionally, if both *ortho* positions to the acid were unsubsituted, di-alkynylated products were observed. The ratio of this twofold C–H functionalisation depended on the sterical demand of the remaining substituents and on the general reactivity of the acid. Notably, phenylacetic acids were also tolerated under slightly different conditions. The reaction was also performed with unprotected indolyl- and thiophenyl-3-carboxylic acids yielding 2-alkynylated products in moderate yields (49–67%).

Protocol B, the alkynylation catalysed by [RuCl_2_(*p*-cymene)]_2_ showed a similar substrate scope (see Scheme 14B).^161^ The conversion for electron-poor substrates was also lower than for more activated compounds.

A similar procedure was reported by Dorel (Scheme 14C).^162^ This time DCE instead of an alcohol was used as solvent, a disadvantage regarding green chemistry efforts. As the protocol was also applied in a phenol-directed *peri* alkynylation, free phenols were also accepted as substrates for this protocol. Additionally, also nitro groups and unprotected aldehydes were tolerated FGs for this transformation.

Ackermann reported a transformation with a Ru carboxylate as the catalytic species (Scheme 14D).^163^ In comparison to the transformations above, this protocol showed slightly better yields for electron-poor substrates. However, the reaction conditions were not as mild and di-alkynylation still remained a problem. A decarboxylative protocol was also reported. Under elevated temperatures and addition of acetic acid after the product formation, the carboxyl group was removed without the typically employed Ag or Cu salts.

One example of alkynylation/lactonisation was reported making use of terminal alkynes and Ag_2_O as oxidant (Scheme 14E).^164^ This very fast reaction gave moderate to good yields with a relatively broad substrate scope. Notably, only stoichiometric amounts of alkyne were used and the reaction gave the *Z* isomer exclusively. However, both positions *ortho* to the acid were alkenylated and the alkylated lactone was formed as a side product.

#### 4.1.5. Aminocarbonylation

**Scheme 15:**
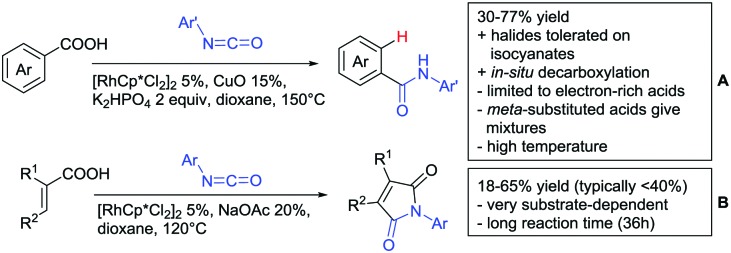
Aminocarbonylation directed by carboxylic acids.

The synthesis of *meta*-substituted aromatic amides can be achieved by an *ortho* amidation and subsequent decarboxylation of benzoic acid derivatives (Scheme 15A).^165^ This Rh catalysed transformation used isocyanates to install the amide group and the carboxylate was subsequently removed using CuO. The methodology was applicable to *ortho* and *para* substituted acids, which gave the same *meta* amidated product.

Arylisocyanates could also be applied in a synthesis of cyclic *N*-aryl imides (Scheme 15B).^166^ In this case, α substituted acrylic acids reacted with the isocyanates under Rh catalysis *via* a β vinyl C–H activation.

#### 4.1.6. C–Heteroatom bond formation

**Scheme 16:**
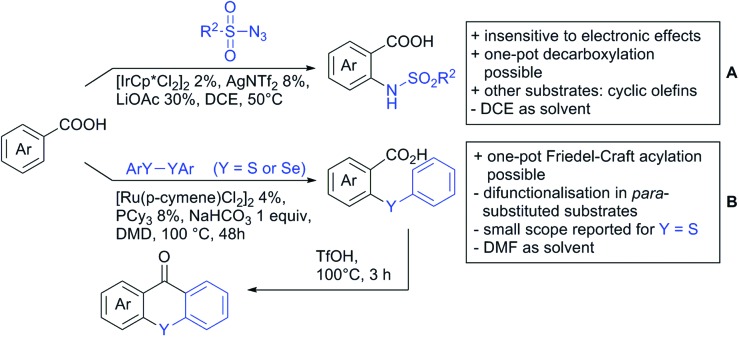
C–Heteroatom bond formation directed by carboxylic acids.

*ortho*-Directed C–H functionalisation of benzoic acid derivatives with sulfonyl azides under Ir catalysis yielded *N*-sulfonyl substituted anthranilic acids (see Scheme 16A).^167^ A one-pot procedure for protodecarboxylation (either with Pd or Cu), giving access to *N*-sulfonyl substituted anilines was also disclosed. Furthermore, also cyclic olefins were transformed under these reaction conditions.

Selenation and thiolation could be achieved by reaction with diselenides or disulfides under Ru catalysis (Scheme 16B).^168^ The process proceeded generally to mono-chalcogenation for *meta* substituted arenes. However, apart from MOM-protected 4-hydroxybenzoic acid, *para* substituted acids were functionalised in both *ortho* positions to the acid. Different substituted diselenides could be transformed with good yields and the protocol also tolerated diphenyl disulfides. More importantly, this method provides the possibility to synthesise chalcogenxanthones *via* an intramolecular Friedel–Craft acylation by addition of triflic acid to the crude mixture after the reaction. For this annulation benzoic acids carrying halides, ethers, alkyl, and aryl groups were tolerated and also thiophene carboxylic acid was used as substrate giving good yields (58–86%) over two steps.

### 4.2. C(sp^3^)–H activation

**Scheme 17:**
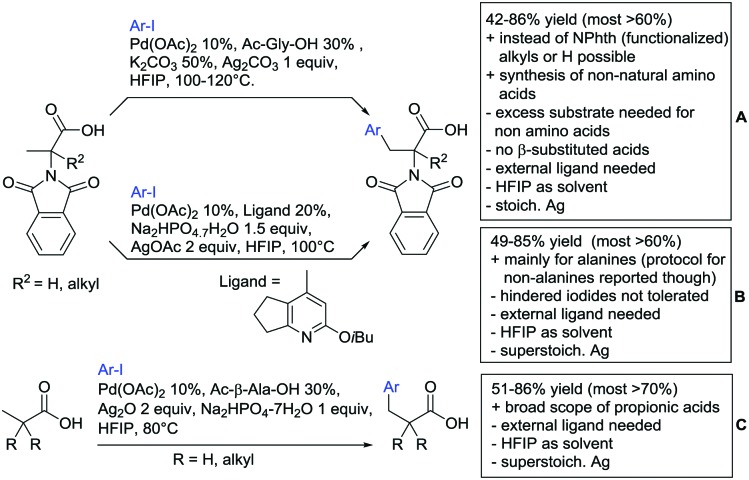
Functionalisation of C(sp^3^)–H bonds directed by carboxylic acids.

Advances have been made in the β-C(sp^3^)–H activation and arylation of propionic (also α-amino) acids. Zhao,^169^ Yu^170^ and van Gemmeren^171^ all reported Pd catalysed protocols for this transformation (Scheme 17A–C).

Zhao (Scheme 17A) focused on the arylation of *N*-phtaloyl alanines to synthesise non natural amino acids in a one-step procedure.^169^ Generally, the scope of the transformation regarding aryl iodides was good, and the yields reported are in the 62–90% range. The reactions covered a wide range of *para*- and *meta*-substituted aryl iodides, one example of *ortho* substitution was also reported (1-fluoro-2-iodobenzene). Notably, different substituents in the α position of the acids were also accepted, and gave good yields if the aryl iodide was the limiting substrate (45–75%). Interestingly, it was even possible to arylate substrates with a quaternary α carbon. Limitations were shown with acids substituted in β position as they gave only traces of the product.^169^

The protocol developed by Yu (Scheme 17B) focused again on *N*-phtaloyl alanine as substrate.^170^ Here instead of a MPAA a fused pyridine ligand was used. This protocol also accepted acids with a quaternary carbon adjacent to the site of activation.

An improvement regarding the generality of the method of Zhoa has been achieved with the use of another MPAA as ligand and by using a different base and Ag salt (Scheme 17C).^171^ This protocol showed a broad substrate scope regarding the propionic acid derivatives and also tolerated a quaternary centre at the α position to the acid. In contrast to the above method, the acid derivatives were used as limiting substrate here.

## 5. Esters as DGs

The ester group is omnipresent in natural as well as synthetic organic molecules. Furthermore, esters can be introduced or interconverted by a variety of methods and the need for the development of reliable ester-directed C–H functionalisation procedures is therefore obvious. Pioneering work was performed in the 90s by Trost^172^ and Murai.^173^ However, esters coordinate weakly to metal catalysts, which make the development of successful protocols difficult. Therefore, not many contributions have yet been reported in the field. Compared to the free acids much fewer examples have been disclosed in the last three years, which will be discussed in the following section.

### 5.1. C(sp^2^)–H activation

#### 5.1.1. Alkylation

**Scheme 18:**
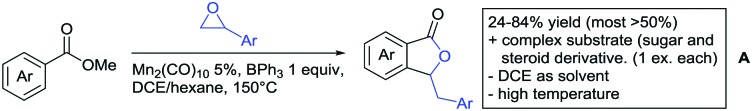
Mn-Catalysed lactonization directed by esters.

Mn could be successfully applied as catalyst for an alkylation reaction (Scheme 18A).^174^ This borane promoted transformation used epoxides and methyl benzoates to furnish five-membered lactones. Different aromatic esters could be converted. Notably, the authors also showed that the protocol was applicable on more complex molecules. One example of a sugar and steroid derivative were provided. With regard to the reagent, the oxiran and arylated derivatives were also reported as effective coupling partners.

Also 2,2-diphenyloxiran was converted, albeit with low yield (23%). If the aryl residue was replaced with a *t*-butyl group the unsubstituted furanone was formed. Presumably a Meinwald rearrangement leads to the formation of formaldehyde that is incorporated into the five membered ring.

#### 5.1.2. Alkenylation

**Scheme 19:**
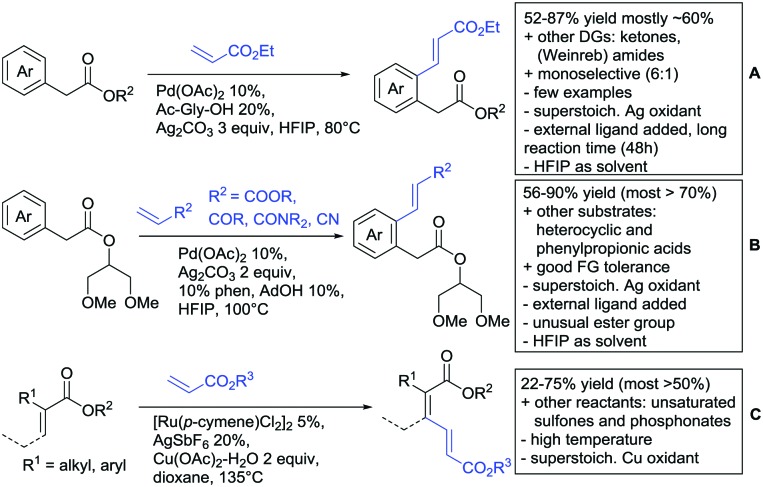
Ester-directed oxidative alkenylation reactions.

Oxidative alkenylations with α,β-unsaturated olefins to alkyl phenylacetates could be achieved under Pd catalysis with MPAAs as ligands (Scheme 19A).^175^ The protocol could also be applied to amides, Weinreb amides and ketones as DGs (see Sections 2, 6 and 7). If electron rich esters were used, the reported yields were good (52–87%). Compared to Weinreb amides, the reaction proceeded with better mono-selectivity (6 : 1 for ester DG in comparison to 1 : 2 for Weinreb amide DG on phenylacetate). The authors argued that the products were easier released by the metal species and hence di-alkenylation did not preferentially occur.

Also under Pd catalysis, Zhao and co-workers reported a similar transformation, with quite an broad substrate scope (Scheme 19B).^176^ Ac-Gly-OH was replaced with 1,10-phenantroline as bidentate ligand, and instead of alkyl esters, they employed a 2-(1,3-dimethoxy)propyloxy handle. The authors speculated that this residue enhanced the coordination ability of the substrate (compared to methyl, ethyl or 2-methoxyethyl residues) to prevent an unselective Fujiwara–Moritani reaction and mono selectivity of the reaction (33 : 1) was achieved.

Notably, with this method also synthetically useful yields were reported for starting materials carrying electron withdrawing groups (60–82%). Heterocyclic esters also proved to be good substrates. Additionally different esters were converted with good yields: instead of phenyl acetates, also phenylpropionic acid esters and α substituted phenylacetates were possible substrates. Additionally, the method was not limited to acrylates but also α,β unsaturated nitriles, amides, and ketones were applicable as reaction partners.

Loh reported alkenylations of α-substituted acrylates with olefins (mostly acrylates) (Scheme 19C).^177^ The vinylic C–H activation was catalysed by Ru and used Cu(OAc)_2_ as oxidant. Different alkyl and aryl substituents were reported as possible substrates for the transformation. To achieve full conversion of the α-substituted acrylate, 2 equiv. of the coupling partner were necessary, as homodimerization of the unsubstituted acrylate was also observed. The stereoselectivity (*Z*/*E*) was usually about 9 : 1 and increased if a bulkier α substituent (R^1^) was used as coupling partner. Regarding olefin coupling partners, α,β-unsaturated sulfones (56%) and phosphonates (22%) could be also converted.

**Scheme 20:**
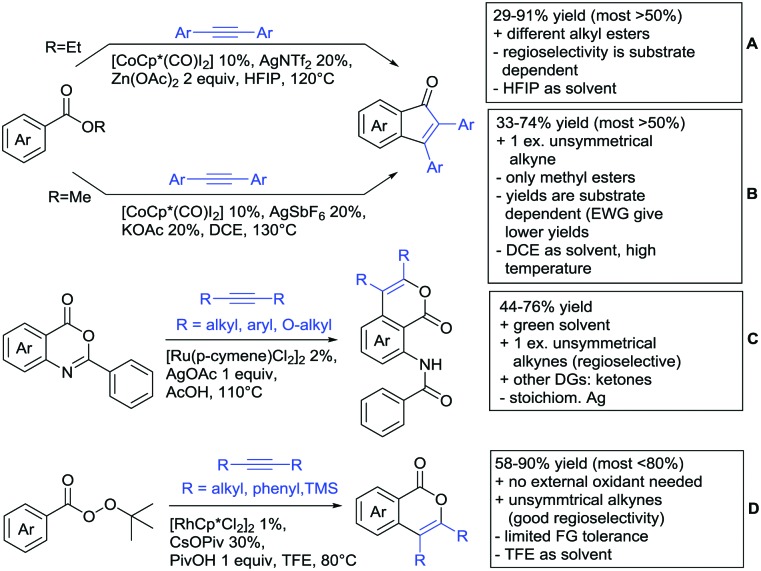
Ester-directed alkenylation and consecutive annulation.

Alkenylation reactions with consecutive annulation were also reported with ester DGs as depicted in Scheme 20.

The synthesis of indenones under Co catalysis was reported by Li (Scheme 20A)^178^ and Zhang (Scheme 20B).^179^ Both protocols employ [CoCp*(CO)I_2_] as precatalyst and a Ag salt as additive. The method developed by Li *et al.* used additionally Zn(OAc)_2_ to enhance reactivity by coordination of the metal to the ester. If the substrate carried a *meta* substituent, the regioselectivity depended on the size and ligating ability of the residue. A *meta*-methoxy ester gave a regioisomeric mixture of 1.2/1, whereas the smaller fluorine atom also coordinated to the catalyst, directing the alkyne into the more hindered position (15/1 regioisomeric ratio).^179^

The second Co catalysed annulation (Scheme 20B) seemed to be more sensitive to the electronic effects of the aryl substituents. Activating (OMe, Me) residues led to good yields, whereas low yields were observed if esters, –CF_3_ or other electron-withdrawing substituents were present in the substrate. Different symmetrical aryl alkynes were converted in moderate to good yields (44–72%). Additionally, the authors provided one example of an unsymmetrical alkyne (methyl, phenyl). In this case the regioisomer with the phenyl group α to the carbonyl was preferred (2 : 1).

Lactones can also be used as DGs. Patel *et al.* reported on an oxidative annulation of alkynes to 2-arylbenzoxazinones (Scheme 20C).^180^ The authors suggest the opening of the oxazinone to *N*-benzoylanthranilic acid under AgOAc/AcOH mediation. The free acid is then coordinating to the Rh catalyst to give a five membered ruthenacycle, which directs the alkyne to be incorporated into the desired position. The coordinating ability of the nitrogen in the substrates did not hinder the transformation in any way and the use of different internal alkynes (alkyl, aryl and alkoxy substituted) were demonstrated. Furthermore, it was also shown in one example that an unsymmetrical alkyne gives only one regioisomer. In this case the smaller residue was situated *para* to the carbonyl group. Different FGs on the lactone were tolerated such as halides and SMe. Annulation with 2-phenylquinolin-4(1*H*)-one as substrate was also demonstrated.

Cui reported a Rh catalysed alkenylation/cyclisation directed by peroxyesters, serving as internal oxidant (Scheme 20D).^181^ Several different peroxyesters were used in the reaction. However, only limited FG tolerance was observed. Different internal alkynes (alkyl and aryl) were also used as substrates. Additionally, also unsymmetrical alkynes were converted with very good regioselectivity (again the smaller residue is situated adjacent to the aromatic ring).

#### 5.1.3. Borylation

The C–B bond is of great importance in organic chemistry, mainly due to cross-coupling chemistry. However, methods for DG assisted C–H borylations are relatively rare.^31^

**Scheme 21:**
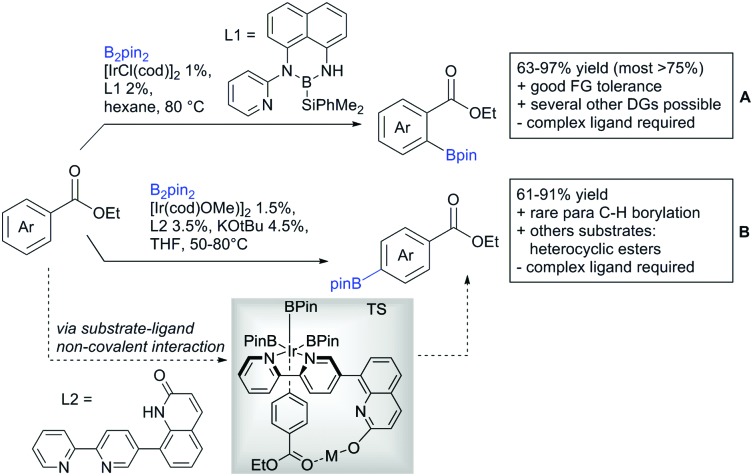
Ligand-assisted *ortho* and *para* borylation.

Li reported on a N,B ligand that enables *ortho*-directed C–H borylations with esters as DGs (Scheme 21A).^182^ In combination with [IrCl(cod)]_2_ as precatalyst, the authors reported high yields for the C–H borylation with B_2_pin_2_. Di-borylation could be excluded if large alkoxy groups (*t*Bu) were used at the ester.

Although not strictly a group-directed reaction (the ester moiety does not coordinate to the catalyst itself but to its ligand), *para* selective borylation was achieved by the group of Chattopadhyay (Scheme 21B).^183^ This reaction was also catalysed by Ir. The excellent *para* selectivity was achieved by non-covalent interaction between the ester moiety of the substrate and the ligand (see TS in Scheme 29B). Different aryl esters were borylated with good to excellent yields. The transformation was also applicable to electron poor and rich heteroaryls. The authors showed, that esters were necessary to direct the C–H activation, since ketones, aldehydes, and carboxylic acids were all reduced under the reaction conditions, and did not yield the desired product.

## 6. Monodentate amides as DGs in C–H functionalisation reactions

Substituted as well as unsubstituted amides proved to be extremely valuable as DGs in C–H activation. The first example utilizing a primary amide (CONH_2_) in C–H activation was presented by Li *et al.* in 2012.^184^ By now, amides are amongst the most frequently applied DGs in C–H functionalisation chemistry. The largest part of these contributions takes advantage of bidentate DGs including an amide moiety. However, these groups are treated in a separate chapter (see Section 14). Within this chapter, solely amide DGs are treated which use a mono-dentate binding mode.

Due to the plethora of examples reported since 2015 contributions with great variations regarding the introduced residue have been disclosed, and also the substrate variety is significant including the activation of C(sp^2^)–H and C(sp^3^)–H bonds alike. Most contributions still take advantage of precious metals, but it can be seen that base metal catalysis is moving forward and becomes more and more important.

### 6.1. C(sp^2^)–H functionalisation

#### 6.1.1. Arylation

**Scheme 22:**
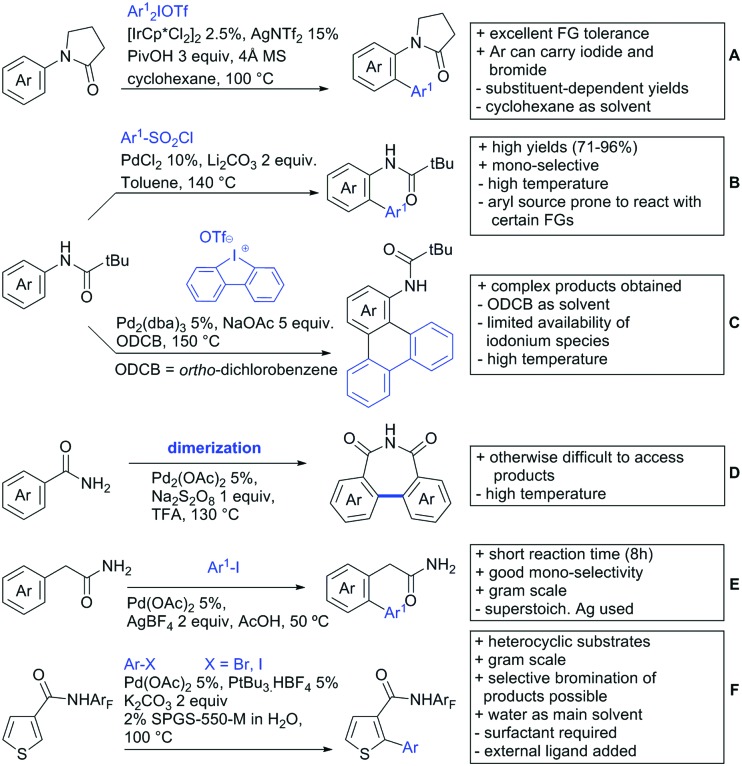
Amide-directed arylation reactions.

Arylation reactions are the most frequently reported transformation in C–H activation chemistry. A variety of different aryl sources can be used as coupling partner, which is also reflected in the examples for amide-directed arylation reactions displayed in Scheme 22. In contrast to alkylation reactions, the reports on arylation reactions mainly relied on a Pd species as catalyst. One exception was the Ir catalysed arylation with diaryliodonium salts published by Shi and Yuan (Scheme 22A).^185^ In this contribution, they applied a protocol previously reported for *ortho* arylation of *O*-methyl ketoximes^186^ to *N*-aryl-2-pyrrolidinone as substrate. FG tolerance was excellent, but electron withdrawing residues reduced the yields to the 50% range, whereas electron donating ones gave yields also >90%. On the Mes-I-Ar^+^OTf^–^ species, which was used as coupling partner, also bromo and iodo were tolerated and Ar could also be thiophene or naphthalene.

The group of Kianmehr introduced arylsulfonyl chlorides as aryl source in amide-directed *ortho* arylation (Scheme 22B).^187^ The reaction system was rather simple comprising of PdCl_2_ and Li_2_CO_3_ but required 10 mol% catalyst loading. Still, yields were high (71–96%) and the reaction gave only mono-arylated products. In fact, the presence of an *ortho* methyl group shut down the reaction completely.

A quite peculiar transformation was reported by Park and Hong. They developed an efficient strategy to build up triphenylene frameworks by using a cyclic diaryliodonium salt as aryl source, which basically resulted in *ortho* and *meta* arylation of mainly *N*-phenyl pivalamide substrates (Scheme 22C).^188^ The reaction required the presence of a substituent in one of the *ortho* position for obtaining high yields (71–95%), since otherwise only a maximum of 30% was obtained. Switching to *N*-aryl-2-pyrrolidinone as substrate allowed a two-fold reaction with the iodonium salt species leading to heptacyclic products in moderate yields.

Dimerisation of benzamides towards 5*H*-dibenzo[*c*,*e*]azepine-5,7(6*H*)-diones was reported by the group of Bao (Scheme 22D).^189^ Since the reaction required a relatively strong oxidant to proceed, the authors favoured a Pd(ii)/Pd(iv) catalytic cycle, however, they did not carry out mechanistic investigations. Regarding the benzamide substrate, *meta* and *para* substitutents were tolerated (Me, OMe, *t*Bu, F, Br, Cl) but *ortho* substitution shut down the reaction.

Kumar and co-workers used primary phenylacetamides as substrates, which suggested that in this case not a 5-membered, but a 6-membered palladacycle was involved in the catalytic cycle.^190^ In this case, aryl iodides were the coupling partners, which allowed the presence of bromide in the substrate (Scheme 22E). Otherwise, mainly “inert” FGs were used such as Me, OMe, Cl, F and NO_2_. Noteworthy was the good selectivity for mono-arylation and a gram scale experiment which showed the potential for scale up.

Tan and co-workers focused on the arylation of 3-carboxamido thiophene and aryl bromides (Scheme 22F).^191^ The amide nitrogen contains a pentafluorphenyl. Arylation took initially part in position two and in some cases 2,4-diarylated compounds were observed (maximum 15%). Also heterocyclic bromides could be used as coupling partner and it has to be mentioned that 2 equiv. of halide were applied in order to get good yields (51–87%, 15 examples). Besides the thiophene substrate, in one case also a 3-furancarboxamide was arylated using phenyl iodide (59% mono, 17% diarylation). Additionally, gram scale experiments were carried out as well and starting from 2-aryl-3-thiophencarboxamide, bromination conditions for selectively brominating either in position 4 or 5 were disclosed.

**Scheme 23:**
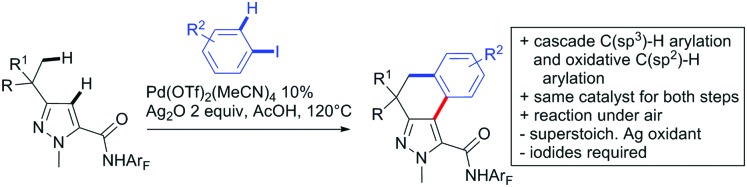
Tandem amide-directed arylation-oxidative coupling.

A cascade of C(sp^3^)–H and C(sp^2^)–H activation has been reported by Stamos and Yu (Scheme 23 see also Section 15.5).^192^ According to the proposed catalytic cycle, the first step was a pyrazole-directed arylation of a *t*-butyl group (Scheme 23, blue bond formed), whereas the second step was an oxidative coupling between position 4 of that same pyrazole and the introduced aryl ring (Scheme 23, red bond formed). This C–H activation step of the pyrazole was indeed amide-directed. Both steps were Pd catalysed and could be run under air, not uncommon for an oxidative coupling step. The range of FGs on the aryl iodide was remarkable and yields between 45–78% were obtained. The *t*-butyl group could be substituted for other quaternary centres, whereas the arylation always took place at a methyl group.

#### 6.1.2. Alkylation and allylation

Alkylation reactions are generally less frequently reported as arylation reactions. Still, a series of interesting alkylation reactions has been reported using quite a variety of alkyl sources.

**Scheme 24:**
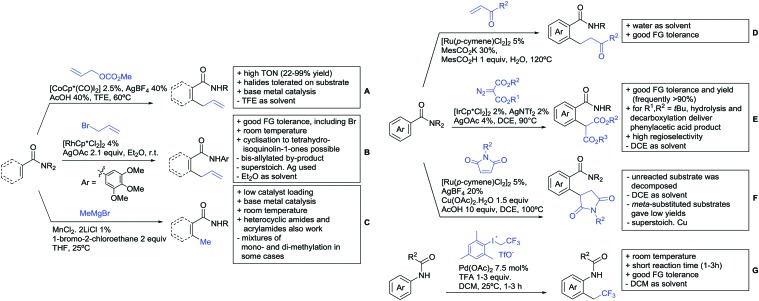
Amide-directed alkylation and allylation reactions.

The group of Glorius reported Co catalysed allylation using methyl allyl carbonate as the allyl source (Scheme 24A).^193^ Besides using pyrimidine as DG, *N*-alkylated amides were applied as well and it was shown that mono- and di-alkylated amides worked as well and that steric bulk on the amide was well tolerated, including a *t*-butyl group. Besides substituted benzamides, also acrylamides and 3-furan- and 3-thiophencarboxamides could be allylated as well. In most cases yields were in the 60–80% range.

Another allylation procedure was reported by Yan, this time using allyl bromides and a Rh catalyst (Scheme 24B), which made the procedure less attractive but the advantage was the commercial availability of the Rh catalyst, which is unfortunately not yet the case for many Co species.^194^ Additionally, the reaction was carried out at room temperature. Yields were in a preparatively useful range (34–93%) but in this case also di-allylated products were formed. Further cyclisation of the allylated products to tetrahydroisoquinolin-1-ones was reported as well.

A significant challenge are typically methylation reactions since the often applied alkyl sources, namely olefines, are not an option and alternative methyl sources need to be found. Ilies and Nakamura have published a Mn catalysed protocol using MeMgBr as alkyl source, which worked with remarkable low catalyst loading (0.01–1 mol%) (Scheme 24C).^195^ Amongst the advantages of this protocol was the simplicity of the catalyst, MnCl_2_·LiCl without any ligand, the mild conditions, and high TONs. The use of Grignard species as methyl source naturally raised the question of FG tolerance. Remarkably, even bromide was tolerated. A disadvantage was again the formation of mono- and di-methylated products in case the substrate allowed a second methylation.

The group of Ackermann reported alkylation using methyl vinyl ketone as alkyl source, introducing a butanone side chain on an aryl ring (Scheme 24D).^196^ The reaction was Ru catalysed and required MesCOOH (1 equiv.) and MesCOOK (0.3 equiv.) as additives but water could be used as solvent. Yields were preparatively useful (45–81%) and electron donating and electron withdrawing substituents on the aryl ring are tolerated as well as bromine. Further cyclisation of the initial products to 2-methylquinolines was reported as well.

An Ir-catalysed reaction of aromatic amides with diazomalonates has been reported by the group of Wang (Scheme 24E).^197^ Using dimethyl diazomalonate led to the formation of products with both ester moieties still in place. Switching to methyl *t*-butyl malonates led to methyl esters of arylacetic acids, since the *t*-butyl group was cleaved under the reaction conditions with subsequent decarboxylation. The reaction worked also on electron rich heterocycles and tolerated the presence of halides (F, Cl, Br, I) and series of other FGs without significant influence on the yields (typically >80%!).

Very recently, they expanded this method, most importantly to methylation reactions as well.^198^ In this case the alkyl source was a sulfonium salt and stoichiometric amounts of Cu(OAc)_2_ were required. Yields were mediocre in most cases, but still it was one rare example for a methylation reaction (not shown).

*N*-Substituted succinimides have been used as alkylation source in the broader sense as well. Naturally, this allowed only the introduction of a very specific group. Prabhu and co-workers showed that this transformation works under Ru catalysis and several additives (Scheme 24F).^199^ The FG tolerance was not that broadly investigated, but iodine was tolerated which was important for further elaboration of the scaffold. In competition experiments it was shown that in presence of other potential DGs (ketone, aldehyde, acid, ester) it was still the amide which exhibited the directing effect.

The group of Novak disclosed a Pd catalysed protocol for the trifluoroethylation of *N*-aryl-anilides with an iodonium salt (Scheme 24G).^200^ The introduction of fluorine moieties onto molecules is of course highly interesting in context of medicinal chemistry projects and drug development. The protocol showed high yields (typically >70% and up to 95%) and remarkable FG tolerance. Most remarkable, esters, boronic acid esters and NO_2_ were tolerated. In the presence of an acetyl group it was still the amide which directed the trifluoroethylation.

**Scheme 25:**
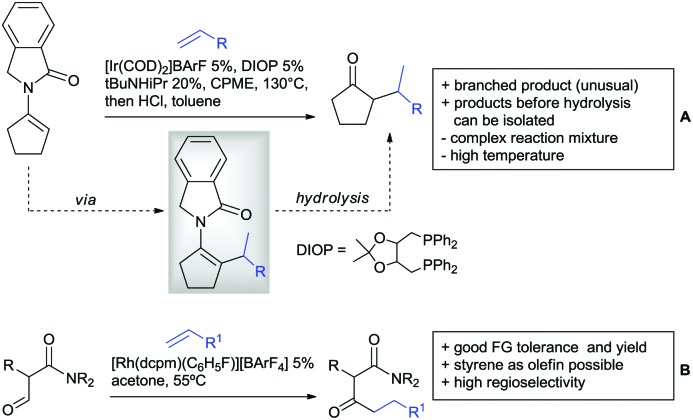
Amide-directed enamide and aldehyde alkylation.

A special case was the enamide directed α-alkylation with unactivated alkenes reported by Dong (Scheme 25A).^201^ As mentioned, the starting material was a cyclic enamide, which was first alkenylated. Adding afterwards hydrochloric acid to the reaction mixture cleaved the DG *in situ* and delivered α-alkylated ketones as products. With unactivated olefins, the new C–C bond is formed towards the C2 position of the olefin substrate, and not to C1 as it is the case with often applied acrylate species. Only few FGs were present in the starting materials and yields ranged from 20–73%.

The group of Willis reported that also a formyl C–H bond can be alkylated and alkenylated.^202^ The carbonyl oxygen of the amide was in this case involved in the formation of an intermediate rhodacycle using β-amido aldehydes as substrates (Scheme 25B). However, the amide DG can be replaced by a keto- or ester group as well. FG tolerance on the alkene was quite good (Br, OH, acetal, ester, carbamate, aryl) and frequently yields >90% were observed.

**Scheme 26:**
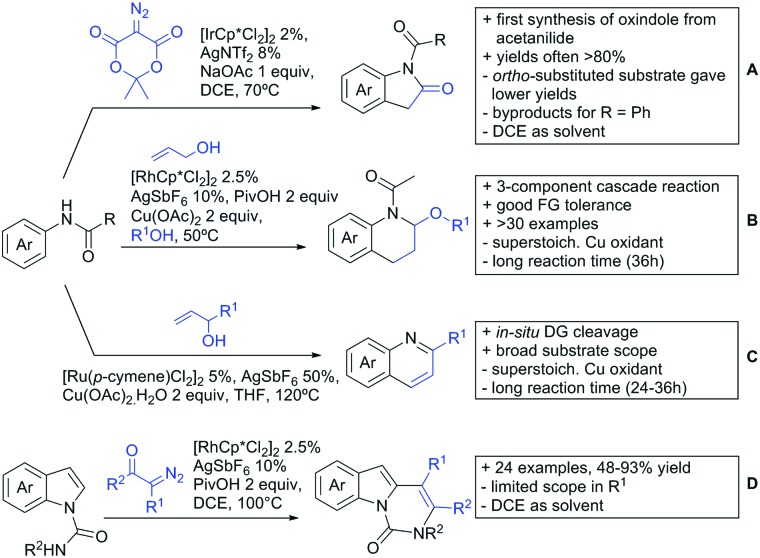
Amide-directed alkylation/allylation–cyclisation sequences.

A special case are alkylation reactions in which the alkylated product immediately cyclised under the applied reaction conditions to heterocyclic ring systems. Several such examples have been reported in the last three years. Patel and Borah reported the synthesis of oxindoles from acetanilides und Ir(iii) catalysis.^203^ The diazo derivative of Meldrum's acid was the required reaction partner (Scheme 26A). Initially, the acid was attached with its C2 carbon to the *ortho* position of the amide function. Subsequent proton induced (formed from NaOAc) intramolecular cyclisation with the amide group delivered the target compounds.

Huang and co-workers reported an allylation–cyclisation sequence to tetrahydroquinoline derivatives.^204^ Overall, they carried out a complex four component reaction using aniline derivatives, an anhydride (in most cases acetic anhydride), allylic alcohol, and 2,2,2-trifluoroethanol (Scheme 26B). The anhydride and the aniline derivative form *in situ* the corresponding amide, which directed the allylation with the allylic alcohol leading to a 3-arylpropanal derivative. Next, this intermediate cyclised to a 2-hydroxy-*N*-acetyl tetrahydroquinoline derivative and after a final etherification with trifluoroethanol (or other alcohols) the desired products were obtained. Due to this complex sequence there was a larger variation in yields (41–91%) but excellent functional group tolerance was observed.

Kapur and co-workers reported a similar sequence, but in absence of a second alcohol.^205^ Hence, the etherification did not occur but elimination of water and further aromatisation to quinoline derivatives (Scheme 26C). More than 30 examples have been reported in this contribution.

The group of Wei Zeng reported an alkylation/annulation sequence towards pyrimido[1,6-*a*]indole-1(2*H*)-ones under [Cp*RhCl_2_]_2_ catalysis with α-diazocarbonyl reagents (Scheme 26D).^206^ The method gave good access to this scaffold with the majority of examples in yields >70%. In the indole substrate Br and COOMe was tolerated amongst other less reactive FGs. The scope in R^1^ was mainly limited to carboxylic acid esters whereas R^2^ seemed to tolerate a wide range of alkyls and aryls.

#### 6.1.3. Alkenylation

Alkenylation reactions are of significant importance since the introduced double bond can be used as handle for subsequent elaboration of a scaffold. Often, the alkenylated product is not the final target, but *in situ* further transformed *via* cyclisation reactions to different heterocyclic products. Such examples will be treated in the following section. Here, only procedures will be discussed in which no further reactions took place and the alkenylated product was isolated.

**Scheme 27:**
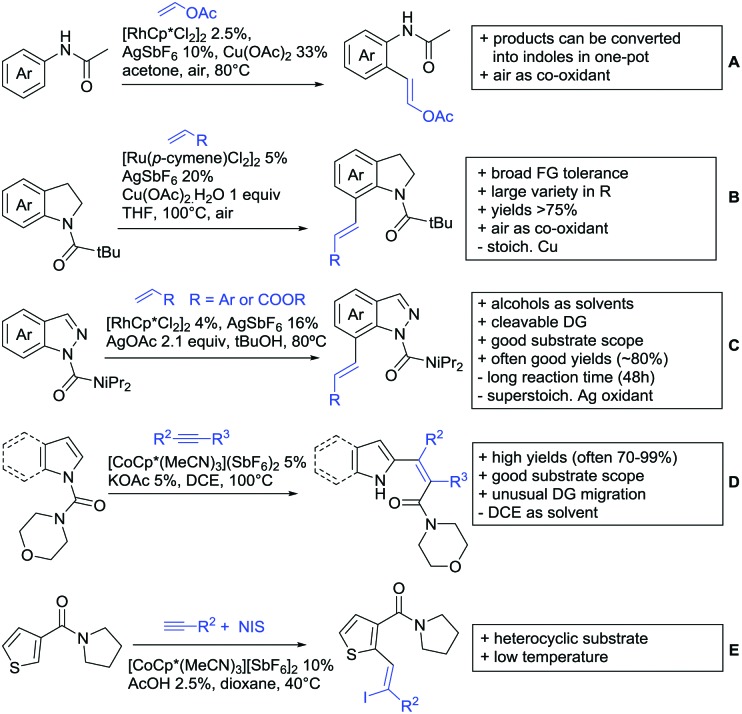
Amide-directed alkenylation reactions.

Alkenylation using vinyl acetate was reported by Zhang and Wen under Rh catalysis.^207^ This Fujiwara–Moritani reaction required only 2.5 mol% [Cp*RhCl_2_]_2_ as catalyst and was carried out under air (Scheme 27A). Hence, 0.3 equiv. of Cu(OAc)_2_ were sufficient to get to good yields. Many FGs were tolerated including bromine. *ortho* and *meta* substituted *N*-arylacetanilides gave only mono-alkenylated products, however, the unsubstituted or *para*-substituted derivatives gave mixtures of mono- and di-alkenylated products. The reaction was also carried out on a 7 mmol scale and it was shown that the products can be further cyclised to *N*-acetylindoles by adding 6 M HCl.

Huang and Zhao showed that acrylates could be used to alkenylate *N*-acylindolines and tetrahydroquinolines in positions 7 and 8, respectively, under Ru catalysis (Scheme 27B).^208^*ortho* alkenylation of *N*-arylpivalamides was also demonstrated under the same conditions. Besides acrylates, many other olefinic substrates carrying an electron withdrawing group could be used as olefin source as well. Generally, good to excellent yields (typically 70–95%) were obtained.

Shortly thereafter, Xu and Pan showed another remote alkenylation, this time of indazoles and under Rh catalysis in which they also demonstrated the cleavage of the DG (Scheme 27C).^209^

An interesting example was reported by Yoshino and Matsunaga. In this example, the DG actually migrated from the indole nitrogen to the C2 carbon of the introduced olefin group (Scheme 27D).^210^ Usually, proto demetallation would deliver the *N*-acylated 2-alkenylated indoles using alkynes as olefin source. Slowing down this proto demetallation step was key in order to allow DG migration. [Cp*Co^III^(CH_3_CN)_3_](SbF_6_)_2_ was identified as best performing catalyst and regarding the DG, morpholine amide performed significantly better as compared to other *N*-disubstituted amides. Good FG tolerance was observed and yields were frequently above 70% (up to 99%).

A second Co catalysed protocol was reported by Ellman (Scheme 27E).^211^ The first step was again an alkenylation reaction with a terminal alkyne. However, in the presence of NXS (mainly NIS), the olefine moiety was immediately halogenated. Mainly thiophene substrates were used (see Scheme 27E), but furan and pyrrole were also tested in single examples. The reaction performed at low temperature (40 °C) and the halide was incorporated on the C2 of the former alkyne with high regioselectivity.

**Scheme 28:**
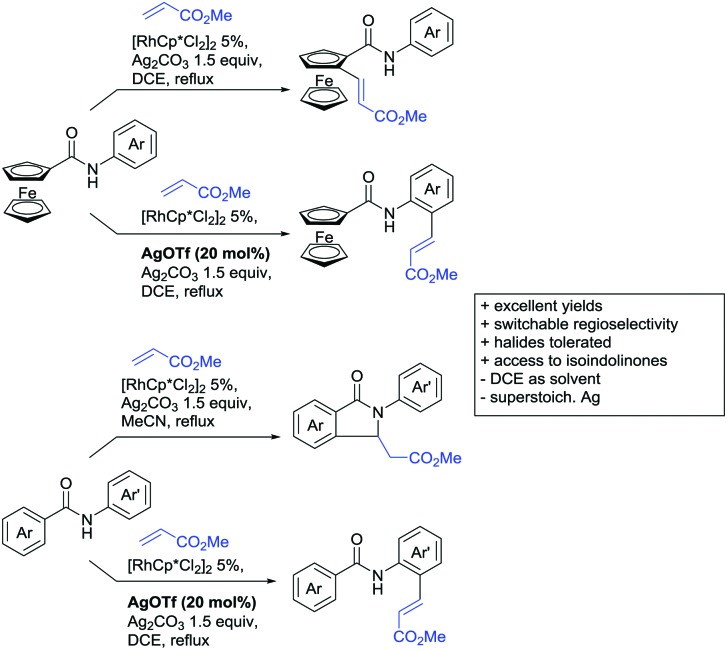
Amide-directed alkenylation with acrylates.

If *N*-arylbenzamides were the substrates, the question arises whether the *N*-aryl or the *C*-aryl ring gets functionalised. Wang *et al.* found that in a [Cp*RhCl_2_]_2_ catalysed alkenylation reaction, the key to control the alkenylation side was the presence of a non-coordinating anion, here introduced by AgOTf (Scheme 28).^212^ Initially, *N*-tolylferrocencarboxamide was used in a test reaction and in the absence of AgOTf the ferrocene moiety was alkenylated exclusively with methyl acrylate. When adding 20 mol% AgOTf the regioselectivity switched completely and only the tolyl ring was alkenylated. This arylation of the *N*-aryl ring was then further explored with *N*-aryl–*C*-ferrocenyl substrates as well as with *N*-aryl–*C*-aryl substrates. C-Ring alkenylation with methyl acrylates led to isindolinones due to intramolecular cyclisation.

**Scheme 29:**
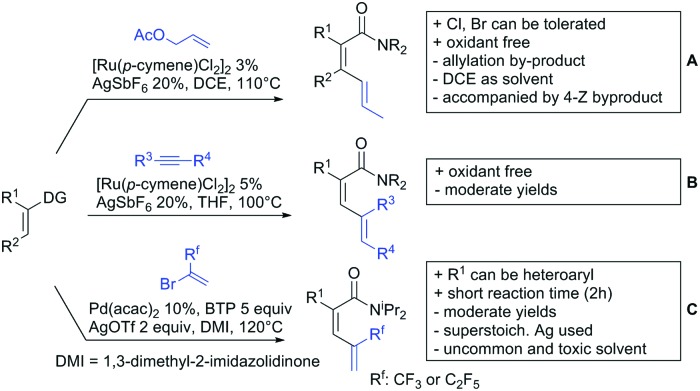
Amide-directed alkenylation of non-aromatic C(sp^2^) centres.

So far, only alkenylation of aromatic systems has been discussed. However, also olefinic systems have been alkenylated *via* amide-directed C–H activation. Zhang and Zhong reported two protocols for α,β-unsaturated amides, naturally in the β-position giving rise to products of 2*Z* configuration.^213^,^214^ In their first contribution (Scheme 29A), allyl acetate was the reaction partner, leading to (2*Z*,4*E*)-dienamide products. As could be expected, the allylated byproduct was formed as well but under the optimised conditions a selectivity of 95 : 5 or better for the desired product was reached. Also for the 2*Z*,4*E* configuration the reaction was not perfect, giving *Z*/*E* selectivities between 72 : 28 and 92 : 8 with typical yields in the 60–70% range. The second protocol used internal alkynes as alkenylation agent (Scheme 29B), which naturally eliminated the possibility for allylated byproducts giving 2*Z*,4*Z* configured products preferentially (frequently with >99 : 1 selectivity, depending on the nature of R^3^).

Also Bouillon and Poisson contributed to this field (Scheme 29C). In their case, 2-bromo-3,3,3-trifluoropropene was the coupling partner and gave the 2*Z* configuration.^215^ This is a rare case in which a bromide is the leaving group since typically oxidative couplings (*e.g.* with acrylates) are predominant. Position 2 of the substrate was mainly substituted by aryl groups and substituents on the aryl moiety had only little impact on the yield (typically ∼65%). Interestingly, when this position was not substituted, *i.e. N*,*N*-diisopropylacrylamide was used as the substrate, the configuration was *E* and not *Z*. The authors suggested that a Pd catalysed isomerisation might take place rapidly in this case.

**Scheme 30:**
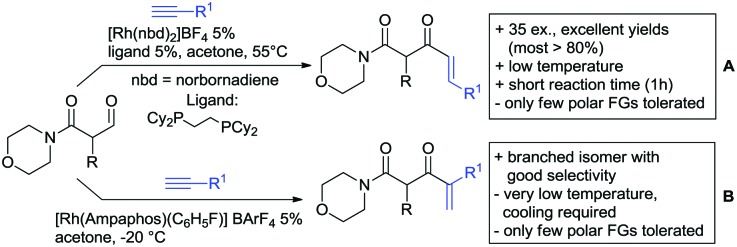
Amide-directed aldehyde olefination.

The group of Willis disclosed an impressive amount of work on the C–H activation of aldehydes, mainly directed by amides but also examples for keto- and acid ester direction were reported.^202^ They showed that switching between two Rh catalysts they could either get the linear (Scheme 30A) or the branched olefin (Scheme 30B) when terminal alkynes were the coupling partner. Linear products were obtained in excellent yields, frequently >90%. However, relatively few FGs were present. For the branched products, yields were a bit lower and the branched: linear ratio varied between 2 : 1 and 17 : 1. Using olefins as coupling partner alkylated products were obtained (not shown).

#### 6.1.4. Alkenylations with further cyclisation

This section focuses on examples where the alkenylated compound is immediately further transformed to a cyclised product. In such reactions, the amide DG is sometimes also involved in the cyclisation and one or more atoms end up in the cyclised products. The most common source for the olefin moiety in such alkenylation reactions are alkynes or acrylates, respectively derivatives thereof.

**Scheme 31:**
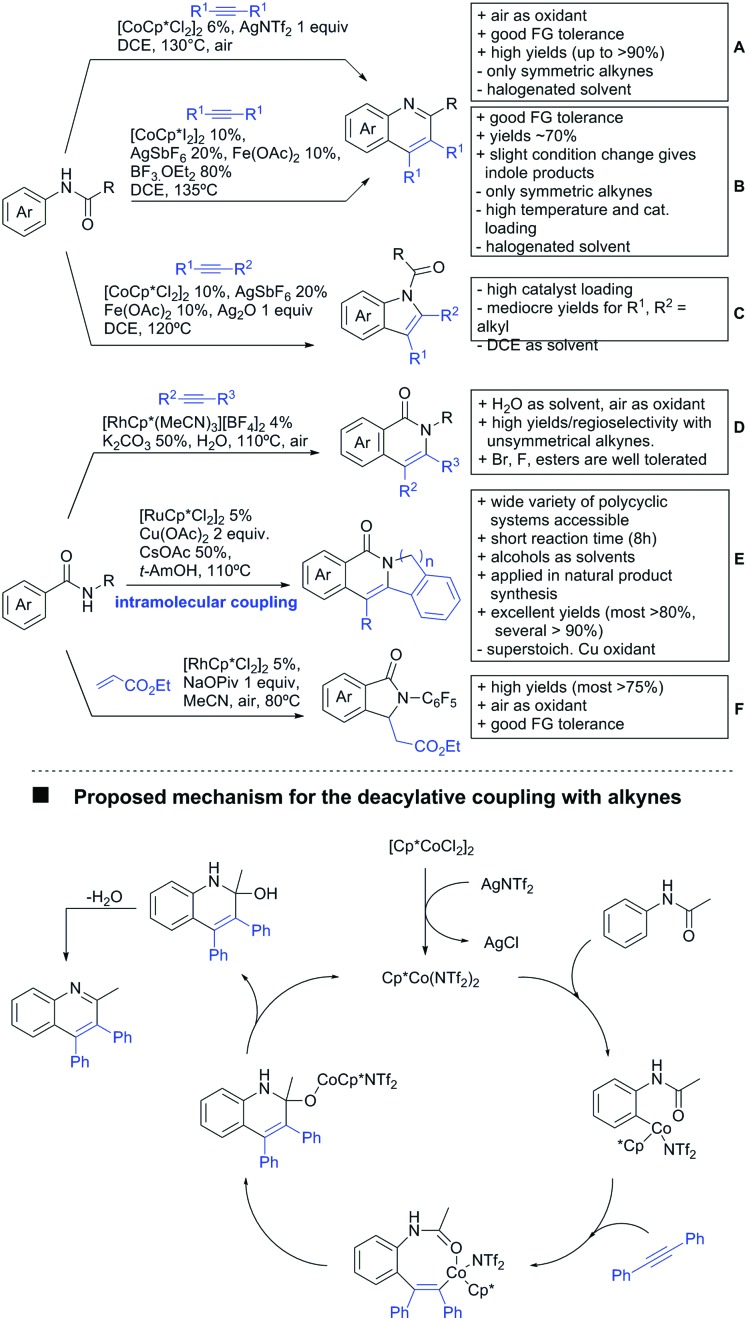
Amide-directed alkenylation–cyclisation sequences.

Several protocols have been reported in which the final product is a quinoline. In all these cases, the amide was attached *via* its nitrogen to the aryl moiety and the amide nitrogen ends up as the heterocyclic nitrogen. Within a few weeks, the groups of Li (Scheme 31A)^216^ and Glorius (Scheme 31B)^217^ reported Co catalysed protocols for this type of quinoline synthesis. The conditions are quite similar, Li used [Cp*CoCl_2_]_2_ as catalyst whereas Glorius used [Cp*CoI_2_]_2_ both in DCE as solvent and a Ag salt as additive. Li's protocol required 1 equiv. AgNTf_2_ whereas Glorius used 20 mol% AgSbF_6_ combined with 10 mol% Fe(OAc)_2_ at slightly higher catalyst loadings. The yields and the substrate scope in both protocols were comparable as both reported that halides (amongst other FGs) were well tolerated. Typically, symmetrically substituted alkynes were used as coupling partners and in the few cases where unsymmetrically substituted alkynes were used, mixtures of regioisomers were obtained. In most examples acetamide was the DG, but also examples with other alkyls were reported to work, unless steric bulk was too large. In two examples, Glorius showed that addition of 1 equiv. Ag_2_O gave *N*-acylated indole products (Scheme 31C) but better yields for this product class were obtained with urea derived DGs (see Section 9).

If the amide DG was attached to the aryl system the other way round, *i.e. via* the carbonyl carbon, reactions with alkynes led upon cyclisation to isoquinolones (or a related system, depending on the specific substrate). Chuang and Chen reported such a protocol under quiet environmentally benign conditions, since the reaction could be carried out in water under air with a Rh catalyst (Scheme 31D).^218^ Again, symmetric aryl–aryl alkynes were the coupling partners in most cases but in three examples aryl–alkyl alkynes were used. The regioselectivity was actually quite good (87 : 13 up to 98 : 2) and the aryl ring always ended up in position 3 in the main product. One exception was the reaction of *N*-methyl-4-methoxybenzamide and *N*,*N*-dimethyl-3-phenyl-prop-2-yn-1-amine, where the regiochemistry was reversed and the aryl moiety was exclusively in position 4. A related transformation on thiophenecarboxamide substrates was reported by Miura and coworkers (not shown).^219^

Similar, but Ru catalysed cyclisations have been disclosed by the groups of Swamy^220^ and Urriolabeitia (not shown).^221^ The former one used chromene-3-carboxamides as substrates, the latter one various heterocyclic amides. Hence, in both cases a pyridinone ring was annulated to another heterocyclic ring, giving access to interesting scaffolds.

Tian and Van der Eycken published an intramolecular variant of this type of transformation (Scheme 31E).^222^ This allowed them the synthesis of polycyclic systems which occurred in natural products as well. In their paper, they applied this method for building up a variety of polycycles in good to excellent yields, and also demonstrated the synthesis of two natural products, rosettacin and oxypalmatine.

When using acrylates instead of alkynes as the olefination reagent, isoindolin-1-ones are the resulting products as demonstrated by Lu and Yu (Scheme 31F).^223^ The olefinated product could be isolated only in a few cases (*e.g.* with *N*-C_6_F_5_-2-furancarboxamide as substrate or when styrenes were used as olefinic coupling partner instead of acrylates) and mainly spontaneous cyclisation to the isoindolin-1-ones *via* base mediated 1,4-conjugate addition was observed. Typical yields were in the range of 80–93%.

#### 6.1.5. Miscellaneous C–C bond forming reactions

In this section, all C–C bond forming reactions which do not fit in any previous section are summarized.

**Scheme 32:**
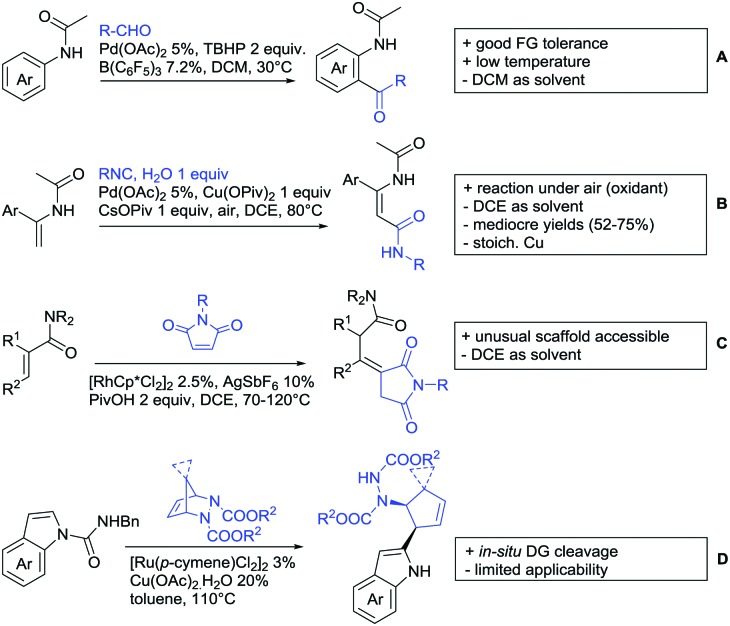
Miscellaneous amide-directed reactions.

*ortho* acylation of *N*-aryl acetamides with aldehydes was reported by Novak and co-workers (Scheme 32A).^224^ They found that the presence of a catalytic amount of B(C_6_F_5_)_3_ had a beneficial effect on catalysis and a series of aryl–aryl ketones were synthesized. Using aliphatic aldehydes, the yields were generally lower but overall there was a high variance of yields. Alternatively to aldehydes, α-oxocarboxylic acids can be used as acyl source in a decarboxylative reaction. Again Pd(OAc)_2_ is used as catalyst in presence of an oxidant, in this case K_2_S_2_O_8_ (not shown).^225^

Carboxamidation of enamides by isocyanides was reported by Liang and Luo (Scheme 32B).^226^ So the amide-directed the introduction of another amide group at a terminal double bond. Fair to good yields were obtained and also good FG tolerance in this Pd(OAc)_2_ catalysed protocol was observed.

The protocol reported by In Su Kim is according to the proposed mechanism a C–H alkylation in the first step.^227^ However, this product was not isolated but a double bond migration occurred and hence this example was displayed in this special section (Scheme 32C). Acrylamides were the substrates and maleimides the coupling partners, which led to quite unusual but interesting product structures. With the optimized protocol ([Cp*RhCl_2_]_2_, AgSbF_6_, PivOH) almost two dozen examples were reported in low to excellent yield range. The high yields (also >90%) were obtained with acrylamide derived substrates but other types of substrates performed significantly worse.

Finally, a peculiar cyclopentenylation reaction has been reported by Radhakrishnan and co-workers (Scheme 32D).^228^ The reaction required a specific type of bicyclic diaza olefin and hence its utility was limited to very specific examples.

#### 6.1.6. Halogenation

So far only C–C bond forming reactions were presented and these are also the majority of contributions. Starting from this section, also other transformations are discussed.

**Scheme 33:**
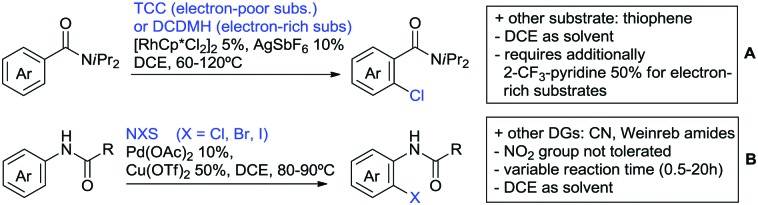
Amide-directed halogenation reactions.

Halogenation reactions *via* directed C–H-activation are of particular interest since they can be used to override the directing effects of substituents present in the substrate. Mück-Lichtenfeld and Glorius developed an *ortho* chlorination protocol of benzamides using a Rh catalyst (Scheme 33A).^229^ Interestingly, often applied NCS did not work but trichloroisocyanuric acid (TCC) or 1,3-dichloro-5,5-dimethylhydantoin (DCDMH) in combination with 2-trifluoromethylpyridine as activator. The most interesting examples were of course in which the chlorination was directed at the *meta* position compared to a usually *ortho*/*para* DG (and of course *ortho* to the carboxamide group) such as methoxy or methyl.

Kapur and Das showed that under Pd catalysis, Weinreb amides and *N*-acylanilines could direct NXS promoted halogenation in their *ortho* positions (Scheme 33B).^230^

A similar transformation but with a Pd@MOF nanocomposite catalyst was reported by Martin-Matute and co-workers. The work was mainly dedicated to pyridine-directed halogenation, but also amides worked and recyclability of the catalyst was demonstrated (not shown).^231^

#### 6.1.7. C–O bond formations

**Scheme 34:**
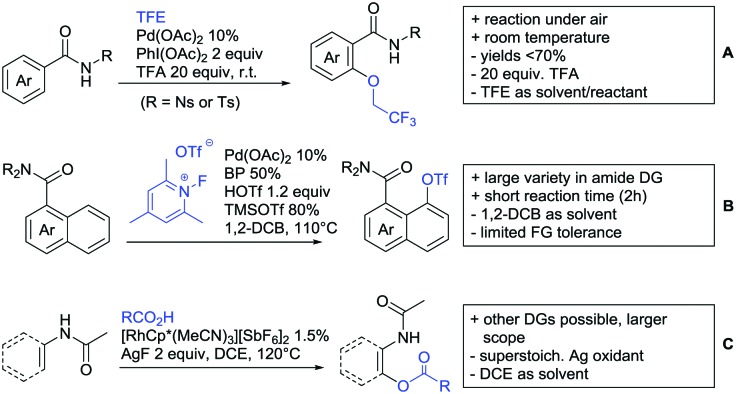
Amide-directed C–O bond forming reactions.

*N*-Sulfonylbenzamide-directed trifluoroethoxylation was reported by Ji and Li.^232^ Since the sulfur was not involved in the directing effect, this example was still treated in the amide section (Scheme 34A). Pd(OAc)_2_ was used as catalyst and PhI(OAc)_2_ as oxidant. Amongst other FGs, bromine was tolerated and typically yields in the 60% range were obtained. Application of the method in the synthesis of the drug flecainide was demonstrated as well.

C8-Triflation of naphthalenes was developed by Xiao and Fu (Scheme 34B).^233^ The optimised reaction conditions turned out to be quite complex since besides 10 mol% Pd(OAc)_2_ also 0.5 equiv. benzophenone (BP), 1.2 equiv. TfOH, 0.8 equiv. TMSOTf and 2 equiv. *N*-fluoro-1,3,5-trimethylpyridinium triflate reagent were required giving moderate to good yields.

Rh catalysed acyloxylation was reported by Xu and Li (Scheme 34C).^234^ The publication focused mainly on heterocyclic DGs but included also a table of amide-directed examples. Yields around 60% were typical.

Additionally, Chakraborti and co-workers developed a Pd catalysed hydroxylation protocol which they mainly applied to heterocyclic DGs (see Section 15.9).^235^ However, five examples of amide direction were reported but only three of them gave good yield (not shown).

#### 6.1.8. C–N bond formation

**Scheme 35:**
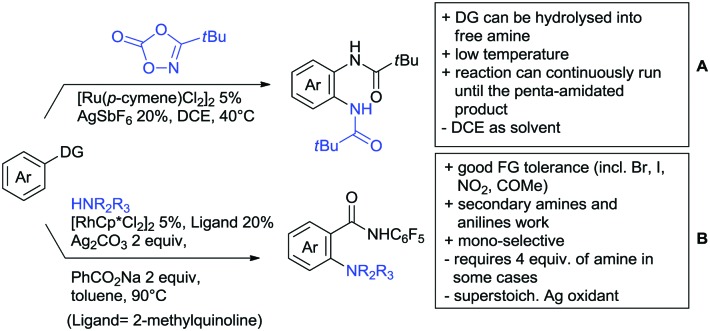
Amide-directed C–N bond forming reactions.

Sukbok Chang and co-workers reported an unusual example of amide-directed C–H activation (Scheme 35A).^236^ The group they introduced was actually again an amide, which in turn could direct another amidation and so on and so forth. Overall iterative amidation was reported with dioxazolones as amidating reagent. Starting from *N*-phenyl pivalamide, the penta-amidated product could be obtained exclusively when 6 equiv. dioxazolone were used. The hexa-amidated product was never observed, most likely due to steric reasons.

The introduction of amine groups is a particular challenge in C–H activation chemistry. One example was disclosed by Sun and Yu using *N*-C_6_F_5_-benzamides and secondary dialkylamines as coupling partners (Scheme 35B).^237^ Hence, only tertiary amines were obtained. Switching to anilines as coupling partners, secondary *N*,*N*-diarylamines were the products. Yields are generally good in this [Cp*RhCl_2_]_2_ catalysed method and FGs are tolerated to a relatively good extend.

### 6.2. C(sp^3^)–H activation

The activation of C(sp^3^)–H bonds is typically more challenging than activation of their C(sp^2^)–H counterparts. Not surprisingly, much less work has been published on this topic in recent years. In fact only three amide-directed examples (*i.e.* mono-dentate amides!) have been reported all coming from the lab of Jin-Quan Yu.

**Scheme 36:**
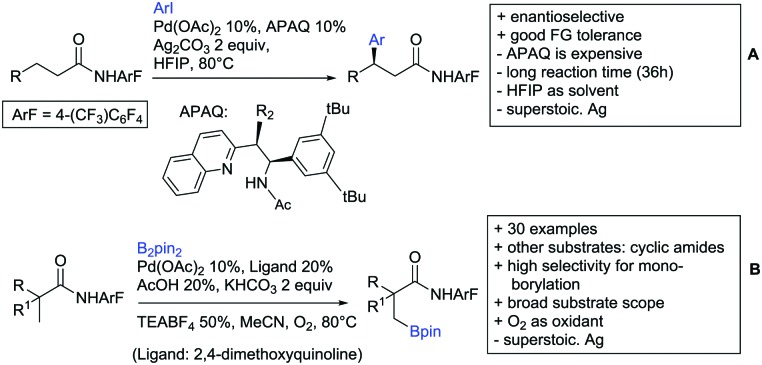
Amide-directed C(sp^3^)–H functionalisations.

The first one dealt with arylation of methylene groups in β-position to the CONH(4-CF_3_-C_6_F_4_) DG (Scheme 36A). In this case, even an enantioselective method was developed using a chiral *N*-acetyl protected 2-aminoethyl quinoline (APAQ) as ligand.^238^ They obtained e.r. of typically 95 : 5 using Pd(OAc)_2_ as catalyst and aryl iodides as aryl source. Good FG tolerance and yields between 45–89% were reported. In a second contribution enantioselective alkenylation and alkynylation reactions were also reported (not shown).^239^

The second contribution reported a Pd catalysed borylation with B_2_pin_2_ as coupling partner (Scheme 36B).^240^ Methyl or methylene groups in β-position were borylated whereas the less sterically demanding was favoured (CH_3_ over CH_2_) in cases where there was competition. Noteworthy, also saturated carbocyclic systems (C3–C7) were successfully borylated.

## 7. *N*-Alkoxy- and Weinreb amides

Besides CONH_2_, CONHR and CONR_2_ amides, also *N*-alkoxy amides *i.e.* Weinreb amides have been used as DGs, however less frequently. Not surprisingly, relatively similar catalytic systems as compared to ordinary amides are applied also in this case and also the types of reactions reported show a large overlap with the examples in Section 6.

### 7.1. Arylation

**Scheme 37:**
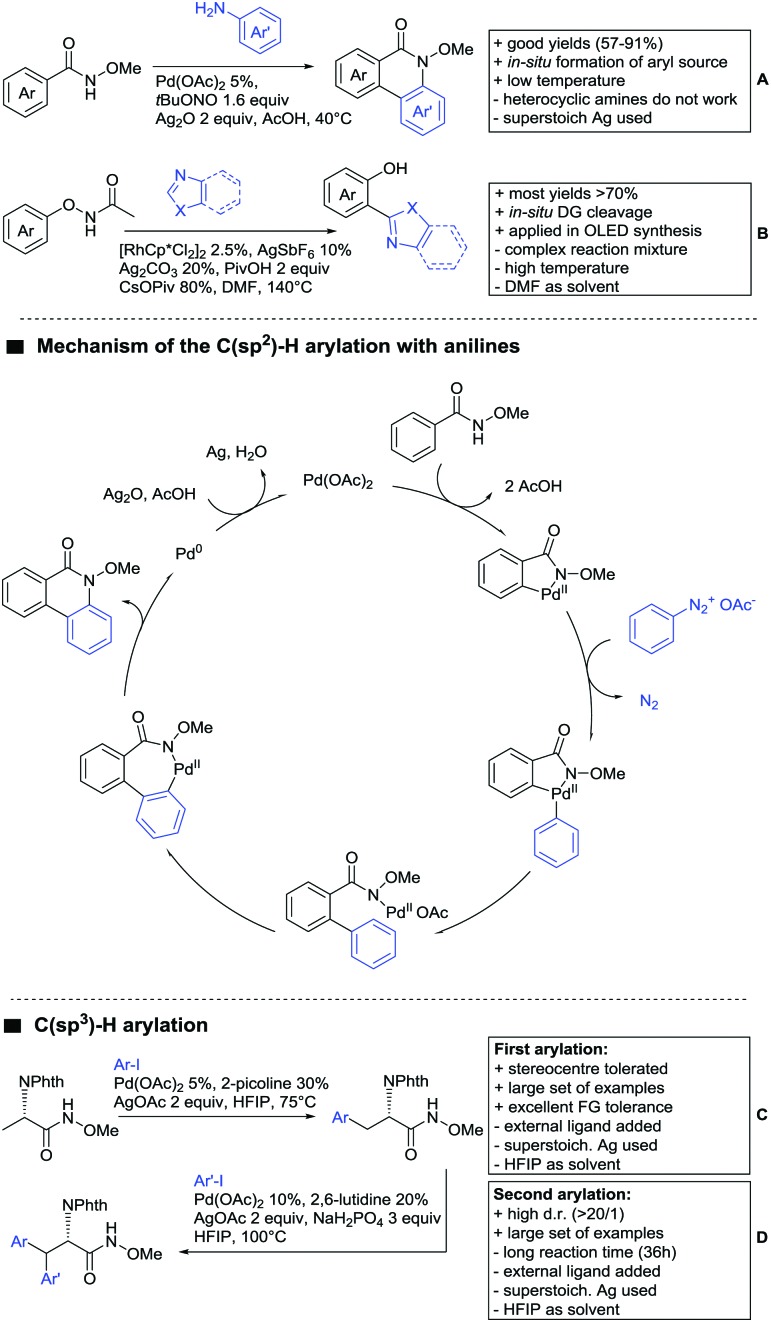
Arylation reactions directed by *N*-alkoxy and *N*-aryloxyamides.

Only two examples for arylation reactions of *N*-alkoxybenzamides have been reported in recent years. Bhanage and co-workers used anilines as aryl source, which were *in situ* converted to the corresponding diazonium salts (Scheme 37A).^241^ After the initial C–H activation and arylation, the alkoxyamide actually directs a second C–H activation on the previously introduced aryl ring and delivers phenantridinones as the final products after reductive elimination. Mild reaction conditions and good to excellent yields (57–91%) are the main advantages of this protocol. Additionally, halogens (F, Cl, Br) are tolerated on the *N*-alkoxybenzamide substrate.

Land and You reported another arylation protocol, this time of *N*-aryloxyacetamides, under Rh catalysis. In this case, oxidative coupling between the position 2 of azoles and benzoazoles was realized with typical yields >70% (Scheme 37B).^242^ Mainly oxazoles and benzoxazoles were applied but one example with benzothiazole and one with *N*-methylbenzimidazole was reported as well. Under the applied reaction conditions, the *N*-aryloxy amide was N–O cleaved and overall *ortho* arylated phenols were obtained as the products.

Jin-Quan Yu and co-workers reported a C(sp^3^)–H arylation catalysed by simple Pd(OAc)_2_ and 2-picoline as ligand, which led to mono-arylation products (Scheme 37C).^243^ These mono-arylated products could be subjected to a second arylation when a different ligand, namely 2,6-lutidine was used (Scheme 37D). The substrates applied were typically enantiopure and it was demonstrated that the stereocenter was not compromised by the reaction conditions. After the second arylation step, the products were obtained in d.r. >20 : 1. Additionally, also the conversion of the *N*-methoxyalkanamide to a methyl ester was demonstrated and could be carried out in a single operation, again without compromising the stereocenter. Aryl- and heteroaryl iodides were the applied coupling partners and the yield range was relatively large spanning from 32–91%. Excellent FG tolerance was reported.

### 7.2. Alkylation and allylation

**Scheme 38:**
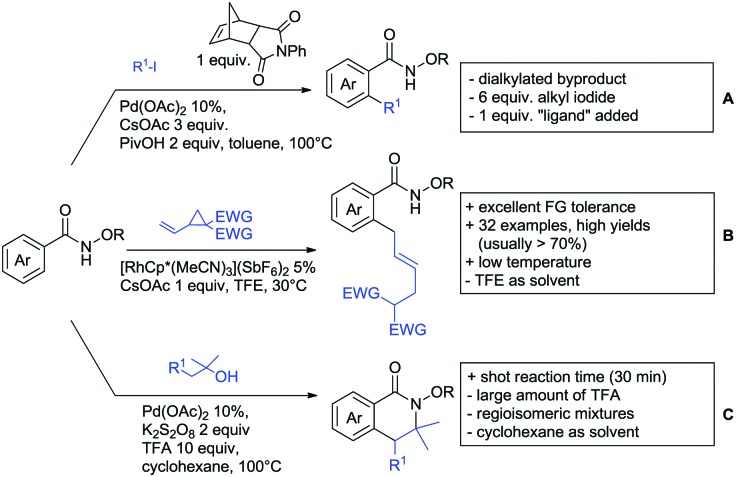
*N*-Alkoxyamide-directed alkylation/allylation reactions.

The group of Huang reported on the alkylation of *N*-methoxybenzamides using alkyl iodides as coupling partners (Scheme 38A).^244^ The reaction was mono-selective for substrates carrying a substituent already in *ortho*- or *meta*-position. In case of *para* substituted compounds or the parent *N*-methoxybenzamide, mixtures of mono- and di-alkylated products were obtained. On average, the mono-alkylated product was favoured in a 6 : 1 ratio, which is remarkable considering the large excess of iodide (6 equiv.). A weakly coordinating ligand was important for achieving this degree of mono-selectivity. Amongst others, bromine as FG was well tolerated in this Pd(OAc)_2_-catalysed protocol. Notably, *N*-methylbenzamide gave the alkylated product only in 17% yield, whereas the *N*-methoxybenzamides gave products in the 60–90% range.

Huang, Li, and Wang disclosed a Rh(iii)-catalysed allylation protocol using 2-vinylcyclopropane-1,1-dicarboxylate (or related compounds) as allyl source (Scheme 38B).^245^ The reaction showed excellent FG tolerance (including Br, I, NO_2_, CN, COOMe) and yields around 80%. A large set of examples was reported and the products showed good *E*/*Z* selectivity (5 : 1 in the worst case, often ∼20 : 1).

The group of Huang reported the synthesis of dihydro-isoquinolinones *via* a Pd(OAc)_2_ catalysed alkylation–cyclisation sequence (Scheme 38C).^246^ Initially, di-*t*butyl peroxide was the alkyl source and the reaction proceeded smoothly in cyclohexane, unfortunately requiring 25 equiv. of TFA. Notably, if *N*-alkyl- or *N*-arylbenzamides were used, the reaction was inefficient, whereas most examples for *N*-alkoxybenzamides gave a yield ∼80%. By adding 2 equiv. of K_2_S_2_O_8_ to the reaction mixture, the peroxide could be replaced with a tertiary alcohol. The reaction with *t*-butanol gave essentially the same yield as with the corresponding peroxide. In cases were one methyl group was replaced by a longer alkyl chain, cyclisation occurred either to the methyl, or the other alkyl group leading to mixtures of products.

### 7.3. Alkenylation

Moving to alkenylation reactions, several more examples as compared to alkylation reactions were reported. They can be clustered into reactions in which the alkenylated product is isolated as the target molecule, and in examples in which the alkenylation is followed by a cyclisation step to an *N*-containing heterocycle, without isolation of the intermediate product.

**Scheme 39:**
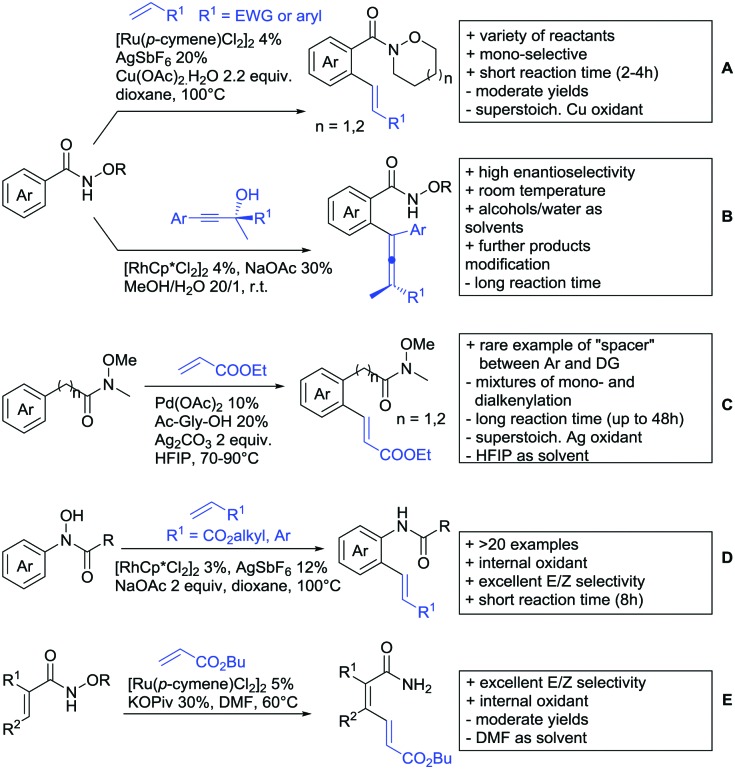
*N*-Alkoxy- and *N*-hydroxyamide-directed alkenylation reactions.

Kapur reported pure alkenylation using cyclic aromatic Weinreb amides as substrates.^247^ The reaction was Ru-catalysed and acrylate or styrene derivatives were used as alkene sources (Scheme 39A). A wide range of electron withdrawing groups on the olefin were accepted, but yields were typically only in the 41–73% range. Cu(OAc)_2_·H_2_O was used as oxidant and AgSbF_6_ as additive. In the end, the authors demonstrated typically further transformations of Weinreb amides and converted them into an aldehyde or ketone moiety.

A special case is the reaction with propargylic alcohols as coupling partners. Ma and co-workers showed that in this case an allene moiety was attached at the *ortho* position to the *N*-methoxybenzamide DG (Scheme 39B).^63^ The most remarkable feature of this transformation was the fact that in case of chiral propargylic alcohols, the allene products are formed in ees between 94–98%. It has to be mentioned that the reaction required long reaction times, typically 72 hours but delivered typical yields ∼70%. Furthermore, the intramolecular cyclisation of the products to several types of heterocycles was demonstrated as well, showing the synthetic potential of the disclosed method.

Alkenylation and also acetoxylation (not shown) of Weinreb amides was reported by Yu and co-workers (Scheme 39C).^248^ This protocol was also applied for other DGs such as ketones (see Section 2.3) and esters (see Section 5.1.2). In case of the Weinreb amides the substrate scope included only FGs which can be considered as innocent (F, Cl, CF_3_, OMe), since they do not undergo any transformations under a wide range of C–H functionalisation conditions. Yields for the alkenylation were high (70–92%) for examples in which no di-alkenylation was possible. In the other cases, mixtures of mono- and di-alkenylated products were obtained. For acetoxylation, the yields were generally ∼10% lower and the same issue with a two-fold functionalisation occurred.

In one case also an *N*-hydroxyanilide has been used as substrate.^249^ Acrylates were used as coupling partners in this Rh catalysed protocol (Scheme 39D). The resulting products were almost exclusively of *E* configuration but in two examples isomerisation occurred giving then *E*/*Z* mixtures. These were the *t*-butyl and ethyl acrylates, however why exactly these two examples showed isomerisation was not explained. Also in this case the final products were amides since the *N*-hydroxy group was cleaved in course of the reaction (internal oxidant).

Zhang and Zhong reported an alkenylation protocol of *N*-methoxy amides, in which the methoxy group is cleaved under the applied Ru catalysed conditions (internal oxidant), leading to alkenylated amides as final products (Scheme 39E).^250^ In this case not aromatic but α,β-unsaturated *N*-methoxyalkancarboxamides were used as substrate leading to the corresponding 2,4-diolefins with excellent *Z*/*E* selectivity often of 99/1. Yields however were low to good (22–70%).

### 7.4. Alkenylation with concomitant cyclisation

Several papers were published in which the alkenylation step is immediately followed by a cyclisation to a nitrogen containing heterocycle.

**Scheme 40:**
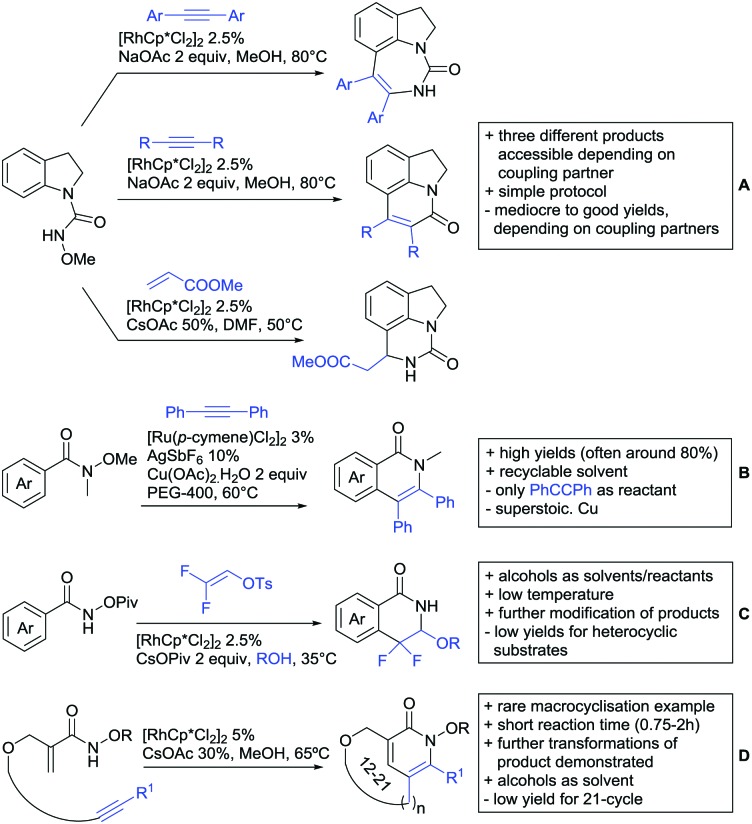
Alkenylation–cyclisation sequences directed by *N*-alkoxyamide/ureas.

Li, Yang, and Zhou showed that indolines carrying an *N*-methoxycarbamoyl in position 1 can be alkenylated with alkynes in position 7 and then further cyclised to 6- or 7-membered rings, depending on the nature of the alkyne (Scheme 40A).^251^ Dialkyl alkynes gave the 6-membered cyclisation product, whereas diaryl alkynes gave the 7-membered derivative. Otherwise, the reaction conditions in this Rh catalysed protocol were identical. Also alkylation with olefins and further cyclisation was reported. For this transformation the additive and solvent had to be changed (from NaOAc and MeOH to CsOAc and DMF) and acrylates were the reaction partners.

A Ru catalysed alkenylation–cyclisation reaction was reported by Bhanage and co-workers in PEG-400 as recyclable solvent (Scheme 40B).^252^*N*-Alkoxy or Weinreb amides were the best substrates and diphenylacetylene was the sole coupling partner and after cyclisation, in which the N–OMe bond is broken, isoquinolinones were the obtained products. Also carboxylic acids were applicable DGs for this reaction sequence giving isocoumarins. If styrenes were the olefin source, no subsequent cyclisation occurred (not shown). For all transformations yields were typically >80%.

Li and Wang investigated the reaction of 2,2-difluorovinyl tosylate with *N*-acyloxybenzamides under various Rh(iii)-catalysed conditions, which gave access to different types of product.^253^*N*-(Pivaloyloxy)benzamides led to 4,4-difluoro-3-alkoxy-3,4-dihydroisoquinolin-1(2*H*)-ones (Scheme 40C). If the pivaloyloxy group was exchanged for a methoxy group, no cyclisation took place but β-fluoride elimination delivered the alkenylated products (not shown). In addition, detailed DFT calculations were carried out in order to elucidate the reaction mechanism.

Finally, Meyer and Cossy reported an *N*-alkoxycarboxamide-directed macrocyclisation with an intramolecularly accessible alkyne (Scheme 40D).^254^ Macrocyclisation reactions are important transformations for natural product synthesis and new methods therefore highly welcome. The formation of 12-, 14, and even a 21-membered macrocyclic pyridinone were demonstrated. For such a challenging transformation, yields were actually quite good, getting as high as 82% in one example. The 21-membered ring was still formed in 32% yield.

### 7.5. Other transformations

**Scheme 41:**
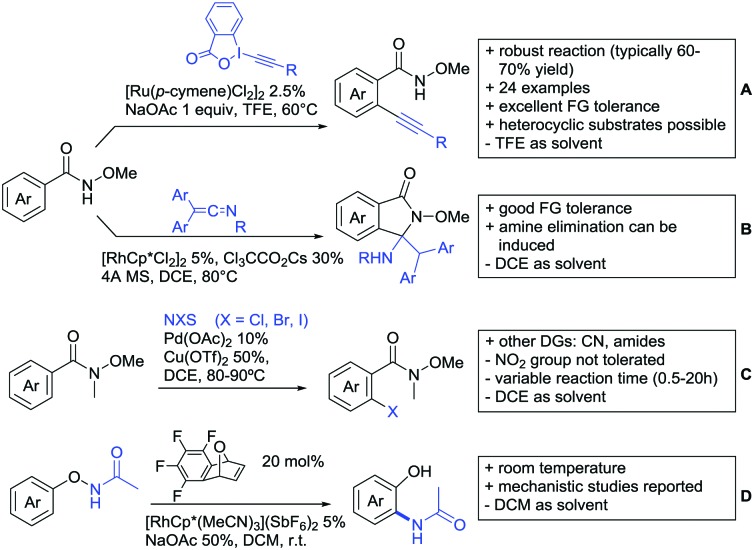
Various transformations directed by *N*-alkoxy- and *N*-aryloxy- and Weinreb amide.

The group of Chien-Hong Chen disclosed a Ru-catalysed alkynylation of aryls and heteroaryls using hypervalent iodine–alkyne reagents (Scheme 41A).^255^ FG tolerance was excellent (including bromine) and yields were typically around 70%.

Ketenimines were used for the annulation of *N*-methoxybenzamides under Rh catalysis (Scheme 41B).^256^ Initially, 3-amino-1,1-diarylmethylisoindolin-3-ones were isolated as products, which can be further converted to 3-(diarylmethylene)isoindolin-1-ones under Lewis acidic conditions *via* elimination of the amine. Using *N*-methoxy-1-naphthamides as substrates, this elimination occurred even spontaneously without any additional Lewis acid.

The halogenation of Weinreb amides (besides nitriles and amides, see Sections 10 and 6.1.6) was reported by Kapur (Scheme 41C).^230^ NXS was used as halide source and 22 examples with yields between 49–81% were reported in this Pd(OAc)_2_ catalysed protocol. The main focus was on bromination, but also iodination and chlorination was demonstrated. The presence of a nitro group shut down the reaction completely.

A quite peculiar transformation was reported by the group of Glorius (Scheme 41D).^257^ They used *N*-aryloxyacetamides as starting materials which were converted to *N*-(2-hydroxyaryl)acetamides. So overall, an intramolecular amidation took place *via* migration of the amide group. The reaction required a bicyclic olefin as co-catalyst which enabled the amide migration. DFT-calculations and experimental mechanistic studies allowed them to propose a mechanism for this transformation. Electron withdrawing groups (CF_3_, COOEt) directly on the aryl ring were not well tolerated, however, if they were separated by an aliphatic spacer yields were again high (typically 70–90%).

## 8. Carbamates

Only a few examples of carbamate-directed C–H activation reactions have been disclosed in the past three years. Hence, it is avoided to divide this part into further subsections. All reported examples deal with activation of C(sp^2^)–H bonds, either of aromatic or olefinic systems.

**Scheme 42:**
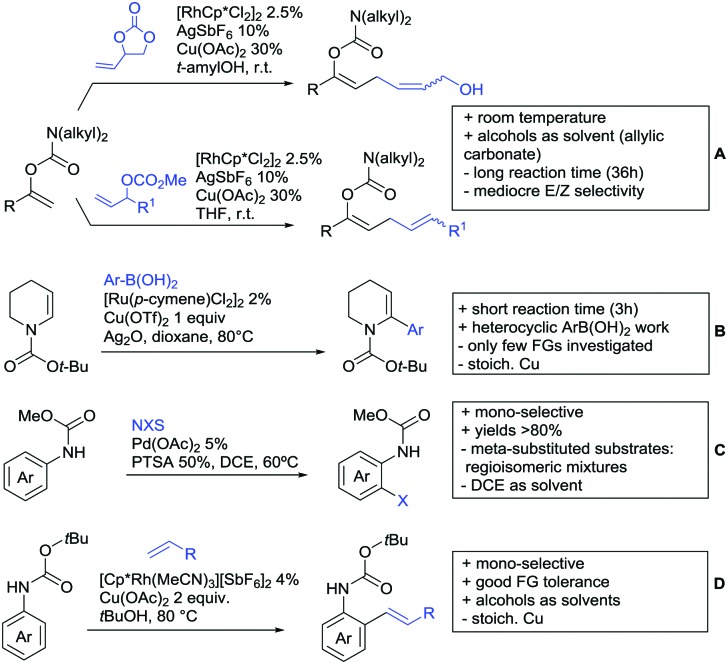
Carbamate-directed transformations.

Kim and co-workers developed an allylation protocol for vinylic C(sp^2^)–H bonds using cyclic or acyclic allylic carbonates as the reaction partner (Scheme 42A).^258^ The reaction was catalysed by [RhCp*Cl_2_]_2_ and performed at room temperature but consequently also took 36 h of reaction time. The yields in this transformation were low to good (28–82%) and in cases were *Z* or *E* products can be obtained, the selectivity is never better than 5 : 1. The same group also reported “alkylation” with maleimides under almost identical conditions.^259^

Boc-protected 2,3-dehydropiperidines can be arylated with arylboronic acids in position 2 using [RuCl_2_(*p*-cymene)]_2_ as catalyst (Scheme 42B).^260^ The method tolerated also heterocyclic boronic acids and delivered good yields, typically >70%. Formyl protection of the dehydropiperidine worked as well.

Halogenation protocols using NXS under Pd catalysis have been reported with a number of different DGs. Also carbamates promote this chemistry as disclosed by Moghaddam and Jafari (Scheme 42C).^261^ The halogenation is mono-selective and the second *ortho* position stayed untouched. In fact, if one *ortho* position carried a substituent already, no reaction occurred. Chlorine, bromine and iodine could be introduced equally well and yields were generally >80%.

Finally, Satoh and Miura disclosed a carbamate directed alkenylation protocol *via* Rh catalysis. The transformation required 2 equiv. of Cu(OAc)_2_ as oxidant, and could be carried out in an environmentally benign alcoholic solvent, namely *t*BuOH (Scheme 42D). It was shown, that after Boc-cleavage and imine formation, a thermal cyclisation to a pyridine ring could be carried out, leading to polyannelated ring systems.^262^

## 9. Urea derivatives as DGs

Urea derivatives have not been used as frequently as DGs as compared to amides or esters, but some progress has been made in the last three years. In several cases, urea derivatives are often tested concomitantly with related DGs such as amides or *N*-alkoxyamides, often in these contributions only for showing broader applicability of a method. In this section, examples will be presented in which urea based DGs make up for a large part of the corresponding contribution and are not only treated as a side note.

**Scheme 43:**
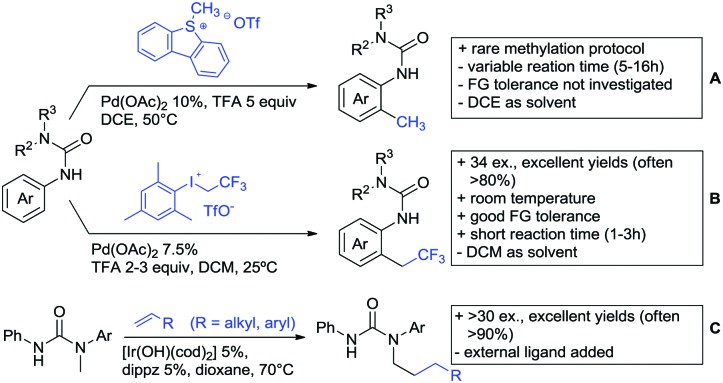
Urea-directed alkylation reactions.

The group of Novak reported the alkylation of amides and urea derivatives using alkyl sulfonium salts as alkylating agents (Scheme 43A).^198^ The most interesting application is in methylation reactions, since methods for introducing the CH_3_ group *via* C–H activation are actually quite rare. The reaction conditions in this Pd(OAc)_2_ catalysed reaction were quite mild (50 °C) but 5 equiv. of TFA were required. It has to be mentioned that FG tolerance was not really investigated in this report and yields were between 48–89%. The same group reported also an *ortho*-trifluoroethylation reaction of *N*-arylureas, where they basically applied a protocol they had previously used for the same transformation with amide substrates (Scheme 24G) and obtained excellent yields (Scheme 43B).^263^

A third alkylation protocol was reported by Nishimura and co-workers.^264^ Mainly starting materials of the type *N*,*N*′-diaryl-*N*-methylurea were used. Alkylation took place at the *N*-methyl and not on the arene system (!), which was an unusual finding (Scheme 43C). Actually, the reaction worked with excellent yields, regularly >90%. More than 30 examples were reported which showed excellent FG tolerance. This Ir-catalysed method used terminal olefins as the starting material and required one free NH group of the urea in order to allow coordination of the Ir species. Then, a 5-membered metallacycle was formed with the metal group before alkene insertion and reductive elimination delivered the final products.

**Scheme 44:**
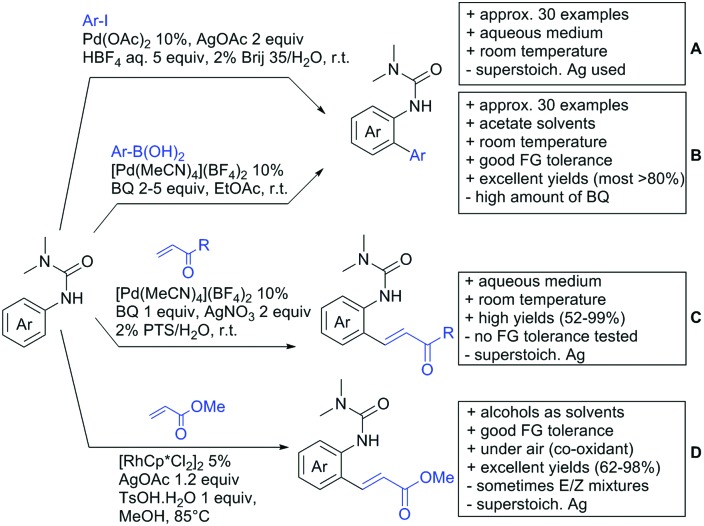
Urea-directed arylation and olefination.

The group of Lipshutz reported Pd catalysed arylation of *N*-aryl-*N*′,*N*′-dimethylureas on the aryl systems.^265^ Depending on the choice of catalyst, they could use either aryl iodides (Scheme 44A, Pd(OAc)_2_) or arylboronic acids (Scheme 44B, [Pd(MeCN)_4_](BF_4_)_2_) as aryl source. Both arylation protocols gave good to excellent yields, whereas the boronic acid protocol gave higher yields in those examples where the same product was formed *via* both methods. Additionally, oxidative alkenylation, *i.e.* Fujiwara–Moritani reaction, was also reported, again with remarkably high yields in most cases. This transformation was particularly interesting since it could be run in water with low amounts of a surfactant (wt 2% PTS) at room temperature as well (Scheme 44C).

Later, a Rh catalysed variant was reported by Yi and co-workers (Scheme 44D).^266^ Also in this case *N*-aryl-*N*′,*N*′-dimethylureas were the starting materials to be alkenylated on the aryl systems. However in this case, the alkenylated urea products were not isolated but further converted by ammonolysis and esterification (MeOH is the solvent in this reaction) to the corresponding *O*-methyl carbamates. Quite good yields (frequently >90%) and substrate scope as well as good *E*-selectivity was observed.

**Scheme 45:**
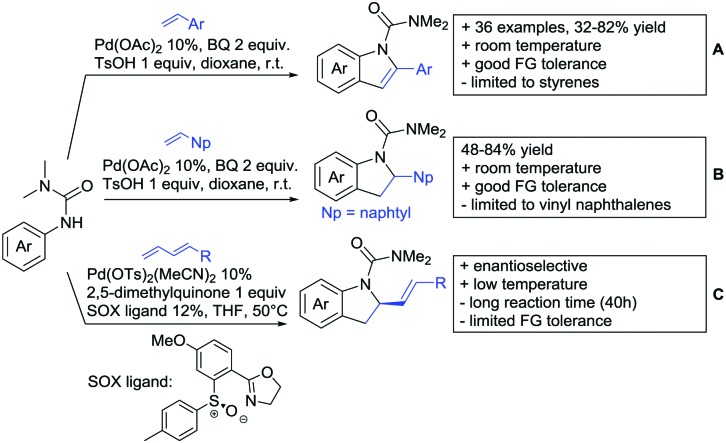
Urea-directed alkenylation–cyclisation sequences.

The group of Jana reported reaction conditions, which gave two different products, just depending on the electronic nature of the applied olefine coupling partner.^267^ Again the same substrate class as in the previous examples was used in combination with styrene derivatives. In case of styrenes with electron withdrawing or only slightly donating (*e.g.* CH_3_) substituents, 2-arylated indoles were the product stemming from *ortho* alkenylation and subsequent aza-Wacker cyclisation as suggested by the authors (Scheme 45A). In case of 4-methoxystyrene or vinyl-naphthalenes, the corresponding 2-arylindolines were the products (Scheme 45B). The authors proposed that a π-benzyl Pd species was formed which was better stabilised by methoxy-arenes or naphthalenes, hence avoiding β-hydride elimination and allowed the immediate attack of this species with the free NH group.

A Pd catalysed alternative indoline synthesis was reported by the group of Han (Scheme 45C).^268^ In fact, the authors disclosed even an asymmetric variant of this transformation using Pd(OTs)_2_(MeCN)_2_ as precatalyst and a chiral sulfoxide-oxazoline (SOX) ligand. 1,3-Butadiene derivatives were the coupling partners of choice. Yields were mainly in the 50% range and the butadiene needed to carry an electron withdrawing group. However, e.r. of up to 95 : 5 were obtained.

**Scheme 46:**
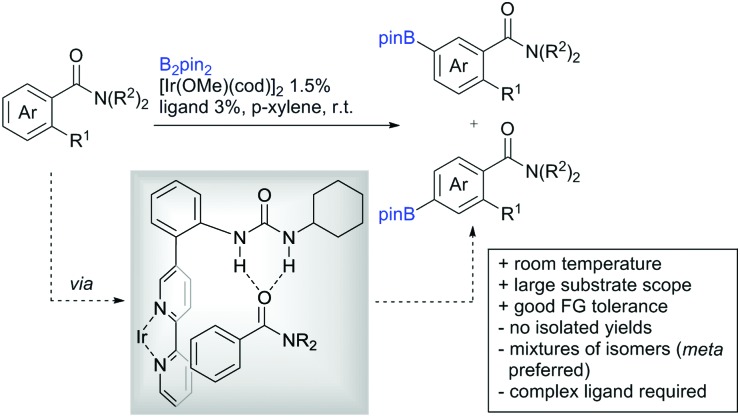
Ligand-assisted *meta* borylation.

A quite special case is the *meta* selective borylation of benzamides as reported by Kuninobu and Kanai.^269^ Of course the question arises why this example is presented in the urea section, and arguments could be found for both cases (and even for putting this example in the amine section). Actually, the amide group does not coordinate the metal, but the two NH groups of a urea derivative, and it is this urea derivative, which has included a bidentate pyridine group which coordinates the Ir catalyst (Scheme 46 bottom). So a secondary weak interaction *via* hydrogen bonding delivers the metal species *via* the ligand in proximity of the C–H bond to be broken. A wide variety of substituted benzamides and also carboxylic acid esters, and phosphoric acid esters (and derivatives thereof) have been used as starting materials demonstrating that the oxygen acting as hydrogen bond acceptor did not need to come from an amide. Interestingly, yields (only determined as NMR yields from *meta*/*para* mixtures) were either ∼50% or >90% whereas electron withdrawing groups lead to the high yields. The *meta* selectivity showed quite some variation ranging from 3.3 : 1 up to >30 : 1.

## 10. Nitriles as DGs

In recent years, the application of nitriles as DGs has been dominated by directing functionalisation of an aromatic system *meta* to the DG.^270^–^273^

The nitrile function typically sits at the end of a spacer and points towards the C–H bond to be activated. Due to its linearity, the nitrile function is perfectly suited for this kind of activation mode. Only few *ortho* directing transformations have been reported. These will be treated here at the beginning of this chapter.

### 10.1. *ortho* directing

**Scheme 47:**
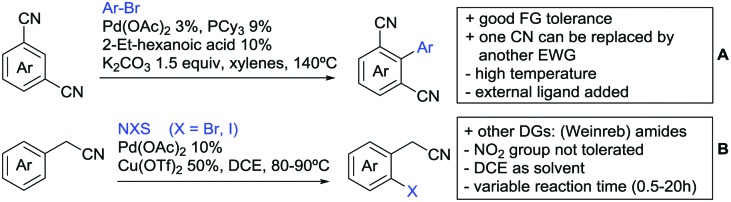
Cyano-directed *ortho* functionalisation.

1,3-Dicyanobenzene has been arylated in position 2 under Pd(OAc)_2_ catalysis using aryl bromides as the aryl source (Scheme 47A).^274^ Arylation occurred in position 2 in yields ranging from 45–81%. Interestingly, 2-bromo pyridine did not react whereas 3-bromo- and 4-bromopyridine did. One cyano group could be replaced by another electron withdrawing group such as Cl, F, or NO_2_. 1,2-Dicyanobenzene reacted as well. Mechanistic studies of a similar arylation protocol were reported by the group of Ren.^275^

Finally, Kapur reported on *ortho* halogenation using several DGs, including benzyl nitriles (Scheme 47B).^230^ The reaction conditions were identical with those reported in previous sections (see Sections 6.1.6 and 7.5) and bromination and iodination with NBS and NIS were reported. As compared to the other DGs, namely Weinreb amides and anilides, yields were pretty much the same for comparable substrates.

### 10.2. *meta* and *para* directing

**Fig. 3:**
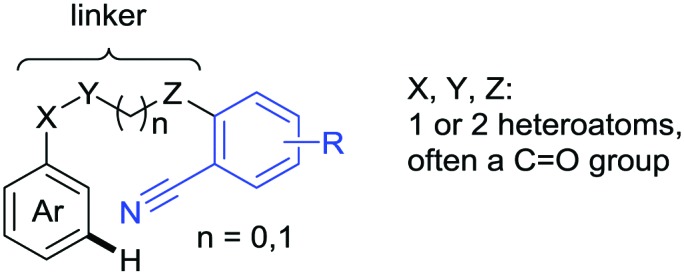
General strategy for designing *meta*-directing moieties.

Further examples directed by the cyano function were mainly *meta* directing. As already mentioned, the cyano group has to be attached to a linker, in order to be able to bring the ligated metal in proximity to the C–H bond in *meta* position. What all examples have in common is the *ortho* substituted benzonitrile marked in blue in Fig. 3. The nature of the linker chain in between can vary. Interestingly, the *ortho* position of the benzonitrile group does not get functionalised and the functionalisation of the more distant ring is favoured! It can be suspected that the metal catalyst gets coordinated by the nitrile nitrogen, which of course disfavours *ortho* functionalisation. The reported protocols differ in the length of the linker chain. However, most often a 3 or 4 atom linker was used whereas typically one or two of them are heteroatoms. Regarding the transformations reported in recent years, there was a strong focus on alkenylation reactions as can be seen in the subsequent section.

**Scheme 48:**
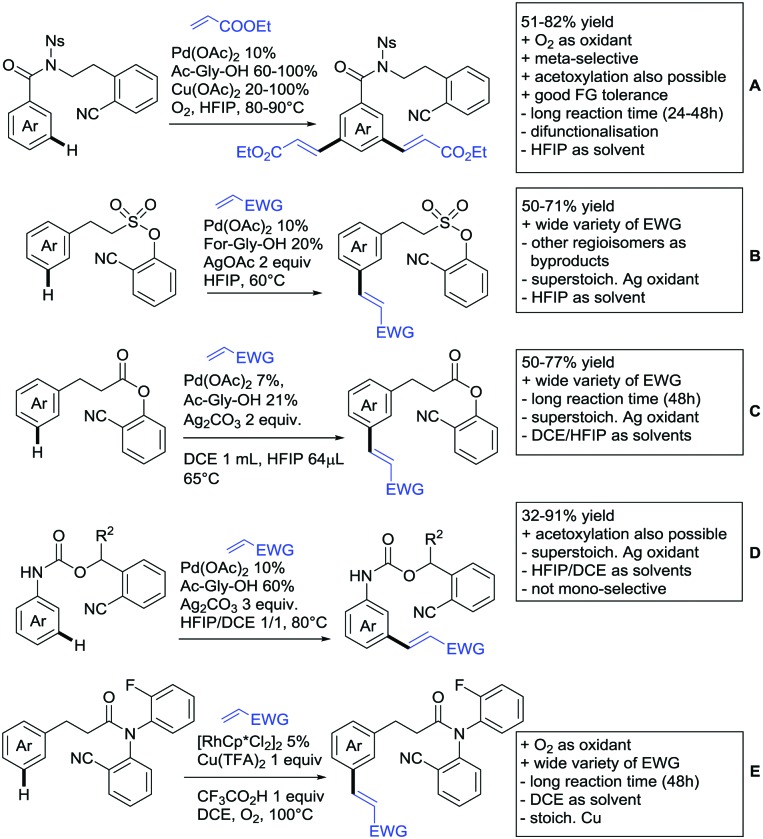
Cyano-directed *meta*-alkenylation.

All examples in Scheme 48 used a four atom linker to connect the benzonitrile group, however, different solutions for this bridge were reported. Shangda Li and co-workers used *N*-nosylated amides (Scheme 48A),^276^ the group of Maiti relied on a sulphonic- or carboxylic acid ester (Scheme 48B and C),^277^ and Gang Li and co-workers applied a carbamate linker (Scheme 48D).^278^ All three protocols used Pd(OAc)_2_ as precatalyst and a mono-protected amino acid (MPAA) mainly Ac-Gly-OH as ligand and olefins carrying electron withdrawing groups as coupling partners in HFIP as solvent. The unique features of this solvent have been highlighted recently.^279^,^280^ Differences can be found in the choice of oxidant. Also the yields of these three protocols were in a similar range. Li's work had the largest substrate scope regarding the aryl ring,^276^ whereas Maiti showed that a wide range of electron withdrawing groups is tolerated in his protocol.^277^ However, the reaction was not exclusively *meta* selective and also other regioisomers are obtained in ratios between 6 : 1–20 : 1. Additionally, Shangda Li and Gang Li showed in their reports also a few examples of *meta* acetoxylation.^276^,^278^

Yu and Sun also used an amide linker, however his protocol is Rh catalysed and did not require Ac-Gly-OH or HFIP (Scheme 48E). In his catalytic system, [RhCp*Cl_2_]_2_ was the catalyst and Cu(CO_2_CF_3_)_2_ the oxidant with additional amounts of CF_3_COOH in oxygen atmosphere and DCE as solvent. Also in this protocol, the reaction was not perfectly *meta* selective and ratios of *meta* : other isomers between 10 : 1–20 : 1 were observed. The yields are in the same range as for the previously discussed examples (49–85%).

**Scheme 49:**
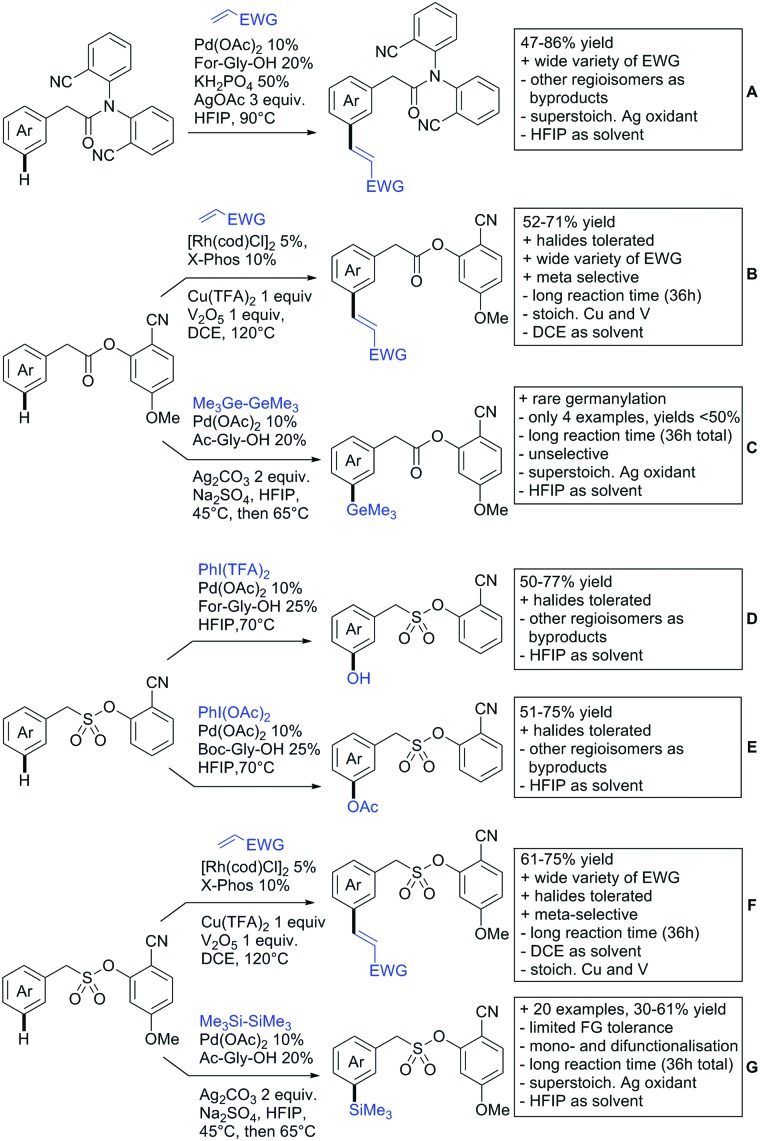
Cyano-directed *meta*-functionalisation.

The same group also showed that the number of linker atoms could be reduced to three as well and still the protocol using Pd(OAc)_2_/MPAA in HFIP as solvent could be applied.^281^ Instead of Ac-Gly-OH, formyl-Gly-OH was the ligand in this case. Again, *meta* selectivity was 20 : 1 at best, and no significant difference between the scope and yield of the above mentioned examples was observed (Scheme 49A).

The group of Maiti also used “shortened linker” DGs for *meta* functionalisation of arenes. In a first publication they demonstrated hydroxylation (Scheme 49D) and acetoxylation (Scheme 49E)^282^ in a second one alkenylation (Scheme 49B and F)^283^ and finally also silylation (Scheme 49G) and germanylation reactions (Scheme 49C).^284^ For the hydroxylation, acetoxylation, silylation, and germanylation protocol the linker was sulphonic acid ester based. For the alkenylation, both sulphonic acid ester and carboxylic acid ester linkers were applied. The olefination reactions showed the same features as for the previously discussed 4-atom linker system. Hydroxylation reactions worked in yields between 50–77% and good to excellent *meta*-selectivity. Acetoxylations worked in the same yield range but with lower selectivity. In silylation and germanylation reactions only between 40–60% yield was obtained.

Again the group of Yu showed alkenylation reactions using a nitrile DG with a 5 atom linker (not shown). The method showed the same features discussed previously: Pd(OAc)_2_, Ac-Gly-OH as ligand and HFIP as solvent. However, Ac-Gly-OH seemed not to be ideal for starting materials already carrying a substituent on the aromatic ring. Hence, Ac-Val-OH was identified as superior ligand in a second screening campaign. The results regarding *meta* selectivity were comparable to those in the examples discussed previously. The same was true for the yields and substrate scope. As an add-on in this publication, significant effort has been put into elucidation of the reaction mechanism *via* experimental and theoretical methods.

**Scheme 50:**
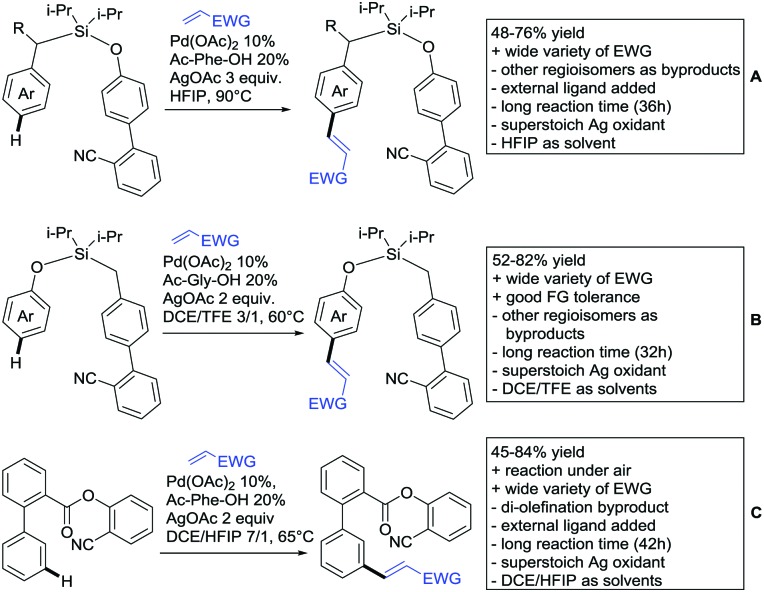
Cyano-directed *para*-(and *meta*-)alkenylation.

The group of Maiti investigated biphenyl-templates as *para*-DGs in olefination reactions (Scheme 50A and B).^285^,^286^ Therein, the directing nitrile moiety was attached to a biphenyl system, which was linked to the arene system to be alkenylated *via* a silyl ether. It was shown that it did not matter which of the aromatic systems was directly linked to the oxygen. Crucial for success was that the silyl moiety in the linker chain was bulky, since this forced the arene and biphenyl system to face into the same direction and hence enabled the nitrile to bring the metal catalyst in proximity of the *para* C–H bond. Yields for both types of substrate were in the synthetically useful range of >50%, in a good number of cases around 70%. Besides the *para*-alkenylated products also other regioisomers were formed favouring the *para* product in ratios between 5 : 1 and 20 : 1. A few examples of acetoxylation were reported as well.^285^

By “inverting” the substrate, meaning that now the nitrile DG is placed on the arene part, *meta* alkenylation of biphenyls was achieved (Scheme 50C).^287^

## 11. Imines as DGs

Imines have been prominently used as DGs in recent years. One large application field was their use as so called “transient” DGs, in which the imine is formed *in situ*, enables a C–H activation reaction, and then gets cleaved again. Reactions such as hydroacylation, hydroarylation, hydroalkylation, arylation, alkenylation, amination, and halogenation have been realised with this approach. Since this field has been reviewed just now,^102^,^103^ these examples are excluded from this chapter and only examples are presented in which the imine is present from the beginning (and eventually cleaved after directing the metal catalyst), or *in situ* formed but then remains in the final product.

### 11.1. Arylation

**Scheme 51:**
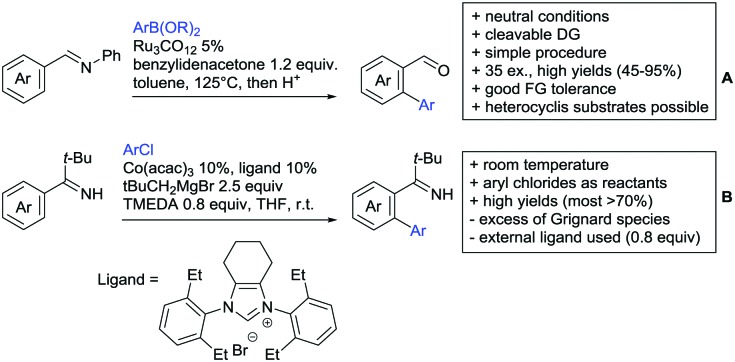
Imine-directed arylation reactions.

Imine-directed arylations have not been the centre point of recent research in the field as can be seen by the small amount of examples. Szostak and co-workers reported a Ru(0)-catalysed protocol which works under neutral conditions (Scheme 51A),^288^ which have been applied previously for C(sp^3^)–H arylation of saturated heterocycles.^289^ Arylboronic acid esters were the aryl source. The protocol gave good to excellent yields (up to 96%) and tolerated the most common FGs. In one example it was shown that the imine could be formed *in situ* as well, which would then fall into the category of transient DGs.^102^,^103^

A second example was disclosed by Yoshikai using Co(acac)_3_ as catalyst and aryl chlorides as aryl source (Scheme 51B).^290^ Both of these features make the protocol highly attractive, with the drawback of the requirement of 2.5 equiv. of a Grignard species and the resulting limited FG tolerance. Still, the protocol worked well on suitable examples with typical yields around 80% and the DG could be converted to a nitrile function.

### 11.2. Alkylations and allylations

Alkylation reactions represent a very special case in imine directed C–H functionalisation. Interestingly, non-precious metals play a dominant role in this field and examples for Fe, Co, and Mn catalysis have been reported.^55^,^61^,^70^,^72^

**Scheme 52:**
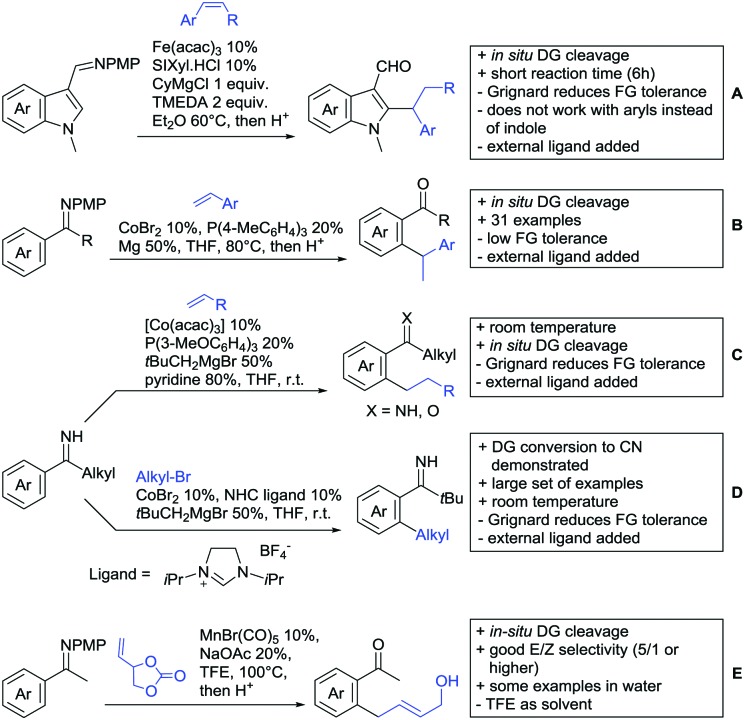
Imine-directed alkylation/allylation reactions.

Yoshikai, one of the pioneers in non-precious metal catalysed C–H activation reported on the C2 alkylation of indoles carrying an imine group in position three (Scheme 52A).^291^ In the same contribution also alkenylation was disclosed, which will be discussed in the subsequent section. As catalyst simple Fe(acac)_3_ could be applied, however an NHC ligand had to be added. One drawback in this type of chemistry was the necessity for a Grignard species, which naturally limited the FG tolerance. Still, in the presented examples good to excellent yields (up to 93%) were reported. Notably, the reaction worked well on indole but was inefficient on simple arene systems, however, a protocol for this type of substrates was reported previously.^292^

This aforementioned protocol also had the limitation of the requirement for Grignard reagents. In subsequent work, they tried to address this issue and it was found that addition of 50 mol% magnesium served the same purpose (Scheme 52B).^293^ However, still only examples with FGs not sensitive to Grignard species were included. Noteworthy, with styrenes as the alkyl source the branched products were obtained, whereas with vinyl-trimethylsilane the linear products were formed, potentially due to the steric demand of the SiMe_3_ group.

The two previous reports always used PMP protected imines as DG. However, the same group also demonstrated that unprotected imines can be used as well if ketimines are used (Scheme 52C).^294^ In fact, in competition experiments between PMP-protected imines and free imines, it was found that the latter reacted preferentially. In the present example vinyl silanes and terminal aliphatic olefins were the coupling partners always giving the linear products in good to excellent yields (up to 99%). In this contribution, the alkylated imines were also isolated in a number of cases. In case of pivalophenone imine, excellent selectivity for mono-alkylation products was observed due to the steric bulk introduced by the *t*-butyl group. In a later contribution, the alkylated imines and their further photochemical transformation to nitriles was the focal point (Scheme 52D).^290^ This time, alkyl bromides were used as the alkyl source and it can be speculated that the alkyl bromides might eliminate to the corresponding olefins *in situ*.^295^ Again, the reactions worked typically with high yields, often in the 70–90% range. In both contributions the presence of a Grignard reagent was required again, leading to the same issues with FG tolerance.

Ackermann and co-workers showed *N*-PMP protected ketamine-directed introduction of a substituted allyl-group stemming from dioxolanone (Scheme 52E).^296^ The reaction was catalysed by MnBr(CO)_5_ and gave the final products in decent yields with an *E*/*Z* selectivity of at least 7.3/1, often >20/1.

### 11.3. Alkenylation

**Scheme 53:**
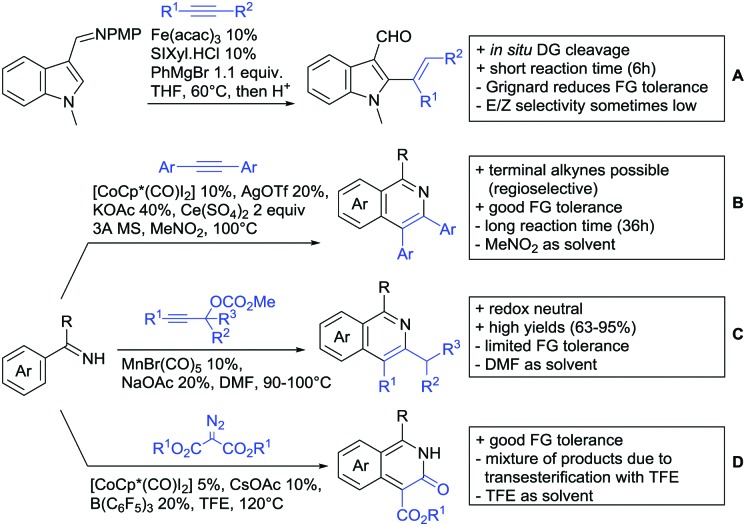
Imine-directed alkenylation and alkenylation–cyclisation reactions.

C2-Alkenylation of indoles was reported by Yoshikai and co-workers under Fe catalysis (Scheme 53A).^291^ Reaction conditions were similar to the ones for the alkylation disclosed in the same contribution. It has to be mentioned that *E*/*Z* selectivity was not good in 3 out of 8 examples and also yields varied between 18–84%, the lowest one being obtained by a dialkyl alkyne.

As we have seen in previous chapters, alkenylation reactions are often followed by a cyclisation reaction with a heteroatom nucleophile leading to heterocyclic systems. Imine DGs showed the same behaviour, since the lone pair of nitrogen could attack suitable groups which were introduced *via* the C–H activation step.

Several isoquinoline syntheses have been reported. Li and Wang developed a reaction between aromatic imines and alkynes using Cp*Co(CO)I_2_ as catalyst (Scheme 53B).^297^ In most cases the reaction was quite efficient giving yields >70%. Most examples used diaryl alkynes, but also dialkyl or terminal alkynes were applied successfully. Additionally, the transformation showed good FG tolerance. Later, a slight variation of that protocol was reported as well.^148^

Another base metal catalysed isoquinoline synthesis was reported by Glorius and co-workers, this time using MnBr(CO)_5_ under redox neutral conditions (Scheme 53C).^298^ As coupling partners, tertiary propargylic carbonates were applied. The authors proposed that after C–H activation and olefination a carbonate elimination took place to deliver a reactive allene species, which was intramoleculary attacked by the imine to deliver the isoquinoline products. Yields were good to excellent, however not much FG tolerance was explored.

Using diazomalonates as reaction partners, isoquinolin-3-ones were the obtained products, again under Co catalysis (Scheme 53D).^299^ The reaction performed in TFE and unfortunately, transesterification of the product took place leading to mixtures of compounds in an otherwise high yielding protocol.

**Scheme 54:**
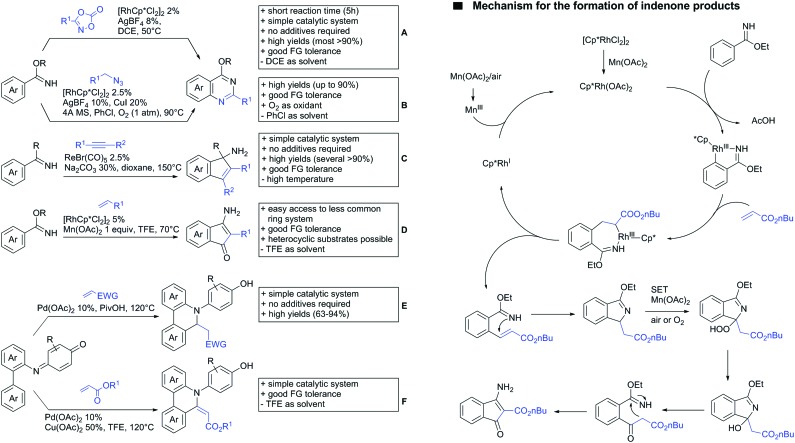
Imine-directed functionalisation–cyclisation sequences.

Almost simultaneously, Zhu^300^ and Jiao^301^ reported two different protocols for the synthesis of quinazolines starting from carboximidates (Scheme 54A and B). Both groups relied on the same catalyst, namely [Cp*RhCl_2_]_2_ but used different sources for the nitrogen ending up in position 1 of the quinazoline. Zhu used dioxazolones whereas Jiao preferred benzylic azides in most cases. Comparing both protocols it can be seen that the use of dioxazolones gives a bit better yield, but also with azides >70% are achieved in almost every example. FG tolerance was basically identically good. Overall, Zhu's protocol was operationally simpler as could be seen by comparing the reaction conditions in Scheme 54A and B.

A rare case of rhenium catalysis was reported by Wang and colleagues (Scheme 54C).^302^ In this contribution, an imine starting material was again reacted with an internal alkyne, as seen previously in isoquinoline syntheses. However, under ReBr(CO)_8_ catalysis a [3+2] cycloaddition took place and the imine nitrogen did not end up in the ring but in position 1 of an indenamine product. The reaction worked well with a number of alkynes (diaryl, dialkyl, and aryl–alkyl). Good to excellent yields were obtained (up to 98%) in this operationally simple protocol.

A second protocol in which the imine nitrogen ended up as amine function in an exocyclic position was disclosed by Zhang and co-workers.^303^ They reported that carboximidates could be alkenylated in *ortho* position using acrylates and [Cp*RhCl_2_]_2_ as catalyst (Scheme 54D). Until this point, this would be a quite standard transformation, however the consecutive sequence to the indenone products was quite interesting. It was proposed that an initial intramolecular cyclisation led to an 1*H*-isoindole intermediate which was oxidised by Mn(OAc)_2_*via* a single electron transfer forming in presence of O_2_ a peroxide intermediate (detected by LC-HRMS). This intermediate eventually ring opened and cyclised again, this time to the final product (Scheme 54, right). For a detailed discussion of this pathway we refer to the original paper.

The group of Jun reported a similar cyclisation, however without the subsequent oxidation-ring opening-cylisation sequence and hence delivering 1*H*-isoindoles as the products (not shown).^304^

Also iminoquinones have been used as DG, which have certainly different properties than ordinary imines, still this example is presented in this section (Scheme 54E and F).^305^ The reaction allowed fast access to a tricyclic system involving a number of olefins carrying different electron withdrawing groups. Depending on the catalytic system, different products were obtained. If only Pd(OAc)_2_ was the catalyst, the saturated products of Scheme 54E were isolated, whereas if additionally 0.5 equiv. Cu(OAc)_2_ oxidant was present, the products with an exocyclic double bond were formed (Scheme 54F), both in high yields.

**Scheme 55:**
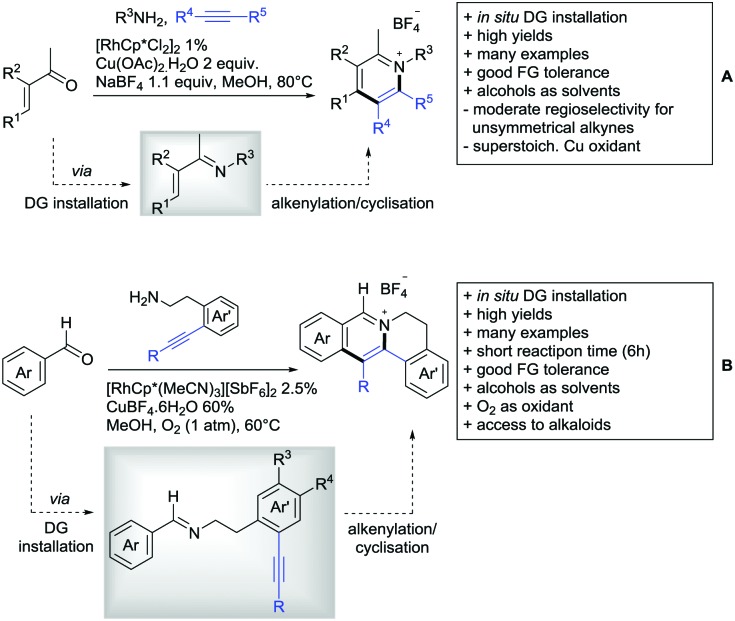
*In situ* formed imine DGs and subsequent cyclisation.

In all previously discussed examples the imine group was already present in the starting material. There were also examples reported in which the imine was only formed *in situ* and then cyclisation took place to pyridium derived heterocycles. Besides the starting materials in Scheme 55, the imine intermediates are shown as well.

Both contributions in this field stem from the group of Cheng and run under Rh catalysis.^306^,^307^ In the first report, a three component reaction was carried out between a ketone or an aldehyde, an amine, and an internal alkyne. *Via* the imine intermediate, the desired pyridinium salts were formed quite efficiently with typical yields of >80%.^306^ The second protocol was only a two component reaction, because the amine carried already an alkyne function for a subsequent intramolecular cyclisation.^307^ Also for this much more complex ring systems, good to excellent yields were reported surpassing 80% in almost every example. *Via* this route, facilitated access to protoberine alkaloids was granted.

## 12. Oximes and oxime derivatives

Oxime containing DGs are quite frequently applied and used in several variants, namely ketoximes, aldoximes and ethers and esters thereof. In 2016 the subject has been comprehensively reviewed,^308^ but since then again a good number of examples have been reported, which are now covered in this section. In several cases, besides the oximes also N-heterocyclic DGs have been tested in the same transformation. However, this chapter focuses solely on the oxime examples.

### 12.1. Arylation

Arylation reactions are usually amongst the most detailed studied transformation with any DG. For oximes, only a few examples have been reported in the last three years.

**Scheme 56:**
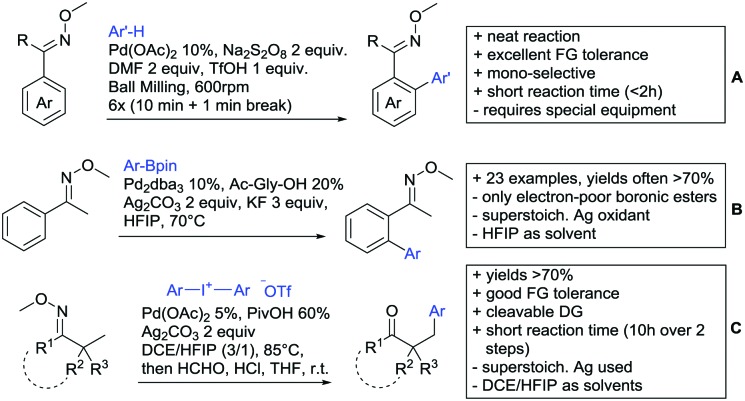
*O*-Methyl oxime-directed arylation reactions.

The group of Xu developed a site-selective biaryl synthesis *via* dehydrogenative C–H/C–H arylation under ball milling conditions (Scheme 56A).^309^ Mechanochemistry is an interesting, yet underrepresented method to introduce energy into a reaction system. In a dehydrogenative approach, the question of regioselectivity is a major issue in cases were substituted arenes are the coupling partners. In this protocol, arenes bearing electron donating groups showed excellent *para* selectivity, whereas arenes with electron withdrawing groups such as acetophenone or methyl benzoate gave good *meta* selectivity, presumably because the *meta* position is more reactive towards S_E_Ar reactions. Overall, the transformation showed excellent FG tolerance and gave yields in the 50–80% range.

Pd-Catalysed *ortho*-C–H arylation of acetophenone oxime ethers with aryl boronic acid esters was reported by Lee and co-workers (Scheme 56B).^310^ The corresponding biaryl products were obtained in yields between 42–80%. Interestingly, besides the parent phenyl boronic acid ester, only derivatives carrying electron withdrawing substituents (CF_3_, NO_2_, COOEt) were applied. Noteworthy, the boronic acid ester can be used as crude material stemming from an Ir catalysed direct borylation.

The β-arylation of oxime ethers using diaryliodonium salts was developed for C(sp^3^)–H bond activation by Chen and co-workers (Scheme 56C).^311^ The arylated oxime ethers were not isolated but immediately hydrolysed to the corresponding ketones. The reaction worked for a wide variety of ketoximes and generally gave yields around 80%. Most interestingly, the method was also applied to the arylation of complex natural product derived molecules (steroids).

### 12.2. Alkylation

**Scheme 57:**
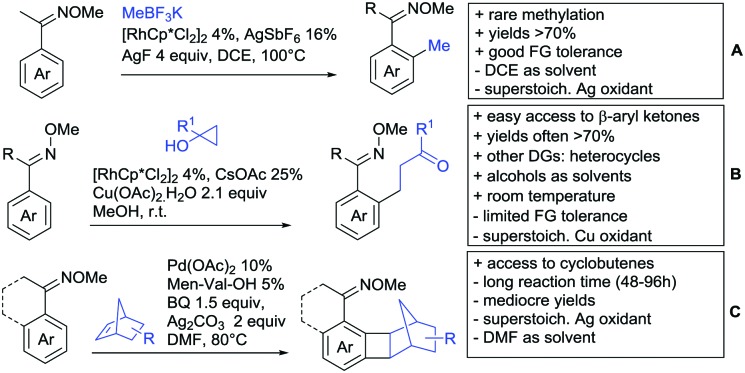
*O*-Methyl oxime-directed alkylation reactions.

Only few alkylation reactions have been reported in the last three years. Li and co-workers reported Rh(iii)-catalysed direct alkylation of arenes using commercially available alkyl trifluoroborates (Scheme 57A).^312^ The method is especially interesting, since it allows simple methylation reactions. Yields were good, between 64–90% but only electron withdrawing FGs were reported amongst the examples (CN, NO_2_, CF_3_, COOEt).

*Via* ring opening of cyclopropanols β-aryl ketones were made accessible by the group of Li.^313^ 16 examples were presented but not much FG tolerance was explored (Scheme 57B).

An alkylation in the broader sense was the synthesis of benzocyclobutenes *via* a Pd catalysed reaction with norbornenes (Scheme 57C).^314^ Norbornene is a quite useful auxiliary in C–H activation chemistry, for example in the Catellani reaction.^11^,^315^ However, in the present transformation, the norbornene was not an auxiliary but a reaction partner and remained in the final products.

### 12.3. Alkenylation

*ortho*-Directed alkenylation reactions have been exploited extensively with a number of different DGs. As discussed in previous chapters, the alkenylation reaction was often immediately followed by a cyclisation towards a (hetero)cyclic ring system. Also with oxime derived DGs this behaviour was observed.

**Scheme 58:**
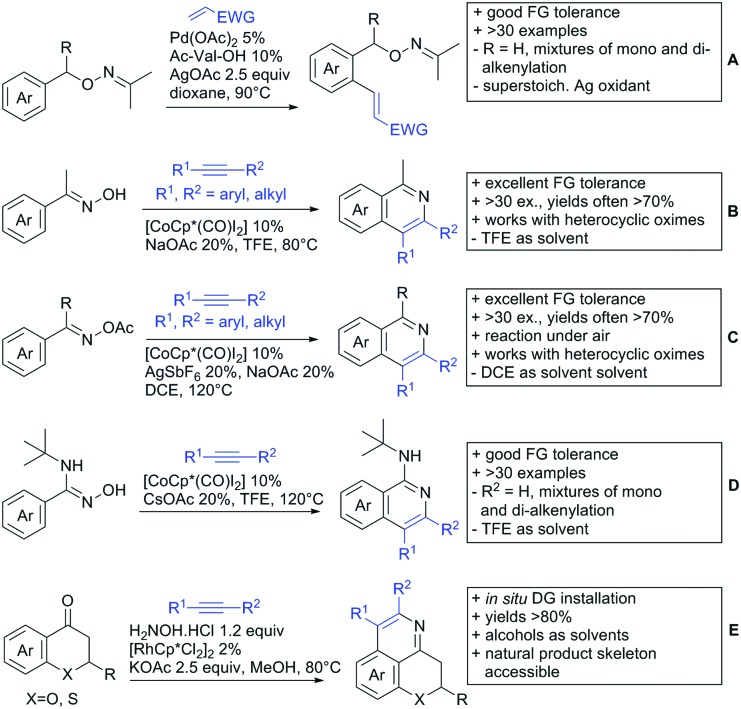
Alkenylation reactions and alkenylation–cyclisation sequences directed by oximes and derivatives.

However, the first example to be discussed was dedicated to an alkenylation reaction in which no further cyclisation occurred. The group of Zhao reacted acrylates with benzylic alcohols masked by acetone oxime ethers under Pd catalysis (Scheme 58A).^316^ The resulting alkenylated products were formed *via* a six- or seven-membered *exo*-acetone oxime ether palladacycle, in contrast to the often dominating 5-membered metallacycles. The reason why in this example no cyclisation occurred could be found in the architecture of the substrates. In this specific case, oxime benzyl ethers were the starting materials and olefination took place on the benzyl system. Hence, an intramolecular attack of the oxime nitrogen was disfavoured and the alkenylated products were stable.

Several isoquinoline syntheses have been reported in recent years. Basically simultaneously, the groups of Sundararaju^317^ and Ackermann^318^ disclosed [Cp*Co(CO)I_2_]-catalysed alkenylation/cyclisation sequences of oxime derivatives and diaryl alkynes. In the Sundararaju protocol, free OH ketoximes were used as substrates (Scheme 58B), whereas in Ackermann's transformation *O*-acetylated oximes were used (Scheme 58C). Both moieties act as internal oxidant in the respective transformations. The reaction conditions were almost identical, with the difference that the Ackermann protocol required AgSbF_6_ as additive whereas in Sundararaju's procedure yields were better in its absence. Both transformations showed excellent FG tolerance and high yields.

An almost identical reaction was reported by Chen with the addition that besides oximes also *N*′-hydroxybenzimidamides were used as DG which then delivered 1-amino-isoquinolines as products (Scheme 58D).^319^

Hua and co-workers developed an alkenylation–cyclisation sequence in which the oxime DG is formed *in situ via* reaction of a carbonyl group with hydroxylamine hydrochloride (Scheme 58E).^320^ Starting from chroman-4-ones, tricyclic fused pyridines were easily accessible, which allowed rapid access to the skeleton of cassiarins, a family of alkaloids with antimalarial activity. Yields were typically >80% and a hydroxyl and bromine function were tolerated without any loss in efficiency.

### 12.4. Acylation

Several examples for *O*-Me ketoxime-directed acylation of π-systems, aromatic and olefinic ones, have been reported.

**Scheme 59:**
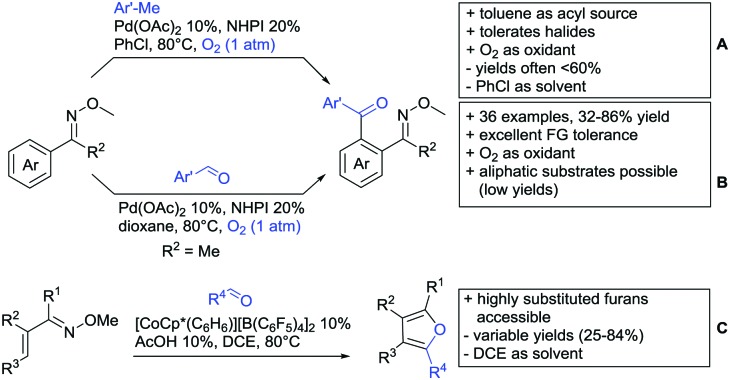
*O*-Methyl oxime-directed acylation and acylation/cyclisation reactions.

The group of Jiao reported acylations of aryl systems using toluene under aerobic conditions as the acyl source, leading to benzophenone derivatives (Scheme 59A).^321^ This Pd(OAc)_2_ catalysed process is quite attractive since toluene is a simple, cheap, and stable starting material. The reaction required *N*-hydroxyphthalimide (NHPI) as additive which induced in presence of oxygen a radical process leading to the oxidation of toluene to a benzoyl radical, which could then be transferred to the *ortho* position of the oxime. Yields were in the 42–81% range and the most interesting FG tolerated was bromine, since that leaves a handle for further reactions.

The same group reported a similar approach, this time starting from benzylic alcohols or benzaldehydes, again with NHPI under aerobic conditions (Scheme 59B).^322^ This modification showed broader FG tolerance with similar yields and also examples of aliphatic aldehydes were reported, allowing also the synthesis of aryl alkyl ketones.

The acylation of α,β-unsaturated *O*-methyl ketoximes with benzaldehydes resulted overall in an interesting new method for the synthesis of highly substituted furan derivatives (Scheme 59C).^323^ Highly functionalised furans were synthesised from α,β unsaturated *O*-methyl oximes by using Co catalysed aldehyde addition and cyclative capture. Twelve examples were prepared tolerating several important FGs (*e.g.* Br, COOR) with moderate to good yields (25–84%).

### 12.5. C–O bond formation

A good amount of oxime-directed C–O bond formations have been disclosed in recent years. Acetoxylation, hydroxylation and alkoxylations have been reported, all using Pd catalysis and various oxidants.

#### 12.5.1. Acetoxylation

**Scheme 60:**
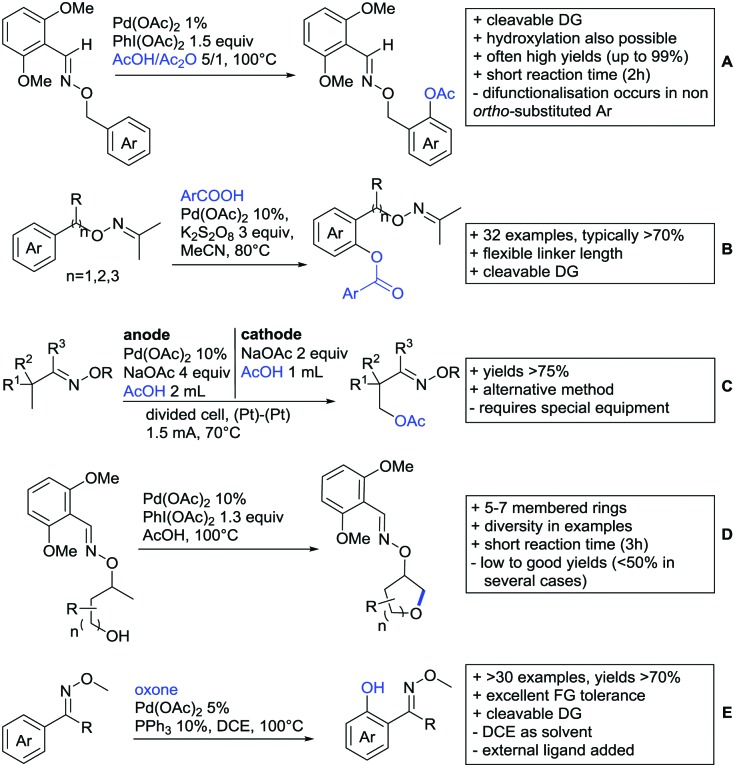
C–O bond forming reactions directed by oxime derivatives.

Dong and co-workers used 2,6-dimethoxy benzaldoxime as an efficient *exo*-type DG for arene C(sp^2^)–H acetoxylation (Scheme 60A).^324^ In this case, Pd(OAc)_2_ was used as catalyst and PhI(OAc)_2_ as the oxidant in a mixture of AcOH/Ac_2_O as solvent. A low catalyst loading of 1 mol% was sufficient to obtain the desired products in good to excellent yields in cases where only mono-acetoxylation was possible. If there was no sterics induced preference for a single reaction, mixtures of mono- and diacetoxylation products were obtained.

Using substituted benzoic acids, Liao and Ji developed a mono-selective *ortho*-aroyloxylation protocol of *O*-benzyl acetoximes, again catalysed by Pd(OAc)_2_ (Scheme 60B).^325^ The benzoic acid as well as the benzyl part could carry common FGs and the transformation robustly gave 60–80% yield. As oxidant simple K_2_S_2_O_8_ could be applied. In two examples also aliphatic acids were used, which worked as well as the benzoic acid derivatives.

An alternative to commonly used oxidants in organic chemistry was used by the group of Mei. In their acetoxylation of the β-position of an *O*-alkyl ketoximes, they relied on electrochemical oxidation (Scheme 60C).^326^ They described a large set of examples and compared the yields with an alternative oxidant, namely NaNO_3_/O_2_. In most examples, the electrochemical oxidation proved to be superior, giving yields >70% in many cases. Eventually this report inspires more research in this direction since replacing typical strong oxidants can be quite useful.

#### 12.5.2. Hydroxylation and alkoxylation

Intramolecular alkoxylation towards saturated 5-, 6-, and 7-membered oxygen heterocycles has been reported by the group of Dong (Scheme 60D).^327^ As in their previously discussed acetoxylation protocol, they used Pd(OAc)_2_ as catalyst and PhI(OAc)_2_ as oxidant. The efficiency of this intramolecular cyclisation was dependent on the relative stereochemistry of the substrates. If anti diol derivatives were used, the yield was significantly higher as compared to the syn diastereoisomers. This was a nice extension to the repertoire of cyclisation reactions toward saturated O-heterocycles.

Pd-Catalysed *O*-methyl oxime-directed *ortho* C–H hydroxylation of arenes was reported by the group of Jiao under neutral conditions (Scheme 60E).^328^ Simple oxone was used as oxidant and also the catalyst system was simple (Pd(OAc)_2_ + PPh_3_). Overall, more than 40 examples were reported demonstrating the excellent FG tolerance and the high yields (typically 70–90%) in this protocol.

### 12.6. C–N bond formation

**Scheme 61:**
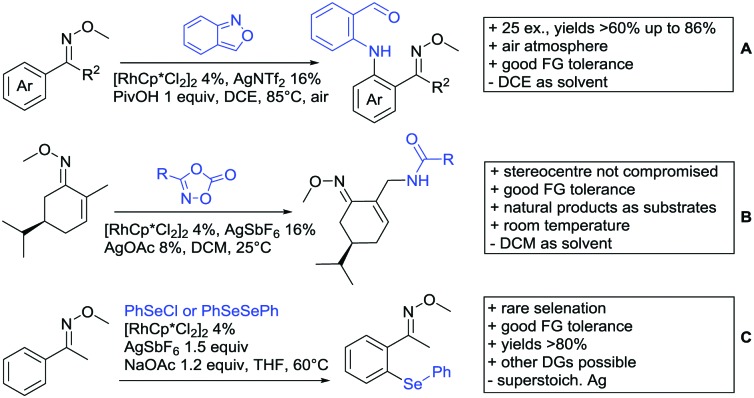
C–N and C–Se bond forming reactions directed by *O*-methyl oximes.

Another interesting transformation developed by Jiao is the amination of oxime ethers with anthranil (Scheme 61A).^329^ Anthranil is here a quite special reagent, since it gets ring opened and overall delivers a 2-formyl aniline moiety into the *ortho* position of the substrate. So basically two FGs are introduced at once. The reaction proved to be quite robust with yields between 62–86%. Regarding FGs, besides MeO and halides no further moieties were investigated, but the oxime DG can be replaced also by N-heterocycles.

In recent years, 3-substituted-1,4,2-dioxazol-5-one has established itself as a good source for the amide group in C–H activation chemistry. The group of Li also made use of this reagent in the amidation of CH_3_ groups in carvone derived oxime methylether (Scheme 61B).^330^ The most interesting example was the late stage functionalisation of natural products, namely the oxime ether of (–)-santonin which worked with a number of 3-substituted-1,4,2-dioxazol-5-ones in 64–94% yield.

Besides acylations, the group of Jao disclosed also *ortho* nitration of aromatic oxime ethers using *t*-butyl nitrite as NO_2_ source under radical conditions (not shown).^321^ Acetophenone oximes (23 examples) bearing substituents such as Me, OMe, *t*-Bu, Br, and F were successfully converted into the corresponding *ortho*-nitrated products.

### 12.7. C–Se bond formation

Selenium-containing organic compounds are not very common and hence methods for introducing selenium are relatively rare. However, Wan and Li developed an aromatic oxime ether-directed Rh(iii)-catalysed selenylation protocol of arenes (Scheme 61C). As selenylating reagents, electrophilic selenyl chlorides and diselenides were used. The catalytic system was highly efficient under mild conditions and delivered the corresponding products in typically >80% yields.^331^

## 13. Hydrazine and hydrazone derivatives as DGs

Hydrazines and hydrazones are not frequently applied as DGs in C–H activation chemistry. In most cases, it is not the parent FG which acts as DG, but substituted (or protected) variants thereof.

**Scheme 62:**
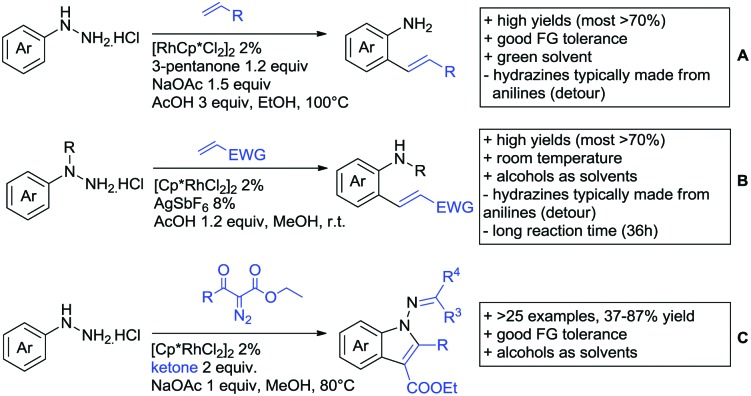
Hydrazine·HCl- and hydrazine-directed transformations.

For hydrazines, few examples have been reported in which simple aryl hydrazines were the substrates. In the first one, the group of Cheng developed an alkenylation protocol in which the final products are *ortho* alkenylated anilines (Scheme 62A).^332^ The actual DG is a hydrazone, since 1.2 equivalents of ketone, namely 3-pentanone, transform the hydrazine *in situ* to a hydrazone. Only then *ortho* C–H insertion by Rh took place and the olefination occurred with styrene derivatives or acrylates in quite good yields (>70%). The catalyst was then also involved in the cleavage of the N–N bond and formation of the final aniline products.

A similar olefination reaction has been disclosed by Zhu and co-workers, however in this example there was no ketone added and hence it is the hydrazine itself which exhibited the directing effect (Scheme 62B).^333^ With acrylates as coupling partners again *ortho* alkenylated aniline derivatives were the final products and also in this protocol yields were very good, typically >80%. Additionally, the FG tolerance was also significant. Alternatively, alkyl–aryl alkynes could be reacted under the same reaction conditions. In these reactions indoles were obtained as products *via* a cyclisation after the olefination step. Interestingly, the aryl group always ended up in position 2 and the alkyl group in position 3 of the indole derivative.

Furthermore, Cui and co-workers developed a synthesis of 1-aminoindoles *via* a Rh-catalysed intramolecular three component annulation (Scheme 62C).^334^ The aryl hydrazines were used as their HCl salts together with ethyl diazoacetoacetate and ketone using [Cp*RhCl_2_]_2_ as catalyst. According to the proposed mechanism, the hydrazine formed with the ketone the corresponding hydrazone and it was actually the sp^2^ hybridized nitrogen of the hydrazone which directed the Rh to close proximity to the *ortho* C–H bond. This allowed the transfer of the ethyl acetoacetate to this position and cyclisation with the NH group delivered the final products. The transformation showed broad FG tolerance with typical yields between 50–80%.

**Scheme 63:**
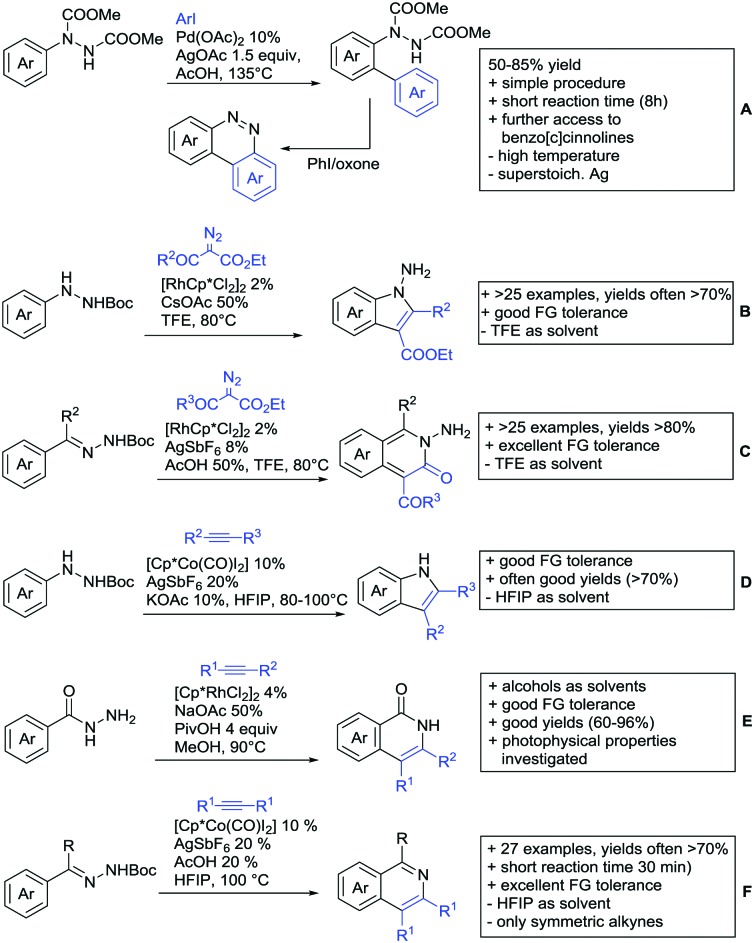
Miscellaneous transformations directed by hydrazine/hydrazide/hydrazone derivatives.

Reddy and co-workers reported a direct arylation protocol of 1-arylhydrazine-1,2-dicarboxylates with aryl iodides and Pd(OAc)_2_ as catalyst (Scheme 63A).^335^ The arylation worked quite well giving yields between 50–86% and some FG tolerance, most importantly a second iodine. The obtained products can be cyclised under oxidative conditions to the corresponding benzo[*c*]cinnoline derivatives.

Basically simultaneously to Cui's indole synthesis, the group of Zhu published another variant of this type of reaction. Starting from 1-aryl-2-Boc-hydrazines and diazodiesters or diazoketoesters 1-aminoindoles were obtained (Scheme 63B).^336^ The catalytic system was quite similar and a series of examples with high yields and good FG tolerance was reported. When they used *N*-Boc aromatic hydrazones as substrates, isoquinoline-3-ones were the products (Scheme 63C). Excellent yields were obtained using 0.5 equiv. AcOH as additive and slightly lower yields when LiOAc was used instead. More than 30 examples were reported with most yields in the 90% range.

Another indole synthesis starting from hydrazines, this time *N*-aryl, *N*′-Boc hydrazines, was reported by the group of Glorius under [Cp*Co(CO)I_2_] catalysis (Scheme 63D).^337^ Mainly symmetric diaryl alkynes were the coupling partners but in some cases also unsymmetrically substituted (*e.g.* aryl–alkyl) ones were used. The reaction showed good FG tolerance and also good to excellent yields, often >85%.

Using [Cp*RhCl_2_]_2_ benzoyl hydrazines have been transformed to isoquinolones *via* the reaction and subsequent cyclisation with diaryl alkynes (Scheme 63E). The resulting products have large conjugated π-systems and the authors also investigated their photophysical properties.^338^

The group of Zhu applied the starting material mentioned in Scheme 63C again, this time in a reaction with diaryl alkynes under [Cp*Co(CO)I_2_] catalysis (Scheme 63F).^339^*Via* this method, 3,4-diarylated isoquinolines were obtained as products. Again, this is a well working reaction with good FG tolerance. The group of Sun disclosed a similar reaction under Rh catalysis (not shown).^340^

**Scheme 64:**
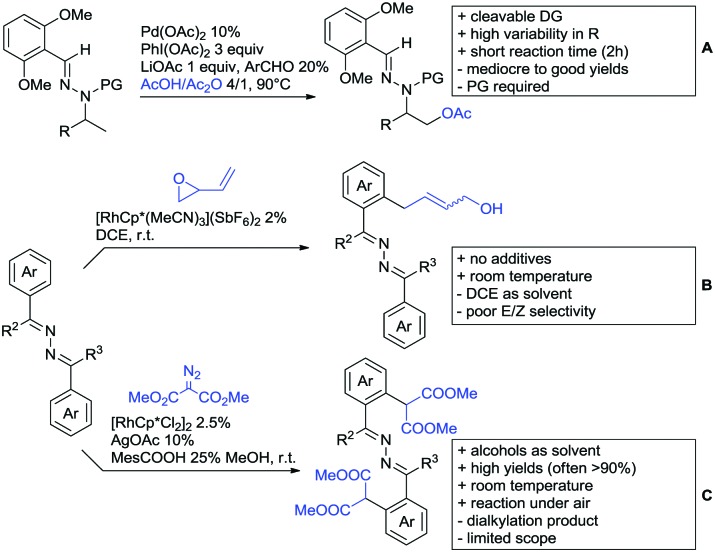
Hydrazone and dibenzylidenehydrazine-directed transformations.

So far all examples dealt with C(sp^2^)–H activation. Dong and co-workers could also realise a C(sp^3^)–H activation, namely a Pd(OAc)_2_ catalysed acetoxylation (Scheme 64A).^341^ As substrates served *N*2-protected hydrazones derived from 2,6-dimethoxybenzaldehyde and PhI(OAc)_2_ was the acetyloxy source. As *N*-protecting group 5 different sulfones were used and acetoxylation occurred in β-position. Quite a range of substrates were subjected to the reaction conditions and the typical yield was in the 50–70% range.

Dibenzylidenehydrazines can be considered as a hydrazone derivative as well. Two transformations on this type of substrate have been reported since 2015, all under Rh catalysis. Zhu reported the reaction with vinyloxirane leading to unsaturated alcohols (Scheme 64B).^342^ Interestingly, only mono-functionalisation occurred with 1.2 equiv. vinyloxirane.

The second procedure used diazodiesters as coupling partners and with 2 equivalents, difunctionalisation on both aromatic rings was obtained in good to excellent yields (Scheme 64C).^343^

## 14. Bidentate DGs

In this section, recent advances in the astute use of bidentate DGs in C–H bond functionalisation will be depicted. Indeed, from the seminal work of Daugulis on the use of the amide derived from 8-aminoquinoline as a bidentate chelating auxiliary,^344^ the scientific community put a lot of efforts in the design and use of a large panel of bidentate groups considerably enlarging the current tool-box of C–H bond functionalisation reactions. Consequently, over the years, a myriad of transformations was developed playing with the unique properties of various bidentate DGs and were already abundantly described in the literature.^57^,^108^,^345^–^350^ Indeed, such bidentate DGs appear today as key tools to achieve efficient transition metal catalysed directed C–H bond activation and were even successfully employed in strategies involving transient DGs, which will not be depicted in this review.^103^,^351^–^359^ In this section, an overview of the major contributions made in the 2015–2017 period will be provided. In particular, this part will focus on the recent advances made in transition metal-catalysed bidentate group-directed functionalisation of both aromatic and aliphatic compounds. At first, transition metal catalysed C–H bond functionalisation involving *N*,*N*-bidentate DGs will be discussed. Then, a focus on the promising, albeit still less studied, *N*,*S*- and *N*,*O*-bidentate DGs will be reported.

### 14.1. *N*,*N*-Bidentate DGs

Among all bidentate DGs, the *N*,*N*-bidentate ones represent the most commonly used auxiliaries for directed C–H bond activation.

#### 14.1.1. Amides derived from 8-aminoquinoline

Amides derived from 8-aminoquinoline are by far the most common bidentate DGs and have received a lot of attention. Due to the large number of examples, a selection of the most relevant ones will be presented herein, providing to the reader a nice overview of the various possible transformations. It is worth mentioning that in contrast with most of the other bidentate DGs, amides derived from 8-aminoquinoline were successfully applied in transformations of both C(sp^2^) and C(sp^3^) centers. C(sp^3^)–H functionalisations are particularly challenging transformations as the functionalisation of less reactive aliphatic chains (with or without the help of the Thorpe–Ingold effect) in an efficient manner remains an intensive quest. In this regard, the combination of using high-valent transition metals with bidentate DGs turned out to be an efficient approach in various reactions.

Note that in some cases the transition metal-catalysed functionalisation of 8-aminoquinoline itself (rather that the substrate) at the remote C5 position was observed when using these amides as DGs. These cases will not be discussed in this review.^360^–^369^ To overcome such an issue, though, a second generation of bidentate DGs was designed and the amides derived from 5-chloro-8-amino-quinoline and 5-methoxy-8-amino-quinoline were investigated (*vide infra*).

As the directing group often needs to be cleaved after the metal catalysed C–H bond functionalisation event, several strategies were developed leading to the corresponding carboxylic acid, amide or ester. Indeed, classical approaches relied on using basic or acid conditions to cleave the DG.^370^–^378^

If difficulties are met, additional steps such as *N*-methylation^379^–^382^ or *N*-Boc^383^–^387^ protection might be helpful in some cases for the deprotection of the DG under basic conditions. More recently, it has been demonstrated that oxidative cleavage^388^ (ozonolysis followed by hydrolysis) as well as Ni-389,390 and Co-catalysis^387^ were also good alternatives for instance although so far less commonly used compared to classical acid and basic conditions.

##### 14.1.1.1. C(sp^2^)–H functionalisation

###### 14.1.1.1.1. Arylation

**Scheme 65:**
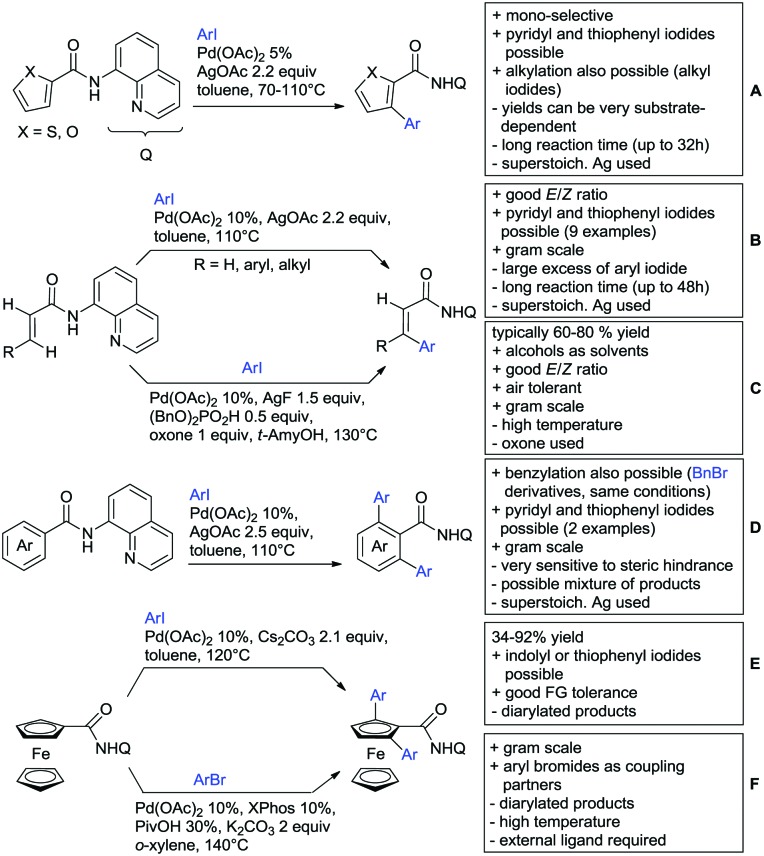
Pd-Catalysed arylation of C(sp^2^) centres with 8-aminoquinoline-derived amides as DG.

The arylation of C(sp^2^) centres has been extensively studied. In 2015, Babu reported a regiocontrolled arylation of C2-substituted thiophene or furan using aryl iodides as coupling partners.^391^ Good chemo-selectivity was observed, with tolerance of a bromide substituent for further post-functionalisations (Scheme 65A) and yields typically around 60%.

Similar catalytic systems were applied for the arylation of acrylamides towards the synthesis of *Z*-cinnamamides by Babu^372^ (Scheme 65B) and Xue/Jiang (Scheme 65C).^374^ In the former case, a large scope of carboxamides was described with moderate to good yields, up to 88%. In the last case, attempts to extend the reaction towards the alkylation of acrylamides were performed, but with only low conversions.

The Pd-catalysed arylation of arenecarboxamides was reported in 2016 by Babu.^392^ Di-arylated products were usually obtained (Scheme 65D) with moderate to good yields (25–85%), except when 5-iodoindole and 4′-iodoacetophenone were used, leading either to the exclusive formation of mono-arylated product or a mixture of compounds, respectively. Depending on the substitution patterns on the arenecarboxamides, mono-arylation or a mixture of mono- and di-arylated compounds were also observed (46–63%). The replacement of aryl iodides by a benzyl bromide derivative enabled the mono-benzylation reaction in decent yields.

The arylation reaction was extended in 2016 to *N*-(quinolin-8-yl)ferrocenecarboxamide by Kumar.^393^ This Pd-based catalytic system gave access to di-arylated and mono- or di-alkylated ferrocenecarboxamides (Scheme 65E) and demonstrated a good FG tolerance on the aryl iodide partners (alkyl, CF_3_, OMe, NMe_2_, Cl, I, acyl, NO_2_). The efficiency of Ni(OTf)_2_ was also evaluated, but lower yields were obtained.

A study performed by Yang, Wu an Wu also investigated the arylation of ferrocene derivatives (Scheme 65F).^394^ The authors evaluated the potential of 8-aminoquinoline and 2-(2-pyridyl)-2-isopropylamine (PIPNH_2_) derived carboxamide as DGs to promote the transformation. Good yields, around 70–85%, were usually obtained with the 8-aminoquinoline-derived amide.

**Scheme 66:**
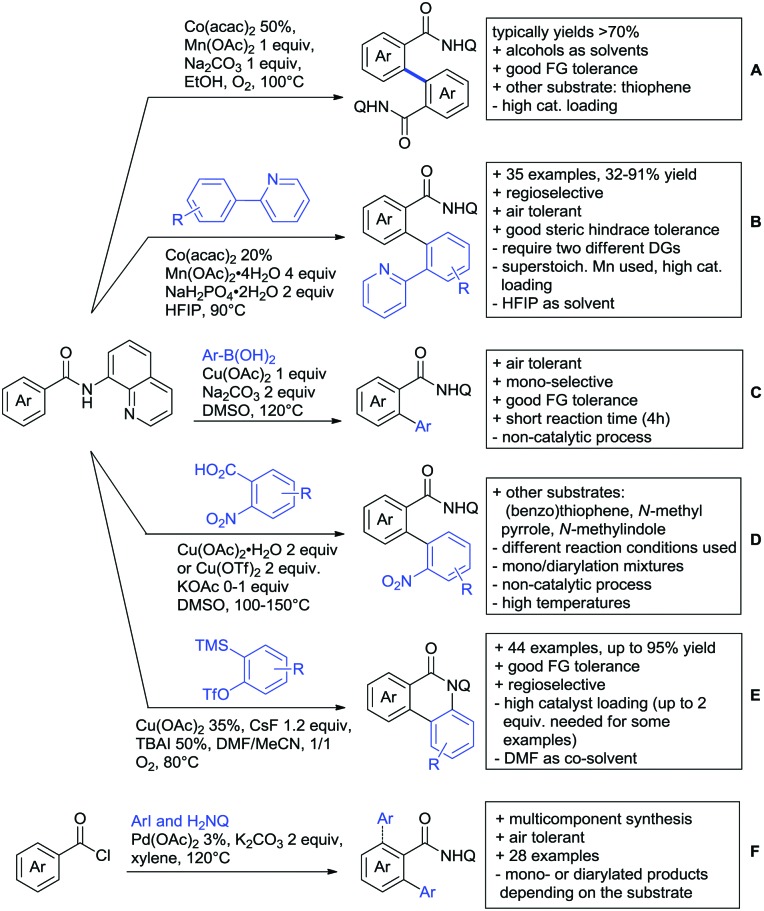
Arylation of 8-aminoquinoline-derived amides as DG.

Other metals than Pd were also investigated. For instance, mechanistic studies were undertaken by Lan on the Ru-catalysed arylation of benzamide.^395^ Using Co(acac)_2_ as catalyst, 8-aminoquinoline-derived amides were shown to dimerize.^375^ An excellent regioselectivity was observed (Scheme 66A), but attempts to achieve the hetero-coupling of two different arenecarboxamides failed to get high selectivity. This reaction tolerated several common FGs (I, Br, NO_2_, OMe, CF_3_). Another example was reported in 2016, but required a stoichiometric amount of Co salt.^396^

A mixed DG strategy adopted by Niu and Song allowed the Co-catalysed arylation of benzamides and arylpyridines, offering an access to valuable biaryl systems.^397^ This process required two parallel activations (SET and CMD pathways) for the formation of a high-valent Co intermediate coordinated to the coupling partners (Scheme 66B).

Three alternatives to the use of aryl halides were reported in the 2015–2017 period: cross coupling with arylboronic acids, a decarboxylative reaction with benzoic acids, and the use of an aryne precursor. Tan and coworkers published the arylation of arenecarboxamides with arylboronic acids mediated by Cu (Scheme 66C).^379^ A stoichiometric amount of Cu(OAc)_2_ is required, but a good selectivity as well as a good FG tolerance (F, Cl, Br, OMe, SMe, alkyl, aryl, CF_3_, CN) were observed. The corresponding products were generally obtained in moderate to good yields (48–79%). A considerable kinetic isotope effect (KIE) was observed with deuterium-labelled compounds, suggesting that C–H bond cleavage was involved in the rate-determining step of the mechanism. A similar Co-based system proved to be efficient in the same transformation and was also suitable for the arylation of acrylamide derivatives.^380^

Concerning the decarboxylative process, Hirano and Miura reported a Cu-mediated arylation of benzamides using *ortho*-nitrobenzoic acids.^398^ This reaction allowed the synthesis of biaryl compounds bearing a nitro group (Scheme 66D) with low to excellent yields (14–85%). By slightly changing the reaction conditions (addition of a base), access to phenanthridinones was possible (not shown).

The use of arynes was reported in 2017 by Zhang, with a Cu-based catalytic system.^399^*o*-(Trimethylsilyl)aryl triflates were employed as aryne precursors and the reaction offered access to phenanthridinones by means of an annulation process (Scheme 66E). The use of arynes is not common in C–H arylation processes, and this reaction is therefore a very interesting contribution to the field. A broad array of FGs was well tolerated on the amide partner (OMe, alkyl, CF_3_, Br, I, F, CN, ester). In order to avoid the extra DG installation step, Wan published in 2015 a multicomponent protocol, where the amide formation and the Pd-catalysed arylation occurred as a cascade process with moderate to excellent yields (47–95%).^400^ This step-economic approach directly employed acyl chlorides, aryl iodides and 8-aminoquinoline to obtain the 2,6-disubstituted products (Scheme 66F). The presence of a methyl group at the *ortho*- or *meta*-position of benzoyl chlorides led to the selective formation of mono-arylated products.

###### 14.1.1.1.2. Alkylation

**Scheme 67:**
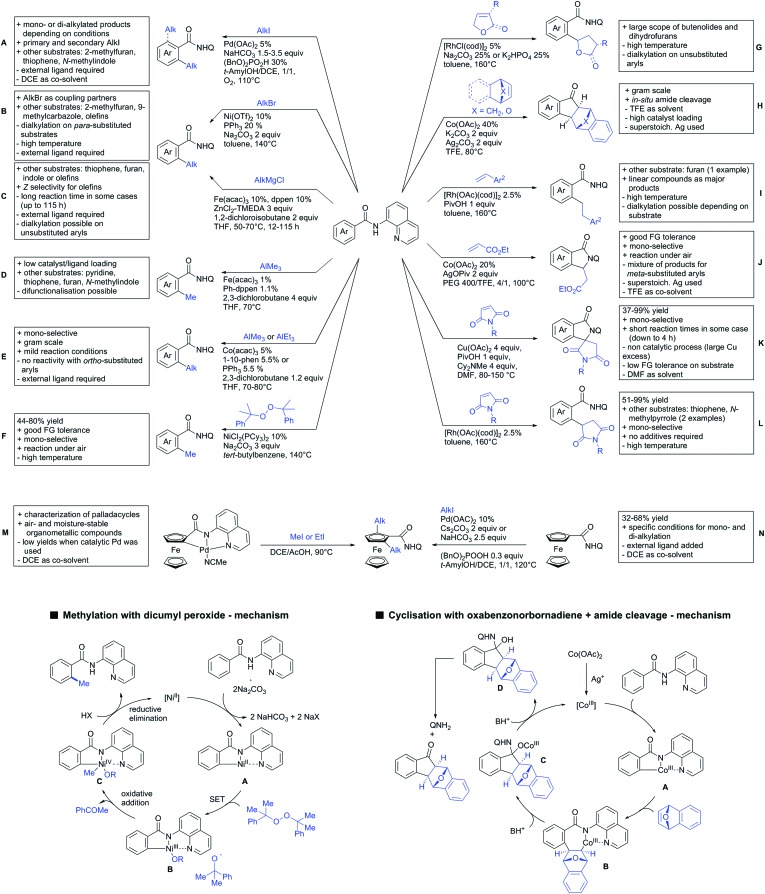
Alkylation of C(sp^2^) centres using 8-aminoquinoline-derived amides as DG.

A selective Pd-catalysed mono-alkylation of 8-aminoquinoline-functionalised benzamides was reported by Chen in 2015 (Scheme 67A).^376^ Notably, the control of the reaction time and the amount of NaHCO_3_ added regulated the selectivity of the reaction, favouring either the mono- or the di-alkylated product with good yields with primary alkyl iodides (75–87%) and moderate to good yields with secondary alkyl iodides (31–88%).

A Ni-catalysed system was also reported in the same period by Chatani.^401^ This protocol employed alkyl bromides with a wide range of substituted arenecarboxamides and acrylamide derivatives (Scheme 67B), resulting in good yields, typically >70%. An example with *para*-substituted arene carboxamide yielded the di-alkylated product. Benzylation and allylation reactions were also presented.

The Pd-based catalytic system described by Babu for arylation of thiophene and furan was also successfully applied with alkyl iodides (see Scheme 65A).^391^ An Fe-catalysed alkylation of acrylamides and arenecarboxamides using a broad range of Grignard reagents was established by Ilies and Nakamura in 2015 (Scheme 67C).^402^ A broad variety of substrates was used with excellent yields, mostly >90%. To avoid the homocoupling reaction of the alkyl partners and a possible β-H elimination, the stabilisation of an organoiron(iii) species by the DG and an additional diphosphine ligand was crucial.

Other reagents were also suitable for the directed C–H alkylation. Indeed, the same group developed in 2015 a Fe(acac)_3_ catalytic system using AlMe_3_.^403^ Efficient with both 8-aminoquinoline- and picolinamide-derived DGs, this system was applied to a large scope of (hetero)arenecarboxamides (Scheme 67D) with moderate to excellent yields (48–99%), and was proven to be also active with AlEt_3_.

The combination of organoaluminium reagents with 2,3-dichlorobutane as oxidant was reported by Xu and Zhang with a Co-based catalytic system.^404^,^405^ An excellent mono-selectivity was observed and AlMe_3_ gave yields between 73–93%. Moderate to good yields (40–87%) were also obtained using AlEt_3_ as alkyl source (Scheme 67E). Importantly, one example of C(sp^3^)–H bond methylation was reported with a moderate yield.

Another original reagent, dicumyl peroxide, was reported as a methyl precursor by Chatani in 2016 for the methylation of arenecarboxamides.^406^ This selective methylation occurred selectively on the less hindered *ortho*-position (Scheme 67F), and was totally inhibited by the addition of radical scavengers, demonstrating the radical character of the process. The following mechanism was proposed: coordination of the Ni catalyst with the bidentate DG followed by formation of the corresponding complex **A**. Then, the intermediate Ni(iii) **B** was obtained from peroxide along with an alkoxy radical *via* a SET process. After decomposition of the alkoxy radical into a methyl radical and acetophenone, the Ni(iv) species (**C**) was formed, which underwent reductive elimination/protonation steps to access the corresponding product and regenerated the Ni catalyst.

The *ortho*-alkylation of arenecarboxamides was also possible with butenolides and dihydrofurans using a Rh catalyst,^409^ generally providing mono-alkylated products (Scheme 67G) in synthetically useful yields (49–87%). Unfortunately, di-alkylation occurred with unsubstituted arenecarboxamides. In 2016, Cheng performed the reaction between the 7-oxabenzonorbornadiene and derivatives with arenecarboxamides bearing an 8-aminoquinoline pattern, with typically excellent yields (>70%, up to 93%).^410^ A [3+2] amide/alkene annulation took place, with cleavage of the 8-aminoquinoline group (Scheme 67H). A plausible mechanism was proposed: after coordination of the Co catalyst to the amide substrate, the formation of a metallacycle occurred, followed by the insertion of the bicyclic alkene, an intramolecular nucleophilic addition, a protodemetallation step and the elimination of the 8-aminoquinoline.

The Rh-catalysed directed alkylation with styrenes was reported by Chatani providing the corresponding linear products with excellent yields, mostly >80% (Scheme 67I).^411^ An electron-donating group on the arenecarboxamides accelerated the reaction, and a large panel of styrene derivatives were suitable.

The formation of isoindolin-1-ones by C–H bond alkylation/cyclisation with Co catalysis and electron-deficient alkenes was described by Ackermann in 2015.^412^ This approach demonstrated good FG tolerance and selectivity, except in the case of two *meta*-substituted arenecarboxamides (Me or F as substituents), for which a second isomer was obtained (Scheme 67J). A broad range of substituents was tested, including Cl, Br, I, F, OMe or NO_2_, and led to moderate to good yields (42–85%).

Another oxidative coupling, using maleimides as coupling partners and mediated by Cu(OAc)_2_ was published by Hirano and Miura.^413^ This method gave direct access to spirosuccinimide derivatives (Scheme 67K). A key role of Cy_2_NMe was suggested to promote the C–H bond cleavage and to increase the rate of the reaction. This process required a large excess of Cu, but a similar reaction was reported by Jeganmohan using only a catalytic amount of Co(OAc)_2_ in 2017.^414^ Maleimides were also used by Chatani, without intramolecular cyclisation, giving access to the corresponding alkylated products.^415^ This reaction was performed under Rh catalysis (Scheme 67L) and was also efficient under Ru-based conditions.

The alkylation of a C(sp^2^)–H bond of Cp rings was also examined in 2015. Methylation and ethylation of two palladacycles derived from ferrocene and a Co sandwich compound were realised (Scheme 67M) with moderate yields (31–66%).^407^ The Pd-catalysed system developed by Kumar for (hetero)arylation of ferrocenecarboxamides^393^ was equally efficient for the mono- and di-alkylation with alkyl iodides (Scheme 67N). The key to control the selectivity was the nature of the base: NaHCO_3_ promoted mono-alkylation while Cs_2_CO_3_ yielded di-alkylated products. A catalytic system using Co(acac)_2_ for alkylation of ferrocene derivatives was also studied, but only one example using an 8-aminoquinoline-derived amide as DG was reported, with 28% yield.^408^

###### 14.1.1.1.3. Allylation

**Scheme 68:**
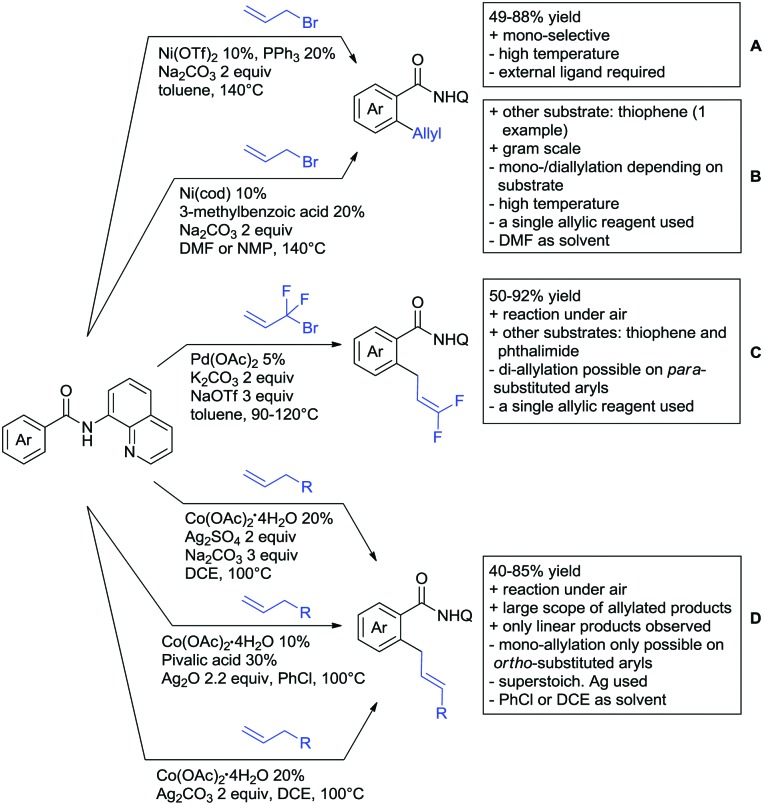
Allylation of C(sp^2^) centres using 8-aminoquinoline-derived amides as DG.

The introduction of allyl groups was also studied in recent years. In course of their studies regarding the alkylation reaction of amides, Chatani demonstrated that the same catalytic system might be used for the allylation reaction of arenecarboxamides in presence of an allyl bromide.^401^ For all examples, the presence of a substituent at the *ortho*- or *meta*-position allowed a steric control in order to give exclusively the mono-allylated products (Scheme 68A).

Sundararaju published in 2015 another Ni-based catalytic system using allyl bromide.^416^ With *ortho*- and *meta*- substitution relative to the DG, mono-allylation occurred with moderated to good yields (40–87%) while di-allylation took place only with *para*-substituted or unsubstituted arene carboxamides (Scheme 68B). A Pd catalysed system was developed by Liu using 3-bromo-3,3-difluoropropene as coupling partner (Scheme 68C).^383^ The reaction tolerated a wide range of substituents on the arenecarboxamides (OMe, Br, F, CF_3_, alkyl, aryl).

Finally, three different groups (Maiti,^417^ Jeganmohan^418^ and Chatani^419^) concomitantly reported a Co-catalysed allylation of arenecarboxamides using aliphatic olefins. In all cases, in order to avoid a mixture of mono- and di-functionalised compounds in this cross-dehydrogenative reaction, the aromatic carboxamide partners were *ortho*-substituted and only linear allylated products were observed (Scheme 68D).

###### 14.1.1.1.4. Alkenylation, alkynylation and annulation

**Scheme 69:**
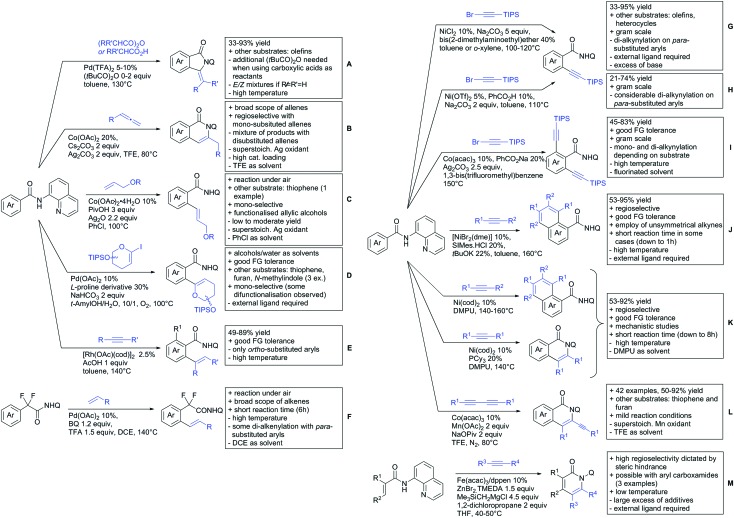
Alkenylation, alkynylation and annulation of C(sp^2^) centres using 8-aminoquinoline-derived amides as DG.

The formation of isoindolinones was reported by Wei using readily available reagents such as carboxylic acids or anhydrides as coupling partners (Scheme 69A).^420^ In this process, the presence of an additional pivalic anhydride was required for the reaction with carboxylic acids.

Another original oxidative coupling using allenes was reported by Cheng in 2017.^421^ The reaction took place specifically on the terminal carbon of mono-substituted allenes (Scheme 69B) and yields between 30 and 83% obtained. Note that when unsymmetrical or cyclic allenes were used, mixtures of products were obtained.

The Co-based catalytic system developed by Jeganmohan, mentioned in the allylation part,^418^ was also efficient for alkenylation reactions. The addition was totally selective but only low to moderate yields (23–42%) were obtained. Surprisingly the acetate group was preserved during the process, and the reaction was applied to substituted allylic alcohols (Scheme 69C). It is worth to mention that a mechanistic study regarding the Pd-catalysed regioselective C–H olefination reaction with aliphatic alkenes was reported by Peng, Paton and Maiti.^422^

A Pd-based catalytic system was used by Wu and Ye to achieve the functionalisation of arenecarboxamides with 1-iodoglycals.^423^ An additional ligand derived from l-proline was required to obtain good to high yields (51–91%) and mono-selectivity (Scheme 69D), even if traces of diglycosylated compounds were generally detected. Various substituents on the amide partner were tolerated, such as a bromine atom particularly useful for further post-modifications.

In 2017, alkynes were also used by Chatani to perform alkenylation of *ortho*-substituted arenecarboxamides with alkynes relying on a Rh catalyst (Scheme 69E).^424^ This alkenylation reaction tolerated a broad array of substituents on the aromatic ring (Me, OMe, Cl, F, Br, Ac, NO_2_, aryl).

Zhang performed a Pd-catalysed alkenylation of aryldifluoroacetic acid derivatives bearing the 8-aminoquinoline scaffold, *via* a six membered palladacycle.^425^ The reaction showed excellent selectivity with only mono-substituted compounds detected with a large scope of styrenes and allylated reagents (Scheme 69F) with moderate to excellent yields (27–99%). However, small amounts of di-alkenylated compounds were observed with *para*-substituted arenecarboxamides, especially with F, COMe and SO_2_Me as substituents.

The alkynylation of C(sp^2^)–H bond was reported multiple times using a Ni-based catalytic system. In 2015, the groups of Li and Balaraman used (triisopropylsilyl)ethynyl bromide as coupling partner for the functionalisation of benzamides.^426^,^427^ In the former case, the reaction was also applied to acrylamide derivatives, but the use of a flexible bis(2-dimethylaminoethyl)ether (BDMAE) as ligand was required (Scheme 69G). The system developed by Balaraman required additional benzoic acid (Scheme 69H). In this case, important amounts of di-alkynylated products were observed with *para*-substituted arenecarboxamides.

In 2016, Balaraman published a Co-catalysed di-alkynylation of arenecarboxamides with an 8-aminoquinoline pattern as DG (Scheme 69I).^428^ Di-Alkynylated compounds were generally obtained except in few cases, and several common FGs were tolerated (Br, Cl, F, Me, OMe, CF_3_, NMe_2_).

Chatani and co-workers published an annulation reaction after a twofold C–H bond activation (Scheme 69J).^429^ The annulation reaction could be inhibited by addition of a proton source, such as pivalic acid, resulting in alkenylation of the aromatic ring in *ortho* position, and various FGs on the amide coupling partner were tolerated (alkyl, OR, SMe, CF_3_). A similar annulation reaction with alkynes was developed by Huang using Ni(cod)_2_ as catalyst without any additive (Scheme 69K). Otherwise, the use of an electron-rich phosphine ligand favoured a [4+2] annulation yielding isoquinolones.^430^

Other metals were also studied in the last three years.

In 2017, Nicholls disclosed a Co-catalysed alkenylation of arenecarboxamides with various 1,3-diynes to form alkynylated isoquinolone derivatives (Scheme 69L).^431^ Note that the remaining alkyne was able to undergo another C–H activation process to synthesize bis-heterocycles.

Similarly, Ilies and Nakamura reported an oxidative annulation of acrylamide derivatives with alkynes catalysed by Fe, leading to the formation of pyridones (Scheme 69M) in typically excellent yields (up to 99%).^432^ When arenecarboxamides were used, the corresponding isoquinolones were obtained albeit with a narrow scope. Due to the key role of the *in situ* formed silylmethylzinc reagent, slight modifications in the reaction conditions led to the formation of mono-alkenylated products.

###### 14.1.1.1.5. Isocyanide insertion and reaction with nitroalkanes

**Scheme 70:**
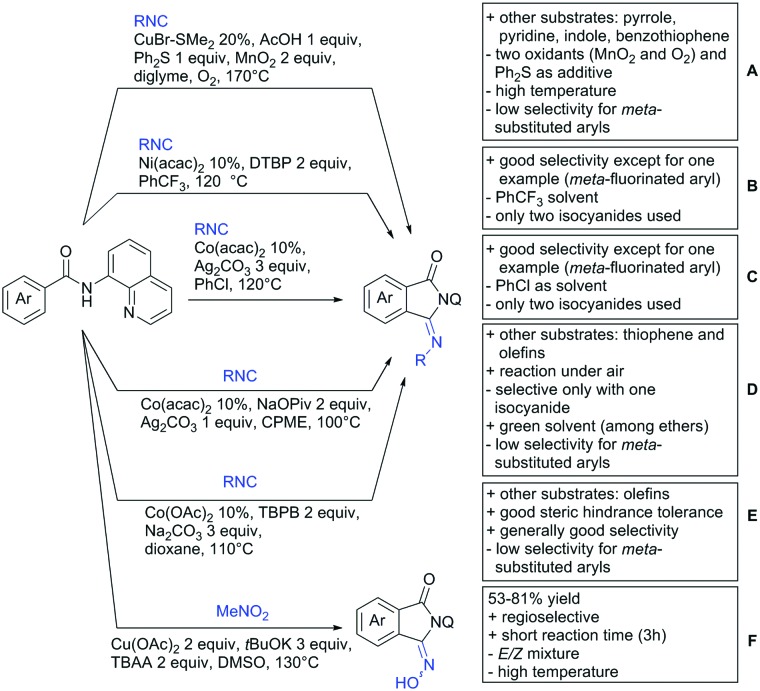
Reactions with isocyanide and nitromethane using 8-aminoquinoline-derived amides as DG.

The synthesis of 3-iminoisoindolinones was realized with various catalytic systems using amides derived from 8-aminoquinoline as DG. A Cu-catalysed formal [4+1] cycloaddition of benzamides with isonitriles was reported by Hirano and Miura in 2015 (Scheme 70A) giving moderate to good yields (21–85%).^433^ In 2016, Lei and Hao published a Ni(acac)_2_ catalysed reaction giving access to the same products (Scheme 70B) with typically good yields (>70%).^434^

In 2016 and 2017, three protocols using a Co catalyst were studied by the groups of Hao,^435^ Ji^437^ and Sundararaju.^436^ In the former case (Scheme 70C), synthetically useful yields were obtained (30–85%) and a mixture of regioisomers was only observed for the arenecarboxamide bearing a fluorine as substituent at the *meta* position.

The protocol reported by Sundararaju provided the same selectivity (Scheme 70D), good yields (35–96% yield), and a mixture of regioisomers was only observed with *meta*-trifluoromethylated arenecarboxamides.^436^ An extension to acrylamide derivatives was possible, albeit the corresponding products were obtained only with poor to moderate yields (27–63%).

The system developed by Ji showed good tolerance to steric hindrance, for instance tolerating a mesitylene group attached to the isocyanide partner (Scheme 70E).^437^ Generally, aromatic isocyanides are known to be difficult coupling partners. The transformation was efficient with arenecarboxamides (typically >70% yield) and compatible with acrylamide derivatives giving moderate yields in the latter case (45–74%).

The use of nitromethane as a coupling partner was studied by the group of Tan.^438^ This reaction gave direct access to 3-hydroxyimino-1-isoindolinones (Scheme 70F). Note that 3-methylene-1-isoindolinones were obtained when other nitroalkanes were used.

###### 14.1.1.1.6. Carbonylation

**Scheme 71:**
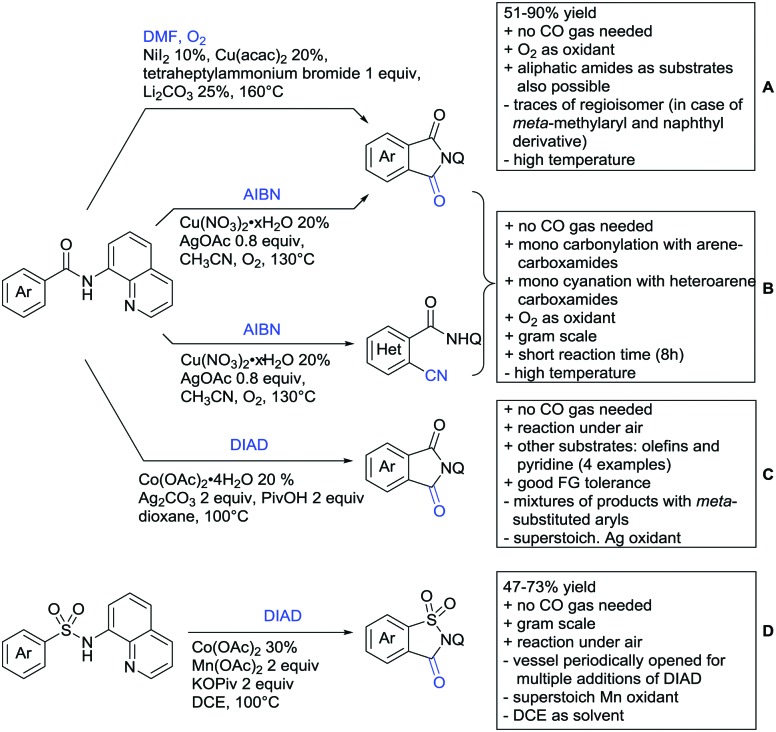
Carbonylation of C(sp^2^) centres using 8-aminoquinoline-derived amides as DG.

In the period 2015–2017, four protocols for carbonylation of C(sp^2^) centres avoiding the use of toxic CO gas were disclosed. The first, reported by Ge, employed DMF as a carbonyl surrogate and was applied to a wide range of amides, including arenecarboxamides (Scheme 71A) and aliphatic amides for the direct carbonylation of C(sp^3^) centres.^439^ A synergistic Ni/Cu system under oxygen was adopted, the Ni being coordinated to the DG and the Cu generating the electrophilic species from DMF.

A second Cu-catalysed carbonylation using 2,2′-azobisisobutyronitrile (AIBN) was reported in 2016 by Koley.^440^ With arenecarboxamides, only carbonylated compounds were observed with good yields (49–96%), but attempts to generalise this method to heteroarenecarboxamides gave only cyanated compounds (Scheme 71B), typically with excellent yields (mostly >90%).

Another carbonylation was reported in 2016 by Zhang, using diisopropyl azodicarboxylate (DIAD) as carbon monoxide surrogate.^441^ Various arenecarboxamides were used, including compounds substituted with F, Cl, Br, CN, NO_2_, CF_3_ or OMe and yields were typically around 70%. Several azodicarboxylates were also efficient for this reaction and three acrylamide derivatives were functionalised (Scheme 71C).

The last example was published by Daugulis in 2017, also using DIAD and a Co-based catalytic system, for the carbonylation of sulfonamide derivatives (Scheme 71D).^442^ This reaction gave easy access to 2-sulfobenzoic acid imides in moderate to good yields (47–73%).

###### 14.1.1.1.7. Trifluoromethylation

**Scheme 72:**
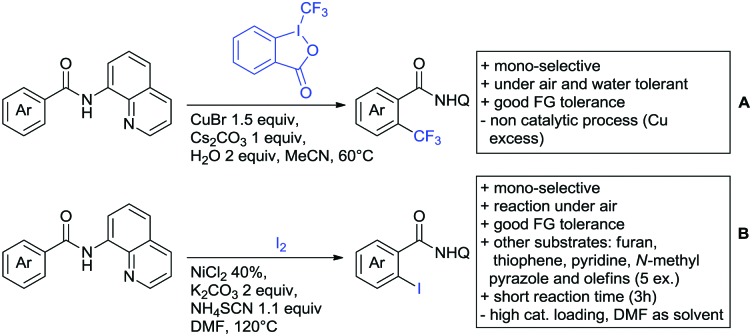
Trifluoromethylation and halogenation of C(sp^2^) centres using 8-aminoquinoline-derived amides as DG.

Tan published in 2016 a Cu-mediated protocol for the trifluoromethylation of arenecarboxamides using the Togni reagent II under mild conditions (Scheme 72A). This selective method was robust (water as additive, air tolerant), efficient (36–80% yield) and tolerant to a large scope of substituents (alkyl, F, Cl, CF_3_, OR, CO_2_R, aryl).^381^

###### 14.1.1.1.8. Halogenation

In 2016, Koley reported a Ni catalysed iodination of amides derived from 8-aminoquinoline.^443^ This process used molecular iodine and gave selectively mono-iodinated compounds (Scheme 72B) in good yield (typically >70%), with a good FG tolerance (alkyl, OR, CF_3_, Cl, Br, NO_2_, CN, aryl). Note that one non-aromatic amide was also reactive and was obtained in good yield.

###### 14.1.1.1.9. C–N bond formation

**Scheme 73:**
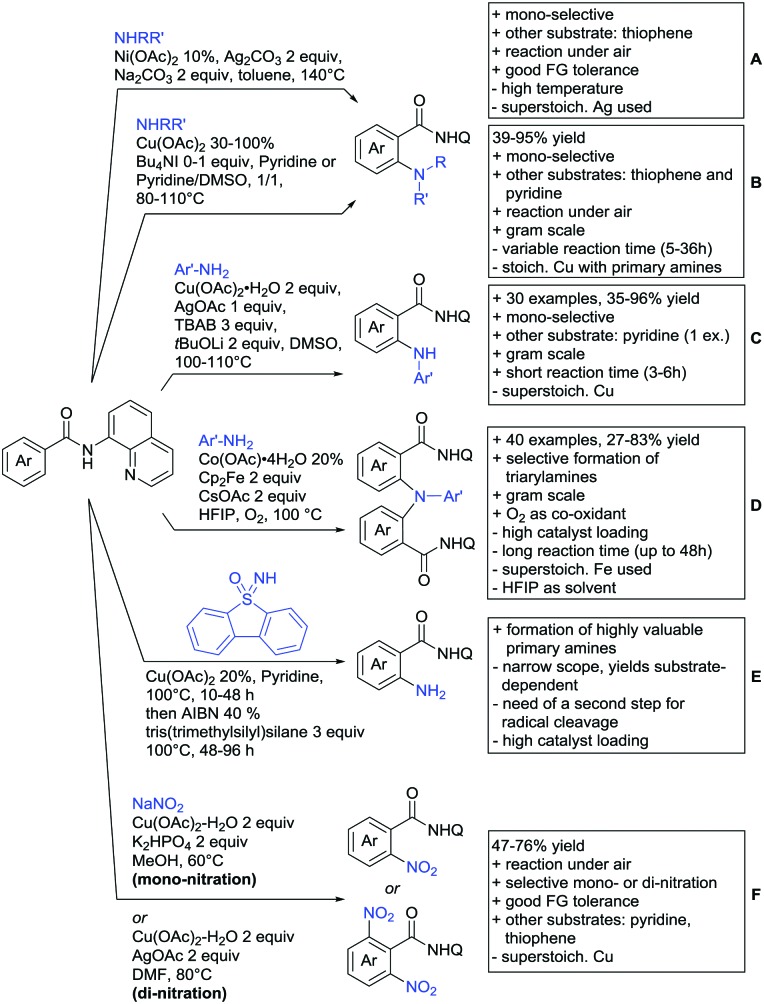
Amination and nitration of C(sp^2^) centres using 8-aminoquinoline-derived amides as DG.

Direct amination of arenecarboxamides was reported by Liu and Zhang using a Ni catalyst.^444^ Being efficient (48–81% yield) with several secondary amines (Scheme 73A), this reaction provided mono-functionalised products and mechanistic studies suggested the involvement of a Ni^I^/Ni^III^ catalytic cycle.

A Cu-catalysed protocol was reported by Daugulis in 2016.^445^ The transformation was efficient with secondary amines, but the use of 1 equivalent of Cu was required in case of primary amines and aniline derivatives leading to the corresponding products in moderate to good yields (Scheme 73B). Note that slightly modified conditions were used for the amination of electron-poorer derivatives such as sulfonamides, which was also described in this study.

Another Cu-based system was developed by Jana for the amination of arenecarboxamides, using anilines and an excess of hydrated Cu(OAc)_2_ (Scheme 73C). A broad range of substrates with different substitution patterns was functionalised and the synthetic utility of this transformation was further demonstrated by the gram scale synthesis of a bioactive compound, namely mefenamic acid.^446^ Worth to mention that an extension to heteroaryls as coupling partners was reported by Punniyamurthy also relying on a Cu-mediated transformation.^447^

The synthesis of valuable triarylamines was performed in 2017 by Niu and Song by means of a Co-catalysed C–H bond activation strategy in the presence of an excess of aniline (Scheme 73D).^448^

Studying the properties of sulfoximines, Bolm used dibenzothiophene sulfoximine as an interesting ammonia surrogate in order to access valuable primary amines.^449^ The surrogate was recycled and mono-amination was observed in all cases (Scheme 73E), but unfortunately only few examples were high yielding (20–86% yield, mostly <60%)

In addition to amination, the nitration reaction was also investigated, and *ortho*-nitration of (hetero)arenecarboxamides was performed by a Cu mediated C–H bond activation, and tolerated common FGs (aryl, alkyl, F, Cl, Br, CF_3_, CO_2_R, OMe).^450^ By changing the solvent and the base and under higher reaction temperature, the selectivity of the reaction was switched from mono- to di-nitration (Scheme 73F).

###### 14.1.1.1.10. Hydroxylation

**Scheme 74:**
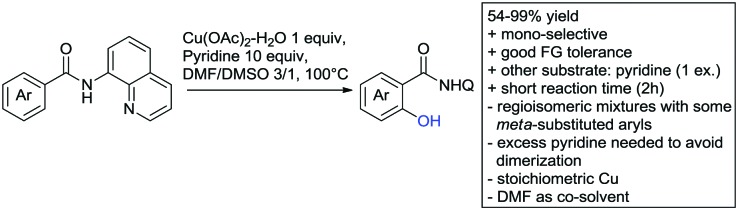
Hydroxylation of C(sp^2^) centres using 8-aminoquinoline-derived amides as DG.

A Cu-mediated *ortho*-hydroxylation of arenecarboxamides was reported by Jana and the chemoselectivity was controlled using pyridine as suggested by the author, probably by stabilizing a monomeric species and facilitating the reductive elimination (Scheme 74A). Indeed, in its absence, a dimerization process took place with some substrates.^377^ Note that, in comparison with the PIP DG, the amide derived from 8-aminoquinoline was less efficient to promote the related alkoxylation reaction.^451^

###### 14.1.1.1.11. Thiolation and trifluoromethylthiolation

**Scheme 75:**
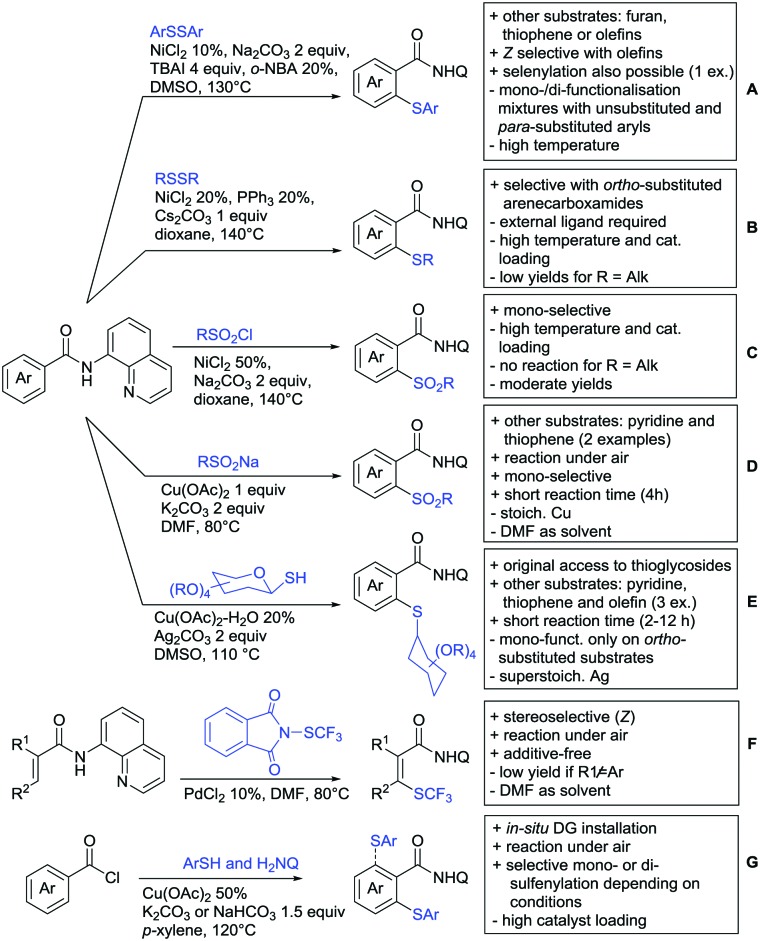
Thiolation and trifluoromethylthiolation of C(sp^2^) centres using 8-aminoquinoline-derived amides as DG.

In 2015, a direct Ni catalysed *ortho*-thiolation was reported by Zhang with diaryl disulfides.^452^ Both, arenecarboxamides and acrylamide derivatives were suitable substrates, giving desired products in good yields (mostly around 80%) and the presence of TBAI and *o*-nitrobenzoic acid as additives was required (Scheme 75A).

Kambe also studied Ni-catalysed thiolation and sulfonylation reactions with disulfides.^453^ Although the transformation was efficient (36–96% yield), a mixture of mono- and di-thiolated compounds was obtained with unsubstituted or *meta*- and *para*-substituted arenecarboxamides (Scheme 75B). Regarding the sulfonylation reaction, mono-sulfonylation of arenecarboxamides occurred (33–55% yield) along with traces of sulfonylation of the 8-aminoquinoline auxiliary with sulfonyl chloride under Ni-catalysis (Scheme 75C).

Another sulfonylation reaction was reported by Tan in 2015 using a Cu-mediated system and sodium sulfinates, prepared from sulfonyl chlorides.^454^ The reaction showed good tolerance to steric hindrance, moderate to good efficiency (46–80% yield) and a large variety of sulfinates proved to be good coupling partners (Scheme 75D).

An original reagent for C–S bond formation was used by Messaoudi in 2016, performing the thioglycosylation of arenecarboxamides.^384^ Such thioglycosides (Scheme 75E) obtained with moderate to good yields (25–88%) could mimic their *O*-glycosides equivalents, with enhanced stability. However, in case of arenecarboxamides unsubstituted at the *ortho*-position, mixtures of mono- and di-functionalised compounds were obtained.

In 2017, a diastereoselective trifluoromethylthiolation of acrylamide derivatives was reported by Bouillon and Besset, using PdCl_2_ as catalyst.^455^*Z*-SCF_3_-containing olefins were selectively synthesised in good yields (typically >70%), using the Munavalli reagent as both the SCF_3_ source and the oxidant (Scheme 75F). Mechanistic studies suggested that the C–H bond activation event was the rate-determining step.

Finally to avoid the installation of the DG, a one-pot procedure was reported in 2017 by Liu and Wei for the C–S bond formation *via* C–H bond activation.^456^ The initial conditions gave access to di-thiolated compounds, and mono-thiolated products were selectively prepared in moderate to good yields (53–81%) by changing the base and using a lower amount of thiol derivatives (Scheme 75G).

###### 14.1.1.1.12. Selenylation

**Scheme 76:**
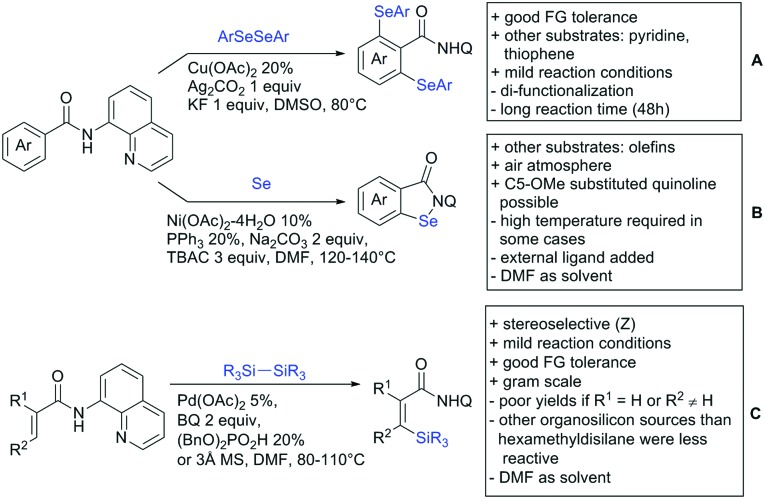
Selenylation and silylation of C(sp^2^) centres using 8-aminoquinoline-derived amides as DG.

Two protocols for C–Se bond formation by C–H activation were reported in the 2015–2017 period. The first one, published by Baidya in 2016, reported the arylselenylation of arenecarboxamides catalysed by Cu.^457^ Selective di-functionalisation was observed and a wide range of FGs was tolerated on both the arenecarboxamides or the selenated coupling partners (Scheme 76A). Substitution of the 8-aminoquinoline auxiliary was also well tolerated.

The second example was the Ni-catalysed synthesis of benzoisoselenazolone derivatives under air atmosphere, reported by Nishihara in 2017.^458^ Mechanistic studies suggested that the C–H bond cleavage is the rate-determining step of the reaction (Scheme 76B).

###### 14.1.1.1.13. Silylation

A regio- and stereo-selective silylation of acrylamide derivatives was reported by Zhang in 2017.^459^ The reaction had a good FGs tolerance (Scheme 76C) and was efficient with α-substituted acrylamides. In contrast, only moderate yields were obtained with β-substituted derivatives. A stable palladacycle was characterized and explained the observed *Z*-selectivity.

##### 14.1.1.2. C(sp^3^)–H functionalisation

The 8-aminoquinoline derived amides have been widely investigated in transition metal catalysed directed functionalisation of C(sp^3^)–H bonds and represent without any doubt a privileged bidentate DG for series of transformations.

###### 14.1.1.2.1. Arylation

Pd-Catalysed arylation of unactivated aliphatic amides at the β-position constituted the most exemplified transformations with this DG. Since 2015, different coupling partners were used such as (diacetoxyiodo)arenes, the commonly used aryl halides (mainly iodide) and more scarcely polyfluoroarenes.^460^

**Scheme 77:**
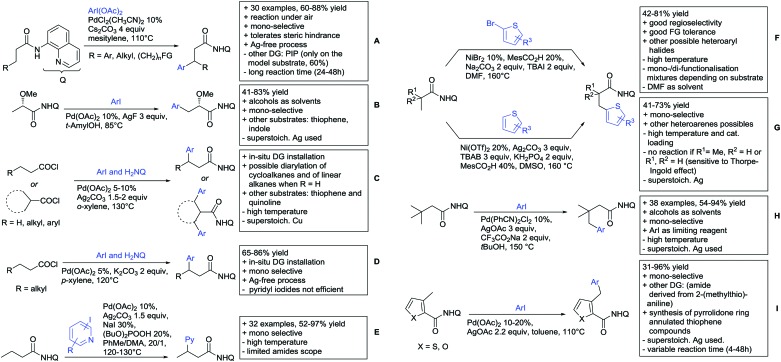
Arylation of C(sp^3^) centres with 8-aminoquinoline-derived amides as DG.

Qin reported on Pd(ii)-catalysed arylation of unactivated aliphatic amides using (diacetoxyiodo)arenes as arylation reagents *via* a Ag salt-free process, a real asset compared to other approaches (Scheme 77A).^461^ Note that in this transformation the presence of Cs_2_CO_3_ was crucial. The selective mono-functionalisation of secondary C(sp^3^)–H bonds was achieved, albeit in case of a primary C(sp^3^)–H, a mixture of mono- and di-functionalised products was obtained.

Using aryl iodides as coupling partners, the Pd(ii)-catalysed arylation of *O*-methyl lactic acids provided an efficient access to chiral β-aryl-α-methoxy acids under mild conditions (Scheme 77B).^462^ In this approach disclosed by Shi, no diarylation was observed. Note that the reaction was restricted to the functionalisation of primary C(sp^3^)–H bonds. One of the main limitations was the necessity to install the DG on the substrate. Consequently, as an improved protocol, the *in situ* generation of the bidentate DG and its use in a Pd-catalysed arylation reaction of unactivated aliphatic amides were independently studied by the groups of Babu^463^ and Liu.^464^ They developed a multicomponent reaction of acid chloride, aryl iodide and 8-aminoquinoline with a similar Pd-based catalytic system. In the former study, the monoarylation of linear aliphatic amides (22 examples, 50–97% yield) was reported. Note that in case of cycloalkanes, products resulting from a diarylation reaction (14 examples, 25–83% yield) was obtained (Scheme 77C). It is worth to mention that the Ag salt-free protocol reported by Liu was tolerant to various alkyl chains (Scheme 77D).

Moreover, the Pd-catalysed arylation of α-cyano aliphatic amides was also investigated, offering an access to useful and privileged more complex molecules.^465^ Extension of these reactions to the use of heteroaryl iodides as coupling partners was possible. Indeed, transition metal-catalysed heteroarylation of aliphatic amides were reported using iodopyridines as studied by Bach (Scheme 77E).^466^ Thiophene derivatives were also used by Xu and Qiu (Scheme 77F)^467^ as well as by Xia and Yin (Scheme 77G).^468^ In the last cases, the heteroarylation did tolerate several FGs on the heteroaromatic ring (Cl, Br, I, OR, C(O)R, CO_2_R, aryl) and was carried out under Ni catalysis.^469^,^470^

This bidentate DG was not restricted to β-functionalisation as the arylation at the γ position was also successfully achieved *via* Pd-catalysis by Maiti (Scheme 77H)^471^ and Babu (Scheme 77I),^472^ although so far restricted to specific substrates with blocked β-position.

**Scheme 78:**
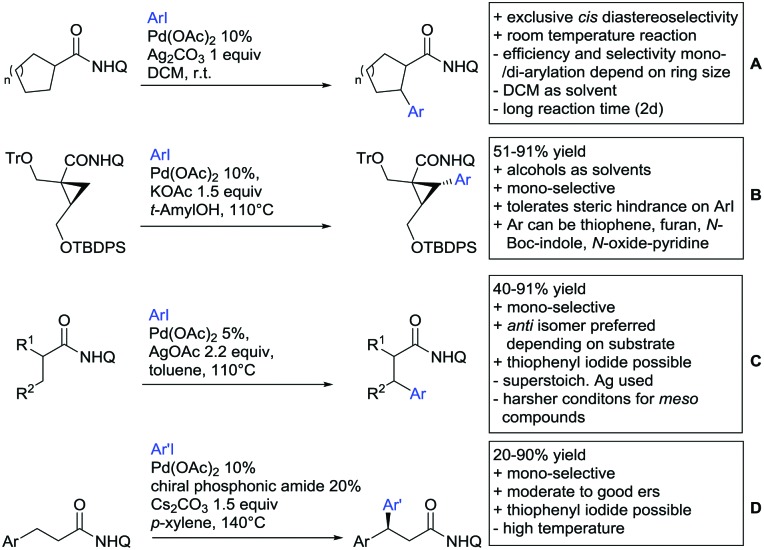
Diastereoselective or enantioselective arylation of C(sp^3^) centres with 8-aminoquinoline-derived amides as DG.

Furthermore, cyclic saturated compounds were also functionalised. Pd-Catalysed arylation of symmetric cycloalkanecarboxamides at the β-methylene position was investigated by Chen (Scheme 78A).^473^ Derivatives of various ring sizes were studied giving efficient access to the corresponding mono-arylated products with high *cis* diastereoselectivity. Note that the efficiency of the reaction was dependent on the ring size of the substrate; 5-membered rings (24–68% of mono-arylated products and 2–5% of di-arylated products), 4 membered ring (25–32% mono- and 46–64% di-), 6 membered ring (33–54% mono- and 15–17% di-) and 7 membered ring (43% mono- and 5% di-). Surprisingly, the commonly used amide derived from pivalic acid was unreactive.

Hoshiya and Shuto disclosed a new protocol for the synthesis of 1,1,2,3-type chiral tetra-substituted arylcyclopropanes by means of a diastereoselective Pd(ii)-catalysed C–H bond functionalisation (Scheme 78B).^474^ The nature of the alcohol protecting group turned out to be crucial for the efficiency of this difficult reaction by influencing steric and electronic features of the cyclopropane. Noteworthy, the tri-substituted chiral cyclopropane used as starting material was prepared in 5 steps from (*R*)-epichlorohydrin.

In addition, functionalisation of cyclobutanes was also described in a process involving a Pd(ii)-catalysed cascade reaction: (1) synthesis of cyclobutanes from 6-bromohexanamides *via* a Pd(ii)-catalysed intramolecular methylene C–H alkylation followed by a subsequent (2) intermolecular C–H bond functionalisation (*i.e*. arylation, alkenylation, alkylation, alkynylation, allylation, benzylation and alkoxylation) with various RX coupling partners offering an access to quaternary carbon centered cyclobutanes (not shown).^475^

Only rare protocols investigated diastereoselective or enantioselective arylation of aliphatic amides. In 2015, Babu reported a Pd-catalysed diastereoselective arylation of aliphatic chains of a prochiral methylene group in β-position, with different substitutions patterns such as derivatives bearing a substituent at the α- or the γ-position and α- and β-disubstituted ones as well as (*S*)-2-phenylbutanamide for instance (Scheme 78C).^371^

The enantioselective arylation of secondary C(sp^3^) centers at the β-position of aliphatic amides was studied by Duan (Scheme 78D).^476^ The presence of chiral phosphoric acids and amides enabled controlling the enantioselectivity and was beneficial to the reaction rate.

**Scheme 79:**
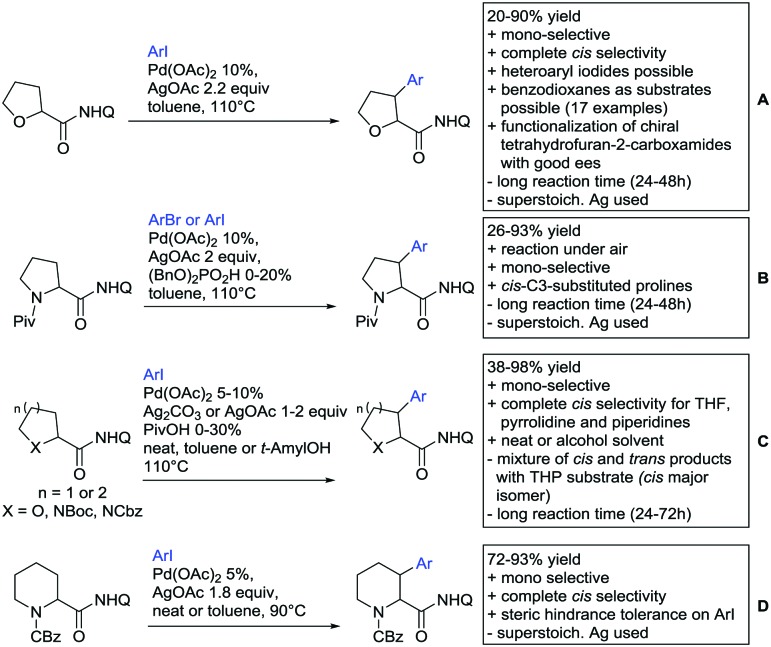
Arylation of C(sp^3^) centres of saturated heterocycles with 8-aminoquinoline-derived amides as DG.

Several groups investigated the arylation of unactivated secondary C(sp^3^)–H bonds of saturated heterocycles such as tetrahydrofuran- and 1,4-benzodioxanecarboxylic acid (Babu, Scheme 79A),^370^ proline (Liu and Zhang, Scheme 79B),^477^ furan-, *N*-protected piperidine- and THP carboxylic acid derivatives (Bull, Scheme 79C)^378^ as well as l-pipecolinic acid derivatives (Cao and Wu, Scheme 79D).^478^ In the last case, the method appeared to be robust with all excellent yields, typically around 80%, and tolerated a variety of FG such as free alcohol, nitrile and halogens. Moreover, using a similar catalytic system (Pd(OAc)_2_ (5 mol%), AgOAc (2 equiv.), toluene, 110 °C), the β-arylation of dipeptides was achieved offering the possibility to functionalise protected amino acids as well as at the C-terminus of peptides (not shown).^479^

###### 14.1.1.2.2. Alkylation

**Scheme 80:**
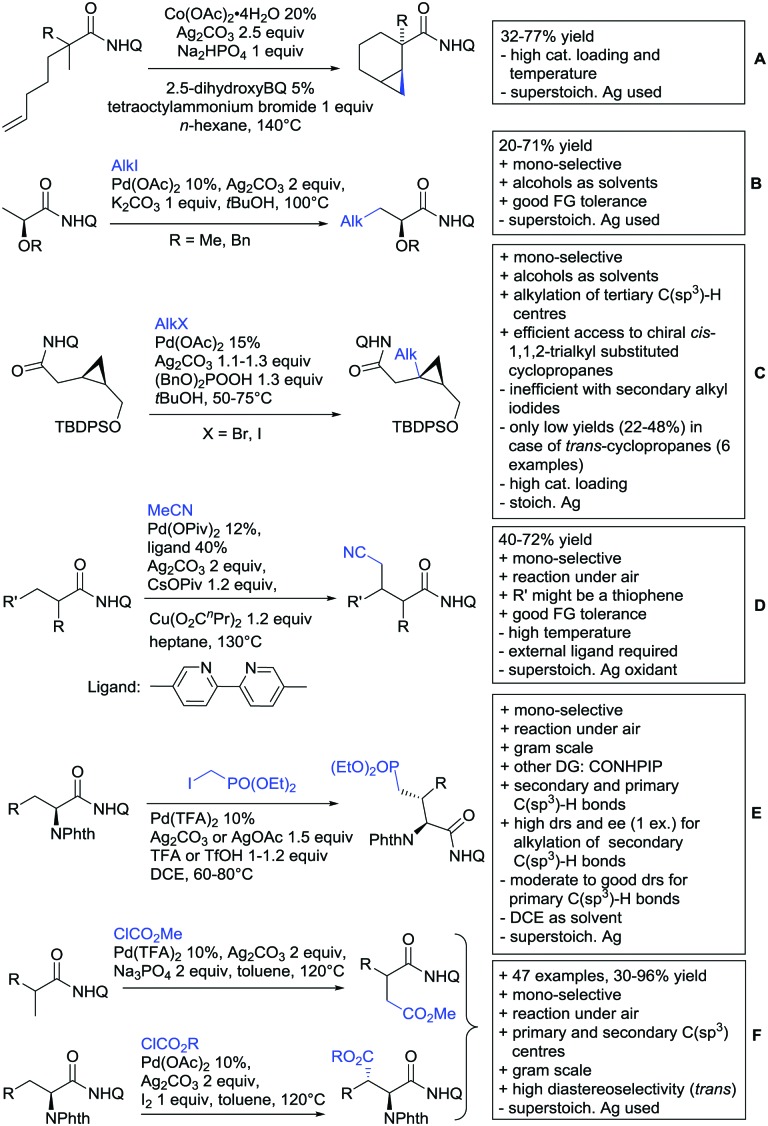
Alkylation, cyanomethylation and alkoxycarbonylation of C(sp^3^) centres with 8-aminoquinoline-derived amides as DG.

The Shi group reported a protocol based on a Co-catalysed intramolecular process leading to bicyclo[*n*.1.0]-alkanes in decent to good yields (32–77%) (Scheme 80A).^480^ The main drawbacks were a rather high catalyst loading and a relatively narrow FG tolerance (alkyl, Br, F). The same group developed an intermolecular process based on the Pd-catalysed functionalisation of *O*-alkylated lactic acid derivatives with alkyl iodides (linear or branched ones) to access chiral α-alkoxy acids in average (50–60%) yields (Scheme 80B).^481^ Only the mono-alkylated products were synthesised and the synthetic potential of this approach was demonstrated by a good tolerance to several FGs (halogene, amides, ester, protected alcohol and amine among others).

Alkylation of more rigid skeletons was possible as reported by Uenishi, who successfully developed a Pd-catalysed C–H bond functionalisation of a tertiary carbon of chiral cyclopropane derivatives with alkyl iodides and bromides (Scheme 80C) obtaining low to excellent yields (22–93%).^482^

In an interesting example, acetonitrile was used for Pd-catalysed cyanomethylation of unactivated aliphatic amides, by primary and secondary C(sp^3^)–H bond activation. The transformation displayed good β-selectivity, tolerated a broad scope of bulky aliphatic groups, and the methyl C–H bonds were preferentially functionalised in the presence of methylene C–H bonds (Scheme 80D).^385^

Phosphonoalkyl iodides were also suitable coupling partners as demonstrated by the Pd-catalysed alkylation of aliphatic chains from Yang, which offered access to valuable chiral γ-phosphono-α-amino acids using (iodomethyl)phosphonate (Scheme 80E) with yield typically around 70% and up to 97%.^483^

###### 14.1.1.2.3. Alkoxycarbonylation

Shi investigated the alkoxycarbonylation reaction of both primary and secondary C(sp^3^)–H bonds *via* a Pd(ii)/Pd(iv) catalytic cycle (Scheme 80F).^484^ The use of alkyl chloroformates as carbonyl sources constituted a real asset for this transformation. The quantity of Ag salt was important to prevent the intramolecular C–N bond reductive elimination as alternative reaction pathway, even though its exact role was not elucidated. Functionalisation at the β- and even γ-position was possible, albeit in lower yields in the last case.

###### 14.1.1.2.4. Alkenylation

**Scheme 81:**
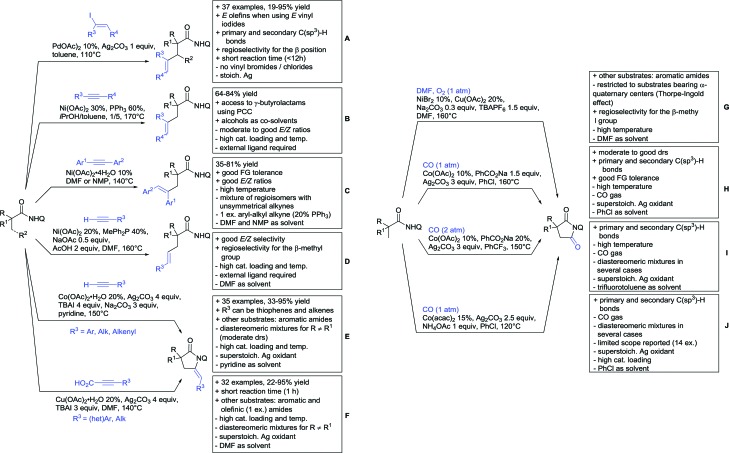
Alkenylation and carbonylation of C(sp^3^) centres with 8-aminoquinoline-derived amides as DG.

Transition metal catalysed alkenylation of aliphatic amides were investigated using different coupling partners. Rao reported a procedure based on a Pd-catalysed alkenlyation with vinyl iodides. Access to linear *E*-olefins from *E*-iodo alkenes and *Z*-isomers from *Z*-olefins was obtained, albeit with moderate *E*/*Z* ratios in the latter case (Scheme 81A).^485^

Internal alkynes turned out to be suitable coupling partners in the alkenylation of aliphatic chains under Ni-catalysis as demonstrated by the methodologies developed by You (Scheme 81B)^486^ and Maiti (Scheme 81C).^487^ Zhang used terminal alkynes (Scheme 81D).^308^ The system developed by Maiti was efficient with a broad scope of diaryl alkynes, and tolerant to common FGs (OR, CO_2_R, NO_2_, CF_3_). Alternatively, organoborate reagents were employed in an Fe-catalysed transformation, albeit restricted to few examples of alkenylated products (3 cases).^488^

A Co-catalysed reaction of aliphatic amides with terminal alkynes leading to the corresponding pyrrolidinones was reported by Zhang (Scheme 81E).^489^ Alkenylation of unactivated aliphatic C(sp^3^)–H bonds was also investigated by the same group, who developed a Cu(ii)-catalysed and Ag(i)-mediated sequential alkynylation with alkynyl carboxylic acids and annulation *via* a decarboxylative process, offering access to pyrrolidinone derivatives (Scheme 81F).^490^

Shi also demonstrated that a Ni/Cu co-catalyst system might be used for the oxidative coupling of unactivated aliphatic amides with ethynyltriisopropylsilane as the terminal alkyne (not shown).^491^

###### 14.1.1.2.5. Carbonylation

Carbonylation of aliphatic chains has attracted a lot of attention as demonstrated by recent contributions. In course of their investigations towards the carbonylation of aromatic amides by means of Ni/Cu catalysis, Ge extended this approach to the functionalisation of aliphatic amides using DMF as carbon source in an oxygen atmosphere (Scheme 81G) giving good yields in the 60–80% range.^439^ Transformations involving Co catalysts with carbon monoxide reagent were also efficient as depicted by the group of Gaunt (Scheme 81H) with moderate to good yields (49–89%) and a good FG tolerance with for instance presence of a phthalimide group.^492^ Similar systems were also reported by Sundararaju (Scheme 81I) with decent to good yields (15–87%)^493^ and by Lei (Scheme 81J) typically with yields around 70%.^494^

###### 14.1.1.2.6. Halogenation

**Scheme 82:**
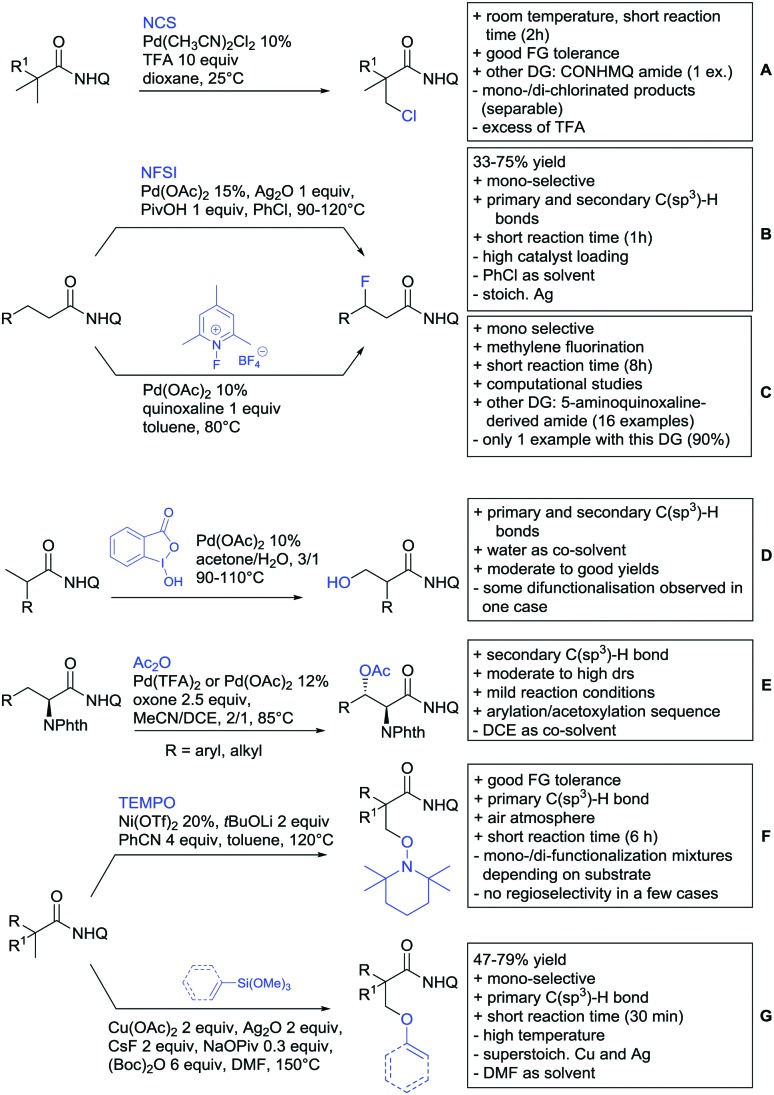
Halogenation and C–O bond formation on C(sp^3^) centres with 8-aminoquinoline-derived amides as DG.

Only one report using the amide derived from 8-aminoquinoline was depicted for a chlorination reaction between 2015–2017. Besset studied the Pd-catalysed chlorination of unactivated aliphatic chains at room temperature in presence of NCS (Scheme 82A).^495^ The transformation was regioselective and even though both mono- and di-chlorinated products were obtained, they were easily separable by column chromatography. The reaction also tolerated the presence of common FGs such as halides, and gave typically a total yield between 60 and 70%. Worth to mention is that no chlorinated product at the C5 position of the 8-aminoquinoline was detected.

In addition, to the reports from Yu^496^ and Shi^497^ who investigated the Pd-catalysed fluorination of amino acid derivatives at the β-position with other DGs, the Pd-catalysed directed fluorination of unactivated aliphatic chains with this bidentate DG was studied by the groups of Xu (Scheme 82B)^498^ as well as Wu, Zhang and Huang (Scheme 82C).^499^ In both cases, an electrophilic fluorinated reagent was used and a Pd(ii)/Pd(iv) process was proposed to promote the reductive elimination step in this challenging transformation.

###### 14.1.1.2.7. C–O bond formation

The Pd-catalysed C–O bond formation by C(sp^3^)–H activation was investigated by several groups. A protocol was proposed by Chen and Rao for the β-hydroxylation of aliphatic amides (Scheme 82D) with moderate to good yields (24–72%).^500^ In this catalytic system, water was used as the hydroxyl group source and the 1-hydroxy-1,2-benziodoxol-3-(1*H*)-one as oxidant. The reaction is moisture tolerant and in most cases, mono-hydroxylation occurred except for substrates bearing two methyl groups.

Acetoxylation reactions of methylene groups on aliphatic chains and benzylic positions were studied by Shi (Scheme 82E)^501^ under oxidative conditions (use of oxone/Ac_2_O) and gave the corresponding *anti*-β-hydroxy-α-amino acid amides in typically a yield around 60%, and up to 86%. Interestingly, these compounds were prepared in moderate to high diastereoselectivity *via* a sequential C–H functionalisation (mono-arylation or alkylation followed by an acetoxylation reaction). Note that to get a better understanding on the diastereoselectivity in Pd-catalysed alkoxylation of cyclic alkanecarboxamides, a study was reported by Rao (not shown).^502^

Stable nitroxyl radicals were used by the group of You to perform aminoxylation of C(sp^3^)–H bonds (Scheme 82F).^503^ This Ni-catalysed system gave fair to good yields (35–91%) and was tolerant to various FGs (halides, allyl, OMe, alkyl, aryl). Organosilanes were also suitable coupling partners allowing the aryloxylation/vinyloxylation of unactivated aliphatic chains as described by Zhang (Scheme 82G).^149^

###### 14.1.1.2.8. C–N bond formation

**Scheme 83:**
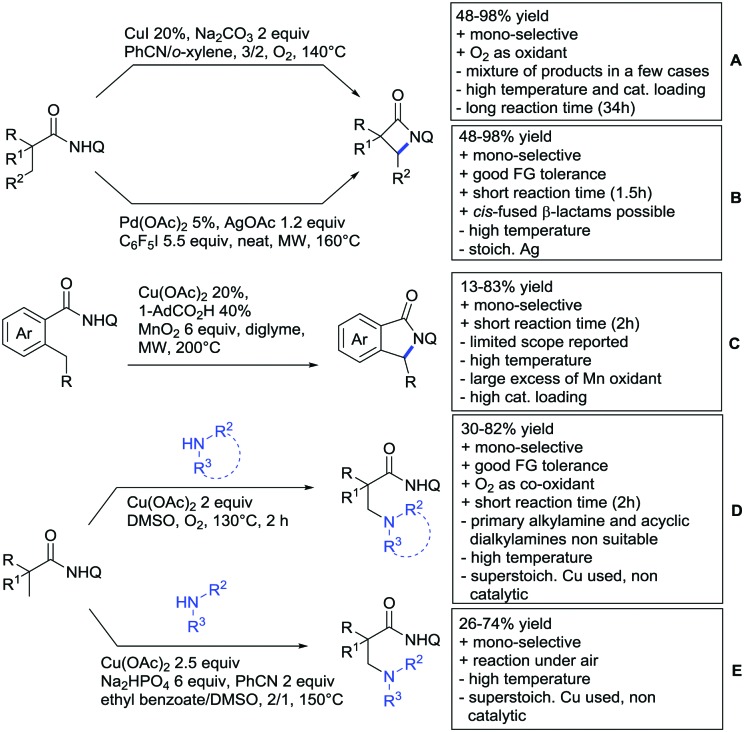
Amination of C(sp^3^) centres with 8-aminoquinoline-derived amides as DG.

The transition metal catalysed-C–N bond formation was investigated by several research groups over the 2015–2017 period. The access to β-lactams was achieved by intramolecular processes by Yang and You (Scheme 83A)^504^ and by Cao and Wu (Scheme 83B).^505^ In the first case, the use of Cu as catalyst was effective. In the second one, Pd was required. A large array of FGs was tolerated (alkyl, OR, F, Cl, Br, NO_2_, aryl) and excellent yields were typically obtained, mostly around 80–90%. Isoindolinones were also accessible thought a benzylic C(sp^3^)–H bond activation, as described by Hirano and Miura (Scheme 83C).^506^ Intermolecular processes were also disclosed, using simples alkylamines (Scheme 83D and E).^507^,^508^ Both systems are Cu-mediated, but in the first case the scope was limited to secondary cyclic alkylamines. However, a broad variety of substituent was tolerated on the amide partner (aryl or bulky aliphatic groups). In the latter case, both cyclic and acyclic dialkylamines were efficient.

###### 14.1.1.2.9. Sulfenylation, sulfonylation and trifluoromethylsulfenylation

**Scheme 84:**
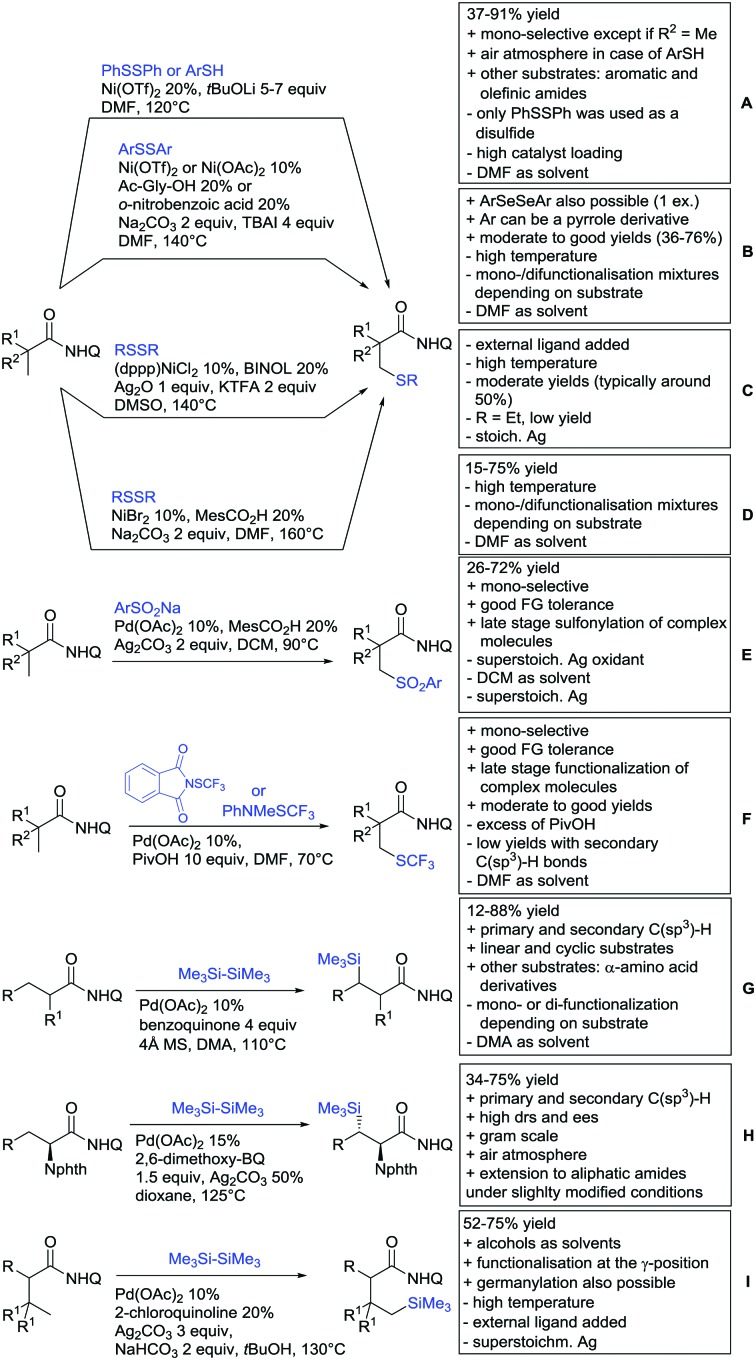
Sulfenylation, sulfonylation, trifluoromethylthiolation and silylation of C(sp^3^) centres with 8-aminoquinoline-derived amides as DG.

Since 2015 several groups (Shi,^509^ Zhang,^510^ Shi^511^ as well as Qiu, Xu and Yin^512^) investigated the Ni-catalysed sulfenylation of aliphatic chains using generally disulfides or thiol derivatives (Scheme 84A–D). These protocols were complementary using either aromatic disulfides and even thiols (Scheme 84A and B) or both aromatic and aliphatic ones (Scheme 84C and D). Note that depending on the reaction conditions and the substrate skeleton, mono- and/or di-functionalised products were obtained. This approach was not restricted to the C–S bond formation, since an extension to a C–Se bond construction was also reported (Scheme 84B).

A first example of a Pd-catalysed sulfonylation using sodium sulfinates was studied by Shi (Scheme 84E).^513^ Efficient with a large scope of sodium sulfinates, this reaction also tolerated the presence of common FGs on the amide partner (alkyl, aryl, F, Cl, CF_3_, Br). In addition, the Pd-catalysed direct introduction of a SCF_3_ group by C–H bond activation was developed by Besset, the sole report of the trifluoromethylthiolation of a primary C(sp^3^)–H bond reported so far (Scheme 84F).^373^ Decent yields were obtained (up to 53%) and this reaction was compatible with the presence of various common FGs. Besides, a large variety of aliphatic carboxamides were functionalised such as those derived from pivalic acid, α,α-dimethyl hydrocinnamic acid, phenylacetic acid and even more challenging substrates bearing a C–H bond at the α position of the carbonyl group. The synthetic utility of this transformation was further illustrated by the synthesis of analogues of bioactive molecules (ibuprofen and naproxen).

###### 14.1.1.2.10. Silylation

Silylation of unactivated aliphatic chains, α-amino acid derivatives by means of Pd-catalysis at the β-position and even at the γ-one were studied by Zhang (Scheme 84G),^514^ Shi (Scheme 84H)^515^ and Maiti (Scheme 84I),^516^ respectively. Albeit an excess of the coupling partners was generally necessary, with these three protocols functionalisation at the β- and even at the γ-position when the β-position was di-substituted was possible and extension to germanylation was even possible as depicted by Maiti.

#### 14.1.2. Amides derived from 5-chloro-8-aminoquinoline and from 5-methoxy-8-amino-quinoline

As the C5 position of the 8-aminoquinoline part of the bidentate DG might also be reactive, competitive pathways might be observed and in some reports, the selective functionalisation at the C5 position was achieved.^360^–^369^ To tackle this issue, the design of new bidentate DG was realized and the amides derived from either 5-chloro-8-aminoquinoline (Q′NH_2_) or 5-methoxy-8-amino-quinoline (MQNH_2_) were investigated instead.

##### 14.1.2.1. C(sp^2^)–H functionalisation

**Scheme 85:**
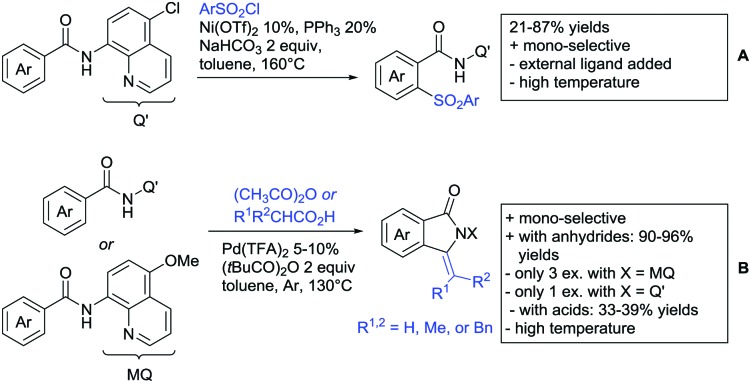
Functionalisation of C(sp^2^) centres using amides derived from 5-chloro-8-aminoquinoline (Q′NH_2_) and from 5-methoxy-8-amino-quinoline (MQNH_2_) as DGs.

The amide derived from 5-chloro-8-aminoquinoline was employed as bidentate DG in the Ni(ii)-catalysed sulfonylation of carboxamides as reported by Chatani (Scheme 85A).^517^ A mechanism involving a Ni(ii)/Ni(iv) catalytic cycle was proposed, albeit the authors cannot rule out a radical pathway. Note that the 5-methoxy-8-quinolinyl derived amides led to the decomposition of the starting materials.

In the course of their studies regarding the synthesis of isoindolinones *via* Pd catalysis, Wei showcased that the amides derived from 5-chloro-8-aminoquinoline and from 5-methoxy-8-aminoquinoline were efficient DGs in Pd-catalysed C–H bond functionalisation of benzamides with anhydrides and carboxylic acids (Scheme 85B).^420^ Note that only four examples were reported.

##### 14.1.2.2. C(sp^3^)–H functionalisation

**Scheme 86:**
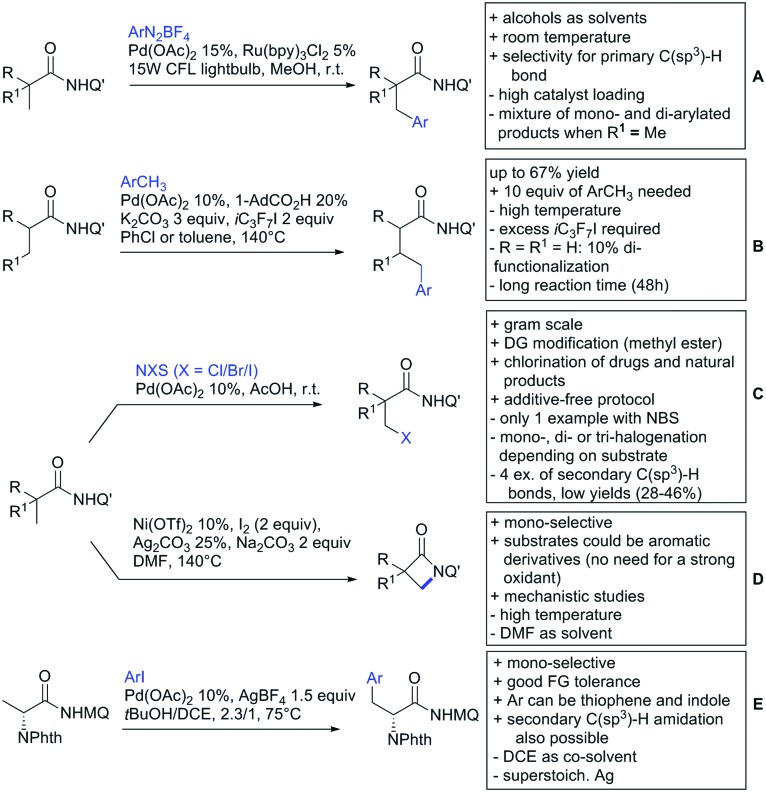
Functionalisation of C(sp^3^) centres using amides derived from 5-chloro-8-aminoquinoline (Q′NH_2_) and from 5-methoxy-8-amino-quinoline (MQNH_2_) as DGs.

It was reported that for arylation of C(sp^3^) centres, aryldiazonium salts were suitable coupling partners. Indeed, by combining Pd catalysis and photoredox, Polyzos achieved the arylation of aliphatic amides derived from Q′NH_2_ with α-quaternary centers in moderate to good yields (21–78%, Scheme 86A).^518^ The transformation was selective towards primary C(sp^3^) centres and even a benzylic methylene group remained unaffected.

The Pd(ii)-catalysed benzylation of aliphatic amides *via* a cross dehydrogenative coupling was disclosed by the group of Chatani (Scheme 86B).^519^ The transformation was selective for the β-position leading to the corresponding products in 40–75% yields and a real asset of this reaction was the use of toluene derivatives as reagent (10 equiv.) and not as a solvent. The use of this bidentate DG was not restricted to the C–C bond formation as the β-halogenation of unactivated aliphatic chains was also reported by Rao^520^ and Chatani.^521^ Rao developed a protocol enabling the halogenation of C(sp^3^) centres at room temperature *via* Pd catalysis (Scheme 86C). This bidentate DG was crucial for the success of the transformation since when the amide derived from 8-aminoquinoline was used, introduction of the chlorine atom at the C5 position was observed. Key features need to be highlighted such as (1) good FG tolerance (NO_2_, Br, F, acetyl, CF_3_), (2) the functionalisation of substrates bearing secondary, tertiary and quaternary centers at the α-position of the carbonyl groups (28–85% yields) and (3) the possibility to convert the C–X bond into various C–FG bonds.^520^ Note that depending on the skeleton of the amides, mono- or poly-halogenation occurred. Importantly, the amide derived from Q′NH_2_ was easily cleavable under acidic conditions (BF_3_·Et_2_O or concentrated H_2_SO_4_, 100 °C).^520^

Chatani showed that a Ni catalyst was suitable for the iodination reaction of aromatic and aliphatic amides derived from Q′NH_2_ with simple molecular iodine (I_2_), leading to the corresponding iodoarenes (44–83%) (not shown) or β-lactams (27–91%), respectively (Scheme 86D).^521^ When aromatic amides were used as substrates, mono- and di-iodinated products were obtained depending on the substitution patterns of the starting material (not shown), albeit in case of aliphatic chains, the corresponding iodinated products were not isolated affording a direct access to β-lactams.

Amides derived from 5-methoxy-8-aminoquinoline (MQNH_2_) were used in few reports involving Pd^505^ or Cu^504^,^506^ catalysed C–N bond formation and Pd-catalysed C–Cl bond formation.^495^ Indeed, although the methodologies were mainly developed for the amide derived from 8-aminoquinoline (QNH_2_) as DG, the good reactivity and the easy cleavage of the MQ-based DG in the presence of CAN^495^,^504^,^505^,^521^–^523^ or a demethylation/oxidation sequence^506^ towards the corresponding amides make this DG particularly attractive. Worth is to mention that only a small number of examples with MQ-based DG was depicted in these reports (1 to 3 max), hence only showcasing the potential of this DG. In addition, it was successfully applied in the C–C bond formation. Indeed, recently, Shi reported the Pd-catalysed β-arylation of alanine derivative (15 examples, 68–93% yields, Scheme 86E) and the subsequent amidation *via* an intramolecular process leading to the corresponding highly valuable α-amino-β-lactams.^522^ It is important to highlight that the cleavage of the DG was achieved for several products (6) in moderate to high yields (23–83%).

#### 14.1.3. Sulfonamides featuring 8-aminoquinoline and amides featuring phenylenediamine as DGs

**Scheme 87:**
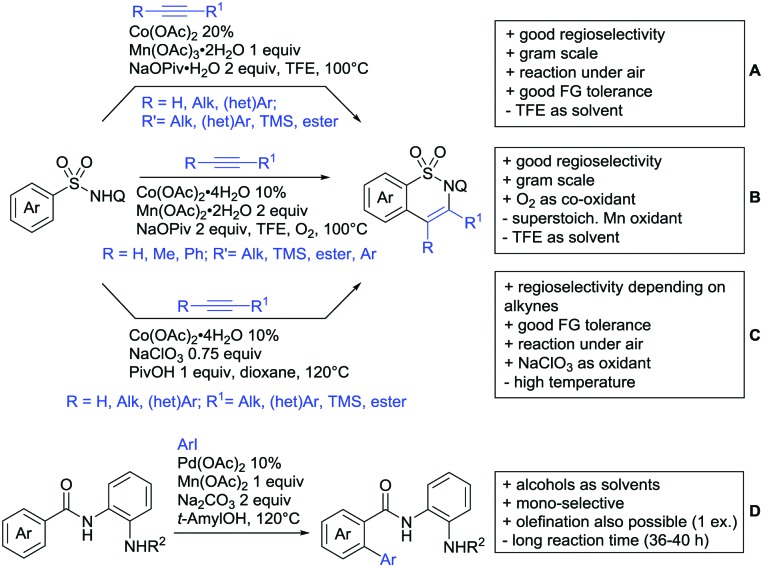
Functionalisation of C(sp^2^) centres using sulfonamides featuring 8-aminoquinoline and amides derived from phenylenediamine as DGs.

In 2015, Whiteoak, Ribas and co-workers investigated sulfonamides featuring 8-aminoquinoline as an analogue of the corresponding amide with first row transition metals (Scheme 87A).^524^ Indeed, a Co-catalysed reaction between arenesulfonamides with terminal and internal (symmetrical or not) alkynes yielding sultams was developed in the presence of Mn(OAc)_3_. This approach turned out to be highly regioselective and with good functional group tolerance. Lots of efforts were made to cleave the DG although without success.

Then, Sundararaju reported a similar study involving Co-catalysis (45–90% yield) (Scheme 87B).^525^ Finally, a slightly different catalytic process was developed by the group of Yang (Scheme 87C).^526^ In that case, no Mn salt was required as NaClO_3_ was used as an alternative oxidant and PivOH played a key role. Aromatic and aliphatic terminal alkynes were suitable coupling partners as well as internal alkynes. When unsymmetrical aryl alkyl alkynes were used a mixture of regioisomers was obtained, the aryl group being at close proximity of the nitrogen atom as major isomer.

Very recently, a new *N*,*N*-bidentate DG based on a 2-aminophenyl moiety was designed by Watkins (Scheme 87D).^527^ It was successfully employed in a Pd-catalysed arylation of benzamide derivatives (>30 examples, up to 84% yield). Mono-arylated compounds were obtained and (hetero)aromatic iodides such as benzophenone, pyrimidines, and pyridine were suitable coupling partners. With this bidentate DG, the typical use of a Ag salt was no longer necessary as it could be replaced with Mn(OAc)_2_. The DG was efficiently removed under basic conditions (NaOH/EtOH under reflux).

#### 14.1.4. Amide derived from 2-(2-pyridyl)-2-isopropylamine

The PIP based DG derived from 2-(2-pyridyl)-2-isopropylamine (PIPNH_2_) has been extensively studied since its design in 2013.^528^,^529^ This removable DG has shown great potential in direct C–H bond functionalisation with various transition metals, the most commonly used being Ni, Co, Cu and Pd catalysts. Its removal might be achieved *via* a nitrosylation/hydrolysis sequence,^451^,^530^–^535^ a two step process *via* the *in situ* esterification of an electrophilic pyridinium triflate intermediate^497^ or under acidic conditions.^536^–^538^

##### 14.1.4.1. C(sp^2^)–H functionalisation

###### 14.1.4.1.1. Arylation

**Scheme 88:**
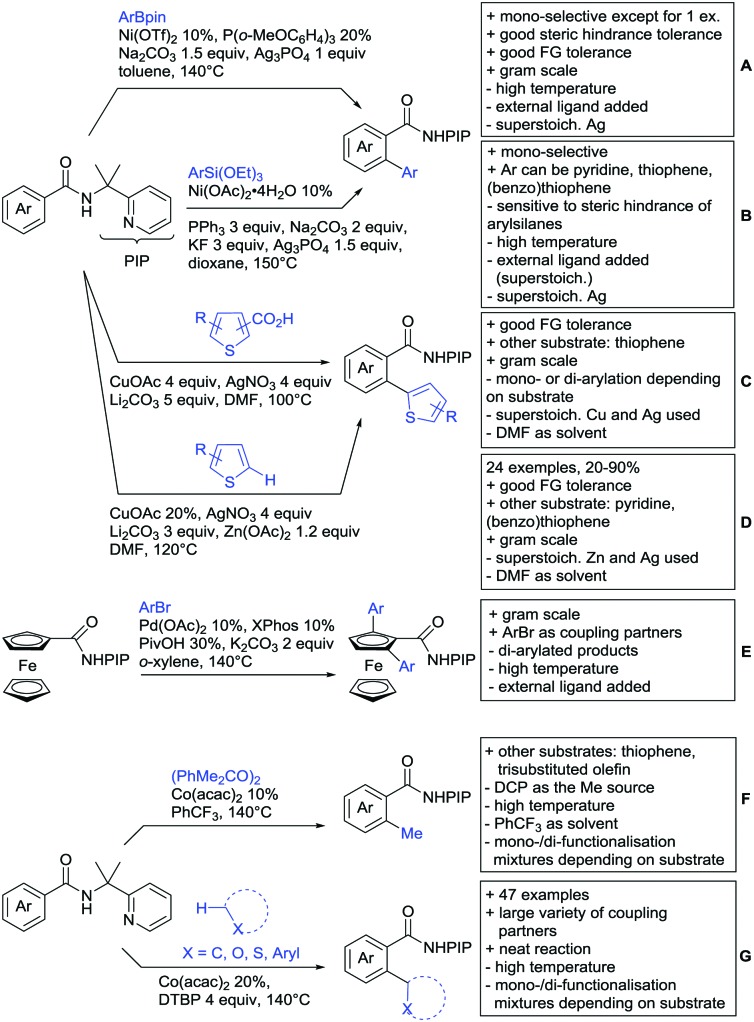
Transition metal catalysed functionalisation of C(sp^2^) centres using the amide derived from 2-(2-pyridyl)-2-isopropylamine as DG.

Since 2015, several groups were interested in arylation and heteroarylation of carboxamides. Two reports from Shi focused on Ni catalysed arylation of (hetero)arene carboxamides using aryl boron reagents^539^ or arylsilanes^530^ as alternatives to the more classically used Ar_2_I^+^X^–^ and ArX coupling partners. In the former case, a Ni(ii)/Ni(iii) catalytic pathway was proposed and the reaction proved to be efficient (40–85%), tolerant against several FGs (Cl, CF_3_, OMe, F) and steric hindrance (Scheme 88A).^539^ In the latter case, the use of non-toxic and safe to handle arylsilanes are key advantages and the transformation proceeded *via* a fluoride-promoted transmetallation in moderate to good yields (26–92% yields, Scheme 88B). The PIP based DG was removed after a nitrosylation/hydrolysis sequence affording the corresponding acid.^530^

A complementary approach relied on a Cu/Ag-mediated arylation of (hetero)arenecarboxamides using 2-thiophenecarboxylic acids as coupling partners (Scheme 88C).^531^ In this reaction, a protodecarboxylation/dehydrogenative coupling process was involved. The same authors reported that thiophenes were also suitable coupling partners. Indeed, they developed a Cu-catalysed oxidative dehydrogenative arylation of a panel of thiophene compounds and the reaction turned out to be tolerant to halogen and carbonyl groups for instance (Scheme 88D).^540^ To further demonstrate the synthetic utility of this bidentate DG, a Pd-catalysed PIP-directed arylation of C(sp^3^)–H bonds was successfully developed by Baudoin, offering an access to the aeruginosin family of marine natural products (not shown).^541^

Arylation reactions with this DG were not restricted to het(arenes) as ferrocenecarboxamides were functionalised with aryl iodides *via* Pd catalysis (30–81% yield) as developed by Yang, Wu and Wu (Scheme 88E).^394^ Note that the amide derived from 8-aminoquinoline was also a suitable DG in this transformation (see Section 14.1.1).

###### 14.1.4.1.2. Alkylation

The alkylation of aromatic carboxamides bearing PIP as DG were reported by Lu and Li (Scheme 88F and G). Indeed, they developed two Co catalysed protocols. In one case, the dicumyl peroxide (DCP) was employed playing the role of both source of the methyl group and hydrogen acceptor *via* a radical process.^542^ In the second case, an oxidative cross dehydrogenative coupling with various alkylated derivatives (alkanes, (thio)ethers and toluene derivatives) was reported (47 examples, up to 85% yield).^543^

###### 14.1.4.1.3. Alkynylation

**Scheme 89:**
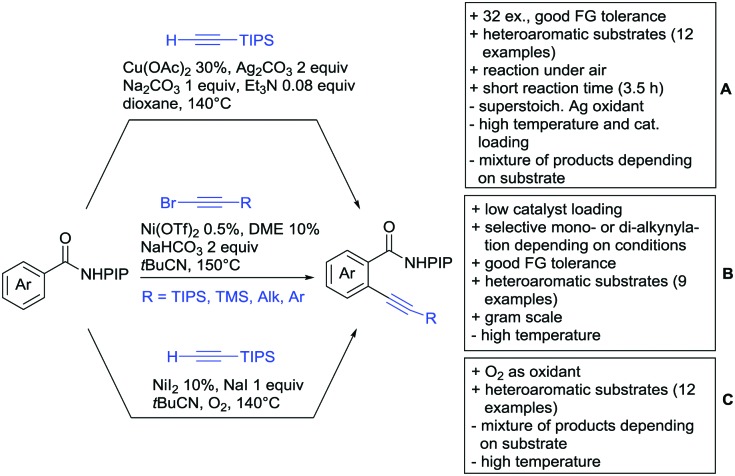
Alkynylation of C(sp^2^) centres using the amide derived from 2-(2-pyridyl)-2-isopropylamine as DG.

The group of Shi investigated the alkynylation of aromatic and heteroaromatic amides. At first, a protocol using a Cu catalyst and terminal alkynes was developed, a clear advantage compared to the existing approaches (Scheme 89A).^532^ Then, they studied a modified process involving a low Ni catalyst loading with bromoalkynes as coupling partners (Scheme 89B).^533^ The efficient reaction turned out to be chemoselective towards the synthesis of mono- or di-alkynylated products depending on the amount of alkynes used, in 62–98% yield and 52–99% yield, respectively.

Additionally, a Ni(ii)-catalysed dehydrogenative alkynylation using O_2_ as the sole oxidant was developed,^544^ a clear advantage compared to their previous report,^532^ in which a superstoichiometric amount of a Ag salt was necessary (55–95%, 28 examples, Scheme 89C). It is worth to note the key role of PIP as DG since all other bidentate DGs failed except the one derived from 8-aminoquinoline providing the expected product only in poor yield (37%).

###### 14.1.4.1.4. Halogenation

**Scheme 90:**
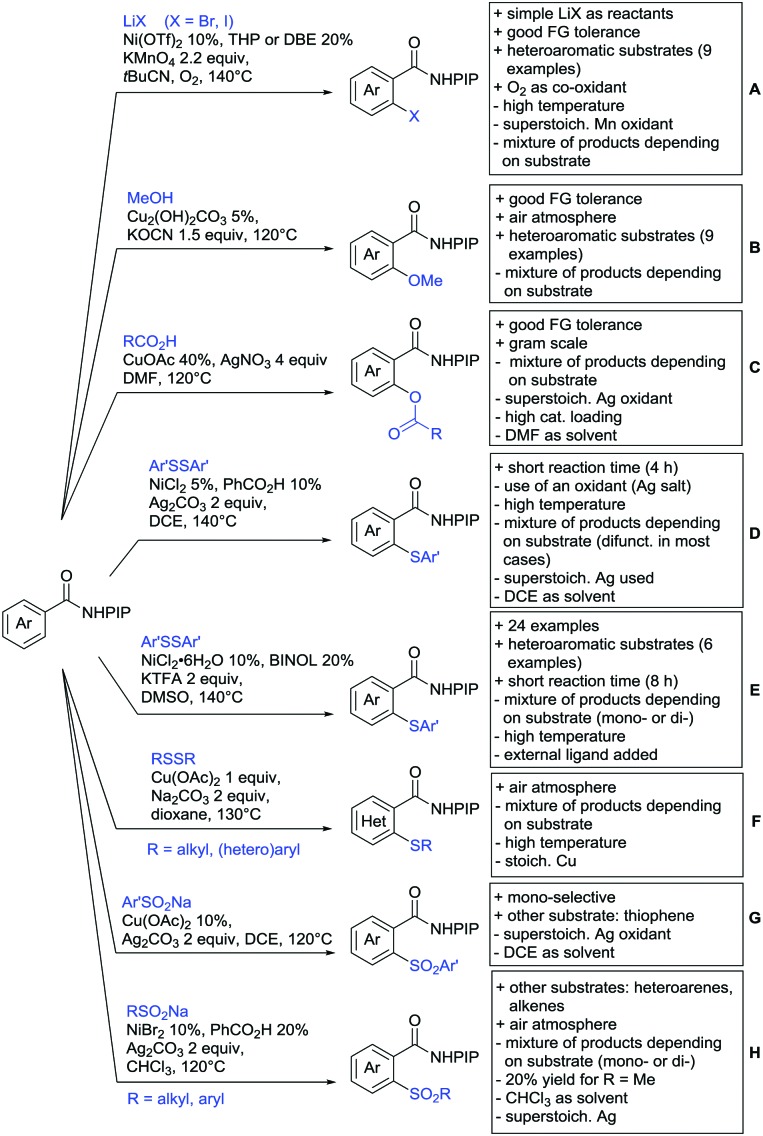
C(sp^2^)–heteroatom bond formation using the amide derived from 2-(2-pyridyl)-2-isopropylamine as DG.

Shi developed in 2016 the first Ni-catalysed halogenation of aromatic and heteroaromatic amides using inexpensive and readily available LiX salts as halide source (Scheme 90A).^536^ Note that a slight change of the catalytic system (NiCl_2_(PPh_3_)_2_ 10%, LiCl 3 equiv., KMnO_4_ 2.2 equiv., *t*BuCN, O_2_, 140 °C, 24 h), compared to the one used for the bromination and iodination reaction, allowed the formation of a C–Cl bond (not shown). Aromatic derivatives bearing various FGs (halogen, Ac, CF_3_) were functionalised in about 41–92% yield (20 examples) as well as heteroaromatic amides, albeit in somehow lower yields (30–76%, 9 examples). For unsubstituted and some *para*-substituted arenecarboxamides, mixtures of mono- and di-halogenated products were observed.

###### 14.1.4.1.5. Alkoxylation

C–O bond formation was also studied in recent years and two protocols for methoxylation (Scheme 90B)^451^ and acyloxylation (Scheme 90C)^545^*via* Cu catalysis were developed by Shi. In the first case, aromatic and heteroaromatic derivatives bearing various FGs (*e.g.* CF_3_, NO_2_, halogen) were methoxylated in moderate to good yields (25–96%). In the latter case, sterically hindered benzoic acids were crucial, as no expected product was obtained in presence of benzoic acid. The scope is pretty narrow (11 examples, 29–70%) and in several cases, mixture of products (mono- and di- as well regioisomers (one case) were obtained). The authors proposed a Cu(ii)/Cu(iii) catalytic cycle.

###### 14.1.4.1.6. Sulfenylation and sulfonylation

The PIP bidentate based DG was particularly efficient for sulfenylation and sulfonylation reactions *via* Ni catalysis.

In 2014, seminal works on transition metal catalysed C–S bond formation were developed using Pd^546^ or Rh^547^ catalysts. One year later, key contributions in that field were reported by Lu and Shi using low cost metals, especially Ni catalysts. Indeed, using aryl disulfides as electrophilic sulphur sources, Lu developed a Ni-catalysed sulfenylation reaction; the use of a catalytic amount of benzoic acid was essential to promote the reaction in typically about 80–90% yields, except for three derivatives (Scheme 90D).^537^ Afterwards, Shi disclosed another Ni-catalysed protocol which did not require an additional oxidant, although in that case an extra ligand was required (up to 93% yield, Scheme 90E).^548^ For this transformation a Ni(ii)/Ni(iv) catalytic cycle was proposed. Note that a complementary study was reported by Shi dealing with the Cu-mediated sulfenylation of heteroaromatic amides (37 examples, 83–99% yield, Scheme 90F).^549^

The sulfonylation reaction using sodium sulfinates was also studied by Shi^534^ (26 examples, up to 80% yield) as well as Gong and Song (33 examples, up to 90% yield),^550^ using a Cu- or Ni-catalyst, respectively (Scheme 90G and H).

##### 14.1.4.2. C(sp^3^)–H functionalisation

**Scheme 91:**
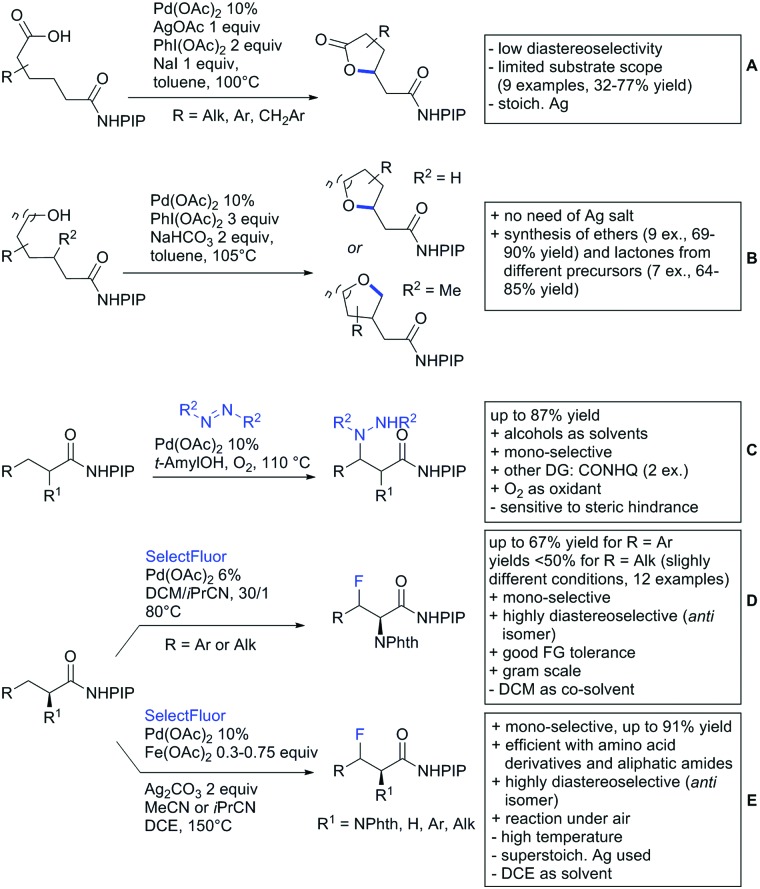
Pd-Catalysed functionalisation of C(sp^3^) centres using the amide derived from 2-(2-pyridyl)-2-isopropylamine as DG.

The PIP-based bidentate DG was also successfully employed in transition metal catalysed C–O, C–N and C–F bond formation by activation of C(sp^3^)–H bonds. Regarding C–O bond formation, this DG was mainly employed in intramolecular processes for the functionalisation of secondary C(sp^3^) centers leading to the corresponding lactones and cyclic ethers as concomitantly studied by the groups of Shi^551^ and Ye,^535^ using a similar catalytic system (Scheme 91A and B).

###### 14.1.4.2.1. Amination

Zhang studied Pd-catalysed direct amination of unactivated methylene C(sp^3^)–H bonds using azodiformates as the amino source, especially the diethyl azodicarboxylate, offering access to β-amino acid analogues (Scheme 91C).^538^ The transformation was achieved on primary and secondary C(sp^3^) centers and turned out to be sensitive to steric hindrance. Note that the amide derived from 8-aminoquinoline was also a suitable substrate even though providing the expected derivatives in lower yields.

###### 14.1.4.2.2. Fluorination

The Pd-catalysed fluorination of amino acids at the β-position was independently reported by the groups of Shi^497^ and Ge^552^ using SelectFluor as the fluorine source. Shi reported the first example of the fluorination of aliphatic and benzylic methylene C(sp^3^)–H bonds *via* an inner sphere mechanism and the PIP amide group was cleaved leading to the corresponding esters (Scheme 91D).^497^ In the latter case, the functionalisation was extended to the fluorination of aliphatic amides and good yields were obtained on unactivated aliphatic amides with various skeletons (Scheme 91E).^552^

#### 14.1.5. Amides derived from 2-(2-pyridyl)ethylamine and 2-(aminomethyl)pyridine

In contrast, only a handful of reactions used amides derived from 2-(2-pyridyl)ethylamine (PE) and 2-(aminomethyl)pyridine (PM), probably due to a less favourable geometry of the intermediate, have been reported.

**Scheme 92:**
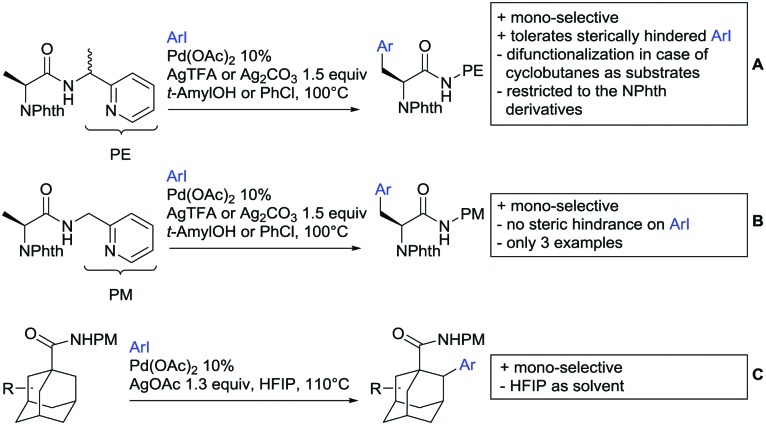
Functionalisation of C(sp^3^) centres using the amides derived from 2-(2-pyridyl)ethylamine (PE) and 2-(aminomethyl)pyridine (PM) as DGs.

In 2016, He and Chen reported a Pd-catalysed arylation of phthaloyl alanines at the β-position using amides derived from PE as bidentate DG (Scheme 92A).^553^ With two sets of reaction conditions (conditions A, 16–90%; conditions B, 15–72%), they successfully employed sterically hindered *ortho*-substituted aryl iodides (*e.g.* OMe, NO_2_, Me, OAc, COMe, F), a significant synthetic challenge. The transformation was really selective as no product resulting from di-arylation or β-lactam byproducts were observed. However, when the arylation at the β-methylene position was concerned, mono-arylated products were obtained in rather lower yields (<48%). Note that PE-directed arylation of isoleucine derivatives at the γ-position was previously reported by the authors.^554^ In comparison, when PM was used as DG (Scheme 92B), the reaction was really sensitive to steric hindrance leading to poor yields (15–26%) with 2-substituted aryl iodides but the transformation was still efficient with *para*-substituted aryl iodides (62–86% yields).^553^

The PM DG was also employed in the Pd-catalysed arylation of diamondoids with aryl iodides (36–87%), a synthetic challenge due to the large number of possible C–H bonds to be functionalised (Scheme 92C).^555^

#### 14.1.6. Picolinamides

Since the pioneering work of Daugulis,^344^ the use of this *N*,*N*-bidentate DG has been extensively investigated in various transformations.^556^–^558^ The cleavage of this DG was conducted under acidic,^559^,^560^ basic^561^–^566^ and reductive acidic^562^ conditions as well as after a treatment with PCl_5_/2,4-lutidine then MeOH and an aqueous work-up.^567^

##### 14.1.6.1. C(sp^2^)–H functionalisation

###### 14.1.6.1.1. Arylation and alkylation

**Scheme 93:**
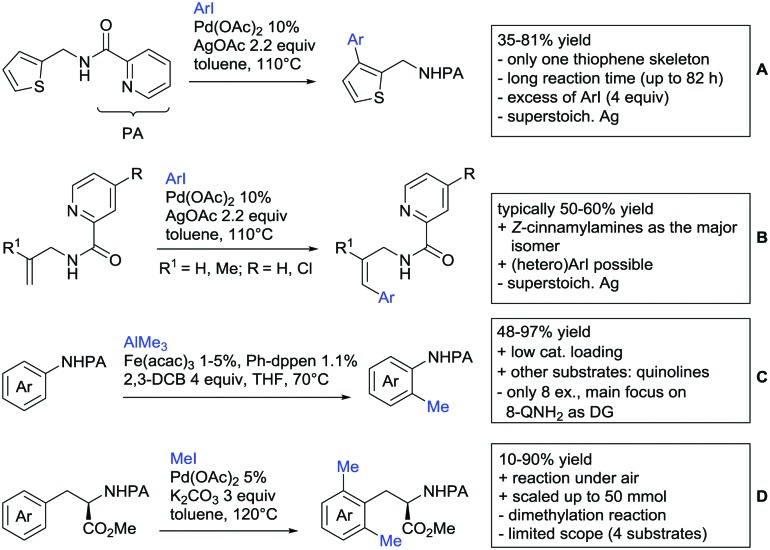
Picolinamide-directed arylation and alkylation of C(sp^2^) centres.

Pd-Catalysed arylations of thiophene derivatives and allylamines were reported. In 2016, Babu and co-workers disclosed picolinamide-directed arylation of the C3-position of 2-(aminoethyl)thiophenes with aryl iodides and heteroaryl iodides in low to good yields and in a regioselective manner (Scheme 93A).^568^ The same group used a similar catalytic system for arylation of allylamines at the γ-position leading to the corresponding cinnamylamines with good to high *E*/*Z* ratios (up to 2 : 98) in the favour of the challenging *Z* isomers (Scheme 93B).^569^ Note that substrates bearing a primary γ-C(sp^3^) center and a γ-C(sp^2^) center led to di-arylated products. Furthermore, Shao and He reported the Pd-catalysed cyclisation of alkenyl anilines *via* oxidative arylation (not shown).^570^

For the methylation of arenes, two reports were published using either AlMe_3_ or MeI as the methyl source. In 2015, Ilies and Nakamura developed a protocol allowing methylation of arenes *via* Fe catalysis, involving a plausible Fe(iii)–Me intermediate (Scheme 93C).^403^ Although efficient, only a couple of examples were reported and the selectivity for mono-methylation *vs.* di-methylation depended on the substrate substitution pattern.

Another protocol from Zhang and Ma described the Pd-catalysed methylation of the amide derived from (*S*)-tyrosine analogues (Scheme 93D).^559^ It is worth to mention that only this DG was efficient for this di-methylation reaction and a scale up on *ca.* 20 g was successfully achieved with the complete retention of the chiral information.

###### 14.1.6.1.2. Alkenylation

**Scheme 94:**
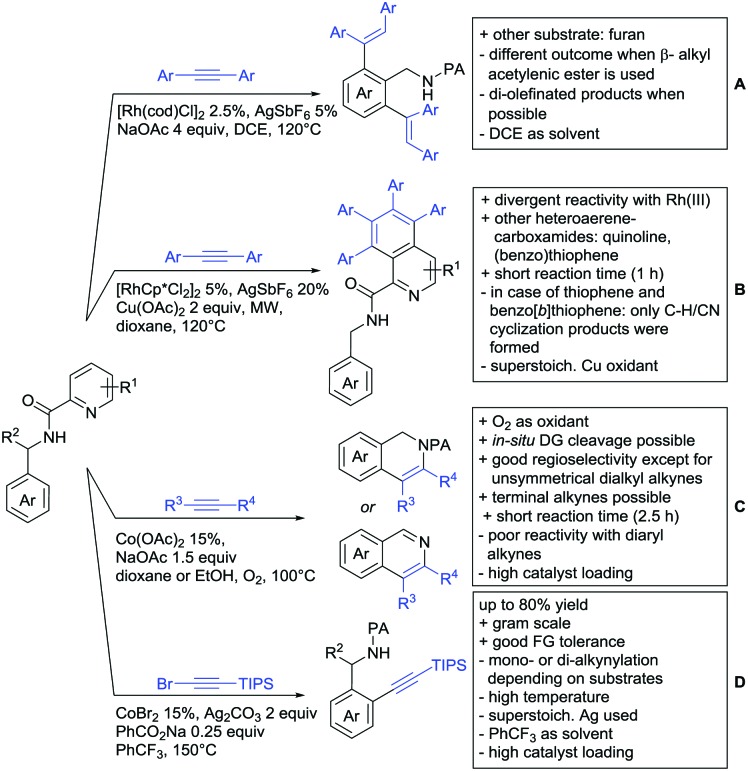
Picolinamide-directed alkenylation and alkynylation of C(sp^2^) centres.

Two examples of alkenylation/annulation reactions have appeared since 2015. Rodriguez, Arrayás and Carretero reported a Rh(i)/Rh(iii) catalysed functionalisation of aromatic picolinamide derivatives with internal alkynes (Scheme 94A and B).^561^ Depending on the choice of Rh catalyst (Rh(i) *vs.* Rh(iii)), a nice divergent site-selectivity control was achieved *via* selective functionalisation of either the aromatic or the heteroaromatic part (the DG itself) of *N*-substituted picolinamide compounds. Depending on the reaction conditions, the synthesis of isoquinoline derivatives or the olefination of benzylamine and phenethylamine derivatives was achieved. Note that in the latter case, the di-olefinated products were obtained except on specific substrates. Symmetrical and unsymmetrical alkynes were used as coupling partners; the observed regioselectivity depended on the alkyne substitution. This study was strongly supported with several mechanistic studies and DFT calculations.

Two years later, the same group reported a protocol for the synthesis of 1,4-dihydroisoquinoline compounds (56–96% yield) from benzylamines taking advantage of Co catalysis and using molecular O_2_ as oxidant (Scheme 94C).^562^ An interesting feature is that, in contrast to Rh catalysis, dialkyl alkynes were more efficient in that case as compared to diaryl ones. Additionally, terminal alkynes were suitable coupling partners as well. When unsymmetrical alkynes were used, the regioselectivity seemed to be controlled by electronic factors: 1-aryl-1-alkynes provided only one single diastereoisomer albeit no regiocontrol was observed with dialkylated alkynes.

The Co-catalysed alkynylation of benzylamine derivatives was studied by Balaraman and co-workers (Scheme 94D).^571^ The transformation was FG tolerant (NO_2_, Br, OMe) and importantly, primary, secondary, tertiary and even enantiopure benzylamines were suitable substrates. Depending on the substrate, mono-, di-alkynylated products or even a mixture was obtained.

###### 14.1.6.1.3. Cyanation

**Scheme 95:**
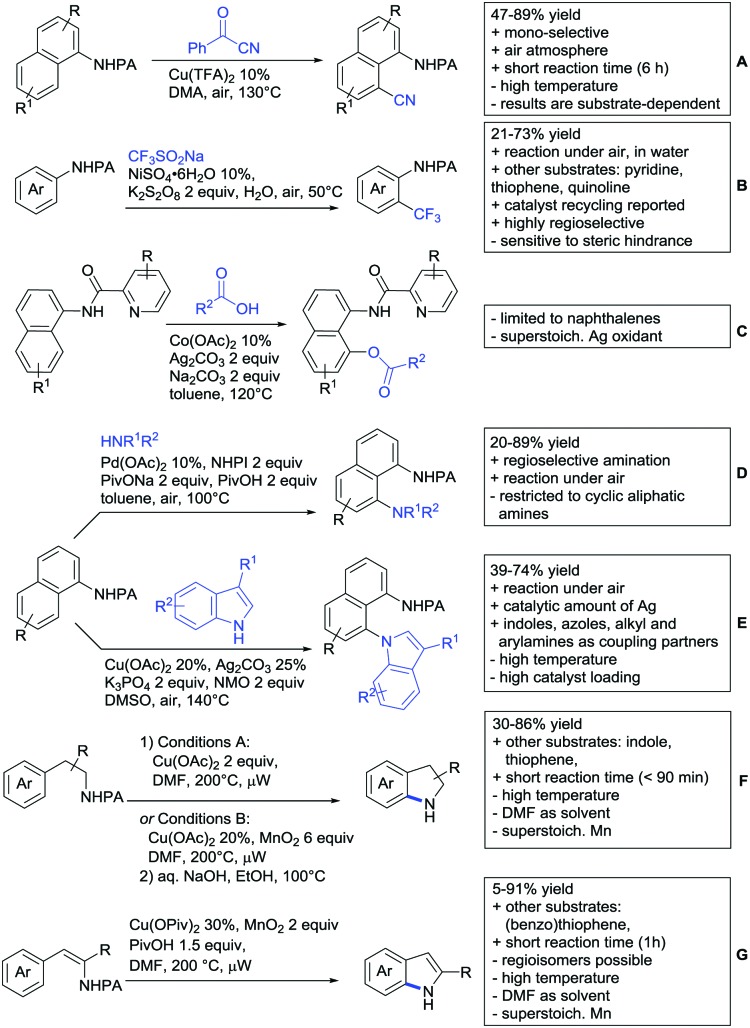
Picolinamide-directed functionalisation of C(sp^2^) centres.

Recently, the use of picolinamide as DG allowed the cyanation of naphthalene derivatives *via* Cu catalysis. Using this cost-attractive transition metal, the functionalisation of a panel of naphthalenes was possible using benzoyl cyanide as the cyano source (Scheme 95A).^560^ Although efficient, the transformation turned out to be sensitive to the substitution pattern of the substrate.

###### 14.1.6.1.4. Trifluoromethylation

Using an inexpensive Ni catalyst and the Langlois’ reagent, Zhang reported the trifluoromethylation of arylamines under mild conditions (50 °C, air atmosphere) with cheap oxidant leading to the corresponding trifluoromethylated products in moderate to good yields (Scheme 95B). It is worth mentioning that this transformation was carried out in water and the catalytic system was recycled and reused about eight times.^572^

###### 14.1.6.1.5. Acetoxylation

In 2017, Zeng reported a Co-catalysed cross dehydrogenative coupling (CDC) between picolinamide derivatives with aliphatic and aromatic carboxylic acids (Scheme 95C).^573^ It is worth mentioning that this transformation was mainly restricted to naphthalene compounds and only two *N*-benzyl substituted 2-pyridinecarboxamides were functionalised as well, although in low yields (25 and 37%). Acetoxylation at the remote ε-C(sp^2^)–H bond was studied by Babu using this bidentate DG (not shown).^574^

###### 14.1.6.1.6. Amination

The regioselective amination of 1-naphthylamine derivatives at the C8-position was independently reported by Yang, Wu and Wu^563^ as well as Punniyamurthy^564^ using a Pd or Cu catalyst, respectively (Scheme 95E and F). The Pd-catalysed mono-amination of naphthalene derivatives was achieved (up to 89% yield). Although the diversity in term of naphthalene derivatives was rather limited (4 different skeletons), several cyclic secondary aliphatic amines were suitable.^471^ Under Cu catalysis the introduction of indoles was possible in moderate to good yields (39–74%) and a large variety of FGs was tolerated on the indole skeleton (*e.g.* halogen, ester, vinyl, allyl) and even 7-azaindole.^564^ The approach was also applied to the introduction of azoles, albeit only 3 examples were reported (51–69%). Note that the arylation of the NH of the picolinamide part was also reported with boronic acids (not shown).^575^

Hirano, Miura and co-workers also investigated the Cu-promoted intramolecular amination reaction on phenethylamine derivatives. Indeed, in 2015, two sets of reaction conditions were established for the synthesis of indolines under microwave irradiation *via* a Cu-mediated or catalysed process using MnO_2_ as a terminal oxidant in the last case (Scheme 95F).^576^ In 2017, the same authors reported a DG controlled divergent process which allowed, in case of NHPA as DG, the selective access to indole derivatives starting from enamides *via* a Cu-catalysed intramolecular C–H bond amination reaction (Scheme 95G).^577^

##### 14.1.6.2. C(sp^3^)–H functionalisation

**Scheme 96:**
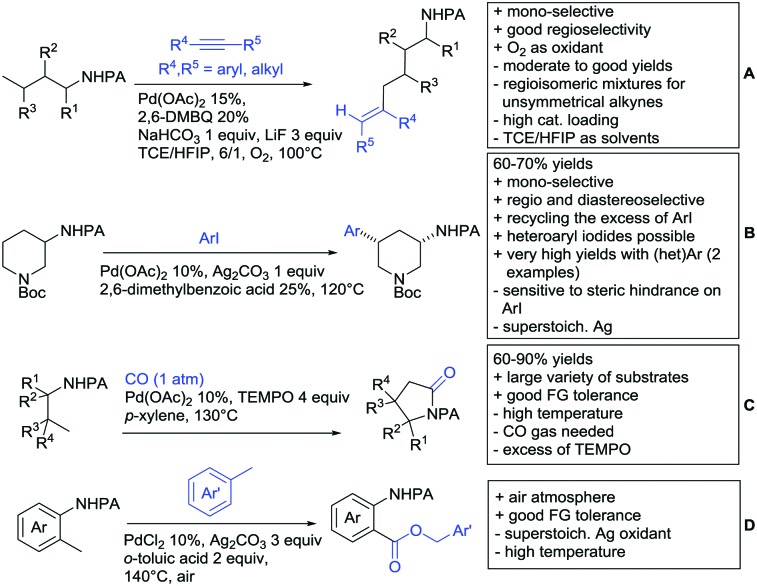
Picolinamide-directed alkenylation, arylation, carbonylation and esterification of C(sp^3^) centres.

The picolinamide DG has played an interesting role in the Pd-catalysed alkenylation of aliphatic amines at the δ-position with alkynes in a highly selective manner.^567^ Indeed, to date δ-C(sp^3^)–H bond functionalisation remains scarce and often occurred on substrates on which the γ-position was less accessible or sterically hindrance favoured a six-membered intermediate rather than the kinetically favoured five-membered metallacycle. Impressively, the functionalisation was completely site-selective even in presence of accessible γ-C(sp^3^)–H bonds. The reaction provided the corresponding products in moderate to good yields, and the role of 2,6-dimethylbenzoquinone (2,6-DMBQ) was crucial as both ligand and co-oxidant (Scheme 96A). Note that when unsymmetrical alkynes were used, mixtures of isomers were obtained, with moderate to good selectivity depending on the specific examples. Remote functionalisation was also successfully achieved by the Maes group, who developed a protocol for the remote C5-(hetero)arylation of 3-aminopiperidine *via* a Pd-catalysis (Scheme 96B).^565^ Indeed, starting from commercially available 1-Boc-3-aminopiperidine, they offered an access to *cis*-3,5-disubstituted piperidines in typically 60–70% yields (up to 82%). The mono-arylation only occurred in a regio- and stereo-selective manner. The nature of the nitrogen protecting group had a strong impact on the outcome of the reaction: a shutdown of the reactivity was observed with benzyl and carbamoyl protecting groups, while lower to good yields were obtained with pivaloyl, tosyl and alkoxycarbonyl groups. Albeit a large excess of (het)ArI was used, the unreacted reagent was easily recovery. Finally, very high yields were obtained with heteroaromatic compounds as coupling partners (two examples, 84 and 95% yields). In addition, the Pd-catalysed carbonylation of *N*-alkyl picolinamides was possible using this bidentate DG (Scheme 96C).^566^ With the methodology developed by Wang, not only γ-lactams and γ-amino acids were synthesized in high yields (60–90% yield) but this approach allowed the total synthesis of bioactive *rac*-pregabalin. Note that TEMPO was used as oxidant in the transformation.

Finally, the regioselective Pd-catalysed esterification between two different alkylbenzenes was developed by Liu and Zhang (Scheme 96D) and products were obtained in a typical 50–70%.^578^ In this transformation, the role of picolinamide as DG was crucial to ensure the esterification reaction since the tested benzamide and acetanilide were completely inefficient; the oxygen from the benzyl esters comes from the O_2_ in the air.

#### 14.1.7. Triazolyldimethyl (TAM) based amides

Since the first design of a triazole-based bidentate auxiliary by the group of Ackermann in 2014,^579^ the triazolyldimethyl (TAM) based amides proved to be an efficient bidentate DG in C–H bond activation involving Pd, Fe, Mn and Co catalysis. In addition, the DG was easily cleavable under acidic conditions^580^–^584^ or using nitrosonium tetrafluoroborate under mild conditions.^585^

##### 14.1.7.1. C(sp^2^)–H functionalisation

###### 14.1.7.1.1. Alkylation

**Scheme 97:**
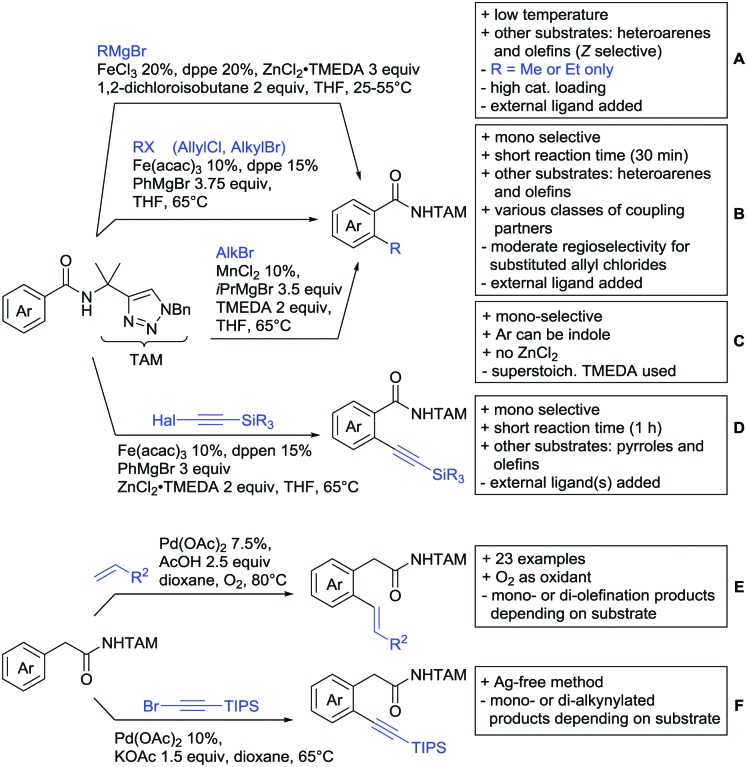
Alkylation, allylation, alkenylation and alkynylation of C(sp^2^) centres using triazolyldimethyl amide as DG.

In 2015, the combination of Fe catalysis and TAM as bidentate DG allowed the methylation and ethylation of anilides (Scheme 97A).^580^ Using cheap FeCl_3_ as catalyst in presence of ZnCl_2_·TMEDA as additive, most likely involved in the transmetallation step, the methylation of arenes (71–96%) and heteroaromatic derivatives such as pyrrole, furan and thiophene was achieved (49–60% yield). The approach was also successfully extended to the functionalisation of the challenging olefinic C–H bonds leading to the selective formation of the *Z* isomers (57 and 90% yields).

The same group further investigated the potential of the bidentate DG in Fe-catalysed C–H allylation and alkylation of amides (Scheme 97B).^585^ Using allyl chloride, the functionalisation of a broad range of substrates was possible including arenes, ferrocenes,^408^ heteroarenes and alkenes (47–92% yield). When substituted allyl chlorides were reacted, allylated benzamides were obtained with moderate to good regioselectivity (from 2 : 1 to 5.7 : 1) in favour of the branched products. Moreover, primary and secondary alkyl bromides were also suitable coupling partners. Note that such transformations were not restricted to Fe catalysis, since very recently Ackermann and co-workers reported a MnCl_2_-catalysed alkylation of amides with alkyl halides (up to 90% yield) (Scheme 97C).^582^ They developed a process involving a low-valent Mn catalyst, for which the presence of zinc additives and phosphine ligands were no longer required.

###### 14.1.7.1.2. Alkynylation and alkenylation

In 2017, Ackermann and co-workers reported the Fe-catalysed alkynylation of aromatic amides using several silylated alkynyl halides (Scheme 97D).^586^ This is an efficient process, which allowed the synthesis of mono-alkynylated products in 44–71% yields under mild reaction conditions. This reaction was also successfully applied to heteroaromatic and olefinic compounds (54–59%, 4 examples). Note that the methodology was further applied to the synthesis of the corresponding isoquinolones *via* a two-step sequence (alkynylation/desilylation–cyclisation).

A TAM-DG promoted olefination of aromatic systems using activated olefins such as acrylates, vinyl phosphonate, or vinyl sulfones was reported by Shi and co-workers (Scheme 97E).^587^ Interestingly, O_2_ was suitable as oxidant. Although efficient (typically 80–90% yield), mixture of mono- and di-olefinated compounds were observed in case of *meta*- and *para*-substituted derivatives. In 2016, Chen and Shi, published a Pd-catalysed alkynylation reaction under mild reaction conditions (Scheme 97F).^583^ This reaction was tolerant to various FGs (*e.g.* NO_2_, CF_3_, F), provided the corresponding products in high yields (76–93%) and no Ag was required, a real asset.

##### 14.1.7.2. C(sp^3^)–H functionalisation: alkylation and Alkynylation

**Scheme 98:**
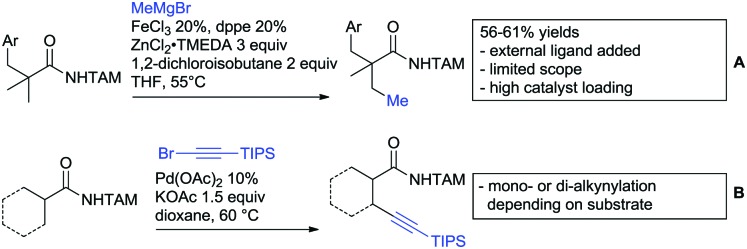
Alkylation and alkynylation of C(sp^3^) centres using triazolyldimethyl amide as DG.

In the course of their investigation of Fe-catalysed methylation of anilides,^580^ the group of Ackermann showcased the generality of their approach by an extension to the methylation of unactivated aliphatic amides (3 examples) in moderate to good yields (Scheme 98A). This bidentate DG also demonstrated its efficiency in the alkynylation of primary and secondary C(sp^3^)–H bonds (Scheme 98B). Various aliphatic derivatives (16 examples) including *N*-protected amino acids were functionalised in moderate to high yields (45–86%), moderate to good diastereoselectivity and di-functionalisation reactions were observed in some cases.^583^

#### 14.1.8. Other bidentate DGs based on heterocycles

Other *N*,*N*-bidentate DGs based on heterocycles were reported including those combining an amide part and a heterocyclic unit such as an oxazoline, pyrazole, benzothiadiazole and alkylbenzimidazole. Those DGs were mainly used in the formation of C–O and C–S bonds either on aromatic or aliphatic derivatives.

##### 14.1.8.1. Amide-oxazoline

###### 14.1.8.1.1. Alkylation

**Scheme 99:**
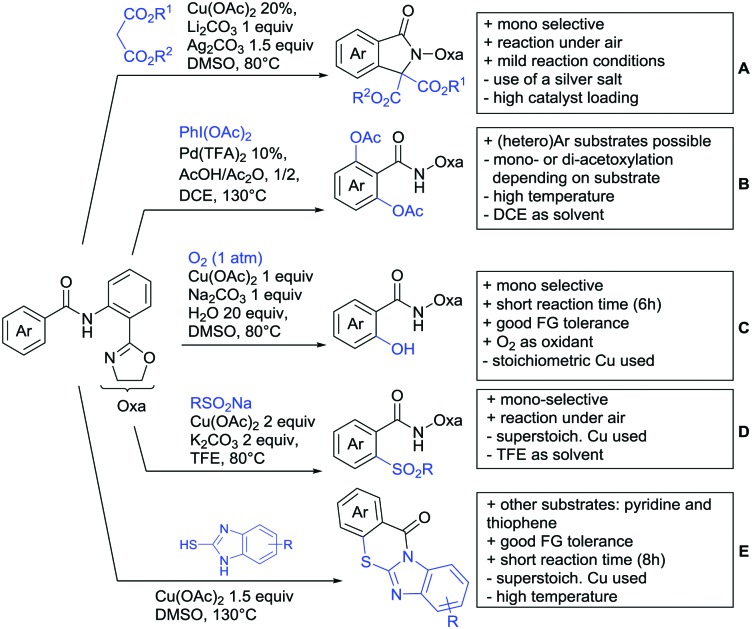
Functionalisation of C(sp^2^) centres using 2-(2-oxazolinyl)phenyl amide as DG.

Using the 2-(2-oxazolinyl)phenyl moiety on the amide nitrogen, the group of Yu developed a Cu-catalysed oxidative coupling of aromatic amides with malonate derivatives, offering a straightforward access to isoindolinone compounds in 40–75% yields (Scheme 99A).^588^

###### 14.1.8.1.2. Acetoxylation

In 2015, Ge and Li reported the Pd-catalysed acetoxylation of amide-tethered oxazoline. Indeed, *via* a six-membered bidentate complex with Pd(ii), the di-acetoxylation of various aromatic and heteroaromatic amides was achieved in the presence of PhI(OAc)_2_ playing the role of both oxidant and OAc source in 32–73% yields (Scheme 99B).^589^ It turned out that depending on the substitution pattern of the substrate, mono-acetoxylation was achieved. Note that using very simple conditions, Yu also developed Cu-mediated hydroxylation of aromatic amides (Scheme 99C).^590^ The transformation was tolerant to various FGs (alkyl, CF_3_, F, I, Cl, vinyl moiety) and provided the products in 37–86% yields.

###### 14.1.8.1.3. Sulfenylation

The formation of a C–S bond by making us of *N*-[2-(2-oxazolinyl)phenyl] amides was also studied by Manolikakes (Scheme 99D)^591^ as well as Liu and Chen (Scheme 99E)^592^*via* Cu mediated transformations using either sodium sulfinates (22–82% yield) or 2-mercaptobenzimidazoles as coupling partners (up to 84%). In both transformations, a good FG tolerance was noted (*e.g.* Cl, CF_3_, acetyl).

#### 14.1.9. Other *N*,*N*-bidentate DGs

**Scheme 100:**
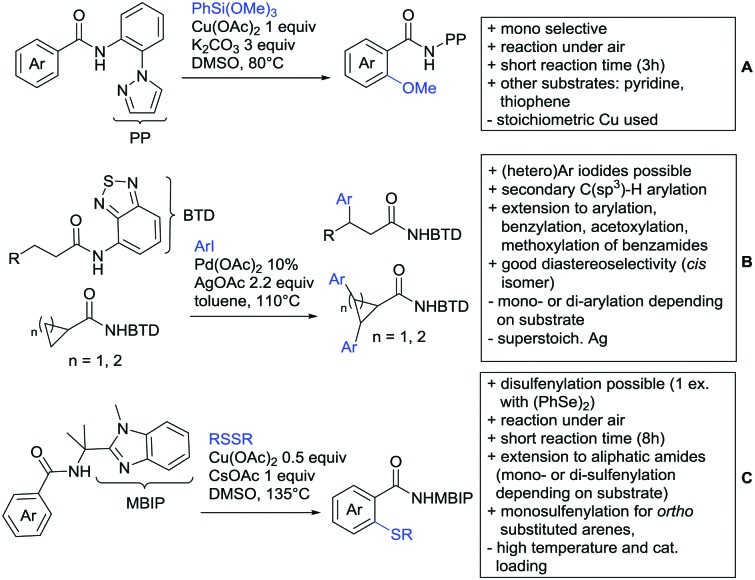
Functionalisation using amides derived from other heterocyclic amines as bidentate DGs.

The amide derived from (2-aminophenyl)pyrazole (PPNH_2_) was employed for the Cu-mediated etherification of (hetero)arenes and alkene derivatives using PhSi(OR)_3_, Si(OR)_4_ or ROH/Si_2_Me_6_ as coupling partners leading to the corresponding products with yields up to 87% (Scheme 100A). The DG was easily removed under Lewis acid mediated methanolysis (BF_3_·Et_2_O/MeOH, 100 °C) leading to the corresponding ester.^593^

Amide derived from 4-amino-2,1,3-benzothiadiazole (BTDNH_2_) was studied by Babu in Pd-catalysed arylation and acetoxylation (2 examples) of linear aliphatic carboxamides in yields 52–97% yields (Scheme 100B).^594^ In case of cyclic carboxamides, di-functionalisation generally occurred although in some cases, mixtures of mono- and di-functionalised products were obtained. Its cleavage was achieved under basic conditions (NaOH, ethanol, 85 °C) to access the corresponding carboxylic acid.

Furthermore, the amide derived from [2-(1-alkylbenzimidazolyl)]-2-isopropylamine offered a good alternative, in Cu-catalysed sulfenylation of aromatic and olefinic amides (47 examples, Scheme 100C).^595^ Note that the Pd-catalysed arylation of thiophene derivatives using pyrazine-2-carboxamide as DG was also reported (not shown). A two steps sequence (*N*-Boc) protection/hydrazinolysis (NH_4_I, NH_2_NH_2_·H_2_O) under microwave irradiation afforded the Boc-protected MBIPNH_2_ and the corresponding *N*-acylhydrazide in good yield.^568^

### 14.2. *N*,*S*-Bidentate DGs

An interesting alternative to *N*,*N*-bidentate DGs relies on the use of *N*,*S*-bidentate DGs, easily cleavable under acidic (HCl, MeOH, 100 °C)^596^ or basic^597^,^598^ (KOH, 80–85 °C) conditions. Although restricted to a handful of examples, these *N*,*S*-bidentate DGs were successfully applied in the arylation of alkanecarboxamides, especially on challenging cyclopropane derivatives.

**Scheme 101:**
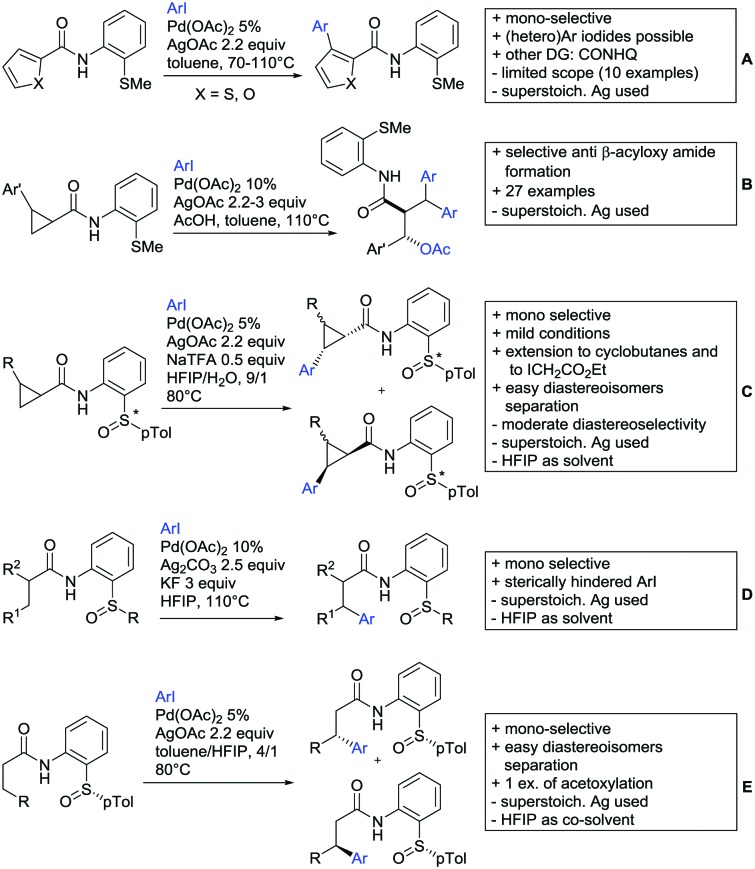
Arylation using *N*,*S*-bidentate DGs.

In 2015, Babu studied the arylation of thiophene- and furan-2-carboxamides by directed C–H bond activation using either the amide derived from 8-aminoquinoline or the 2-(methylthio)aniline.^391^ Using the bidentate *N*,*S*-DG, a selective Pd-catalysed C–C bond formation of thiophen- and furan-2-carboxamides at the C3 position was achieved using (hetero)aryl iodides as coupling partners in 25–81% yields (Scheme 101A). Then, the same group investigated the use of this *N*,*S*-DG in the functionalisation of C(sp^3^)–H bonds. Hence, they described the Pd-catalysed arylation of cyclopropanecarboxamides followed by ring opening of the cyclopropanes using the amide derived from 2-(methylthio)aniline as DG (not shown).^472^ Interestingly, di- or tri-arylation and the formation of a C–O bond was reported within a one pot process, leading to anti β-acyloxy amides in 10–86% yields, although an excess of Ag salt was necessary (Scheme 101B).^599^

Then, Wencel-Delord and Colobert employed amides derived from enantiopure sulfinyl anilines in the diastereoselective arylation of cycloalkanes (cyclopropanes and cyclobutanes) leading to the corresponding compounds in moderate diastereoselectivities (60/40 to 80/20), in 26–92% total yield of both diastereoisomers. It is worth to mention that both diastereomers were easily separated by column chromatography offering access to enantiopure compounds after cleavage of the DG (Scheme 101C).^600^

In 2017, Chen and He published a Pd-catalysed β-methyl and even more challenging β-methylene C–H arylation with sterically hindered aryl iodides by playing with the nature of the *ortho*-sulfinyl aniline auxiliaries (R^3^ = Me, C_6_H_4_Me) (Scheme 101D).^601^ The reaction showed a good FG tolerance (NO_2_, F, OMe, CO_2_Me) for *ortho* substituted aryl iodides and about 60–70% as typical yields. The same year, a diastereoselective transformation was developed by Wencel-Delord and Colobert (Scheme 101E), offering an access to arylated aliphatic amides at the β-methylene position (up to 9/1 dr), which was extended to one example of acetoxylation (91% yield, 7/3 dr).^598^

Miura and Shishido reported Ru-catalysed alkenylation of *N*-tosyl aromatic amides with internal alkynes followed by a cyclisation process affording the corresponding isoindolinones in 27–71% yields (not shown).^602^

### 14.3. *N*,*O*-Bidentate DGs

In this section the recent progresses involving *N*,*O*-bidentate DGs will be discussed. Indeed, a couple of them were investigated such as the bidentate amide derived from 2-pyridinamine-*N*-oxide, 1-aminoanthraquinone and the oxalyl amides.

#### 14.3.1. Amide derived from 2-aminopyridine-*N*-oxide

The bidentate amide derived from 2-aminopyridine-*N*-oxide (PyO) demonstrated remarkable features in several C–H bond activation transformations. Both, the NO and NH part of the amide are generally crucial to ensure the reaction.

##### 14.3.1.1. C(sp^2^)–H functionalisation

The pyridine-*N*-oxide moiety was used with first row eco-friendly transition metals such as Co and Ni catalysts. Besides its efficiency, a key advantage of this bidentate DG is its removal, which could be easily realized under basic conditions.^603^–^608^

###### 14.3.1.1.1. Alkenylation

Such DGs were successfully applied in Co- and Ni-catalysed C(sp^2^)–H alkynylation/annulation reactions.

**Scheme 102:**
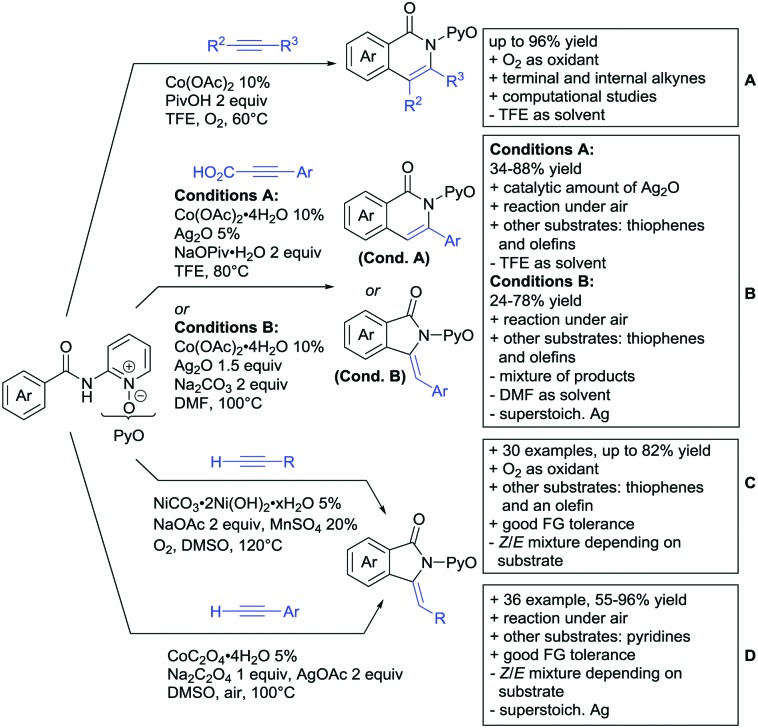
Alkenylation of C-(sp^2^) centres using 2-pyridine-*N*-oxide amide as DG.

An interesting approach towards heterocycles was developed by the groups of Macgregor and Ackermann (Scheme 102A).^609^ Under mild conditions and using oxygen as the sole oxidant, the synthesis of 6-membered isoquinolone derivatives from aromatic derivatives was achieved using Co(ii)-catalysis. The transformation turned out to be particularly efficient and highly regioselective and terminal as well as internal alkynes were suitable coupling partners. The regioselectivity of the reaction steered from electronic reasons as suggested by computational DFT investigations.

Niu and Song also investigated the synthesis of isoquinolone and isoindolinone derivatives by slightly changing the catalytic system *via* a Co-catalysed decarboxylative C–H activation using alkynylcarboxylic acids (Scheme 102B).^610^ The use of a catalytic amount of a Ag salt in the catalytic system to access isoquinolone compounds was a real asset of this approach, O_2_ playing the role of terminal oxidant.

Furthermore, Niu and Song reported the Ni-catalysed functionalisation of a panel of amides with terminal alkynes *via* a twofold C–H activation process leading to substituted 3-methyleneisoindolin-1-ones (Scheme 102C).^611^ The reaction demonstrated a good FG tolerance including halides and thiophene derivatives, and the stereoselectivity of the reaction was moderate to excellent (*Z*/*E* ratio from 52/48 to 100/0).

The same authors also developed a Co-catalysed alkynylation/annulation reaction of amides with terminal alkynes as coupling partners in presence of AgOAc as the terminal oxidant (Scheme 102D).^612^ The nature of the bidentate DG turned out to be crucial as other DGs were inefficient in that transformation. Both Co(ii) and Co(iii) might be used, CoC_2_O_4_·4H_2_O being the most efficient. The reaction was applied to a wide range of compounds (36 examples), although internal alkynes and aliphatic ones were not suitable coupling partners.

###### 14.3.1.1.2. Alkoxylation, amination

**Scheme 103:**
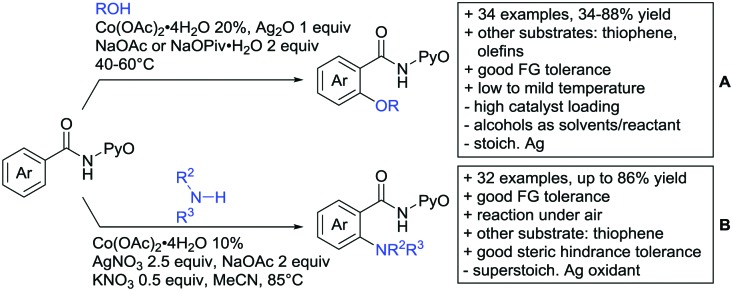
Alkoxylation and amination of C(sp^2^) centres using 2-pyridine-*N*-oxide amide as DG.

Another example showcasing the unique properties of this bidentate DG was reported again by Niu and Song, who published Co-catalysed alkoxylation of aromatic and olefinic carboxamides under mild reaction conditions (Scheme 103A).^604^ Later on, the same group extended this approach to the amination of arenecarboxamides with alkylamines (Scheme 103B).^605^

##### 14.3.1.2. C(sp^3^)–H functionalisation

**Scheme 104:**
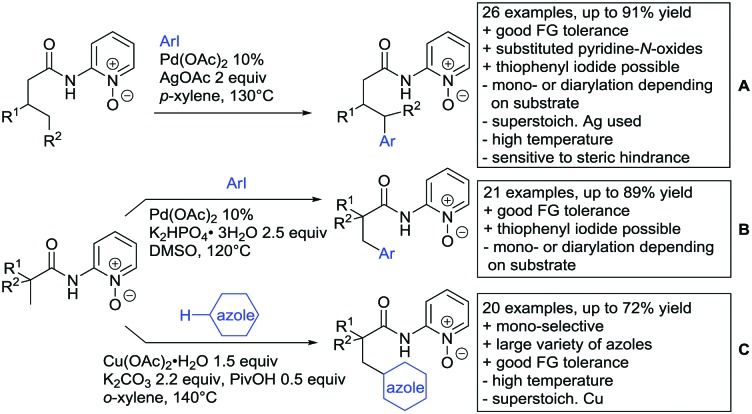
Arylation and amination of unactivated C(sp^3^)–H bonds using 2-pyridine-*N*-oxide amide as DG.

This DG was also successfully applied in C(sp^3^)–H bond functionalisation. In 2015, Zeng and Lu reported a Pd catalysed pyridine-*N*-oxide-directed arylation of alkane carboxamides with aryl iodides (Scheme 104A).^606^ The functionalisation of both primary and secondary C(sp^3^) centres was achieved at the β- and even the γ-position on suitable substrates. Substitution on the pyridine part of the DG with halides, CF_3_ and NO_2_ groups was tolerated. Note that mono- and di-arylated products were obtained and the overall efficiency of the transformation was substrate-dependent.

A similar transformation was then further studied by the same authors, and aliphatic amides were functionalised with a large number of aryl iodides leading to mono- or di-functionalised products depending on the substrate (Scheme 104B).^607^

This DG was not restricted to the formation of C–C bonds, since very recently, the group of You demonstrated that this bidentate DG was successfully used in the oxidative amination of unactivated C(sp^3^)–H bonds with azoles *via* a Cu mediated process (Scheme 104C).^608^ Note that an extension of this transformation *via* Ni catalysis was also reported using in that case the amide derived from 8-aminoquinoline as DG.

#### 14.3.2. Oxalyl amide group

In 2014, Yao and Zhao designed a new auxiliary, the *N*,*O*-bidentate oxalyl amide group (NHOA or NHCODIPA).^613^ Since this initial study, the system was applied for several transformations. This DG was mainly cleaved under basic conditions (KOH/EtOH, 80 °C;^590^ NaOH, THF/MeOH, 50–100 °C^614^–^617^ or NaOMe, MeOH, rt^618^) or *via* a *N*-Boc protection and then cleavage under basic conditions (KOH, EtOH/Et_2_O, rt).^591^

##### 14.3.2.1. C(sp^2^)–H functionalisation

###### 14.3.2.1.1. Arylation and alkenylation

**Scheme 105:**
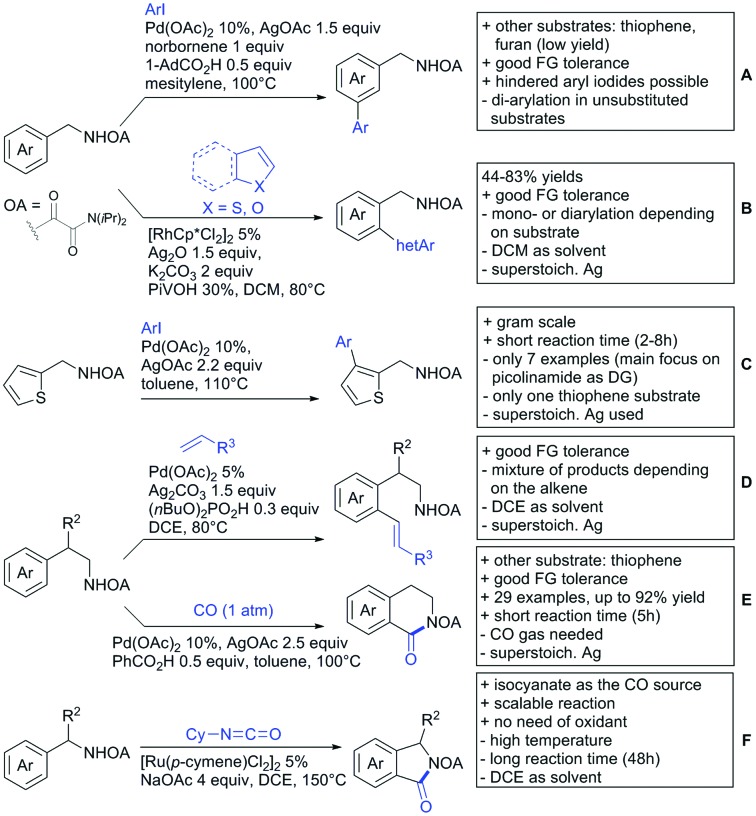
Functionalisation of C(sp^2^) centres using oxalyl amide as DG.

Shi and Zhao reported the Pd-catalysed *meta* arylation of β-arylethylamine *via* a Catellani reaction using NHOA as a bidentate DG (Scheme 105A).^619^ Using norbornene as the transient mediator, the transformation was tolerant to various FGs, leading to the expected compounds in good yields and various aryl iodides bearing *para*, *meta* or *ortho* substituents were suitable. It is worth to mention that the mono *meta* arylation was possible on substrates bearing substituents at the *ortho* and *meta* positions. In contrast, when unsubstituted arenes were reacted, only *meta* di-arylated products were obtained. To further showcase the synthetic utility of their approach towards the synthesis of polyfunctionalised β-arylethylamines, the authors investigated the Pd-catalysed oxalyl-amide-directed functionalisation of *meta* arylated β-arylethylamines. This protocol turned out to be general and alkynylation, iodination, acetoxylation and amination reactions were achieved in moderate to good yields (42–82%), although some di-alkynylated products were produced in 17–30% yields (not shown).^620^

The same group also investigated Rh(iii)-catalysed oxidative C–H/C–H cross-couplings of benzylamines and heteroarenes (Scheme 105B).^614^ When the *ortho* position was unsubstituted, mixtures of mono- and di-heteroarylated products were obtained, the ratio mono-/di-being dependent on the substitution pattern of the aromatic system.

In the course of their investigation for the Pd-catalysed arylation of 3-(aminoethyl)thiophene derivatives, the group of Babu showed that among the possible DGs for this transformation, the NHOA DG turned out to be efficient affording the products in 57 to 75% yields and the reaction was also performed on a gram scale (Scheme 105C).^568^

The NHOA DG was also used in Pd-catalysed olefination of β-arylethylamines (Scheme 105D).^621^ The reaction works with a broad array of functionalised olefins and a good selectivity for mono-olefination was observed in case of electron rich olefins such as allyl acetate, 3,3-dimethyl-1-butene and styrene derivatives. In contrast for more reactive olefins such as acrylamide, acrylonitrile, acrylaldehyde and vinyl sulfone, a mixture of mono- and di-olefinated products was obtained.

###### 14.3.2.1.2. Carbonylation

Carbonylation of β-arylethyl- and benzylamines were achieved using different reagents. Indeed, in 2016, a first report from Huang and Zhao on the Pd-catalysed carbonylation using carbon monoxide as the CO source was reported (E).^615^ Then, Zhao and Shi developed an improved protocol based on a Ru-catalysed carbonylation reaction, which demonstrated interesting features such as the use of isocyanate as CO source and the absence of oxidants (F).^618^ 21 derivatives were functionalised with up to 96% yields.

###### 14.3.2.1.3. Silylation and germanylation

**Scheme 106:**
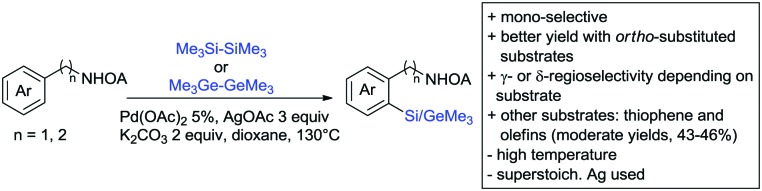
Silylation and germanylation of C(sp^2^) centres using oxalyl amide as DG.

This bidentate DG was also employed for the regioselective and monoselective silylation of benzylamine and phenylethylamine compounds *via* Pd catalysis at the γ- and δ-positions, respectively (Schemes 106, 26 examples, 43–90%).^616^ This reaction was not restricted to aromatic derivatives since, using slightly modified reaction conditions, the silylation of 2-methylaniline derivatives was possible, albeit restricted to four examples in low to moderate yields (21–49%). It is worth to mention that the germanylation of benzylamines was also achieved by replacement of the hexamethyldisilane with Ge_2_Me_6_ (4 examples, 37–82%).

##### 14.3.2.2. C(sp^3^)–H functionalisation

**Scheme 107:**
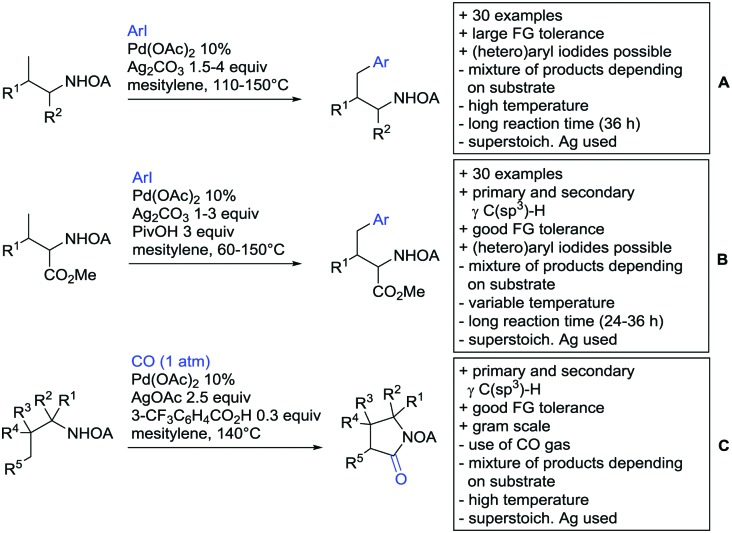
Functionalisation of C(sp^3^) centres using oxalyl amide as DG.

In 2015, the γ-arylation of aliphatic amines with aryl and heteroaryl iodides was investigated by Zheng, Huang and Zhao (Scheme 107A).^622^ Upon Pd-catalysis, mono- or di-arylated products were obtained (47–83%) depending on the substitution pattern of the substrates. When heteroaryl iodides were used as coupling partners, low to high yields (23 to 78%) were obtained. Later, the same group used a similar catalytic system for the functionalisation of amino acids (Scheme 107B).^623^ ArI and heteroaryliodides were suitable coupling partners leading to the corresponding products in 45–86% and 36–83% yields, respectively. The carbonylation of aliphatic amines *via* a Pd-catalysed C–H bond functionalisation under carbon monoxide atmosphere was reported by Yao and Zhao (Scheme 107C).^617^ Primary and secondary C(sp^3^) centers were functionalised in low to high yields (12–87%) and extended to the functionalisation of benzylamines (8 examples, 71–98%) (not shown). The reaction was even conducted on a gram scale. The authors suggested that 3-(trifluoromethyl)benzoic acid was important to stabilize the Pd intermediate.

#### 14.3.3. Amide derived from 1-aminoanthraquinone

**Scheme 108:**
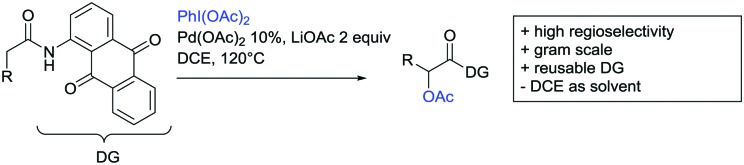
Acetoxylation of C(sp^3^) centres using amide derived from 1-aminoanthraquinone as DG.

A unique example of Pd-catalysed directed acetoxylation using the amide derived from 1-aminoanthraquinone as bidentate DG was reported. In 2015, Cheng, Zhu and Zhang disclosed the Pd-catalysed acetoxylation of aliphatic amides at the α-position *via* a four-six-membered bicyclic metallacycle (Scheme 108A). Amides containing primary α-C(sp^3^)–H and α-methylene C(sp^3^)–H bond were smoothly functionalised in a selective manner (32–92% yields) and only the mono-acetoxylated products were observed. The bidentate DG was cleaved under basic conditions (NaOH, MeOH, reflux) and recycled.^624^

## 15. Heterocyclic DGs in C–H functionalisation

As for carbonyl-based and bidentate moieties, heterocycles are also a very common class of DGs for the functionalisation of C(sp^2^) and C(sp^3^) centres. The heterocycles that can be used as DGs are those featuring a C(sp^2^)-hybridised nitrogen atom, which act as the coordinating moiety for the formation of the metallacyclic intermediate essential to the C–H functionalisation. These DGs have attracted a lot of interest in the field for a number of reasons: (1) heterocycles are commonly found in bio-active and drug-like molecules, which allows late-stage modifications without requiring DG installation/removal; (2) if needed can be installed on the substrate in a number of ways; (3) despite being generally considered strong-coordinating DGs, their coordinating properties can be somewhat altered by substitution or by changing the heterocycle used for a specific transformation.

Although DG cleavage can be sometimes tricky with monoheterocycles *(i.e.* pyridine), several methods have been developed during the years, thus increasing their synthetic utility. In the case of diheterocycles, cleavage (*e.g.* for pyrimidine) or modification of the DG (for example by ring opening) is intrinsically easier, although not often reported in publications involving less common heterocycles. The available cleavage/modification methods for heterocyclic DGs are discussed in each specific sub-section. In this section, yields have been specifically reported in case of particularly high yields (most examples >80–90%) or lower than average (most <70% or lower) results, and in case of strong substrate-dependence. If not reported, average yields of 70–80% for most examples is to be assumed (see discussion in the Introduction section).

### 15.1. Pyridine as DG

The use of pyridine (as well as other heterocycles such as pyrazole and pyrimidine) as a DG in C–H functionalisation chemistry dates back to the late 1990s–early 2000s, when it was pioneered by Murai/Chatani first, who reported Ru-catalysed alkylations and carbonylations on C(sp^2^) and C(sp^3^) centres,^625^–^627^ and then by Sanford^628^,^629^ and Daugulis,^630^ who extended its use to Pd-catalysed acetoxylations and arylations. Since then, pyridine is one of the most frequently applied DGs in C–H functionalisation chemistry. This is possibly due to its simplicity and the easy installation into aromatic rings or aliphatic chains *via* cross coupling^631^–^633^ or other methods.^634^ A great number of reactions have been reported with this DG, with a variety of protocols, reagents and reaction conditions. Recently this DG has also been commonly used for *meta*-selective C(sp^2^)–H functionalisation using Ru catalysts,^635^,^636^ which will not be treated in this review. Moreover, pyridine is the DG of choice for specific substrates, such as 2-pyridones, for which other DGs have proved ineffective.

Although this DG is typically not removable when attached to the substrate *via* a C–C bond, the ubiquity of pyridine rings in many complex molecules (*e.g.* bioactive compounds), partially compensates this disadvantage, as its removal might not be required in all cases. The removal of this DG can nonetheless be achieved when attached to the substrate *via* a C-heteroatom bond (as is the case for *N*-(2-pyridyl)indoles, *N*-(2-pyridyl)anilines, or *O*-(2-pyridyl)phenols). These cleavage methods are discussed in Section 15.3.

#### 15.1.1. C(sp^2^)–H bond functionalisation

##### 15.1.1.1. Arylation

**Scheme 109:**
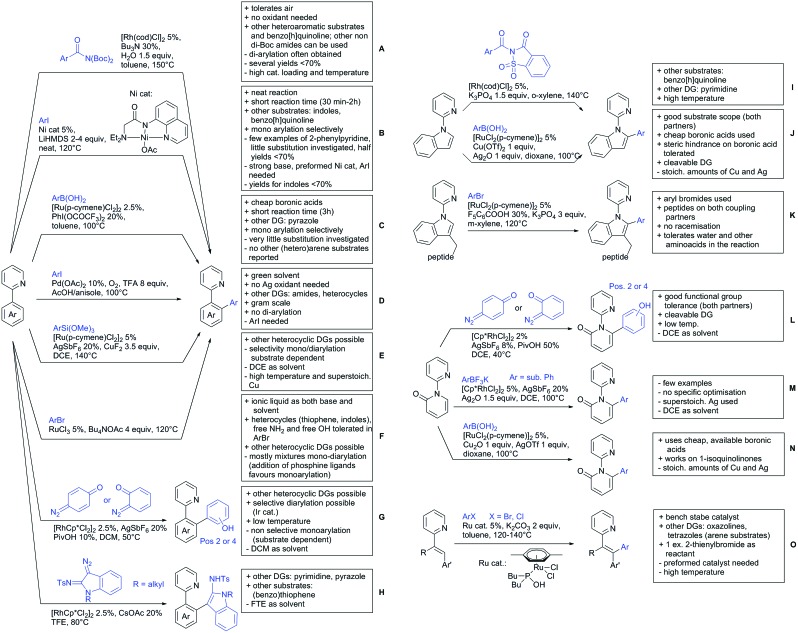
C(sp^2^)–H arylation using pyridine as DG.

Many arylation procedures with pyridine as DG have been reported before 2014, and therefore are not included in this review. Nonetheless, several methods have been developed in the last few years. Szostak reported an unusual C–C cleavage/C–H functionalisation using activated benzamides as arylating agents (Scheme 109A),^637^ while boronic acids under Ru catalysis were utilised by Maheswaran (Scheme 109C).^638^ The hyper-valent iodine additive in this reaction was suggested to form a charge-transfer complex with the substrate, generating an aromatic radical cation^639^ which facilitates the formation of the ruthenacycle intermediate.

Punji and Wang reported the use of aryl iodides with Ni and Pd catalysis respectively (Scheme 109B and D).^640^,^641^ While the first required preformed Ni(ii) catalysts and the use of strong base, the latter employed O_2_ as oxidant and green solvents, although a 10% Pd loading was required. Notably, both methods provide selective monoarylation, which is not always achieved. A coupling with aryl silanes was reported by Szostak (Scheme 109E).^642^ Although this procedure avoids the use of stoichiometric Ag oxidants, still a superstoichiometric amount of CuF_2_ was required, and a substrate-dependent selectivity (mono or diarylation) was observed. A recent paper by Kantchev demonstrated the use of the ionic liquid Bu_4_NOAc as both base and solvent in the RuCl_3_-catalysed coupling with aryl bromides (Scheme 109F).^643^ The reaction gave mostly mixtures of mono and diarylated products, but at the same time tolerates heteroaryls, free NH_2_- and free OH-substituted aryl bromides. In 2015 Wang reported the Rh (monoarylation) or Ir (diarylation) catalysed reaction with diazoquinones leading to phenol-functionalised arenes (Scheme 109G).^644^ The next year a related transformation, using 3-diazoindolin-2-imines as reactants was disclosed by Wang and Lu, giving 3-aryl-2-aminoindoles (Scheme 109H).^645^

Inspired by Szostak's arylation method using aromatic amides as arylation reagents (Scheme 109A), Zeng performed the arylation of indoles (and benzo[*h*]quinoline) using *N*-acyl saccarines as reagent under similar conditions (Scheme 109I).^646^ The use of boronic acids for indole arylation was instead reported by Kapur. This method provides good substrate scope and yields, although requiring stoichiometric amounts of both Cu and Ag (Scheme 109J).^647^ Ackermann recently reported the arylation of peptide-substituted indoles using aryl bromides. This procedure afforded good yields of arylated products, with no racemisation and good robustness. Importantly, peptides are tolerated on both coupling partners (Scheme 109K).^648^

The arylation of 2-pyridones with heteroaromatic compounds *via* cross dehydrogenative coupling was first reported by Miura and Hirano in 2014.^649^ Since then, further arylation procedures have been reported making use of diazoquinones and arylboron compounds (Scheme 109L–N).^650^–^652^

An example of stereoselective C–H arylation of olefins was reported by Ackermann in 2016 using a preformed phosphinous acid Ru(ii) complex as catalyst (Scheme 109O).^653^ This method could also be applied to aromatic substrates using tetrazoles and oxazolines as DGs.

##### 15.1.1.2. Alkylation

Alkylation can occur *via* different mechanisms: (i) addition to unsaturated moieties, (ii) cross couplings, and (iii) carbene insertions. Due to the many protocols reported for such functionalisation with pyridine as DG, these strategies are treated separately. Cascade alkylation/cyclisation involving the pyridine DG will also be reported separately due to the different nature of the products obtained.

##### 15.1.1.3. Addition to double bonds

**Scheme 110:**
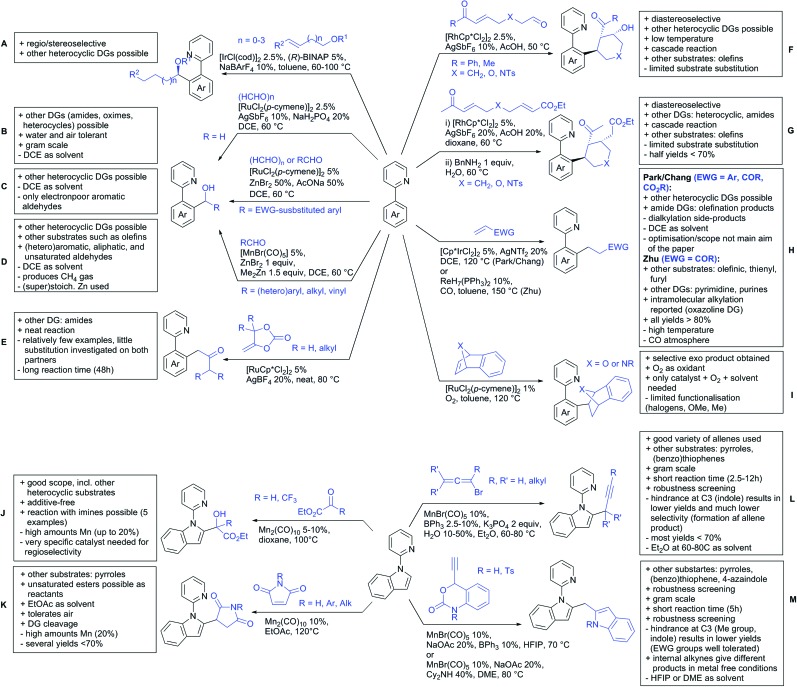
Pyridine-directed C(sp^2^)–H alkylations *via* addition to double bonds.

In 2015 Nishimura reported the Ir-catalysed regioselective (branched products) hydroarylation of vinyl, allyl and other alkenyl ethers, leading to alpha-arylated ethers.^654^,^655^ This reaction could be performed in a stereoselective manner using chiral biphosphine ligands (Scheme 110A).

The group-directed addition to aldehydes has been reported a number of times, and results in alpha-arylated alcohols. A Lewis or Brønsted acid additive in these reactions is necessary to activate the carbonyl group, and reduce the reversibility of the aldehyde insertion into the C–M bond. Zhou^656^ and Ding^657^ reported the Ru-catalysed addition to formaldehyde and electron-poor benzaldehydes (Scheme 110B and C). The scope in aldehydes was notably increased by Wang *et al.*, using a Mn catalyst and a combination of ZnBr_2_ and Me_2_Zn. This allowed the use of (hetero)aromatic, aliphatic, and even unsaturated aldehydes as reactants; moreover, heteroaromatic and olefinic substrates could also be easily functionalised (Scheme 110D).^658^ The synthesis of 2-oxoalkyarenes was achieved by Kakiuchi using cyclic alkenylcarbonates, synthetic equivalents of enolates (Scheme 110E).^659^ Despite the originality of the protocol, its use with pyridine as DG was only demonstrated on relatively few examples, and required a long reaction time (48 h).

An interesting protocol for a directed cascade C–H functionalisation/cyclisation was reported by Ellmann, giving diastereoselective formation of 3 contiguous stereocentres (Scheme 110F and G).^660^,^661^ The benzylamine in the second protocol acts as an organocatalyst (enamine catalysis) to facilitate the intramolecular Michael addition, which is more difficult than in the first case.^661^ These are two of the relatively rare examples where the cascade cyclisation does not involve the DG itself, which is often the case (*e.g.*Scheme 112).

Park and Chang reported the effect of different DGs on the chemoselectivity of the reaction of aromatic substrates with activated alkenes.^662^ While with pyridine DGs, addition to the double bond was obtained selectively (Scheme 110H), the use of an amide DG in the same conditions resulted in selective olefination instead. This switch in selectivity was limited to the Ir catalyst used. The same reaction, catalysed by Re, was instead reported by Zhu and co-workers (Scheme 110H).^177^ This protocol, occurring under CO atmosphere (CO acts as ligand for Re), is one of the few Re-catalysed C–H activation processes, and proved very efficient for a variety of substrates, including olefinic and heteroaromatic ones. In 2015 Bolm reported the hydroarylation of unactivated bicyclic alkenes with Ru catalysis using O_2_ as oxidant (Scheme 110I).^663^

The functionalisation of indoles with activated ketones or aldehydes (alpha EWG-substituted) under Mn catalysis was reported by Ackermann (Scheme 110J).^664^ Although high loadings of Mn catalyst were used (up to 20% of the metal), the reaction gave good yields with a broad scope, and no additives were used (*cf.*Scheme 110B–D). It is worth noting that the C2 regioselectivity, despite the DG, was only observed with two very specific Mn catalysts ([Mn_2_(CO)_10_] and [MnBr(CO)_5_]), while all the other catalysts tested resulted in C3 functionalisation (no DG effect).^664^

A similar procedure for the addition to maleimides was reported recently by Song and Gong (Scheme 110K).^665^ The reaction was air tolerant and worked effectively in EtOAc. Importantly, both these protocols were applied to the functionalisation of other heterocycles (*e.g.* pyrroles, selectively mono-functionalised).

Recently, the Glorius group developed two important methods for the C–H propargylation and 2-(indolyl)methylation of indoles using substituted bromoallenes and 4-ethynyl benzoxazinanones (Scheme 110L and M).^666^,^667^ Propargylation is a challenging transformation, as the corresponding allenylation might instead occur more easily. For this Mn-catalysed process, the use of BPh_3_ as Lewis acid and a catalytic amount of H_2_O proved crucial for obtaining good yields and selectivity (with respect to the allene product). The reaction works well with a variety of functionalised allenes, including 1,3,3-trialkyl, 3,3-dialkyl (giving terminal alkyne products), 1,3-dialkyl and 1-monoalkyl allenes, and with other heterocyclic substrates such as pyrroles and (benzo)thiophenes (Scheme 110L).^666^ A robustness screening was also performed for this reaction, showing compatibility with functionalities such as aniline, furan, ketones, amides and acetals. The use of 4-ethynyl benzoxazinanones, still under Mn catalysis, results in the C–H (2-indolyl)methylation of indoles and other heterocycles *via* alkyne insertion into the intermediate manganacycle, release of CO_2_, formation of an allene–Mn intermediate complex, and cyclisation with the nitrogen nucleophile (Scheme 110M).^667^ Even in this case a robustness screening showed compatibility with a variety of FGs.

Pyridine-directed, Ni-catalysed alkylation of 2-pyridones using a variety of aliphatic alkenes and dienes (including cyclooctadiene) was reported by Miura and Hirano in 2017.^668^ Despite being one of the few examples of alkylation on such substrates in recent times, the reaction proved fairly sensitive to the substitution pattern on the pyridone and the identity of the aliphatic alkenes/dienes used.

##### 15.1.1.4. Cross coupling and carbene insertion

**Scheme 111:**
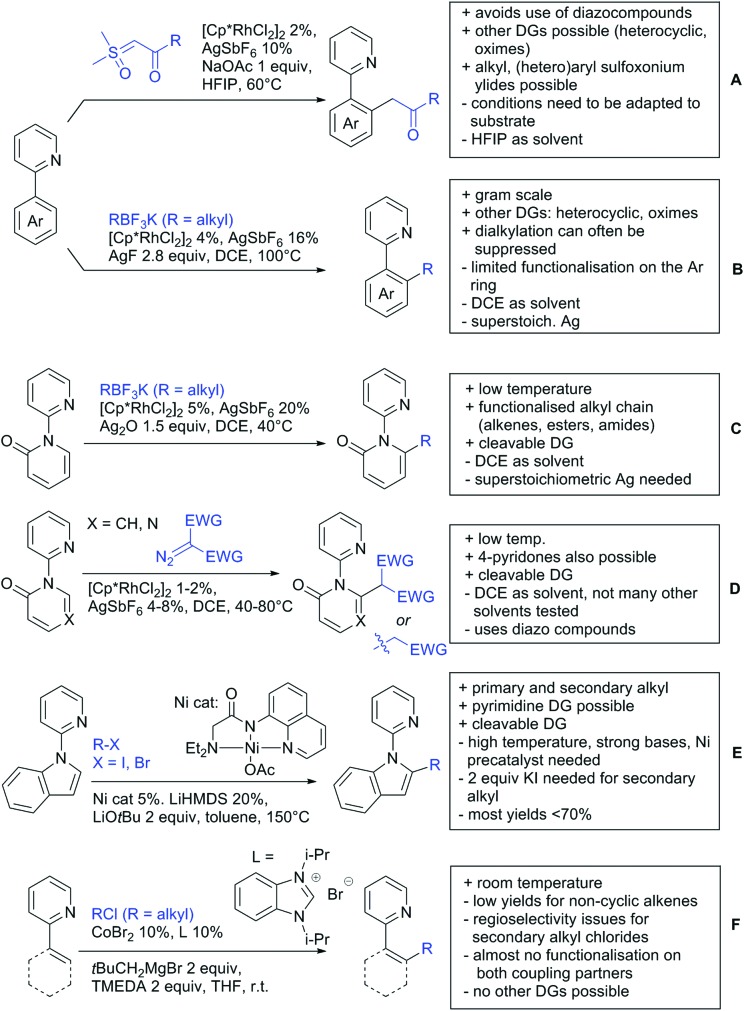
Pyridine-directed C(sp^2^)–H alkylations *via* cross-coupling and carbene insertion.

A number of alkylation reactions based on cross coupling type of reactions have been reported. A very recent example by Aissa's group demonstrated the use of sulfoxonium ylides as a replacement reactant for diazo compounds, leading to α-arylated aromatic or aliphatic ketones in good yields (Scheme 111A).^669^ This was the first example of the use of such reactants, safer alternatives to diazonium compounds,^670^ in group-directed C–H functionalisation. Li and Liu reported very similar conditions for the alkylation of arenes and 2-pyridones respectively with alkyltrifluoroborates (Scheme 111B and C).^651^,^671^ The C6 alkylation of 2-pyridones was also performed *via* carbene insertion using diazo compounds (Scheme 111D).^672^

The Ni catalysed functionalisation of indoles using alkyl halides was achieved by Punji and co-workers (Scheme 111E).^673^ Although being a rare example of Ni-catalysed alkylation, this method required the use of a preformed Ni-catalyst, catalytic amounts of strong Li-amide bases, 2 equiv. of base and high temperatures to result in medium to good yields of functionalised indoles. The use of secondary alkyl halides was also possible, but 2 equiv. of KI as additive were required in this case.^673^

The functionalisation of non-aromatic C(sp^2^)–H bonds is considerably rarer, and much underdeveloped. Yoshikai reported the alkylation of cyclic alkenes with alkyl chlorides using Co catalysis (Scheme 111F).^674^ Despite having benefits such as being base metal-catalysed and allowing functionalisation at room temperature, the reaction requires the use of both a catalytic carbene and a superstoichiometric amine ligand (TMEDA), has a limited scope, and presents some regioselectivity issues for secondary alkyl chlorides, and therefore lacks generality.

##### 15.1.1.5. Alkylation/cyclisation

**Scheme 112:**
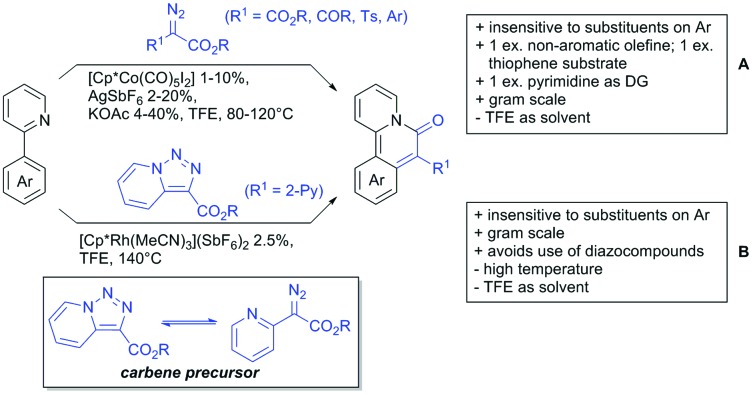
Pyridine-directed alkylation/cyclisation.

The functionalisation of C–H bonds with unsaturated moieties can result in the subsequent involvement of the DG in a cyclisation process between the newly installed functionality and the nucleophilic nitrogen atom of the pyridine. In 2015 Glorius investigated the use of diazomalonates in combination with Co catalysis. Instead of obtaining the expected alkylation product, the corresponding fused heterocycles, derived from nucleophilic attack of the pyridine to the ester moiety of the alkyl group, were obtained (Scheme 112A).^675^ Later the same group reported the use of pyridotriazoles as carbene precursors for a similar reaction. In this case, Rh catalysis had to be used, as Co pre-catalysts did not produce any cyclised or alkylated product (Scheme 112B).^676^

##### 15.1.1.6. Allylation

A large variety of allylation procedures for the functionalisation of indoles have been reported in recent years, with a number of different reactants and leading to variedly functionalised side chains. Two main different classes of reactants have been utilised: alkenes with (1) a leaving group, or (2) a strained ring in alpha to the double bond. In both cases most mechanistic proposals involve a migratory insertion of the double bond into the metallacycle initially formed, followed by a general β-elimination of a suitable leaving group (β-oxygen, β-fluoro, β-bromo), or the release of ring strain *via* a β-carbon cleavage. For the latter, the β-carbon elimination is favoured over β-hydride elimination due to the release of the ring strain *via* ring opening. Alternative mechanisms occurring *via* oxidative additions have been suggested in some instances.^677^

**Scheme 113:**
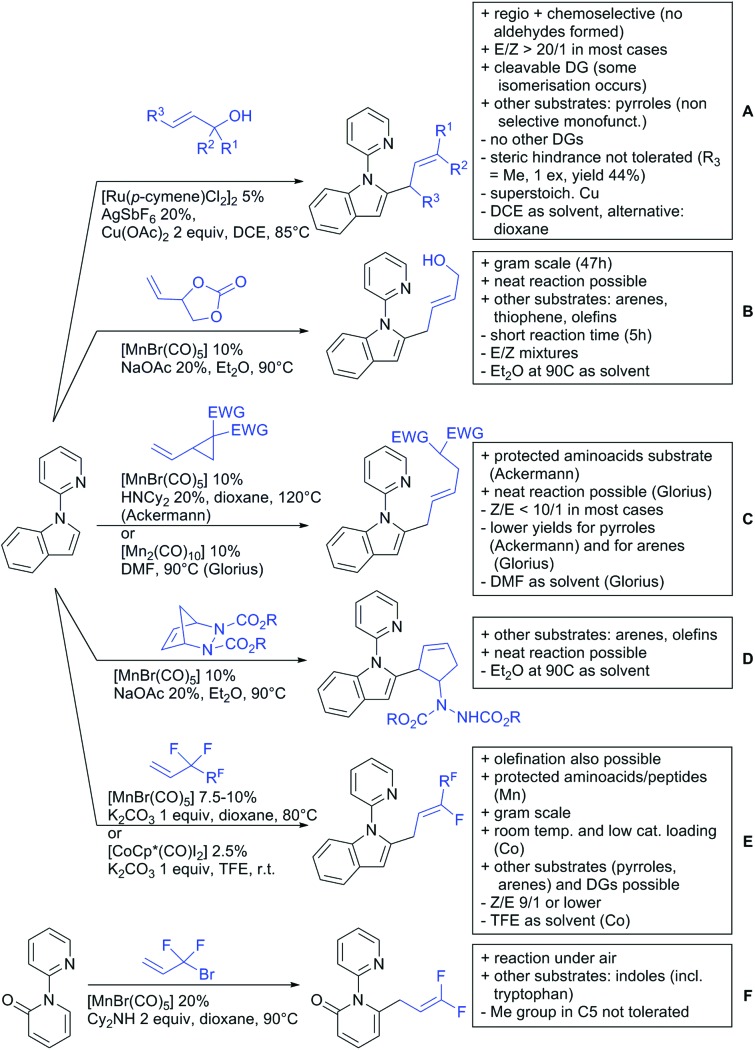
Pyridine-directed allylations on C(sp^2^) centres.

In 2016 Kapur reported the allylation of indoles with allyl alcohols (unsubstituted, 1-monosubstituted, 1,1-disubstituted), which selectively resulted in non oxygenated products (Scheme 113A).^678^ The chemoselectivity arises from a selective β-hydroxide elimination rather than β-hydride elimination step in the mechanism. Advantages are regio-, chemo-, and diastereoselectivity, and the cleavage of the DG in a number of compounds.^678^ Many papers have appeared using Mn and Co catalysis, in combination for example with vinyldioxazolones^679^,^680^ (Scheme 113B), vinylcyclopropenes^679^,^681^,^682^ (Scheme 113C, Mn cat. shown), or diazabicyclic compounds^679^ (Scheme 113D). Among these, notable are the fluorinated side chains introduced with Ackermann's (Mn,^683^Scheme 113E, and Co^684^) and Zhang's (Mn, Scheme 113F)^685^ procedures.

##### 15.1.1.7. Alkenylation

**Scheme 114:**
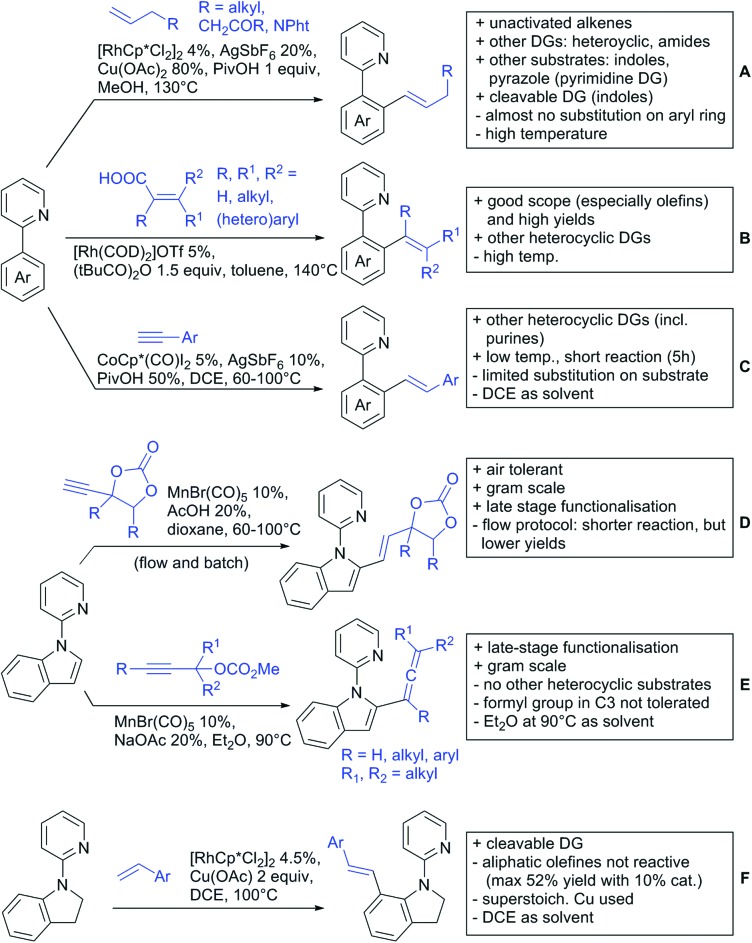
Pyridine-directed C(sp^2^)–H olefination.

Olefination reactions can occur *via* different mechanisms, mostly oxidative dehydrogenative coupling with olefins (Fujiwara–Moritani coupling, mostly with activated olefins), and hydrofunctionalisation of alkynes. Zhang reported the use of unactivated olefins for the functionalisation of arenes (Scheme 114A).^686^ The use of such alkenes as reactants is rare, as most C–H olefinations employ activated styrenes and acrylates. Li and Xu reported the use of acrylic acids as reactants, in combination with pivalic anhydride as additive (Scheme 114B).^687^ The formation of vinylic anhydride or mixed pivalic/vinylic anhydrides as intermediate results in a decarbonylative olefination process. The method shows good scope and generally excellent yields. Co catalysis was used for the hydroarylation of aromatic alkynes (Scheme 114C).^688^ The hydrofunctionalisation of alkynes bearing a leaving group in the propargylic position can proceed in two different ways. In case a Brønsted acid is added to the reaction, the olefination product is obtained, as recently reported for the olefination of indoles (Scheme 114D).^689^ In such a case the leaving group is maintained in the molecule and can be utilised successively. In the absence of acids the corresponding allene is obtained instead, as in Scheme 114E.^689^,^690^ Finally, noteworthy is the C7 functionalisation of indolines using styrenes, reported by Loh (Scheme 114F).^691^

##### 15.1.1.8. Alkenylation/cyclisation

**Scheme 115:**
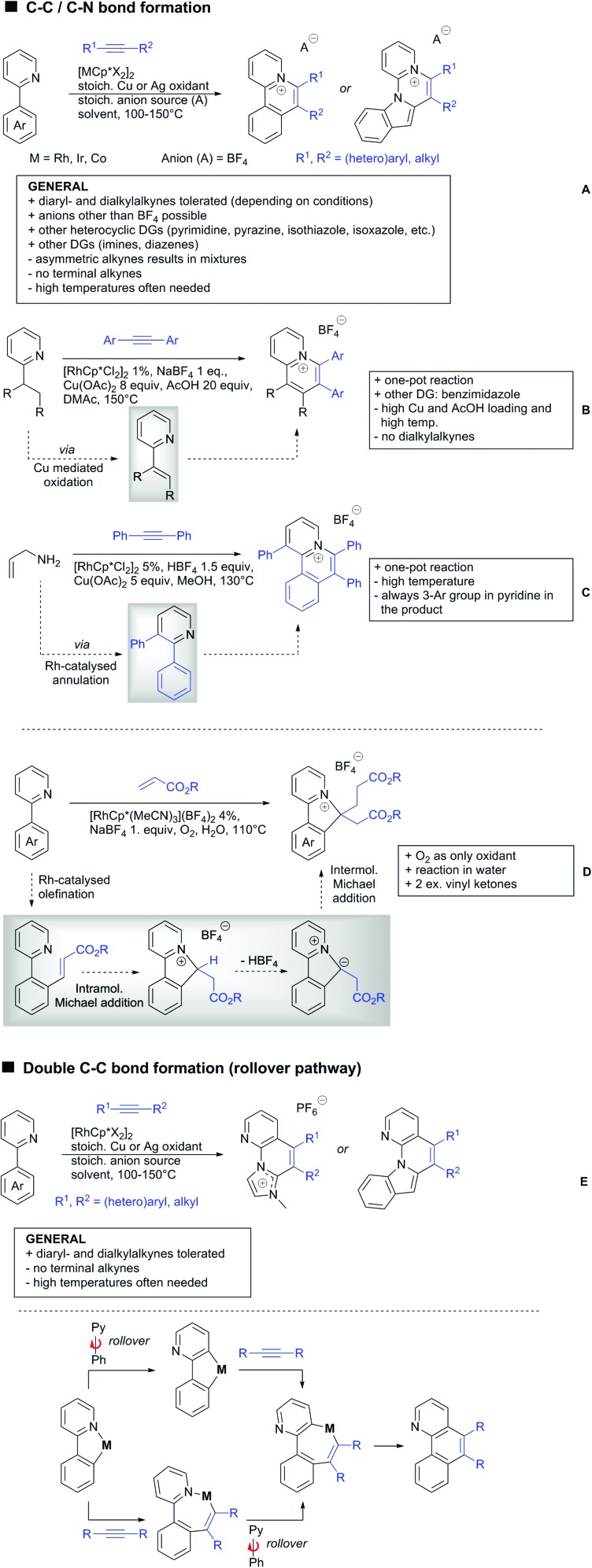
Pyridine-directed olefination/cyclisations *via* C–N bond formation or rollover pathways.

The hydrofunctionalisation of dialkyl- or diarylalkynes in the presence of Rh(iii), Ir(iii), or Co(iii) catalysts, a (super)stoichiometric oxidant and a non-coordinating anion source can result, instead of the olefinated product, in cyclisation products (extended π systems), interesting for their fluorescent properties and potential applications in material chemistry.^692^ This process results in the formation of a C–C and a C–N bond in a cascade manner (Scheme 115A). Several examples have been reported recently, making use of diaryl- or dialkylalkynes,^693^–^695^ including the isolation of a 7-membered cobaltacycle intermediate (see general mechanism in Scheme 115, bottom).^696^ Interesting one-pot protocols have been reported by Cheng and Jun: in one case the formation of the olefinic substrate from an alkyl chain occurred *in situ via* oxidation, and in the other case the reaction of allylamine with two equivalents of the alkyne resulted in the formation of the 2-pyridinylarene substrate first, followed by olefination/cyclisation (Scheme 115B and C).^697^,^698^ Another cyclisation product can be obtained by using acrylates or similar as the olefination agent. As reported by Cheng, the Rh-catalysed reaction with 2 equivalents of acrylates in water and using O_2_ as oxidant, results in the formation of pyridoisoindolium salts (Scheme 115D).^699^ The process was proposed to occur *via ortho* olefination, followed by intramolecular Michael addition of the pyridine to the activated olefin, deprotonation, and finally a second, intermolecular, nucleophilic attack to a second molecule of the acrylate.

Alternatively, the use of Rh might also result in the formation of two C–C bonds *via* a so-called “rollover cyclometallation” mechanism, where the nitrogen atom of the pyridine ring is not involved in the cyclisation, and can be used to promote a further C–H functionalisation (Scheme 115E). While the rollover mechanism is more common for doubly coordinating ligands, such as bipyridines,^700^ or in the case of a pyridine–carbene moiety like the one in Scheme 115E,^701^ its occurrence is far less common in other substrates (*e.g.* indole substrate in Scheme 115E),^702^ and is obviously facilitated by the absence of a non-coordinating anion in the reaction mixture.

##### 15.1.1.9. Alkynylation

Generally speaking, alkynylation reactions in C–H functionalisation typically employ two main reactants: the hypervalent iodine compound TIPS-EBX (1-[(triisopropylsilyl)ethynyl]-1,2-benziodoxol-3(1*H*)-one), and TIPS-bromoacetylene. Variations include other silyl protecting groups instead of TIPS or, more rarely, aryl(bromo)acetylenes or alkyl(bromo)acetylenes. Recent additions to the reagent pool for this transformation include propargylic alcohols (see below). Although the silyl protecting group can be easily cleaved, and the resulting acetylene subsequently functionalised, this is still an intrinsic limitation of this type of functionalisation. The use of terminal alkynes was shown to result in the formation of considerable amounts of Glaser–Hay coupling products.^703^,^704^

**Scheme 116:**
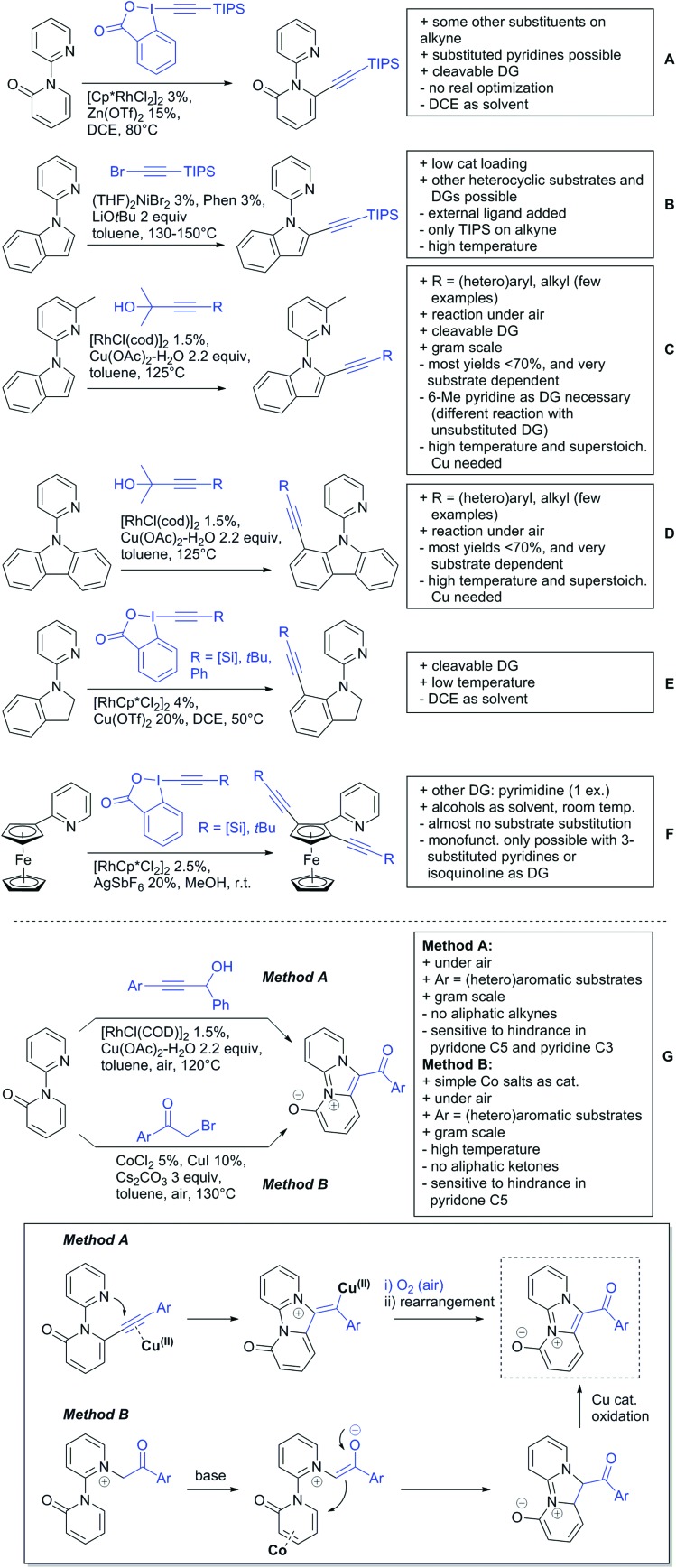
Alkynylation and alkynylation/cyclisation using pyridine-based DGs.

Relatively few examples of alkynylation with a pyridine DG have been reported recently. In 2015 Li reported the C6-alkynylation of pyridones with TIPS-EBX with a Rh catalyst,^705^ while Ni-catalysed reaction of indoles with TIPS-bromoacetylene was reported by Punji^706^ (Scheme 116A and B). Wen reported the use of 1,1-dimethylpropargyl alcohols as alternative reactants for alkynylation reactions. This reactant avoids the use of halogenated compounds and only results in the formation of acetone as side product. The intermediate metal–alkyne complex is obtained *via* β-carbon elimination from the alcohol-ligated catalyst, as previously reported.^707^ Application of such propargylic alcohols to the functionalisation of indoles and carbazoles,^703^ although resulting in relatively low and substrate-dependent yields, allows a larger variety of alkynes to be used (alkyl and aryl substituted, Scheme 116C and D) than the classical reactants. Noteworthy in this case are the monofunctionalisation of carbazoles (carbazoles get usually doubly functionalised) and the necessity of a 6-Me pyridine DG for the indoles (without the 6-Me another transformation occurs^704^).

Finally, alkynylation of indolines (C7) and ferrocenes have been reported by Loh and You using R-EBX (R = [Si], *t*Bu) hypervalent iodine species (Scheme 116E and F).^691^,^708^

Cascade alkynylation–cyclisation reactions are also possible. For instance, two methods for the synthesis of dipyridoimidazolium compounds were reported by Li (Scheme 116G).^709^,^710^ Despite giving the same product, the two methods are mechanistically very different. While Method A is technically a cascade directed alkynylation/cyclisation (Rh–alkyne complex is obtained *via* β-carbon elimination from the alcohol-ligated intermediate^707^), Method B was proposed to involve pyridine alkylation first, followed by enolisation and nucleophilic attack of the enol to the Co-activated pyridone ring, and Cu-mediated oxidation.

##### 15.1.1.10. Cyanation

Classical reactions for the cyanation of organic compounds are the Sandmeyer or the Rosenmund von Braun reaction, or cross coupling reactions between aryl halides and cyanide salts. All methods, in their classic version, require prefunctionalised substrates, and more importantly, the use of inorganic cyanides (very toxic) in the reaction. Although a number of (safer) reactants have been recently utilised for the cyanation of C–H bonds, still a common feature is the atom uneconomic nature of these reactants, where more than 50% of the reactant mass is actually wasted.

**Scheme 117:**
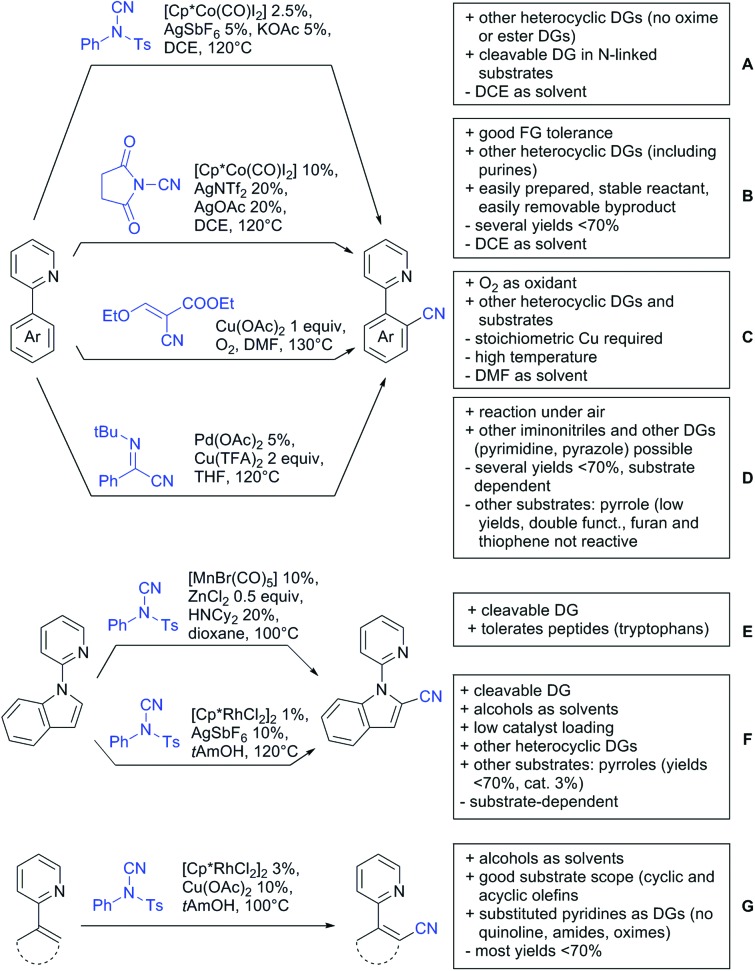
Pyridine-directed *ortho* cyanation of C(sp^2^)–H bonds.

Co-catalysed reactions were reported by Ackermann711*a* and Chang711*b* in 2015, employing respectively *N*-cyano-*N*-phenyltosylamide (NCTS) and *N*-cyanosuccinimide (Scheme 117A and B). While the first protocol required a lower amount of additives and catalyst, the use of a more stable reactant, and the good FG tolerance in the second protocol are noteworthy. A third protocol, reported by Jiang and co-workers, employed ethyl(ethoxymethylene)cyanoacetate as a new reagent under aerobic oxidation conditions, but unfortunately stoichiometric Cu salts were required (Scheme 117C).^712^ Similar conditions (superstoichiometric Cu, air, 5% Pd cat.) were reported for the use of iminonitriles as cyanating agent (Scheme 117D).^713^ The generation of cyanide ions from these two reactants has not been yet fully understood, but it appears to occur through oxidative processes. The cyanation of indoles with NCTS was reported by Ackermann^714^ and Anbarasan^715^ using Mn/Zn and Rh catalysis respectively (Scheme 117E and F). Notable features are the functionalisation of tryptophans in the first case and the substrate scope of substituted pyrroles (often only explored as secondary substrates) in the second. Anbarasan also reported the cyanation of non-aromatic C(sp^2^)–H bonds in both cyclic and acyclic olefins using a similar procedure (Scheme 117G).^716^ This reaction was also applied to the formal synthesis of chlorpheniramine-based hystamine antagonists.

##### 15.1.1.11. Acylation

**Scheme 118:**
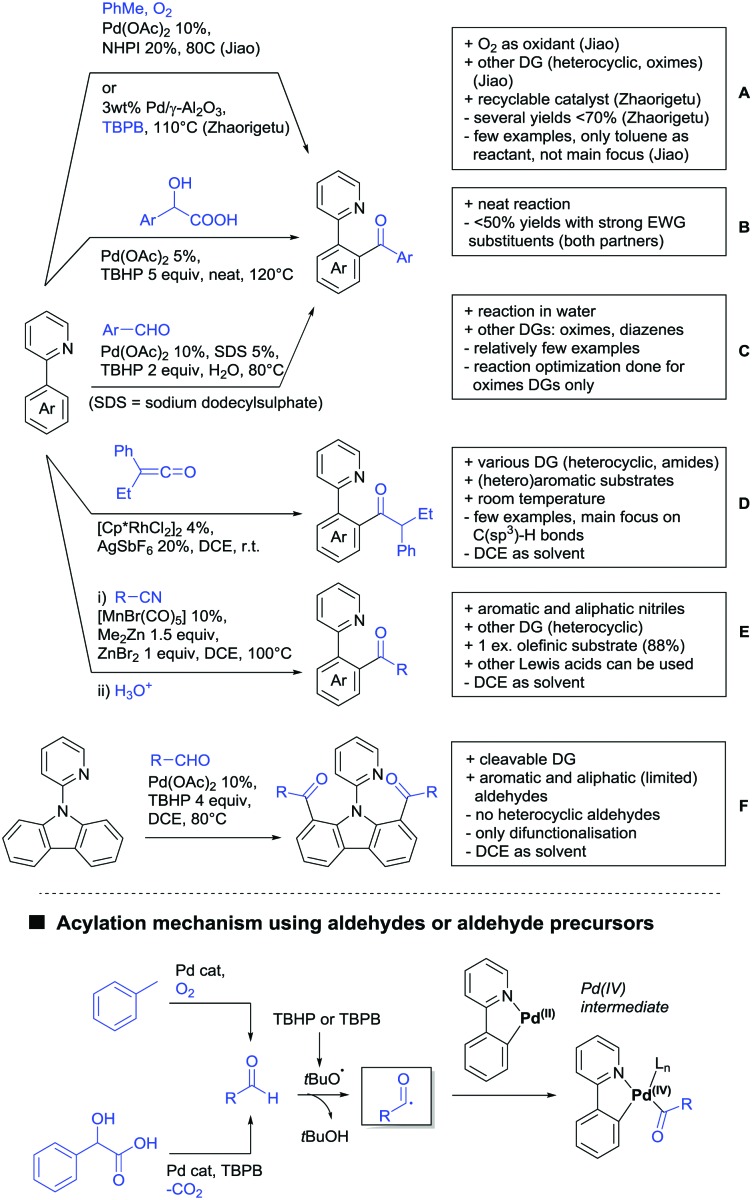
C(sp^2^)–H acylation using pyridine as DG.

This transformation is commonly achieved by using aldehydes or aldehyde precursors. An H-atom abstraction by an oxidant delivers the acyl radical, which then reacts with the cyclometallated Pd complex, giving a Pd(iv) intermediate (Scheme 118, bottom). A subsequent reductive elimination furnishes the acylated arene.

Aldehydes were generated *in situ* by aerobic oxidation of toluene to benzaldehyde in Jiao's protocol.^717^ A heterogeneous version of this reaction was reported by Zhaorigetu, using *tert*-butylperoxybenzoate (TBPB) as oxidant (Scheme 118A).^718^ A decarboxylative procedure was instead developed by Liu, using mandelic acid derivatives as reactants (Scheme 118B).^719^ The use of aldehydes themselves was reported by Deng in water, using sodium dodecylsulphate (SDS) as a phase-transfer catalyst/surfactant and *tert*-butylhydroperoxide (TBHP) as oxidant (Scheme 118C).^720^

Exceptions to the general mechanism were reported by Li and Wang, where the acylation product was obtained by formal addition to ketenes (also applied to C(sp^3^)–H bonds)^721^ and nitriles (followed by hydrolysis)^658^ using Rh and Mn catalysis respectively (Scheme 118D and E).

Following the classical protocol with aldehydes, Dash demonstrated the functionalisation of carbazoles, resulting in double acylation (Scheme 118F).^722^

##### 15.1.1.12. Alkoxy- and aminocarbonylation

**Scheme 119:**
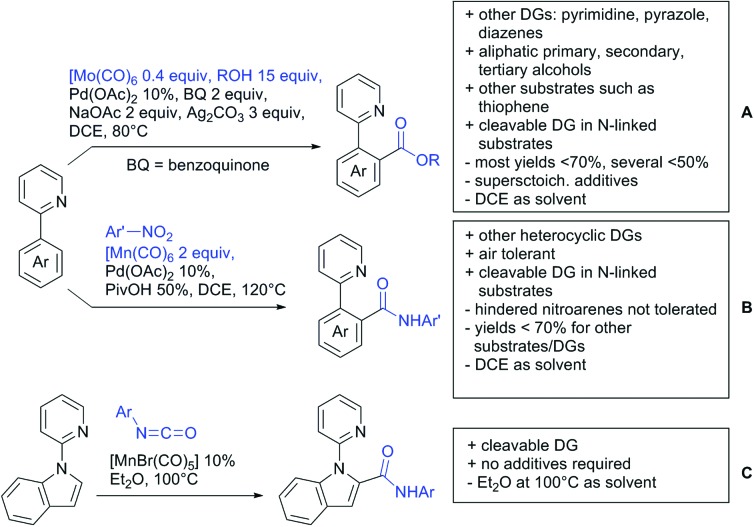
Pyridine-directed alkoxy- and aminocarbonylation.

Although other examples appeared with other DGs, only a few reports on pyridine-directed alkoxy- and aminocarbonylation were published recently. One example of the former was published by Wu in 2016, making use of Mo(CO)_6_ as CO surrogate, and requiring the superstoichiometric addition of benzoquinone (BQ), NaOAc and Ag_2_CO_3_ (Scheme 119A).^723^ This protocol worked for a variety of aliphatic alcohols, but not for other nucleophiles. The aminocarbonylation of similar substrates was recently achieved by Driver using Mn(CO)_6_ and nitroarenes (Scheme 119B).^724^ Nitrosoarenes, formed *in situ* by reduction by Mn, were identified in this case as the reactive species. In 2015 Ackermann reported a Mn-catalysed aminocarbonylation of indoles with isocyanates,^725^ one of the most commonly used reactants for this type of reactions.^726^ This reaction only required the catalyst and solvent, without the addition of any other additives or ligands (Scheme 119C).^725^

##### 15.1.1.13. C–S bond formation

**Scheme 120:**
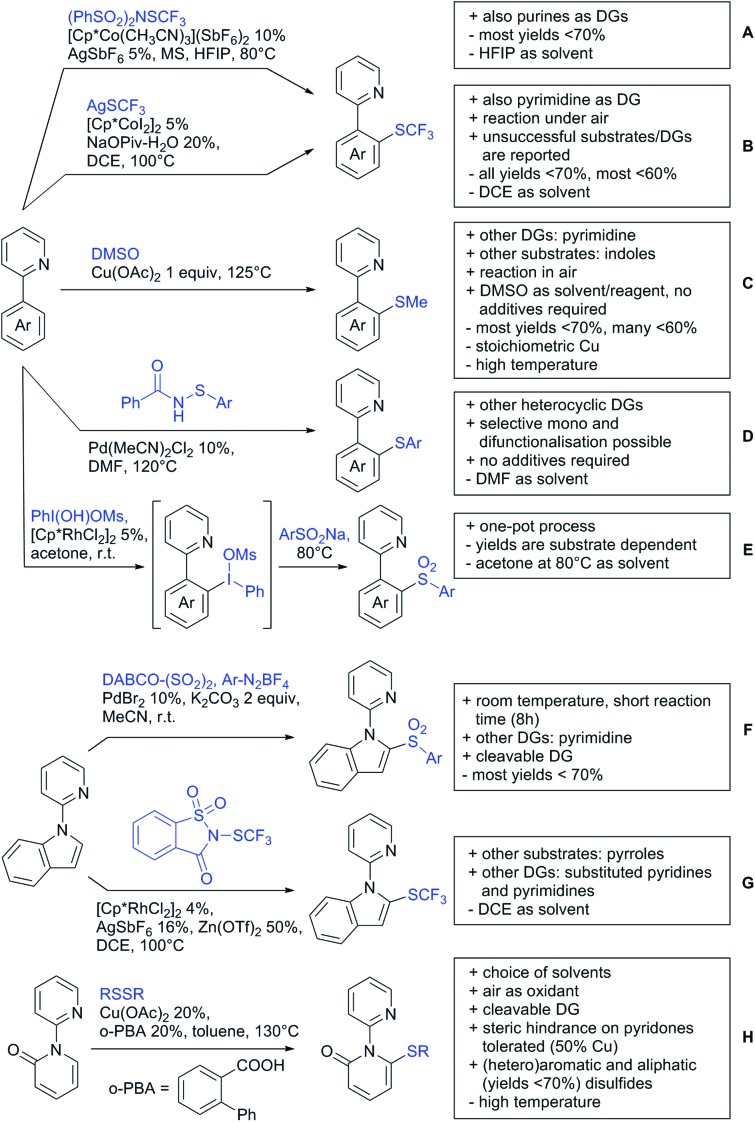
C(sp^2^)–S bond formation with pyridine DGs.

Very recently, Matsunaga^727^ and Wang^728^ reported the first protocols for Co-catalysed trifluoromethylthiolation of (hetero)aromatic substrates using (PhSO_2_)_2_NSCF_3_ and AgSCF_3_ as reactants (Scheme 120A and B). In both cases, halogenated solvents had to be used and relatively low yields (<70%) were obtained.

The use of DMSO as reactant for the methylthiolation of similar substrates was reported by Jain *via* a Cu-mediated reaction (Scheme 120C).^729^ Arylthiolation was reported by Zhang using *N*-(arylthio)benzamides,^730^ while sulphonylation can be performed *via* an Umpolung reaction with hypervalent iodine compounds, followed by reaction with sodium sulphinates^731^ (Scheme 120D and E). Notably, the latter method is also suitable for the reaction with other nucleophiles.

The introduction of arylsulfonyl functionalities on indoles was recently reported with another method, using DABCO·(SO_2_)_2_ and aryldiazonium tetrafluoroborates in a multicomponent reaction catalysed by Pd(ii) (Scheme 120F).^732^ The reaction is initiated by the formation of an arylsulfonyl radical, which is then introduced at the indole C2 position.

Trifluoromethylthiolation of indoles and pyrroles was reported by Li using *N*-(trifluoromethylthio)saccharin under Rh/Lewis acid catalysis (Scheme 120G).^733^ Interestingly, the functionalisation of 3-substituted indoles also occurred in the absence of Rh and Ag additive, suggesting that Lewis acids alone could promote the reaction on such compounds.

Finally, Liu demonstrated the aryl- and alkylthiolation of 2-pyridones with disulfides under Cu/Brønsted acid catalysis (Scheme 120H).^734^ It is worth mentioning that, although some of these reactions required an air atmosphere, an O_2_ atmosphere proved deleterious for the reaction,^728^,^729^,^734^ presumably due to sulphur oxidation in the reactants/products.

##### 15.1.1.14. C–B and C–Si bond formation

**Scheme 121:**
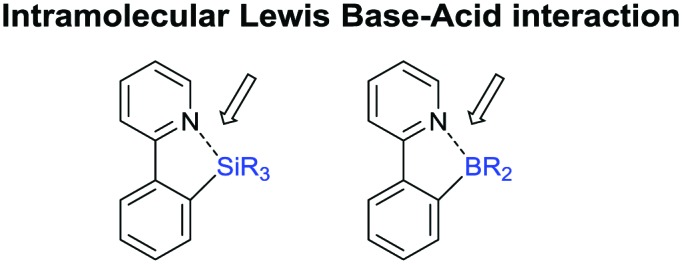
Si–N and B–N non-covalent interactions during pyridine-directed functionalisations.

Organosilanes and organoboranes have important applications in organic chemistry, for example in cross coupling reactions, and C–H borylation and silylation have therefore been considerably investigated even before 2015.^31^,^735^ Pyridine-directed C(sp^2^)–H borylation and silylation have been reported by a number of research groups. The compounds obtained in this way, which often contain an internal Lewis acid–base interaction as shown in Scheme 121, also have applications in material science, due to their fluorescent properties.^736^–^739^ Due to these non-covalent interactions, the C–H functionalisation process itself has been suggested to occur with two main different mechanisms. In the first one, the reaction is initiated by the C–H cleavage/formation of a DG-coordinated metallacycle (metal = catalyst, as for most of group-directed functionalisations), followed by insertion of the boryl/silyl group.^740^–^743^ The other mechanism assumes the Lewis acid–base interaction as the initial step, followed by catalyst coordination/C–H cleavage, on the basis of non-successful metallacycle formation or to early stage Lewis acid–base interactions observed spectroscopically.^739^,^744^

**Scheme 122:**
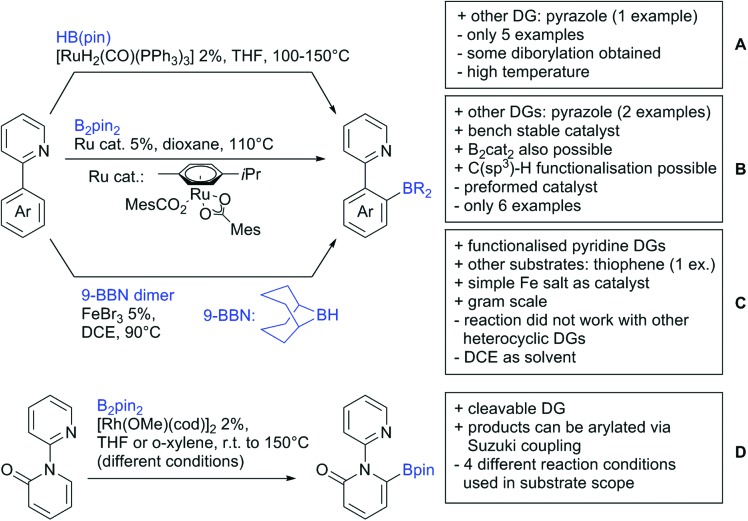
Pyridine-directed borylation of C(sp^2^) centres.

Borylation employs diboranes or boranes as reactants; in the latter case, the addition of a hydrogen acceptor (generally an olefinic compound) to promote the reaction (as commonly used for silylation with hydrosilanes, *vide infra*) might instead result in complete inhibition due to hydroboration of the unsaturated moiety.^739^ Several examples of borylation of 2-phenylpyridines and analogues have appeared in the recent literature. Murata and Ackermann reported the use of HBpin or B_2_pin_2_ in combination with Ru catalysts (Scheme 122A and B),^740^,^742^ however only a few examples of C(sp^2^)–H borylation were shown (Ackermann's protocol was nonetheless also applied for C(sp^3^)–H borylation, see Scheme 136).

Kuninobu reported an interesting Fe-catalysed protocol using 9-BBN (9-borabicyclo[3.3.1]nonane) as borylating agent (Scheme 122C),^739^ and Miura/Hirano disclosed a Rh-catalysed borylation of 2-pyridones (Scheme 122D).^741^ The use of the reaction products as substrates in a Suzuki coupling nicely demonstrates the potential for applications in synthesis.

**Scheme 123:**
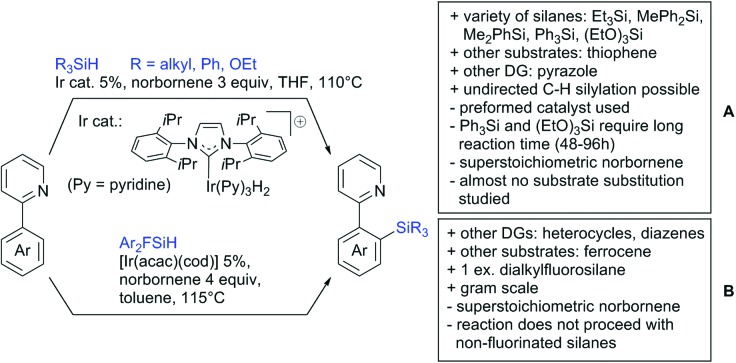
Pyridine-directed silylation of C(sp^2^) centres.

Typical reactants for silylation are disilanes or hydrosilanes, the latter generally requiring the addition of a hydrogen acceptor to be efficient (norbornene is often used for this role, in superstoichiometric amounts).^735^ Hydrosilanes/norbornene combinations have been used for the Ir-catalysed silylation of 2-arylpyridines by Oro/Iglesias and Kuninobu/Kanai (Scheme 123A and B).^743^,^744^ In the first case a preformed NHC–Ir(iii) hydride complexes was used as catalyst for the silylation of 2-phenyl- and 2-thienylpyridines with a variety of silanes, ranging from triethyl to triphenyl and trialkoxy silanes. Interestingly, the catalyst also proved efficient for a variety of undirected C(sp^2^)–H silylations on (hetero)arenes.^743^ Kuninobu/Kanai's protocol was developed instead for diarylfluorosilanes, and was not effective for non-fluorinated reactants.^744^

##### 15.1.1.15. C–N bond formation

###### 15.1.1.15.1. Amidation

**Scheme 124:**
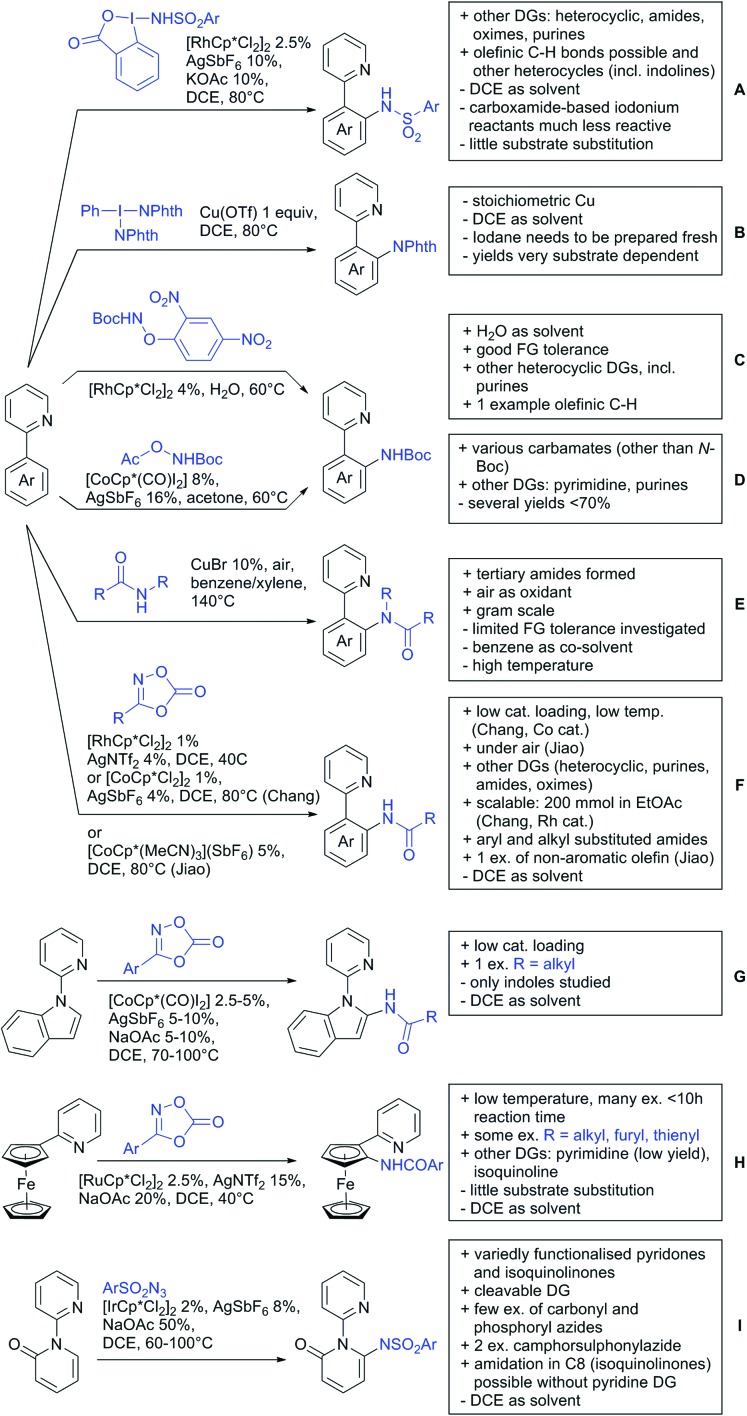
Amidation reactions with pyridine DGs.

While until 2014 a number of procedures were reported for C–H amidation using acyl azides as nitrene precursors, recently a number of different reactants have been employed for this transformation. Reactions with aminated hypervalent iodine reactants have been reported by Loh and DeBoef using catalytic Rh and stoichiometric Cu respectively, for the synthesis of *N*-aryl sulfonamides and phthalimides (Scheme 124A and B).^745^,^746^ A reaction with the electrophilic nitrogen source *N*-Boc-*O*-2,4-dinitrophenylhydroxylamine in water under Rh catalysis was developed by Lu (Scheme 124C).^747^ Notable features of this procedure are: the use of water as solvent, the good FG tolerance, and the possibility of functionalisation of purine-based nucleosides. Chang used a similar hydroxylamine reactant, *N*-Boc-*O*-acetylhydroxylamine, in the Co-catalysed amidation of arenes: in this case a variety of carbamates (other than *N*-Boc) could be employed in the reaction, and purines could be used as DG (Scheme 124D).^748^

The Cu-catalysed dehydrogenative C–H amidation with secondary amides has been reported by Chen using air as the sole oxidant (Scheme 124E).^749^ Here the formation of tertiary amides is remarkable, as most protocols result in the formation of secondary amides instead.

In 2015 Chang introduced dioxazolones as reactants for C–H amidation. Dioxazolones are safer and more reactive than the corresponding acyl azides, due to their relative stability and stronger coordination to the metal centre, which promotes faster reactions. Moreover, these reactants are simple to prepare and to handle. Chang^750^–^752^ and Jiao^753^ reported their use in the directed amidation of arenes using Rh and Co catalysts, showing the reaction to be suitable for a number of different DGs (including purines, amides, and oximes, Scheme 124F). Notably, Chang reported the use of EtOAc as alternative solvent for a 200 mmol scale reaction.^751^ Ackermann subsequently applied a similar procedure to the Co-catalysed amidation of indoles,^754^ while You employed Rh for the functionalisation of ferrocenes^755^ (Scheme 124G and H).

Very recently, the amidation of pyridones (C6) and isoquinolinones (C3) was reported with sulphonyl azides under Ir catalysis (Scheme 124I).^756^ Interestingly, C8-functionalisation of isoquinolinones could be performed in similar conditions in the absence of the heterocyclic DG.

###### 15.1.1.15.2. Amination

**Scheme 125:**
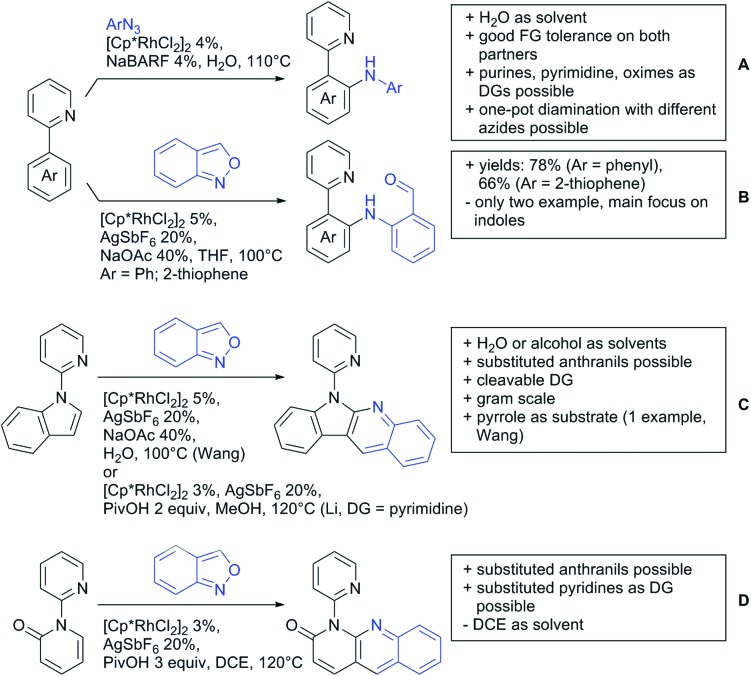
Amination and related reactions with pyridine DGs.

C–H amination reactions also typically involved nitrenes obtained *in situ* from azides. For example, Lu demonstrated the reaction of 2-phenylpyridines with aryl azides in water, also allowing for a double *ortho*-functionalisation with two different azides (Scheme 125A).^757^ The use of anthranils (2,1-benzisoxazoles) as alternative, more stable nitrene precursors was independently reported by Wang and Li.^758^,^759^ The reaction of indoles with anthranils using polar protic solvents (H_2_O or MeOH) and Rh catalysis resulted in a tandem amination–cyclisation process, leading to the formation of indoloquinolines (Scheme 125C).^758^,^759^ The same reaction was also shown to occur on 2-pyridones (Scheme 125D).^759^ Interestingly, the cyclisation did not occur when phenyl or thienyl substrates were investigated, and the reaction only resulted in the corresponding *o*-formylaniline-substituted product (Scheme 125B).^758^

###### 15.1.1.15.3. Azidation

**Scheme 126:**
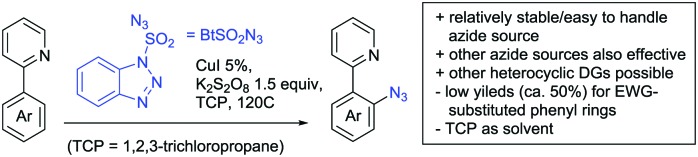
Azidation of aranes using pyridine as DG.

Narula reported in 2015 a protocol for the Cu-catalysed azidation of 2-phenylpyridines using benzotriazole sulfonyl azide (BtSO_2_N_3_) and K_2_S_2_O_8_ as oxidant (Scheme 126).^760^ NaN_3_ and 1-imidazole sulfonyl azide (ImSO_2_N_3_) also proved effective in these conditions.

##### 15.1.1.16. C–O bond formation

Examples of C–O bond formation are somewhat limited in comparison to C–C and other C–heteroatom bond forming procedures, with yields often <70% or even <50%, possibly due to the strong coordinating ability of O, which might inhibit the catalysis by preventing reductive elimination or forming relatively stable complexes.^761^,^762^ Nonetheless, interesting advances are being made in terms of greener oxidants, cheaper base metal catalysts, and milder reaction conditions.

A number of papers on pyridine-directed C–O bond formation reactions on (hetero)arenes have been reported using Ru/PIDA,^761^ solid-supported Pd/PIDA^763^,^764^ or simple Pd salts/peroxides^765^,^766^ combinations, leading to *ortho* acetoxylated, hydroxylated or alkoxylated compounds (in case of PIDA as oxidant, when H_2_O or an alcohol are used as solvent, one-pot hydrolysis or alcoholysis of the acetoxylated product is obtained).

**Scheme 127:**
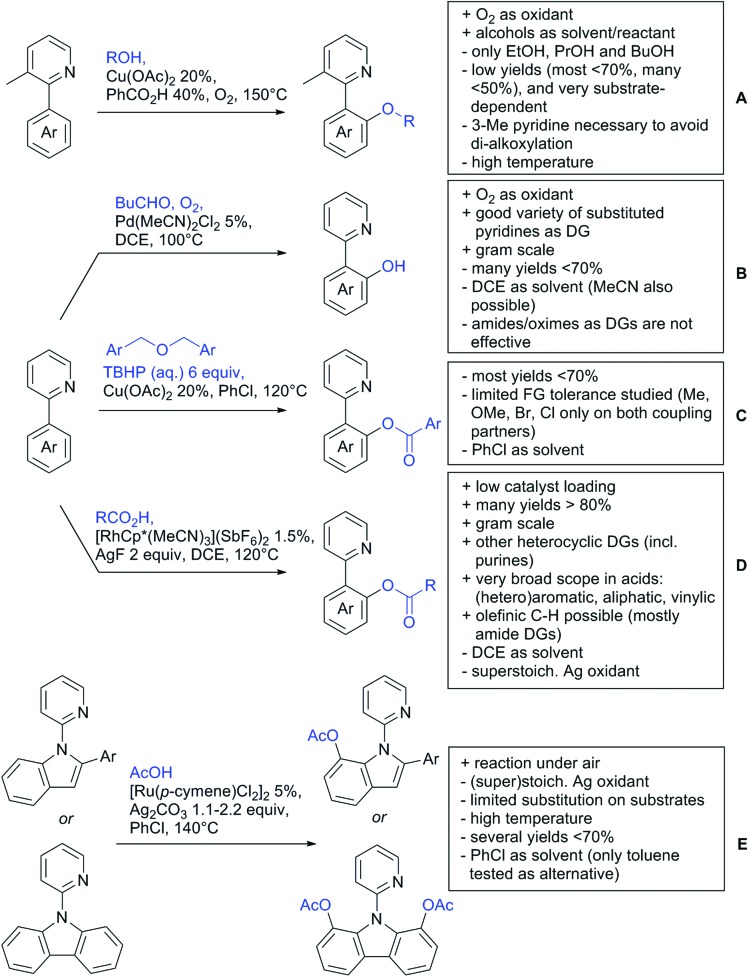
Pyridine-directed C(sp^2^)–O bond formation.

In the past few years, some reports appeared where O_2_ was used as oxidant. Shen reported a direct alkoxylation with alcohols (only 3-methylpyridine could be used as DG, Scheme 127A),^767^ while Guis used aldehydes/O_2_ as reagents in combination with Pd catalysis.^768^ In this case the reaction occurred *via* formation of an intermediate acyl peroxo-radical, followed by its reaction with the arene, ultimately resulting in hydroxylated compounds (Scheme 127B). Patel disclosed the benzoxylation of arenes using benzylethers and TBHP as oxidant under Cu catalysis (Scheme 127C).^769^ Although limited in yields and scope, this protocol is interesting as it shows different product selectivity from its Pd-catalysed counterpart (which gives acylated products).^770^ The formation of the ester product from the benzylic ether derives from Cu/TBHP mediated oxygenation to benzaldehyde and further to *t*-butyl arylperoxyester. The arylcarboxy radical generated by homolitic cleavage of this intermediate is finally introduced at the *ortho* position in the substrate.^769^ A very recent remarkable paper by Li and Xu demonstrated the acyloxylation of 2-phenylpyridines and analogues with carboxylic acids and Rh catalysis (Scheme 127D).^234^ Though using a superstoichiometric amount of Ag as oxidant, this protocol was applicable to a broad range of carboxylic acids: aliphatic (incl. saturated heterocyclic rings), (hetero)aromatic and vinylic reacted in good yields, often >80%. Moreover, a range of DGs proved effective in this protocol, and amides were used for the acylation of olefinic substrates, although in modest yields (<70%).^234^

Finally, the acetoxylation of indoles (2-substituted, C7 functionalisation) and carbazoles was reported by Miura using acetic acid, Ru catalysis, and Ag oxidant (Scheme 127E).^771^ It is worth noting that the functionalisation of unsubstituted indoles was not observed, either in C7 or in C2.

##### 15.1.1.17. C–Halogen bond formation

**Scheme 128:**
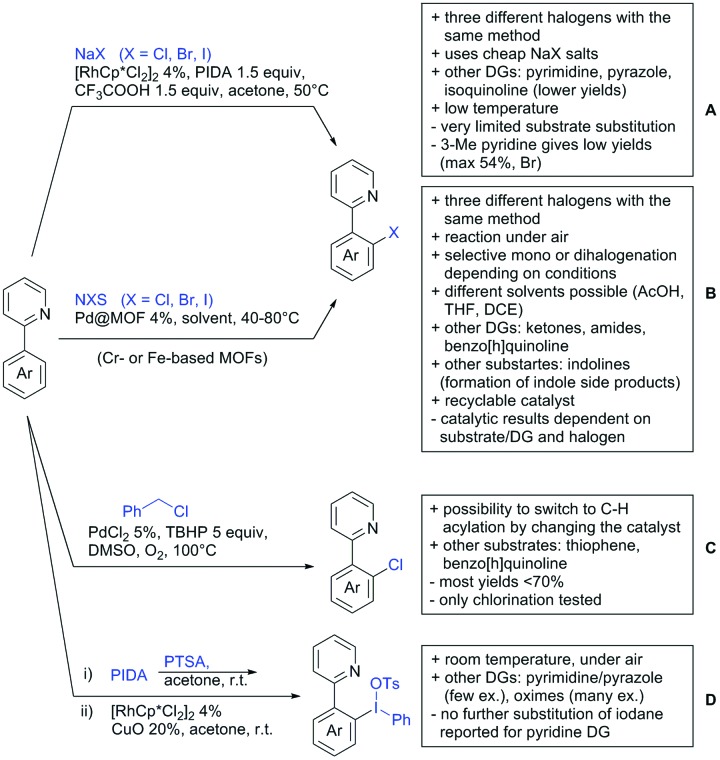
Pyridine-directed C(sp^2^)–H halogenation reactions.

C–H halogenation, as for arylation reactions, also suffered from the formation of mixtures of mono and di-halogenated compounds. In recent years, a few synthetic methods showing good selectivity were reported by a number of research groups and in different conditions. The use of simple and cheap NaX salts as reactants for the introduction of Cl, Br and I reported by Wang in 2015 is certainly a landmark (Scheme 128A).^772^ This method is the most atom economic reaction available in the series, although a limited substrate scope was investigated. The use of *N*-halosuccinimides as halogenating reactants using Pd supported on Cr- or Fe-based MOFS as heterogeneous catalysts was investigated by Martin-Matute (Scheme 128B).^773^ Although having a number of clear advantages, such as mild conditions and recyclability of the catalyst, the catalytic results are unfortunately considerably dependent on the substrate and DG employed, presumably due to the steric hindrance of the starting materials and the rigid structure of the MOFs. It is worth mentioning that, although the catalysts could be reused for several runs, some degradation of the solid structures was observed, as well as its halogenation as a side process. Similar reactions, using Pd@CNT (carbon nanotubes)^763^ or other Pd@MOFs (Zr-based)^774^ were reported by Ellis and Cohen.

Wu reported the use of benzyl chloride as halogenating agent under oxidising conditions (Scheme 128C).^775^ While using PdCl_2_ as catalyst, and under O_2_ atmosphere, selective C–Cl bond formation was obtained, switching the catalyst to a preformed palladacycle with a ferrocenylimine ligand and adding a base under air, resulted in the selective acylation of the C–H bond (not shown).^775^ In the chlorination case, the intermediate *t*BuOCl is suggested as actual reactant, while in the second case the formation of an acyl radical is responsible for the reaction. In 2015 Li reported the synthesis of diaryliodonium salts starting from PIDA, PTSA (*p*-toluensulfonic acid) and a DG-equipped arene under Rh catalysis (Scheme 128D).^776^ These compounds can be used as intermediates for the formation of C–C, C–O, C–N, C–S and C–P bonds using a variety of nucleophiles (see also Scheme 120E). It is worth noting, however, that many procedures have been reported for the direct formation of such bonds with the same DGs, and the synthetic utility of this two-step procedure is therefore rather limited.

#### 15.1.2. C(sp^3^)–H bond functionalisation

The functionalisation of activated C(sp^3^)–H bonds, such as benzylic, allylic, or in α to a heteroatom, has been much investigated, even without the facilitating effect of a DG. The activation and functionalisation of unactivated C(sp^3^)–H bonds is instead considerably more difficult, due to the intrinsically stronger bonds, and selectivity is also challenging due to the presence of several similar bonds in a typical organic molecule. Group-directed C(sp^3^)–H bond functionalisation has been explored during the last two decades, and brought important improvement in the field. A plethora of effective transformations has been reported throughout the years. Although mostly bidentate DGs have been used for the functionalisation of C(sp^3^)–H bonds in the last few years,^777^,^778^ several examples employing monodentate DGs, including heterocycles, have appeared, after the pioneering work of Sanford,^629^,^779^ Chatani,^780^ and Daugulis,^630^ among others. For the azine-based DGs, the substrates investigated fall mainly in two classes: 8-methylquinoline and 2-ethylpyridine derivatives, which will be treated in this section.

8-Methylquinolines are the most common substrate investigated, and its functionalisation is generally easier than that of 2-ethylpyridines, due to its benzylic nature. Typically, substitution on the aromatic rings is tolerated, although in some instances strong EWG substituents and steric hincrance in C7 or C2 can inhibit the reaction to various degrees. In general, apart from a few cases, substitution at the benzylic position (*e.g.* 8-ethylquinoline) strongly suppresses C–H activation.

2-Ethylpyridines (and in general any other flexible substrate), on the other hand, present the intrinsic issue of the flexibility of the alkyl chain, which makes the coordinating ability of the azine less efficient (metallacycle formation). In such substrates, monofunctionalisation may also pose some challenges, due to the lack of steric hindrance in close proximity. These issues are generally tackled by using branched alkyl chains instead of linear ones, to reduce flexibility/rotation (Thorpe–Ingold effect) and increase the steric hindrance around the desired C–H bond.

As C–C bonds connect the azine to the functionalisable C–H bonds, the DG is not cleavable in such substrates. Quinoline substrates can, however, be easily reduced to tetrahydroquinoline with NiCl_2_/NaBH_4_,^781^–^783^ H_2_/Pd@C,^721^ H_2_/PtO_2_,^784^ or NaBH(CN)_3_/BF_3_–Et_2_O,^785^ hereby enlarging the application scope. The same reduction is in theory possible for 2-ethylpyridine derivatives, to produce piperidines, but it has not been used in the context of C–H functionalisation.

##### 15.1.2.1. Arylation

**Scheme 129:**
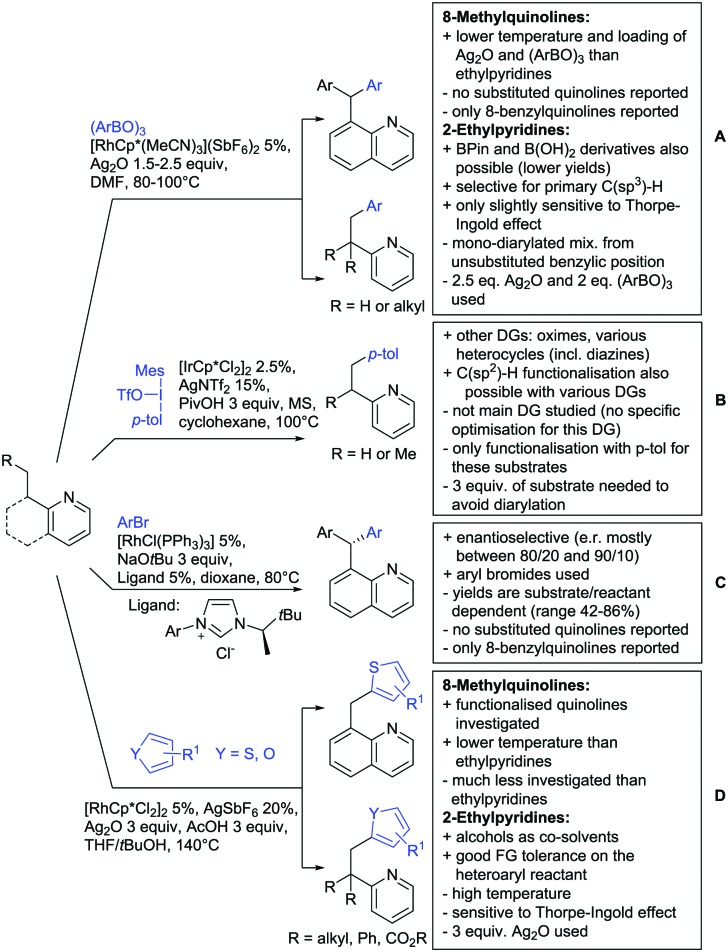
Arylation of 2-ethylpyridines and 8-methylquinoline derivatives.

A few arylation procedures have been reported by Glorius, Shi/Xia and You under Rh/Ir catalysis, for the functionalisation of both 8-methylquinolines and 2-ethylpyridines. In 2015 Glorius reported the Rh-catalysed arylation of both substrates with aryl boroxines (Scheme 129A).^786^ Despite the use of up to 2 equiv. of boroxine (6 equiv. Ar) and superstoichiometric Ag as oxidant (due to boroxine homocoupling as a side reaction) the reaction gave good yields for both substrates. While only 8-benzylquinoline was functionalised for this class of substrates, both branched and linear 2-ethylpyridine analogues were tested in the reaction: branched substrates reacted to give selectively monoarylated products, while linear-chain substrates resulted in varying amounts of diarylated product.^786^

The arylation of 2-ethylpyridines with diaryliodonium salts was reported by Shi under Ir catalysis (Scheme 129B).^787^ This reaction, although requiring 3 equiv. of the substrate to avoid difunctionalisation, works well with a variety of azines (pyridines, pyrazines, quinolines, 73–95% yield) and azoles (pyrazole, isoxazole, 45–52%). The possibility to introduce aromatic rings other than *p*-tolyl was demonstrated using aliphatic oximes as substrates.^787^ In 2016 Glorius reported another protocol for the arylation of 8-benzylquinolines, this time with aryl bromides (Scheme 129C).^788^ The use of a chiral carbene ligand allowed the formation of enantio-enriched products (e.r. between 80/20 and 90/10). This reaction proved to be fairly substrate sensitive, with yields ranging between 42 and 86%, depending on the substituent on the benzylic ring of the substrate and the aryl bromide used.^788^

Finally, an interesting protocol from the You group specifically aimed at the heteroarylation of 2-ethylpyridines and 8-methylquinolines appeared in 2017 (Scheme 129D).^789^ Apart from the specific functionalisation with substituted thiophenes, benzothiophenes and furans, this is a challenging dehydrogenative coupling between a C(sp^3^)–H and a C(sp^2^)–H bond. Whereas the high temperature (140 °C), the 3 equiv. of oxidant (6 equiv. Ag overall) required, and the sensitivity to the Thorpe–Ingold effect can be seen as drawbacks, very notable features are: the use of alcohols as cosolvents for 2-ethylpyridines, the use of substituted quinolines as DGs, and the FG tolerance on the heteroarene partner, which includes esters, amides, aldehydes, halides and alkenes, furnishing yields in most cases >60%.^789^

##### 15.1.2.2. Alkylation

**Scheme 130:**
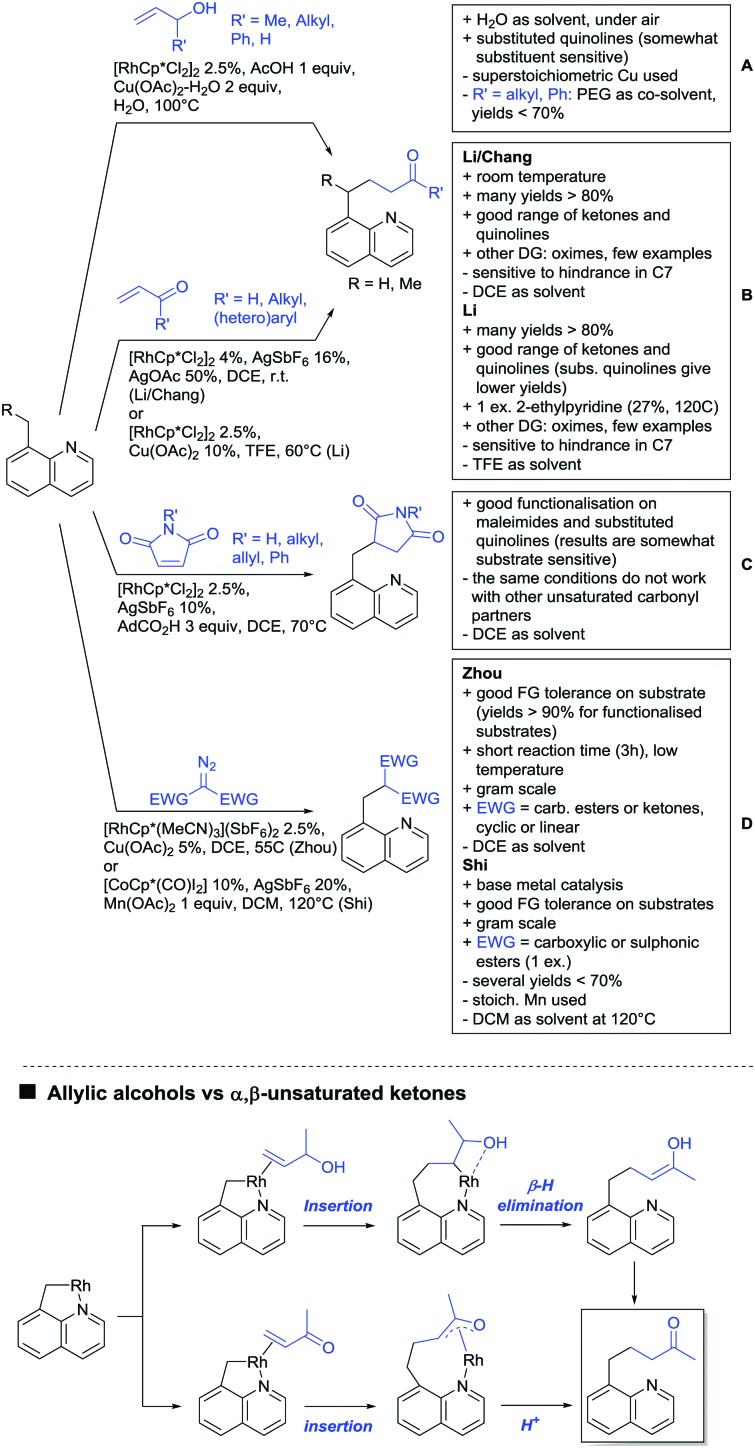
Alkylation of 8-methylquinoline derivatives.

For alkylation, 8-methylquinoline is the substrate of choice and, to our knowledge, no alkylation on 2-ethylpyridine derivatives have appeared in the last 3 years. Investigations using typical reactants such as α,β-unsaturated carbonyls and diazocompounds have been undertaken. A notable exception is the use of cyclopropanols as alkylating agents, reported by Li.^790^ In this case only two examples of C(sp^3^)–H alkylation were reported, thus this method is not covered in Scheme 130. Another interesting exception is the use of allylic alcohols as alkylating agents, reported in 2017 by Kim (Scheme 130A).^783^ Apart from the new reactant used, this reaction occurs in water under air and a range of substituted quinolines could be functionalised in good yields. The use of alcohols other than 3-buten-2-ol required a mixture H_2_O/PEG (polyethylene glycol) as solvent, and resulted in generally lower yields (<70%).^783^

Two very similar protocols for the alkylation with α,β-unsaturated ketones were reported by Li/Chang and Li (Scheme 130B).^781^,^791^ Both protocols, using Rh catalysis in halogenated solvents at low/mild temperatures gave generally high yields of the desired alkylated quinolines. Somewhat lower yields were obtained with functionalised substrates, in particular sensitivity to steric hindrance in C7 was noted in both cases. The reactions proceeded well with aliphatic and (hetero)aromatic vinyl ketones.^781^,^791^ The formation of the same product starting from both vinyl alcohols and α,β-unsaturated ketones is due to different mechanisms. For the latter, the formal 1,4-addition product is obtained by migratory insertion of the double bond into the initial rhodacycle, followed by hydrolysis (protodemetallation). In the case of allylic alcohols, a migratory insertion/β-hydride elimination is the suggested pathway to the alkylated final products (Scheme 130, bottom).

The use of maleimides as coupling partners has been reported by Kim in 2016, leading to succinimide-functionalised methylquinolines (Scheme 130C).^782^ Although the reported conditions did not prove effective with other α,β-unsaturated carbonyl partners, the reaction proceeded in good yields with a range of *N*-functionalised maleimides and substituted quinolines (strong EWG substituents gave considerably low yields).

Two papers appeared recently on the alkylation of 8-methylquinolines making use of diazocompounds, one under Rh and the other under Co catalysis (Scheme 130D).^784^,^785^ Both protocols are characterised by a good FG tolerance on the quinoline substrate; for instance, strong EWG substituents can still lead to high yields in the Rh-catalysed process,^784^ while a variety of useful functionalisations are tolerated in the Co-catalysed process, such as alkenes, alkynes, free NH_2_ and Bpin.^785^

##### 15.1.2.3. Alkenylation and alkynylation

**Scheme 131:**
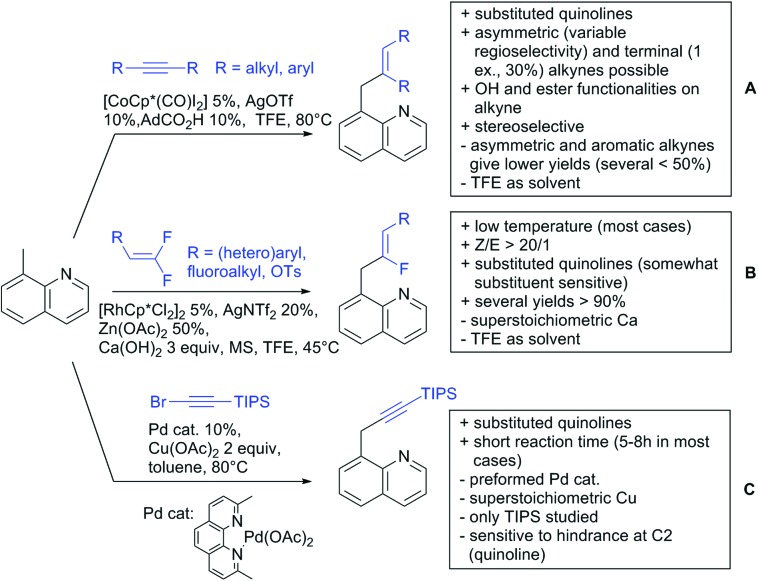
Olefination and alkynylation of 8-methylquinolines.

Due to inherently poor reactivity and selectivity, olefination of C(sp^3^)–H bond is in general rare, and only a few papers have appeared in the literature specifically on the introduction of these functionalities on 8-methylquinolines. Sundararaju reported a Co-catalysed addition to internal alkynes, generating olefins in a stereoselective (*syn*) manner (Scheme 131A).^792^ The reaction with asymmetric alkynes resulted in varying degrees of regioselectivity, depending on the substituents. Interestingly, one example of addition to a terminal alkyne was also reported, albeit in 30% yield.

The reaction with geminal difluoroalkenes (F as leaving group) under Rh catalysis was developed by Li and Wang, and also occurred with good stereoselectivity for a variety of substituted 8-methylquinolines (Scheme 131B).^793^ Interestingly, a few examples of heteroaromatic (32–35% yield) and fluoroalkyl-substituted (94%) alkenes were demonstrated. Surprisingly, it was also shown that fluorine can act as a leaving group even in the presence of a tosylate group, which remained unaltered in the product.^793^

Only one example of alkynylation of 8-methylquinolines has been reported recently (Scheme 131C).^794^ A preformed Pd(ii) catalyst was utilised for the coupling with TIPS-substituted acetylene bromide. A range of differently substituted quinolines (including alkene and ester substituents) could be coupled efficiently under these conditions. The Cu(OAc)_2_ in superstoichiometric amounts, despite being mentioned as oxidant, probably has another role, as no oxidant should be required for this transformation.

##### 15.1.2.4. Acylation

**Scheme 132:**
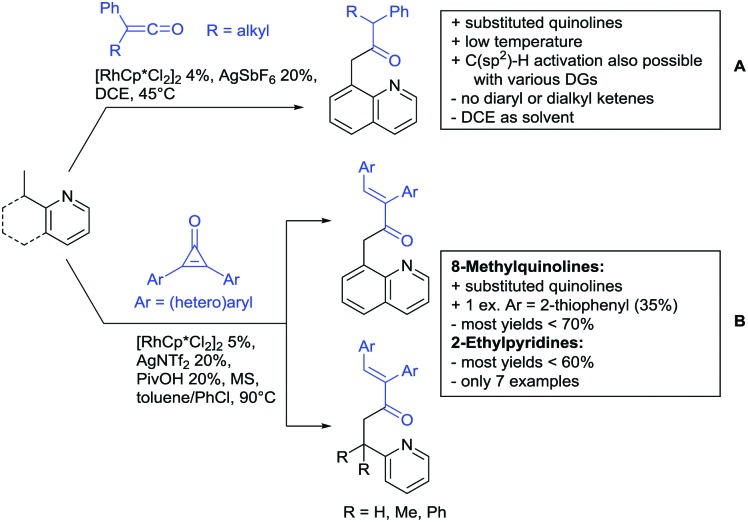
Acylation of 8-methylquinolines and 2-ethylpyridines.

Li reported two protocols for the C(sp^3^)–H acylation of 8-methylquinolines and 2-ethylpyridines, using relatively reactive ketenes or cyclopropenones with Rh catalysts (Scheme 132A and B).^721^,^795^ In both protocols substituted 8-methylquinolines were well tolerated, even at the C7 position. The acylation with cyclopropenones, although not particularly high-yielding, allows the introduction of enones with a single reactant, and can also be applied to 2-ethylpyridine derivatives.

##### 15.1.2.5. C–N bond formation

A relatively large number of examples of C(sp^3^)–N bond formation in 2-ethylpyridine or 8-methylquinoline derivatives have recently appeared in the literature. With a few exceptions, these mostly occur under Rh catalysis, using dioxazolones, amides, azides, hypervalent iodine compounds (amidation), anthranils (amination), and organic nitrites (nitration) as reactants.

###### 15.1.2.5.1. Amidation

Amidation reactions have been studied with a range of nitrene precursors, such as azides, dioxazolones, and amidated hypervalent iodine compounds.

**Scheme 133:**
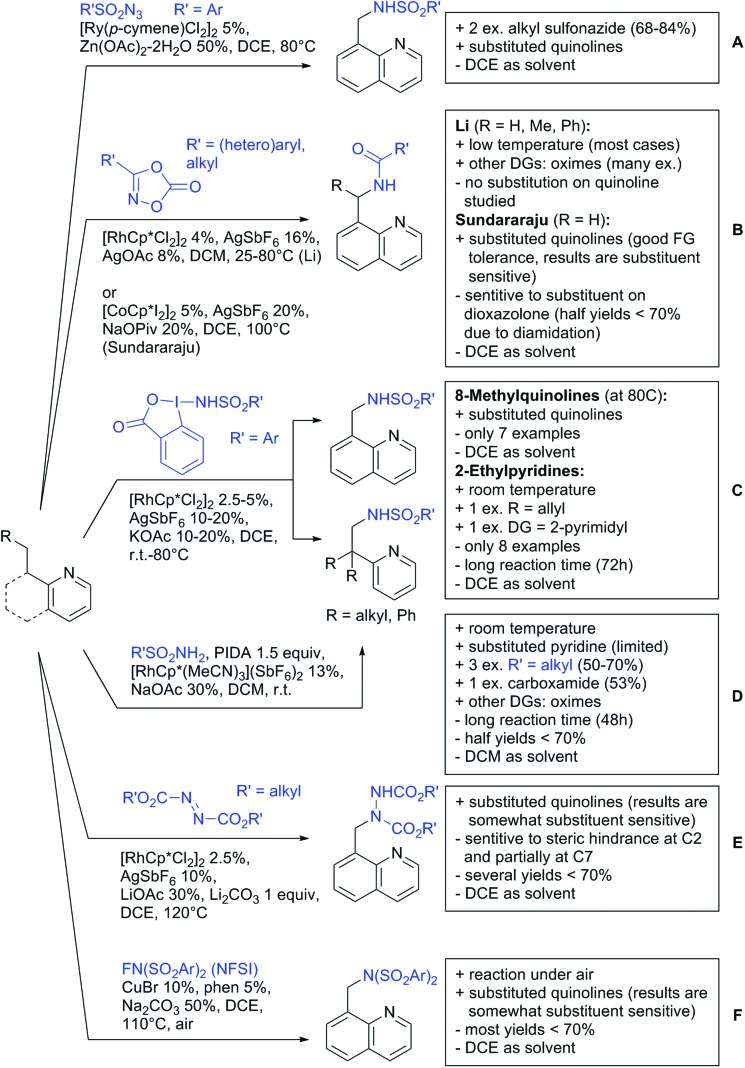
Directed amidation on 8-methylquinoline and 2-ethylpyridine derivatives.

The reaction with sulfonazides works efficiently with a variety of substituted coupling partners (Scheme 133A).^796^ Notably, EWG substituents on the quinoline provided better results than EDGs, which rarely occurs. Li and Sundararaju employed dioxazolones for the amidation, using respectively Rh and Co dimers as precatalysts (Scheme 133B).^797^,^798^ Importantly, the reaction with Rh was demonstrated to occur even on 8-ethyl or 8-benzylquinolines, although substitution on the quinoline itself was not investigated. Notably, many examples of C(sp^3^)–H amidation, including more complex molecules, were reported with this protocol making use of oximes ad DGs.^797^ The protocol under Co catalysis, although being fairly sensitive to the substitution pattern in both coupling partners,^798^ is one of the rare examples of base metal-catalysed C(sp^3^)–H activation, and is therefore an important advancement.

The use of amidated iodanes, either preformed or formed *in situ*, for the functionalisation of both C(sp^2^)–H and C(sp^3^)–H bonds was reported by Loh and You respectively under Rh catalysis (Scheme 133C and D).^745^,^799^ In particular, the amidation of 2-ethylpyridine derivatives can be performed at room temperature, although requiring a long reaction time (48–72 h). Interestingly, this functionalisation seems to require a quaternary carbon α to the functionalised C–H bond to be effective (Scheme 133C bottom and Scheme 133D). A couple of other procedures have been reported for the amidation of 8-methylquinoline, which proceed *via* a different mechanism (no nitrenes involved). Azodicarboxylates have been used by Kim and Park, while *N*-fluoroarenesulfonimides were utilised by Zheng under Cu catalysis (Scheme 133E and F).^800^,^801^ In both cases certain sensitivity to the substitution patterns on the quinoline was observed, resulting in some cases in poor yields.

###### 15.1.2.5.2. Amination and nitration

**Scheme 134:**
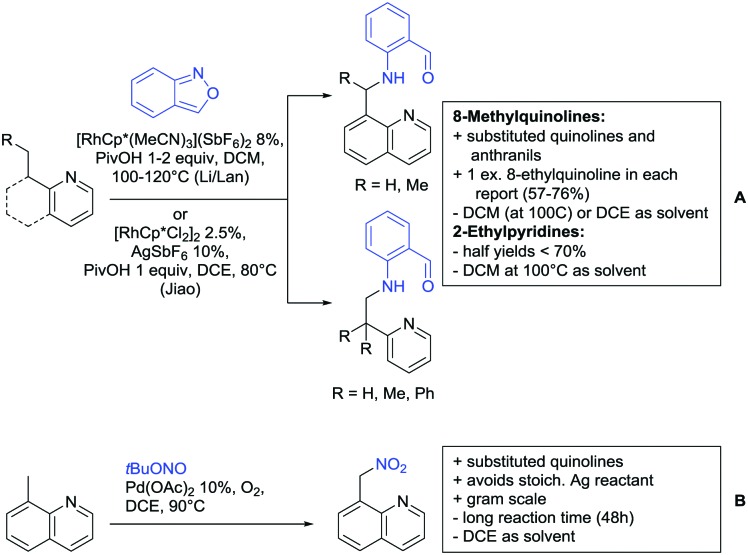
Directed amination and nitration on 8-methylquinoline and 2-ethylpyridine derivatives.

Jiao and Li/Lan reported the amination of 8-methylquinolines and 2-ethylpyridines using anthranils under very similar conditions (Scheme 134A).^802^,^803^ A notable point, apart from various functionalities tolerated on both coupling partners, is the possibility to employ 8-ethylquinolines as substrates, which are generally not reactive.

The use of *t*-butylnitrite in combination with oxygen and a Pd catalyst for C(sp^3^)–H nitration was reported in 2015 by Liu (Scheme 134B).^804^ This protocol is interesting as it avoids the use of stoichiometric AgNO_2_ as reactant, often used in C–H nitration. Although proceeding relatively slowly (48 h reactions), variedly substituted 8-methylquinolines, including strong EWG-substituted, could be functionalised in good yields.

##### 15.1.2.6. C–O bond formation

**Scheme 135:**
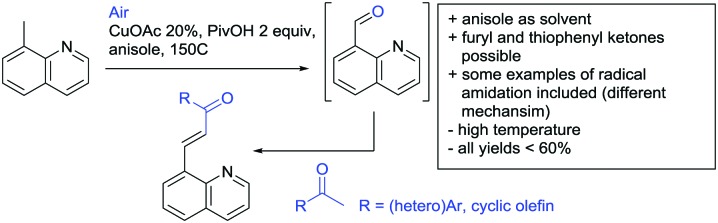
C–O bond formation on 8-methylquinolines.

C(sp^3^)–O bond formation for this class of DG has not been much investigated. Apart from two isolated examples of methoxylation and acetoxylation of 8-methylquinoline (99 and 90% respectively) under heterogeneous Pd catalysis reported by Ellis in 2015,^763^ Chan demonstrated the coupling of 8-methylquinolines with methylketones catalysed by Cu.^805^ The proposed mechanism for this reaction does not actually involve coordination of the quinoline nitrogen to Cu and is therefore not entirely within the scope of this review. Nonetheless, it has been described in Scheme 135 for completeness. The reaction seems to be initiated by an acid-promoted aerobic oxidation, similar to a reaction developed in the Maes group,^806^,^807^ leading to the corresponding quinoline aldehyde. A subsequent (cascade) condensation with the methylketone results then in the formation of the enone.^805^

##### 15.1.2.7. C–B and C–Si bond formation

**Scheme 136:**
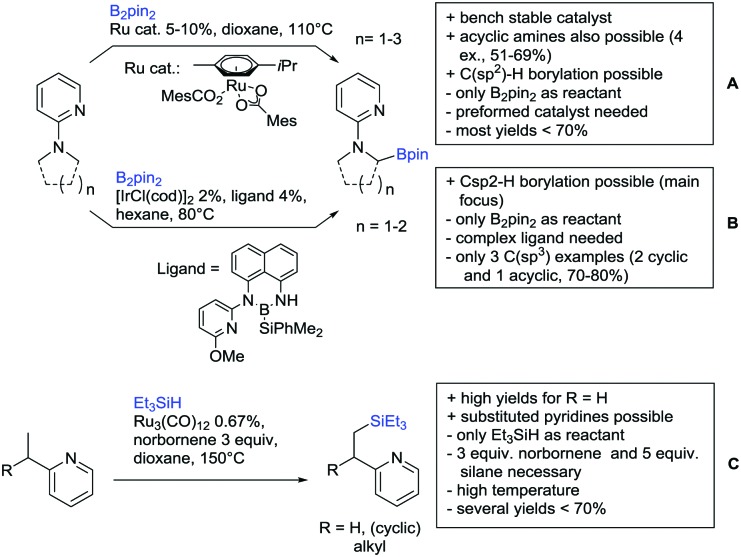
C(sp^3^)–H borylation and silylation.

Few examples have been reported of C(sp^3^)–B bond formation, investigating cyclic and acyclic amines, rather than the typical 2-ethylpyridines and 8-methylquinolines.^740^,^808^ The functionalisation of cyclic amines is an interesting topic, and has been relatively little explored.^809^ The borylation of such compounds are therefore of interest. Ackermann's protocol utilises a preformed, bench-stable Ru–carboxylate catalyst, while Ir catalysis and a complex pre-ligand were used by Li (Scheme 136A and B).^740^,^808^ Although higher yields were obtained in this case, only three examples of C(sp^3^)–H functionalisation were shown, with the main focus of the paper being C(sp^2^)–H functionalisation with a number of different DGs.

C(sp^3^)–Si bond formation has been investigated instead on 2-ethylpyridine derivatives (Scheme 136C).^810^ Despite clear disadvantages such as the use of 5 equivalents of silane and 3 of norbornene as additive (hydrogen acceptor), and the high temperature required, the process gave good yields for a variety of substrates, including substituted pyridines and unsubstituted alkyl chains, using a 2% Ru loading (total [Ru]).

### 15.2. Pyrimidine as DG

Pyrimidine is often tested as secondary DG in many protocols developed for pyridine-based strategies, and in many cases works as well in the same or very similar reaction conditions. Nonetheless, there are several cases in the literature where the pyrimidine DG works differently, and many transformations are specific for this DG. In particular, pyrimidine is often the DG of choice for the functionalisation of indoles (and pyrroles) in C2, and in most cases is used only for such substrates. Because of this, a separate section has been devoted to this DG. Protocols that are very similar to those reported for pyridine will not be reported again in this section (the interested reader can find many information in Section 15.1.1), and more selected literature will be discussed here.

**Scheme 137:**
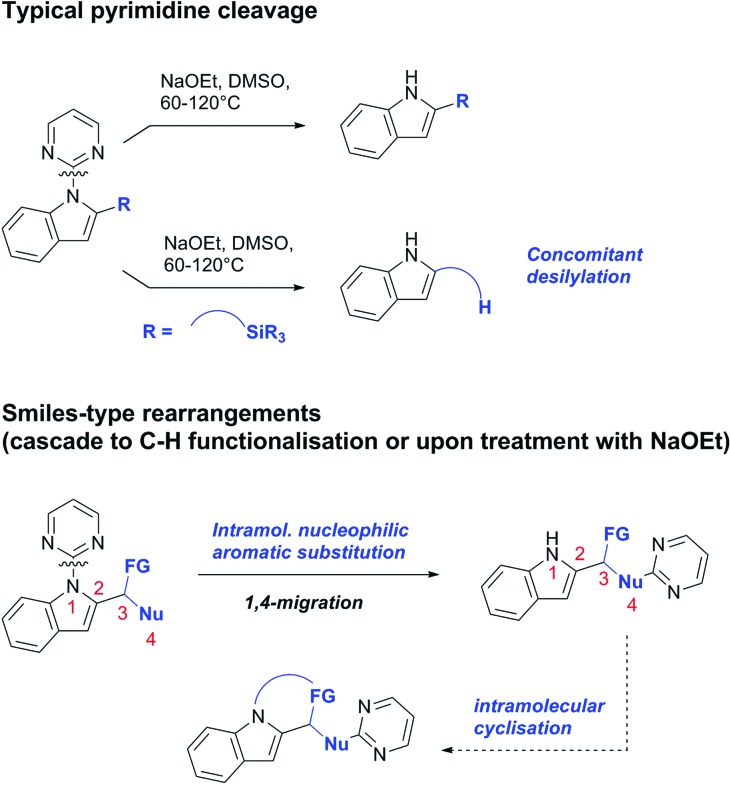
Common strategies for pyrimidine DG cleavage or migration.

The cleavage of the pyrimidine ring is, as for pyridine, typically not possible when attached *via* a C–C bond, but it is easy when the DG is attached *via* a C–N bond (*e.g.* in indoles, Scheme 137). In this case no quaternisation is necessary (as it is with pyridine), and often simply heating with NaOEt in DMSO is enough to produce the pyrimidine-free substrate (side reactions may occur depending on R, for example in the case of silanes, Scheme 137). Unlike pyridine, however, pyrimidine commonly gives rise to rearrangements, instead of the desired cleavage. In particular, Smiles-type rearrangements (intramolecular nucleophilic aromatic substitution), resulting in 1,4-migration of the pyrimidine ring, have been observed several times when a nucleophilic site is available at a specific position, as shown in Scheme 137. These are specifically reported in the text whenever they were noted in the original publication.

#### 15.2.1. Arylation

**Scheme 138:**
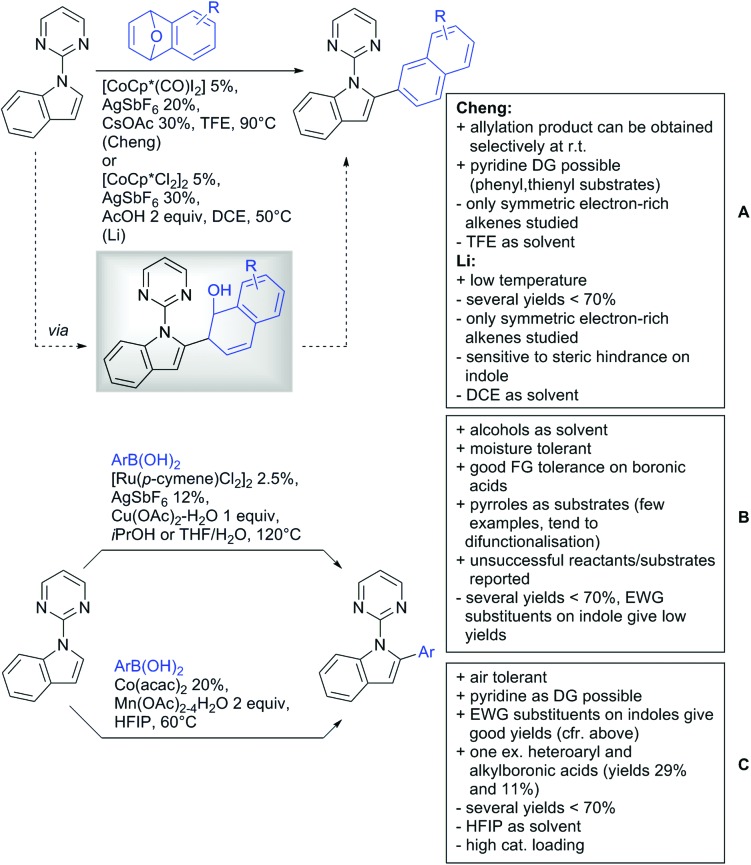
Pyrimidine-directed arylation of indoles.

As many arylation reactions have been reported before 2015 and many protocols are similar to those developed for pyridine DGs, few examples will be reported here. Li and Cheng reported the naphthylation of indoles using 7-oxabenzonorbornadienes as reactants under Co catalysis (Scheme 138A).^811^,^812^ This class of compounds had been used before with other DGs and under Rh catalysis,^813^,^814^ but the use of base metals constitutes an important improvement. The reaction occurs *via* addition of the indole to the double bond of the alkene, leading to an intermediate cyclic allylation product.^812^ Interestingly, Cheng found that the reaction can be stopped at this step if performed at room temperature instead of 90 °C. Arylation with arylboronic acids was reported by Pilarski and Niu/Song respectively with Ru and Co catalysts under oxidising conditions (Scheme 138B and C).^815^,^816^ Despite the relatively high amount of Co and the HFIP solvent used in the latter protocol, the use of Co catalysis results in higher tolerance of electron-withdrawing substituents on the indole moiety, which instead result in low yields in the Ru-catalysed reaction (Scheme 138B).

#### 15.2.2. Alkylation

**Scheme 139:**
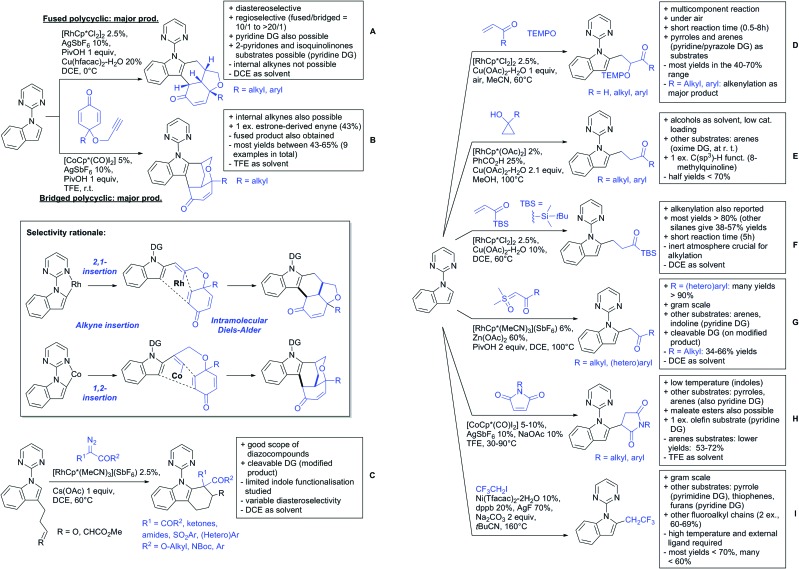
Pyrimidine-directed alkylation of indoles.

Some interesting reports on formal alkylation reactions leading to complex molecules have been recently published. Li showed an alkyne insertion/intramolecular Diels–Alder process between *N*-pyrimidylindoles and benzoquinonic 1,6-enynes leading to fused and bridged polycyclic compounds (Scheme 139A and B).^817^ While with Rh catalysis the fused polycyclic product was obtained almost exclusively, the use of Co allowed preferential formation of the bridged one, although not exclusively. The product switch arises from the different regioselectivities of the initial olefination step (Rh: alkyne 2,1-insertion; Co: 1,2-insertion), in turn due to different steric sensitivities (the Co catalyst is suggested to be more sterically sensitive, giving preferentially the least hindered insertion intermediate). It is worth mentioning that the use of the classical pyrimidine cleavage protocol (NaOEt in DMSO, see Scheme 137) on the fused product did not result in DG cleavage, but in the cleavage of the oxa-cycle, giving the corresponding phenol.^817^

Alkylation with diazo compounds, followed by *in situ* intramolecular aldol or Michael-type reactions was reported by Zhou. In this case, 2,3-fused indoles were obtained (Scheme 139C).^818^ Reactants featuring ester, amide, aromatic and heteroaromatic rings could be successfully employed in the reaction with good yields. Huang and Wang recently reported a multicomponent reaction between *N*-pyrimidylindoles, acrolein derivatives and TEMPO (Scheme 139D).^819^ The first step of this reaction is the addition of indole to the double bond of the acrolein (Rh-catalysed), followed by Cu-mediated introduction of the TEMPO radical in α to the carbonyl. Interestingly, MeCN as solvent proved crucial in suppressing other reaction pathways (*e.g.* β-hydride elimination and protonation), despite failing to do so when acrylic ketones were used instead of aldehydes. Apart from acyclic derivatives, Li introduced the use of cyclopropanols as new reactants for the introduction of carbonylethyl chains (Scheme 139E).^790^ Not being electrophilic compounds, their use constitutes a challenge and requires oxidising conditions (superstoichiometric Cu used). The reaction requires low Rh loadings (2%) and can be performed on arenes as well as indoles, but the yields obtained do not appear competitive in comparison to the use of the more common acrylates. Even in this case, the use of NaOEt in DMSO for pyrimidine cleavage did not result in the desired product, but in the migration of the pyrimidine from the indole to the alkyl chain.^790^

The use of acryloyl silanes was reported by Loh and Feng, and resulted in high yields of the corresponding alkylated products (Scheme 139F).^820^ Li and Ackermann/Li reported the use of sulfoxonium ylides (carbene precursors) and maleimide for the alkylation of indoles (Scheme 139G and H).^821^,^822^ These reactants have also been used with pyridine DGs, with somewhat different procedures (*cf.*Scheme 110K and Scheme 111A). In the case of Scheme 139H, again the use of NaOEt to cleave the DG resulted in pyrimidine migration. Finally, Shi reported an interesting Ni-catalysed trifluoroethylation of indoles using trifluoroethyl iodide as reactant. Unfortunately, not very high yields were obtained for a range of indoles and other heterocycles, with most yields remaining below the 70% threshold (Scheme 139I).^823^

#### 15.2.3. Allylation

**Scheme 140:**
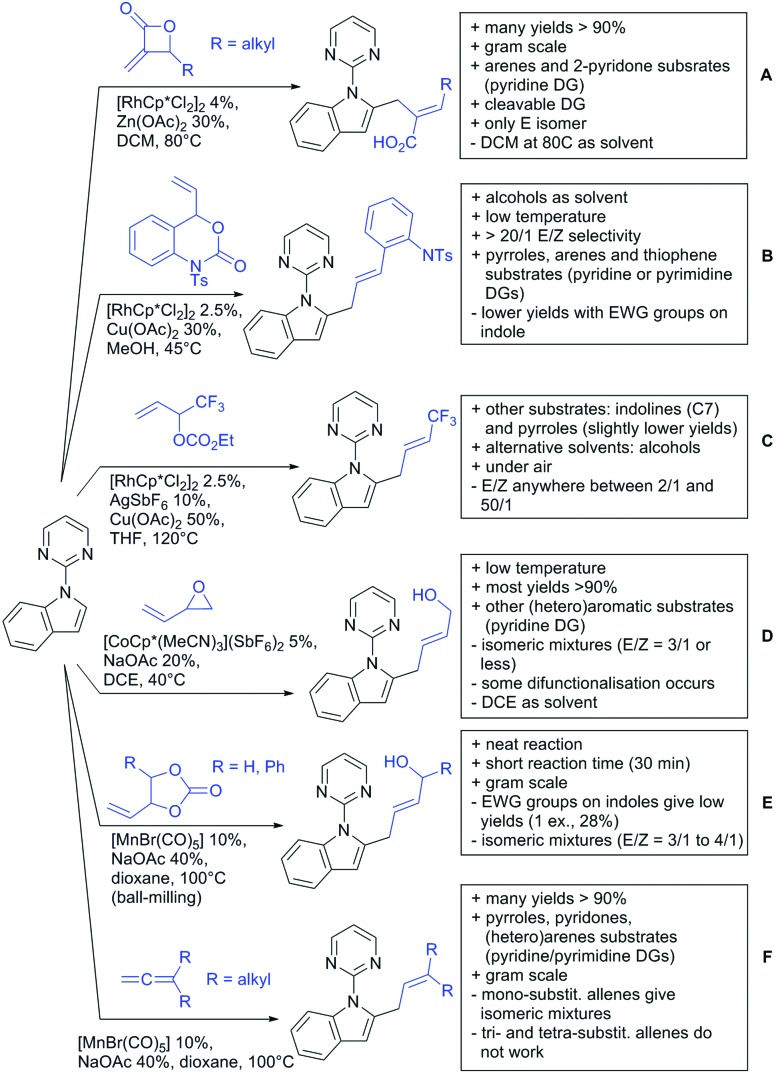
Pyrimidine-directed allylation of indoles.

As previously reported for pyridine DGs, many allylation protocols involve reactions with allyl alcohols, halides, or other activated allylic precursors (strained rings or leaving groups). In this context, a few procedures using different precursors or methodologies have been reported for the allylation of indoles. Li reported the use of both 4- and 6-membered rings as allylic precursors in Rh-catalysed reactions (Scheme 140A and B).^824^,^825^ While in the first case the driving force is the opening of the strained 4-membered lactone, the release of CO_2_ facilitates the opening of the 6-membered ring. Importantly, both reactions proved to be selective for the *E* isomer. Another interesting procedure was reported by Kim for the synthesis of trifluoromethylallylated indoles and indolines, employing α-trifluoromethylallyl carbonates (Scheme 140C).^826^ The reaction was performed in THF, but *t*AmylOH was found to be a valid alternative. Notably, the allylation of indolines in C7 did not occur with the pyrimidinyl DG, but required carbonyl-based DGs.^826^ The use of NaOEt on the allylated indole did not result in the pyrimidyl cleavage, but in olefin isomerisation instead, although in modest yields (52%). Moving to base metals, Li reported the use of vinyloxiranes under Co catalysis (Scheme 140D),^811^ allowing allylation of indoles in high yields and at low temperatures, despite the low stereoselectivity. As vinyloxiranes, vinyldioxolanones had also been previously reported as allylating agents, but Yu disclosed their use in a solventless ball-milling protocol under Co catalysis (Scheme 140E).^827^ Despite the neat conditions and only requiring 30 min for completion, mixtures of isomers were obtained from the reaction, as previously observed with these reactants (*cf.*Scheme 113B).

An interesting Mn-catalysed allylation reaction with 1,1-disubstituted allenes was reported by Wang (Scheme 140F).^828^ Very high yields were obtained for many examples, and the procedure is applicable to a variety of (hetero)arenes apart from indoles, using either pyrimidine or pyridine DGs. Unfortunately, monosubstituted allenes resulted in isomeric mixtures, and tri- and tetra-substituted ones proved ineffective in the reaction.

#### 15.2.4. Alkenylation

**Scheme 141:**
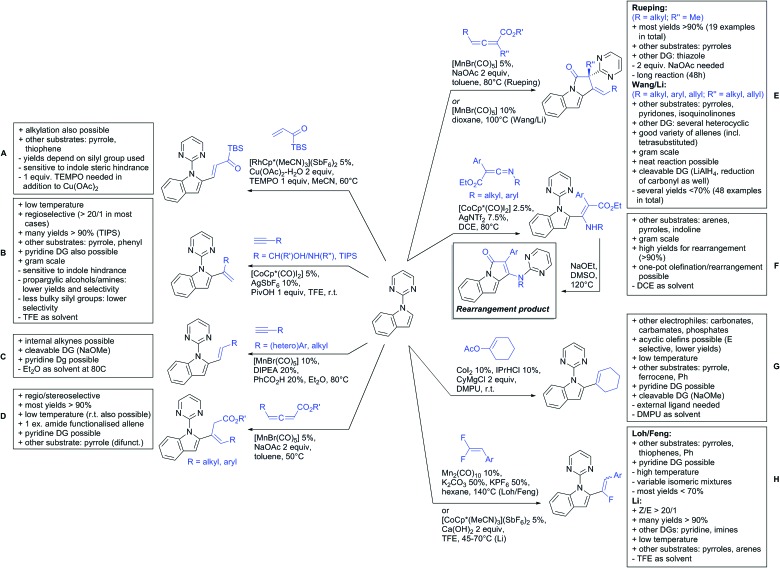
Pyrimidine-directed olefinations.

Olefination protocols have been investigated on *N*-(2-pyrimidyl)indoles exploiting either oxidative dehydrogenative coupling with alkenes (Fujiwara–Moritani reaction), addition to alkynes or allenes, or *via* cross-coupling type reactions. Loh and Feng reported the reaction with acryloyl silanes for the formation of both alkylated (see Scheme 139F above) and alkenylated products (Scheme 141A).^820^ In the case of alkenylation, 2 equiv. Cu(OAc)_2_ were used as oxidant, together with 1 equiv. TEMPO, which was suggested to prevent silane polymerisation and assist in the oxidative turnover of the Rh catalyst. It is noteworthy that this reaction is somewhat sensitive to the steric hindrance on both indole's C3 and C7 (not commonly observed) and on the identity of the silane group.^820^

Li and Lan reported a rare selective 1,2-insertion of terminal alkynes using Co catalysis, giving α-*gem*-vinylindoles (Scheme 141B).^829^ The use of Co and TFE as solvent was crucial for the selectivity, due to the high steric sensitivity of Co and the H-bonding abilities of TFE. Propargylic alcohols, amines and silanes performed well when used as reactants, although yields and selectivity depended on their structure. The use of NaOEt for the cleavage of the DG resulted in the transfer of the heterocycle to the hydroxyl of the propargylic moiety.

A complementary procedure, giving the 2,1-insertion product with terminal alkynes was reported by Li and Lei using Mn as catalyst (Scheme 141C).^830^ Aryl- and alkyl-substituted alkynes proved efficient reactants in most cases, and the reaction could be performed on internal alkynes as well. A practical downside here is the use of Et_2_O at 80 °C.

Rueping and Wang/Li independently reported the Mn-catalysed olefination of indoles with allenic esters. The use of 1,3-disubstituted allenes (Rueping) resulted in the hydroarylation product (arylation at the C2 position of the allene) in a regio- and stereoselective manner (Scheme 141D).^831^ Interestingly, the reaction with tri- or tetra-substituted allenes (Rueping, Wang/Li) resulted in a cascade hydroarylation/DG migration *via* a Smiles-type rearrangement (see Scheme 137), furnishing the products in Scheme 141E.^831^,^832^ While Rueping's procedure^831^ resulted in higher yields (often >90%), but required 2 equiv. of NaOAc as additive, Wang/Li's protocol^832^ gave lower yields in several cases (although a much broader substrate scope was reported), but without the addition of any additive. A number of post-functionalisation reactions were reported by both authors, including: alkene reduction, amide hydrolysis (with consequent pyrimidine migration back to the indole), decarboxylative ring opening, and DG cleavage with LiAlH_4_ (concomitant carbonyl reduction).^831^,^832^

The Co-catalysed hydroarylation of ketenimines resulted in the olefination product depicted in Scheme 141F in generally good yields (lower yields on pyrroles, arenes and indolines).^833^ The use of NaOEt on these compounds resulted in a pyrimidine migration similar to that in Scheme 141E, *via* intramolecular nucleophilic attack of the enamine to the pyrimidyne ring.^833^ Finally, a couple of substitution procedures for the olefination of indoles were reported using alkenyl acetates or *gem*-difluoroalkenes. The reaction with cyclic and acyclic alkenyl acetates and similar electrophiles was reported by Ackermann under Co catalysis (Scheme 141G).^834^ The Mn and Co-catalysed reaction with difluoroalkenes was reported respectively by Loh/Feng and Li (Scheme 141H).^835^,^836^ While the substrate scope remains similar, the Co-catalysed process performed much better in terms of reaction temperature, yields and stereoselectivity (*Z* selective). In this reaction, Ca(OH)_2_ is suggested to act as a scavenger for the HF produced, pushing the reaction forward.^836^ It is worth noting though, that the Mn-catalysed reaction shows a certain preference for the less stable *E* isomer for certain substrates.^835^

#### 15.2.5. Alkynylation

**Scheme 142:**
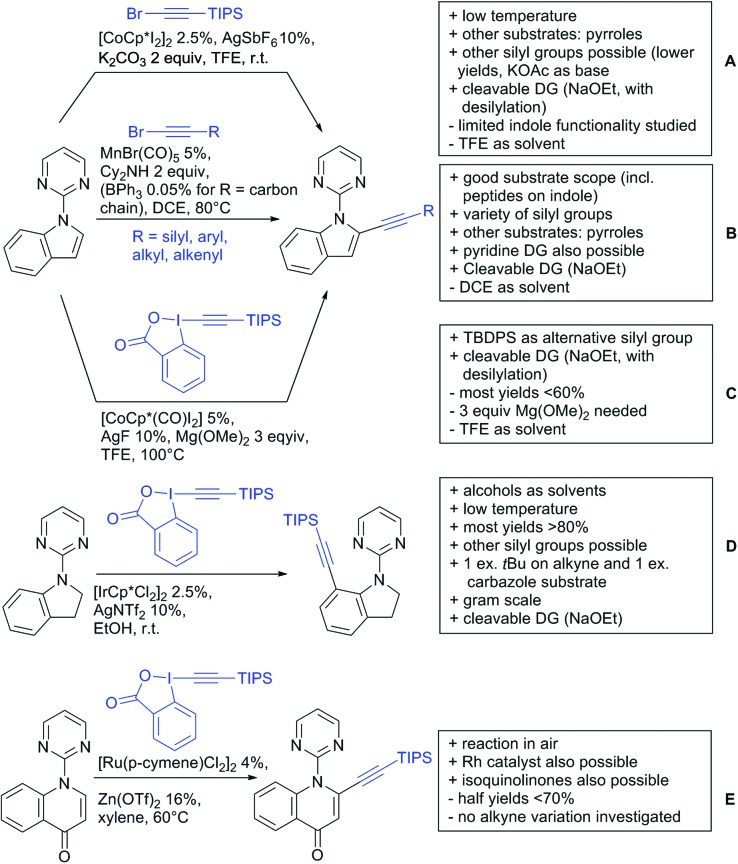
Pyrimidine-directed alkynylations.

Concerning alkynylation, although the reactants used are very limited and are the same reported for the pyridine DG, a few reports have appeared on the use of base metal catalysis for these functionalisations (which is not common for pyridine DG), mostly in halogenated solvents. Thus, Ackermann reported both Co- and Mn-catalysed reactions with bromoalkynes for the C2 functionalisation of indoles (Scheme 142A and B).^837^,^838^ In particular, the Mn catalyst (Scheme 142B)^838^ proved very versatile, allowing the use of either silylated (common), and aryl-, alkyl-, alkenyl-functionalised bromoalkynes (rare). The key for the use of non-silylated reactants was the addition of a small amount (0.05%) of BPh_3_ as Lewis acid, which promoted yields in many cases >80%. This protocol shows an overall good substrate scope, and peptides were tolerated on the indole substrate. Another Co-catalysed protocol was reported by Shi, making use of the hypervalent iodine reactant TIPS-EBX (Scheme 142C).^839^ This protocol gave generally modest yields and required 3 equivalents of Mg(OMe)_2_ as additive. Its role in the reaction was not clarified.

Li and Zhou disclosed a good example of C7 alkynylation of indolines using Ir catalysis (Scheme 142D).^840^ The protocol presents several advantages, such as the high yields, the use of ethanol as solvent at room temperature, and one example of reaction with *t*Bu-EBX (yield 84%). Finally, Hong reported a Ru- or Rh-catalysed alkynylation of 4-quinolones and isoquinolinones with TIPS-EBX (Scheme 142E).^841^ This is a rare example of the use of pyrimidine as DG for the functionalisation of such type of compounds, usually performed using pyridine as DG. The reaction proceeds well in air, but no variation in the reactant (other silyl or carbon groups) was investigated. It is noteworthy that the cleavage of the pyrimidyl DG using basic conditions (NaOEt or NaOMe) when silanes are present in the final product results in the concomitant desilylation (see also Scheme 137), which might constitute an advantage for synthetic purposes.

#### 15.2.6. Cyanation, acylation and aminocarbonylation

**Scheme 143:**
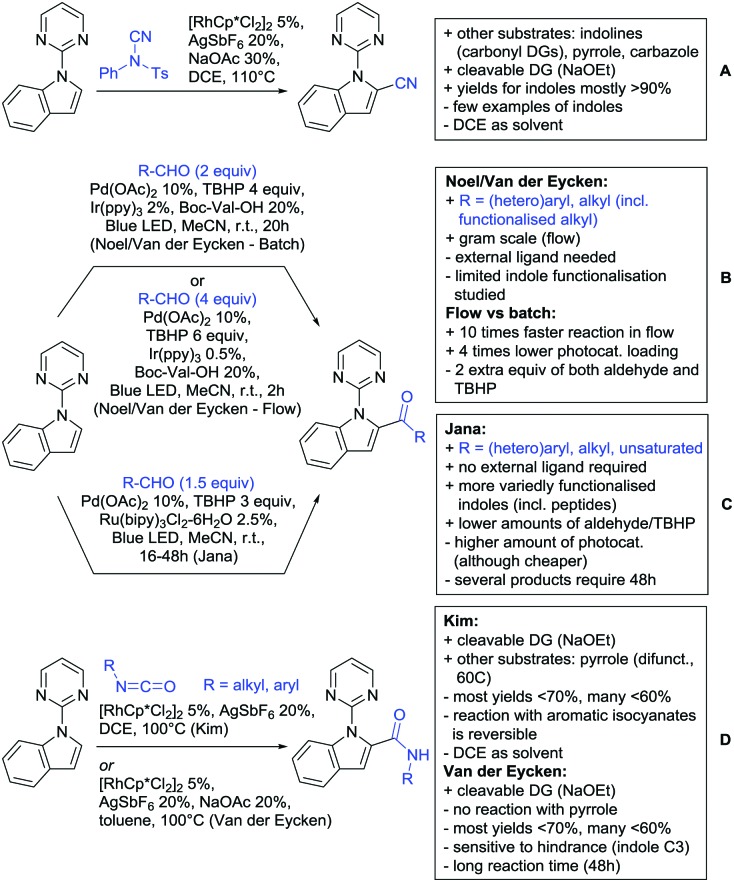
Pyrimidine-directed cyanation, acylation and aminocarbonylation of indoles.

An example of cyanation of indoles using NCTS as reactant under Rh catalysis has been reported by Kim (Scheme 143A).^842^ Only a few examples of indole functionalisation were reported in his publication (high yields, mostly >90%), but the same reaction conditions (at 130 °C instead of 110 °C) can be applied to the C7 cyanation of indolines, with carbonyl-based DGs. The pyrimidine DG could be employed for the mono-cyanation of pyrrole and carbazole.

As for the pyridine DG, a number of publications on the use of Pd catalysis in combination with peroxides and acyl radical precursors (aldehydes, α-diketones, toluene) for the introduction of an acyl group onto *N*-pyrimidinylindoles have been reported.^843^–^845^ The general conditions are: 5–10% Pd(ii) catalyst, 3–5 equiv. peroxide, apolar solvent (*e.g.* toluene, THF), at 90–120 °C, and the reaction follows the general mechanism shown in Scheme 118. From these conditions, Noel/Van der Eycken and Jana independently reported a protocol for the group-directed acylation of indoles with aldehydes using a dual photoredox-Pd catalytic strategy which allows functionalisation at room temperature.^846^,^847^ Noel/Van der Eycken reported both a batch and a flow protocol. Although only slightly higher or comparable yields were obtained in flow, and much higher amounts of aldehyde and TBHP were required, the reaction time was drastically decreased using this methodology (2 h instead of 20 h), as well as the photocatalyst loading (Ir) (Scheme 143B).^846^ Jana's protocol (batch), although requiring reaction times up to 48 h, was tested on more variedly functionalised indole substrates, giving overall comparable yields to the Noel/Van der Eyken protocol. An important advantage is the avoidance of the external ligand and the use of a lower excess of aldehyde/TBHP (Scheme 143C).^847^ Both protocols could be applied to benzylic alcohols (oxidised to aldehydes *in situ*), and the DG could be cleaved in basic conditions (NaOEt).

The photoredox catalyst in these procedures facilitates the reaction by promoting the formation of the *t*BuO radical first, needed to generate the acyl radical, and then the oxidation of the Pd(iii) intermediate to Pd(iv), required for the reductive elimination step (see also Scheme 118, bottom). As these activations are not available in the simple Pd(ii) catalytic reaction, high temperatures are required to push the reaction forward.

Two very similar procedures for the aminocarbonylation of indoles with aromatic or aliphatic isocyanates were reported by Kim and Van der Eycken in 2015 using a Rh dimer as catalyst (Scheme 143D).^848^,^849^ Both processes required 2.5 to 3 equiv. of isocyanates to perform decently, but the yields remain modest in both cases. Importantly for synthetic purposes, Kim observed the reaction with aromatic isocyanates to be reversible, thus explaining in these case the low yield obtained.^848^ Such reversibility had been observed before for the Rh-catalysed hydroarylation of imines.^850^ Both aliphatic and aromatic amide products could be treated with NaOEt to provide the deprotected indole. Interestingly, Kim showed that the use of EtOH as co-solvent in the process (standard solvent: DMSO) facilitates the reaction.^848^,^851^

#### 15.2.7. C–N bond formation

The C–N bond formation still relies mostly on precious metal catalysis, and only few examples are available in the literature on the use of base metals.

**Scheme 144:**
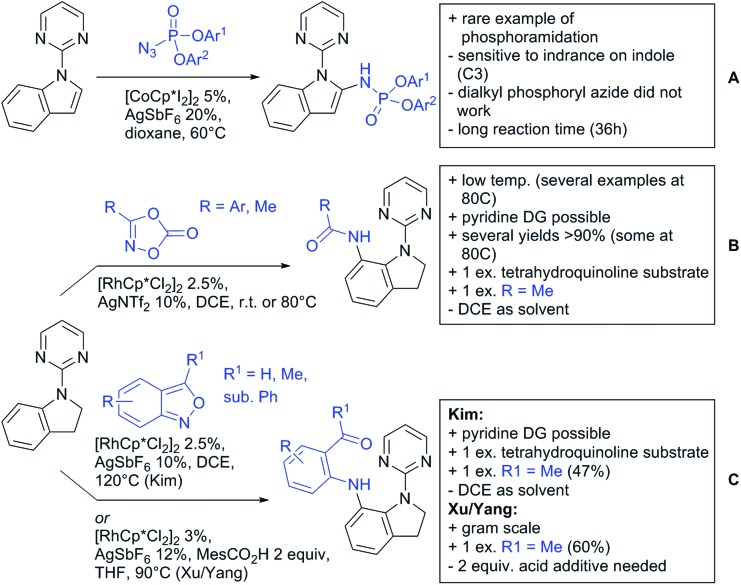
Phosphoramidation and amination on indoles and indolines.

Indole phosphoramidation was reported by Kanai and Matsunaga, notably under Co catalysis (Scheme 144A).^852^ Although the process presents some drawbacks, such as the long reaction times and the sensitivity to steric hindrance in the C3 position of the substrate, this is a rare base metal-catalysed example of a C–H phosphoramidation reaction. Substituted dioxazolones were instead employed for the carboxyamidation of indolines at the C7 position at room temperature (or 80 °C for several examples, Scheme 144B).^853^ The reaction proceeds giving generally good yields. Interestingly, the reaction proved efficient on tetrahydroquinoline as well, a not commonly studied substrate.

Concerning indoline amination, anthranils were used under Rh catalysis by both Kim and Xu/Yang (Scheme 144C).^854^,^855^ The substrate scope investigated and the results are comparable in the two cases. For these substrates, subsequent intramolecular cyclisation to quinoline fused indoline (as observed in indoles, *cf.*Scheme 125), did not occur *in situ*, but can be promoted by reaction of the formylaniline product (R^1^ = H) with TFA.^854^

#### 15.2.8. C–S bond formation

**Scheme 145:**
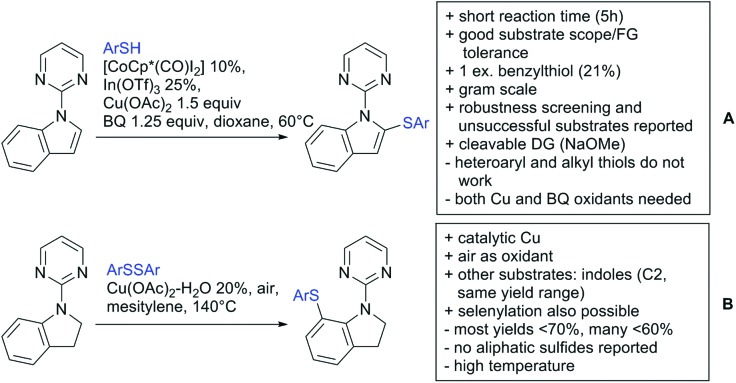
Directed C–S bond formation on indoles and indolines.

A few procedures for the C–S bond formation in pyrimidyl-functionalised substrates have appeared. Kambe reported on the use of disulfides and superstoichiometric amounts of Cu(ii) salts to promote the thiolation of carbazoles and indoles. Although good yields were obtained in many cases, and both aromatic and aliphatic disulfides could be efficiently employed, temperatures in the range 140–160 °C were used, and difunctionalisation of indoles at the C2 and C3 positions occurs under these conditions.^856^,^857^ The thiolation of indolines was instead reported by Wang using Rh catalysis and stoichiometric amounts of Ag oxidant; even in this case, high temperatures had to be used.^858^ More recently, two interesting reports were released by Ackermann and Glorius for the thiolation of indoles and indolines respectively. Glorius's approach focused on the use of aromatic thiols and Co(iii) as catalyst, requiring the combined action of superstoichiometric Cu and BQ as oxidants (Scheme 145A).^859^ Despite these drawbacks, short reaction times, much lower temperatures than usual (60 °C) and a good FG tolerance make this approach stand out. Unfortunately, functionalisation with alkyl or heteroaryl thiols proved unsuccessful. The C7 thiolation of indolines reported by Ackermann is also particularly interesting since no stoichiometric oxidant was used apart from air, requiring only a catalytic amount of Cu (Scheme 145B).^860^ The same conditions proved effective also for thiolation and selenylation of indoles. Drawbacks of this protocol are the high temperature required and the relatively modest yields obtained for most examples.

### 15.3. N-, O-, S-, and Si-Linked (di)azines as DGs

**Scheme 146:**
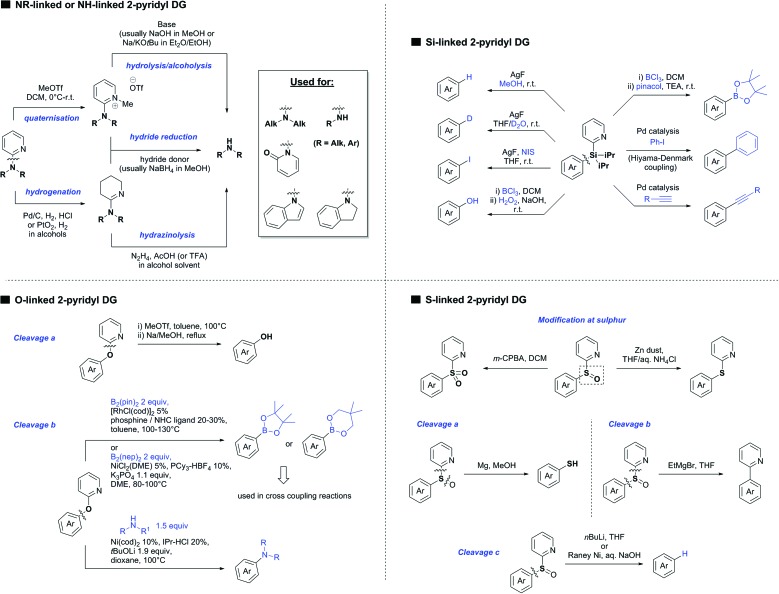
Protocols for the cleavage/modification of N-, O-, S-, and Si-linked pyridine DGs.

The pyridine (and pyrimidine) DGs are not easily cleavable when attached *via* a C–C bond. Nonetheless, when attached *via* a C–heteroatom bond, the cleavage becomes relatively easy. This type of bond is found, apart from pyridine- or pyrimidine-functionalised indoles, indolines and pyridones, in a number of other substrates, where the DG is attached *via* a NH_2_-, O-, Si-, or SO/SO_2_ linker. Apart from their sometimes particular reactivity pattern, the cleavage of these DGs is a very important feature for synthetic purposes. Scheme 146 summarises the different cleavage/modification methods available in the literature for such DGs.

The N-linked pyridine DG is by far the most explored among this family of DGs, and several methods have been developed with time for its cleavage, and routinely used. A common method utilises the “quaternisation-hydrolysis/alcoholysis” strategy,^861^ which involves methylation of the pyridine with MeOTf (no other methylating agent is generally used), followed by hydrolysis or alcoholysis. Other methods are the “quaternisation-hydride reduction”,^862^ where hydrolysis/alcoholysis is replaced by the reaction with a hydride donor. Alternative cleavage procedures involve a first hydrogenation step, leading to an unstable aminal. The cleavage of the latter can then occur using a hydride donor, or *via* hydrazinolysis.^862^–^864^ The quaternisation-based methods are most commonly encountered in the literature, with little variations from the conditions shown in Scheme 146, and are used for pyridine removal from indoles, pyridone, indolines, aliphatic amines and even anilines.

O-Linked 2-pyridyl DGs can also be easily cleaved (Scheme 146). A general procedure is the quaternisation of the pyridine ring, followed by treatment with NaOMe, resulting in the corresponding phenol.^865^ Apart from this traditional cleavage, a few cross coupling methods have been reported by Chatani and Wang to cleave the arene–O bond and introduce a boronic ester (which can be used in subsequent cross couplings)^866^,^867^ or an amino functionality^868^ in its place.

The use of a Si-linked 2-pyridyl and 2-pyrimidyl DG has the great advantage of being very easily cleaved at the arene–Si bond *via* substitution or cross-coupling reactions, as demonstrated in a seminal paper by the Gevorgyan group in 2011 (Scheme 146).^869^

The cleavage of S-linked 2-pyridyl DGs has been reported in a few papers, although it has not been extensively explored on a broad number of substrates. Interestingly, different possibilities are available for this linker, in particular modification (oxidation or reduction of sulfoxides) is straightforward,^870^,^871^ and cleavages at both the pyridine–S bond and the aryl–S bond have been reported (Scheme 146).^870^–^872^

#### 15.3.1. N-Linked (di)azines

Heteroaromatic DGs, in particular pyridines and pyrimidines have also been used as DGs when linked to a substrate *via* a free NH moiety. Apart from increasing the flexibility of the DG, the nucleophilicity of the free NH makes a number of tandem reactions possible, in particular with unsaturated moieties. This often leads to the formation of unsaturated or saturated rings, and has been exploited for the synthesis of various heterocycles. It is important to note that, although pyridine and pyrimidine can often promote the same transformation in the above cases, marked reactivity differences are instead often observed in the case of N-linked DGs, due to the presence of an amidine/guanidine moiety with strong (Lewis) basicity. Due to the limited number of examples in the literature, all recent transformations available with N-linked pyridine and pyrimidines will be reported in two sub-sections only.

##### 15.3.1.1. Functionalisation without cyclisation

A number of functionalisations have been reported which do not involve tandem cyclisation at the free NH moiety. In the C–H functionalised product, the DG can be removed *via* cleavage of the NH-heterocycle bond, resulting in functionalised anilines (Scheme 146).

**Scheme 147:**
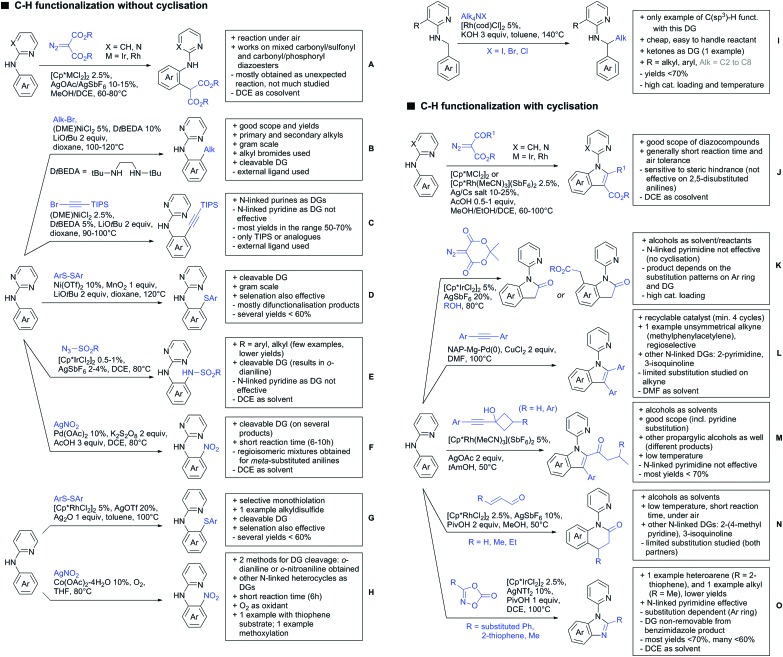
C(sp^2^)–H and C(sp^3^)–H functionalisation with NH-linked pyridine and pyrimidine DGs.

**Scheme 148:**
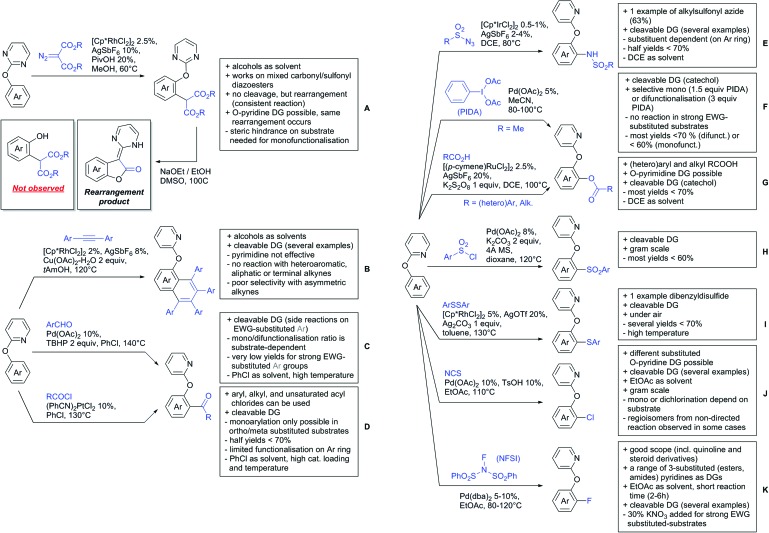
Functionalisation with O-linked pyridine and pyrimidine DGs.

The alkylation with diazodiesters has been reported a number of times with Rh or Ir catalysts (Scheme 147A).^873^–^875^ Ackermann reported the Ni-catalysed *ortho*-alkylation and alkynylation of *N*-(2-pyrimidyl)anilines, using alkyl/alkynyl bromides (Scheme 147B and C).^876^–^878^ The same group also reported a Ni-catalysed thiolation with disulfides, leading mostly to difunctionalised products (Scheme 147D).^878^ Interestingly, a similar protocol using Rh catalysis and *N*-(2-pyridyl) or *O*-(2-pyridyl) as DG was shown to provide selectively the monofunctionalised phenols (Scheme 147G and Scheme 148I).^879^ Using *N*-(2-pyrimidyl), Cui reported the Ir-catalysed reaction with sulfonyl azides adding only catalytic amounts of AgSbF_6_ to the reaction. Although resulting in lower yields (max 63%), two examples of alkylsulfonyl amides were also reported, which adds value to the procedure (Scheme 147E).^880^ In this case, the use of aq. HCl on the reaction products results in the cleavage of both DG and amide, giving *ortho*-phenylenediamines. Two procedures for *ortho* nitration were reported with AgNO_2_ as nitro source. While Pd catalysis on *N*-(2-pyrimidinyl)anilines using K_2_S_2_O_8_ as oxidant resulted in regioisomeric mixtures in *meta*-substituted anilines (Scheme 147F),^881^ this was not observed on the *N*-(2-pyridinyl) analogues using aerobic Co catalysis (Scheme 147H).^882^ Noteworthy in this case is also the range of other N-linked heterocycles effective in the reaction, including pyrimidine, quinolines, pyridazine and benzothiazole. Also two different cleavage protocols were reported, leading to either the *o*-nitroaniline or the *o*-phenylenediamines.^882^ Finally, Schnürch reported the alkylation of benzylic C(sp^3^)–H bonds in *N*-(2-pyridinyl)benzylamines using Rh catalysis and tetraalkylammonium halides as terminal olefins surrogates (Scheme 147I).^883^ To our knowledge this is the only case of C(sp^3^)–H functionalisation with this DG type.

##### 15.3.1.2. Functionalisation with cyclisation

In these reactions the free NH of the DG reacts with an unsaturated moiety, leading to the formation of aza-heterocycles, particularly indole derivatives. In the final compounds the heterocyclic DG can be generally cleaved by the typical methods described in Scheme 146. The reaction with diazo-β-ketoesters or similar results in 3-alkoxycarbonyl-2-alkylindoles (Scheme 147J, *cf*. Scheme 147A),^873^–^875^,^884^ while the use of Meldrum's acid furnishes 1,3-dihydroindol-2-ones, although the product obtained strongly depends on the substitution pattern on the DG or the aryl ring (Scheme 147K).^885^ 2,3-Diarylated indoles can be obtained by reaction with alkynes, of which Kantam recently reported a heterogeneous version using Pd supported on nanocrystalline Mg oxide [NAP–Mg–Pd(0)] (Scheme 147L).^886^ This heterogeneous catalyst could be recycled and reused for at least 4 runs without considerable changes in yields.

An interesting alternative, reported by Zeng, utilises arylalkynylcycloalkanols for the regioselective formation of 2-acyl-3-arylindoles, occurring *via* consequent ring opening of the propargylic alcohol employed (Scheme 147M).^887^ The formation of dihydroquinolinones *via* Rh-catalysed alkylation/cyclisation with unsaturated aldehydes was recently reported by Huang, and to our knowledge this is the only 6-membered ring formation reported for such systems (Scheme 147N).^888^ Finally, an Ir-catalysed amidation/condensation protocol was developed for the synthesis of functionalised benzimidazoles (Scheme 147O).^889^ Unfortunately, the cleavage of the DG in the final product did not succeed using previously reported methods. In all these functionalisations, the heterocyclic DG is maintained unaltered in the final product, and can be exploited for further functionalisation of the heterocycles, *e.g.* at the C7 in indoles or analogous positions.

#### 15.3.2. O-Linked (di)azines

O-Linked (di)azines, mostly limited to pyridine, have been used less often than the corresponding N-linked analogues, but in general for different transformations, *e.g.* acylation, C–O bond formation. The heterocycle can be cleaved at the O-heterocycle bond to give functionalised phenols, or at the arene–O bond (*via* cross coupling) giving other doubly functionalised arenes (Scheme 146). As before, all the transformations will be treated in one section.

The alkylation with diazocompounds under Rh catalysis occurs in good yields on a variety of substituted phenols, although monofunctionalisation was only possible in somewhat hindered substrates (Scheme 148A).^890^ When known cleavage methods for pyridine/pyrimidine DGs were applied, no cleavage was observed, instead a Smiles-type rearrangement took place (see Scheme 137, unusual for pyridines). Interestingly, this rearrangement proved consistent and occurred in good yields for a number of different products.^890^ Cui reported the aromatic homologation of *O*-(2-pyridinyl)phenols with internal arylalkynes to result in substituted 1-naphthols in good yields using *t*AmOH as solvent (Scheme 148B). Unfortunately, the reaction with alkyl or heteroarylalkynes did not occur, and asymmetric alkynes resulted in very poor regioselectivity.^891^ Acylation reactions were reported by Wu and Huo, using aldehydes and acyl chlorides as reactants. The first method (Scheme 148C)^892^ uses TBHP as oxidant and follows the mechanism depicted in Scheme 118, resulting mostly in monoacylation (difunctionalisation is substrate-dependent), giving low yields for strongly EWG-substituted phenols. The second method (Scheme 148D)^893^ is a group-directed Friedel–Crafts acylation, and does not require oxidants or additives, but 10% of expensive Pt catalyst was employed for the reaction. The use of PhCl as solvent results in the formation of difunctionalised phenols, while monoacylated ones could be obtained in hindered substrates.

Comparatively more protocols for C–heteroatom bond formation were reported with this class of DG, although suffering in several cases from low yields (<70%). The reaction with arylsulfonyl azides was reported by Cui (Scheme 148E),^894^ and C–O bond formation can occur *via* reaction with PIDA or carboxylic acids (Scheme 148F and G).^895^,^896^ The DG cleavage on the products obtained results in *ortho*-hydroxyanilines or in catechols. C–S bond formation has been reported making use of sulfonyl chlorides or disulfides (Scheme 148H and I),^879^,^897^ and C–Cl and C–F bonds are formed under Pd catalysis using NCS of NFSI as electrophilic halogen sources (Scheme 148J and K).^898^,^899^

#### 15.3.3. Si-Linked (di)azines

The use of pyridyl- and pyrimidyldiisopropylsilyl DGs was developed by the Gevorgyan group. These DGs are characterised by a truly unique variety of cleavage options, which makes this DG type synthetically very interesting (Scheme 146). This advantage is, however, somewhat counterbalanced by a lower efficacy of these DGs, which results in long reaction times, or the necessity of a large excess of reactants/reagents.

**Scheme 149:**
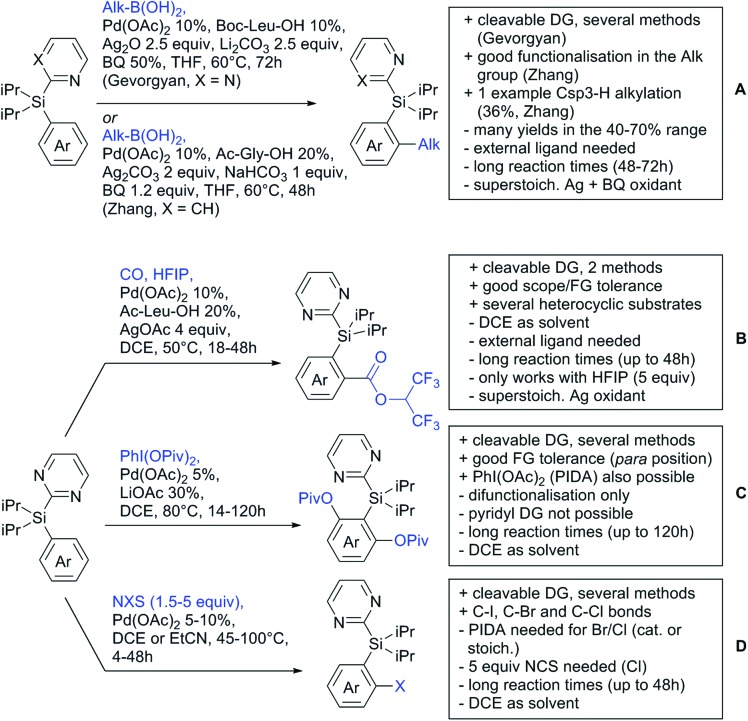
Functionalisation with Si-linked pyridine and pyrimidine DGs.

Alkylation reactions were reported in 2016 by Gevorgyan (pyrimidylsilyl) and Zhang (pyridylsilyl), using very similar protocols: Pd catalysis, alkylboronic acids as reactants, superstoichiometric amounts of Ag salts as oxidant, an inorganic base and an aminoacid as ligand (Scheme 149A).^900^,^901^ Both protocols require long reaction times (48–72 h) and produce yields often in the range of 40–70%. Notable features are the smooth cleavage/substitution of the DG (Gevorgyan), the use of variedly functionalised alkylboronic acids (Zhang) and a single example of C(sp^3^)–H functionalisation (Zhang). Alkoxycarbonylation was reported by Gevorgyan using CO and hexafluoroisopropanol (HFIP, Scheme 149B).^902^ A good substrate scope and FG tolerance was obtained for this protocol; particularly noteworthy is the use of several heterocyclic substrates (dibenzofuran, thiophene, carbazole, indoline, and tetrahydroquinoline), featuring medium to good yields (47–77%). The same group also reported the C–O and C–halogen bond formation on the same starting materials. Diacyloxylation with hypervalent iodine reactant PhI(OPiv)_2_ proved effective on a number of *para*-substituted arenes in good yields, but required up to 120 h (Scheme 149C).^903^ C–halogen bond formation was achieved using NXS as reactants (X = I, Br, Cl), with different reaction conditions for each halogen (Scheme 149D).^903^

#### 15.3.4. S-Linked (di)azines

**Scheme 150:**
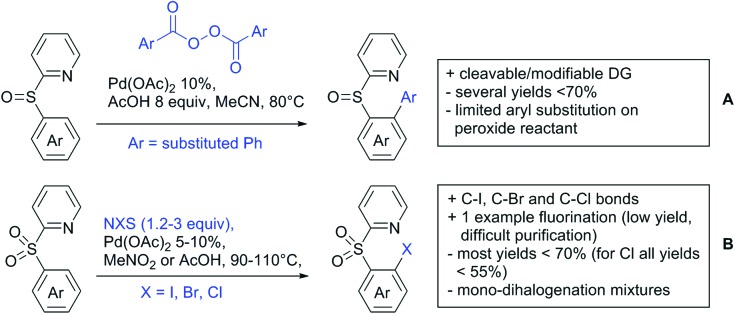
Functionalisation with S-linked pyridine DGs.

Only a couple of reports on the use of S-linked (di)azines have appeared recently and focused on the use of pyridine. Arylation with benzoyl peroxides was reported by Sun, and halogenation with NXS by Hierso (Scheme 150A and B).^871^,^904^ Several yields were <70% for both reports, and product mixtures were obtained in the case of halogenation (mono- + dihalogenated products). Interestingly, modification (oxidation to sulfone, reduction to sulphide), desulphurisation (to 2-arylpyridines), and cleavage of the DG (obtaining thiols) was demonstrated by Sun (Scheme 146).^871^

### 15.4. Quinoline-*N*-oxide as substrate/DG

While the activation of C–H bonds (either metal-catalysed or metal-free) at the *ortho* position of pyridine-*N*-oxides has been known for long time, this activation is mostly due to electronic effects.^905^–^907^ These cases will therefore not be treated here. Instead, metal-catalysed reactions where the *N*-oxide is thought to have a coordinating and directing role will be discussed. It is important to mention that the *N*-oxide moiety is the DG here, and is therefore limited to the functionalisation of azines. Although, strictly speaking, the coordinating atom is not itself part of a heterocycle, this DG has been considered in the heterocyclic section for simplicity and immediate analogy to its heterocyclic precursors. For the use of different *N*-oxide moieties in C–H functionalisation see Section 14.3.1 and Section 16.9.

The functionalisation of quinoline-*N*-oxides at the 8 position is most common in this area. Considering quinolines and their analogues are very common motifs in biologically-active molecules, the simple introduction (*e.g.* with peroxides) and cleavage (*e.g.* sulphur or phosphorus reagents)^907^,^908^ of the *N*-oxide moiety is very convenient for the selective functionalisation of these substrates. The facility of the *N*-oxide removal or the oxygen-transfer to the quinoline C2 (forming 2-quinolinones) or to an unsaturated moiety in the product (*N*-oxide as internal oxidant), make several of these procedures occur as cascade/tandem processes, leading to interesting compounds. These types of transformation have only been studied from 2014, and all the examples till 2017 have been reported in this section. Due to their limited number, all type or reactions are treated in one single section.

**Scheme 151:**
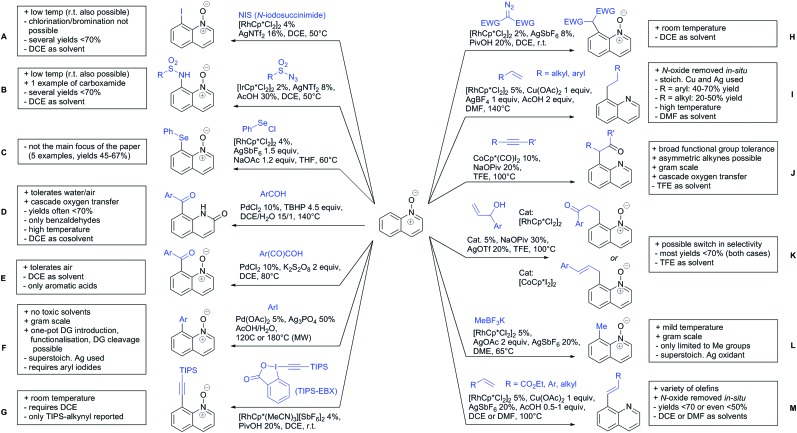
C8-Functionalisation of quinoline-*N*-oxides.

In 2014 Chang reported iodination and amidation with *N*-iodosuccinimide (NIS) and sulfonyl azides (Scheme 151A and B),^909^ also applied to the synthesis of Zinquin ethyl ester (a zinc indicator for biological systems). The only other report on the functionalisation with heteroatoms involved the use of selenyl chlorides for the formation of C–Se bonds. In this case, however, the *N*-oxide was not the main DG investigated and only 5 examples (45–67% yield) were included (Scheme 151C).^910^

Two acylation procedures were reported by Wu and Cui, both catalysed by PdCl_2_ in similar conditions. The use of TBHP (*tert*-butylhydroperoxide) with benzaldehydes resulted in the 8-acylation followed by *in situ* oxygen transfer from the *N*-oxide to the 2 position of the quinoline, producing 8-benzoyl-2-quinolinones (Scheme 151D).^911^ The use of α-oxocarboxylic acids as reagents in combination with K_2_S_2_O_8_ as oxidant produced instead 8-benzoylquinoline-*N*-oxides *via* decarboxylative coupling (Scheme 151E).^912^ A Pd-catalysed arylation with aryl iodides was reported by Larionov. Interestingly, this reaction performs best in AcOH/H_2_O, and no toxic solvents were required (Scheme 151F).^913^ On the other hand, high amounts of Ag_3_PO_4_ additive (1.5 equiv. total [Ag]) were used, presumably acting as both base and stoichiometric halide scavenger for the reactant. A similar procedure was later reported by the same authors for the dehydrogenative homocoupling of quinoline-*N*-oxides.^914^ 8-Alkynylation can be achieved using TIPS-EBX (Scheme 151G).^915^ This reaction, although requiring DCE as solvent and the use of a wasteful reactant, proceeds with good yields at room temperature.

Formal alkylation at the 8 position can be achieved with a number of reactants. Chang reported the use of diazomalonates or similar reactants at room temperature (Scheme 151H).^915^ Very recently, alkylation *via* reaction with styrenes or aliphatic olefins was reported by Sharma (Scheme 151I).^916^ Although the reported yields are relatively low (<70% for styrenes and <50% for aliphatic olefines), even long aliphatic chains could be attached to the quinoline moiety, which is generally quite uncommon. The reaction was suggested to take place *via* the olefinic intermediate, then reduced *in situ* with formic acid, derived from the DMF solvent. The suggested mechanism (only partially) explains the requirement of stoichiometric amounts of both Cu and Ag for an overall non-oxidative coupling.^916^ This method was also applied to the total synthesis of an EP4 antagonist. In 2014 the reaction with internal alkynes under Rh catalysis was independently reported by Chang and Li. This reaction occurred *via* formal addition to the alkyne, followed by an intramolecular oxygen transfer from the *N*-oxide to the new functionality, resulting in the ketones shown in Scheme 151J.^917^,^918^ The same reaction was then reported by Sundararaju under Co catalysis,^919^ and showed considerable improvements in terms of coupling partners: diaryl-, dialkyl-, and arylalkyl–alkynes could be easily coupled with good yields. The reaction with allylic alcohols resulted in two different compounds depending on the catalyst. While the use of Rh produced aroylethyl-functionalised compounds, the use of Co in the same conditions only resulted in allylated compounds (Scheme 151K).^920^ A methylation procedure using MeBF_3_K was recently reported by Liu (Scheme 151L);^921^ the same procedure can be applied for C–H arylation using aromatic trifluoroborates, but very few examples with <40% yields were reported in this case.

Two examples of olefination have appeared from 2014. Shibata first reported the Rh(i)-catalysed reaction with internal alkynes, a reaction that was plagued by isomeric mixtures and yields often <70%.^922^ A second example was reported in 2015 by Sharma *via* reaction with terminal olefins. Although even in this case many yields were low (even <50%), olefination took place with a number of acrylates, styrenes and other linear olefins, also resulting in *N*-oxide cleavage *in situ* (Scheme 151M).^923^

### 15.5. Pyrazole as DG

**Scheme 152:**
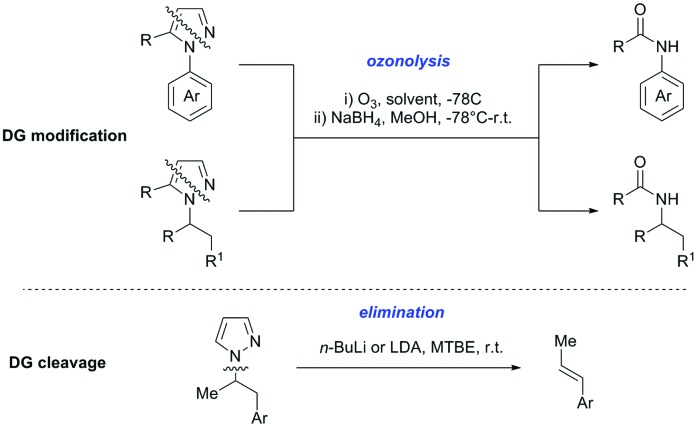
Examples of cleavage/modification of pyrazole DGs.

Pyrazoles are the most common 1*H*-diazole DG type used, and an important pioneering publication by Chatani on the specific use of this DG dates back to 2003.^924^ As many other heterocycles, in recent years pyrazole DGs are often tested as secondary DGs in procedures mostly developed using pyridine-based groups. In general, in these cases pyrazole performs giving comparable or little lower yields than the corresponding pyridine-directed reaction, and only one or two examples are nowadays generally reported in most publications. It is worth noting that the lower yields obtained in these cases might just be due to a lack of specific optimisation for pyrazole DGs, and do not necessarily mean that pyrazole is a worse DG than pyridine. In fact, from a practical point of view, there are a few differences that might make pyrazole a much better choice than pyridine depending on the synthetic aim. Most commonly 1-aryl/alkyl-1*H*-pyrazoles are used as substrates, while 3-aryl/alkyl-1*H*-pyrazoles are much less explored. In the former substrates, the DG is linked to the substrate *via* a C–N bond, thus making DG introduction easier than with a 2-pyridyl DG requiring a C–C linkage. For example, 1-alkyl/aryl-1*H*-pyrazole can be easily obtained by: (i) metal-free (or metal-catalysed) nucleophilic substitution/cross coupling; (ii) reaction of functionalised hydrazines with 1,3-diketones or unsaturated carbon moieties, including *in situ* hydrazine formation from amines.^925^ Moreover, its cleavage/modification, although rarely reported, is easier than with pyridine for arenes or olefins (*e.g.* ozonolysis/reduction^926^,^927^ or elimination reactions,^928^Scheme 152), provided that there is FG compatibility with the required reagents.

**Scheme 153:**
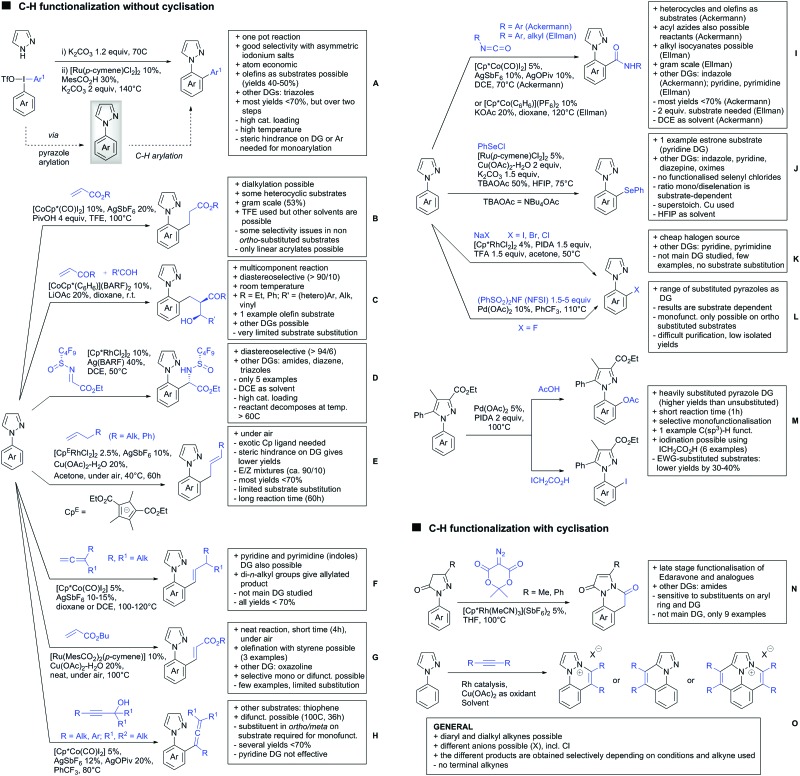
Pyrazole-directed C(sp^2^)–H functionalisation.

Although not too many, several C–H functionalisation methods specifically developed for pyrazoles (or with more than 1 or 2 examples anyway) have been published recently. Greaney reported an elegant, atom economic one-pot DG introduction/C–H arylation using diaryliodonium salts. In this procedure, pyrazole is first reacted with the iodonium salt in metal-free conditions (or with catalytic CuI) to provide the 1-phenyl-1*H*-pyrazole substrate, then a Ru catalyst is added to the reaction to trigger the *ortho* C–H arylation with the aryl iodide formed *in situ* during the first step (Scheme 153A).^929^ The most notable feature is the possibility to use asymmetric diaryliodonium salts: *N*-arylation of the pyrazole occurs selectively with the less electron-rich arene, or to the more sterically hindered one. The same procedure can be applied to styryl(phenyl)iodonium salts, giving 1-styryl-1*H*-pyrazoles, and subsequent arylation of the olefin.^929^ A number of alkylations have been reported *via* addition to double bonds. Sundararaju demonstrated the Co-catalysed addition to acrylates (Scheme 153B),^930^ while diastereoselective protocols have been reported by Ellmann. A multicomponent/cascade reaction with 1-phenyl-1*H*-pyrazoles, α,β-unsaturated ketones, and aldehydes provide access to functionalised branched alkyl chains with two consecutive stereocentres, which could be obtained in good diastereoselectivity (above 90/10, Scheme 153C).^931^

The addition of 1-phenyl-1*H*-pyrazoles to chiral *N*-perfluorobutanesulfinyl imines also occurs with good diastereoselectivity, furnishing phenylglycine derivatives (Scheme 153D).^932^ It is worth mentioning that the same reaction does not work at all on the corresponding non-fluorinated substrates. A dehydrogenative allylation reaction was reported by Tanaka and Shibata using terminal olefins and Cu/air as oxidant (Scheme 153E). The reaction does not require leaving groups in the olefin, thus increasing the atom economy of the process. However, it has several disadvantages: (i) the exotic Cp^E^ ligand [1,3-bis(ethoxycarbonyl)cyclopentadienyl] is necessary for the reaction to proceed; (ii) a large excess of the substrate (3 equiv.) is required to prevent difunctionalisation in non *ortho*-substitutes substrates; (iii) many of the reported yields remain below the 70% threshold; and (iv) the reaction is slow (60 h, Scheme 153E).^933^

Addition to 1,1-dialkylallenes under Co catalysis provides olefinated arenes, although only medium yields were obtained using pyrazole as DG (Scheme 153F). Olefination was also performed *via* dehydrogenative coupling with acrylates with a Ru catalyst under aerobic conditions (Scheme 153G).^934^ Under these conditions, selective mono- or difunctionalisation was possible, only using different (small) excess of acrylates. Few examples were reported, but a similar reaction was demonstrated also for olefination with styrenes.^934^ Sundararaju published in 2017 a study on the reaction of 1-phenyl-1*H*-pyrazoles with propargyl alcohols, leading to the corresponding allenylated product (Scheme 153H).^935^ Whereas mono-functionalised products could be obtained selectively in good yields only on *ortho*/*meta*-substituted substrates, difunctionalisation was obtained on *para*-substituted arenes.

Two protocols for the aminocarbonylation of 1-phenyl-1*H*-pyrazoles, both exploiting Co catalysis and isocyanates as reactants, were reported independently by Ackermann and Ellmann in 2015 (Scheme 153I).^936^,^937^ Ackermann's protocol could also be applied to heterocyclic substrates (thiophene and furan) and to olefins, but only aromatic isocyanates were used. Ellman's protocol could instead be used with aliphatic isocyanates (2 examples), and generally provided higher yields, but 2 equiv. of the substrate were required.

Regarding C–heteroatom bond formation, selenation with phenylselenyl chloride was reported by Zhang (Scheme 153J),^938^ and halogenation by Wang and Hierso. Wang used Na halides and Rh catalysis for iodination, bromination and chlorination of a few 1-phenyl-1*H*-pyrazoles (Scheme 153K),^772^ while fluorination could be achieved using NFSI with Pd catalysis, as reported by Hierso (Scheme 153L).^939^ Notably, this functionalisation proved to be fairly substrate-dependent, requiring varying equivalents of NFSI or leading to mono or difluorinated products depending on the substitution pattern.

Acetoxylation and iodination were finally reported using a heavily functionalised pyrazole as DG. 4-Methyl-1,5-diaryl-1*H*-pyrazole-3-ethylcarboxylate was used as a model representing a class of pharmaceutically relevant substrates (Scheme 153M).^940^ Selective monoacetoxylation (due to steric hindrance) using Pd(OAc)_2_/PIDA/AcOH was achieved in good yields on a number of substrates, including aliphatic substrates (1 example). The corresponding iodinated product was obtained using iodoacetic acid instead under the same conditions.^940^ It is noteworthy that the presence of a EWG substituent on the substrate reduces the yields by 30–40% for both transformations.

Tandem C–H functionalisation/cyclisation processes similar to those reported for pyridine DGs (see Schemes 112 and 115) have also been developed for pyrazoles. Yi and Xu reported the late stage modification of the drug Edaravone *via* alkylation/cyclisation with Meldrum's acid diazo derivative (Scheme 153N).^941^ The reaction of 1-phenyl-1*H*-pyrazole with internal alkynes, apart from giving the typical olefination product, also results in neutral or ionic cyclisation products similar to those observed with pyridines and other heterocycles (Scheme 153O). In this case three different cyclisation products can be obtained, including ‘rollover’-derived products, strongly depending on the reaction conditions and the identity of the alkyne.^942^,^943^

**Scheme 154:**
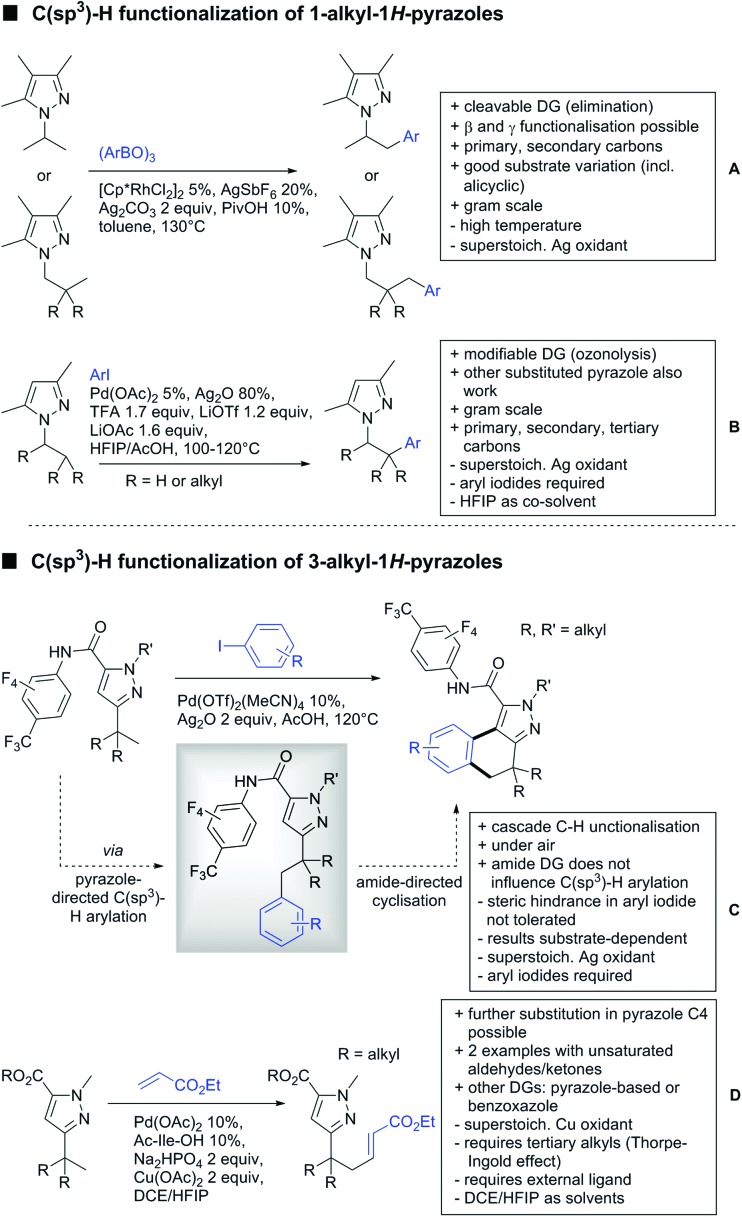
Pyrazole-directed C(sp^3^)–H functionalisation.

Interestingly, a couple of C(sp^3^)–H bond functionalisation studies have been recently reported using pyrazole DGs. Using 3,4,5-trimethyl-1*H*-pyrazole as DG, Zhao demonstrated that both β and γ arylation is possible using Rh catalysis and aryl boroxines (Scheme 154A).^928^ While arylation at the β position is to be expected because of the formation of a 5-membered metallacycle intermediate, γ-functionalisation *via* 6-membered metallacycle selectively occurred when a tertiary or quaternary carbon in β was present.^928^ Another notable feature is the absence of any diarylation products, often observed when multiple functionalisable positions are available. More recently, Daugulis reported a similar arylation, but using Pd(OAc)_2_ and aryl iodides (Scheme 154B).^926^ A notable feature is the possibility to arylate secondary and even tertiary positions (1 example on adamantane substrate).

Although a few papers have appeared in the literature about C(sp^2^)–H functionalisation of 3-aryl-1*H*-pyrazole derivatives,^944^,^945^ these are mostly related to olefination/cyclisation processes similar to those previously described, and will therefore not explicitly be discussed here. Nonetheless, two reports by Yu's and Stamos’ research groups on C(sp^3^)–H functionalisation have appeared. A domino process was developed where an initial C(sp^3^)–H functionalisation was promoted by a pyrazole DG, followed by an intramolecular, dehydrogenative, amide-directed C(sp^2^)–C(sp^2^) bond formation (Scheme 154C).^946^ The use of two different DGs for two different activations in cascade is a very innovative and useful concept for synthetic applications. The following year a C(sp^3^)–H olefination of 3-alkyl-1*H*-pyrazoles was reported by the same authors using ethyl acrylate and analogous reactants (Scheme 154D).^947^ To date this is the only example of pyrazole-directed C(sp^3^)–H olefination.

### 15.6. 1,2,3-Triazole as DG

Due to the easy access to triazoles, these heterocycles have also been used as DGs. Substrates can be attached to the DG at two different positions: (i) attached to the C4 atom of the DG (1,4-disubstituted triazoles, obtained by click chemistry), or (ii) attached to the N2 atom (2-monosubstituted triazoles, obtained *via* other methods).

#### 15.6.1. 1,4-Disubstituted 1,2,3-triazoles

**Scheme 155:**
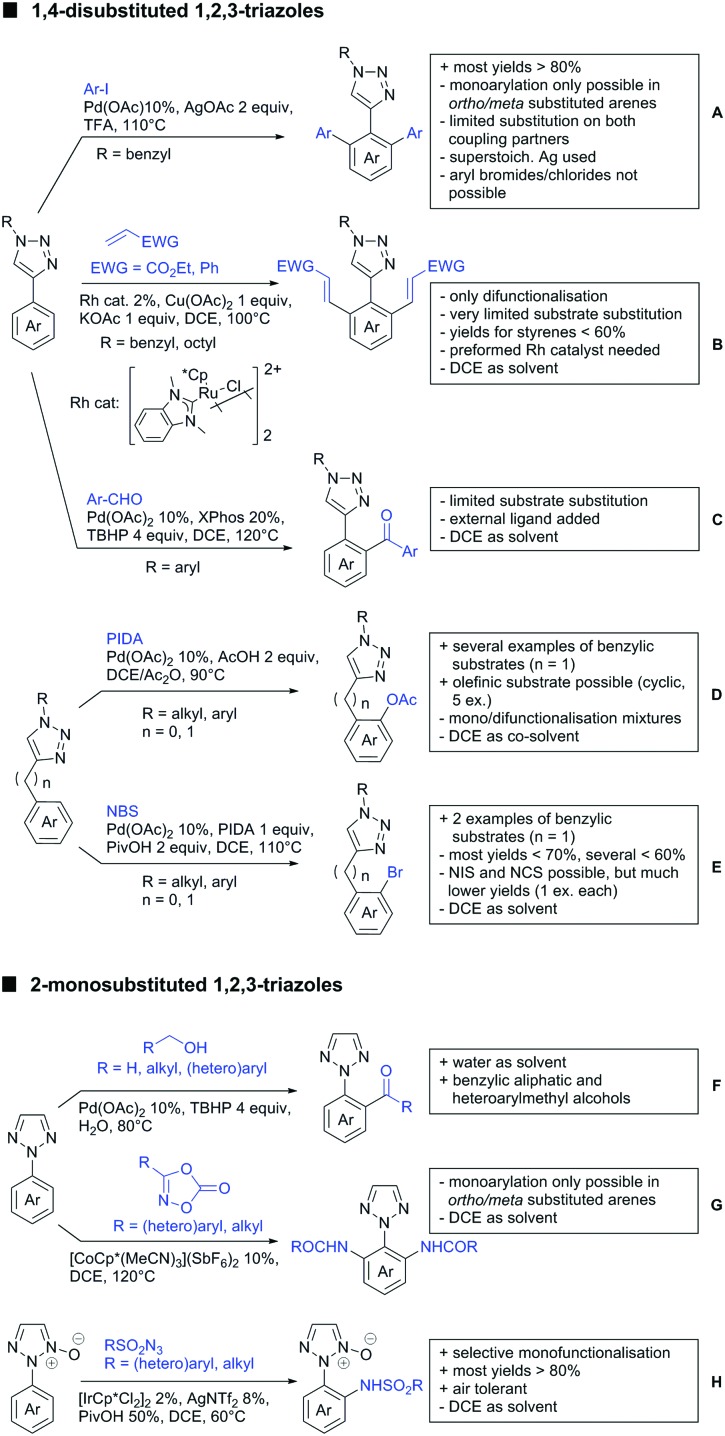
1,2,3-Triazoles as DGs.

C(sp^2^)–H arylation and olefination on the aromatic ring in C4 were reported by Jiang and Wu/Chen *via* Pd-catalysed reaction with aryl iodides and Rh-catalysed Fujiwara–Moritani coupling with acrylates. Although the reported yields are generally good, only difunctionalisation could be obtained (unless *ortho*/*meta* substituents were present in the substrate) (Scheme 155A and B).^948^,^949^ Acylation was reported using the classical Pd/TBHP method (Scheme 155C),^950^ while acyloxylation and halogenation were disclosed recently by Correa and co-workers, making use of Pd/PIDA and Pd/NBS systems (Scheme 155D and E).^951^,^952^ While the acyloxylation gave rise to mixtures of mono- and difunctionalised products in non *ortho*/*meta* substituted substrates, this was not observed for the halogenation, and selective monofunctionalisation was possible. Notably, in both cases, C(sp^2^)–H functionalisation of benzylic substrates (formation of a 6-membered palladacycle) was also demonstrated, and occurred with yields generally comparable to those obtained for aryl substrates.^951^,^952^

The selective functionalisation of the aryl ring in the C4 position of the triazole in the presence of a second aryl substituent in N1 (as for R = aryl in Scheme 155C–E) is suggested to be due to the higher electron density on the N3 in comparison to N2, which promotes selective coordination to this N atom and, consequently, selective functionalisation of the aryl ring in C4.^950^,^953^

#### 15.6.2. 2-Monosubstituted 1,2,3-triazoles

This class of compounds can be prepared using a variety of methods,^954^ and has been subjected to C–H functionalisation studies in a few recent examples.

Acylation in water using Pd/TBHP was reported by Jain (Scheme 155F).^955^ Interestingly, benzyl alcohols were used directly as reactant in this protocol, with oxidation to aldehyde (and formation of the acyl radical) occurring *in situ*. Importantly, heterocyclic-substituted (thiophene, furan) methylalcohols could also be used in this protocol. These substrates were also used in amidation reactions.

One protocol appeared making use of dioxazolones under Co catalysis. (Scheme 155G).^956^ Unfortunately, this reaction resulted in mixtures of mono- and difunctionalised products unless *ortho* or *meta* substituted substrates were employed. The same issue was observed using sulphonyl azides as reactants with Ru catalysts.^957^ In this respect, an interesting contribution came from Wu and Cui, who reported the use of triazole *N*-oxide as DG (under Ir catalysis) to suppress the undesired difunctionalisation due to the increased steric hindrance (Scheme 155H).^958^

### 15.7. 9*H*-Purines and analogues as DG

**Scheme 156:**
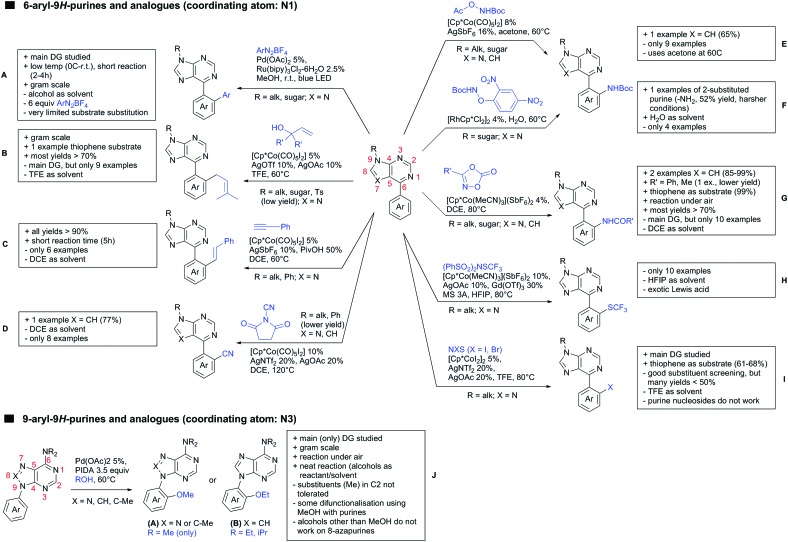
Purine-directed C–H functionalisations.

Purine-based compounds are an important class of biologically active molecules, and the modification of purine-containing starting materials has recently been explored in C–H functionalisation chemistry. The state of the art of the use of purines as both substrates and DGs in C–H functionalisation before 2015 has been summarised by Kapdi/Fairlamb.^959^ In this section the use of purine as heterocyclic DGs in the last few years is summarised. As most of the papers included in this section have already appeared in previous chapters (other heterocyclic DGs), the present section has mostly the purpose of collecting all the transformations together from a practical point of view for the reader, given the importance of the core structure, but little further discussion will be added. It is important to note that purines are mostly tested as secondary DG, therefore no specific optimisation and only few examples are typically reported. If not specified in Scheme 156, yields are around 60–70%; in case of considerably different results (higher or lower yields), these are specifically mentioned in the scheme.

Several types of functionalisation have been studied on 6-aryl-9*H*-purines, where the N1 atom of the pyrimidine ring acts as the coordinating moiety. Arylation with diazonium salts under photoredox catalysis was reported by Guo. The reaction worked nicely on a few purines and nucleosides, despite requiring 6 equiv. of the diazonium reactant and the limited substrate substitution investigated (Scheme 156A).^960^ Co-catalysed allylation and olefination were reported by Matsunaga/Yoshino and Yu/Chen respectively (Scheme 156B and C).^688^,^961^ Both these processes occurred in higher yields than the average 60–70% for these substrates, but only few examples were reported. Cyanation could be performed using *N*-cyanosuccinimide and Co catalysis, according to a protocol developed by Chang (Scheme 156D).^711^

Concerning the formation of C–heteroatom bonds in 6-aryl-9*H*-purines, three amidation methods were reported using electrophilic *N*-Boc *O*-acyl hydroxylamines or dioxazolones (Scheme 156E–G).^747^,^748^,^753^ This last procedure, in particular, furnished high yields, and offers the possibility of thiophene functionalisation (Scheme 156G). Finally, trifluoromethylthiolation^727^ and halogenation^962^ were reported by Matsunaga/Yoshino and Pawar, both under Co catalysis (Scheme 156H and I). A good substrate screening was investigated for the halogenation (especially for the iodination), allowing a comparison of the results. This shows that *para* EWG substituents on the aryl ring of the substrate generally result in much lower yields than *para* EDG substituents.^962^

A rare example of functionalisation on 9-aryl-9*H*-purines (coordinating atom: N3) was reported by Du. Alkoxylation was studied under Pd catalysis using alcohols as reactants/solvents and PIDA as oxidant. While methoxylation was successfully achieved on the 8-azapurine analogue (Scheme 156J, product **A**), ethoxylation did not occur on such substrate, and standard purines had to be used (Scheme 156J, Product **B**).^963^

### 15.8. 7-Azaindoles as DG

7-Azaindoles have been used several times as DGs, due to their presence in a number of biologically active compounds. An important aspect of this DG is the relatively broad functional group tolerance on its core, which ranges from halides, alkyl chains, olefins, carbonyls, to (hetero)arenes.

**Scheme 157:**
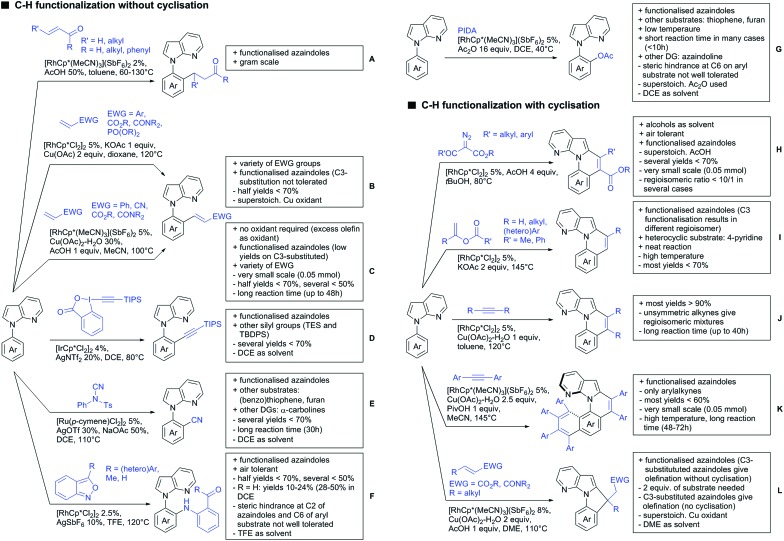
C–H functionalisation directed by 7-azaindole moieties.

A number of functionalisations have been reported using procedures similar to those for other DGs, exclusively under Rh (in one case Ir) catalysis. Among the alkylation and olefination with α,β-unsaturated moieties (Scheme 157A–C),^964^–^966^ an interesting cross-dehydrogenative olefination procedure which does not require the presence of a stoichiometric oxidant (as is usually the case), was reported by Dong (Scheme 157C).^966^ In this reaction the Rh(iii) hydride intermediate formed at the final stage of the olefination process reacts with the acid, releasing H_2_. This is in turn suggested to react with the excess of unsaturated reactant (2 equiv.), which acts as a hydrogen acceptor.^966^ Another version of this transformation (not shown in scheme), was reported by Miura and Satoh. Despite the use of superstoichiometric Ag as oxidant and DME as solvent, this method allows fast reactions (3–6 h) under mild conditions (40–90 °C), giving generally good to excellent results.^967^

Reactions with TIPS-EBX, *N*-cyano-*N*-phenyl-*p*-toluenesulfonamide, and anthranils result in *ortho* alkynylation, cyanation and amination as described in Scheme 157D–F.^968^–^970^ Notable is the use of heterocyclic substrates such as (benzo)thiophenes and furans in the cyanation protocol.^969^ One example of C–O bond formation was reported by Deb using PIDA and Rh catalysis. In contrast to many similar procedures employing Pd catalysis, the use of Rh allowed the reaction to run at a low temperature (40 °C) furnishing good yields often in a short time (Scheme 157G).^971^

The Dong group reported four procedures for the cascade alkylation–cyclisation or olefination–cyclisation, where a “rollover” mechanism after the initial C–H activation results in the formation of π-conjugated fused systems (see Scheme 115, bottom). The group-directed *ortho*-alkylation with diazocompounds or *via* addition to alkenyl esters, followed by intramolecular activation of the azaindole C2, and finally by dehydration or β-H elimination, results in the products in Scheme 157H and I.^972^,^973^ The reaction with alkynes, occurring as previously reported for other DGs, was also investigated (Scheme 157J and K).^974^,^975^ Interesting in this respect is the reaction with 3 equiv. of alkyne, which results in both *ortho* olefination–cyclisation and arene homologation, ultimately furnishing azahelicenes, interesting compounds for their fluorescent properties.^975^

Finally, the reaction with electron-deficient alkenes resulted in an oxidative olefination, followed by rollover and intramolecular Michael addition, giving the compounds shown in Scheme 157L.^976^ An interesting aspect is that steric hindrance at the C3 position of the azaindole DG prevents the final intramolecular cyclisation, selectively furnishing the olefination product.^976^

### 15.9. Oxadiazoles and benzisoxazoles as DGs

In the same way that oximes can act as internal oxidants when used as DGs (see Section 12), *via* cleavage of the N–O bond, also 1,2,4-oxadiazoles and 1,2-benzisoxazoles (cyclic oximes equivalents) have been used as DG/internal oxidant combinations, in particular for the synthesis of isoquinoline derivatives upon reaction with alkynes.

**Scheme 158:**
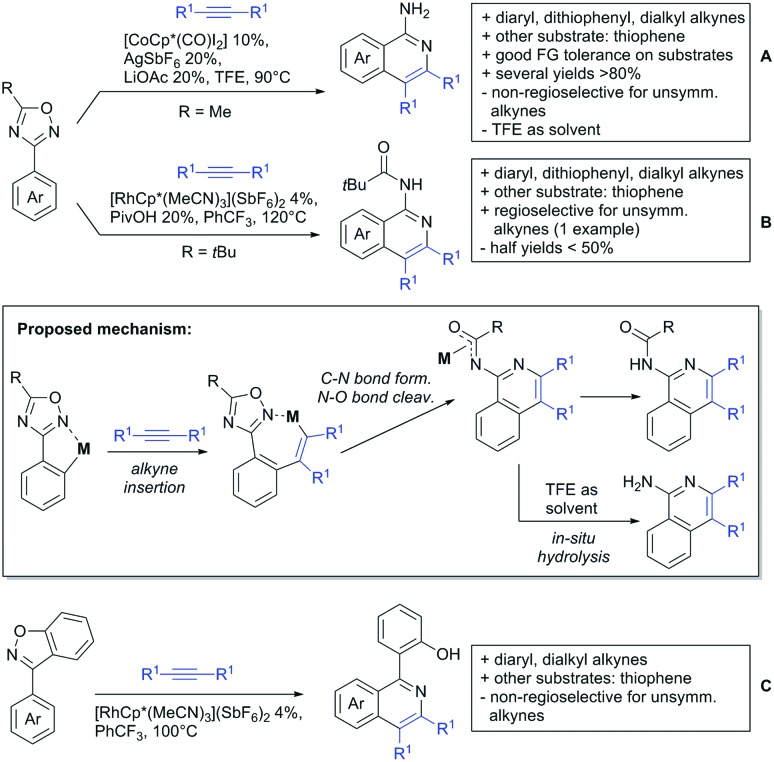
Oxadiazoles and benzisoxazoles as DGs.

Zhu and Miura independently reported in 2017 the use of 1,2,4-oxadiazoles, leading to 1-aminoisoquinolines and 1-acylaminoisoquinolines respectively, using Co and Rh catalysis^977^,^978^ (Scheme 158A and B). Either diaryl, dithiophenyl, or dialkyl alkynes could be used as coupling partners, while terminal alkynes seem unsuitable for this reaction.^978^ Interestingly, while in the Co-catalysed reaction the use of unsymmetrical alkynes gave rise to regioisomeric mixtures, under Rh catalysis only one regioisomer was obtained. The mechanism proposed for this transformation involves the insertion of the alkyne into the initial metallacycle formed, followed by a concerted or stepwise C–N bond formation/N–O bond cleavage, leading to the 1-acylaminoisoquinoline product. The presence of TFE in Zhu's protocol provides *in situ* hydrolysis and formation of the unprotected 1-amino compound.^977^

Zhu also reported analogous reactions employing 1,2,4-oxadiazolones as DGs. In this case the N–O cleavage results in the elimination of CO_2_ from the reaction product.^979^,^980^

In 2017 the use of 1,2-benzisoxazoles as DGs for a similar reaction with alkynes, was disclosed by Miura. With this procedure, isoquinolines functionalised in C1 with a phenolic moiety were formed (Scheme 158C).^981^ The mechanism suggested is analogous to that proposed above.

### 15.10. Oxazolines and benzoxazoles as DGs

**Scheme 159:**
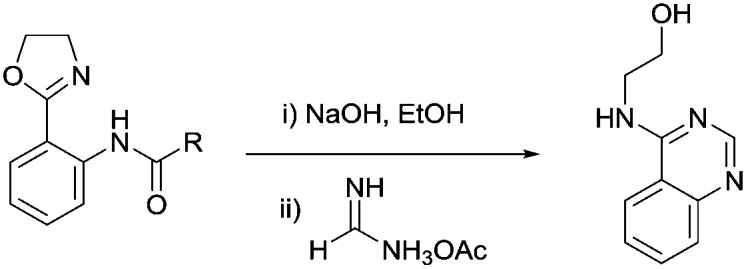
Ring-opening/cyclisation involving oxazoline DGs.

Oxazolines have been used a few times as DGs in C–H functionalisation. One of the reasons is that these functionalities can be easily introduced from the corresponding carboxylic acid, *via* condensation with 2-aminoalcohols, and can also be easily cleaved to return to the original carboxylic acid using a number of procedures: (i) strongly acidic or basic hydrolysis; (ii) activation *via N*-chlorination, sulphonylation or acylation, followed by hydrolysis (milder conditions). The oxazoline ring can also be reductively cleaved to aldehydes using: (i) *N*-quaternization, followed by reduction with NaBH_4_ and hydrolysis; (ii) reduction in the presence of NiCl_2_, followed by acidic hydrolysis.^982^ These and other methods are nicely summarised in a review by Meyers,^983^ and will therefore not be discussed in detail here. An interesting one-pot hydrolysis/cyclisation method, first discovered by Harrity, for *ortho*-amidated 2-aryloxazolines involves basic hydrolysis of the amide, and subsequent cyclisation with formamidine acetate, with consequent opening of the oxazoline ring (Scheme 159).^754^,^984^

**Scheme 160:**
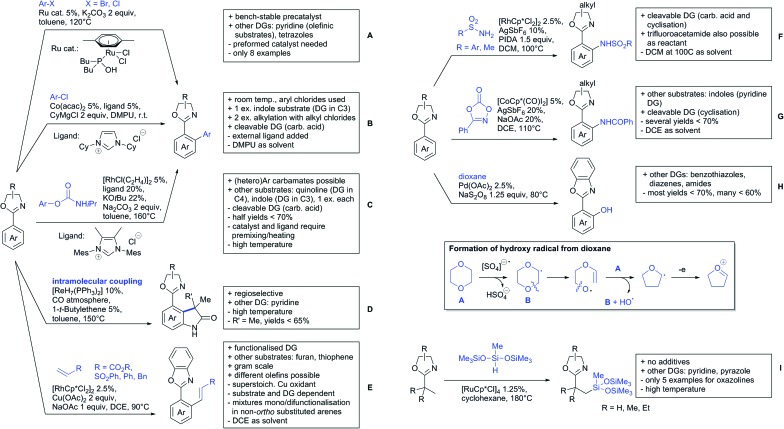
Oxazolines and benzoxazoles as DGs.

Sparse examples of oxazolines as DGs are generally reported in papers mostly dealing with other DGs, but a few specific investigations have been recently published. Arylation with aryl halides or aryl carbamates was reported by Ackermann and Chatani using Ru, Co or Rh catalysis (Scheme 160A–C),^653^,^985^,^986^ typically requiring preformed catalysts or the addition of a ligand to reach good results. An example of intramolecular alkylation leading to the formation of heterocycles was reported by Zhu (Scheme 160D).^177^ Interestingly, the intramolecular addition to an unsaturated moiety occurs regiospecifically in α-position to the carbonyl, instead of β, although the origin of for such selectivity was not investigated. Olefination by Fujiwara–Moritani coupling with acrylates and benzoxazoles as DG was reported by Hou under Rh catalysis (Scheme 160E).^987^ This reaction proved generally substrate- and DG-dependent, with yields ranging from 33 to 95%, and no monoselectivity could be achieved in non *ortho*-functionalised substrates. Nonetheless, this protocol was efficient for the coupling of different classes of olefins, including allylbenzene (unusual reactant for this type of reaction).^987^ Two protocols for amidation were reported by Harrity and Ackermann, using amides or dioxazolones respectively (Scheme 160F and G).^754^,^984^,^988^ A hydroxylation reaction on 2-arylbenzoxazoles was demonstrated by Chakraborti in 2015, where the hydroxy radical was generated *in situ* from 1,4-dioxane, used as solvent (Scheme 160H).^989^ Interestingly, this protocol only required 2.5% Pd loading, considerably lower than other analogous procedures. Finally, a C(sp^3^)–H silylation on 2-ethyloxazoline derivatives was reported by Murata and co-workers in 2016 (Scheme 160I).^990^ The reaction was performed at 180 °C with HSi(Me)(OSiMe_3_)_2_. This reaction did not require the use of a hydrogen acceptor, probably due to the high temperature used.

### 15.11. Benzothiazoles as DGs

A handful of papers on the use of benzothiazole DGs have appeared in the recent literature. The cleavage/ring opening of 2-functionalised (carbon chains/rings) benzothiazoles have been reported in a few cases,^991^–^995^ but these protocols have not been used in the context of DG cleavage in C–H functionalisation.

**Scheme 161:**
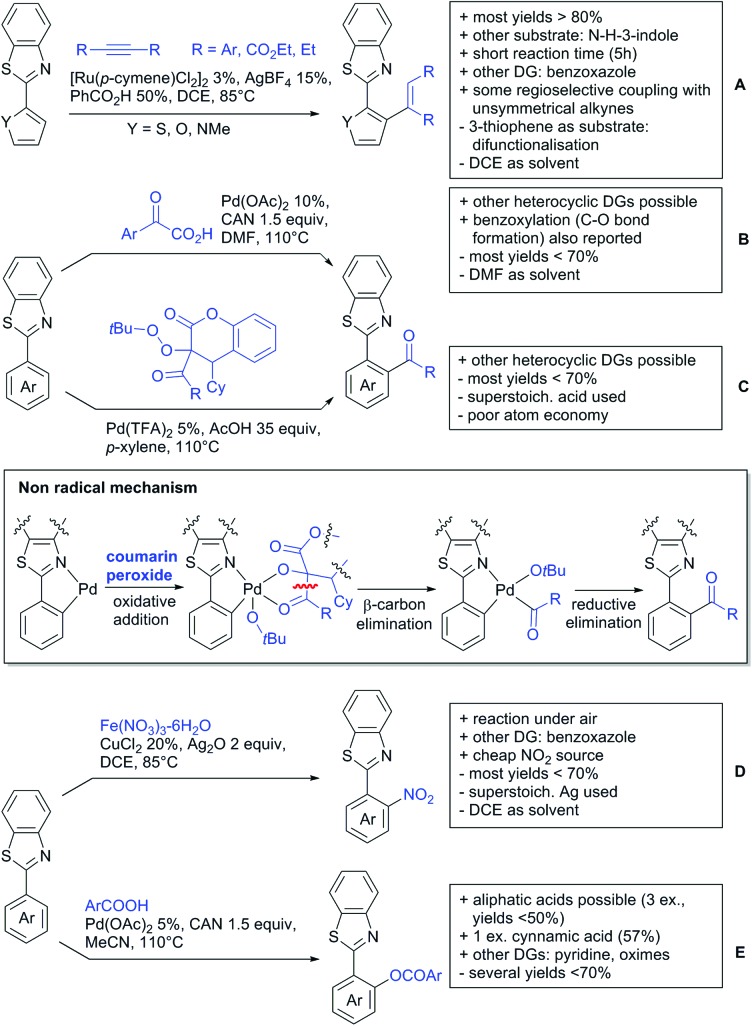
Benzothiazoles as DGs.

Chakraborti investigated the use of benzothiazole DGs specifically for the olefination of heterocycles with alkynes using Ru catalysis (Scheme 161A).^996^ This protocol resulted in good yields of the desired olefinated products, and works efficiently on 2-thienyl, 2-furyl, 2-pyrrolyl and 3-indolyl substrates, and a few examples of regioselective olefination with unsymmetrical alkynes were demonstrated as well.^996^ Acylation of benzothiazole-equipped arenes was reported using classical Pd/oxidant/acyl radical precursor methods (mechanism in Scheme 118).^762^,^997^ Of note here, is the use of CAN (ceric ammonium nitrate) as a new alternative oxidant instead of peroxides or O_2_. Patel reported the use of CAN with α-ketocarboxylic acids for C–H benzoylation of 2-arylbenzothiazoles and a variety of other heterocyclic compounds (Scheme 161B).^762^ Another method sees the use of acylperoxycoumarins as acylating agents (Scheme 161C).^998^ Strangely, this reaction does not proceed *via* a (free) radical pathway, as demonstrated by radical trapping experiments, but instead occurs *via* a β-carbon elimination from an alkoxy-coordinated Pd species, which delivers the acyl groups directly to the metal, and a subsequent reductive elimination releases the acylated product.^998^ An example of Cu-catalysed C–H nitration was reported making use of Fe(NO_3_)_3_ as nitro group source (Scheme 161D).^999^ Although utilising a cheap nitro source, the reaction still requires 2 equiv. of Ag_2_O (4 equiv. of Ag in total). With a similar protocol to that shown in Scheme 161B, the benzoxylation of 2-aryl benzothiazoles occurs using benzoic acids (Scheme 161E).^762^ Mechanistically, a benzoxy radical is generated by CAN, and subsequently coordinates to the intermediate palladacycle, which ultimately results in its introduction onto the substrate.

## 16. Other DGs employed in C–H functionalisation

The synthetic value of C–H functionalisation chemistry is strongly related to the modularity of the DG used. Ideally, a DG should be traceless or easily convertible into an array of common functionalities. An alternative approach consists in direct utilisation of functionalities naturally present on a molecule of interest to play the role of DG. In such a case particularly sustainable transformations take place as installation and removal of the DG is not required anymore. Following these considerations, over the last decade intensive efforts have been focused on designing DGs based on heteroatoms such as P, S, Si, O, N, *etc.* Notably, these modern DGs are generally easily cleavable or may also remain present in the final product. An additional advantage of these scaffolds is their potentially stereogenic character, and thus diastereoselective C–H functionalisation reactions can be explored, even without the use of external chiral ligands. In the following sections, recent advances on the use of these increasingly important DGs are summarised. Due to their less common nature though, relative to the above reported DGs, important publications from before 2015 will also be included.

### 16.1. Thioether as DG

Due to the strong coordination properties of the sulfur compounds, and in particular thioethers, this motif has been considered, for years, an inefficient DG, leading to catalyst poisoning. However, between 2012 and 2015 few reports have been published showing that the thio unit can be astutely employed in the context of directed functionalisation,^1000^–^1003^ and these pioneering contributions have already been summarized.^17^ More recently, due to a widespread presence of thio groups in natural products and biologically active scaffolds, several research groups reported complementary studies.

**Scheme 162:**
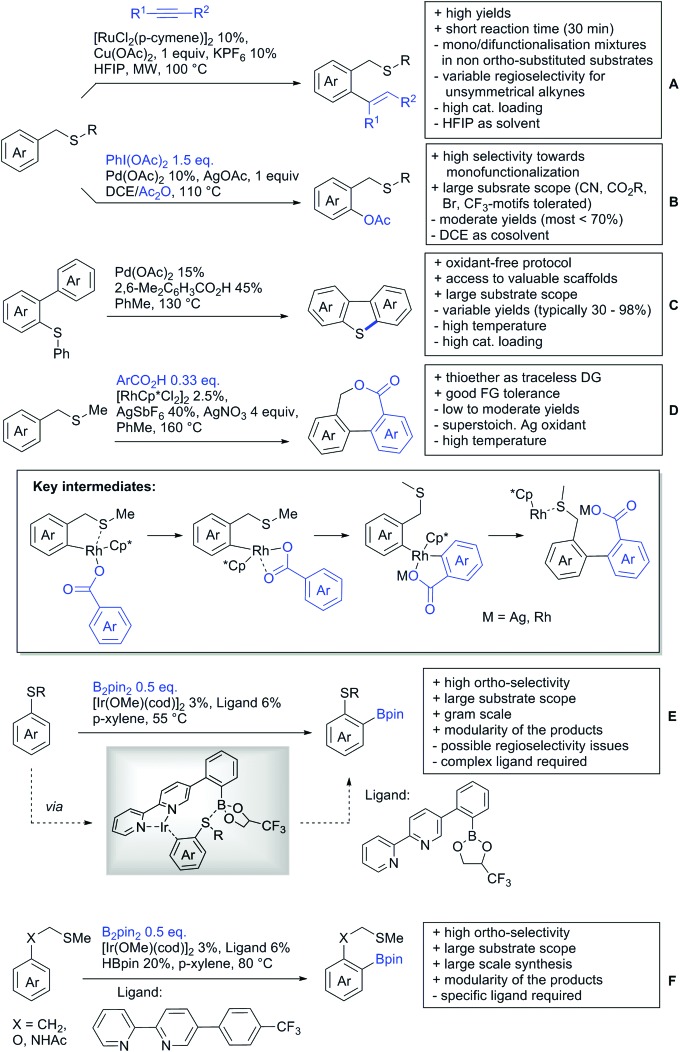
C(sp^2^)–H functionalisation using thioether-based DGs.

In 2015, Urriolabeitia expanded the scope of previously known Rh-catalysed direct olefinations of benzylthioethers^1000^,^1002^,^1003^ by developing a related C–H hydroarylation catalysed by the [Ru(*p*-cymene)Cl_2_]_2_ complex (Scheme 162A).^1004^ Installation of the thioether motif at the benzylic position is crucial to allow efficient formation of a 5-membered ruthenacyclic intermediate. The optimization study revealed that choice of a protic but not too acidic solvent such as HFIP is essential for a high efficiency. Unfortunately, moderate selectivity between mono- and diarylation is observed, calling for exemplification of this methodology on *ortho*-substituted substrates. Interestingly, the reaction occurs smoothly under microwave heating (100 °C for 30 min), which enables to drastically reduce the reaction time (typically 24 h) required under conventional heating. The reaction well tolerates several aliphatic symmetric alkynes and the coupling occurs as a syn addition, delivering *E*-trisubstituted vinylic fragments (Scheme 162A).^1004^ Besides, a regioselective transformation was achieved when using 1-phenylpropyne coupling partner (9 : 1 isomeric ratio) although a significant drop in regioselectivity was witnessed for 2-hexyne. The reaction shows good FG tolerance on the substrate, both on the aromatic ring and the *S*-substituent (aliphatic and aromatic motifs are efficient), delivering the desired vinylic compounds in good to excellent yields (16 examples, 54–96% yield).

To further expand the scope of molecular scaffolds accessible *via* thioether-directed C–H functionalisation Liu and Zhang reported a direct acetoxylation protocol (Scheme 162B).^1005^ As in the previous example, the reaction requires benzothiophene substrates but the DG may also be installed at homobenzylic position (formation of 6-membered metallacyclic intermediate). Using standard Pd(OAc)_2_ catalyst, in combination with Ac_2_O, PIDA, and AgOAc, the desired C–O bond formation takes place. Interestingly, this protocol permits selective mono-functionalisation and a remarkable array of FGs is well tolerated, including bromine and chlorine atoms, but also coordinating moieties such as nitrile, ester and OCF_3_ moiety. Yields are generally moderate to good (21 examples, 40–84% yield). Importantly, the DG may be removed from the final product either using RANEY® Ni to furnish the corresponding *o*-tolyl acetate, or by addition of *m*-CPBA to convert the thio-motif into an aldehyde.

Thiol-directed C–H activation can also be astutely used to access benzo-fused thiophenes *via* intramolecular C–H arylation, as illustrated in 2016 by Tobisu and Chatani (Scheme 162C).^1006^ The authors discovered that biphenylsulfide, in the presence of Pd(OAc)_2_ and under carboxylic acid assistance (2,6-Me_2_C_6_H_3_CO_2_H used) undergoes a tandem C–H cleavage/intramolecular C–S bond formation/S–C cleavage, delivering the desired heterocyclic products under oxidant-free conditions. This operationally simple and high-yielding protocol using readily accessible starting materials and reactants gives access to a large panel of unsymmetrical dibenzothiophenes, important scaffolds in advanced material science and bioactive molecules. It is noteworthy that this oxidant-free protocol allows the suppression of the usually observed reactivity for this type of compounds, *i.e.* C–H/C–H intramolecular arylation to give isomeric benzothiophene compounds.^1007^

In addition to this intramolecular thioether-directed arylation, Shi *et al.* developed a very interesting intermolecular coupling between benzylthioethers and carboxylic acids, yielding dibenzooxepinones (Scheme 162D).^1008^ This RhCp*-catalysed transformation involves initial C–H metallation of the thiophene substrate, followed by carboxylate-assisted second C–H activation and reductive elimination, and Rh- or Ag-assisted cyclisation and C–S cleavage. Accordingly, the thiophene moiety is clearly a traceless DG. Although rather harsh reaction conditions required (use of 4 equivalents of AgNO_3_ and reaction temperature of 160 °C) and the relatively low yields (28–75%), the reaction still well tolerates substituents such as Cl, Br, but also strongly electron withdrawing CF_3_ or ester moieties.

In 2017 Kuninobu and Kanai designed an interesting *ortho*-selective borylation of arylsulfides.^1009^ In this case the thioether DG is directly installed on the aromatic ring as, contrary to the previous examples, the S atom does not coordinate directly to the Ir catalyst, but generates a Lewis acid–base intermediate with a bipyridine-ligated boron species (Scheme 162E).^1009^ Such an unusual interaction directs the borylation highly selectively at the *ortho*-position. Fine-tuning of the catalytic system and ligand design allowed the development of a truly efficient and versatile transformation, occurring under mild reaction conditions and compatible with a range of functionalised substrates. Notably, the reaction is equally efficient at gram scale. As the newly introduced Bpin substituent is a handle for further modification of the molecular scaffolds, this transformation holds great promise for widespread applications. Few months later the authors further expanded the potential of this transformation by conceiving an *ortho*-borylation of phenol and aniline derivatives (Scheme 162F).^1010^ In this case the methylthiomethyl group installed on hydroxy- or amino-moieties allows, *via* the same type of interaction as above, directing the borylation at the *ortho*-position. As previously, the high regioselectivity observed, efficiency and possible post-modifications of the newly obtained scaffolds, render this coupling attractive, as further illustrated by its application in an expedient synthesis of a Ca receptor modulator.^1010^ The removal of the DG (*i.e.* deprotection of hydroxyl group) occurs smoothly using I_2_ in MeOH, furnishing the corresponding phenol in high yield.

**Scheme 163:**
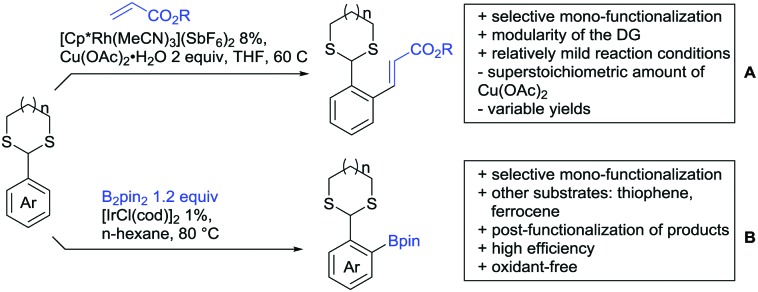
Rare examples of dithiane DGs.

Drawing inspiration from the standard thioether-directed C–H functionalisation, Satoh and Miura disclosed in 2015 the use of dithiane motifs (commonly used as aldehyde protecting group) as an effective DG for Rh-catalysed Fujiwara–Moritani couplings (Scheme 163A).^1011^ This C–C bond forming transformation, occurring under relatively mild conditions (60 °C, no need for acidic additives and use of Cu(OAc)_2_·H_2_O as mild oxidant) is not only highly monoselective but also compatible with an array of functionalised substrates bearing challenging motifs such as nitro, ketone, ether, thioether or halide substituents. The protocol is also compatible with a cyclic sulfide substrate. The synthetic value of this transformation arises from a straightforward post-modification of the dithiane-DG that can be either removed using RANEY®-Ni or converted into aldehyde in the presence of Dess–Martin periodinane.^1011^

Very recently, Li *et al.* used the same DG to achieve an Ir-catalysed *ortho*-borylation (Scheme 163B).^1012^ This ligand-free coupling with B_2_pin_2_ is effective for the direct functionalisation of both aromatic and heteroaromatic moieties, like thiophene and ferrocene backbones. It is worth noting that the employment of the dithiane motif outcompetes thioether DGs as mono-functionalisation occurs selectively, whereas mixtures of mono- and biarylated products are typically generated when thioethers are used. The cleavage of the 1,3-dithiane was achieved using NaI, Fe(acac)_3_ in H_2_O_2_, delivering the corresponding aldehyde.

**Scheme 164:**
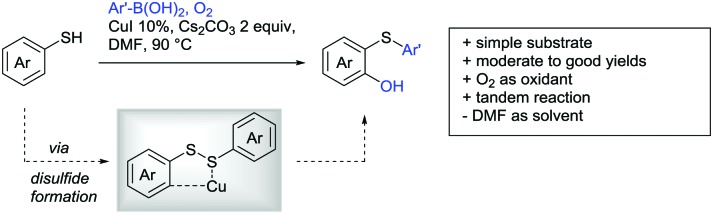
Disulfide-directed C–H hydroxylation.

Further exploring the potential of the thioether motif as DG and drawing inspiration from the observation that benzylthioethers are effective in C–H activation reactions, Wang and Shi described an elegant example of disulfide-directed C–H hydroxylation (Scheme 164).^1013^ The optimized Cu-based catalytic system allows a sequential transformation involving: (1) thiophenol oxidation to disulfide, (2) disulfide-directed *ortho*-hydroxylation and (3) *S*-arylation using boronic acid coupling partner, affording otherwise difficult to access 1,2-thio-phenol derivatives. Remarkably, this complex transformation is rather high-yielding and shows good substrate scope, in particular with regard to halogenated substituents at both coupling partners. Besides, applying the same concept for a synthesis of thiophenol-substituted quinones further showcased the potential of this reaction. Few months later, Wang and Li reported a closely related Cu-catalysed disulfide-directed *ortho*-hydroxylation. In this case disulfide substrates were directly employed, and a Cu–bipy complex was selected as the optimal catalyst.^1014^

### 16.2. Sulfoxide as DG

In addition to thioethers, higher oxidation state sulfur moieties also established themselves as powerful DGs. In particular between 2012 and 2014 several research groups employed with success sulfoxide DGs in Pd,^1015^–^1017^ Rh,^1018^ and Ru^1019^-catalysed C–H activation. Since 2015, few additional key contributions have been published,^29^ mainly relating to the use of enantiopure sulfoxides for stereoselective C–H activation.

**Scheme 165:**
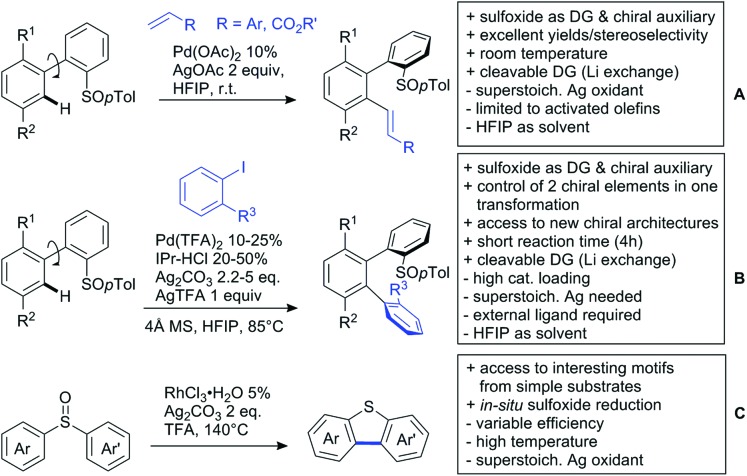
Sulfoxide-directed C–H functionalisation.

In 2013 Leroux and Colobert reported first example of diastereoselective sulfoxide-directed oxidative olefination of biaryl precursors leading to atropoenriched, axially chiral scaffolds.^1020^ This pioneering work clearly showcases the potential of enantiopure sulfinyl, accessible in a large scale from cheap menthol,^1021^ to play the double role of DG and chiral auxiliary. However, both efficiency and stereoselectivity of the catalytic system were rather moderate. A clear improvement was achieved in 2014 and 2016 by Wencel-Delord and Colobert, who discovered that a much more powerful and general transformation is achieved when changing the reaction medium from DCE to HFIP (Scheme 165A).^1022^,^1023^ Under such modified reaction conditions the Fujiwara–Moritani reaction may be conducted at room temperature and using a significantly lower amount of the Ag oxidant (2 equiv. *vs.* 6 equiv. initially required), delivering a large panel of axially chiral biaryls with excellent atroposelectivity and high yields. Besides, as the sulfoxide moiety undergoes exchange with Li bases, trapping of the resulting axially chiral Li species with an array of electrophiles could be performed, delivering variety functionalised biaryls without loss of stereopurity (introduction of functionalities such as aldehydes, Me, OH, CO_2_H, *etc.*).^1022^ This methodology was also further used to perform direct acetoxylation and iodination.^1023^ The synthetic potential of this methodology was further illustrated while designing a novel retrosynthetic route towards biologically active, axially chiral Steganacine scaffolds.^1024^ Remarkably, the synthesis based on this atropo-diastereoselective transformation delivered the direct precursor of the targeted enantiopure molecule in only 10 steps and in very high total yield (42%).

Very recently the concept of sulfoxide-directed atroposelective C–H functionalisation was employed to build up original tridimensional structures, *i.e. ortho*-orientated terphenyl scaffolds with two perfectly controlled chiral axis (Scheme 165B).^1025^ These molecules exhibiting “open clam shell architectures” could be obtained *via* direct arylation of the biaryl-sulfoxide precursors with *ortho*-substituted iodoarenes. During this process two chiral Ar–Ar axes, are induced in a single transformation with very high stereocontrol. Importantly, the traceless character of the sulfoxide, combined with the good FG tolerance of the catalytic system towards the iodoarene partner permits straightforward derivatization into unprecedented bidentate ligands.

Dibenzothiophenes are important molecular motifs in natural products, pharmaceuticals and functional materials. Accordingly, designing of C–H functionalisation-based strategies to access these molecular units has gathered the attention of several research groups. In 2013 Colobert described a Pd-catalysed intramolecular arylation of 2-bromodiarylsulfinyle, delivering dibenzothiophenes *S*-oxides.^1026^ Going one step further, in 2015 Huang and Lin reported an intramolecular two-fold C–H functionalisation of diarylsulfoxides (Scheme 165C).^1027^ Interestingly, under this Rh-based protocol deoxygenation of the newly formed dibenzothiophenes S-oxides occurs *in situ*. Unfortunately, the efficiency of the reaction is strongly affected by the nature of the starting material and harsh reaction conditions are required (140 °C).

### 16.3. Sulfones, sulfonamides & sulfoximines DG

**Scheme 166:**
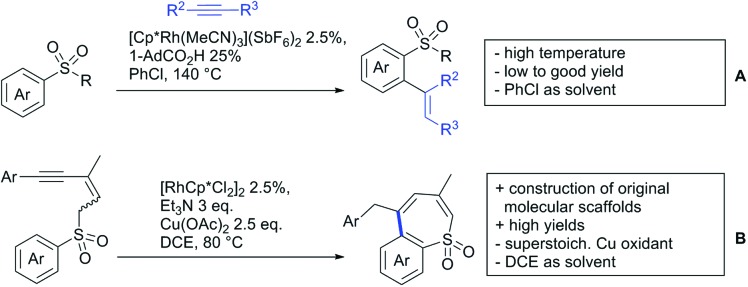
Sulfone-directed C–H functionalisation.

In contrast to thioether and sulfoxides that have been regularly employed as DGs, the use of sulfones in this context has remained fairly unexplored till recently, despite the easy access to such molecules *via* electrophilic sulfonylation of aromatic rings. Initially, the sulfone moiety was used as a handle to install other DGs, as in the case to 2-pyridylsulfonate DG.^1028^ In this case the heterocycle enhances and dictates the metallation event whereas the SO_2_ moiety can be considered as a removable anchor (for examples of such transformations see Section 15.3.4). Satoh and Miura proved however in 2015 that sulfones may act as efficient DGs themselves, allowing *ortho*-selective alkenylation of aromatics (Scheme 166A).^1029^ The catalytic system based on Rh complexes, combined with adamantanecarboxylic acid additive gave good results with internal aromatic and aliphatic alkynes, delivering the desired vinylic products in moderate to good yields, but a high reaction temperature was generally required.

An additional attracting example of sulfone-directed intramolecular transformation was reported in 2015 by Zhou (Scheme 166B).^1030^ This cascade-type reaction involves sulfone-directed metallation, followed by base-assisted 1,5-H shift affording allene intermediates. Insertion into the C–Rh bond finally delivers seven-membered heteroaromatic products such as benzothiepines. This strategy constitutes an interesting pathway towards molecules of interest despite the complexity of the starting materials used.

**Scheme 167:**
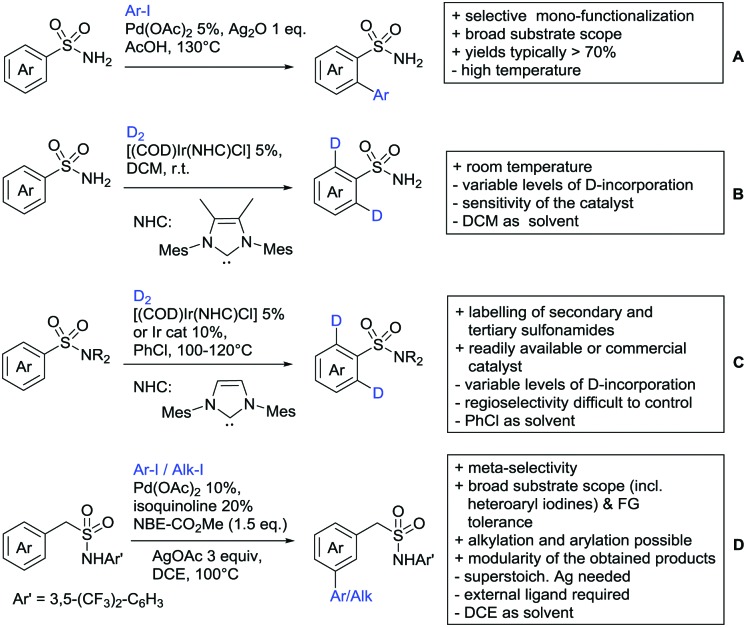
Direct functionalisation using sulfonamides as DG.

Sulfonamide is amongst the most significant pharmacophores, frequently encountered in biologically active scaffolds. However, this weakly coordinating motif has attracted only limited attention from the scientific community working in the field of directed C–H functionalisation. In 2011 Yu *et al.* described a late stage modification of sulfonamide-containing motifs, *via* direct olefination, carboxylation, carbonylation, iodination, arylation and alkylation.^1031^ The variety of sulfonamide-directed transformations was enlarged in 2016 when Singh developed a Pd-catalysed direct arylation using iodoarenes as coupling partners (Scheme 167A).^1032^ This transformation is highly mono-selective, although the products are isolated in moderate to good yields (typically between 60–70%) and prolonged heating at 130 °C in acetic acid is necessary to promote this transformation.

In parallel, Kerr and Tuttle endeavored on designing a catalytic system to regioselectively install deuterium on aromatics (Scheme 167B).^1033^ Following this target they discovered that primary sulfonamides undergo highly selective H/D exchange, when using an Ir–NHC complex. Importantly, D_2_ may be employed as deuterium source and this transformation is compatible with an array of functionalities. Unfortunately, the level of deuterium incorporation is highly dependent on the substitution pattern of the substrates, possibly due to their different solubility in DCM. This protocol could however be successfully used to label pharmaceuticals, such as Celecoxib and Mavacoxib.^1033^ In 2017 the synthetic potential and field of application of this methodology was further expanded by conceiving a closely related deuteration of secondary and tertiary sulfonamides. Atzrodt and Derdau established a protocol based on an Ir catalyst bearing either a monodentate NHC or bidentate NHC/oxazoline ligand (commercially available Burgess catalyst), effective in chlorobenzene at higher temperature (typically 100–120 °C) (Scheme 167C).^1034^ However, as in the previous example, the outcome of the transformation might be difficult to predict in terms of deuterium incorporation and regioselectivity (in particular if other coordinating moieties are present on the substrate or when tertiary sulfonamides are employed).

Very recently, Yu and coworkers described an astute application for sulfonamide motifs installed at the benzylic position to promote *meta*-selective functionalisation (Scheme 167D).^1035^ The regioselectivity switch was reached using modified norbornene (NBE-CO_2_Me: 2-carbomethoxynorbornene) as a transient mediator, while addition of an external isoquinoline ligand was crucial for the efficiency of the catalytic system. Remarkably, both *meta*-arylation and *meta*-alkylations could be performed and the protocol features broad substrate scope as well as excellent FG tolerance. The synthetic value of this reaction was further illustrated by the modularity of the obtained products, which could be easily converted into the corresponding sodium sulfonates, sulfonate esters, other sulfonamides or olefins. Besides, a two-step procedure allows cleavage of the DG under Julia olefination conditions delivering the corresponding styrene.^1035^

**Scheme 168:**
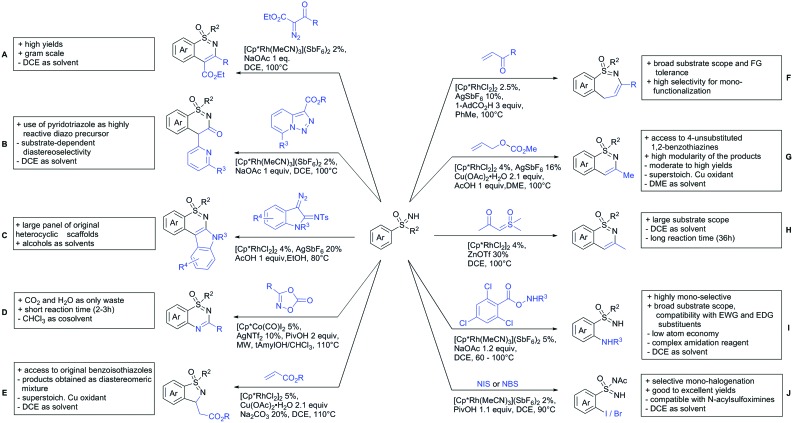
(Free-NH)-sulfoximine-directed C–H functionalisation.

Finally, sulfoximines further expand the portfolio of S-based DGs. In particular, heterocyclic molecules featuring the sulfoximine group are tempting for drug design. Hence, the development of modern, efficient and straightforward pathways to access them is of great importance. Accordingly, several research groups astutely used sulfoximine-directed C–H functionalisation/cyclisation protocols to access an array of benzothiazines congeners. Amongst pioneering contributions in this field, Bolm described in 2013 the Rh-catalysed oxidative annulation of sulfoximines and internal alkynes, delivering 1,2-benzothiazines with fully substituted heterocyclic cores.^1036^ Despite the synthetic value of this methodology, the use of unsymmetrical alkynes raised regioselectivity issues and mixtures of regioisomeric products were generally produced. To tackle this limitation, in 2015 the same research group combined Rh-catalysed sulfoximine-directed C–H alkylation with diazo compounds (Scheme 168A).^1037^ Rewardingly, the reaction between NH-sulfoximines and a large variety of alkyl- and phenyl-containing diazo esters occurred smoothly in presence of a Rh catalyst and NaOAc additive, delivering structurally diversified 1,2-benzothiazines in generally excellent yields. Besides, a scale-up of this reaction was achieved and the newly obtained scaffold could be straightforwardly converted into several added-value congeners.

Following this idea, Lee and coworkers developed an alternative synthesis of 1,2-benzothiazine derivatives (Scheme 168B).^1038^ Using pyridotriazoles as highly reactive diazoimine precursors (see also Scheme 112), pyridine-substituted 1,2-benzothiazines were afforded *via* a domino C–H functionalisation/cyclisation/elimination sequence in good to excellent yields regardless of the substitution pattern of the aromatic substrate. Few months later the same group further expanded the panel of related transformations, now employing 3-diazoindolin-2-imines as coupling partners.^1039^ Under Rh catalysis the desired direct coupling/annulation sequence occurred producing a indolo-1,2-benzothiazines with various substituents at both, the substrate and the indole core (Scheme 168C).^1039^

An efficient cyclometallation event for sulfoximines substrates combined with the high reactivity of the resulting metallacyclic intermediates towards carbene migratory insertion encouraged further elaboration of innovative synthetic routes towards original heterocycles. Accordingly, in 2017 Chen performed direct C–H amidation of sulfoximines using a Co catalyst and 1,4,2-dioxazol-5-ones as coupling partners (Scheme 168D).^1040^ Under optimized microwave assisted conditions, the expected aza-heterocycles were delivered, generating only CO_2_ and H_2_O as waste.

The metallacyclic intermediates can be generally considered as nucleophiles and may hence participate in reactions with various electrophiles, such as Michael acceptors for example. Such reactivity was surveyed by Li and Dong, who synthesized benzoisothiazoles *via* Rh-catalysed coupling between sulfoximines and activated olefins (Scheme 168E).^1041^ Unfortunately, although the original 5-membered heterocyclic products are generally afforded in good yields (typically 70–90%), poor stereoselectivity was observed (usually 1 : 1 ratio), clearly hampering the synthetic potential of the reaction. A complementary reaction delivering original heterocyclic scaffolds was achieved by combining C–H activation and coupling with unsaturated ketones (Scheme 168F).^1042^ The reaction sequence involving formal metal-catalysed functionalisation and subsequent [4+3] annulations produced 7-membered 1,2-benzothiazepines 1-oxides. Broad substrate scope observed for this transformation, quite high efficiency and compatibility with cheap 3d-metal catalysts render this transformation appealing in drug-design science. Finally Bolm illustrated that benzothiazine may also be build-up while performing a Rh(iii)-catalysed domino allylation/oxidative cyclisation between sulfoximines and allyl carbonates (Scheme 168G).^1043^ This transformation is a synthetic route of choice to prepare 4-unsubstituted 1,2-benzothiazines, scaffolds difficult to access when employing diazo-type coupling partners. Noteworthy, both electron rich and electron poor substituents may be introduced on the aromatic ring of the sulfoximine, and several different alkyl *S*-substituents are well tolerated. Thus obtained benzothiazine may also be rapidly diversified into few molecules of interest for pharmaceutical industry. An alternative route towards 4-unsubstituted 1,2-benzothiazines involves coupling between the metallacyclic intermediate and sulfoxonium ylides, used now as novel C2 synthons (Scheme 168H).^1044^ This RhCp*-catalysed transformation was accelerated in presence of Zn(OTf)_2_ additive, is of particular interest if benzothiazines bearing various, aliphatic and aromatic substituents at 3-position are targeted as an array of sulfoxonium yield performed very efficiently in this transformation.

Finally, it should be mentioned that using a standard RhCp*-catalyst, in combination with NH-Boc oxycarbamates, direct amidation of sulfoximines occurs smoothly for both, electron-poor and electron-rich aromatics (Scheme 168I).^1045^ Furthermore, as the newly accessed *ortho*-amidated sulfoximines may be converted into few steps into thiadiazine 1-oxides. In addition, still benefitting from efficient metallation of sulfoximine substrates in presence of RhCp*-catalysts, Bolm *et al.* reported direct C–X coupling (Scheme 168J).^1046^ Indeed, in presence of NIS or NBS as halogenating agents sulfoximes may be directly converted into *ortho*-halogenated congeners in high to excellent yield. This reaction allows therefore late-stage installation of amenable motifs for further diversifications.

**Scheme 169:**
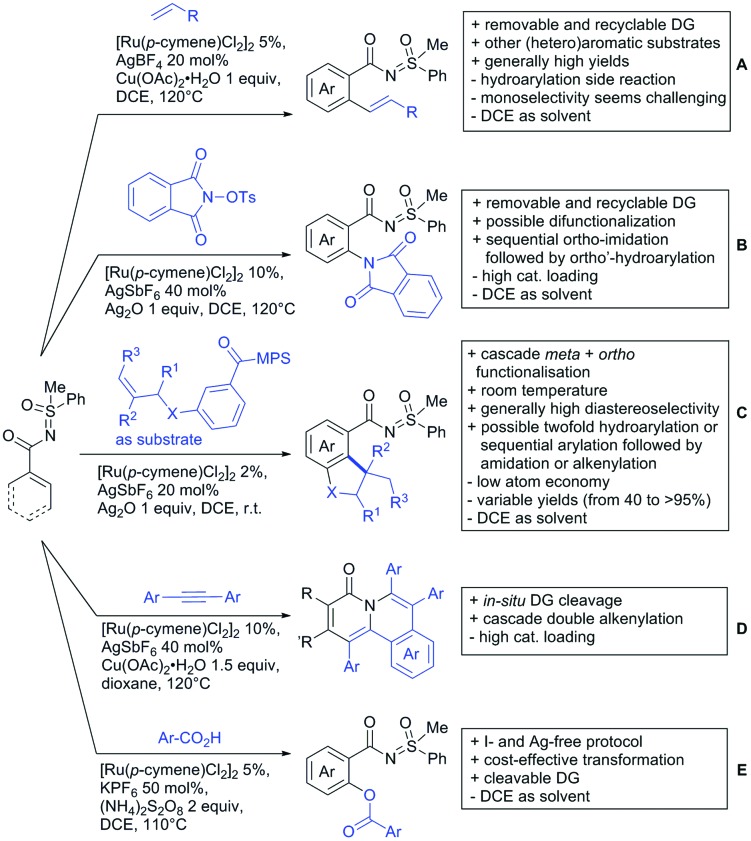
(*N*-Benzoyl)sulfoximine and related DGs.

A further key contribution to the field of sulfoximine-directed C–H activation was made by Sahoo who designed a *S*-methyl-*S*-phenylsulfoximine (MPS) DG.^16^ This motif, straightforwardly installable on carboxylic acids, easily removable (under basic conditions using NaOH in MeOH/H_2_O or acidic conditions using aq. HCl at 80 °C) and recyclable, demonstrated excellent reactivity under Ru- and Pd-catalysis, undergoing efficiently direct hydroxylation^1047^,^1048^ or amidation^1049^ reactions. In 2014 the versatility of this chemistry was further spread by devising a protocol allowing *ortho*- and chemoselective C–H alkenylations (Scheme 169A).^1050^ This oxidative Heck reaction catalysed by Ru(*p*-cymene) complex was compatible with an array of sulfoximine substrates but activated olefines (typically acrylates) were required. Following this exciting research field Sahoo discovered that MPS-equipped substrates, in presence of the same Ru-complex and *N*-protected phthalimides, undergo *ortho*-selective C–N bond formation (Scheme 169B).^1051^ Interestingly, the reaction protocol tolerates several functionalised aromatics (including halo-substituted and N-heteroaroyl ones) and, in presence of a large excess of the phthalimide, double *ortho*,*ortho*′-C–H activation occurs. Subsequently, intramolecular direct heteroarylation of MPS-sulfoximines was disclosed. Sahoo and coworkers installed on an aromatic substrate a judiciously selected olefin tether (Scheme 169C).^1052^ After the Ru-catalysed C–H cleavage, spacial proximity between the metallacyclic intermediate and an external double bond enhanced precoordination of a double bond by Ru, to favour migratory insertion into the C–Ru bond and final protodemetallation liberating a cyclised product. Quite remarkably, the same approach may also be used for two-fold unsymmetrical functionalisation. In such a case after initial intramolecular hydroarylation the Ru-catalyst re-enters a new catalytic cycle, inserts into the C–H bond at *ortho*′-position and final intermolecular amidation delivers a large panel of highly decorated scaffolds. MPS-bearing vinylic substrates are equally appealing precursors for this chemistry.^1053^ Indeed, one-pot double annulation of MPS-acrylamides occurred smoothly using a standard Ru(*p*-cymene)-catalyst in combination with internal alkynes, afforded pyrido-fused isoquinolone scaffolds (Scheme 169D). In course of this reaction both, symmetrical alkynes and two distinct unsymmetrical coupling partners may be employed. Besides, heteroaromatics substituted by sulfoximine motifs are equally efficient *via* a closely related protocol, delivering π-conjugated polycyclic amides in high yields.^1054^ Noteworthy, the MPS auxiliary is a traceless DG that can be readily removed after the C–H functionalisation (under basic conditions) or is directly cleaved during the annulation events.

In parallel, Ackermann designed a C–H oxygenation reaction of sulfoximines. As for previous examples, Ru(*p*-cymene) is an optimal catalyst and after fine optimization, an iodine(iii) and Ag(i)-free protocol was established to allow direct C–O coupling between sulfoximine benzamides and aromatic carboxylic acids (Scheme 169E).^1055^ This highly monoselective transformation well tolerates both electron-donating and electron-withdrawing substituents on the carboxylate, delivering the desired products in moderate to good yields (typically 50–70%). Notably, the functionalised sulfoximines may be readily converted into salicylic acids under acidic conditions.

### 16.4. Thioamide, thioketones, alkoxythiocarbonyls and thiocarbamates as DG

**Scheme 170:**
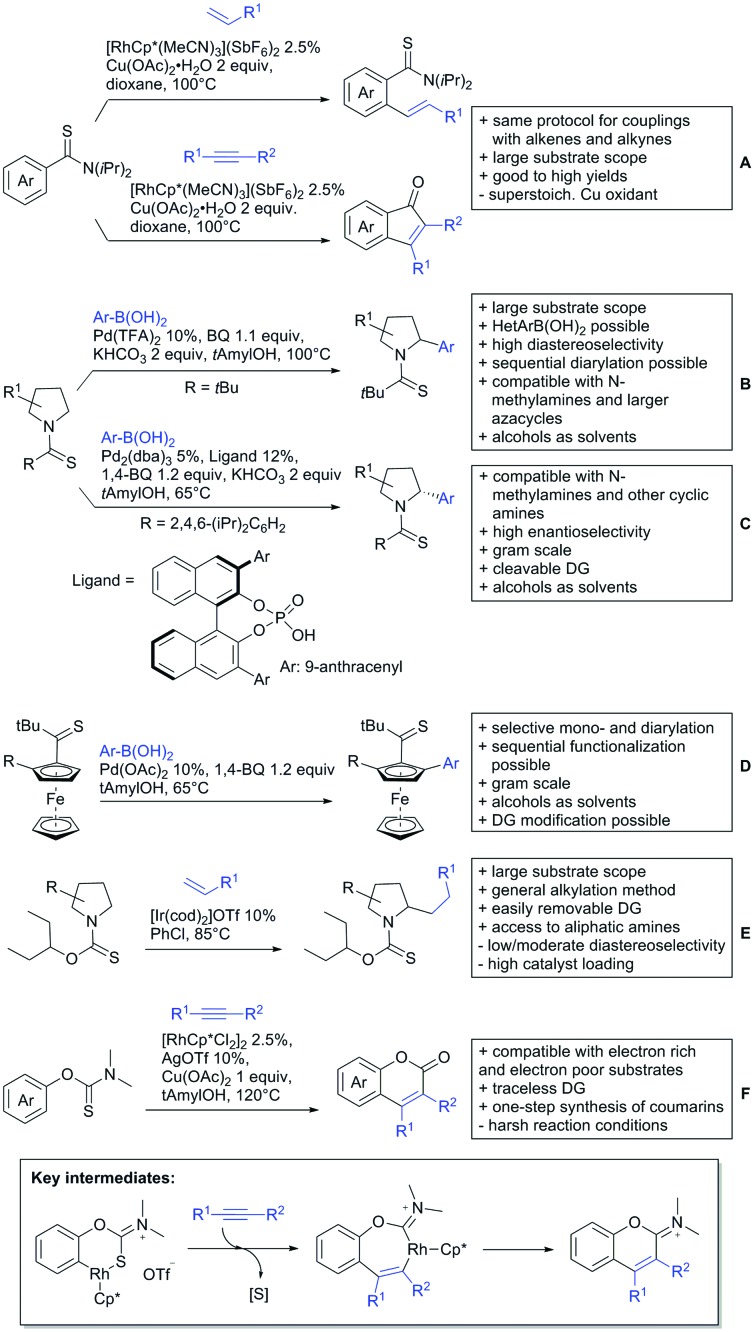
Thioamide, thioketones, alkoxythiocarbonyls and thiocarbamates as DG.

Another possibility of employing sulfur in a coordinating group relates to the use of thiocarbonyl motifs. Pioneering work in this challenging field was reported in 2015 by Satoh and Miura who disclosed Rh-catalysed direct coupling of benzothioamides with olefines and alkynes.^1056^ In this case *N*,*N*-diisopropylthioamide was used as DG and the expected functionalisation performed well under standard conditions. Importantly, oxidative Heck reaction with acrylates and styrenes delivers *ortho*-olefinated products while intramolecular cyclisation and desulfurization occur in presence of an alkyne partner, delivering indenone-derived products (Scheme 170A). Both couplings are high yielding and support well both electron-rich and electron-poor substrates. Few months later Yu and co-workers showcased that installation of a thiocarbonyl moiety on heterocycles such as pyrrolidine, piperidine, azapines or *N*-methyl amine is an efficient approach for α-arylation.^1057^ The corresponding thioamides undergo smoothly Pd-catalysed metallation and following coupling with boronic acids affords functionalised amine-derivatives (Scheme 170B). Remarkably, this protocol well tolerates a large panel of boronic acids, including sterically demanding *ortho*-substituted ones as well as heteroaromatic partners. High levels of diastereoselectivity are generally reached when substituted pyrrolidine substrates are used and complete diastereoselectivity is observed when one pot, sequential diarylation is performed. Importantly, this thiocarbonyl motif can be removed with methyl lithium as base and the generated amine is protected as the Boc-carbamate. Alternatively, the corresponding amide may also be accessed by oxidation with silver salts. In 2017 the same group reported an enantioselective version of this transformation.^1058^ Chiral induction (up to 98/2 e.r.) was accessed in presence of a phosphoric acid ligand (Scheme 170C). This arylation reaction is compatible with both cyclic and acyclic amines and as cleavage of the DG might be achieved (2-steps procedure including reduction of the thiocarbonyl in presence of NiCl_2_ and NaBH_4_, followed by BCl_3_-mediated debenzylation) it can be considered as an interesting strategy to access stereogenic amines.

Further potential of the thiocarbonyl moiety to enhance C–H activation was illustrated by You who disclosed thioketone-directed arylation of ferrocenes (Scheme 170D).^1059^ Under reaction conditions close to the one developed by Yu, palladation of thiocarbonylferrocene occurs straightforwardly, delivering either mono- or bi-arylated ferrocenes in generally high yields. The protocol is also applicable for sequential diarylation. The final product could be converted either into the corresponding ketones or a RANEY®-nickel mediated reduction delivered a benzylated congeners.

To further improve the traceless character of a thiocarbonyl based DG, Yu designed in 2017 an alkoxythiocarbonyl auxiliary.^1060^ This novel sulfur-based DG is particularly appealing in Ir-catalysed C–H activation, affording alkylated compounds in presence of non-activated olefin coupling partners (Scheme 170E). Thanks to a large scope of this coupling and convenient removal of the auxiliary (treatment with ac. solution of TFA followed by *N*-protection) an array of original aliphatic amines may be build-up efficiently.

An alternative C=S type directing group, thiocarbamate, was disclosed by Xia.^1061^ Remarkably, in this case the DG group is truly traceless; in presence of an internal alkyne coupling partner, the C–H activation step and subsequent C–C bond formation event are followed by annulation, delivering coumarins (Scheme 170F).

### 16.5. Phosphine oxides and sulfides as P-based DG

Organophosphorus molecules are key structural motifs in a large panel of pharmaceuticals, agrochemicals and organic materials. Straightforward access to such scaffolds hence presents an appealing synthetic challenge for organic chemists and therefore it is not surprising that diversification of phosphorus-containing molecules by means of C–H activation has been attracting a great deal of attention. The pioneering key contribution in this field were already elegantly summarized elsewhere.^17^ The use of auxiliary phosphorus-containing DGs, such as monophosphonic acid phosphonate, presented however a serious drawback related to a low modularity of these DGs. Accordingly, the improvements that were expected in this domain concerned: (1) design of transformations implying generation of the functionalised P-containing molecules where the auxiliary is voluntarily presented in a final product skeleton; (2) conception of innovative, readily transformable or traceless DGs.

**Scheme 171:**
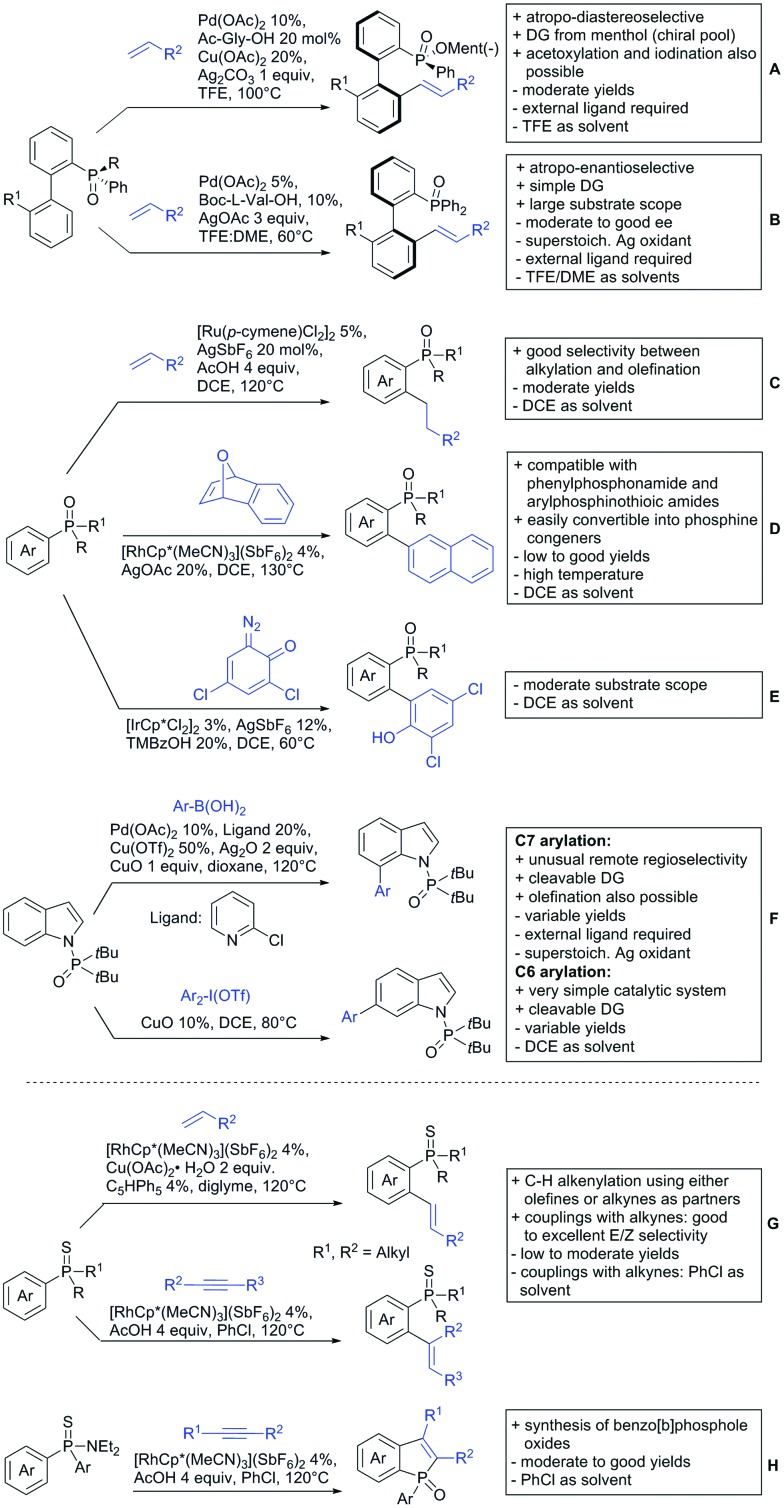
Phosphine oxide- and sulfide-directed transformations.

Amongst phosphorus-based DGs, phosphine oxides are particularly interesting motifs. Indeed biaryl phosphines are key ligands for both non-asymmetric and stereoselective transformations and thus synthesis of (chiral) biarylphosphine oxides is highly appealing and much work has been already done in this field.^1062^ This coordinating group exhibits a modular activation mode as coordination of the metal catalyst can be achieved either *via* oxygen or phosphorus atoms, depending on the (1) hard/soft properties of the catalyst used and (2) geometrical considerations and size of a metallacyclic intermediate formed. Accordingly, if functionalisation of biaryl substrates bearing the phosphine oxide-DG is targeted, Pd-based catalytic systems seem particularly suited as a precoordination of this soft metal by the soft P-atom is expected. In accordance with these predictions, a variety of Pd-catalysed functionalisations of phosphine oxides biaryls have been achieved. More recently, this approach was valorized by synthesis of axially chiral phosphine ligands. Initially, a diastereoselective approach has been devised using a chiral phosphine based DG, bearing a menthol substituent as chiral source (Scheme 171A). Rewardingly, direct functionalisation of the corresponding substrates occurred smoothly, delivered atropisomerically highly enriched products *via* C–C, C–O and C–I couplings.1063*a* Subsequently, a closely related acylation of biaryl substrates bearing now a P-chiral DG was disclosed.1063b,c This methodology was further complemented with an enantioselective transformation. Indeed, Yang *et al.* discovered that if a simple diphenylphosphine oxide DG is installed on biaryl substrates, Fujiwara–Moritani coupling catalysed by Pd(OAc)_2_ in presence of a chiral amino acid ligand, takes place efficiently in *ortho*′-position with an excellent enantioselectivity (Scheme 171B).^1064^ The extensive ligand screening revealed that structurally simple Boc-l-Val-OH ligand is optimal in terms of chiral induction, delivering the atropisomeric olefin/phosphineoxides in typically 90% ee. The reaction occurs under relatively mild reaction conditions (60 °C) and probably implies a dynamic transformation such as dynamic kinetic resolution and/or dynamic catalytic asymmetric transformation.

Aside from Pd-catalysed transformations, phosphine oxide DGs are also compatible with Ru, Rh and Ir-based catalysts. Likewise sulfoxide-DGs, as phosphine oxide coordinate these rather hard catalysts *via* the O-atom, a 5-membered metallacyclic intermediates is hence generated when this DG is installed directly on an aromatic ring. Following this analysis, Soulé reported recently direct alkylation of arylphosphine oxides in presence of a Ru-based catalyst (Scheme 171C).^1065^ This monoselective C–C coupling occurs with moderate to high yields, but the newly accessed scaffolds can be considered as original phosphoros ligand.

Straightforward *ortho*-metallation of arylphosphine oxides may also be performed in presence of a RhCp*-catalyst, as illustrated by Satoh and Miura (Scheme 171D).^1066^ The authors discovered that heterocyclic compounds are also suitable coupling partners, allowing direct arylation. This strategy is an interesting alternative to commonly used iodoarenes as coupling partners, delivering biaryl compounds in variable yields ranging from 30 to 91%. Metallacyclic Ir/phosphine oxide intermediates are also prone to undergo migratory insertion of metal carbenes (Scheme 171E).^1067^ This synthetic route delivers original biaryl scaffolds up to gram scale and their diversification *via* FG interconversion was illustrated.

Outstanding application of the phosphine oxide DG was reported in 2016 by Shi.^1068^ The authors installed this coordinating motif on the N-atom of indole substrates and observed unexpected regioselectivity during a Pd-catalysed coupling with boronic acids. Indeed, selective arylation at C7, over generally observed C2-functionalisation could be attained in synthetically useful yields when installing bulky phosphine oxide DGs, such as P(O)(*t*Bu)_2_ (Scheme 171F). Due to steric reasons, the O-atom is perfectly oriented to perform C–H activation at this specific C7 position. In addition, bulky *t*Bu-substituents on P-atom prevent N–P bond rotation further favouring the observed selectivity. Few months later the same research group succeeded in designing a complementary Cu-catalysed, C6-selective transformation. Drawing inspiration from Gaunt,^1069^ they reacted the same di-*t*Bu-phosphine oxide substituted indole with iodanes.^1070^ In line with the expectations, C7 *vs.* C6 regioselectivity shift occurred *via* transfer of the aromatic ring bound to Cu (resulting from oxidative addition of Cu into iodine) to C6 position *via* the Heck-type four-membered-ring transition state. Accordingly, using the same indol substrate with a judiciously selected DG the functionalisation could be triggered at unusual position, by simple changing the catalyst used and the nature of the arylating agent. Importantly, the DG can be removed by treatment with lithium aluminum hydride to give the unprotected indole derivatives.

In addition to phosphine oxide, phenylphosphine sulfides may also act as efficient DG in RhCp*-catalysed transformations. Such a P=S DG was used in 2014 by Satoh and Miura to provide new protocols for direct alkenylation occurring either *via* oxidative Heck reaction or redox-neutral coupling with internal alkynes (Scheme 171G).^1071^ Also thiophosphinamides exhibit a somehow related reactivity as illustrated Satoh and Miura who used this P-DG in Rh-catalysed coupling with heterobicyclic alkenes.^1066^ The same substrates may also undergo *ortho*-alkenylation and subsequent cyclisation delivering arylthiophosphinamides,^1072^ compounds of interest due to their intriguing electronic and optical properties (Scheme 171H).

**Scheme 172:**
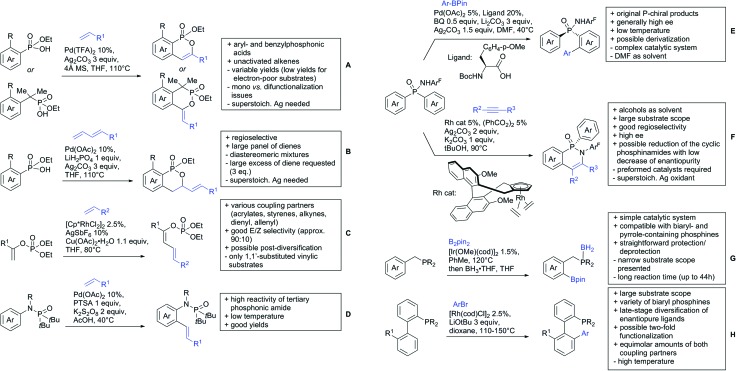
Other P-based DGs in C–H functionalisation.

Phosphonic acids constitute another sub-category of P-based DGs. The originality of this DG arises from the presence of an OH-free group on the phosphorus atom and accordingly precoordination of a metal by this motif is expected. Several interesting examples of C–H activation reactions directed by this moiety have been reported before 2015 and they were highlighted elsewhere.^17^ More recently, the group of Lee focused on synthesis of phosphaisocoumarins *via* C–H activation strategy directed by P-containing DG (Scheme 172A). The first successful protocol relates to a phosphoannulation reaction of aryl- and benzylphosphonic acids with unactivated alkenes *via* direct C–H coupling/C–O cyclisation step sequence.^1073^ This reaction is efficient for both, aryl-and benzylphosphonic acids. The olefination reaction delivers products in moderate to good efficiency. Few months later the same research group provided an alternative approach towards dihydrophosphaisocoumarins *via* C–H activation followed by C–O cyclizing bond formation (Scheme 172B).^1074^ In this case 1,3-dienes are used as coupling partners and the regioselectivity of this transformation may be arguably attributed to a formation of Pd π-intermediates. Unfortunately, the reaction does not allow diastereocontrol and the products are obtained as mixture of diastereomeric products albeit in high yields.

Targeting design of transformable/traceless P-containing DGs, several research groups focused on auxiliaries installed on an aromatic substrate *via* a heteroatom like O, or NH motif. Following this idea, enol phosphate DGs have been designed^1075^–^1078^ and employed them in direct functionalisation of aromatics. In 2015 Loh expanded this reactivity by developing a catalytic system able to functionalise vinyl C–H bonds (Scheme 172C).^1079^ Cationic RhCp* catalyst in combination with Cu(OAc)_2_ and pivalic acid allowed selective alkenylation of phenylvinyl phosphates. The reaction well tolerates an array of alkene substrates and the diene products are generally obtained in high yields (typically 70–90%) and good *E*/*Z* selectivity (approx. 90 : 10). Besides, when using vinyl ketones as coupling partners selective hydroalkenylation occurs and also unsymmetrical alkynes may be used in this C–C coupling. Finally, in accordance with the general target of devising traceless DGs, efficient removal of this motif using DIBAL-H or TBAF based protocols. In addition, the O-tethered DG may also be considered as a reactive site to perform Ni-catalysed C–C coupling delivering an arylated product.

Besides O-tethered P-containing DGs, use of N-linked directing groups also attracted scientific curiosity. In 2013 Lee developed phosphoramidite-directed arylation.^1076^ More recently, Oestrich *et al.* designed phosphinic amides as novel handle for Fujiwara–Moritani reaction (Scheme 172D).^1080^ Both, NH and NMe anilline derived phosphonic amides performed well. Besides, the oxidative olefination with this DG occurred smoothly at temperature as low as 40 °C, *vs.* 90 °C required for the activation of substrates bearing an NH-anilide. The transformation well tolerates few electron-donating substituents on the aromatic ring of the substrate as well as few activated olefins.

Alternative, as P-chiral molecules are intriguing motifs, in particular for asymmetric synthesis, design of stereoselective C–H activation reactions directed by P-motifs and delivering chiral molecules is highly appealing. Following this idea Han surmised that if symmetrical phosphinamides are used, a direct activation of one of the aromatic substrates would result in a desymmetrisation of the product, hence delivering P-stereogenic molecules (Scheme 172E).^1081^ As expected, when using a Pd-catalyst in combination with mono protected amino acid ligands, desymmetrizative arylation of phosphinamides occurred, delivering the corresponding P-chiral molecules in moderate to good yields and good enantiomeric excess of approx. 90%. Notably, the post-diversifications were performed with little or no loss of chiral information.

In 2017 Cramer reported a complementary catalytic system based on the use of chiral RhCp-derived catalyst bearing an atropisomeric Cp-type ligand (Scheme 172F).^1082^ During this directed alkynylation intramolecular annulation takes place delivering P-chiral cyclic phosphinamides. The reaction shows large scope, good tolerance towards substrates bearing a variety of electron-withdrawing and electron-donating substrates and very high regioselectivity with respect to unsymmetrical alkynes. The yields are moderate to good end ees of roughly 90% were obtained. Subsequently, this chemistry was employed to functionalise racemic dissymmetric phosphinic amides. The same catalytic system was now employed to perform direct stereoselective C–C coupling *via* kinetic resolution.^1083^

Finally, the panel of P-based DGs is complemented by using simple phosphines as coordinating motifs. This approach seems highly challenging and counterintuitive as phosphines are excellent ligands and their use in a stoichiometric amount as DG could lead to “catalyst poisoning”. In addition, oxidative conditions frequently applied in C–H activation might lead to the oxidation of this DG. However, in 2014 Clark reported Ir-catalysed phosphine-directed *ortho*-borylation of aromatics (Scheme 172G).^1084^ Like in a case of thioether (see Section 16.1), the phosphine is installed at the benzylic position to allow generation of 5-membered metallacyclic intermediates. This reactivity could also be extended to diaryl and heteroaryl compounds where a PR_2_-motif directs functionalisation at *ortho*′-position. Noteworthy, *in situ* protection of the phosphine as the borane complex takes place, enabling convenient isolation of otherwise air-sensitive boronate esters, and deprotection in presence of Et_2_NH can be conducted when requested.

Following this seminal work, in 2017 Shi discovered that direct C–H functionalisation may be applied as an efficient handle to diversify privileged biaryl-monophosphine ligands. Indeed, a PR_2_-motif is able to transiently coordinate a Rh(i)-catalyst and *via* formation of a metallacyclic intermediate direct the metallation step at the *ortho*′-position and further promoting C–C coupling with arylbromides (Scheme 172H).^1085^ Use of an equimolar ratio between a phosphine substrate and Ar–Br partner permits selective mono functionalisation while an increased amount of the coupling partner favours formation of diarylated congeners. Remarkably, this protocol is compatible with an array of commercially available monophosphine ligands such as CyJohnPhos, DavePhos and cataCXium auxiliaries like PinCy or POMeCy. Noteworthy, with enantiopure substrates such as binaphthylphosphine H-MOP or Tang's ligand, the arylation occurs with no loss of the chiral information. Accordingly, this reaction is a unique tool for the late stage diversification of ligands.

Noteworthy, the phosphine motif can also be applied in Cu-mediated alkynylation reactions, delivering, after a cyclisation step, an array of phosphindolium salts.^1086^ Unfortunately, although this protocol gives straightforwardly access to important heterocyclic motifs, need for 2 equivalents of the metal salt limits its synthetic attractiveness.

### 16.6. Si-Based DGs

One of the major limitations of directed C–H activation relates to the need for preinstalled coordinating motifs, prompt to precoordinate a metal catalyst and thus enhance regioselective metallation. Although over the last years particular attention has been focused on devising C–H activation reactions benefitting from the functionalities inherently present on a desired product that may play a role of a DG, many catalytic systems still call for the use of non-modular and/or difficult to remove DGs that become static entities. In such a case synthetic value of the C–H activation methodology is clearly reduced. To obviate this serious limitation, the concept of “traceless” DGs emerged recently, enhancing conception of innovative coordinating motifs that are easy to install but also readily cleaved or transformed under mild reaction conditions after the C–H activation step.^1087^ In this context use of silicone moieties is particularly appealing due to: (1) relative stability and easy access to silicone derivatives; (2) availability of cheap silicone precursors; (3) straightforward Si–X bond cleavage allowing removal of these tethers under mild and often chemoselective protocols, such as the Tamao–Fleming oxidation^1088^ or the Hiyama coupling.^1089^ Besides, in intramolecular transformations, if a SiR^1^R^2^H motif is used, this moiety can play a dual role of both DG and reagent.

Following this general concept several research groups entered the field of Si-based DGs in C–H functionalisation.^735^,^1090^,^1091^ Already more than one decade ago several research groups questioned the potential of “SiR_2_H” to play a double role of a DG to drive the C–H activation event at one specific position and to provide, in an intramolecular fashion, a silicone-based coupling partner. The potential of such reactions holds also a major synthetic value as the newly formed C–Si bonds might be further interconverted into several functionalised motifs.

**Scheme 173:**
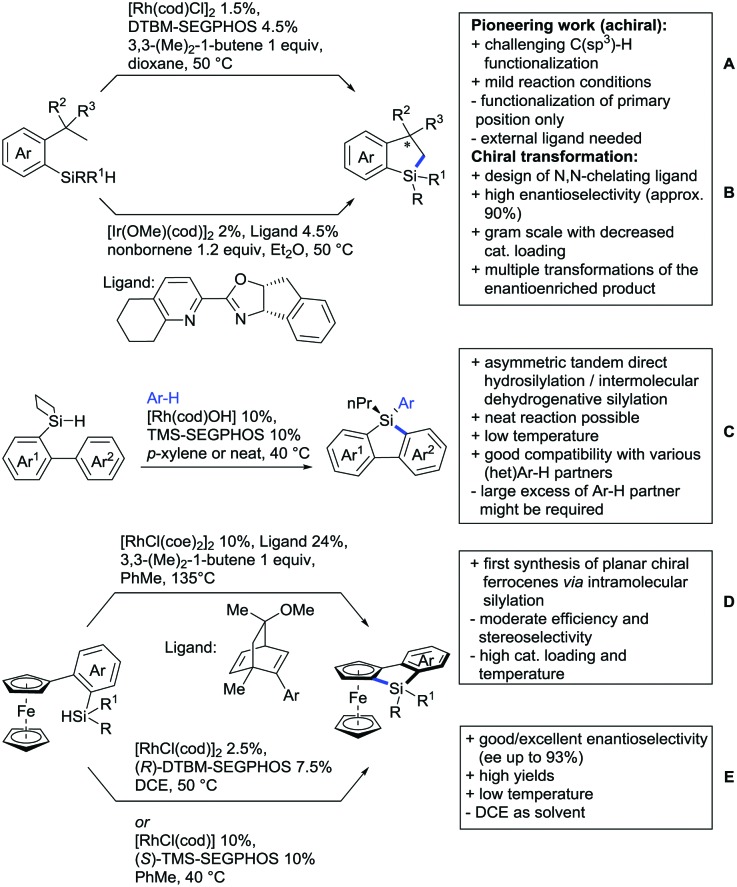
Silanes as DGs (intramolecular silylation).

Following this concept, before 2015 several catalytic systems allowing direct silylation of aromatics and heteroaromatics were reported whereas direct functionalisation of aliphatic substrates remained a less developed field.^1092^–^1094^ Besides, design of stereoselective transformations was a challenging goal. Following several pioneering key contributions, Murai and Takai reported in 2015 an improved Rh-based catalytic system for intramolecular silylation of unactivated C(sp^3^)–H bonds (Scheme 173A).^1095^ The authors discovered that replacement of the previously employed dppp diphosphine ligand by an axially chiral, structurally well defined SEGPHOS auxiliary in combination with an hydrogen acceptor (such as an olefin) drastically accelerates the desired reaction, enhancing smooth conversion of the starting materials into products at a temperature as low as 50 °C *vs.* 180 °C previously requested. Importantly, use of a chiral ligand and milder reaction conditions permitted a stereoselective outcome of this transformation, however only moderate chiral induction was observed (ee up to 40% was reached using the BINAP ligand). Highly enantioselective silylation of aliphatic substrates was achieved two years later, in 2017. Hartwig *et al.* discovered that switching from Rh–diphosphine catalyst towards an Ir complex bearing a chiral *N*,*N*-donor ligand, such as indane-fused oxazoline, clearly improved this challenging C–H activation reaction that now delivers the silylated compounds in high yields and good enantioselectivities (ee of approx. 90%) (Scheme 173B).^1096^ Importantly, the newly generated stereoenriched dihydrobenzosiloles (accessible also on gram scale using a largely decreased catalyst loading) are a unique platform to access an array of chiral molecules *via* chemoselective functionalisation of each C–Si bond.

In addition to stereoselective silylation of unactivated C(sp^3^)–H bonds, several research groups applied the same concept to synthesize chiral molecules *via* functionalisation of C(sp^2^)–H bonds of prochiral substrates. The pioneering key contribution by Takai and Kuninubu provided access to original enantiomerically pure spirosilabifluorenes *via* two-fold intramolecular silylation.^1097^ Expanding this methodology, He reported in 2017 a tandem transformation involving C–H desymmetrizating silylation, followed by intermolecular dehydrogenative silylation (Scheme 173C). Such a transformation, catalysed by a Rh–SEGPHOS complex, allows the interconversion of silacyclobutanes into chiral tetraorganosilicons in moderate to good yields and ees between 65 and 93%.^1098^

Intramolecular stereoselective C(sp^2^)–H silylation was also astutely used to access planar chiral benzosiloloferrocenes. The groups of Shibata,^1099^ Murai/Takai^1100^ and He^1101^ reported simultaneously but independently intramolecular silylation of metallocenes. Although when employing the first protocol (Scheme 173D) based on a Rh-catalyst and a chiral diene ligand only moderate chiral induction was observed (up to 86% ee),^1099^ clearly more appealing ees were obtained in presence of Rh–SEGPHOS catalyst (Scheme 173E).^1100^,^1101^ Notably, using the second protocol either reaction temperature could be decreased to 40 °C or significantly lower catalyst loading (2.5 equiv.) was employed.

**Scheme 174:**
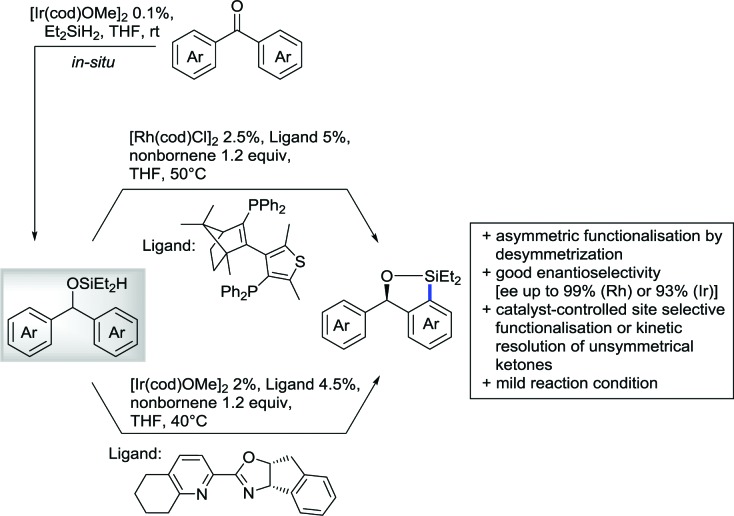
*In situ* formed silyl ethers as DGs (C(sp^2^)–H intramolecular silylation).

An alternative approach to push even further the synthetic value of the above described methodology consists of installing the silicone moiety as a temporary tether such as a silyl ether group. This elegant concept, developed in 2010 by Hartwig, implies *in situ* generation of silyl ethers from ketones or aldehydes *via* Ir-catalysed hydrosilylation, followed by direct intramolecular silylation catalysed by a second Ir-catalyst.^1102^ Importantly, thus accessed scaffolds may now be chemoselectively converted into an array of functionalised molecules *via* selective O–Si or C–Si cleavage reactions using standard protocols such as Tamao–Fleming oxidation or Hiyama coupling. A further key step forward in this field consisted of designing a related stereoselective transformation. Following this target, Hartwig described in 2015 a (hydrido)silyl-directed desymmetrisation reaction of benzophenone derived substrates.^1103^,^1104^ The authors discovered that while conserving the Ir-catalyst for the first step, *i.e.* ketone hydrosilylation, high reactivity and excellent chiral induction for the C–Si bond formation are reached in presence of Rh(i)-catalyst coordinated diphosphine ligand, in particular catASium or Walphos-derivatives and nonbornene as a hydrogen acceptor. The reaction takes place under mild reaction conditions (50 °C). Accordingly, a large panel of highly enantioenriched diarylmethanols is accessed. Noteworthy, when dissymmetric enantiomerically pure diaryketones are used as substrates high catalyst-controlled site-selectivity is observed. Very recently, the same research group designed an alternative catalytic system based on Ir(i) bearing an *N*,*N*-bidentate quinoline-oxazoline ligand.^1105^ The reaction is equally efficient and enantioselective as the Rh-based protocol but even lower reaction temperature, *i.e.* 35 °C, could be used. In addition, kinetic resolution took place when submitting racemic silyl ethers to the standard reaction protocol, affording the silylated products with high selectivity (Scheme 174).

**Scheme 175:**
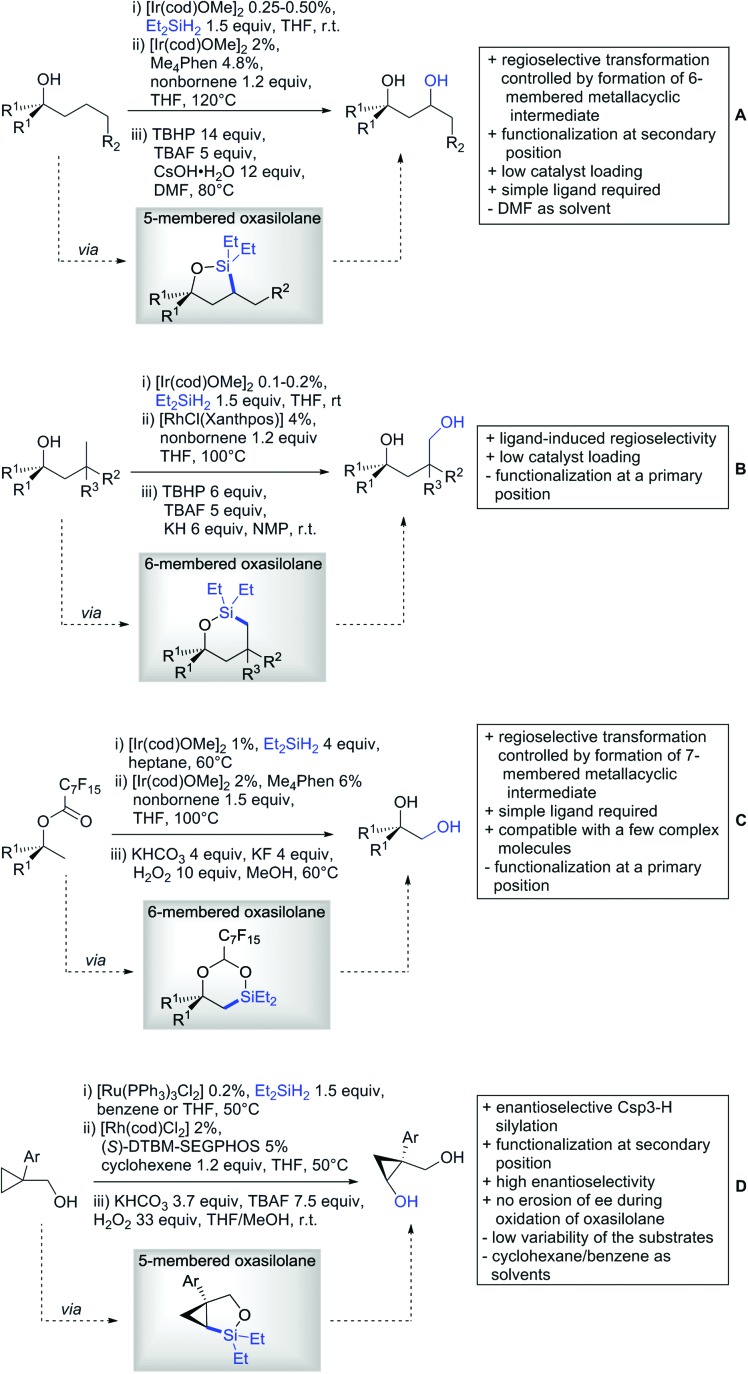
*In situ* formed silyl ethers as DGs (C(sp^3^)–H intramolecular silylation).

In addition to direct C(sp^2^)–H activation under mild reaction conditions, the concept of intramolecular silylation using a silyl ether moiety could be expanded to the functionalisation of simple aliphatic substrates. Such a transformation holds great promise for synthetic chemists as, considering high modularity of both C–Si and O–Si bonds, new synthetic routes towards diols could hence be envisioned. Following this inspiration Hartwig described in his pioneering work in 2014 hydroxy-directed silylation of secondary C(sp^3^)–H bonds (Scheme 175A).^1106^ When a (hydrido)silyl ether (obtained *via* dehydrogenative coupling between a ketone and diethylsilane) is reacted with the Ir-catalyst supported by phenanthroline type ligand, formation of a 6-membered metallacyclic intermediate operates furnishing 5-membered oxasilolane. Finally, smooth oxidation of the newly generated oxasilolanes using TBHP, in combination with CsOH monohydrate and *tert*-butyl ammonium delivers the expected 1,3 diols. Accordingly, the overall transformation allows conversion of aliphatic ketones into 1,3-diols, fully validating the initial hypothesis of the project. Very recently, design of protocols devoted to a synthesis of 1,2 and 1,4-diols spread the outlook of this methodology further. The regioselectivity switch towards more distal, δ-position was possible by means of catalyst design (Scheme 175B).^1107^ Hartwig discovered, in accordance with his previous work, that while using a Rh-catalyst with previously employed phenanthroline or common diphosphine ligands, such as BINAP or SEGPHOS, silylation occurs selectivity at 3-position. However, quite unexpectedly, addition of large bite angle Xanthpos ligand drastically altered the outcome of this C–H activation, directing the metallation event at 4-position and thus providing 6-membered oxasilolanes in good yields (around 70%). Accordingly, a 3-step procedure for direct conversion of aliphatic alcohols into 1,4-diols was devised.

In contrast, 1,2-selectivity for this direct silylation reaction was possible thanks to the substrate designed (Scheme 175C).^1108^ Accordingly, an easy to install and readily cleavable linker needs to be placed between an oxygen atom of a substrate and the silyl-moiety. Detailed investigations revealed that perfluoroacetal is perfectly suited to deliver the silyl DG in close proximity to the targeted δ-position. Accordingly, a reaction sequence including hydrosilylation of a perfluoroalkyl ester, followed by silylation of a C(sp^3^)–H bond and final Tamao–Fleming oxidation at the C–Si bond provides 1,2-diols. The protocol requires use of the Ir-catalysts in two initial steps and harsher reaction conditions are necessary to favour this short-distance functionalisation.

An additional challenge to be addressed in this field concerns stereoselectivity issues and development of an enantioselective directed silylation. The first step towards this goal was achieved in 2016 when the same group disclosed enantioselective synthesis of cyclopropane derivatives (Scheme 175D).^1109^ The authors hypothesized that the hydrosilyl ether of 1-arylcyclopropylmethanol may be an effective substrate under Rh(i)-based protocol. Despite significant steric hindrance, the expected C–Si coupling occurs at the more activated (presence of a phenyl substituent), tertiary carbon. Interestingly, the previously employed SEGPHOS type ligand outcompeted other axially chiral diphosphines such as BINAP, MeOBIPHEP and GARPHOS derivatives, delivering the expected cyclised products with high optical purity of approx. 90% yet in moderate to good yields. The reaction well tolerates an array of aromatic substituents on the cyclopropane substrate. Besides, oxidation of the oxasilolanes occurred with no erosion of the enantiopurity.

**Scheme 176:**
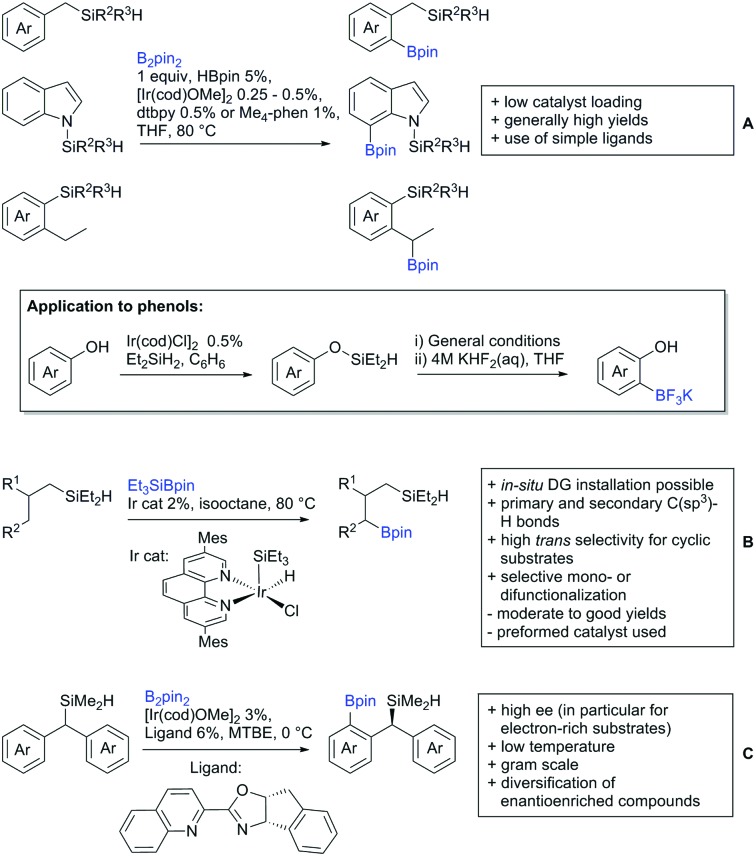
Silane-directed borylation reactions.

In his seminal work in 2008 Hartwig reported for the first time the Ir-catalysed *ortho* borylation of aryldimethylsilanes (Scheme 176A).^1110^ In this case, the Si moiety acts as a traditional DG, rather than a silylating agent, as in the previous cases in this section (*i.e.* the Si only coordinates to the metal, rather than being involved in a Si–C bond). The desired C–H functionalisation required Ir(i)–dtbpy complex and B_2_pin_2_ supplemented with a catalytic amount of HBpin, to provide the expected *ortho*-functionalised compounds. This methodology could be further expanded to silyl phenol-ethers. A sequential transformation thus permits direct conversion of phenols into 2-phenyl trifluoroborate salts (Scheme 176A, box). Besides, the structurally related 5-membered metallacyclic intermediates may also be generated when C–H activation occurs at the benzylic position of 2-alkylarylsilanes.^1111^

In addition, following the same concept, original 7-selective functionalisation of indoles was performed (Scheme 176A). In this example the silyl DG was installed *in situ* on the N-atom and drives the metallation event at C7, suppressing the inherent C2 reactivity of such substrates.^1112^ Accordingly, simple indoles were readily converted into 7-Bpin substituted heterocycles using [Ir(cod)Cl]_2_ catalyst in combination with dtbpy ligand.

A further major challenge in this field was taken up in 2016, when Hartwig devised a slightly modified catalytic system, sufficiently active to promote directed functionalisation of simple aliphatic substrates (Scheme 176B).^1113^ The strategy proposed by the authors consisted in (1) straightforward installation on the Si-based DG on an olefin *via* alkene hydrosilylation generating highly stable compounds; (2) hydrosilyl-directed Ir-catalysed direct borylation; (3) diversification of the accessed molecules *via* chemoselective cleavage and conversion of the C–Si and C–B bonds. The optimization study revealed that efficient and reproducible C–B coupling occurs when using an isolated Ir-complex bearing a mesityl-substituted phenanthroline ligand in combination with highly reactive Et_3_SiBpin boron source, in isooctane and at 80 °C. *Via* this protocol, selective mono-borylation occurs selectively at a secondary γ-position generating *trans*-adducts with high stereoselectivity. Noteworthy, in case of cyclic substrates, geometrical considerations (size of the metallacyclic intermediate formed) override steric issues, as the functionalisation of a secondary carbon at γ-position is favoured over borylation of a primary δ-position.

In 2017, the first example of an enantioselective silyl-directed C–B coupling was disclosed. Drawing inspiration from their previous work on desymmetrizative silylation of diarylketones, Hartwig and Shi developed directed borylation of diarylmethylsilanes (Scheme 176C).^1114^ As Ir-catalysed borylation are generally milder, the intensive efforts were focused on designing a chiral ligand able to promote this challenging transformation at room temperature. Although enantiopure ligands such as BINAP, various bisoxazolines and bipyridine derivatives failed, the desired coupling took place in presence of a quinolyl oxazoline auxiliary. Further fine-tuning of the ligand structure led to the design of the optimal chiral inductor, allowing the desired C–H activation to occur smoothly even at 0 °C and with high chiral induction. As previously mentioned, this catalytic system is robust and the reaction performed at 5 mmol scale delivered the product in comparable yield and ee, albeit significantly decreased catalyst loading (0.75% *vs.* 3% used for small scale reactions). Besides, the enantioenriched borylated products could be further diversified *via* selective FG interconversions.

A complementary application of silyl moieties in the context of C–H activation consists in using these motifs as temporary tethers allowing installation of proper strongly coordinating DGs, like heteroaromatics, or a remote nitrile function. This type of DG is presented in Section 10.2. Alternatively, Si-atom may also be used as linker connecting two moieties of a molecule for an intramolecular coupling initiated by oxidative addition of Pd into a C–X bond.^1115^,^1116^ Finally, inspired from the recent regain of interest in using weakly coordinating DGs, concerns use of silanols and effective precoordination of a metal *via* hydroxy motif.

**Scheme 177:**
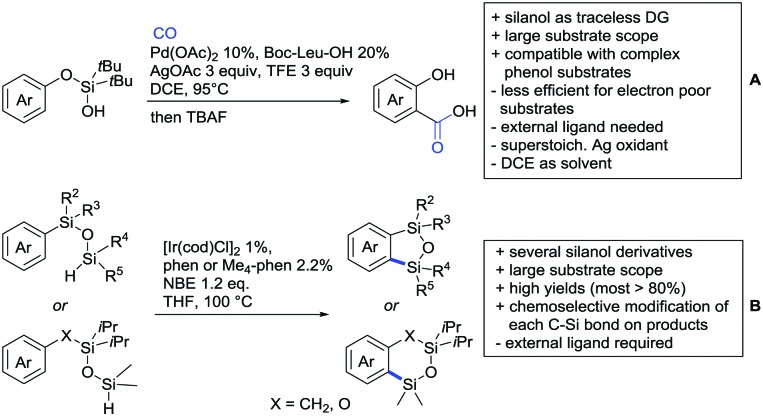
Silanols and siloxanes as DGs.

Silanols have established themselves as appealing weakly coordinating DGs at the beginning of this decade,^1117^ mainly based on key contributions by Gevorgyan^1118^,^1119^ and Ge.^1120^ Accordingly, an OH group directly installed on an aromatic ring allowed efficient and selective Pd-catalysed *ortho*-alkenylation^1118^ and oxygenation.^1119^ More recently, silanol-directed C–H carboxylation has been used to prepare salicylic acid derivatives (Scheme 177A).^1121^ Indeed, this new methodology implying: (i) conversion of an phenol into silanol, (ii) direct C–C bond formation using CO as a coupling partner and (iii) final deprotection of the OH in presence of TBAF delivers an array of salicylic acids in high yields, almost regardless the substitution pattern of the starting material (an array of FGs is well tolerated).

The potential of silanols has been further expanded by Cui and Xu who astutely tethered a second silicone unit on this DG (a siloxane moiety), thus targeting intramolecular C–H silylation (Scheme 177B).^1122^ Rewardingly, using an Ir-based protocol in combination with phenanthroline ligand they established a general protocol for direct C–Si bond formation. Notably, the reaction is compatible not only with arylsilanols, but also benzylsilanols and aryloxysilanols are efficient substrates. The design of the substrates allows installation of different substituents on the distinct silicone atoms hence chemoselective cleavage of C–Si and C–O bond occurs, ensuring remarkable post-modularity of the unsymmetrical cyclic siloxanes obtained.

### 16.7. OH- and OR-Based DGs

**Scheme 178:**
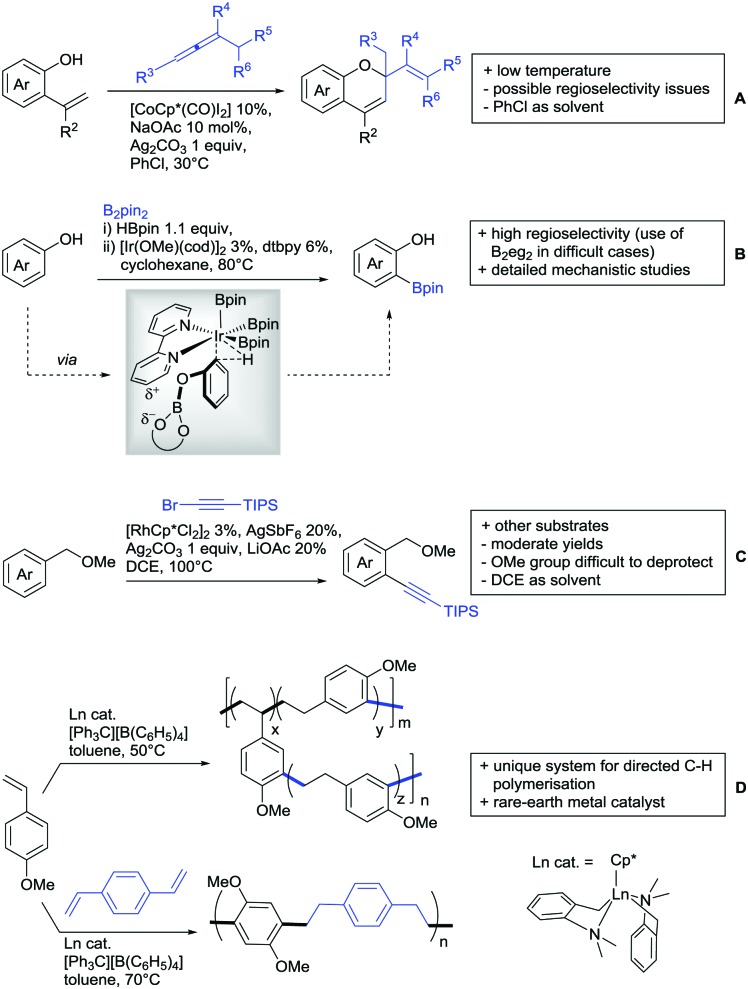
Hydroxy- and methoxy-directed transformations.

Although initially it was commonly recognized that metal-catalysed C–H functionalisation reactions require strongly coordinating DGs to enhance formation of stable metallacyclic intermediates, key contributions by Yu^33^,^248^ and others^1123^ clearly showed the potential of weakly coordinating DGs. This discovery encouraged the scientific community to explore the potential of many different “naturally occurring” DGs, amongst which the hydroxy group has attracted particular attention.^1124^ Indeed, between 2010 and 2015 many innovative direct C–H activation reactions have been reported, relating mainly to the functionalisation of phenols, benzylic alcohols or biarylphenols and they are already elegantly summarized elsewhere.^17^ In 2016 Cheng *et al.* illustrated that OH-directed C–H activation may also be achieved when using CoCp*-catalyst (Scheme 178A).^1125^ The authors astutely used the hydroxy-motif to enhance direct metallation of a vinylic C–H bond, located *ortho* to the DG and subsequent reaction with an allene, acting as a one-carbon coupling partner allows [5+1] annulation to occur delivering 2*H*-chromenes. The reaction occurs under very mild reaction conditions, delivering the targeted heterocyclic products in moderate to good yields. High regioselectivity is attaint when using a simple, mono-substituted allene, however mixtures of products were generally formed when structurally more complex coupling partners were employed.

Alternatively, the OH motif was also used as thether to install temporarily a second, strongly coordinating DG. In this context many examples of O-Py and O-heteroaromatic moieties have been disclosed and these examples are already shown in Section 15.3. Recently, an interesting example of *in situ* conversion of the OH moiety into a potent DG was disclosed by Maleczka, Singleton and Smith (Scheme 178B).^1126^ These research groups reported an *ortho*-borylation of phenols, a reaction elegantly complementing other C–H borylation protocols, for which regioselectivity is usually imposed by steric factors. The unusual selectivity of this reactions results from *in situ* borylation of the hydroxy group and subsequent electrostatic interactions between the OBpin motif and the bipyridine ligand coordinating the Ir-complex driving the metal catalyst at the *ortho* position. While excellent *ortho*-selectivity was observed for the functionalisation of *para*-substituted phenols (*para*-substituent larger than CN or F) with B_2_pin_2_, use of less sterically demanding B_2_eg_2_ was required to induce total regioselectivity for arenes not biased by *para*-substitution.

Although the quite widespread use of the hydroxy motif as DG, ethers are only rarely employed to precoordinate a catalyst and drive the metallation event.^1127^,^1128^ Recently, Echavarren addressed this challenge by designing a RhCp*-based catalytic system promoting highly efficient *ortho*-alkynylation of benzyl ethers (Scheme 178C).^1129^ When TIPS-protected bromoacetylene is used as a coupling partner, a panel of benzylethers is available, as the reaction well tolerates several substituents on the aromatic ring (mainly halogens). However, for *para*-substituted substrates mono- *vs.* difunctionalisation issues arise. Unfortunately, the reaction is efficient only for methyl-benzylethers. It is important to highlight that other weakly coordinating DGs, such as sulfoxides, thioethers, sulfones, dialkylamines, sulfones, etc also perform well under a very similar protocol.

Another interesting example for the use of a methoxy-group as coordinating motif was disclosed by Hou. The authors devised a catalytic system based on half-sandwich rare earth catalysts to enhance unusual simultaneous chain-growth polymerization (by continuous C=C insertion) and step-growth polymerization (C–H addition of anisole units to vinyl groups).^1130^ The same catalyst was further exploited in a copolymerization process between 1,4-dimethoxybenzene and 1,4-divinylbenzene or norbornadiene, delivering original polymeric materials (Scheme 178D).^1131^

### 16.8. Amine-based DGs

Amines occupy a particular position in the field of C–H activation. This motif was amongst the first ones to be explored as a DG while studying stoichiometric cyclometallation reactions.^1132^ Not surprisingly, dialkyl amines have thus been used with some success in catalytic reactions.^17^ In recent years, dialkylamine DGs were astutely used to enhance direct functionalisation of the ferrocene scaffold. In presence of Pd-based catalysts in combination with a chiral ligand, powerful protocols for stereoselective arylation, alkynylation/dehydrogenative annulation and olefination have been designed. These works were already elegantly summarized elsewhere^1133^–^1135^ and will not be detailed in this review.

Aside from ferrocene chemistry, the amine moiety is also a handy tool to promote regioselective, direct diversification of biaryl scaffolds. Indeed, much attention has been focused on 2′-functionalisation of biaryl-2-amines as, likewise biarylsulfoxide substrates for example, coordination of a metal catalyst by the N-atom generates a 6-membered metallacyclic intermediate. This general approach was used for both intramolecular couplings (synthesis of carbazoles for instance)^1136^–^1139^ and intermolecular transformations, like domino olefination/cyclisation,^1140^,^1141^ direct carbonylation delivering heterocyclic compounds^1142^,^1143^ or C–H arylation.^1144^,^1145^

**Scheme 179:**
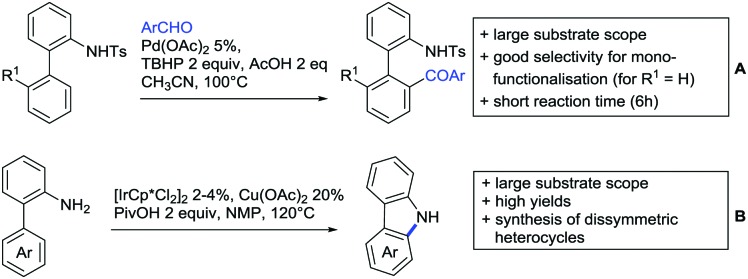
Aromatic amines as DGs.

In 2015 Wang and Ji spread the portfolio of such biaryl functionalisations by developing 2′-selective acylation (Scheme 179).^1146^ The optimized protocol allows selective mono-acylation of the Ts-protected biaryl-2-amines, under Pd-based catalysis and in presence of aldehydes as coupling partners and peroxide oxidants (TBHP). The reaction well tolerates several different FGs, both electron-withdrawing and electron-donating, on the biaryl substrates and a large panel of aldehydes is compatible with this protocol. Besides, in presence of a larger excess of the coupling partner and replacing the AcOH additive by PivOH, difunctionalisation is favoured.

Amine DGs are also a unique handle for one-step carbazole synthesis.^1136^–^1139^,^1147^–^1149^ Biaryls bearing an NH_2_-motif in the *ortho* position are indeed potent substrates for Ir-catalysed C–H activation followed by intramolecular C–N bond formation, delivering the heterocyclic product (Scheme 179B).^1150^ This generally high-yielding protocol is compatible with an array of either electron-rich or electron-poor substrates, straightforwardly furnishing dissymmetric carbazoles.

**Scheme 180:**
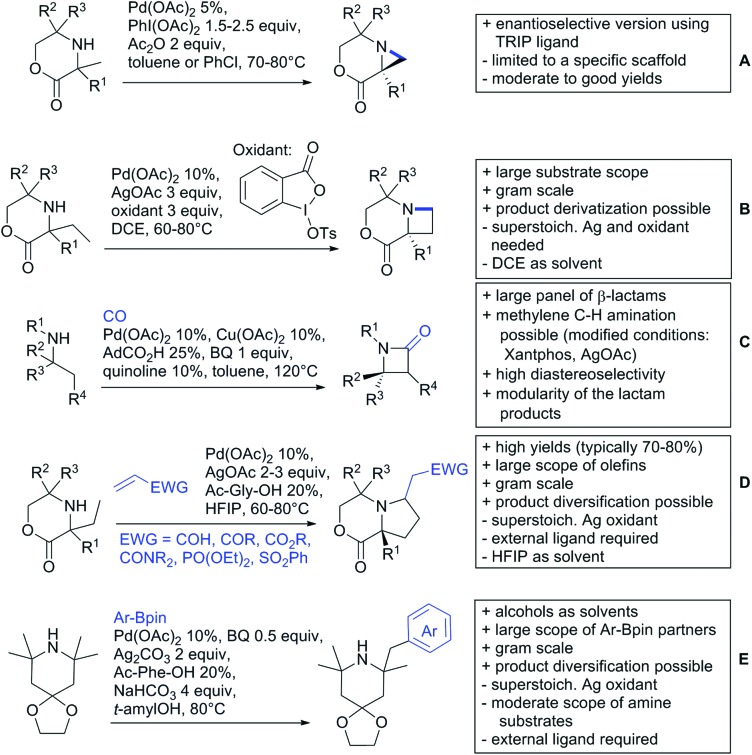
Functionalisation of C-sp^3^ centres directed by aliphatic amines.

A conceptually different strategy towards amine-directed C–H activation has been developed over the last three years by Gaunt and coworkers. This group recognized a lack of precedents in direct functionalisation of aliphatic substrates, and in particular they were interested in devising transformations showing complementary regioselectivity with respect to commonly observed γ-C–H direct couplings. Following this target, they discovered that aliphatic amines may undergo metallation at unusual β-position (*via* formation of an unprecedented 4-membered metallacycle intermediate) and in presence of an oxidant, such as PIDA, aziridine-type products are generated (Scheme 180A).^1151^ This unusual reaction is possible thanks to the steric hindrance of the amine that prevents formation of inactive bis(amine)–Pd complexes but consequently this transformation is mainly limited to structurally complex secondary cyclic amines, typically aminolactone congeners. Importantly, aziridine ring opening can be performed efficiently on the isolated products, delivering a panel of complex amine congeners, including quaternary amino acids. Moreover replacement of the hypervalent iodine oxidant with carbon monoxide results in a clean carbonylation reaction delivering β-lactams, albeit higher reaction temperatures are necessary to obtain synthetically useful yields (120 °C *vs.* 70–80 °C required for the aziridation process). Following this pioneering work, in 2017 the same research group reported a stereoselective version of this transformation. High chiral induction was achieved using BINOL-derived phosphoric acid ligands.^1152^ Another major step forward in this field was achieved by modifying the oxidation system.^1153^ Indeed, replacement of the previously used PIDA-type hypervalent iodine by benziodoxole tosylate in combination with AgOAc alters regioselectivity of the transformation, yet favouring azetidine formation (Scheme 180B). This regioselectivity switch is attributed to the influence of the OTs group. Formation of a 4-membered product is believed to occur *via* a two-step process including initial γ-tosylation followed by internal displacement of OTs by the amine moiety. The broad range of original azetidine compounds could hence be accessed and derivatization of these scaffolds delivers nitrogen-containing original compounds such as diols, amides, hydroxyl acids and hemiaminals.

Following this seminal work, Gaunt expanded also the field of amine-directed C(sp^3^)–H carbonylation reactions (Scheme 180C). Firstly, a general catalytic system promoting highly selective β-functionalisation of primary carbons (even in presence of protons at γ-position) was devised thanks to the addition of hindered carboxylic acid derivatives in combination with a quinone oxidant.^1154^ This modified protocol shows exceptional tolerance towards an array of secondary aliphatic amines so that a large panel of 4-membered amides was formed in good to excellent yields, including few biologically active scaffolds. Few months later it was discovered that addition of an external diphosphine ligand and replacement of the Cu-based oxidant by Ag salts generates a much more reactive catalytic system, prompt now to activate methylene β-C–H bonds of aliphatic amines.^1155^ The reaction is highly regioselective and compatible with an ample panel of substrates, bearing not only electron-donating and electron-withdrawing substituents, but also structurally complex motifs such as heterocycles. The reaction proceeds smoothly even when sterically congested α-tertiary amines are selected as starting materials.^1156^ Accordingly, a large panel of β-lactams may now be accessed from simple, non-protected and non-prefunctionalised aliphatic amines. In addition to the carbonylation reactions, Gaunt and coworkers described in 2017 alkenylation reactions (Scheme 180D). This formal dehydrogenative C–C coupling is assisted by an amino acid ligand (under this protocol the metallation step is reversible yet promoting and facilitating the targeted reactivity) and hence the C–H coupling followed by the annulation step leads to formation of complex heterocyclic structures.^1157^ Also a closely related arylation using arylboronic esters as coupling partners takes place under ligand-assistance (Scheme 180E).^1158^ To further illustrate the synthetic value and versatility of the above-presented projects, Gaunt proposed a new retrosynthetic route towards K-252 (stauroeporinone) implying two amine-directed C–H activation events, *i.e.* Cu-catalysed *ortho*-arylation of dibenzyl *para*-toluidinearyl iodane, followed by aromatic carbonylation/intramolecular C–C bond formation.^1159^

**Scheme 181:**
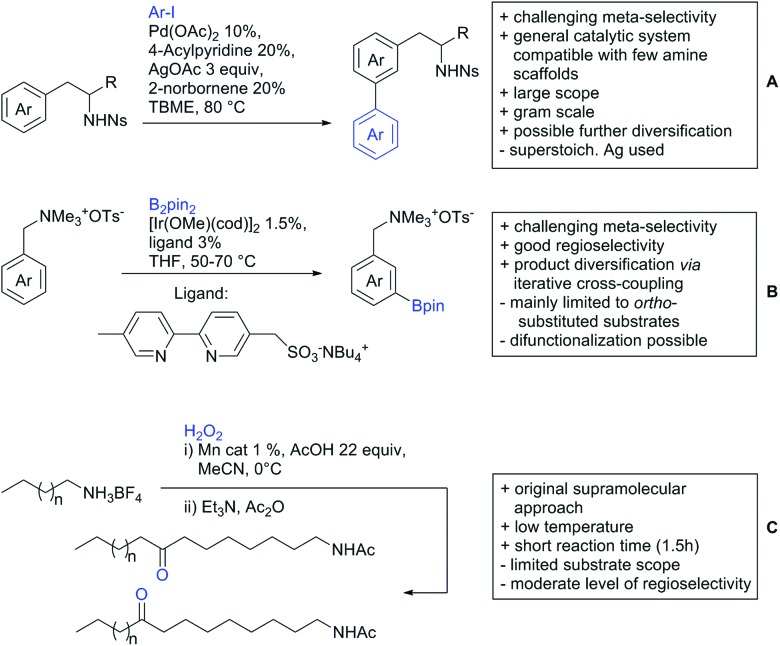
Amine-directed remote functionalisations.

In addition to this elegant work on amine-free directed C–H activation, several examples of C–H transformations based on the use of *N*-protected DGs have been reported. In particular, nosyl-protected phenylethylamine, benzylamines and 2-aryl aniline derivatives could be successfully used in Catellani-inspired^1160^*meta*-selective arylation (Scheme 181A).^1161^ The catalytic system combining Pd-catalyst and a pyridine-derived ligand promotes *meta*-functionalisation thanks to the participation of norbornene in a catalytic cycle. The scope of this transformation is rather remarkable, as not only three substrate scaffolds are effective, but also a diversity of both electron-rich and electron-poor aryliodanes may be used, although the products are generally isolated in rather modest to good yields.

Quaternary ammonium salts have also met growing interest in the context of direct C–H functionalisation. The cationic nature of this moiety renders it particularly appealing in devising alternative, supramolecular strategies for C–H activation. In 2016 Phipps focused on direct C–B coupling catalysed by Ir-bipyridine type complex (Scheme 181B).^1162^ This research group stipulated that a quaternary ammonium moiety present on a substrate should participate in ion-pairing interactions with a modified bipyridine ligand bearing an anionic substituent. Such DG–ligand interactions should thus direct the Ir-catalyst to a more distal position, yet allowing remote C–H activation. Rewardingly, it was found that an Ir-catalyst coordinated by a bipyridine ligand with an appended sulfonate tether participates in strong ionic interactions with a quaternized benzylamine DG, consequently validating the initial hypothesis. This catalytic system allowed formation of the expected *meta*-borylated aromatics in high yields and generally high regioselectivity. The reaction is particularly efficient if *ortho*-substituted substrates are used and for unsubstituted precursors double borylation might be expected. In addition, as the ammonium moiety can be further valorized *via* Pd-catalysed C–C couplings, the newly obtained products are highly modular. The concept of ion pair-directed C–H functionalisation is also effective for *meta*-selective borylation of benzylamine, phenylethylamine and phenylpropylamine-derived amides.^1163^

An alternative approach based on supramolecular recognition between a cationic amine-type DG and a catalyst was disclosed recently by Olivo, Di Stefano and Costas (Scheme 181C). In order to achieve remote and site-selective functionalisation of aliphatic chains, a bioinspired catalyst, *i.e.* Mn complex equipped with 18-benzocrown-6 ether receptors was designed. Reversible preassociation of the crown ether with the protonated aliphatic primary amines approaches C8 and C9 positions of the aliphatic chain to the Mn-atom, yet enhancing regioselective oxidation. Remarkably, the length of the aliphatic chain does not impact the reaction outcome. This conceptually unprecedented transformation is, for the moment, limited to simple, non-substituted substrates and the regioselectivity level still needs improvement.^1164^

Finally we would like to highlight a recent contribution by Suzuki and Yamashita who designed original aluminabenzene–Rh and Ir complexes. Catalytic activity of this new Ir-based complex was evaluated studying C–H borylation of aliphatic tertiary amines and *N*,*N*-diethylbenzylamine.^1165^

### 16.9. Nitrone as DGs

**Scheme 182:**
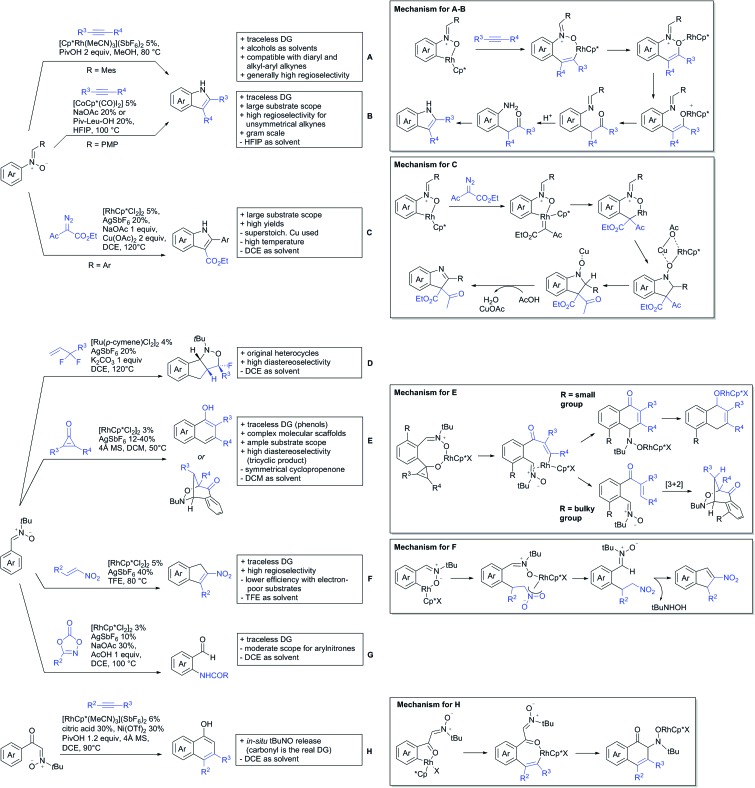
Nitrone-based DGs.

In 2013, the panel of coordinating motifs employed as DG was further expanded, as a potential of nitrones to precoordinate a metal catalyst yet directing its insertion into a particular C–H bond was evidenced by Li.^1166^ Following this pioneering report, several examples of nitrone-directed couplings with alkenes, alkynes, diazo compounds and arenes were reported till 2015. More recently, several research groups astutely used this coordinating motif to build up heterocyclic compounds, and in particular indole derivatives. In 2015 Liu and Lu discovered that nitrone may play the role of an oxidizing DG in RhCp*-catalysed direct coupling with alkynes (Scheme 182A).^1167^ This redox-neutral C–H annulation occurs in absence of an external oxidant affording an array of 2,3-disubstituted indoles with moderate to high yields. Substituents such as CN, F, Br or OMe are well tolerated on the aromatic ring and both aryl–aryl and alkyl–aryl disubstituted alkynes are effective. Targeting more reliable and industrially appealing transformations, Ackermann and collaborators designed a closely related Co-catalysed transformation (Scheme 182B).^1168^ This redox-neutral coupling between nitrones and alkynes, proceeding *via* C–H/N–O functionalisation proceeds smoothly when a cationic CoCp*-catalyst is used in combination with either sodium acetate or an amino acid ligand. The reaction displays an excellent site- and regio-selectivity with unsymmetrical nitrones and alkynes, shows outstanding FG tolerance and is easily scalable.

Alternatively, indole synthesis may also be achieved using a RhCp*-catalyst in combination with diazo compounds as coupling partners (Scheme 182C).^1169^ In contrast to the previous examples, this transformation requires a substoichiometric amount of Cu(OAc)_2_ as oxidant but the substituent present on the nitrone is conserved in a final product (as C2-substituent of the indole scaffold). Accordingly, this strategy is of particular interest if indoles bearing an aromatic moiety at C2 position and an ester group at C3 are targeted.

In 2018 Li astutely used nitrone DG to construct fused isoxazolidines (Scheme 182D).^1170^ The corresponding regio- and diastereoselective C–H coupling of nitrones with perfluoroalkylolefines delivers rare heterocyclic compounds with three continuous stereogenic centers and features remarkable atom economy. The reaction proceeds under Ru(ii)-based catalysis and, although rather harsh reaction conditions are required (temperature of 120 °C) it is compatible with an array of functional motifs, delivering the desired products in moderate to good yields.

A traceless character of the nitrone moiety is an additional key advantage, of particular importance for the C–H activation field. This purpose was elegantly illustrated by Li when conceiving a new synthetic route towards 1-naphthols, based on RhCp*-catalysed coupling of *N-tert*-butyl nitrones with cyclopropenones (Scheme 182E).^1171^ In this original reaction strained cyclopropenones are used as C3-type coupling partners and the catalytic cycle is believed to be completed by *in situ* removal of the nitrone moiety delivering 2,3-disubstituted naphthols. Besides, an alternative scenario is operative for *ortho*-substituted nitrones; a C–H acylation step is followed by [3+2] dipolar cyclo addition pathway, affording complex bicyclic structures. Noteworthy, the reaction occurs under oxidant free conditions and compounds otherwise difficult to access may now be constructed efficiently and straightforwardly. Naphthols may also be obtained *via* direct coupling between α-carbonyl nitrones and alkynes (Scheme 182H).^1172^ This redox-neutral RhCp*-catalysed transformation also implies *in situ* cleavage of the *tert*-butyl nitrone moiety. However, contrary to the previous example, symmetric 3,4-disubstituted naphthols are generated. It should be noted however that in this particular case the carbonyl moiety is rather expected to precoordinate the catalyst and direct the metallation event into the *ortho*-position.

In 2017, a nitrone-directed C–H activation protocol was used to construct indenes (Scheme 182F).^1173^ This reaction implies a direct coupling with internal nitroalkenes and, likewise previous examples, the *N-tert*-butylnitrone moiety is released *via* a Henry-type reaction delivering an ample selection of nitro-functionalised indenes, convertible *via* reduction into the corresponding ketones.

To complement the portfolio of direct functionalisation reactions compatible with nitrone DGs, Li and Wang developed site-selective amidation (Scheme 182G).^1174^ This RhCp*-catalysed transformation implies use of 1,4,2-dioxazol-5-ones as coupling partners and the DG decomposes at the end of the reaction releasing *ortho*-functionalised aldehydes. The optimized protocol allows installation of various amide moieties and several functionalities are also tolerated on the aromatic backbone furnishing the desired products in rather high yields.

## 17. Conclusions and outlook

It can be seen that the field of DG assisted catalytic C–H functionalisation has come a long way since its beginnings more than two decades ago. Most of the FGs you can think of have been used in one or the other example as DG in one or more transformation. This allows making the statement that most of the compounds which occur in a multistep sequence carry a FG which can be exploited as DG in C–H functionalisation chemistry, at least in principle. The question remains why the methodology is still not explored more frequently in complex synthesis or in R&D processes. The following critical outlook discusses the shortcomings of C–H functionalisation, which need to be addressed in the future.

C–H functionalisation chemistry is very often said to be the future technology replacing cross coupling reactions because of its inherent potential for greenness in comparison to the latter. As green chemistry is a major concern for industry, considerations related to its future application in process chemistry need to be made. Nowadays cross coupling reactions can be performed in a variety of (green) solvents, under air, with catalyst loadings lower than 0.5%, catalytic amounts of ligands and stoichiometric bases. These reactions are now routinely used in chemical development and production of fine chemicals. C–H functionalisation, instead, still requires very often specific cocktails of a number of compounds (as discussed in the introduction), often under inert atmosphere, and additionally necessitate installation and cleavage of a DG, unless already present in the starting materials or found in the target product. The demonstration of the cleavage of a DG was specifically highlighted throughout this review since in most examples DG cleavage has not been investigated. Consequently, progress in the area of cleavable or traceless DGs is highly desirable in the near future.

Apart from these issues, less obvious limitations appear evident from the analysis of the literature in the field of C–H functionalisation. For example, a large number of protocols make use of toxic solvents, in particular DCE and other halogenated solvents are widely used. Although other solvents might be used as replacements in some cases, these are normally not thoroughly investigated, and often result in much lower yields. The efficiency of the reaction in more sustainable solvents is therefore not guaranteed. In a typical process the solvent accounts for 50–60% of the entire mass,^1175^ and the use of greener media is therefore of major importance in chemical development programs, although still less studied in academia. In case a C–H functionalisation protocol would be of interest to industry, the process chemists will have to spend considerable efforts in finding a valid replacement solvent before even considering using such a protocol. While investigations on green solvents are nowadays common for many type of reactions, this is not yet the practice for C–H functionalisations. Nonetheless, several examples were reported recently of functionalisations in alcoholic media, and some in alkyl acetates. Both these classes of compounds are listed as preferred solvents in solvent guides.^1176^,^1177^ In particular, the latter type of solvents has been used in a variety of transition metal-catalysed reactions,^908^,^1178^–^1182^ and EtOAc has for instance been demonstrated by Chang to be an effective replacement of DCE in a larger scale (200 mmol) C–H amidation protocol.^751^ This will hopefully inspire further research in this direction.

Regarding the metal catalysts which dominate the field, we still see Pd, Rh, and Ru as the three major players. In a society ever more aware of environmental issues, it is highly desirable to substitute these metals for more abundant ones. Consequently, the number of examples using Fe, Co, Ni, Cu, and Mn are increasing. Not only are these metals less toxic (with the exception of Co, see below), but also their extraction from soil is much simpler and requires less energy or less hazardous chemicals. Hence, as in all fields of metal catalysis, the quest for switching from precious metals to more abundant ones is ongoing and one of the big driving forces of innovation.

Sticking to the issue of the catalyst another important aspect is the often high catalyst loading necessary for C–H functionalisation reactions. Although many protocols utilise lower amounts, it is not uncommon to find a loading of 10% [total metal] in Pd, Rh, Ru or Ir catalysed reactions. While such loadings are still acceptable when using base metals, efforts should be devoted to lowering of these amounts when using noble metals. Aside from matters of cost, the accepted residual levels in *e.g.* formulated drugs, based on permitted daily exposure (PDE), are typically higher for base metals (*e.g.* Cu and Fe) than for noble metals.^1183^ On the other hand, although base metal catalysis is developing quickly in this field, in many cases up to 20% of the metal is used, and several protocols are also reported making use of stoichiometric amounts of metal (*e.g.* for Cu). Catalyst recycling is not often considered in academic publications, but a few interesting examples of heterogeneous catalysts for C–H functionalisation have been reported, which allow easy catalyst recycling and reuse.^1184^ Research in this direction, involving both heterogeneous and homogeneous catalysts, should be developed further.^1185^ Concerning base metals, a very important note has to be made: base metals are not by definition less toxic. In fact, despite the growing use of Co catalysis in C–H functionalisation, Co is not a low-toxicity metal, and the permitted daily exposure for this metal is actually lower than for noble metals used in catalysis such as Pd, Rh, Ru and Ir.^1183^

Concerning the regioselectivity of C–H functionalisation reactions, most examples of DGs listed in this review are *ortho*-DGs, with respect to aromatic C–H functionalisation. This has been identified as a shortcoming and several groups have started to address this issue. A handful of *meta*- and *para*-DGs have been reported and this area will without doubt see more examples in the near future.

Other selectivity issues have also been identified. It is true that regioselectivity, for example on an aromatic substrate, is achieved using a DG, but monofunctionalisation is often challenging. Many of the protocols included in this review produce in several cases mixtures of mono and difunctionalised compounds. This is common, for example, in arylation and olefination reactions, while with other functionalities this is less of a problem. In the less selective cases, monofunctionalisation is generally achieved by blocking (substitution) one of the reactive positions, or of a contiguous one (*e.g.* in arenes substituted in the 5 position, the DG in position 1 often selectively promotes functionalisation in position 2 based on steric reasons). Although this might be considered an acceptable solution in academia, this will be more important to tackle when these protocols are used in the synthesis of specific target molecules, where this specific substitution pattern might not be required/desired. Another strategy to deal with difunctionalisation found in the academic literature is the use of an excess of the DG-equipped substrate to avoid difunctionalisation. Although this might not be a big problem *per se* (as the substrate can be easily recovered), the excess substrate is often the most complex and expensive molecule in the mixture (*e.g.* it contains the DG), and therefore this is also not the ideal solution as efficient separation avoiding column chromatography might be tricky.

Furthermore, in some transformations, the applied coupling partners to date leave significant room for improvements. As an example, arylation reactions are often performed with the use of aryl iodides, which the fine chemical industry typically avoids, while relatively few examples currently involve the use of cheaper, widely available and less wasteful aryl chlorides.

At a more general level, stereoselective transformations for the formation of chiral quaternary centres are still quite limited. Enantioselectivity is here achieved using a chiral ligand, and several examples demonstrate that good to excellent selectivity is possible. The mechanism-guided stereoselective formation of alkenes (*e.g.* after Fujiwara–Moritani reactions or hydrofunctionalisation of alkynes) is often not achieved and mediocre *E*/*Z* ratios are obtained in many cases. As alkenylation is one of the most studied transformations, we believe more emphasis should be put in the development of selective strategies. Similarly, the hydrofunctionalisation of unsymmetrical alkynes usually results in regioisomeric mixtures.

Important advancements are being made concerning the use of aerobic conditions for oxidative couplings. Despite the safety issues related to the use of air/O_2_ in a chemical process (especially in development and production scale),^1186^ the use of oxygen results in higher atom economy and cheaper processes, especially in comparison to the use of other (super)stoichiometric Ag and Cu salt oxidants (utterly common for group 8–10 metal catalysis, see also introduction). Sometimes O_2_/air is used in combination with Cu salt allowing for their use in catalytic amounts. The variety of reported oxidants is anyway limited.

While a huge variety of C(sp^2^)–H functionalisation protocols have appeared, C(sp^3^)–H functionalisation still lags behind, due to higher inherent difficulty in activating these bonds. Nonetheless, important developments have been brought forward by a number of groups, mostly using bidentate amides as DGs. These transformation in many cases require external ligands (such as monoprotected amino acids, MPAA), and conformational restraints (aliphatic rings as substrates or Thorpe–Ingold effect) to be efficient. This field is however very promising, due to the many different compounds potentially obtained *via* this strategy.

It has been stated that many of the most frequently occurring FGs have been explored as DGs in C–H activation chemistry. This offers on the one hand great potential in applications, but on the other hand it also raises questions regarding selectivity, in cases a substrate carries more than one potential DG. Hence, research focused on exploring such substrates and eventually developing orthogonal sets of conditions in which first DG1 and then DG2 exhibits its directing potential would be highly interesting. However, such reports are scarce^192^,^1031^,^1187^–^1190^ and much more work in this direction would be highly desirable, since it would increase the number of cases in which C–H functionalisation could be practically applied.

This very critical discussion is not meant to discourage the use or development of this type of chemistry. Quite the contrary in fact! We hope that by critically analysing the weak points of this technology, research groups around the world would realise where important advancements need to be made, and focus their research not just in developing higher-yielding reactions, changing the metal for catalysis, or developing new reagents/reactants, but also in making these protocols more general, selective, and actually greener and industry-appealing.

## List of abbreviations

@: Supported on
acac: Acetylacetonate
Ac_2_O: Acetic anhydride
AcOH: Acetic acid
AdOH: Adamantane carboxylic acid
AIBN: Azobisisobutyronitrile
Ala: Alanine
ampaphos: *t*Bu_2_PCH_2_P(*o*-C_6_H_4_OMe)_2_
APAQ: Acetylprotected aminoethyl quinolone
Ar_F_: Pentafluorophenyl
BADME: Bis(2-dimethylaminoethyl)ether
B_2_eg_2_: Bis(ethylene glycolato)diboron
B_2_pin_2_: Bis(pinacolato)diboron
BarF_4_- or BARF: Tetrakis[3,5-bis(trifluoromethyl)phenyl]borate
9-BBN: 9-Borabicyclo(3.3.1)nonane
bipy: 2,2′-Bipyridine
Boc: *tert*-Butoxycarbonyl
BP: Benzophenone
BQ: 1,4-Benzoquinone
Brij 35: Poly(oxy-1,2-ethanediyl), α-dodecyl-ω-hydroxy-polymer
BTP: 2-Bromo-3,3,3-trifluoropropene
CAN: Ceric ammonium nitrate
CDC: Cross dehydrogenative coupling
cod: Cyclooctadiene
Cp*: 1,2,3,4,5-Pentamethylcyclopentadienyl
CpE: Diethyl-2,4,5-trimethylcyclopentadienyl-1,3-dicarboxylate
*m*-CPBA: *m*-Chloroperbenzoic acid
CPME: Cyclopentyl methyl ether
Cy: Cyclohexyl
cymene: *p*-Cymene
cym: *p*-Cymene
DABCO: 1,4-Diazabicyclo[2.2.2]octane
dba: Dibenzylideneacetone
DBE: Dibutylether
DCB: 1,2-Dichlorobenzene
2,3-DCB: 2,3-Dichlorobutane
DCDMH: 1,3-Dichloro-5,5-dimethylhydantoin
DCE: Dichloroethane
DCP: Dicumyl peroxide
DCM: Dichloromethane
dcpm: Bis(dicycloheylphosphine)methane
DG: Directing group
DIAD: Diisopropyl azodicarboxylate
DIBAL-H: Diisobutylaluminium hydride
DIOP: *O*-Isopropyliden-2,3-dihydroxy-1,4-bis(diphenylphosphino)butan
DIPEA: *N,N*-Diisopropylethylamine
dippz: 1,2-Bis(diisopropylphosphino)benzene
DMAc or DMA: Dimethylacetamide
2,6-DMBQ: 2,6-Dimethoxy-1,4-benzoquinone
DME: 1,2-Dimethoxyethane
DMF: Dimethylformamide
DMI: 1,3-Dimethyl-2-imidazolidinone
DMPU: 1,3-Dimethyl-3,4,5,6-tetrahydro-2(1*H*)-pyrimidinone
dppb: 1,4-Bis(diphenylphosphino)butane
dppe: 1,2-Bis(diphenylphosphino)ethane
dppen: *trans*-1,2-Bis(diphenylphosphino)ethene
dr: Diastereomeric ratio
drs: Diastereomeric ratios
DTBEDA: *N*,*N*′-Di-*tert*-butylethylenediamine
dtbpy: 4,4′-Di-*tert*-butyl-2,2′-dipyridyl
EBX: Ethynyl-1,2-benziodoxol-3(1*H*)-one
EDG: Electron donating group
ee: Enantiomeric excess
EWG: Electron withdrawing group
FG: Functional group
Gly: Glycine
HBpin: Pinacolborane
hfacac: Hexafluoroacetylacetone
HFIP: Hexafluoroisopropanol
HODmb: 2,2-Dimethylbutyric acid
Ile: Isoleucine
IPr-HCl: 1,3-Bis(2,6-diisopropylphenyl)imidazolium chloride
LDA: Lithium diisopropylamide
LED: Light-emitting diode lamp
Leu: Leucine
LiHMDS: Lithium bis(trimethylsilyl)amide
Men: Menthyl-OC(O)
Me_2_-TP: 4-(Bis(2-(diphenylphosphanyl)phenyl)phosphanyl)-*N*,*N*-dimethylaniline
Mes: Mesitylene
MOF: Metal–organic framework
MPS: Methyl phenylsulfoximine
MTBE: Methyl *tert*-butyl ether
MS: Molecular sieves
*o*-NBA: *o*-Nitrobenzoic acid
nbd: Bicyclo[2.2.1]hepta-2,5-dienyl (= norbornadiene)
NBE: Norbornene
NBS: *N*-Nromosuccinimide
NCTS: *N*-Cyano-*N*-phenyl-*p*-toluenesulfonamide
nep: Neopentyl glycolato
NFSI: *N*-Fluorobenzenesulfonimide
NHC: N-Heterocyclic carbene
NHPI: *N*-Hydroxyphthalimide
NIS: *N*-Iodosuccinimide
NMO: *N*-Methylmorpholine-*N*-oxide
NMP: *N*-Methyl-2-pyrrolidone
Nphth: Phthalimide
NXS: *N*-Halosuccinimide (X = halogen)
ODCB: 1,2-Dichlorobenzene
*o*-PBA: *o*-Phenylbenzoic acid
OTf: Trifluoromethanesulfonate
PCC: Pyridinium chlorochromate
PE: 2-(2-Pyridyl)ethylamine
PEG: Poly ethylene glycol
PEPPSI-IPr: [1,3-Bis(2,6-diisopropylphenyl)imidazol-2-ylidene](3-chloropyridyl)palladium(ii) dichloride
1,10-phen or phen: 1,10-Phenanthroline
Phe: Phenylalanine
PIDA: Phenyliodine diacetate
pin: Pinacolato
PivOH: Pivalic acid
PM: 2-(Aminomethyl)pyridine
PTSA: *p*-Toluenesulphonic acid
PyO: Pyridyl-*N*-oxide
rt: Room temperature
SDS: Sodium dodecylsulfate
SIXyl·HCl: Bis(2,6-dimethylphenyl)imidazolinium chloride
SPGS-550-M: β-Sitosterol methoxypolyethyleneglycol succinate
TAM: Triazolyldimethyl
*t*AmOH: *tert*-Amylalcohol
TBAA or TBAOAc: Tetrabutylammonium acetate
TBAB: Tetrabutylammonium bromide
TBAF: Tetra-*n*-butylammonium fluoride
TBAI: Tetrabutylammonium iodide
TBHP: *tert*-Butylhydroperoxyde
TBPB: *tert*-Butyl peroxybenzoate
TBS: *tert*-Butyldimethylsilyl
TCC: Trichloroisocyanuric acid
TCP: 1,2,3-Trichloropropane
TEMPO: (2,2,6,6-Tetramethylpiperidin-1-yl)oxyl
TFA: Trifluoroacetic acid
TFE: 2,2,2-Trifluoroethanol
THF: Tetrahydrofurane
THP: Tetrahydropyran
TIPS: Triisopropylsilyl
TMBzOH: 2,4,6-Trimethylbenzoic acid
TMEDA: Tetramethylethylenediamine
TMS: Trimethylsilyl
TON: Turn over number
Tween20: Sorbitan, monododecanoate, poly(oxy-1,2-ethanediyl) derivatives
Val: Valine
XPhos: 2-Dicyclohexylphosphino-2′,4′,6′-triisopropylbiphenyl

## Conflicts of interest

There are no conflicts to declare.

## Supplementary Material

Supplementary information
