# Supplementary material for: A comprehensive overview of directing groups applied in metal-catalysed C–H functionalisation chemistry
Source: Chem Soc Rev. 2018 Jul 23;47(17):6603–743. doi: 10.1039/c8cs00201k (PMC6113863; doi:10.1039/c8cs00201k)
Supplement: Supplementary file 1 [file CS-047-C8CS00201K-s001.pdf]

Electronic Supplementary Material (ESI) for Chemical Society Reviews.
This journal is © The Royal Society of Chemistry 2018

# Supplementary material to:

# A comprehensive overview on directing groups applied in metal catalyzed C-H functionalization chemistry

Carlo Sambiagio,[1] David Schönbauer,[2] Remi Blieck,[3] Toan Dao-Huy,[2] Gerit Pototschnig,[2] Patricia Schaaf,[2] Thomas Wiesinger,[2] Muhammad Farooq Zia,[2] Joanna Wencel-DeLord,[4] Tatiana Besset,[3] Bert U. W. Maes,[1] Michael Schnürch[2,*]

[1] Organic Synthesis, Department of Chemistry, University of Antwerp, Groenenborgerlaan 171, B-2020 Antwerp, Belgium

[2]Institute of Applied Synthetic Chemistry, TU Wien, Getreidemarkt 9/163, A-1060 Vienna, Austria

[3]Normandie Univ, INSA Rouen, UNIROUEN, CNRS, COBRA (UMR 6014), 76000 Rouen, France

[4] Laboratoire de Chimie Moléculaire (UMR CNRS 7509), Université de Strasbourg, ECPM 25 Rue Becquerel, 67087 Strasbourg, France

michael.schnuerch@tuwien.ac.at

## Content

Introduction ........ 3

Heterocyclic directing groups ........ 4

Pyridine ........ 5

Bidentate directing groups based on heterocycles ........ 19

Pyrimidine as directing group in C-H activation chemistry ........ 44

Pyrazole derivatives in C-H activation chemistry ........ 57

Triazole derivatives in C-H activation chemistry ........ 65

Tetrazole derivatives in C-H activation chemistry ..... 72

Oxazole based DGs ..... 77

Heterocyclic and related directing groups in C-H activation chemistry ..... 86

Amides as directing groups ..... 95

*N*-Methoxy amides as directing group ..... 111

N-Acyl- containing directing groups ..... 119

Directing groups containing the carbonyl motif ..... 126

Aldehydes as directing groups in C-H activation ..... 126

Carboxylic acid- based directing groups ..... 131

Carboxylic Esters ..... 146

Ketones ..... 151

Hydroxyl- and Phenol- based derivatives ..... 164

Oxime and Oxime derivatives as directing groups in C-H activation chemistry ..... 171

Ketoxime ..... 181

Aldoximes and aldoxime ether ..... 186

Phosphorous-containing directing groups ..... 187

Si-containing directing groups ..... 195

Azo-containing directing groups ..... 199

## Introduction

This supporting material covers literature in the field of directing group assisted C-H functionalization published until 2015. Since the main manuscript covers only literature from 2015 onwards. The SI is mainly organized as tables which are first of all organized according to the type of directing group. Selected examples of each directing group are discussed in the accompanying text sections. In cases where it helps the general understanding of a transformation, additional schemes or figures are added, for example to explain an important mechanism. In the first column of the tables (besides the *Entry* column) the structure of the directing group is displayed. In the second column the type of transformations which have been reported with this directing group will be listed in alphabetical order (e.g. alkylation, arylation, nitration, trifluoromethylation, etc.). Here it has to be mentioned that sometimes different publications use different terms for the very same transformation. For the sake of simplicity, in this review each transformation has the same name in all entries. For example, the coupling of olefins with arenes to give alkylated arenes is either classified as alkylation or hydroarylation. It was decided to look at the reaction product and see what happened to the part of the molecule which carried the DG. Hence, in case the arene carried the DG, the reaction is always classified as alkylation reaction. In the third column the reaction/coupling partner is listed. For some transformations, numerous examples have been reported with a specific directing group. For example, the direct arylation of ketones in ortho position knows many examples in the literature. It was aimed at being comprehensive in the regard that all DGs and potential transformations with the DG are listed, however, reporting every single variant of a given transformation would have been beyond the scope. In such cases typically the first report and important further developments are listed in the tables. For example, if originally for arylation reactive aryl iodides were required, relevant further examples would make more readily available aryl chlorides accessible for the same transformation, just to give an example. It is clear that such a selection will always be biased and you might find that one or the other example should have been selected differently.

In the fourth column the structure of a typical product of the C-H activation reaction will be shown with the newly formed bond clearly indicated (bold or in color). In the fifth column general comments to the specific transformation are given. Most importantly which metal catalyst was required, was there a crucial additive (e.g. a specific base), or which solvent had to be applied. Additionally, information whether the directing group is cleavable or not is included here as well. Finally, in the sixth column the reference to the original research paper is given.

The format with a strong focus on tables and the aim to give a comprehensive overview on all DGs which have been applied in the past brings it about that not every contribution which is listed in a table can be discussed in the text. Discussions have been limited to examples of special interest, e.g. by establishing a new DG or a new catalytic system applicable to a manifold of other transformations. Such a selection made by the authors is naturally biased and not all readers will agree with the selections which have been made. However, once again it should be noted that the most important part are the tables and the information compiled therein.

The speed in which new contributions are brought forward in the field is amazing. This shows the high relevance of this area of research and the potential scientists see in it. For writers of a review it brings certain problems as well, most importantly when to make the cut for selecting contributions to be included. It was decided to include all original papers (full articles and communications) until the end of 2015.

## Heterocyclic directing groups

The common feature of all the directing groups discussed in this section is that they contain a basic nitrogen which precoordinates the metal center. The most common representative in this area is without doubt pyridine. 2-Phenylpyridine is the single most applied starting material in C-H activation chemistry since it is very often used as test system to test a hypothesis for a viable new transformation and to optimize the required reactions conditions, before the method is then expanded to other directing groups. Hence, pyridine could make up for the largest chapter of this review and it could be expected that reviews on the direct functionalization of 2-phenylpyridine have been published in the past. However, this is not the case. Of course, 2-phenylpyridine finds its way into most of the C-H activation reviews in recent years, but not as prominent as the amount of work on this system would suggest. One reason is the limited synthetic potential of substituted 2-phenylpyridines. The DG cannot be cleaved from the arene system and hence, applying pyridine as DG in the framework of complex synthesis is limited to structures which actually do contain the 2-phenylpyridine motif. The same is true for a number of systems, which can be considered as close structural relatives of 2-phenylpyridine, and which are displayed in Scheme 1. It is not uncommon, that contributions dealing with 2-phenylpyridine activation also discuss some of the other examples of Scheme 1, and typically the same reaction conditions can be used without further optimization. All of these substrates have the same problem already mentioned, the permanent nature of the directing group, and hence their limited general applicability. Hence, these systems do not fit into the general goal of this review, as defined in the introduction, and it was decided to largely exclude them from coverage. Only selected examples will be reported, in case these examples fit the purpose of this review, to promote the application of C-H activation chemistry in complex synthesis. This is for example typically the case when conditions for cleaving the heterocyclic DG have been reported, which can be typically done when e.g. pyridine is not bound via a C-C bond to the system to be activated, but by a $C_{pyridine}$-$Hetero_{Substrate}$ bond.

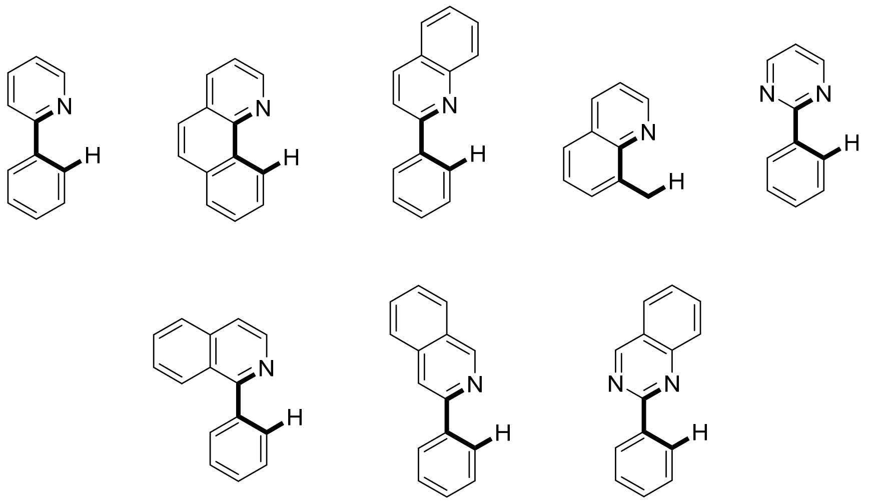

**Scheme 1: 2-Phenylpyridine and related substrates.**

Reports dealing with heterocycle directed C-H activation are in most cases not only dedicated to a single heterocycle, but show the applicability of a method to several heterocycles. Most frequently, pyridine is first established as DG and the substrate scope of a given transformation is mainly shown with this DG. Then, the same set of reaction conditions is used to demonstrate also the applicability of other heterocycles, most frequently pyrimidine, but also pyrazole is an often used example. However, typically for these other heterocycles much less substrate scope explorations have been carried out. Still, cases of such contributions which are of special interest since for example a new heterocyclic DG is reported for the first time are included in this review, even if it might just be a single example.

## Pyridine

As already mentioned, pyridine is the most frequently applied DG in C-H activation chemistry. However, only few examples have been reported in which the pyridine scaffold can be cleaved from the final products, or which show at least the potential for cleaving the DG. In these examples pyridine is typically attached to a heteroatom of the scaffold to be activated.

The group of Maes reported pyridine directed arylation of piperidine derivatives using the well-established $Ru_3(CO)_{12}$ catalyst for this transformation (Table 1, Entry 18). Interestingly, they report a different mechanism than previously described for the same transformation by Sames, although promoted by a different DG (Table 1, Entry 16).[1] Mechanistically, the catalyst seems to be promiscuous, since in another study, the group of Schnürch showed that direct arylation of amines (this time acyclic ones) works under conditions which should favor the Meas mechanism (Table 1, Entries 33, 37-39), but also under conditions which would only allow the mechanism reported by Sames.[2, 3] The method of Maes leads to mixtures of mono- and bis-arylation in cases of simple piperidine, often as 1:1 mixtures (combined yields around 70%). If one α-position is blocked, naturally only mono-arylation occurred and 48-91% of products were obtained. In their paper, they also report a two-step protocol for cleaving the pyridine DG. Initially, it is reduced to a cyclic imine, which can then be hydrolyzed by the conditions reported by Sames previously.[1] In a later paper, they reported alternative cleavage protocols, namely hydrogenation-hydride reduction or quarternization-hydride reduction, adding to the small pool of pyridine removal conditions two more options.[4] To overcome the problem of mixtures of mono- and bis-functionalization products being formed was addresses by the group of Schnürch by using a 3-trifluoromethylated pyridine DG (Table 1, Entry 38).[5] Indeed, only mono-arylation was observed, which facilitated isolation of the products significantly. However, overall conversion remained low and only 60% yield in the best example was obtained. Cleavage of the modified DG required the removal of the $CF_3$ group since the reduction and hydrolysis protocol did not lead to any cleavage. Interestingly, after the reduction step, simple stirring the intermediate in DCM with silica gel led to quantitative removal of the $CF_3$ group, a thus far unprecedented reaction.

The group of Schnürch disclosed three different arylation protocols of a common type of substrate using different aryl sources (Table 1, Entries 33-35, 37-41).[2, 3, 6, 7] Under Ru(0)-catalysis and neutral conditions, arylboronic acid esters were used as coupling partners (Table 1, Entries 33, 37-39).[2, 3] Expanding the method to aryl bromides and iodides required a Ru(II) catalyst and addition of base (Table 1, Entries 34 & 40 ).[6, 7] The requirement for addition of KOPiv suggests that the reaction now proceeds via a

CMD mechanism. In contrast, when aryl chlorides are used as aryl source, KOPiv is not tolerated but a phosphine ligand ($PPh_3$) gives best results, still under Ru(II) catalysis and under basic conditions (Table 1, Entries 35 & 41).[6] Additionally, the addition of a secondary alcohol is required, which acts as transfer hydrogenation agent, reducing an imine byproduct to the desired product.

Direct ortho-arylation of anilines with boronic acids as the aryl source was reported by Schnürch and coworkers (Table 1, Entry 49).[8] Also in this case, the two-step reduction-hydrolysis protocol originally applied by Maes could be used for cleaving the pyridine DG from the aniline amino group. The reaction conditions required $Ag_2O$ as oxidant and benzoquinone, which seems to act as oxidant as well, but also as an important ligand in the transformation.

Direct arylation of *N*-2-pyridyl carbazoles was reported by Wu and coworkers. Unsubstituted carbazoles ($R^{1,2}$ = H) gave high yields of mono-arylation products. If one phenyl ring was already substituted, the nature and position of the substituent had a large effect on the outcome. A nitro group *meta* to the carbazole N led to exclusive arylation of the unsubstituted phenyl ring, whereas the same substituent in para position gave a mixture of two products. The electron donating methoxy group always led to mixtures of three products (mono-arylation of the MeO carrying ring + mono-arylation of the unsubstituted ring + bis-arylation). Other *meta* substituents (always relative to the carbazole N) such as *t*Bu and COMe only gave mono-arylation in the unsubstituted phenyl ring.

An interesting indole synthesis was reported by Wu and coworkers (Table 1, Entry 28).[9] *N*-2-pyridyl anilines were reacted with alkynes using a simple system of Pd on $CeO_2$ under air giving an operationally quite simple protocol. In case of aryl-alkyl alkynes, the alkyl residue ended up in position 3 of the indole with good selectivity (7:1 or better). Alkynes bearing two different alkyl groups were not selective at al.

Cobalt catalyzed C-H activation has gained prominence in recent years. Ackermann and coworkers reported the direct arylation (Table 1, Entry 20) and benzylation (Table 1, Entry 22) of indoles at relatively mild conditions, however in a relatively rarely applied solvent, namely DMPU.[10] The need for 2 equiv of a Grignard species limits naturally functional group tolerance.

A rare example of alkylation with an alkyl boron reagent was reported by the group of Li (Table 1, Entry 10).[11] Under Rh-catalysis, alkyl-$BF_3K$ salts were efficiently reacted with indoles carrying pyridine as DG, amongst others. Especially interesting are the high yields in methylation reactions, since other alkylation protocols relying on alkenes as alkyl source do not give access to methylation products.

Cyanation in position 2 of indoles using *t*BuNC as the cyano source was reported by Xu and coworkers (Table 1, Entries 25 & 36).[12] The DG overrides the intrinsic reactivity of indoles, where the more electron rich 3-position would be preferentially cyanated in absence of a DG.

Oxidative coupling between indoles or pyrrole and N-oxides of quinoline, quinoxaline, and pyridine was reported by You and coworkers (Table 1, Entry 26).[13] Such transformations are always attractive because no leaving group at all is required and basically two C-H bonds are used for the formation of a new C-C bond.

The group of Loh reported oxidative alkenylation (Table 1, Entry 2) and alkynylation (Table 1, Entry 12) under similar conditions using a simple and commercially available Rh catalyst.[14] In both cases, the substrate scope regarding functional group tolerance was remarkable. As substrates indolines were used, and the new C-C bond formation took place in position 7. This is in contrast to indole, where similar methods lead to functionalization in C2 position. For both types of products, the cleavage of the DG was demonstrated as well.

A comprehensive study of [MnBr(CO)$_5$] catalyzed amidation of indoles with isocyanates was reported by the group of Ackermann (Table 1, Entry 14). Mn as catalyst is of course highly attractive and also the functional group tolerance was very good. Even such reactive groups as iodine were well tolerated. A small drawback are the temperature and pressure conditions required, since 100 °C in diethyl ether require special equipment not withstand the pressure built up in the reaction. The same group developed another non-precious metal catalyzed amidation method, this time using a Co catalyst, with 3-substituted 1,4,2-dioxazol-5-ones as amide source (Table 1, Entry 15).[15] In this case, the newly formed bond is a C-N bond leading to regioisomeric amides as compared to the isocyanate method.

Also alkylation and arylation with alkyl chlorides (Table 1, Entry 6) and aryl chloride has been reported under Co-catalysis further demonstrating the potential of Co in C-H activation chemistry.[16] The need for stoichiometric amounts of Grignard species is however a drawback regarding functional group tolerance.

Palladium catalyzed alkenylation directed by 2-pyridylmethyl ether has been reported by You and Lan (Table 1, Entry 51).[17] Coupling with acrylates, acrylamides or styrenes usually worked quite well. Due to the ether linker, the intermediate is a 7-membered palladacyle, usually less favored than 5- or 6-membered ones. Still, the yields obtained were generally good to excellent and the big advantage is the cleavability (via three different protocols as demonstrated!) of the DG leading to ortho-alkenylated phenols.

Alkylation of acyclic amines was reported initially by Jun, who showed that a methyl group in position 3 of the pyridine DG was crucial for good conversion (Table 1, Entry 32).[18] Shibata and coworkers developed later an asymmetric version of this transformation under Ir-catalysis (Table 1, Entry 3).[19, 20] Interestingly, the methyl group in position 3 was not required in this case. Remarkable *ee*s for a C-H activation reaction were obtained, always >70% and in one case even 99% ee were reported.

**Table 1: Pyridine as directing group in C-H activation chemistry with the potential for directing group cleavage**

| Entry | Directing group | Type of transformation | Coupling partner | Typical product structure | Comments | Ref |
|---|---|---|---|---|---|---|
| 1 | 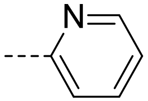 | Alkenylation | 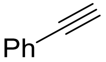 | 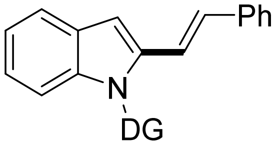 | Substrate (1 equiv), alkyne (1.5 equiv), $MnBr(CO)_5$ (10 mol%), DIPEA (20 mol%), PhCOOH (20 mol%), $Et_2O$, 80 °C, Ar, 12 h.<br>single example, 81% | 21 |
| 2 | 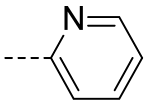 | Alkenylation | 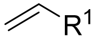 | 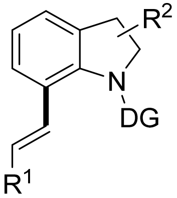 | Substrate (1 equiv), alkene (5 equiv), $[Cp^*RhCl_2]_2$ (4.5-10 mol%), $Cu(OAc)_2$ (2 equiv), DCE, 100 °C, 12-24 h.<br>$R^1$ = aryl, alkyl; $R^2$ = H, Me, Ph;<br>27 examples, 34-94%<br>In case the double bond can migrate this occurred and mixtures of E/Z isomers were obtained<br>DG cleavage: 1) MeOTf, MeCN 2) $NaBH_4$, MeOH | 14 |
| 3 | 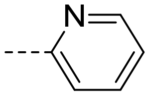 | Alkylation | 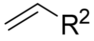 | 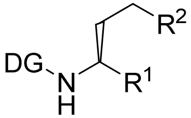 | Substrate (1 equiv), alkene (8 equiv), $[Ir(cod)_2]BF_4$ (10 mol%), (S)-tolBINAP (10 mol%), DME, 75-85 °C, 72 h;<br>$R^1$ = Me, Et, *n*Pr, *n*Pent; $R^2$ = alkyl, aryl, Bn, CH=CHPh, COOR, $SiR_3$, $CH_2SiR_3$; 18 examples, 33-87%; 72-99%ee; | 19, 20 |
| 4 | 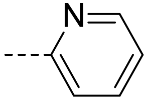 | Alkylation | 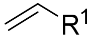 | 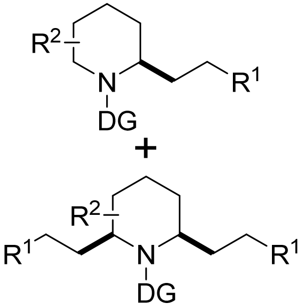 | Substrate (1 equiv), alkene (10 equiv), $Ru_3(CO)_{12}$ (4 mol%), *trans*-Cy(COOH)$_2$ (4 mol%), (*i*Pr)$_2$CHOH (5 equiv), 140 °C, 24 h.<br>$R^1$ = *n*Bu, *n*-nonyl; $R^2$ = H, Ph, COOMe, OMe, -O(CH$_2$)$_2$O-;<br>Mixtures of mono and bis-alkylation products are formed.<br>26-48% mono-product; 43-76% bis-product. | 22, 23 |

| | | | | | | |
|---|---|---|---|---|---|---|
| 5 | 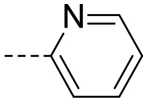 | Alkylation | 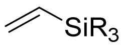  | 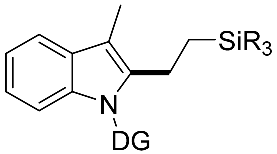  | Substrate (1 equiv), alkene (3 equiv), $[RuCl_2(p\text{-cymene})]_2$ (2.5 - 5 mol%), MesCOOK (30 mol%), toluene, 100 – 120 °C, 18 – 24 h.<br>2 examples, R = Me (95%), Et (77%). | 24 |
| 6 | 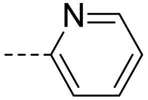 | Alkylation | Alk-Cl | 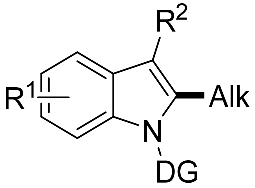  | Substrate (1 equiv), Alk-Cl (1.2 equiv), $Co(acac)_2$ (10 mol%), IPrHCl (20 mol%), CyMgCl, DMPU, 23 °C, 16 h.<br>$R^1$ = H, OMe; $R^2$ = H, Me; Alk = *n*Hex, *n*Oct, $(CH_2)_3Ph$;<br>4 examples, 67-97%. | 16 |
| 7 | 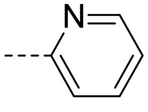 | Alkylation via aziridine opening | 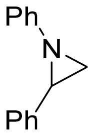  | 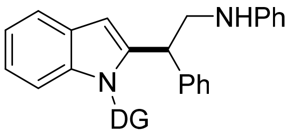  | Substrate (1 equiv), aziridine (2 equiv), $[Cp^*RhCl_2]_2$ (5 mol%), $AgSbF_6$ (30 mol%), PhCl, 100 °C, 20 h.<br>The reaction was developed for 2-arylpyridine derivatives (21 examples, 48-90%)<br>Single example on indole, 61% | 25 |
| 8 | 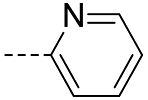 | Alkylation | 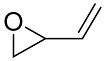  | 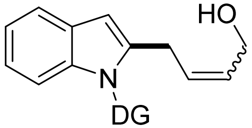  | Substrate (1 equiv), 2-vinyloxirane (1.2 equiv), PivOH (1 equiv), $[Cp^*Rh(MeCN)_3]SbF_6$ (3 mol%), Ar, 25 °C, 16 h<br>Single example, 91% (E/Z = 3.1 : 1) | 26 |
| 9 | 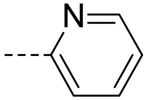 | Alkylation | 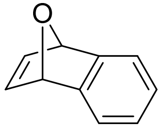  | 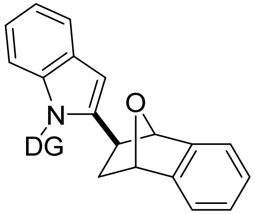  | Substrate (1 equiv), olefine (2 equiv), $[RuCl_2(p\text{-cymene})]2$ (1 mol%), O2 (1 atm), toluene, 120 °C.<br>The reaction was developed for 2-arylpyridine derivatives (14 examples, 40-94%)<br>Single example on indole, 72%. | 27 |
| 10 | 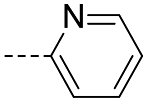 | Alkylation | $R^1$-$BF_3K$ | 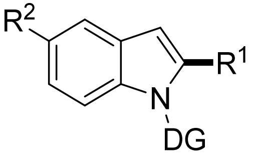  | Substrate (1 equiv), $R^1$-$BF_3K$ (3 equiv), AgF (2.8 - 4 equiv), $[Cp^*RhCl_2]_2$ (4 mol%), $AgSbF_6$ (16 mol%), DCE, 100 °C, 24 h.<br>$R^1$ = Me, *n*Bu, *n*Pent, cyclopropyl, cyclopentyl, Bn; $R^2$ = H, OMe;<br>7 examples, 51-94%. | 11 |

| | | | | | | |
|---|---|---|---|---|---|---|
| | | | | | Many other DGs successfully applied in this contribution | |
| 11 | 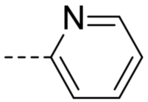 | Alkynylation | 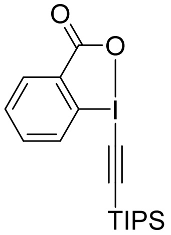 | 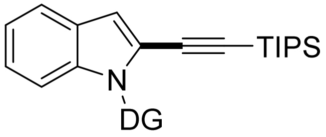 | Substrate (0.2 mmol), R-EBX (0.22 mmol),[RhCp*$Cl_2$]$_2$(2 mol%), Zn(OTf)$_2$(0.02 mmol, 10 mol%), DCE (2 mL), 25 or 80 °C, 16 h<br>Single example, 91%. | 28 |
| 12 | 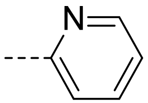 | Alkynylation | 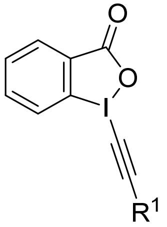 | 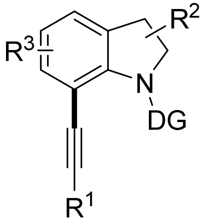 | Substrate (1 equiv), alkyne source (1.3 equiv), [Cp*Rh$Cl_2$]$_2$ (4 mol%), Cu(OTf)$_2$ (20 mol%), DCE, 50 °C, 12 h.<br>$R^1$ = TIPS, TES, TBS, TBDPS, tBu, Ph; $R^2$ = H, Me, Ph; $R^3$ = H, Me, F, Cl, Br; 19 examples, 50-91%<br>DG cleavage: 1) MeOTf, MeCN 2) $NaBH_4$, MeOH | 14 |
| 13 | 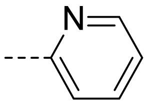 | Amidation | 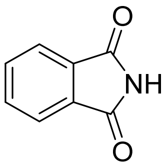 | 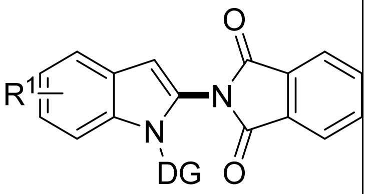 | Substrate (1 equiv), phthalimide (1.2 equiv), CuOAc (20 mol%), toluene/o-dichlorobenzene (1:1), 150 °C, $O_2$, 2-3 days.<br>$R^1$ = H, Me, MeO, CHO, CN;<br>5 examples, 31-78% | 29 |
| 14 | 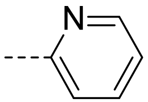 | Amidation | R-NCO | 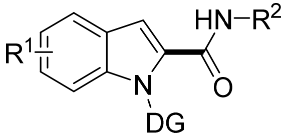 | Substrate (1 equiv), isocyanate (1.1 equiv), [MnBr(CO)$_5$] (10 mol%), $Et_2O$, 100 °C, 16 h.<br>$R^1$ = H, OMe, F, Br, I, COOMe; $R^2$ = series of aryl and alkyl residues<br>28 examples, 60-95% | 30 |
| 15 | 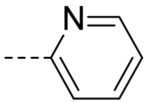 | Amidation | 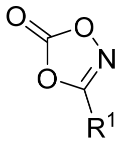 | 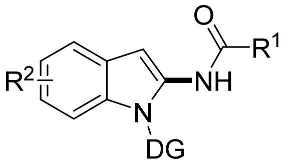 | Substrate (1 equiv), dioxazolone (1.2 equiv), Cp*Co(CO)$I_2$ (2.5 - 5 mol%), $AgSbF_6$ (5 - 10 mol%), NaOAc (5- 10 mol%), DCE, 70-100 °C, 20 h.<br>$R^1$ = Ph, 3-Me$C_6H_4$, 3-F$C_6H_4$, 3-Cl$C_6H_4$; $R^2$ = H, MeO, F, Br, I, COOMe; 13 examples, 64-98%<br>1 example with pyrrole as substrate (55%) | 15 |

| | | | | | | |
|---|---|---|---|---|---|---|
| 16 | 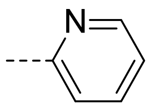 | Arylation | 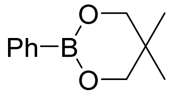 | 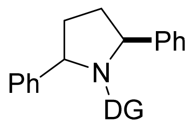 | Substrate (1 equiv), boronic acid ester (1.2 equiv), $Ru_3(CO)_{12}$ (10 mol%), *t*BuCOMe (5 equiv), 150 °C; 84% Tetrahydroquinoline was also used as substrate and gave 70% yield. | 1 |
| 17 | 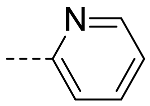 | Arylation | Ph-$B(OH)_2$ | 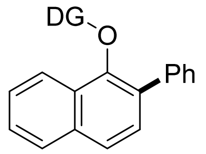 | Substrate (1 equiv), Ph-$B(OH)_2$ (2 equiv), $Pd(OAc)_2$ (5 mol%), $Cu(OTf)_2$ (1 equiv), $Ag_2O$ (1 equiv), toluene, 120 °C, 24 h;<br>Single example, 59% | 31 |
| 18 | 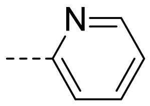 | Arylation | 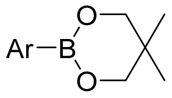 | 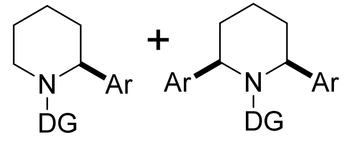 | Substrate (1 equiv), boronic acid ester (3-4 equiv), $Ru_3(CO)_{12}$ (6-8 mol%), 3-ethyl-3-pentanol (1 equiv), reflux, 24 h.<br>12 examples,<br>Mixtures of mono and bis-arylation products are formed.<br>Cleavage of DG: 1) Pd/C, $H_2$ (1 atm), HCl, *i*PrOH; 2) $NH_2NH_2.H_2O$, AcOH, *i*PrOH. | 32 |
| 19 | 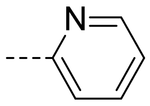 | Arylation | $ArBF_3K$ | 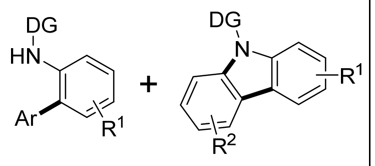 | Substrate (1 equiv), $ArBF_3K$ (4 equiv), $Pd(OAc)_2$ (10 mol%), AgOAc (3 equiv), benzoquinone (1 equiv), DMSO (4 equiv), 1,4-dioxane, 130-140 °C, 48 h.<br>$R^1$ = H, $NO_2$, MeO; $R^2$ = $NO_2$, CHO, F, Cl, Br, *t*Bu, Me, COMe;<br>14 examples, combined yield of both products 45-96%<br>Mixtures of arylation and arylation/cyclization (i.e. carbazoles) products were obtained in ratios between 48:52 – 17:83. | 33 |
| 20 | 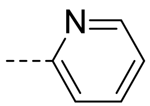 | Arylation | 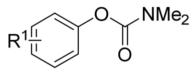 | 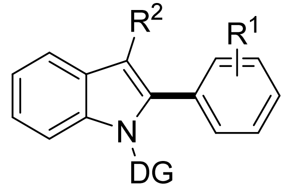 | Substrate (1.5 equiv), carbamate (1 equiv), Co(acac)2 (10 mol%), IMesHCl (20 mol%), CyMgCl (2 equiv), DMPU, 60 °C, 16 h.<br>$R^1$ = F, Me, OMe; $R^2$ = H, Me; 6 examples, 86-94% | 10 |

| | | | | | | |
|---|---|---|---|---|---|---|
| | | | | | Arylation of 2-arylpyridines was the main focus of this paper. | |
| 21 | 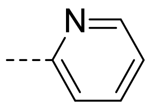  | Arylation | $ArBF_3K$ | 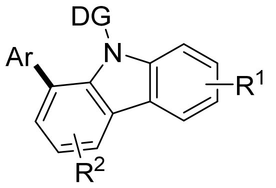  | Substrate (1 equiv), $ArBF_3K$ (2 equiv), $Pd(OAc)_2$ (10 mol%), $AgNO_3$ (3 equiv), benzoquinone (1 equiv), tBuOH, 60-70 °C, 24 h.<br>$R^1$ = H, $NO_2$, MeO; $R^2$ = H, $NO_2$, Br, MeO, *t*Bu, COMe;<br>20 examples, 45-98%.<br>DG cleavage: 1) MeOTf/DCM; 2) 2 M NaOH (aq), MeOH. | 34 |
| 22 | 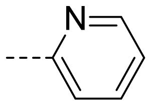  | Arylation | 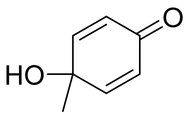  | 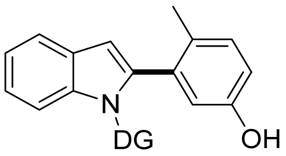  | Substrate (1 equiv), dienone (1.2 equiv), $[Cp^*RhCl_2]_2$ (5 mol%), $AgSbF_6$ (30 mol%), $Zn(NTf_2)_2$ (20 mol%), DCE, 100 °C, 20h<br>Single example, 38% | 35 |
| 23 | 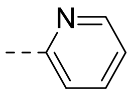  | Benzylation | 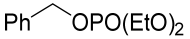  | 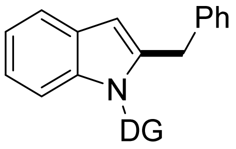  | Substrate (1.5 equiv), carbamate (1 equiv), Co(acac)2 (10 mol%), IMesHCl (20 mol%), CyMgCl (2 equiv), DMPU, 60 °C, 16 h.<br>Single example, 65%<br>Arylation of 2-arylpyridines was the main focus of this paper. | 10 |
| 24 | 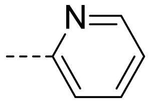  | Carbonylation | alkene, CO | 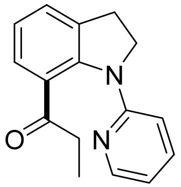  | Indoline (1 mmol), $Ru_3(CO)_{12}$ (0.05 mmol), CO (initial pressure 10 atm at 25 °C in a 5 ml stainless stell autoclave), alkene (ethylene 5 atm), *N,N*-dimethylacetamide (3 mL), 160 °C for 20 h;<br>Single example, 41% | 36 |
| 25 | 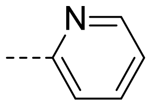  | Cyanation | *t*BuNC | 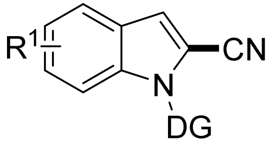  | Substrate (1 equiv), *t*BuNC (3 equiv), $Pd(OAc)_2$ (5 mol%), $Cu(TFA)_2$ (3 equiv), DMF, $O_2$, 130 °C<br>$R^1$ = H, COOMe, OMe, Br, Me;<br>5 examples, 34-85%<br>In absence of a DG cyanation takes place in position 3 of indole. | 12 |

| | | | | | | |
|---|---|---|---|---|---|---|
| 26 | 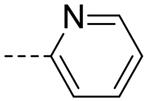 | Heteroarylation | 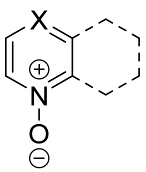 | 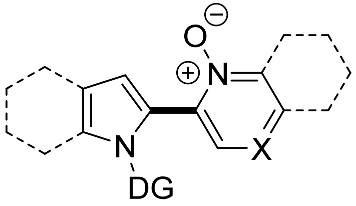 | Indole or pyrrole substrate (1 equiv), N-oxide (4 equiv), $Pd(OAc)_2$ (10-20 mol%), DPPB (10-20 mol%), $Cu(OAc)\cdot H_2O$ (3 equiv), pyridine (2 equiv), 1,4-dioxane, 140 °C, 30 h.<br>Indoles (eventually carrying Cl, Me, MeO) and pyrrole was used as substrate; N-oxides of quinoline, quinoxaline, and pyridine were used; 9 examples, 45-91% | 13 |
| 27 | 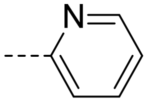 | Imine addition | 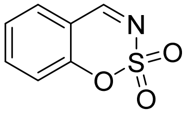 | 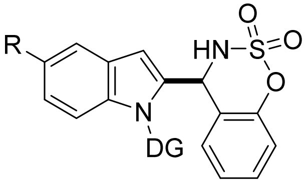 | Substrate (1 equiv), imine (1.08 equiv), $[Cp^*Rh(CH_3CN)_3]SbF_6$ (5 mol%), *t*AmylOH, 85 °C, 16 h;<br>3 examples: R = H (80%), Cl (77%), Br (70%) | 37 |
| 28 | 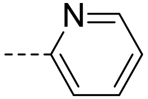 | Indole synthesis | 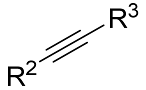 | 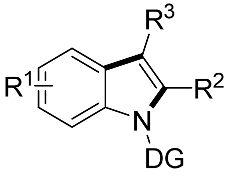 | Substrate (1.1 equiv), alkyne (1 equiv), $Pd/CeO_2$ (5 mol%, CeO 25nm), $Cu(TFA)_2\cdot H_2O$ (20 mol%), DMF, air, 120 °C, 36 h.<br>$R^1$ = H, Me, MeO, SMe, COMe, $OCF_3$, COOMe, F, Cl; $R^2$ = aryl; $R^3$ = aryl or alkyl;<br>24 examples, 8-99% (typically >90%).<br>Mixed aryl-alkyl alkynes gave predominantly the product with the alkyl in $R^3$ position. | 9 |
| 29 | 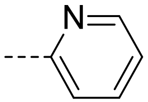 | Selenylation | PhSeSePh | 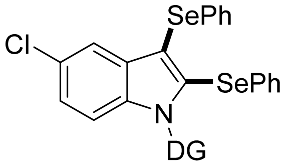 | Substrate (1 equiv), PhSeSePh (1 equiv), $Pd(OAc)_2$ (10 mol%), $CuBr_2$ (2 equiv), DMF, 80 °C, 48 h.<br>Single example, 53% | 38 |
| 30 | 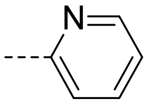 | Sulfenylation | PhSSPh | 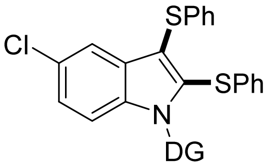 | Substrate (1 equiv), PhSSPh (1 equiv), $Pd(OAc)_2$ (10 mol%), $CuBr_2$ (2 equiv), DMF, 140 °C, 24 h.<br>Single example, 73% | 38 |

| | | | | | | |
|---|---|---|---|---|---|---|
| 31 | 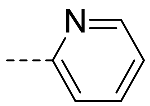 | Trifluoromethylallylation | 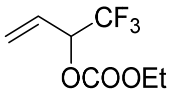 | 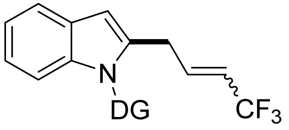 | Substrate (1 equiv), alkene (2 equiv), $[Cp^*RhCl_2]_2$ (2.5 mol%), $AgSbF_6$ (10 mol%), $Cu(OAc)_2$ (50 mol%), THF, air, 120 °C, 24 h<br>Single example 66% (16:1 E/Z) | 39 |
| 32 | 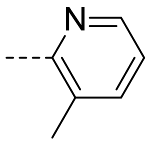 | Alkylation | 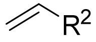 | 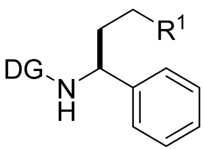 | Substrate (1 equiv), alkene (5 equiv), $Ru_3(CO)_{12}$ (10 mol%), toluene, 130 °C, 6 h.<br>$R^1$ = *n*Bu, *t*Bu, cyclohexyl, Ph, Bn $C_8H_{17}$; 8 examples, 60-95%<br>Also cyclopentene and cyclohexene were applied successfully. | 18 |
| 33 | 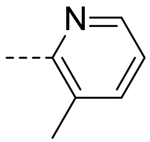 | Arylation | 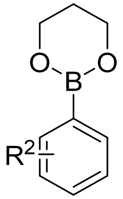 | 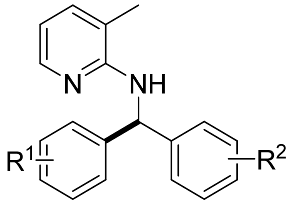 | Substrate (0.5 mmol), coupling partner (1 mmol), $Ru_3(CO)_{12}$ (5 mol%), pinacolone (0.5 mL), 140-150 °C, 24 h<br>$R^1$ = H, Me, Cl, *t*Bu, OMe, F, Cl, $CF_3$, COMe; $R^2$ = H, Me, OMe, O*i*Pr, F, $CF_3$, COOMe; 15 examples (15-76% yield), $NO_2$, CN were not tolerated<br>DG cleavage: 1) MeMgCl, $Boc_2O$, THF, rt; 2) MeOTf, DCM, 0 °C; 3) NaOH, MeOH, $H_2O$, 50 °C | 3,2 |
| 34 | 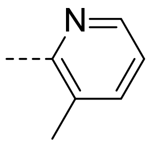 | Arylation | Ar-Br | 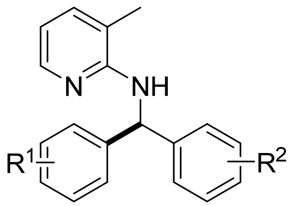 | Substrate (1 equiv), Ar-Br (1.5 equiv), $[RuCl_2(p\text{-cymene})]_2$ (2.5 mol%), KOPiv (30 mol%), $K_2CO_3$ (3 equiv), toluene, 140 °C, 24 h.<br>$R^1$ = H, Me, Cl, *t*Bu, *n*Bu, $NMe_2$, OMe, F, $CF_3$, COOEt, COMe; $R^2$ = H, Me, OMe, O*i*Pr, F, $CF_3$, COOMe<br>22 examples (28-69% yield), $NO_2$, CN were not tolerated<br>DG cleavage: 1) MeMgCl, $Boc_2O$, THF, rt; 2) MeOTf, DCM, 0 °C; 3) NaOH, MeOH, $H_2O$, 50 °C | 6, 7 |

| | | | | | | |
|---|---|---|---|---|---|---|
| 35 | 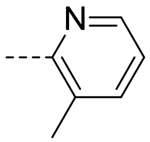 | Arylation | Ar-Cl | 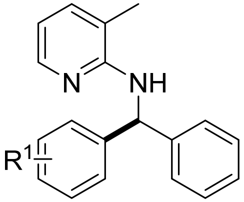 | Substrate (1 equiv), Ar-Cl (1.5 equiv), $[RuCl_2(p\text{-cymene})]_2$ (5 mol%), $PPh_3$ (10 mol%), cyclohexanol (1 equiv), $K_2CO_3$ (3 equiv), toluene, 160 °C, 30 h.<br>$R^1$ = H, Me, OMe, COOMe, F, $CF_3$;<br>7 examples (30-79% yield);<br>DG cleavage: 1) MeMgCl, $Boc_2O$, THF, rt; 2) MeOTf, DCM, 0 °C; 3) NaOH, MeOH, $H_2O$, 50 °C | 6 |
| 36 | 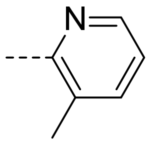 | Cyanation | *t*BuNC | 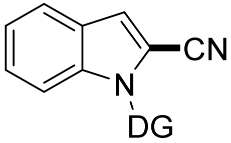 | Substrate (1 equiv), *t*BuNC (3 equiv), $Pd(OAc)_2$ (5 mol%), $Cu(TFA)_2$ (3 equiv), DMF, $O_2$, 130 °C<br>Single example, 54%<br>In absence of a DG cyanation takes place in position 3 of indole. | 12 |
| 37 | 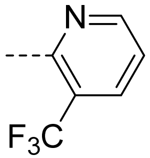 | Arylation | 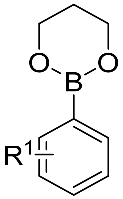 | 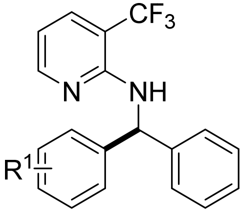 | Substrate (0.5 mmol), coupling partner (1 mmol), $Ru_3(CO)_{12}$ (5 mol%), pinacolone (0.5 mL), 140-150 °C, 24 h<br>$R^1$ = H, Me, *t*Bu, OMe, F;<br>5 examples (51-78% yield)<br>DG cleavage: 1) MeMgCl, $Boc_2O$, THF, rt; 2) MeOTf, DCM, 0 °C; 3) NaOH, MeOH, $H_2O$, 50 °C | 3,2 |
| 38 | 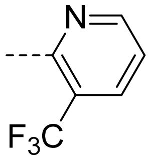 | Arylation | 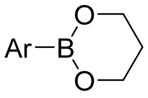 | 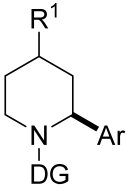 | Substrate (1 equiv), boronic acid ester (4 equiv), $Ru_3(CO)_{12}$ (7 mol%), $CuSO_4 \cdot 5H_2O$ (2 mol%), 1,3-propandiol (0.5 equiv), o-xylene, 140 °C, 24 h.<br>$R^1$ = H, Me, Bn, COOEt; Ar substituents included H, Me, *t*Bu, F, Cl, MeO, $CF_3$; 12 examples, 16-60%<br>Cleavage of DG: 1) $Pt_2O$ hydrate (5 mol%), $H_2$ (1 atm), HCl, *i*PrOH; 2) silica gel, DCM; 3) $NH_2NH_2.H_2O$, AcOH, *i*PrOH. | 5 |

| | | | | | | |
|---|---|---|---|---|---|---|
| 39 | 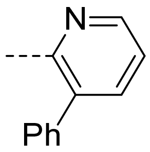 | Arylation | 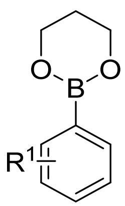 | 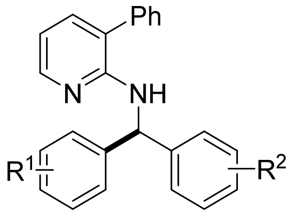 | Substrate (0.5 mmol), coupling partner (1 mmol), $Ru_3(CO)_{12}$ (5 mol%), pinacolone (0.5 mL), 140-150 °C, 24 h<br>$R^1$ = H, Me, Cl, *t*Bu, F, $CF_3$, COMe; $R^2$ = H, Me, *t*Bu, F, $CF_3$; 12 examples (33-96% yield), $NO_2$, CN were not tolerated<br>DG cleavage: 1) MeMgCl, $Boc_2O$, THF, rt; 2) MeOTf, DCM, 0 °C; 3) NaOH, MeOH, $H_2O$, 50 °C | 3,2 |
| 40 | 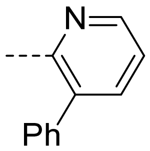 | Arylation | Ar-Br | 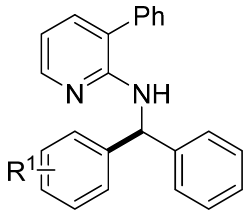 | Substrate (1 equiv), Ar-Br (1.5 equiv), $[RuCl_2(p\text{-cymene})]_2$ (2.5 mol%), KOPiv (30 mol%), $K_2CO_3$ (3 equiv), toluene, 140 °C, 24 h.<br>$R^1$ = H, Me, Cl, *t*Bu, *n*Bu, OMe, COOEt, COMe;<br>9 examples (41-72% yield), $NO_2$, CN were not tolerated<br>DG cleavage: 1) MeMgCl, $Boc_2O$, THF, rt; 2) MeOTf, DCM, 0 °C; 3) NaOH, MeOH, $H_2O$, 50 °C | 6, 7 |
| 41 | 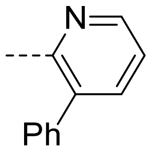 | Arylation | Ar-Cl | 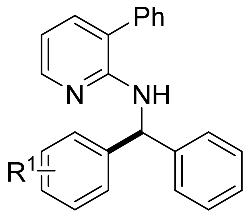 | Substrate (1 equiv), Ar-Cl (1.5 equiv), $[RuCl_2(p\text{-cymene})]_2$ (5 mol%), $PPh_3$ (10 mol%), cyclohexanol (1 equiv), $K_2CO_3$ (3 equiv), toluene, 160 °C, 30 h.<br>$R^1$ = H, Me, *t*Bu, *n*Bu, OMe; 6 examples (39-61% yield); $NO_2$, COMe, COOMe were not tolerated<br>DG cleavage: 1) MeMgCl, $Boc_2O$, THF, rt; 2) MeOTf, DCM, 0 °C; 3) NaOH, MeOH, $H_2O$, 50 °C | 6 |
| 42 | 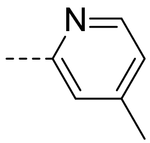 | Amidation | 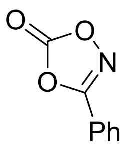 | 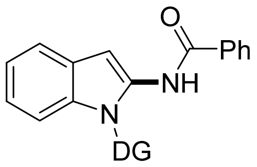 | Substrate (1 equiv), dioxazolone (1.2 equiv), $Cp^*Co(CO)I_2$ (2.5 - 5 mol%), $AgSbF_6$ (5 - 10 mol%), NaOAc (5- 10 mol%), DCE, 70-100 °C, 20 h.<br>Single example, 92% | 15 |

| | | | | | | |
|---|---|---|---|---|---|---|
| 43 | 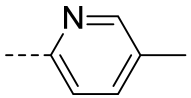 | Amidation | 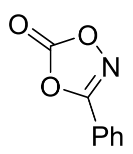 | 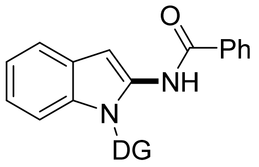 | Substrate (1 equiv), dioxazolone (1.2 equiv), Cp*Co(CO)$I_2$ (2.5 - 5 mol%), $AgSbF_6$ (5 - 10 mol%), NaOAc (5- 10 mol%), DCE, 70-100 °C, 20 h.<br>Single example, 97% | 15 |
| 44 | 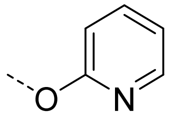 | Acylation | 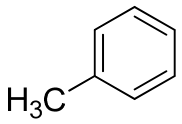 | 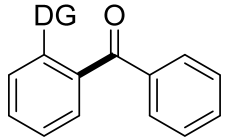 | Substrate (0.5 mmol), $Pd(OAc)_2$ (10 mol%), NHPI (20 mol%), toluene (1 mL) at 80 °C under $O_2$ (1 atm) for 24 h.<br>Single example, 43%<br>Much more comprehensive scope on other systems including 2-arylpyridines | 40 |
| 45 | 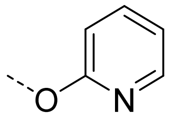 | Arylation | 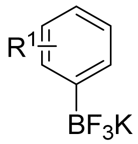 | 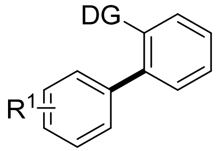 | Substrate (2-phenoxypyridine 1 mmol), $ArBF_3K$ (2.5 equiv), $Pd(OAc)_2$ (10 mmol%), $Ag_2CO_3$ (2 equiv), *p*-benzoquinone (1 equiv), DMSO (4 equiv), $H_2O$ (8 equiv), DCM, 130 – 40 °C, 48 h;<br>$R^1$ = H, $NO_2$, CHO, F, Cl, Br, I, tBu, Me, COMe, COOMe; 12 examples, 7-90%. | 41 |
| 46 | 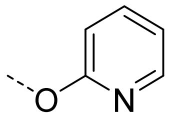 | Nitration | 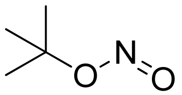 | 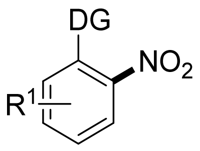 | Substrate (0.3 mmol), $Pd(OAc)_2$ (10 mol%), TBN (2.0 equiv), PhCl (1 mL), at 80 °C under $O_2$ (1 atm) for 24 h.<br>$R^1$ = H, Me, MeO, I<br>7 examples, 56-75% yield. | 40 |
| 47 | 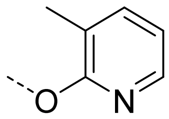 | Nitration | 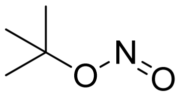 | 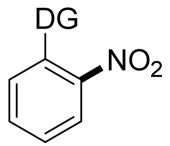 | Substrate (0.3 mmol), $Pd(OAc)_2$ (10 mol%), TBN (2.0 equiv), PhCl (1 mL), at 80 °C under $O_2$ (1 atm) for 24 h.<br>$R^1$ = H, Me, MeO, I<br>Single example, 59% | 40 |
| 48 | 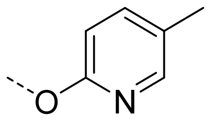 | Nitration | 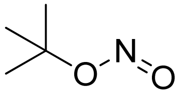 | 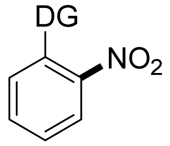 | Substrate (0.3 mmol), $Pd(OAc)_2$ (10 mol%), TBN (2.0 equiv), PhCl (1 mL), at 80 °C under $O_2$ (1 atm) for 24 h.<br>$R^1$ = H, Me, MeO, I<br>Single example, 73% | 40 |

| | | | | | | |
|---|---|---|---|---|---|---|
| 49 | 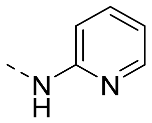 | Arylation | $ArB(OH)_2$ | 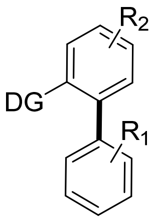 | Substrate (1 equiv), arylboronic acid (3 equiv), $Pd(OAc)_2$ (10 mol%), $Ag_2O$ (1 equiv), benzoquinone (0.5 equiv), THF, 80 °C.<br>15 examples, 43-88%; sterically demanding arlyboronic acids not well tolerated<br>DG cleavage: 1) Pd/C, $H_2$ (1 atm), HCl, *i*PrOH; 2) $NH_2NH_2.H_2O$, AcOH, EtOH. | 8 |
| 50 | 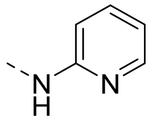 | Arylation | $ArBF_3K$ | 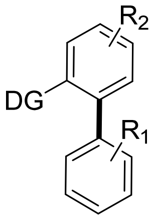 | Substrate (1 equiv), $ArBF_3K$ (1.5 equiv), $Pd(OAc)_2$ (10 mol%), $Cu(OAc)_2$ (2 equiv), benzoquinone (1 equiv), *t*BuOH, 80-90 °C, 4 h.<br>$R^1$ = H, F, Cl, Br, I, $NO_2$, MeO, CHO, COMe, Me, *t*Bu; $R^2$ = H, F, Cl, Br, $NO_2$, MeO;<br>20 examples, 45-98%.<br>DG cleavage: 1) MeOTf/DCM; 2) 2 M NaOH (aq), MeOH. | 42 |
| 51 | 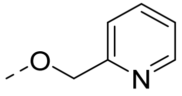 | Alkenylation | 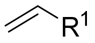 | 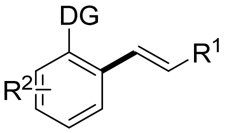 | Substrate (0.5 mmol), alkene (0.75 mmol), $Pd(OAc)_2$ (10 mol%), $KHCO_3$ (2 equiv), Boc-Val-OH (20 mol%), *t*-AmylOH, 90 °C, 1 atm $O_2$, 12 h.<br>$R^1$ = $CONMe_2$, COOalkyl, aryl; $R^2$ = Me, MeO, H, Cl, $NO_2$;<br>20 examples, 31-95%<br>DG cleavage: Pd/C, $H_2$ or Mg, MeOH, or $BBr_3$, DCM, -40 °C - rt | 17 |

## Bidentate heterocyclic directing groups in C-H activation

Due to their versatility and reliability, bidentate directing groups have been heavily used in many types of C-H functionalizations in combination with a broad spectrum of transition-metal based catalysts. Catalytic systems based on *N,N'*- as well as *N,S*-bidentate directing groups have been developed for the functionalization of C($sp^3$) as well as C($sp^2$) carbon centers. As shown by van Koten and coworkers in 1993[43], bidentate groups promote the activation of C-H bonds *via* the formation of a stable metallacycle.

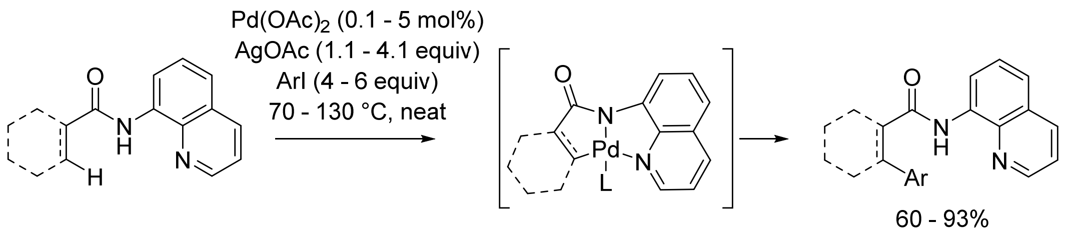


**Scheme 2: Effect of a bidentate directing group.**

The most widely used representative is without doubt the aminoquinoline auxiliary. It has been used for the first time in 2005 in a seminal study by Daugulis et al.[44](Table 2, Entry 62) together with picolinamideand since then was extended to more complex substrates and other coupling partners such as alkyl halides. It was even successfully applied in the total synthesis of celogentin C[45] or pipercyclobutanamide A[46]*via* direct C($sp^3$)-H bond activation.

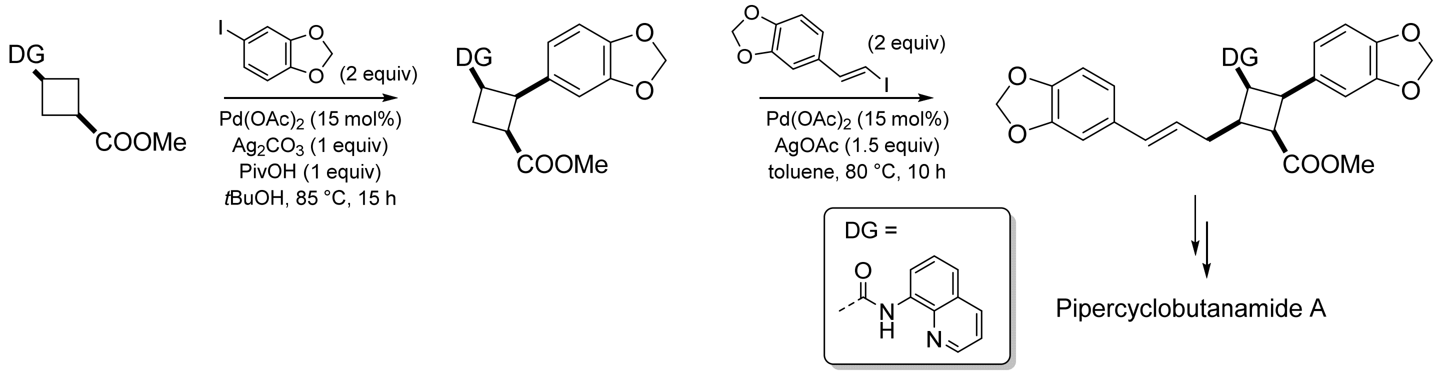


**Scheme 3: Application of bidentate directing groups in total synthesis.**

Wang et al.[47] presented a protocol for the acetoxylation of C($sp^3$)-carbon centers also at relatively complex starting materials with the potential for late stage functionalization(Table 2, Entry 27).

Significant progress has been made in the replacement of precious metals by less expensive ruthenium- nickel- or iron- based systems. In 2012, the Ru(II)-catalyzed arylation of ortho C($sp^2$)-H bonds in aromatic amides was presented(Table 2, Entry 64).[48] The presence of the bidentate aminoquinolineamide directing group was reported as crucial for the reaction to proceed also in the Ru-catalyzed alkylation of α,β-unsaturated ketones (Table 2, Entry 46).[49] The first Ni(II)-catalyzed *ortho*-alkylation of benzamides was published shortly after by the same authors(Table 2, Entry 38).[50]The double chelating aminoquinolineamide and picolinamide have been utilized by Nakamura and coworkers[51] for the C($sp^2$) and C($sp^3$) alkylation (Table 2, Entry 34). A remarkable robust setup has been presented by the Cook group (Table 2, Entry 35).[52] The *ortho*-benzylation of various aromatic or olefinic substrates was achieved on gram- scale in air.

The effects of bidentate directing groups have recently been reviewed in more detail elsewhere.[53-55]

**Table 2: Bidentate heterocyclic directing groups**

| Entry | Directing group | Type of transformation | Coupling partner | Typical product structure | Comments | Ref |
|---|---|---|---|---|---|---|
| 1 | 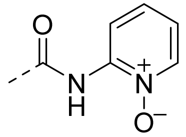 | Aryloxylation | 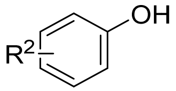 | 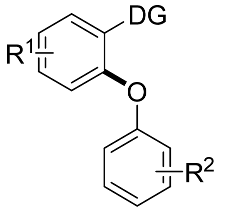 | Substrate (0.2 mmol), phenol (0.6 mmol), $Cu(OAc)_2$ (1 equiv), $Cs_2CO_3$ (1 equiv), *o*-xylene (1 mL), 130 °C, 8 h, air<br>24 examples, selective mono- or diaryloxylation possible, yields between 55 and 75%<br>$R^1$ = Me, halogen, OMe, $CF_3$ | 56 |
| 2 | | Alkoxylation | $R^1OH$ | 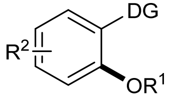 | Substrate (0.2 mmol), $R^1OH$ (0.75 mL), CuCl (0.2 mmol), $K_2CO_3$ (0.1 mmol), pyridine (0.75 mmol), 130 °C, air, 12 h<br>40 examples, 38-94% yield<br>$R^1$ = Alkyl<br>$R^2$ = OMe, Me, $CF_3$, COOMe, $SO_2Me$, $NO_2$, halogen | 57 |
| 3 | | Alkoxylation | $R^1OH$ | 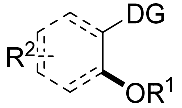 | Substrate (0.2 mmol), $R^1OH$ (1.5 mL), $Co(OAc)_2 \cdot H_2O$ (20 mol%), $Ag_2O$ (0.2 mmol), $NaOPiv \cdot H_2O$ (2 equiv), argon, 40 °C, 12 h<br>33 Examples, 34-83% yield<br>$R^1$ = Alkyl, Bn<br>$R^2$ = OMe, Me, $CF_3$, $NMe_2$, halogen | 58 |
| 4 | 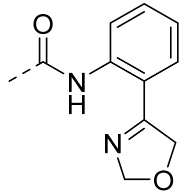 | Alkylation / Cyclization | Malonate | 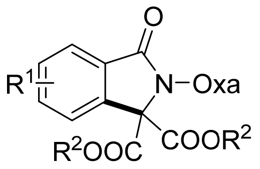 | Substrate (0.1 mmol), malonate (0.2 mmol), $Cu(OAc)_2$ (20 mol%), $Li_2CO_3$ (0.1 mmol), $Ag_2CO_3$ (0.15 mmol), DMSO (4 mL), air, 80 °C, 12 h<br>20 examples, 40-72% yield<br>$R^1$ = Halogen, Me, OMe, $^t$Bu, Ac, $CF_3$<br>$R^2$ =  Me, Et | 59 |

| | | | | | | |
|---|---|---|---|---|---|---|
| 5 | | Amidation | $R^2SO_2NH_2$ /<br>$R^2CONH_2$ | 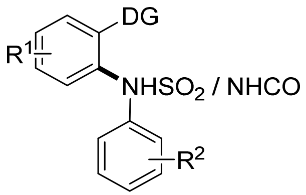 | Substrate (0.1 mmol), sulfonylamide / amide (0.2 mmol), $Cu(OAc)_2$ (0.1 mmol), $Na_2CO_3$ (0.2 mmol), DMSO (1 mL), 80 °C, air, 6 h<br>38 Examples, 9-85% yield<br>$R^1$ = Aryl, vinyl, halogen, Me, OMe<br>$R^2$ = Me, OMe, halogen, $NO_2$, COOMe | 60 |
| 6 | | Arylation | ArBPin | 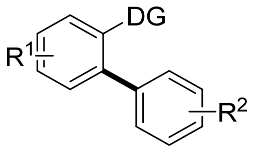 | Substrate (1 mmol), arylboronate (0.25 mmol), $Cu(OAc)_2$ (0.03 mmol), $Ag_2O$ (0.15 mmol), $Na_2CO_3$ (0.2 mmol), KOAc (0.2 mmol), DMSO (1 mL), 70 °C, 4 h<br>28 Examples, 26-70% yield<br>$R^1$ = Aryl, vinyl, halogen, Me, OMe<br>$R^2$ = Me, OMe, halogen, $NO_2$, COOMe | 61 |
| 7 | 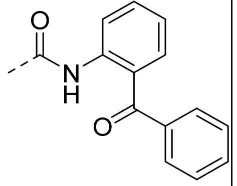 | Acetoxylation/<br>Methoxylation | $Ac_2O$ / MeOH | 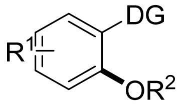 | Acetoxylation*:* Substrate (1 mmol), $PhI(OAc)_2$ (1 mmol), $Pd(OAc)_2$ (0.1 mmol), AcOH (0.5 mL), $Ac_2O$ (0.5 mL), 110 °C, $N_2$<br>Methoxylation*:* Substrate (1 mmol), $PhI(OAc)_2$ (1 mmol), $Pd(OAc)_2$ (0.1 mmol), MeOH (0.5 mL), 110 °C, $N_2$<br>14 examples, 30-85% yield<br>$R^1$ = Me, OMe, OPh, $NO_2$, halogen<br>$R^2$ = Ac, Me<br>- *o*-$NO_2$ is not tolerated | 62 |
| 8 | | Arylation | ArI | 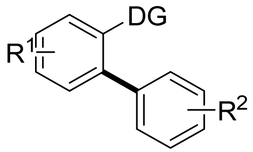 | Substrate (1 mmol), iodoarene (3 mmol), AgOAc (1 mmol), $Pd(OAc)_2$ (0.1 mmol), 110 °C<br>14 Examples, 65-90% yield<br>$R^1$ = Me, OMe, $NO_2$, F<br>$R^2$ = Me, OMe, C(O)Me, | 63 |

| | | | | | | |
|---|---|---|---|---|---|---|
| 9 | 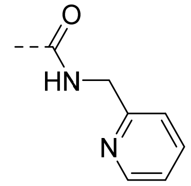 | Arylation | ArBr | 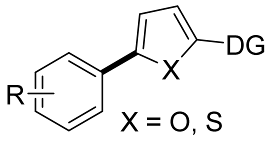 | Substrate (1.5 equiv), aryl bromide (1 equiv), $PdCl(C_3H_5)$(dppb) (0.5 mol%), KOAc (2 equiv), DMAc, 150 °C, 16 h<br>16 examples, also *N*-*n*-propylamide possible, no electron rich coupling partners investigated<br>R = C(O)Me, CHO, CN, $CF_3$, F; pyridine tolerated<br>- Authors state under these reaction conditions, amide does not function as directing group | 64 |
| 10 | | Carbonylation | CO, ethylene | 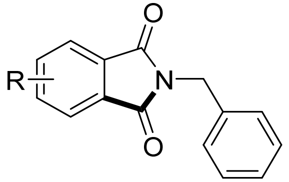 | Substrate (1 equiv), $Ru_3(CO)_{12}$ (5 mol%), CO (10 atm), ethylene (7 atm), $H_2O$ (2 equiv), toluene, 160 °C, 24 h<br>16 Examples, 60-89% yield<br>R = Me, OMe, $NMe_2$, COOMe, C(O)Me, CN, Cl, Br<br>open chain product not observed | 65 |
| 11 | | Carbonylation | CO | 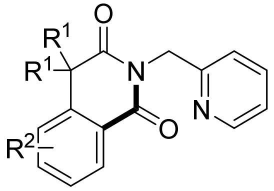 | Substrate (1 mmol), CO (10 atm), ethylene (7 atm), $H_2O$ (2 mmol), $Ru(CO)_{12}$ (0.05 mmol), toluene (3 mL), 160 °C, 24 h<br>9 examples, 41-93% yield | 66 |
| 12 | 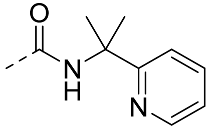 | Arylation | ArI | 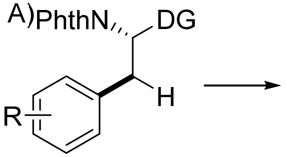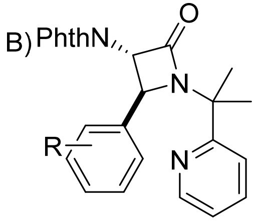 | A)<br>Substrate (0.2 mmol), iodoarene ((0.3 mmol), $Pd(OAc)_2$ (10 mol%), $CuF_2$ (0.3 mmol), DMPU (1 mmol), acetone (2 mL), $N_2$, 100 °C, 24 h<br>17 Examples, 35-89% yield<br>R = Me, OR, Halogen, NHAc, $NO_2$<br>B) –<br>A (0.15 mmol), $Pd(OAc)_2$ (10 mol%), $NaIO_3$ (0.3 mmol), $Ac_2O$ (1.5 mmol), $CH_3CN$ (3 mL), $N_2$, 70 °C, 48 h<br>17 Examples, 46-85% yield | 67 |

| | | | | | | |
|---|---|---|---|---|---|---|
| | | | | | - diastereoselective | |
| 13 | | Arylation | ArBr | 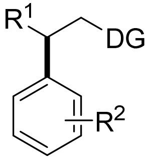 | Substrate (0.15 mmol), bromoarene (0.15 mmol), $Pd(OAc)_2$ (10 mol%), $K_2CO_3$ (2.5 equiv), PivOH (0.2 equiv), *t*-BuOH (1.5 mL), 120 °C, 24 h<br>40 Examples, 23-89% yield<br>$R^1$ = Alkyl, Aryl<br>$R^2$ = Me, Halogen, $CF_3$, CN,<br>COOR, $NO_2$, NHAc, OR | 68 |
| 14 | | Hydroxylation | $Cu(OAc)_2$ | 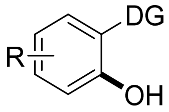 | Substrate (0.2 mmol), $Cu(OAc)_2$ (0.2 mmol), $Ag_2CO_3$ (0.4 mmol), tetrabutylammonium iodide (0.4 mmol), DMF (2 mL), 100 °C, 1 h<br>15 Examples, 31-93% yield<br>R = OMe, Me, $CF_3$, halogen, $NO_2$, NHAc | 69 |
| 15 | | Arylation | ArBr | 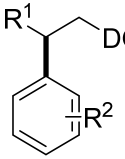 | Amide (0.15 mmol), $Pd(OAc)_2$ (0.1 mol%), $K_2CO_3$ (2.5 equiv), PivOH (0.2 equiv), *t*-BuOH (1.5 mL), 120 °C, 24 h<br>40 Examples, 27-89% yield<br>$R^1$ = Aryl, heteroaryl, alkyl<br>$R^2$ = Me, F, OMe,CN, $CF_3$, COOR, $NO_2$, NHAc | 68 |
| 16 | | Arylation/Amidation | ArI | 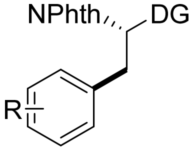 | Amide (0.2 mmol), aryl iodide (1.5 equiv), $Pd(OAc)_2$ (10 mol%), $CuF_2$ (0.3 mmol), DMPU (1 mmol), acetone (2 mL), $N_2$, 100 °C, 24 h<br>21 Examples, 35-82% yield<br>R = Me, $^t$Bu, halogen, OMe, NHAc | 67 |
| 17 | | Alkoxylation | ROH | 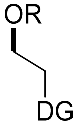 | Amide (0.2 mmol, $Pd(OAc)_2$ (10 mol%), $PhI(OAc)_2$ (1.5 equiv), alcohol/*m*-xylene (1/1, 2 mL), 90 °C, 24 h; 14 – 90% yield | 70 |

| | | | | | | |
|---|---|---|---|---|---|---|
| 18 | | Carbonylation | CO | 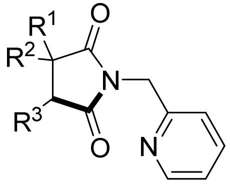 | Substrate (1 mmol), CO (10 atm), ethylene (7 atm), $H_2O$ (2 mmol), $Ru_3(CO)_{12}$ (0.05 mmol), toluene (3 mL), 160 °C, 5 days<br>15 Examples; Yield: 52-83%<br>$R^1$ = Alkyl, Bn<br>$R^2$ = Alkyl<br>$R^1$-$R^3$ = -($CH_2$)-<br>$R^1$-$R^2$ = -$(CH_2)_5$- | 71 |
| 19 | 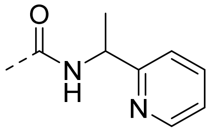 | Carbonylation | CO | 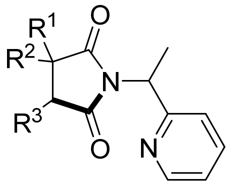 | Substrate (1 mmol), CO (10 atm), ethylene (7 atm), $H_2O$ (2 mmol), $Ru_3(CO)_{12}$ (0.05 mmol), toluene (3 mL), 160 °C, 5 days<br>6 Examples; Yield: 14-81%<br>$R^1$ = Alkyl, Bn<br>$R^2$ = Alkyl<br>$R^1$-$R^3$ = -$(CH_2)_{1\text{-}2}$- | 71 |
| 20 | 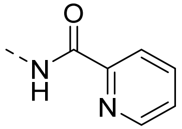 | Alkylation | RI | 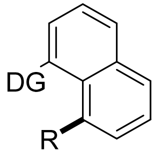 | Substrate (0.25 mmol), $Pd(OAc)_2$ (15 mol%), KOAc (2 equiv), 1,4-dioxane or xylene (5 mL), 130 °C<br>11 examples, 15-82% yield<br>R = Alkyl, Bn | 72 |
| 21 | | Alkylation, Arylation | $R^3X$<br>(X=Br, I) | 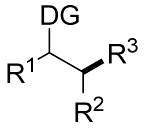 | Substrate (0.74 mmol), $Pd(OAc)_2$ (5 mol%), $K_2CO_3$ (2.5 equiv), pivalic acid (2 equiv), alkyl bromide or iodide (4 equiv), $^t$Amyl-OH solvent, 24 h, 110 °C.<br>15 Examples; Yield: 29-91%<br>$R^1$- $R^2$ = Alkyl<br>$R^2$ = Aryl<br>$R^3$ = Aryl, Alkyl | 73 |

| | | | | | | |
|---|---|---|---|---|---|---|
| 22 | | Intramolecular Amination | | 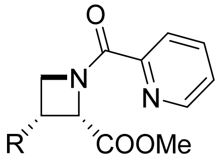 | Substrate (1 equiv), $Pd(OAc)_2$ (5 mol%), $PhI(OAc)_2$ (2.5 equiv), AcOH (2 equiv), toluene, Ar, 110 °C, 24 h<br>6 Examples, 25-91%,<br>R = Alkyl, $O^tBu$ | 74 |
| 23 | | Arylation | ArI | 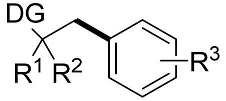 | Substrate (0.1 mmol), iodoarene (0.2 mmol), $Pd(OTFA)_2$ (10 mol%), $Ag_3PO_4$ (0.09 mmol), TBB (1 mL), 100 °C, 3 h<br>14 examples, 40-84% yield<br>$R^1$ = Alkyl<br>$R^2$ = Alkyl<br>$R^3$ = Alkyl, OR, Halogen, OAc<br>- ortho-substitution not tolerated | 75 |
| 24 | | Arylation | ArI | 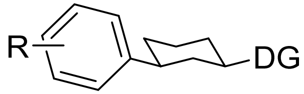 | Substrate (1 equiv), aryliodide (1.5 equiv), $Pd(OAc)_2$ (0.1 equiv), $Ag_2CO_3$ (1 equiv), *t*BuOH, 80 °C, 24 h<br>6 Examples, 31-81% yield<br>R = OMe, COOMe, $NO_2$, OTIPS<br>also alkeneiodides possible (110 °C required)<br>- Stereoselective formation of arylated cyclohexane derivatives | 76 |
| 25 | | Intramolecular Cyclization | | 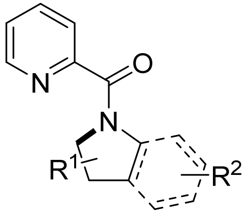 | Substrate (1 equiv), $Pd(OAc)_2$ (5 mol%), $PhI(OAc)_2$ (2 equiv), 80-120 °C, toluene, 24 h<br>14 Examples, 16-86% yield<br>$R^1$ = Alkyl<br>$R^2$ = Cl, OMe<br>Formation of pyrrolidine, indoline and isoindoline possible, | 77 |
| 26 | | Halogenation | $NaXO_3$ | 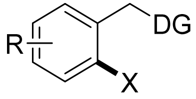 | Iodination: Substrate (1 equiv), $KIO_3$ (2 equiv), $K_2S_2O_8$ (2 equiv), $Pd(OAc)_2$ (10 mol%), n-BuOH, 120 °C, 24 h<br>6 Examples, 43-73% yield | 78 |

| | | | | | | |
|---|---|---|---|---|---|---|
| | | | | | Bromination/Chlorination: Substrate(1 equiv), NaX (1.5 equiv), $NaXO_3$ (1.5 equiv), $K_2S_2O_8$ (2 equiv), $Pd(OAc)_2$ (10 mol%), n-BuOH, 100-110 °C, 24 h<br>12 Examples, 20-65% yield | |
| 27 | 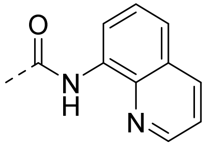 | Acetoxylation | AgOAc | 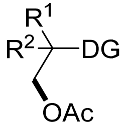 | Substrate (1 equiv), $Cu(OAc)_2$ (1 equiv), AgOAc (5 equiv), NaOAc (1 equiv), NMP, 145 °C, 24h.<br>19 Examples, Yield 39-87%; up to 38% bis-acetoxylation<br>$R^1$ = Alkyl, $CF_3$, aryl<br>$R^2$ = Alkyl | 47 |
| 28 | | Alkenylation / Cyclization | 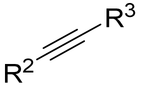 | 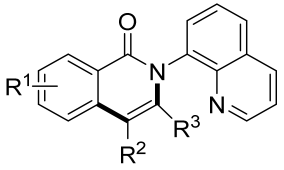 | Substrate (0.4 mmol), alkyne (0.8 mmol), $[RuCl_2(p\text{-}cymene)_2]$ (5 mol%), $Cu(OAc)_2{\cdot}H_2O$, *t*AmOH, 110 °C, 24h.<br>$R^1$ = OMe, Halogen, $NO_2$, CN, Me, $CF_3$, *t*Bu<br>$R^2$ = Aryl, Alkyl<br>$R^3$ = Aryl, Alkyl | 79 |
| 29 | | Alkenylation / Cyclization | 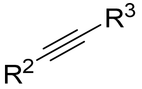 | 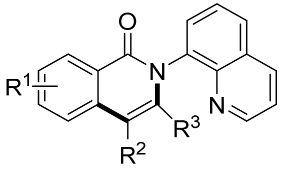 | Substrate (0.5 mmol), alkyne (1.2 equiv), $Co(OAc)_2{\cdot}4H_2O$ (10 mol%), NaOPiv (2 equiv), $Mn(OAc)_2$ (1 equiv), $CF_3CH_2OH$ (5 mL), 80 °C, 16 h<br>18 Examples; Yield: 64-96%<br>$R^1$ = $CF_3$, I, Br, $NO_2$, Me, OMe; thiophene and furan tolerated<br>$R^2$ = Ph, $CH_2OH$, Me, H<br>$R^3$ = $CH_2OH$, alkyl, Ph, COOEt, TIPS, $CH_2NPhth$ | 80 |
| 30 | | Alkenylation / Intramolecular Cyclization | 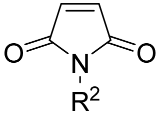 | 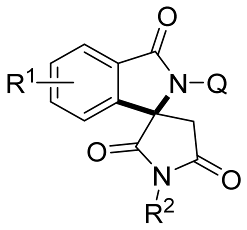 | Substrate (0.25 mmol), maleimide (1.0 mmol), $Cu(OAc)_2$ (1 mmol), PivOH (0.25 mmol), $Cy_2NMe$ (1 mmol), DMF (1.5 mL), 80 °C, 24 h, $N_2$.<br>16 examples, 19-99 % yield<br>$R^1$ = Me, OMe, Cl, $CF_3$ | 81 |

| | | | | | | |
|---|---|---|---|---|---|---|
| | | | | | $R^2$ = Me, Bn, Ph | |
| 31 | | Alkenylation | 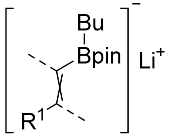 | 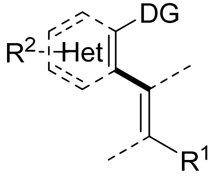 | Substrate (0.4 mmol), borate (4 equiv), $Fe(acac)_3$ (10 mol%), dppen (10 mol%), $ZnBr_2 \cdot TMEDA$ (20 mol%), DCIB (200 mol%), THF, 70 °C, 24 h<br>41 Examples; Yield: 51-96%<br>$R^1$ = Alkyl, aryl,<br>$R^2$ = Alkene, aryl, heteroaryl | 82 |
| 32 | | Alkenylation | 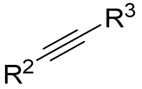 | 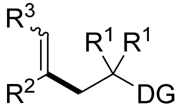 | Substrate (0.6 mmol), alkyne (0.2 mmol), $Ni(OAc)_2$ (30 mol%), $PPh_3$ (60 mol%), *i*PrOH (0.1 mL), toluene (0.5 mL), 170 °C, 24 h<br>18 Examples; Yield: 64-84%<br>$R^1$ = Me, $-(CH_2)_{4-5}-$<br>$R^2$ = Aryl, heteroaryl, alkyl<br>$R^3$ = Aryl, heteroaryl | 83 |
| 33 | | Alkenylation, Alkynylation, Arylation | RI | 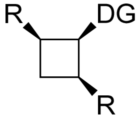 | Substrate (1 equiv), RI (3 equiv), AgOAc (3 equiv), $Pd(OAc)_2$ (5-40 mol%), toluene, 80-100 °C<br>6 Examples; Yield: 77-98%<br>R = Aryl, alkene, alkyne<br>applicable to to synthesis of piperarborenines | 84 |
| 34 | | Alkylation | $AlR^2_3$ | 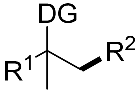 | Substrate (0.5 mmol), $AlR^2_3$ (2 equiv, 2M sol. in hexane), $Fe(acac)_3$ (10 mol-%), Ph-dppen (11 mol-%), 2,3-DCB (4 equiv), THF, 70 °C, 24h.<br>R1 – R2 = Alkyl | 51 |
| 35 | | Alkylation | $R^2X$ | 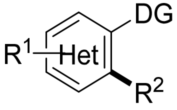 | Benzylation: Substrate (1 equiv), benzylchloride (3-3.5 equiv), ($Fe(acac)_3$ (10-15 mol%), dppe (15-20 mol%), PhMgBr (3.25-4.1 equiv), THF, 65 °C, 8-10 min, under air or $N_2$. | 52 |

| | | | | | | |
|---|---|---|---|---|---|---|
| | | | | | 16 Examples; Yield: 41-91%<br>$R^1$ = Alkyl, halogen, $CF_3$, SMe, $NMe_2$, OMe; thiophene and pyrrole tolerated<br>Alkylation: $Fe(acac)_3$ (10 mol%), dppe (11 mol%), $R^2X$ (secondary) (2.0 equiv), PhMgBr (4.1 equiv), BHT in THF, 65 °C, 5 min, under air or $N_2$.<br>10 Examples; 26-73%<br>$R^2$ = Secondary alkyl | |
| 36 | | Alkylation | $R^4X$<br>(X= Br, I, OTs) | 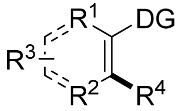 | Substrate (0.4 mmol), *p*-AnisMgBr (3 equiv), $ZnBr_2$·TMEDA (2 equiv), alkyl bromide or iodide (1.2−1.5 equiv), $Fe(acac)_3$ (10 mol%), dppen (10 mol%), NaI (1.5 equiv), THF, 50−70 °C, 9−12 h<br>33 Examples; Yield: 12-93%<br>$R^1$ –$R^2$= Aryl<br>$R^1$ – $R^2$ = Alkyl<br>$R^3$ = Me, OMe, NMe2, halogen; thiophene and indole tolerated<br>$R^4$ = Alkyl | 85 |
| 37 | | Alkylation | $R^2MgCl$ | 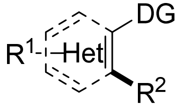 | Substrate (1 equiv), RMgCl (4 equiv), $ZnCl_2$·TMEDA (3 equiv), $Fe(acac)_3$/dppen (10 mol%), DCIB (2 equiv), THF, 70 °C, 15 h.<br>27 Examples; Yield. 51-99%<br>$R^1$ = Me, OMe, halogen, $NMe_2$; furan, thiophene, indole tolerated<br>$R^2$ = Alkyl | 86 |

| | | | | | | | |
|---|---|---|---|---|---|---|---|
| 38 | | | Alkylation | $R^2Br$ | 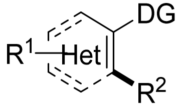 | Substrate (0.3 mmol), RBr (0.6 mmol), $Ni(OTf)_2$ (10 mol%), $PPh_3$ (20 mol%), and $Na_2CO_3$ (2 equiv), toluene (1 mL), 140 °C, 24 h<br>33 Examples; Yield: 50-95%<br>$R^1$ = OR, Me, Ph, C(O)Me, $CF_3$, halogen; heterocyclic and olefinic substrates tolerated<br>$R^2$ = Alkyl | 50 |
| 39 | | | Alkylation | ArI | 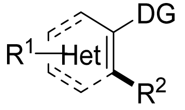 | Substrate (0.3 mmol), ArI (0.6 mmol),$Ni(OTf)_2$ (5-10 mol%), $NaHCO_3$(0.6 mmol) in toluene (1 mL) at160 °C for 20 h<br>$R^1$ = F, Ph, $CF_3$, $NMe_2$, OMe, Me, halogen, C(O)Me; thiophene tolerated | 87 |
| 40 | | | Alkylation | $R^3X$<br>X = I or Br | 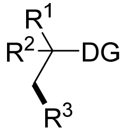 | Substrate (0.3 mmol), RX (5.0 equiv),$Ni(acac)_2$(10 mol%), dppbz (10 mol%), $Cs_2CO_3$ (5.0 equiv), $N_2$(1 atm), 1.2 mL toluene, 150°C, 12−24 h.<br>26 Examples; Yield: 61-91%<br>$R^1$ = Alkyl, benzyl, Ph<br>$R^2$ = Alkyl<br>$R^3$ = Alkyl | 42 |
| 41 | | | Alkylation | $R^2X$<br>(X=Br, I) | 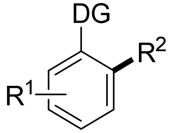 | Substrate (0.74 mmol), $Pd(OAc)_2$ (5 mol%), $K_2CO_3$ (2.5 equiv), pivalic acid (20 mol%), alkyl bromide or iodide (3−4 equiv), $^t$Amyl-OH, 12−96 h, 100−110 °C.<br>16 Examples; Yield: 22-94%<br>$R^1$ = Br, *t*Bu, $CF_3$, OMe<br>$R^2$ = Aryl, alkyl<br>mostly bisarylation | 73 |

| | | | | | | |
|---|---|---|---|---|---|---|
| 42 | | Alkylation | Alkyl iodide | 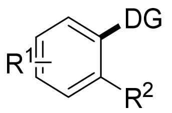 | Monoarylation: Substrate (0.2 mmol), alkyl iodide (3 equiv), $NaHCO_3$ (2 equiv), BP (0.3 equiv), $Pd(OAc)_2$ (5 mol%), 110 °C , 20 h, Ar<br>8 Examples; Yield: 75-87%<br>$R^1$ = OMe, Me, $CF_3$<br>$R^2$ = Alkyl, benzyl<br>Diarylation: Substrate (0.2 mmol), alkyl iodide (4 equiv), $NaHCO_3$ (3.5 equiv), BP (0.3 equiv), $Pd(OAc)_2$ (5 mol%), $O_2$, 110 °C , 20 h<br>4 Examples; Yield: 82%<br>Also bromides and chlorides applicable | 88 |
| 43 | | Alkylation, Arylation | $R^1I$ | 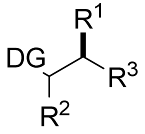 | Substrate (1 equiv), Ri (3-4 equiv), $Pd(OAc)_2$ (0.05 equiv), base (2.5-3.5 equiv), *t*-AmOH<br>13 Examples; Yield: 45-81%<br>$R^1$ = Alkyl, aryl<br>$R^2$ – $R^3$ = Alkyl, aryl<br>bisarylation was observed | 89 |
| 44 | | Alkylation | 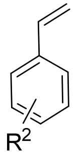 | 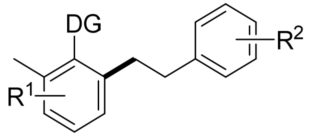 | Substrate (0.3 mmol), styrene (2 equiv), $[Rh(OAc)(cod)]_2$ (2.5 mol%), PivOH (1 equiv), toluene (1 mL), 160 °C, 12 h<br>19 Examples; Yield: 39-90%<br>$R^1$ = OMe, Me, F, OAc, $CF_3$; thiophene tolerated<br>$R^2$ = OMe, alkyl, Ph<br>Heck type reaction is suppressed by addition of PivOH. | 90 |
| 45 | | Alkylation | 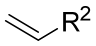 | 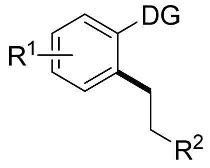 | Substrate (0.3 mmol), alkene (0.6 mmol), $[RhCl(cod)]_2$ (2.5 mol%), KOAc (25 mol%), toluene (1 mL), 160 °C, 12 h<br>24 Examples; Yield: 48-91%<br>$R^1$ = Me, OMe, Ph, $CF_3$, F, Ac, Br; heterocycles tolerated | 91 |

| | | | | | | |
|---|---|---|---|---|---|---|
| | | | | | $R^2$ = COOR, $SO_2Ph$ | |
| 46 | | Alkylation | 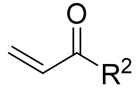 | 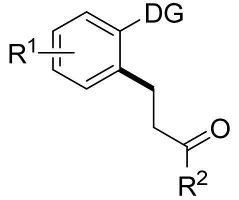 | Substrate (0.5 mmol), vinyl ketone (2 equiv), $[RuCl_2(p\text{-}cymene)]_2$ (10 mol%), NaOAc (25 mol%), toluene (1 mL), 100 °C, 4-6 h.<br>43 Examples; Yield: 16-96%<br>$R^1$ = Me, Ph, $CF_3$, OMe, F, COOMe, halogen, $NMe_2$, $OCF_3$, OAc; heterocycles tolerated<br>$R^2$ = Alkyl, aryl<br>Bisarylation up to 70% | 49 |
| 47 | | Alkylation | alkyl halide | 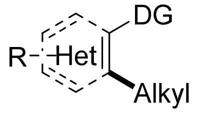 | Substrate (1 equiv), alkyl halide (3 equiv), PhMgBr (3.45 equiv), $[Fe(acac)_3]$ (10 mol%), dppe (15 mol%), 2-Me-THF (1 M), 65 °C, 9 min<br>33 Examples; Yield: 31-90%<br>R = Alkyl, halogen, $CF_3$, OMe, $NMe_2$,; pyrrole tolerated | 92 |
| 48 | | Alkynylation | 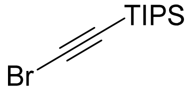 | 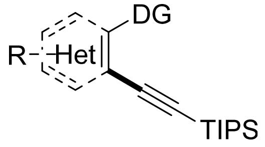 | Substrate (0.2 mmol), alkyne (1.2 equiv), $NiCL_2$ (10 mol%), BDMAE (40 mol%), $Na_2CO_3$ (5 equiv), toluene (2 mL), 100 °C, 24 h<br>25 Examples, 33-95% yield<br>R = Halogen, $NO_2$, OMe, $CF_3$, Me, Ac | 93 |
| 49 | | Alkynylation | 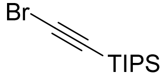 | 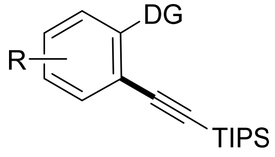 | Substrate (0.3 mmol),alkyne (1.2 equiv), $Pd(OAc)_2$(5 mol%), CsOAc (1 equiv), toluene (0.6 mL), 110 °C,15 h<br>14 Examples; Yield: 66-92%<br>R = Me, OR, $NMe_2$, Br, $CF_3$, COOMe; thiophene tolerated | 94 |
| 50 | | Alkynylation | 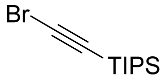 | 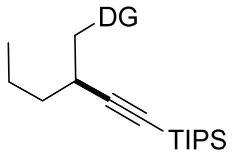 | Substrate (0.21 mmol), alkyne (1.5 equiv), SAuPd, AgOAc (1.2 equiv), LiCl (2 equiv), 135 °C<br>Yields: 80%<br>Pd immobilized on SAuPd, recyclable for up to 10 times | 95 |

| | | | | | | |
|---|---|---|---|---|---|---|
| 51 | | Allylation | 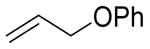 | 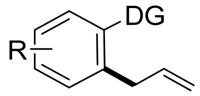 | Substrate (0.4 mmol), allyl phenyl ether (1.2 equiv), $Fe(acac)_3$ (5 mol%), dppen (5 mol%), $ZnCl_2$·TMEDA (1.2 equiv), $^{t}BuCH_2MgBr$ (3.4 equiv), 4 h, 70 °C<br>15 Examples; Yield: 61-97%<br>R = Me, OMe, halogen, $CF_3$, COOMe; heterocycles and polyaromatic compounds tolerated | [96] |
| 52 | | Amidation / Intramolecular Cyclization | - | 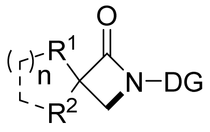 | Substrate (0.3 mmol), $[Ni(dme)_2I_2]$ (10 mol%), TEMPO (3 equiv), $K_2HPO_4$ (2 equiv), TBAI (0.1 equiv), *n*PrCN/PhCN (1.5 mL, 3:2, v/v), 150 °C, 24 h.<br>25 examples, 11-93% yield<br>$R^1$ = Alkyl, Ph, Bn, CH2OAc<br>$R^2$ = Alkyl<br>n = 2-4 | [97] |
| 53 | | Amidation (Intramolecular) | | 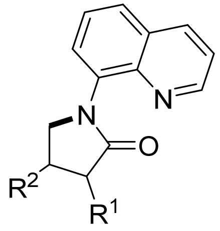 | Substrate (0.2 mmol), $Pd(OAc)_2$ (5 mol%), $PhI(OAc)_2$ (2.5 equiv), toluene, 70-110 °C, Ar, 24 h.<br>7 Examples, Yield: 65-94%<br>$R^1$ = Me, $^{t}$BuO<br>$R^2$ = NHPhth, H | [98] |
| 54 | | Amidation (Intramolecular Cyclization) | - | 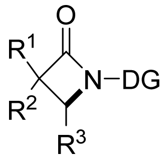 | Substrate (1 equiv), $Cu(OAc)_2$ (20 mol%), $Ag_2CO_3$ (3.0 equiv), DCE, 140 °C, 24h.<br>18 examples, 51-93% yield<br>$R^1$ = Alkyl, Ph, Bn, $CF_3$<br>$R^2$ = Me, Et<br>$R^3$ = Aryl | [99] |
| 55 | | Amination | 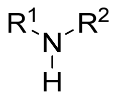 | 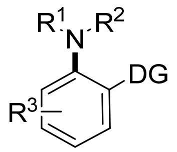 | Substrate (0.2 mmol), amine (0.4 mmol), $Ni(OAc)_2$ (10 mol%), $Ag_2CO_3$ (0.4 mmol), $Na_2CO_3$ (0.4 mmol), toluene (2.0 mL), 140 °C, air, 10 h. | [100] |

| | | | | | | |
|---|---|---|---|---|---|---|
| | | | | | 31 Examples; Yield: 44-80%<br>$R^1$ – $R^2$ = Morpholine, Alkyl<br>$R^3$ = Alkyl, OMe, Ph, $NMe_2$, F, Cl, Br, $CF_3$<br>thiophene tolerated | |
| 56 | | Amination | 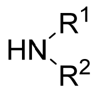 | 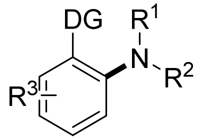 | Substrate (0.5 mmol), amine (2 equiv), Cu(BTC) (25 mol%), NMO (2 equiv), NMP, 90 °C, 6h.<br>12 Examples; Yield: 40-85%<br>$R^1$ - $R^2$ = Morpholine, piperidine, pyrrolidine<br>$R^1$ = $^n$Hex, Ph, Bn<br>$R^2$ = Me, H<br>$R^3$ = OMe, Me, $CF_3$ | 101 |
| 57 | | Amination | 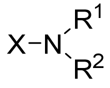<br>X = Cl or OBz | 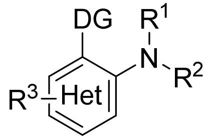 | Substrate (1 equiv), PhMgBr/ THF (3.2−3.4 equiv), $Fe(acac)_3$ (10 mol%),and F-dppbz (15 mol%), amine (2.7 equiv), 65 °C.<br>22 Examples; Yield: 54-99%<br>$R^1$ - $R^2$ = Morpholine<br>$R^1$ = Alkyl<br>$R^2$ = Alkyl, benzyl<br>$R^3$ = Me, OMe, $CF_3$, halogen, $NMe_2$<br>thiophene and indole tolerated | 102 |
| 58 | | Amination | 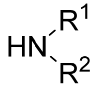 | 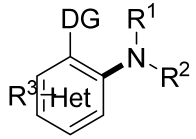 | Substrate (0.5 mmol), $Cu(OAc)_2$ (10-25 mol%), $Ag_2CO_3$ (12-25 mol%), NMP (2 mL), 110 °C, 11-25h<br>20 examples; Yield: 20-87%<br>$R^1$ = H, Me<br>$R^2$ = Bn, alkyl<br>$R^3$ = OMe, F, $CF_3$, alkyl, COOMe<br>heterocycles tolerated | 103 |

| | | | | | | |
|---|---|---|---|---|---|---|
| 59 | | Amino-Alkenylation | 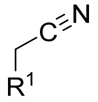 | 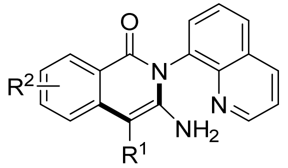 | Substrate (0.4 mmol), ethyl cyanate (1.2 mmol), $Cu(OAc)_2$ (1.2 mmol), $Na_2CO_3$, DMSO, 90 °C, 4–6 h, Ar.<br>24 examples, 49-95% yield<br>$R^1$ = COOR, $CON(Me)_2$, $SO_2CH_3$, $PO(OEt)_2$ | 104 |
| 60 | | Arylation | ArBr | 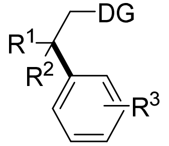 | Substrate (0.3 mmol), ArBr (1.2 mmol), $Pd(TFA)_2$ (0.015mmol), $K_2CO_3$(1.05 mmol), PivOH (0.15 mmol),$^t$Amyl-OH (0.5mL), 120 - 140 °C, 36 h<br>24 Examples; Yield: 9-94%<br>$R^1$ = H, alkyl<br>$R^2$ = Alkyl, Ph<br>$R^3$ = OMe, Me, halogen, Ph, CN, $NO_2$, CHO, $CF_3$, $OCF_3$, OH, NHCOMe; heterocycles tolerated | 105 |
| 61 | | Arylation | 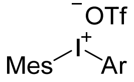 | 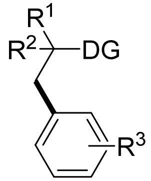 | Substrate (0.3 mmol), diaryliodonium salt (0.36 mmol), $Ni(OTf)_2$ (0.03 mmol), $Na_2CO_3$ (0.6 mmol), MTHP (1 mL), 140 °C, 24 h<br>20 Examples; Yield: 11-93% (NMR yields)<br>$R^1$ = Ph, Bn, alkyl<br>$R^2$ = Ph, alkyl<br>$R^3$ = $CF_3$, COOMe, Ac, $NO_2$, Cl, OMe, Me | 106 |
| 62 | | Arylation | ArI | 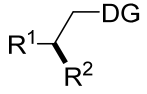 | Substrate (1 equiv), ArI (4-6 equiv), $Pd(OAc)_2$ (0.1-5 mol%), AgOAc (1.1-4.1 equiv), 70-130 °C, 5 min-16 h<br>5 Examples; Yield: 60-93%<br>$R^1$ = Alkyl<br>$R^2$ = Ph, alkyl | 44 |
| 63 | | Arylation | ArI | 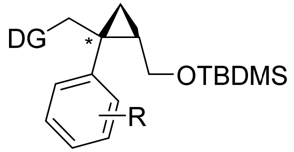 | *cis*-Substrate: Substrate (0.05 mmol), ArI (3 equiv), $Pd(OAc)_2$ (10 mol%), AgOAc (1.5 equiv), toluene (0.2 M), 80 °C, 6 h. | 107 |

| | | | | | | |
|---|---|---|---|---|---|---|
| | | | | | 14 Examples; Yield: 39-95%<br>R = C(O)Me, COOMe, OMe, halogen, CHO, $CH_2OH$;<br>heterocycles tolerated<br>*trans*-Substrate: Substrate (0.05 mmol), ArI (3 equiv), $Pd(OAc)_2$ (10 mol%), AgOAc (1.5 equiv), $K_3PO_4$ (1 equiv), toluene (0.2 M), 80 °C, 6 h.<br>10 Examples; Yield: 31-71%<br>R = C(O)Me, COOMe, OMe, halogen, CHO, $CH_2OH$, CN, halogen; heterocycles tolerated | |
| 64 | | Arylation | ArBr | 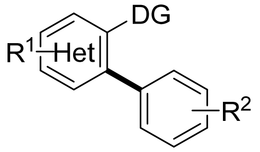 | Substrate (0.3 mmol), ArBr (0.36 mmol), $[RuCl_2(p\text{-}cymene)]_2$ (5 mol%), $PPh_3$ (40 mol%), $Na_2CO_3$ (2 equiv), toluene (2 mL), 130 °C, 15 h.<br>30 Examples; Yield: 43-96%<br>$R^1$ = OMe, OAc, Ph, F, Me; thiophene, pyrrole, chinolin tolerated<br>$R^2$ = $NMe_2$, OMe, Ph, Cl, COOMe, $CF_3$, C(O)Me; pyridine and thiophene tolerated<br>PhCl and PhOTf also tolerated | [48] |
| 65 | | Arylation | ArI | 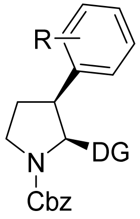 | Substrate (0.9 mmol), ArI (1.8 equiv), AgOAc (1.8 equiv), 110 °C, 20 h, neat<br>27 Examples; Yield: 22-91%<br>R = Me, halogen, OMe, $CF_3$, COOEt, CN, $NO_2$, C(O)Me, CHO, $CH_2OH$<br>pyridine and thiophene tolerated<br>*cis*-selective | [108] |

| | | | | | | |
|---|---|---|---|---|---|---|
| 66 | | Arylation | ArI | 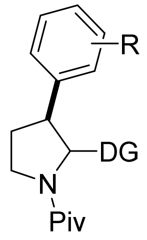 | Substrate (0.2 mmol), ArI (1.0 mmol), $Pd(OAc)_2$ (10 mol%), AgOAc (0.4 mmol), $(BnO)_2PO_2H$ (0.04 mmol), toluene (2 mL), 110 °C, 24 h.<br>26 Examples; Yield: 26-93%<br>R = Alkyl, halogen, $CF_3$, CN, COOMe, $NO_2$, OMe thiophene and pyridine tolerated | 109 |
| 67 | | Arylation | ArI | 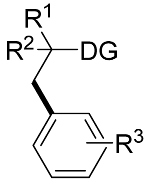 | Amide (0.3 mmol), ArI (0.6 mmol), $Ni(OTf)_2$ (0.1 equiv), MesCOOH (0.2 equiv), $Na_2CO_3$ (2 equiv), DMF (0.6 mL), 140 °C, 24 h<br>21 Examples; Yield: 29-83%<br>$R^1$ –$R^2$ = Alkyl, Bn, Ph<br>$R^3$ = $CF_3$, COOMe, Ac, I, Cl, Me, $NH_2$, $N(Me)_2$, OMe, Me, indole and thiophene tolerated | 110 |
| 68 | | Arylation | ArI | 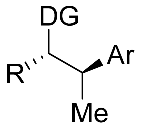 | (±)-Substrate (0.25 mmol), ArI (4 equiv), $Pd(OAc)_2$ (5 mol%), AgOAc (2.2 equiv), toluene (3 mL), 110 °C, 24h.<br>20 Examples, Yield: 40-93%; dr: 52:48 – 86:14<br>R = Ph, H, COOEt, $^t$Bu<br>good diastereoselectivity only if R = Ph | 111 |
| 69 | | Arylation | ArI | 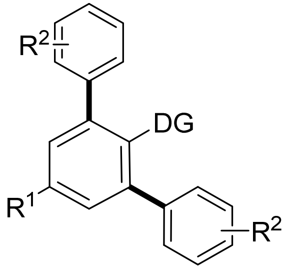 | 8-Amino quinoline (0.3 mmol), benzoyl chloride (0.3 mmol), ArI (0.9 mmol), $Pd(OAc)_2$ (3 mol%), $K_2CO_3$ (0.6 mmol), xylene (2 mL), 120 °C , 12 h.<br>28 Examples; Yield: 47-95%<br>$R^1$ = Me, OMe, Cl<br>$R^2$ = OMe, Me, C(O)Me, $NO_2$, Cl<br>if $R^1$ is not in *meta*-position, monoarylation occurs<br>DG introduction in-situ | 112 |

| | | | | | | | |
|---|---|---|---|---|---|---|---|
| 70 | | Arylation | PyrI | 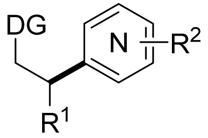 | Substrate (0.1 mmol), $Pd(OAc)_2$ (10 mol%), $Ag_2CO_3$ (1.5 equiv), NaI (30 mol%), $(BuO)_2POOH$ (20 mol%), (toluene-DMA 20/1), 130 °C, 24 h<br>27 Examples; Yield: 52-97%<br>$R^1$ = Alkyl, aryl<br>$R^2$ = Halogen, $CF_3$, CN, COOMe, OMe, Me, NHAc, $NPr_2$, NHPr | 113 |
| 71 | | Carbonylation (Intramolecular Cyclization) | DMF | 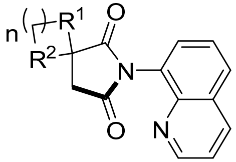 | Substrate (0.2 mmol), $NiBr_2$ (10 mol%), $Cu(acac)_2$ (20 mol%), $Na_2CO_3$ (0.3 equiv), $TBAPF_6$ (1.5 equiv), $O_2$ (1 atm), DMF (5 mL), 160 °C, 24 h.<br>$R^1$ = Alkyl, Ph, Bn, $CF_3$, COOEt<br>$R^2$ = Alkyl<br>n = 2-4<br>The carbon atom in $\alpha$-position to the initial carbonylation must be quaternary | 114 |
| 72 | | Carbonylation (Intramolecular Cyclization) | DMF | 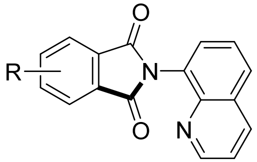 | Substrate (0.2 mmol), $NiI_2$ (10 mol%), $Cu(acac)_2$ (20 mol%), $Li_2CO_3$ (0.4 equiv), THAB (1 equiv), $O_2$ (1 atm), DMF (3.0 mL), 160 °C, 24 h.<br>14 Examples; Yield: 51-90%<br>R = OMe, Me, halogen, $CF_3$, $NO_2$ | 114 |
| 73 | | Carbonylation (Intramolecular Cyclization) | CO | 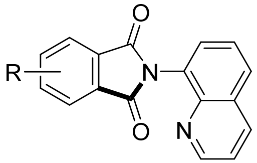 | Substrate (0.5 mmol), $(Co(acac)_2$ (20 mol%), NaOPiv (2 equiv), $Mn(OAc)_3\cdot 2H_2O$ (1 equiv), CO (1 atm), $CF_3CH_2OH$ (5 mL), rt, 16-60 h<br>12 examples, 60-94% yield<br>R = Me, $CF_3$, I, Br, $NO_2$, OMe, $OCF_3$, CN, COOEt | 115 |

| | | | | | | |
|---|---|---|---|---|---|---|
| 74 | | Etherification | $R^1OH$ | 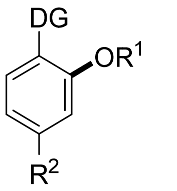 | $R^1$=Ar: Substrate (0.5 mmol), ArOH (0.5 mmol), $Cu(OH)_2CO_3$ (11 mol-%), $K_2CO_3$ (2 equiv), DMF, 110 °C, air.<br>$R^1$= Alkyl: Substrate (0.5 mmol), ROH (5 equiv), $Cu(OH)_2CO_3$ (11 mol-%), TMG (2 equiv), pyridine, 110 °C, air.<br>23 Examples; Yield: 39-85%<br>$R^1$ = Aryl, alkyl<br>$R^2$ = $CF_3$, $NO_2$, CN, OMe, Me<br>pyridine tolerated | 116 |
| 75 | | Fluorination | AgF | 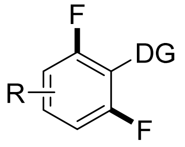 | Monofluorination: Substrate (0.25 mmol), CuI (10–25 mol-%), AgF (3.5–4.0 equiv), NMO (4.5–5.0 equiv) in DMF (1 mL), 50–125 °C, 30–120 min.<br>R = $CF_3$, COOMe, CN, OMe, F, Me<br>pyridine tolerated<br>Difluorination: Substrate (0.25 mmol), CuI (18 – 30 mol%), pyridine (2 equiv), AgF (5–6 equiv), NMO (7–8 equiv), DMF (1 mL), 75–105 °C, 1.5–2 h<br>8 Examples; Yield: 61-77%<br>R = $CF_3$, COOMe, CN, OMe, F, Me<br>pyridine tolerated | 117 |
| 76 | | Nitration | $NaNO_2$ | 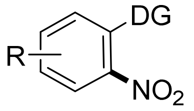 | Mononitration: Substrate (0.3 mmol), $NaNO_2$ (0.9 mmol), $Cu(OAc)_2 \cdot H_2O$ (0.6 mmol), $K_2HPO_4$ (0.6 mmol), MeOH (1 mL) air, 12 h<br>19 Examples; Yield: 57-76%<br>R = Alkyl, OMe, Ph, halogen, COOMe, $CF_3$<br>heterocycles tolerated | 118 |

| | | | | | | |
|---|---|---|---|---|---|---|
| | | | | | Dinitration: Substrate (0.3 mmol), $NaNO_2$ (0.9 mmol), $Cu(OAc)_2 \cdot H_2O$ (0.6 mmol), AgOAc (0.6 mmol), DMF (1 mL), air, 12 h<br>10 Examples; Yield: 47-63%<br>R= Alkyl, OMe, F, COOMe<br>pyridine tolerated | |
| 77 | | Phosphorylation | 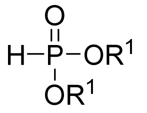 | 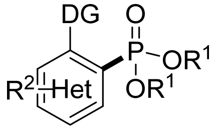 | Substrate (0.2 mmol), $HPO(OR^1)_2$ (2 equiv), $Cu(OAc)_2$ (20 mol%), NMO (2 equiv), $Ag_2CO_3$ (1 equiv), DMSO (0.8 mL), 4Å MS, 55 °C, 12h.<br>24 examples, 36-78% yield<br>$R^1$ = *i*-Pr, Et, *n*-Hex<br>$R^2$ = Me, OMe, Halogen, $CF_3$, $NO_2$, CN, COOMe,<br>pyridine, thiophene tolerated | 119 |
| 78 | | Silylation | $Me_3Si–SiMe_3$ (or $Me_3Ge–GeMe_3$) | 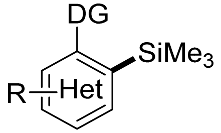 | Substrate (0.2 mmol), $Me_6Si_2$ (1.0 mmol), $Pd(OAc)_2$ (10 mol%), $Ag_2CO_3$ (0.4 mmol), $CaSO_4$ (0.4 mmol), 1,4-dioxane (1.0 mL), 130 °C.<br>17 examples, 28 – 82% yield<br>R = Me, COOMe, OMe, OBn, OAc, C(O)Me, $SO_3R$, $CF_3$; thiophene, benzofuran tolerated | 120 |
| 79 | | Sulfenylation | $F_3CS-SCF_3$ | 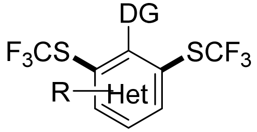 | Substrate (1 equiv), $CF_3S-SCF_3$(2−2.5 equiv), $Cu(OAc)_2$ (0.5 equiv), DMSO, 90−110 °C.<br>10 examples, 43-76% yield<br>R = *t*-Bu, Cl, OMe, Br, F, COOMe<br>pyridine, thiophenetolerated | 121 |
| 80 | | Sulfenylation | ArS-SAr | 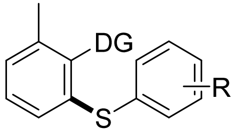 | Substrate (1 mmol), diaryl disulfide (2 mmol), $NiCl_2$ (20 mol%), $PPh_3$ (20 mol%), $Cs_2CO_3$ (1 mmol), and dioxane (1 mL), 21 h, 140 °C | 122 |

| | | | | | | |
|---|---|---|---|---|---|---|
| | | | | | 7 examples, 36-98% yield<br>R = OMe, Me, Cl | |
| 81 | | Sulfenylation | 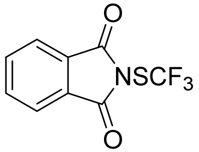 | 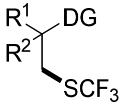 | Substrate (0.266 mmol), $SF_3$-source (0.2 mmol), $Pd[CH_3CN]_2Cl_2$ (20 mol%), PivOH (10 equiv), DMF (2 mL), 70 °C, Ar.<br>22 examples, 8-53% yield<br>$R^1$ = Me, H<br>$R^2$ = Alkyl, Aryl | 123 |
| 82 | | Sulfenylation | $R^2SH$ | 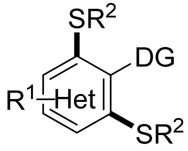 | Substrate (0.2 mmol), thiol (2.5 equiv), $Cu(OAc)_2 \cdot H_2O$ (20 mol%), $Ag_2CO_3$ (2 equiv), bathophen (40 mol%), NMP (2mL), 110 °C, 24-48 h, Ar.<br>30 examples, 16-93% yield<br>$R^1$ = Me, F, Br, $CF_3$, *t*-Bu, CN, $NO_2$,<br>$R^2$ = Alkyl, Aryl | 124 |
| 83 | | Sulfenylation | RS-SR | 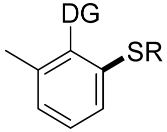 | Substrate (1 equiv), disulfide (2−2.5 equiv), $Cu(OAc)_2$ (0.5 equiv), DMSO, 100−110 °C.<br>6 examples, 69-90% yield<br>R = Ph, *i*-Pr, *t*-Bu, *n*-Bu, Bn, Aryl | 121 |
| 84 | | Sulfonylation | $ArSO_2Cl$ | 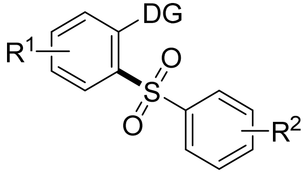 | Substrate (1.0 mmol), sulfonyl chloride (3.0 mmol), $NiCl_2$ (50 mol%), $Na_2CO_3$ (2.0 mmol), dioxan (1.0 mL), 24 h, 140 °C.<br>13 examples, 33-54 % yield<br>$R^1$ = Me, $CF_3$<br>$R^2$ = Me, F, $CF_3$, $^t$Bu | 122 |
| 85 | | Sulfonylation | $ArSO_2Na$ | 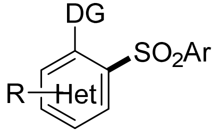 | Substrate (0.3 mmol), sodium sulfinate (0.6 mmol), $Cu(OAc)_2$ (0.3 mmol), $K_2CO_3$ (0.6 mmol), DMF (1 mL), air, 4 h. | 125 |

| | | | | | | |
|---|---|---|---|---|---|---|
| | | | | | 25 examples, 42-80 % yield<br>R = OMe, Me, *t*-Bu, F, Cl, Br, $CF_3$, COOMe, $NO_2$<br>Het = pyridine, thiophene | |
| 86 | | Sulfonylation | $ArSO_2Cl$ | 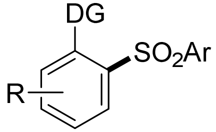 | Substrate (0.3mmol), arylsulfonyl chloride (0.36 mmol), $PPh_3$ (0.06 mmol), $NaHCO_3$ (0.6 mmol), $Ni(OTf)_2$ (0.03 mmol), toluene (2 mL) at 160 °C, 24h.<br>11 examples, 21-85% yield<br>R = Me, OMe, F, $CF_3$ | 126 |
| 87 | 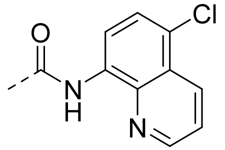 | Benzylation | $CH_3$-Ar | 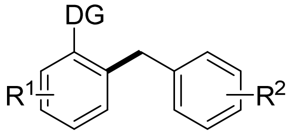 | Substrate (0.3 mmol), $Ni(OTf)_2$ (0.03 mmol), $PPh_3$ (0.03 mmol), $Na_2CO_3$ (0.6 mmol), and ${}^{i}C_3H_7I$ (0.6 mmol), toluene (1 mL), 140 °C, 24 h.<br>28 Examples; Yield: 42-95%<br>$R^1$ = Me, OMe, Cl, $CF_3$, C(O)Me, F,<br>$R^2$ = $CF_3$, COOMe, Me, OMe, halogen, NHAc | 127 |
| 88 | 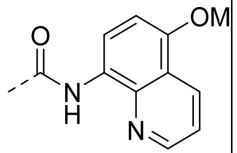 | Amidation (Intramolecular) | | 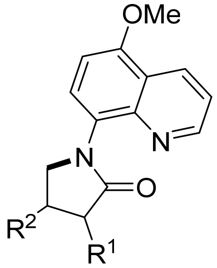 | Substrate (0.2 mmol), $Pd(OAc)_2$ (5 mol%), $PhI(OAc)_2$ (2.5 equiv), toluene, 70-110 °C, Ar, 24 h.<br>7 Examples, Yield: 59-87%<br>$R^1$ = Me, ${}^{t}$BuO<br>$R^2$ = NHPhth, H | 98 |
| 89 | 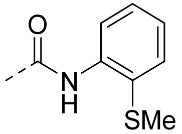 | Arylation | ArI | 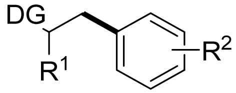 | Substrate (1 equiv), ArI (3-4 equiv), $Pd(OAc)_2$ (0.05 equiv), CsOAc or $K_2CO_3$ (2.5 equiv), toluene, 90-110 °C<br>7 Examples; Yield: 47-79%<br>$R^1$ = Alkyl, OBn, phthalimide<br>$R^2$ = Br, $OCF_3$, alkyl, Cl | 89 |

| | | | | | | |
|---|---|---|---|---|---|---|
| 90 | | Arylation | ArI | 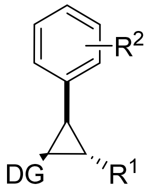 | Susbtrate (0.25 mmol), ArI (1 mmol), Pd(OAc)2 (5 mol%), AgOAc (0.55 mmol), toluene (2-3 mL), 110 °C, 12-24 h<br>10 Examples; Yield: 40-88%<br>$R^1$ = H, Ph<br>$R^2$ = $NO_2$, OMe, alkyl, Ac; thipophene tolerated | 128 |
| 91 | | Arylation | ArI | 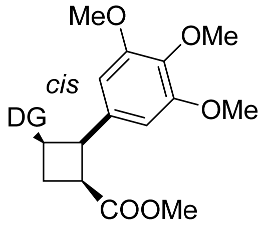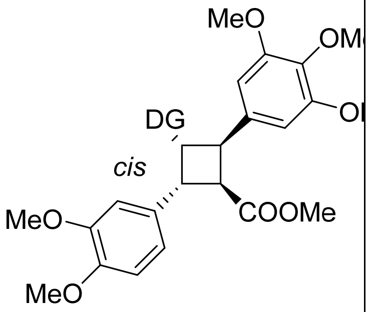 | Substrate (1 equiv), ArI (2 equiv), Pd(OAc)2 (0.15 equiv), Ag2CO3 (1.5 equiv), PivOH (1 equiv), HFIP, 90 °C, 36 h<br>3 Examples; 46-81%<br>applied in the total synthesis of piperarborenine B and D<br>up to 20% diarylated byproduct<br>gram scale | 129 |

## Pyrimidine as directing group in C-H activation chemistry

Pyrimidine is after pyridine the second most frequently applied heterocyclic directing group. Typically, the 2-position of pyrimidine is connected to the substrate to be functionalized. If this attachment is via a C-C bond, pyrimidine is a permanent directing group and will remain in the final substrate. This of course is a drawback and limits the applicability to products which have pyrimidine as part of their structure. In case of a connection to a heteroatom, e.g. nitrogen, the pyrimidine DG can be cleaved using relatively simple conditions (e.g. NaOMe, DMSO, 100 °C). The more facile cleavage as compared to pyridine, where typically a twostep process of either reduction and hydrolysis or N-alkylation and hydrolysis is required, is due to the high electrophilicity of the C2 position due to electron withdrawal of 3 adjacent heteroatoms.

Palladium catalyzed acetoxylation and arylation was reported by the group of Chen (Table 3, Entry 1 & 27).[31] In their case pyrimidine is linked via an oxygen to the system to be functionalized opening potential for DG cleavage, even though this has not been demonstrated. For arylation (Table 3, Entry 27) readily available arylboronic acids were used as coupling partners, whereas the acetoxylation (Table 3, Entry 1) relied on a hypervalent iodine reagent, namely $PhI(OAc)_2$. Both transformations showed a broad functional group tolerance.

Jiao and coworkers investigated the direct acylation (Table 3, Entry 2) and nitration (Table 3, Entry 38, 46) of arenes using series of directing groups including pyridine and pyrimidine.[40] For the nitration, *t*-butylnitrate (TBN) is the source of a $NO_2$ radical which is formed under aerobic conditions, giving overall a PdII/PdIV catalytic cycle. For the acylation, toluene serves as the source of a benzoyl radical in a twostep process. Initially, *N*-hydroxyphthalimide (NHPI) is converted under aerobic conditions to a phthalimido-*N*-oxyl (PINO) radical, which converts toluene to the benzoyl radical via single electron transfer (SET).

Alkenylation reactions of *N*-(2-pyrimidinyl)indole derivatives in position 2 of the indole ring have been reported with several different alkene sources (Table 3, Entries 3-8). The group of Ackermann applied alkenyl acetates (Table 3, Entry 5), alkenyl carbamates (Table 3, Entry 6), alkenyl phosphates (Table 3, Entry 7), and alkenyl carbonates (Table 3, Entry 8) under identical conditions.[130] Notable, the catalytic system consisted of the non-precious metal cobalt, which is of course highly desirable in metal catalysis. The simple salt $CoI_2$ in combination with a NHC ligand and either CyMgCl or DMPU as base promoted the desired transformation. More common are acrylates as reaction partners in alkenylations reactions.[131] However, in such cases (e.g. Table entries 3&4) the substrate scope regarding the olefine is naturally limited.

Manganese catalyzed alkenylation of indole with alkynes was reported by Lei and Li (Table 3, Entries 9 & 26).[21] As non-precious metal, manganese catalysis is especially attractive. In the specific example, depending on the reaction conditions, either the alkenylation product or annulation to carbazoles via twofold reaction with the alkyne component was observed. For both products also cleavage of the DG was demonstrated.

Alkylation reactions are a common transformation in C-H activation chemistry. The most frequently applied alkyl source are actually olefins and also for pyrimidine as DG such examples have been reported (Table 3, Entries 10, 12, 44).[24, 132] An interesting alternative was published in 2015 by Jain and co-workers (Table 3, Entry 15). In their protocol, aliphatic carboxylic acids are used giving overall a decarboxylative alkylation reaction under palladium catalysis.[133] *N*-2-Pyrimidinylindolines were used as substrates

leading to alkylation in position 7. Examples with secondary and mainly tertiary carboxylic acids were reported. Decarboxylative arylation is typically more common and was applied to the C2 arylation of indole (Table 3, Entry 29).[134]

Alkylation via ring opening of 2-vinyloxirane was reported under Rh catalysis by the group of Li (Table 3, Entry 14).[26] Yields were usually very high, often surpassing 90%. One drawback is the poor E/Z selectivity of the double bond in the products, which is often around 2:1, at best 6.1:1.

Meta selective alkylation has been reported by Ackermann and coworkers (Table 3, Entry 17).[135] Naturally, the pyrimidine DG mainly applied cannot reach out far enough to direct Ru insertion into the *meta* C-H bond. Indeed, "normal" *ortho* C-H insertion takes place. This leads to a metal complex in which the bond *para* to the Ru-C bond is weakened and prone to attack by a radical species formed from the tertiary alkyl bromides. This position para to the initially formed Ru-C bond is concomitantly *meta* to the position of the directing group, hence the observed meta alkylation. Detailed mechanistic studies have been carried out which support this mechanistic proposal.

A very comprehensive study on the alkynylation of various substrates has been reported by Xingwei Li and coworkers (Table 3, Entries 18-20).[28] They identified a catalytic system consisting of $[RhCp^*Cl_2]_2$ and $Zn(OTf)_2$, hypervalent iodine-alkyne reagents which worked for the alkynylation of aromatics in combination with a series of heterocyclic DGs, including pyrimidine, pyridine and pyrrazole. Tuning the catalytic system allowed also the used of several other DGs such as *N*-methoxy imines, azomethine imines, secondary carboxamides, azo compounds, *N*-nitrosoamines, and nitrones (see corresponding sections).

Amides are important functional groups in organic chemistry and naturally the introduction of this functionality via C-H activation chemistry was investigated. Amidation reactions have been reported under rhodium catalysis using either isocyanates, *N*-hydroxycarbamates, or *N*-(2,4,6-trichlorobenzolyloxy)amides as amidating reagents (Table 3, Entries 21-25, 45). The latter one shows significant drawbacks regarding atom efficiency, which foils somehow the idea of C-H activation (Table 3, Entry 21).[136] Still, it gives very reliable results, only large amides such as pivaloylamide cannot be introduced via this method. Isocyanates are of course very reactive species and proved to be reliable amide precursors (Table 3, Entry 24).[137] It is worth mentioning that in a DG screening the usually very efficient 2-pyridyl group was significantly outperformed by the 2-pyrimidyl group. Most examples were carried out using aliphatic isocyanates, however also aromatic ones did work but with mediocre yields <50%.

Cyclopropenone ring opening towards chalcones was reported by the group of Li (Table 3, Entry 33).[138] With a single set of reaction conditions ($[RhCp^*Cl_2]_2$ and $AgSbF_6$) the transformation worked in combination with a series of directing groups including 2-pyrimidyl (others were 2-pyridyl, *N*-pyrazyl, and *N*-methoxy imine). These reaction conditions are quite common in C-H activation chemistry. The non coordinationg anion $SbF_6^-$ facilitates the formation of a cationic Rh-species which undergoes C-H insertion. Noteworthy, the authors were able to isolate several Rh(III) complexes and could show that they are part of the catalytic cycle. Hence, a mechanism was proposed strongly supported by experimental evidence.

Meta selective bromination by using NBS as brominating reagent was reported in a study focused on 2-aryl pyridines as substrates, but in three cases also pyrimidine was used as DG (Table 3, Entry 30).[139] The catalyst naturally inserts into the C-H bond ortho to the directing group. This activates the position *para* to this Ru-C bond for attack by a bromine radical generated from NBS. Hence, the meta bromination in respect to the DG.

Trifluoromethylallylation under Rh-catalysis was reported by Kim and coworkerspyrimidine (Table 3, Entry 42).[39] The reaction was quite selective for the E-configuration of the resulting allyl group with E/Z selectivities ranging from 13:1 up to 35:1.

Cyanation in position 2 of indoles using *t*BuNC as the cyano source was reported by Xu and coworkers (Table 3, Entry 43, 47, 48).[12] The DG overrides the intrinsic reactivity of indoles, where the more electron rich 3-position would be preferentially cyanated in absence of a DG.

Cu-catalyzed oxidative coupling between indoles and benzoxazoles has been reported by Hirano and Miura (Table 3, Entry 34).[140] In this paper, they present a stoichiometric and a catalytic variant of this transformation, the latter one using air as terminal oxidant. Also removal of the DG was reported under standard conditions for cleaving *N*-linked 2-pyrimidyl groups. A second oxidative coupling method was reported as well, this time between indoles and *N*-oxides of 6-membered *N*-heterocycles (Table 3, Entry 35).[13]

A noteworthy example is also the indole synthesis reported by the group of Ackermann (Table 3, Entry 37).[141] In this case *N*-2-pyrimidyl anilines are coupled to alkynes under Ni-catalysis, whereas the two carbons of the triple bond end up as C2 and C3 of the final indole products.

**Table 3: Pyrimidine as directing group in C-H activation chemistry**

| Entry | Directing group | Type of transformation | Coupling partner | Typical product structure | Comments | Ref |
|---|---|---|---|---|---|---|
| 1 | 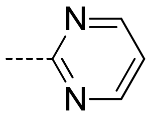 | Acetoxylation | $PhI(OAc)_2$ | 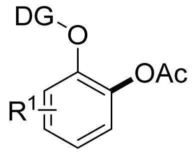 | Substrate (1 equiv), $PhI(OAc)_2$ (1.1 equiv), $Pd(OAc)_2$ (2 mol%), $AcOH/Ac_2O$, 100 °C, 2-12 h;<br>$R^1$ = naphthyl, Cl, Me, COOMe, OMe.<br>17 examples, 24-87% | 31 |
| 2 | 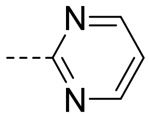 | Acylation | 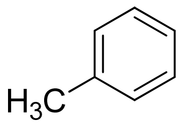 | 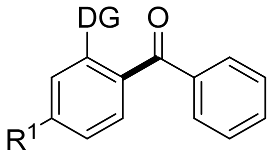 | Substrate (0.5 mmol), $Pd(OAc)_2$ (10 mol%), NHPI (20 mol%), toluene (1 mL) at 80 °C under $O_2$ (1 atm) for 24 h.<br>Bis acylation was observed in several cases.<br>2 examples, $R^1$ = H (62%), MeO (83%) | 40 |
| 3 | 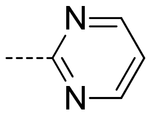 | Alkenylation | 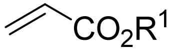 | 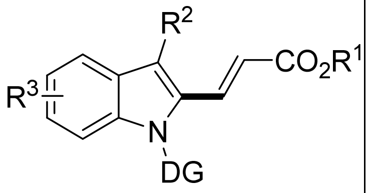 | Substrate (0.25 mmol), alkene (0.30 mmol), $[Cp^*RhCl_2]_2$ (5.0 mol%), and $Cu(OAc)_2.H_2O$ (1 equiv) in DCE under air at 60 °C for 5h.<br>$R^1$ = *n*Bu, *t*Bu, Me; $R^2$ = H, Me, Br, I; $R^3$ = H, OME, CN, Cl, F, $NO_2$, COOMe;<br>20 examples, (25-93% isolated yield)<br>DG removed via NaOEt in DMSO, 100 °C. | 131 |
| 4 | 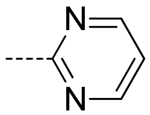 | Alkenylation | 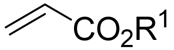 | 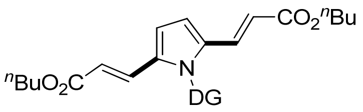 | Substrate (0.25 mmol), Alkene (3 equiv), $[Cp^*RhCl_2]_2$ (5.0 mol%), and $Cu(OAc)_2.H_2O$ (1 equiv) in DCE under air at 60 °C for 5 h.<br>Single example, 75%; No reaction at rt. | 131 |
| 5 | 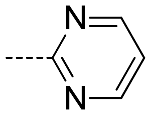 | Alkenylation | 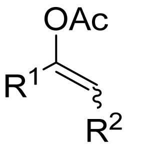 | 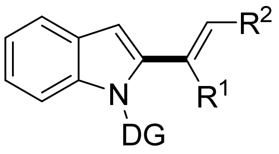 | Substrate (0.50 mmol), acetate (0.75 mmol), $CoI_2$ (10 mol%), ligand IPrHCl (10 mol%), CyMgCl (2.0 equiv), DMPU (1.5 mL), 23 °C, 16 h.<br>$R^1$ = *n*Pr, *n*Bu, Ph; $R^2$ = Et, *n*Pr, Me;<br>4 examples, 50-80% | 130 |

| | | | | | | |
|---|---|---|---|---|---|---|
| | | | | | Removal: NaOMe, DMSO 100 °C | |
| 6 | 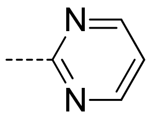 | Alkenylation | 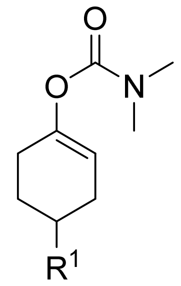 | 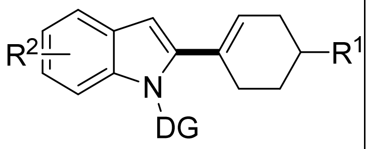 | Substrate (0.50 mmol), carbamate (0.75 mmol), $CoI_2$ (10 mol%), ligand IPrHCl (10 mol%), CyMgCl (2.0 equiv), DMPU (1.5 mL), 23 °C, 16 h.<br>$R^1$ = H (87%), *n*Pent (82%), O*n*Pent (79%);<br>Removal: NaOMe, DMSO 100 °C | 130 |
| 7 | 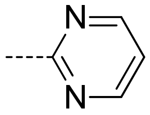 | Alkenylation | 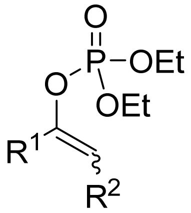 | 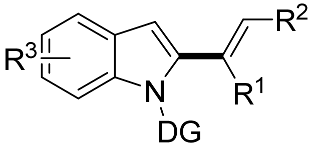 | Substrate (0.50 mmol), phosphate (0.75 mmol), $CoI_2$ (10 mol%), ligand IPrHCl (10 mol%), CyMgCl (2.0 equiv), DMPU (1.5 mL), 23 °C, 16 h.<br>$R^1$ = *n*Pr, *n*Bu, Ph; $R^2$ = Et, *n*Pr, Me; $R^3$ = H, OEt, F;<br>Also cyclohexene-phosphates were used successfully<br>11 examples, 50-83%<br>Removal: NaOMe, DMSO 100 °C | 130 |
| 8 | 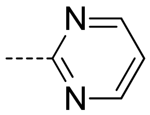 | Alkenylation | 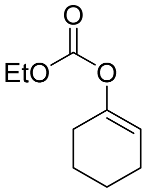 | 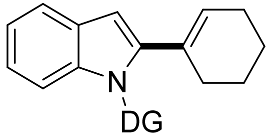 | Substrate (0.50 mmol), carbonate (0.75 mmol), $CoI_2$ (10 mol%), ligand IPrHCl (10 mol%), CyMgCl (2.0 equiv), DMPU (1.5 mL), 23 °C, 16 h.<br>Single example, 56% | 130 |
| 9 | 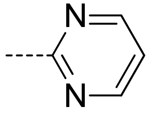 | Alkenylation | 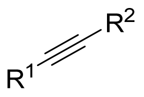 | 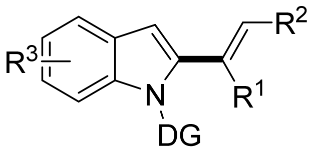 | Substrate (1 equiv), alkyne (1.5 equiv), $MnBr(CO)_5$ (10 mol%), DIPEA (20 mol%), PhCOOH (20 mol%), $Et_2O$, 80 °C, Ar, 12 h.<br>$R^1$ = H, Me, Ph, COOEt, 2-thienyl, 4-tolyl; $R^2$ = aryl, COOEt, 2-thienyl; $R^3$ = H, F, Cl, Br, Me, MeO;<br>24 examples, 17-98%.<br>DG removal: NAOMe (5 equiv), DMSO, 110 °C, 24 h, 1 example, 62%. | 21 |

| | | | | | | |
|---|---|---|---|---|---|---|
| | | | | | Pyrrole as substrate gives bis-alkenylation ($R^{1,2}$ = Ph, 41%). | |
| 10 | 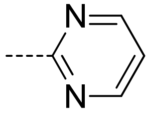 | Alkylation | 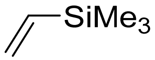 | 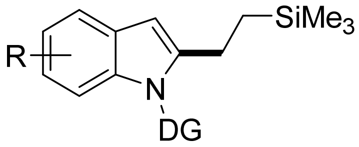 | Substrate (5.0 mmol), vinylsilane (1.5 equiv), $CoBr_2$ (10 mol-%), bathocup (10 mol-%), CyMgBr (60 mol-%) in THF (2 mL) at 60 °C for 12 h.<br>R = H, F, Cl, MeO, Me, Et; other vinylsilanes applied: $CH_2$=$CHSiPh_3$ and $CH_2$=$CHSiMe_2Ph$; 11 examples, 30-80%<br>Reaction also in gram scale reported.<br>Removal: NaOEt in DMSO, 100 °C, 12h. | 132 |
| 11 | 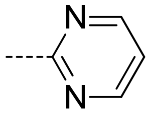 | Alkylation | Alk-Cl | 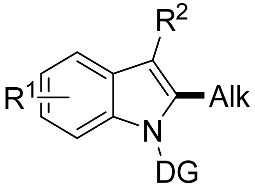 | Substrate (1 equiv), Alk-Cl (1.2 equiv), $Co(acac)_2$ (10 mol%), IPrHCl (20 mol%), CyMgCl, DMPU, 23 °C, 16 h.<br>$R^1$ = H, Et; Alk = *n*Hex, *n*Oct, $(CH_2)_3Ph$;<br>4 examples, 71-86%. | 16 |
| 12 | 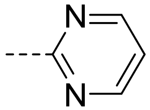 | Alkylation | 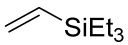 | 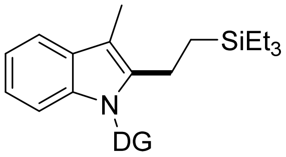 | Substrate (1 equiv), alkene (3 equiv), $[RuCl_2(p\text{-cymene})]_2$ (2.5 - 5 mol%), MesCOOK (30 mol%), toluene, 100 – 120 °C, 18 – 24 h.<br>Single example, 83%. | 24 |
| 13 | 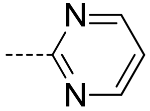 | Alkylation via aziridine opening | 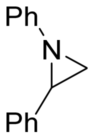 | 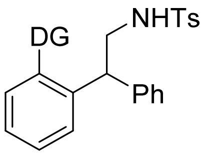 | Substrate (1 equiv), aziridine (2 equiv), $[Cp^*RhCl_2]_2$ (5 mol%), $AgSbF_6$ (30 mol%), PhCl, 100 °C, 20 h.<br>The reaction was developed for 2-arylpyridine derivatives (21 examples, 48-90%)<br>Single example with pyrimidine as DG, 63% | 25 |
| 14 | 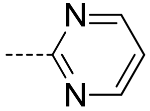 | Alkylation | 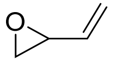 | 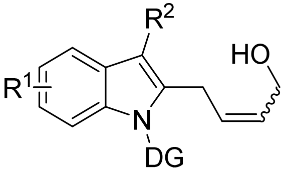 | Substrate (1 equiv), 2-vinyloxirane (1.2 equiv), PivOH (1 equiv), $[Cp^*Rh(MeCN)_3]SbF_6$ (3 mol%), Ar, 25 °C, 16 h<br>$R^1$ = H, Me, Et, OBn, OMe, Br, Cl, COOMe; $R^2$ = H, Me, $CH_2CH(NHBoc)(COOMe)$;<br>17 examples, 59-97% (E/Z = 1.5 : 1 – 6.1 : 1) | 26 |

| | | | | | | |
|---|---|---|---|---|---|---|
| 15 | 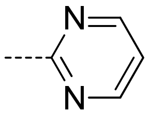 | Alkylation | RCOOH | 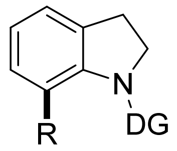 | Substrate (1.0 equiv), reagent (2.0 equiv), $PhI(OAc)_2$ (2.0 equiv), $Pd(OAc)_2$ (10 mol%), 40 °C, 2 h.<br>R = secondary and tertiary alkyls; 10 examples, 73-92%.<br>Inhibited by ascorbic acid. | 133 |
| 16 | 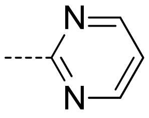 | Alkylation | $R^1$-$BF_3K$ | 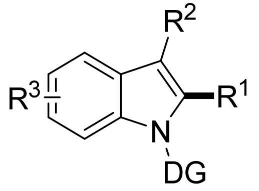 | Substrate (1 equiv), $R^1$-$BF_3K$ (3 equiv), AgF (2.8 - 4 equiv), $[Cp^*RhCl_2]_2$ (4 mol%), $AgSbF_6$ (16 mol%), DCE, 100 °C, 24 h.<br>$R^1$ = Me, *n*Bu; $R^2$ = H, Me; $R^3$ = H, OBn, MeO, Cl, Et;<br>7 examples, 42-91%.<br>Many other DGs successfully applied in this contribution | 11 |
| 17 | 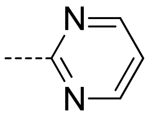 | Alkylation | 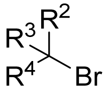 | 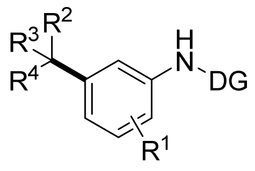 | Substrate (1 equiv), alkyl bromide (3 equiv), $[RuCl_2(p\text{-}cymene)]_2$ (5 mol%), Piv-Val-OH (30 mol%), $K_2CO_3$, 1,4-dioxane, 120 °C, 16 h.<br>$R^1$ = H, OMe, Br, Cl, F; $R^2$ – $R^4$ = various alkyl species;<br>14 examples, 40-66%. | 135 |
| 18 | 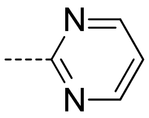 | Alkynylation | 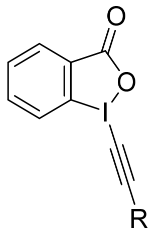 | 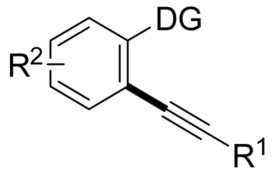 | Substrate (0.2 mmol), R-EBX (0.22 mmol),$[RhCp^*Cl_2]_2$(2 mol%), $Zn(OTf)_2$(0.02 mmol, 10 mol%), DCE (2 mL), 25 or 80 °C, 16 h<br>$R^1$ = TIPS, TES; $R^2$ = 2-Me, 2-Cl, 2-$CF_3$, 4-$CF_3$;<br>4 examples, 50-91%<br>Also thiophene can be selectively mono-alkynylated (80%) | 28 |
| 19 | 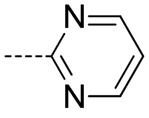 | Alkynylation | 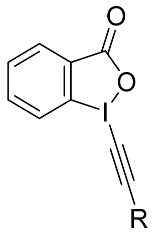 | 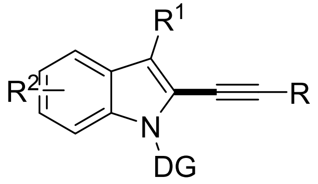 | Substrate (0.2 mmol), R-EBX (0.22 mmol),$[RhCp^*Cl_2]_2$(2 mol%), $Zn(OTf)_2$(0.02 mmol, 10 mol%), DCE (2 mL), 25 or 80 °C, 16 h<br>$R^1$ = TIPS, TES, *t*Bu; $R^2$ = H, Me; $R^3$ = H, OBn, MeO, Cl;<br>5 examples, 85-92%<br>DG cleavage: NaOEt, DMSO | 28 |

| | | | | | | |
|---|---|---|---|---|---|---|
| 20 | 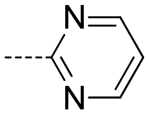 | Alkynylation | 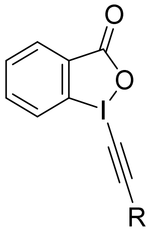 | 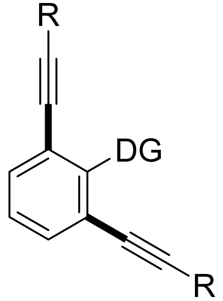 | Substrate (0.2 mmol), R-EBX (0.46 mmol),$[RhCp^*Cl_2]_2$(2 mol%), $Zn(OTf)_2$(0.02 mmol, 10 mol%), DCE (2 mL), 25°C, 16 h<br>R = TIPS (89%), single example | 28 |
| 21 | 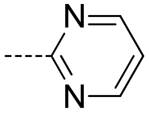 | Amidation | 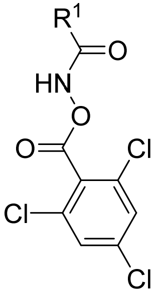 | 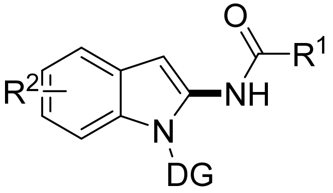 | Substrate (0.20 mmol), acetamide (0.24 mmol), $[Cp^*Rh(MeCN)_3](SbF_6)_2$ (5 mol%), DCE (1 mL), 80 °C, 5-12 h.<br>- No reaction for $R^1$ = t-Bu because of steric hindrance.<br>- DG was removable with EtONa in DMSO, 100 °C.<br>$R^1$ = Me, *i*Pr, Bn, Ph, O*t*Bu, *t*Bu; $R^2$ = H, MeO, CN, Br, F, Cl, NO2, COOMe, Me, CONHBn;<br>24 examples, 54-85% | 136 |
| 22 | 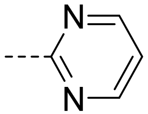 | Amidation | HO-NH-Cbz | 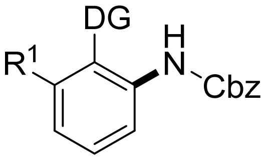 | Substrate (1 equiv), *N*-Hydroxycarbamate (1.2 equiv), $[RhCp^*(CH_3CN)_3](SbF_6)_2$ (2.5 mol%), $Ag_2CO_3$ (1.5 equiv), THF, 100 °C, 10 h, under air.<br>2 examples, $R^1$ = H (60%), Me (64%) | 142 |
| 23 | 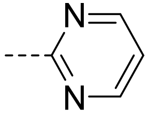 | Amidation | 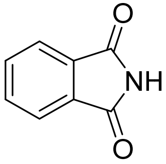 | 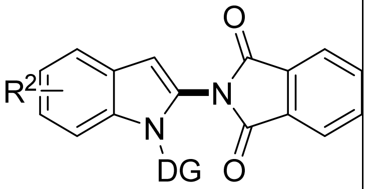 | Substrate (1 equiv), phthalimide (1.2 equiv), CuOAc (20 mol%), toluene/o-dichlorobenzene (1:1), 150 °C, $O_2$, 2-3 days.<br>$R^1$ = H, Me; $R^2$ = H, Me, MeO, F, Cl, Br, CN;<br>8 examples, 31-86% ($NO_2$ was not tolerated) | 29 |
| 24 | 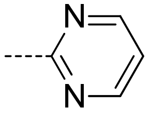 | Amidation | R-NCO | 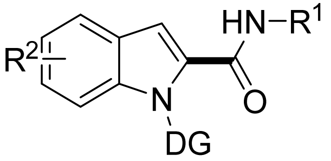 | Substrate ( 1 equiv), isocyanate (3 equiv), $[RhCp^*Cl_2]_2$(2 mol%), AgSbF6 (20 mol %), DCE, 100 °C, 24 h<br>$R^1$ = Et, *n*Bu, *n*Pent, *n*Hex, *n*Oct, Bn, $CH_2CH_2Ph$, cyclopentyl, Ar; $R^2$ = H, OMe, $NO_2$, Br, Cl, F, Me; | 137 |

| | | | | | | |
|---|---|---|---|---|---|---|
| | | | | | Also pyrrole and tetrahydroindole are potential substrates.<br>24 examples, 26-94% | |
| 25 | 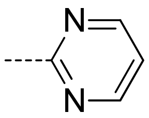 | Amidation | 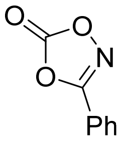 | 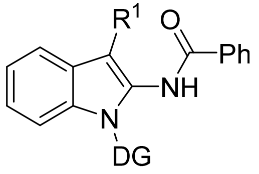 | Substrate (1 equiv), dioxazolone (1.2 equiv), $Cp^*Co(CO)I_2$ (2.5 - 5 mol%), $AgSbF_6$ (5 - 10 mol%), NaOAc (5- 10 mol%), DCE, 70-100 °C, 20 h.<br>$R^1$ = H (94%), Me (65%). | 15 |
| 26 | 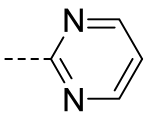 | Annulation | 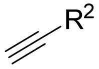 | 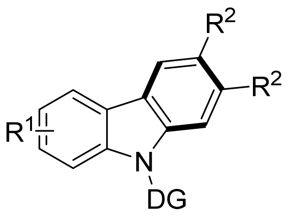 | Substrate (1 equiv), alkyne (2.5 equiv), $MnBr(CO)_5$ (20 mol%), DIPEA (40 mol%), $Et_2O$, 80 °C, Ar, 12 h.<br>$R^1$ = H, 6-Me, 6-Br; $R^2$ = Ph, 4-tolyl, 2-thienyl;<br>5 examples, 14-29%<br>DG removal: NaOMe (5 equiv), DMSO, 110 °C, 24 h, 1 example, 65%. | 21 |
| 27 | 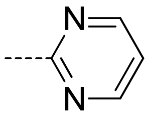 | Arylation | Ar-$B(OH)_2$ | 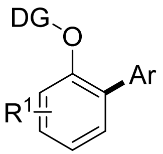 | Substrate (1 equiv), Ar-$B(OH)_2$ (2 equiv), $Pd(OAc)_2$ (5 mol%), $Cu(OTf)_2$ (1 equiv), $Ag_2O$ (1 equiv), toluene, 120 °C, 24 h;<br>$R^1$ = naphthyl, Cl, Me, COOMe, CHO; Ar = Ph, 2-$MeC_6H_4$, 3-$MeC_6H_4$, 4-$MeC_6H_4$, 4-$ClC_6H_4$, 4-$CF_3C_6H_4$;<br>18 examples, 17-75% | 31 |
| 28 | 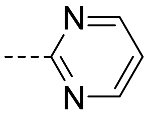 | Arylation | 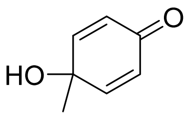 | 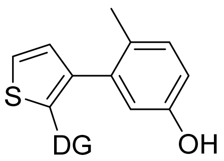 | Substrate (1 equiv), dienone (1.2. equiv), $[Cp^*RhCl_2]_2$ (5 mol%), $AgSbF_6$ (30 mol%), $Zn(NTf_2)_2$ (20 mol%), DCE, 100 °C, 20h<br>Single example, 45% | 35 |
| 29 | 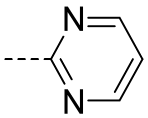 | Arylation | Ar-COOH | 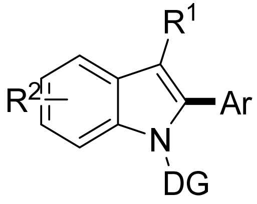 | - Substrate (0.5 mmol), ArCOOH (0.75 mmol), $[Rh(CO)_2Cl]_2$ (2.5 mol%), $(^tBuCO)_2O$ (0.75 mmol), toluene (3.0 mL), 140 °C, 12 h.<br>37 examples, 72-96% isolated yield | 134 |

| | | | | | | |
|---|---|---|---|---|---|---|
| 30 | 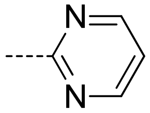 | Bromination | NBS | 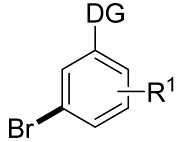 | Substrate (1 equiv, NBS (2 equiv), $[RuCl_2(p\text{-cymene})]_2$ (5 mol%), DMA, 80 °C, 24 h.<br>$R^1$ = H, Me; 3 examples, 84-90%. | 139 |
| 31 | 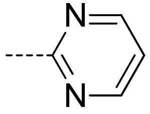 | Carbebnoid insertion | 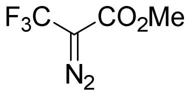<br>$CF_3$-carbenoid | 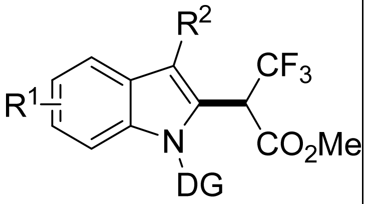 | Substrate (0.2 mmol), reagent (0.24 mmol), $[Cp^*RhCl_2]_2$ (2 mol-%), $AgSbF_6$ (10 mol-%), DCE (2 mL), 80 °C, 4h.<br>$R^1$ = H, $NO_2$, COOMe; $R^2$ = Me, COOMe;<br>5 examples, 68-92% | 143 |
| 32 | 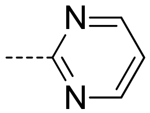 | Carbenoid insertion | 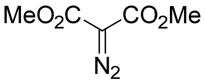 | 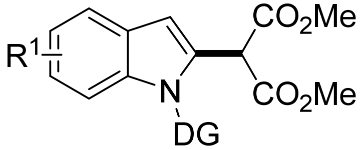 | Substrate (1 equiv), diazo reagent (1.2 equiv), $Cp^*Co(CO)I_2$ (5 mol-%), $AgSbF_6$ (10 mol-%), DCE, 10 °C, 20-48 h.<br>Pyrrole can be used as substrate as well.<br>$R^1$ = H, OMe, Br, OBn, COOMe, CHO, $NO_2$, Cl;<br>14 examples, 24-85% | 144 |
| 33 | 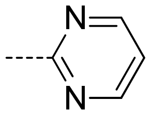 | Cyclopronenone ring opening | 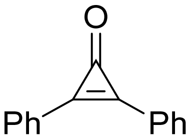 | 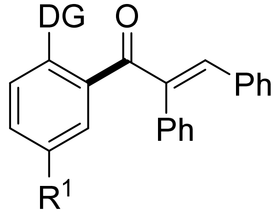 | 2-Arylpyrimidine (0.24 mmol), cyclopropenone (0.2 mmol), $[RhCp^*Cl_2]_2$ (2.5 mol%), $AgSbF_6$(15 mol%),DCM (3 mL), 60 °C, 20 h, sealed tube under argon.<br>$R^1$ = H (82%), Me (85%), COMe (78%); Instead of phenyl 2-thienyl can be functionalized as well (89%). | 138 |
| 34 | 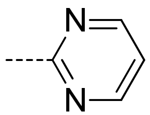 | Heteroarylation | 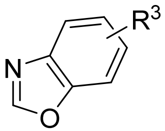 | 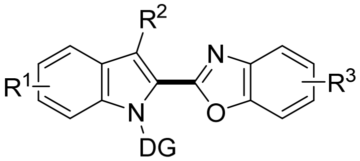 | Substrate (1 equiv), oxazole (2 equiv), $Cu(OAc)_2$ (20 mol%), AcOH (4 equiv), o-xylene, 150 °C, air, 4-6 h.<br>$R^1$ = H, 3-Cl; $R^2$ = H, CN, Cl; $R^3$ = H, $NO_2$, Me; Besides benzoxazoles also 5-aryloxazoles were used as coupling partner; In one case 2-ethylpyrrole was used as substrate;<br>11 examples, 42-81%<br>DG cleavage: NaOMe, DMSO, 100 °C (4 examples, 56-88%). | 140 |

| | | | | | | |
|---|---|---|---|---|---|---|
| 35 | 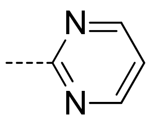 | Heteroarylation | 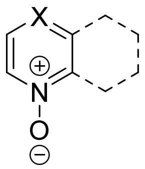 | 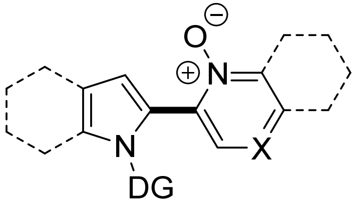 | Indole or pyrrole substrate (1 equiv), N-oxide (4 equiv), $Pd(OAc)_2$ (10-20 mol%), DPPB (10-20 mol%), $Cu(OAc)\cdot H_2O$ (3 equiv), pyridine (2 equiv), 1,4-dioxane, 140 °C, 30 h.<br>Indoles (eventually carrying BnO, MeO) and pyrrole was used as substrate; N-oxides of quinoline, quinoxaline, and pyridine were used; 6 examples, 50-71%<br>DG was cleaved (NaOEt, DMSO, 120 °C) without compromising the N-oxide. | 13 |
| 36 | 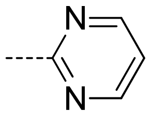 | Imine addition | 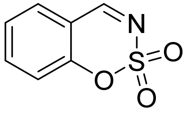 | 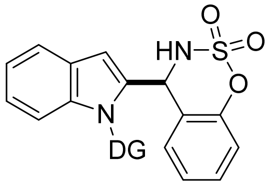 | Substrate (1 equiv), imine (1.08 equiv), $[Cp^*Rh(CH_3CN)_3]SbF_6$ (5 mol%), *t*AmylOH, 85 °C, 16 h;<br>Single example, 74% | 37 |
| 37 | 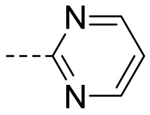 | Indole synthesis | 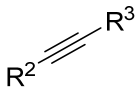 | 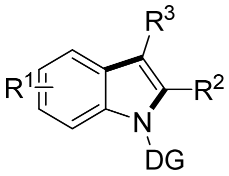 | Substrate (1 equiv), alkyne (3 equiv), $Ni(cod)_2$ (10 mol%), dppf (20 mol%), neat, 160 °C, 20 h.<br>$R^1$ = H, Me, MeO, $CF_3$, Ph, CN, F, Cl; $R^2$ = aryl; $R^3$ = aryl or *t*Bu;<br>20 examples, 55-90%<br>If $R^1$ = 3-F a mixture of 2 regioisomers was isolated. | 141 |
| 38 | 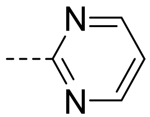 | Nitration | 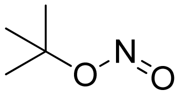 | 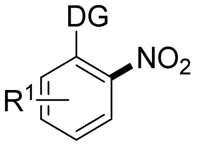 | - Substrate (0.3 mmol), $Pd(OAc)_2$ (10 mol%), TBN (2.0 equiv), PhCl (1 mL), at 80 °C under $O_2$ (1 atm) for 24 h.<br>$R^1$ = 2-Me, 3-Me, 3-MeO; 3 examples, 55-79% | 40 |
| 39 | 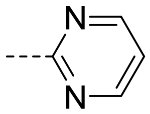 | Selenylation | PhSeSePh | 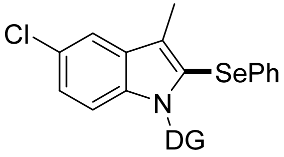 | Substrate (1 equiv), PhSeSePh (1 equiv), $Pd(OAc)_2$ (10 mol%), $CuBr_2$ (2 equiv), DMF, 80 °C, 48 h.<br>Single example, 67% | 38 |

| | | | | | | |
|---|---|---|---|---|---|---|
| 40 | 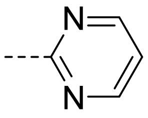 | Selenylation | PhSeSePh | 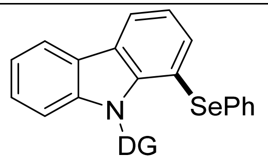 | Substrate (1 equiv), PhSeSePh (1 equiv), $Pd(OAc)_2$ (10 mol%), $CuBr_2$ (2 equiv), DMF, 80 °C, 48 h.<br>Single example, 98% | 38 |
| 41 | 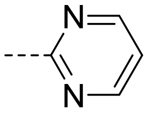 | Sulfenylation | ArSSAr | 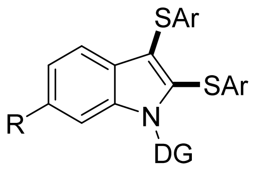 | Substrate (1 equiv), ArSSAr (1 equiv), $Pd(OAc)_2$ (10 mol%), $CuBr_2$ (2 equiv), DMF, 140 °C, 24 h.<br>R = H, Cl; Ar = Ph, 2,5-$Cl_2C_6H_3$; 3 examples 61-69%.<br>For Ar = 4-$MeOC_6H_4$ mono-sulfenylation in position 2 took place; If C3 of indole was blocked by a Me group only C2 sulfenylation took place in 94%. | 38 |
| 42 | 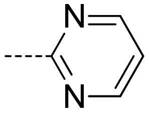 | Trifluoromethyl-allylation | 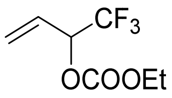 | 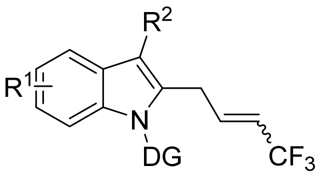 | Substrate (1 equiv), alkene (2 equiv), $[Cp^*RhCl_2]_2$ (2.5 mol%), $AgSbF_6$ (10 mol%), $Cu(OAc)_2$ (50 mol%), THF, air, 120 °C, 24 h<br>$R^1$ = H, OMe, Br, Cl, $NO_2$, Me, F; $R^2$ = H, Me, COOMe;<br>16 examples 66% (16:1 E/Z)<br>Pyrrole is also a substrate for this transformation. | 39 |
| 43 | 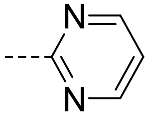 | Cyanation | *t*BuNC | 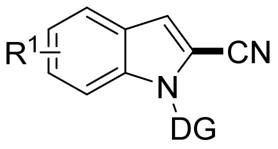 | Substrate (1 equiv), *t*BuNC (3 equiv), $Pd(OAc)_2$ (5 mol%), $Cu(TFA)_2$ (3 equiv), DMF, $O_2$, 130 °C<br>$R^1$ H, OMe, COOMe, Br, Me;<br>7 examples, 33-92%<br>In absence of a DG cyanation takes place in position 3 of indole. | 12 |
| 44 | 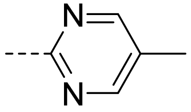 | Alkylation | 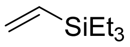 | 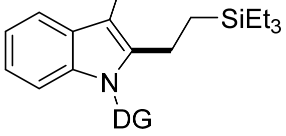 | Substrate (1 equiv), alkene (3 equiv), $[RuCl_2(p\text{-cymene})]_2$ (2.5 - 5 mol%), MesCOOK (30 mol%), toluene, 100 – 120 °C, 18 – 24 h.<br>Single example, 74%. | 24 |

| | | | | | | |
|---|---|---|---|---|---|---|
| 45 | 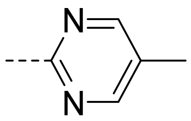 | Amidation | 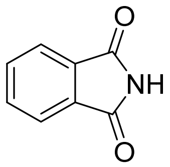 | 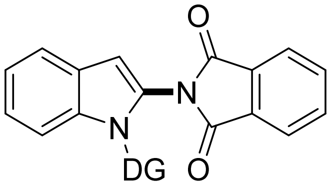 | Substrate (1 equiv), phthalimide (1.2 equiv), CuOAc (20 mol%), toluene/o-dichlorobenzene (1:1), 150 °C, $O_2$, 2-3 days.<br>Single example, 77% ($NO_2$ was not tolerated) | 29 |
| 46 | 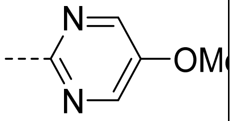 | Nitration | 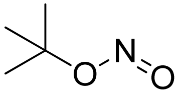 | 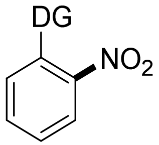 | Substrate (0.3 mmol), $Pd(OAc)_2$ (10 mol%), TBN (2.0 equiv), PhCl (1 mL), at 80 °C under $O_2$ (1 atm) for 24 h.<br>Single example, 78% | 40 |
| 47 | 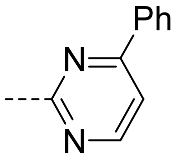 | Cyanation | *t*BuNC | 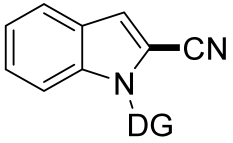 | Substrate (1 equiv), *t*BuNC (3 equiv), $Pd(OAc)_2$ (5 mol%), $Cu(TFA)_2$ (3 equiv), DMF, $O_2$, 130 °C<br>Single examples, 83%<br>In absence of a DG cyanation takes place in position 3 of indole. | 12 |
| 48 | 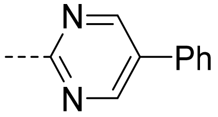 | Cyanation | *t*BuNC | 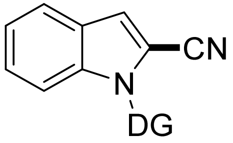 | Substrate (1 equiv), *t*BuNC (3 equiv), $Pd(OAc)_2$ (5 mol%), $Cu(TFA)_2$ (3 equiv), DMF, $O_2$, 130 °C<br>Single examples, 85%<br>In absence of a DG cyanation takes place in position 3 of indole. | 12 |

## Pyrazole derivatives in C-H activation chemistry

Studies which focus on the application of pyrazole as directing group are reaction quite rare. It is much more common that a method is developed for another directing group and then a few examples are given in substrate scope tables showing that also other directing groups can be reacted under certain reaction conditions. It is amongst these "other directing groups" where pyrazole typically finds its place. Hence, the examples given in certain studies are typically only few, which can be seen in the table. Most of the reports have already been discussed in other sections, and hence, here only studies will be discussed in more detail, which have not been discussed elsewhere and in which pyrazole really plays a prominent role.

First of all, in comparison to electron poor heterocycles, pyrazole can be directly functionalized itself under e.g. palladium catalysis[145, 146] and this is a potential side reaction which has to be considered when pyrazole shall be used as DG. Either substituted pyrazoles have to be used which cannot undergo C-H activation anymore, or catalytic systems unable to activate a pyrazole C-H bond have to be found. Hence, typical conditions use Rh, Ru, or Ir but not Pd. Palladium becomes an option when pyrazole cannot be activated, or when this is a desired step, as it is the case in the intramolecular reaction given in Table 4, Entry 20.[147] Here, the reaction consists of actually three C-H activation steps, in which the first one is a pyrazole directed arylation of a C($sp^3$)-H bond of the t-butyl group in position 3 of the pyrazole ring, according to the proposed mechanism. Intramolecular oxidative coupling of the arylated intermediate with position 4 of the pyrazole DG leads to the tricyclic products depicted in entry 5. In this pyrazole activation step, the amide group in position 5 acts as the directing group, so the pyrazole substrate can be considered as a directing group carrying directing group in this specific very elegant cascade of C-H activation steps.

Alkenylations using a Rh or Ru catalyst, an alkyne as coupling partner (leading to alkenes via a hydroarylation pathway), an additive with a non-coordinating anion (e.g. $AgSbF_6$) and a carboxylic acid are quite common and have been reported on a number of occasions. Also *N*-phenylpyrazole was used in such a transformation giving high yields in all prepared examples (Table 4, Entry 1).[148] The alkynes applied were however limited to substituted 1,2-diphenylacetylenes in most cases. When 1-phenyl-1-propyne was used as coupling partner, the new C-C bond was predominantly formed to the C2 position of the alkyne and only minor amounts of isomers were detected.

Phosphoramidation using diphenyl phosphorazidate as coupling partner was reported under iridium catalysis (Table 4, Entry 16).[149] In five examples pyrazole was the DG (typically 2-pyridyl was used) and in all examples yields between 60-69% were obtained. Also in this case it can be expected that a cationic metal complex plays a key role since $[IrCp^*Cl_2]_2$ in combination with $AgSbF_6$ was used. Phosphoramidates are reoccurring motifs in pharmaceuticals making this method interesting for medicinal chemistry applications.

Carbenoid C-H functionalization is an area which has gained some prominence in the last year. Huw Davies pioneered the reaction of a series of saturated carbocyclic and heterocyclic substrate using a chiral Rh catalyst, leading to high ees in most cases.[150-152] These transformations did not require a DG. Directed carbenoid insertion was then first reported by Yu.[153] Using pyrazole as directing group, Osipov and coworkers developed a pyrazole directed protocol (Table 4, Entry 22) which was then expanded to other DGs (ketone methoximes, pyrimidines) in the same contribution.[143] Typical for this type of reactions is the necessity of two electron withdrawing substituents on the

diazo compound, $CF_3$ and COOMe in the present case. In the same year also a Co-catalyzed carbenoid insertion was published using diazo malonates as coupling partners (Table 4, Entry 11).[144]

One of the earliest examples using pyrazole as DG was reported by Chatani and coworkers (Table 4, Entry 12).[154] They used *N*-phenylpyrazole as substrate and developed a carbonylation protocol under neutral conditions using $Ru_3(CO)_{12}$ as catalyst, of CO and ethylene at pressures of 20 atmospheres. Electron donating substituents on the phenyl ring gave significantly better yields than electron withdrawing ones. Also electron rich thiophene could be carbonylated in a reasonable yield of 54%.

Oro and Castarlenas reported the coupling of N-vinylpyrazoles with alkynes to give Markovnikov selective butadienylpyrazole derivatives (Table 4, Entry 23).[155] A rhodium catalyst carrying an NHC ligand proved to be most effective and the reaction worked at relatively mild temperatures of 70 °C. The proposed mechanism, which was supported by isolation of some intermediate Rh-complexes of the catalytic cycle, starts with precoordination of Rh to pyrazole and subsequent activation of the vinyl substituent. Subsequent alkyne coordination, hyrdometallation and reductive elimination delivered the target products.

Miao and Zhang delivered a report in which a pyrazolone directing group played a dual role (Table 4, Entry 24).[156] First, it precoordinated a Rh catalyst allowing C-H insertion in ortho position of the attached phenyl ring. According to the suggested mechanism, the pyrazole N-N bond of the directing group is cleaved, one nitrogen ending up in the indole ring of the final product (red nitrogen Table entry), the other forming the amino group on the side chain. Huang and coworkers reported a similar transformation but using a differently substituted DG as starting material which also leads to differently substituted indole products (Table 4, Entry 25).[78]

**Table 4: Pyrazole as directing group in C-H activation reactions**

| Entry | Directing group | Type of transformation | Coupling partner | Typical product structure | Comments | Ref |
|---|---|---|---|---|---|---|
| 1 | 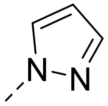 | Alkenylation | 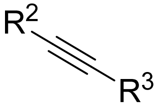 | 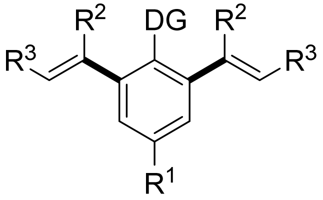 | Substrate (1 equiv), alkyne (2.5 equiv), $[RuCl_2(p\text{-cymene})]_2$ (5 mol%), $AgSbF_6$ (20 mol%), AcOH (4 equiv), 1,4- dioxane, 100 °C, 5 h, $N_2$<br>$R^1$ = H, $CH_3$, MeO, Cl; $R^2$ = aryl, alkyl; $R^3$ = aryl<br>8 examples, 77-89%<br>Amide DGs can enable mono olefination with the same catalytic system | 148 |
| 2 | 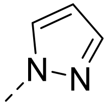 | Alkylation | 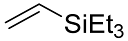 | 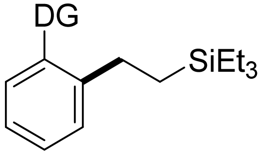 | Substrate (1 equiv), alkene (3 equiv), $[RuCl_2(p\text{-cymene})]_2$ (2.5 - 5 mol%), MesCOOK (30 mol%), toluene, 100 – 120 °C, 18 – 24 h.<br>Single example, 65% | 24 |
| 3 | 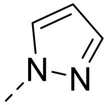 | Alkylation via aziridine opening | 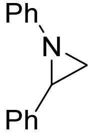 | 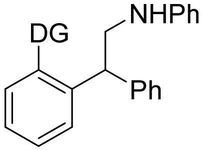 | Substrate (1 equiv), aziridine (2 equiv), $[Cp^*RhCl_2]_2$ (5 mol%), $AgSbF_6$ (30 mol%), PhCl, 100 °C, 20 h.<br>The reaction was developed for 2-arylpyridine derivatives (21 examples, 48-90%)<br>Single example with pyrazole, 58% | 25 |
| 4 | 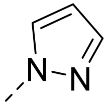 | Alkylation | 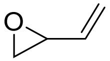 | 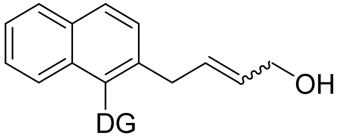 | Substrate (1 equiv), 2-vinyloxirane (1.2 equiv), PivOH (1 equiv), $[Cp^*Rh(MeCN)_3]SbF_6$ (3 mol%), Ar, 25 °C, 16 h<br>Single example, 75% (E/Z = 4.9 : 1) | 26 |
| 5 | 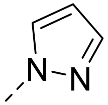 | Alkylation | 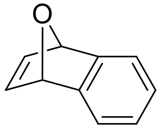 | 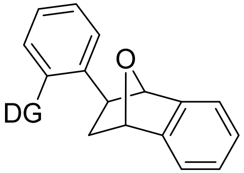 | Substrate (1 equiv), olefine (2 equiv), $[RuCl_2(p\text{-cymene})]_2$ (1 mol%), O2 (1 atm), toluene, 120 °C.<br>The reaction was developed for 2-arylpyridine derivatives (14 examples, 40-94%)<br>Single example on indole, 72%. | 27 |

| | | | | | | |
|---|---|---|---|---|---|---|
| 6 | 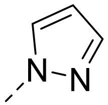 | Alkynylation | 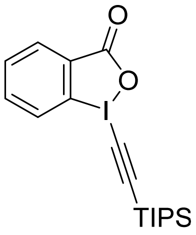 | 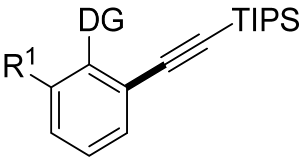 | Substrate (0.2 mmol), R-EBX (0.22 mmol for mono-alkenylation, 0.46 mmol for bis-alkenylation),$[RhCp^*Cl_2]_2$(2 mol%), $Zn(OTf)_2$(0.02 mmol, 10 mol%), DCE (2mL), 25°C, 16 h;<br>One example for mono-alkenylation ($R^1$ = $CH_3$ in the substrate, 78%) and one for bis-alkenylation ($R^1$ = H in the substrate, 91%) | [28] |
| 7 | 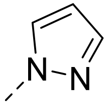 | Amidation | HO-NH-Cbz | 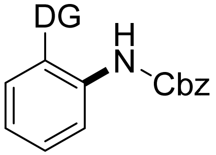 | Substrate (1 equiv), OH-carbamate (1.2 equiv), $[RhCp^*(CH_3CN)_3](SbF_6)_2$ (2.5 mol%), $Ag_2CO_3$ (1.5 equiv), THF, 100 °C, 10h, under air.<br>Single example, 46 % | [142] |
| 8 | 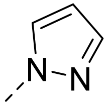 | Arylation | Ar-Br | 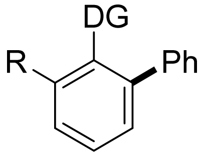 | Substrate (0.5 mmol), PhBr (0.6 mmol), $[RuCl_2(\eta^6\text{-}C_6H_6)]_2$ (0.0125 mmol), $PPh_3$ (0.05mmol), $K_2CO_3$ (1.0 - 2.0 mmol), NMP (1 mL), 120 °C, $N_2$, 20 h.<br>R = Me, 1 example, 92%, R = H: bisarylation occurred (R = Ph), 97%. | [157] |
| 9 | 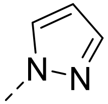 | Arylation | Ph-Cl | 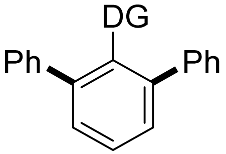 | Phenylpyridine (0.5 mmol), chlorobenzene (1.25 mmol, 2.5 equiv), $[RuCl_2(p\text{-cymene})]_2$ (2.5 mol%), KOPiv (10 mol%), $K_2CO_3$, (3 equiv), DEC (2 mL), 120 °C and 10 h or 80 °C and 24 h<br>Single example, 91% | [158] |
| 10 | 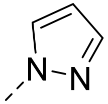 | Arylation | Ar-$B(OH)_2$ | 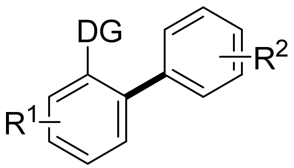 | Substrate (1 equiv), boronic acid (1.2 equiv), $[RuCl_2(p\text{-cymene})]_2$ (2.5 mol%), $PhI(OCOCF_3)_2$ (20 mol%), toluene, 100 °C, 2 h.<br>7 examples, 64-85%<br>Main focus of the paper is the arylation of 2-arylpyridines. | [159] |

| | | | | | | |
|---|---|---|---|---|---|---|
| 11 | 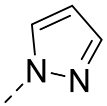 | Carbenoid insertion | 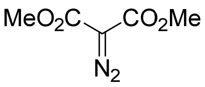 | 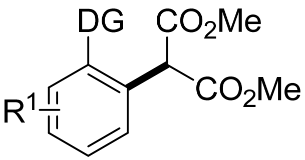 | Substrate (1 equiv), reagent (1.2 equiv), $Cp^*Co(CO)I_2$ (5 mol-%), $AgSbF_6$ (10 mol-%), DCE, 10 °C, 11-48 h.<br>$R^1$ = H, COMe, CN, $CH_2OH$, COOEt, Cl; DG can also be substituted (Me, COMe), 13 examples, 11-80% | 144 |
| 12 | 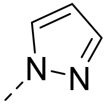 | Carbonylation | CO, $CH_2$=$CH_2$ | 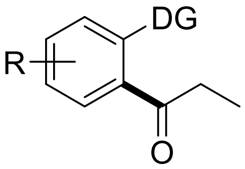 | Substrate (2 mmol), ethylene (7 atm), CO (20 atm), $Ru_3(CO)_{12}$ (0.05 mmol) in DMA (6 mL) at 160 °C for 20 h.<br>9 examples, 31-94%, R = 4-$CH_3$, 4-MeO, 4-$CF_3$, 3-MeO, 3-COOMe, 2-MeO, 2-naphtyl, 3-thienyl | 154 |
| 13 | 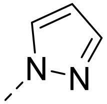 | Chlorination | NCS | 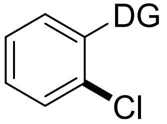 | $Pd(OAc)_2$ (5 mol%), NCS (1.05 equiv), AcOH, 100 °C, 12 h.<br>1 example, 58%. | 160 |
| 14 | 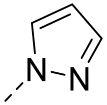 | Cyclopropenone ring opening | 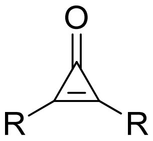 | 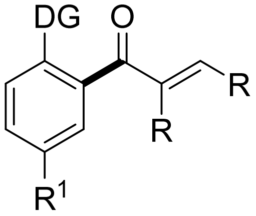 | 2-phenylpyridine (0.24 mmol), cyclopropenone (0.2 mmol), $[RhCp^*Cl_2]_2$ (2.5 mol%), $AgSbF_6$(15 mol%),DCM (3 mL), 60 °C, 20 h, sealed tube under argon.<br>2 examples, $R^1$ = H (72%), COOMe (75%) | 138 |
| 15 | 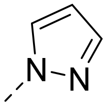 | Nitration | 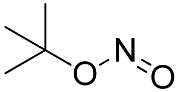 | 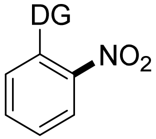 | Substrate (0.3 mmol), $Pd(OAc)_2$ (10 mol%), TBN (2.0 equiv), PhCl (1 mL), at 80 °C under $O_2$ (1 atm) for 24 h.<br>Single example, 61% | 40 |
| 16 | 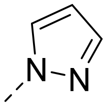 | Phosphoramidation | 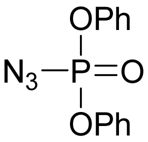 | 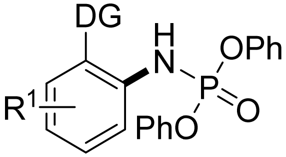 | Substrate (0.1 mmol), 2 (0.2 mmol), $[IrCp^*Cl_2]_2$ (4 mol%), $AgSbF_6$ (16 mol%), AgOAc (50 mol%) and DCM (1.0 mL) under<br>Ar, 24 h, 60 °C.<br>$R^1$ = Me, MeO, Cl; 5 examples, 60 - 69% | 149 |

| | | | | | | |
|---|---|---|---|---|---|---|
| 17 | 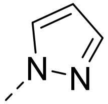 | Silylation | $HSiEt_3$ | 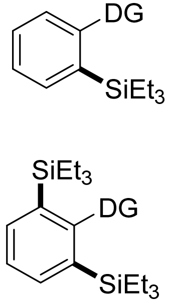<br> | Substrate (1 mmol), $HSiEt_3$ (5 mmol), norbornene (5 mmol), $Ru_3(CO)_{12}$ (6 mol%), toluene (0.5 ml), reflux, 20h<br>Single example, 36% mono silylation, 25% bis silylation | 161 |
| 18 | 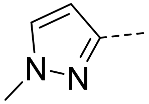 | Silylation | $HSiEt_3$ | 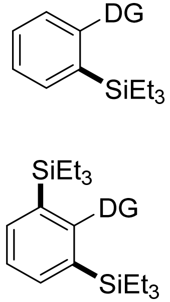<br> | Substrate (1 mmol), $HSiEt_3$ (5 mmol), norbornene (5 mmol), $Ru_3(CO)_{12}$ (6 mol%), toluene (0.5 ml), reflux, 20h<br>Single example, 56% mono silylation, 37% bis silylation | 161 |
| 19 | 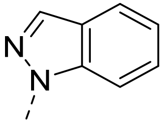 | Arylation | Ar-Br | 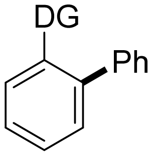<br> | Substrate (0.5 mmol), PhBr (0.6 mmol), $[RuCl_2(\eta^6\text{-}C_6H_6)]_2$ (0.0125 mmol), $PPh_3$ (0.05mmol), $K_2CO_3$ (1.0 - 2.0 mmol), NMP (1 mL), 120 °C, $N_2$, 20 h.<br>1 example, 70% | 157 |
| 20 | 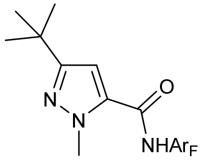<br> | Arylation | Ar-I | 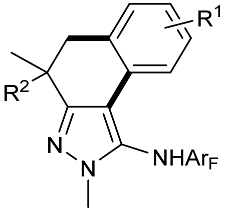<br> | Pyrazole (0.1 mmol), aryl iodide (0.3 mmol), $Pd(OTf)_2(MeCN)_4$ (10 mol%), $Ag_2O$ (0.2 mmol), AcOH (1 mL), 120 °C, 24 h<br>$R^1$ = Me, Halogen, OMe, $PO(OEt)_2$, COOR, $CH_2OAc$; $R^2$ = Alkyl<br>27 examples, 28-83% yield<br>2 directing groups are used for the sequential synthesis of complex pyrazole-derivatives | 147 |

| | | | | | | |
|---|---|---|---|---|---|---|
| 21 | 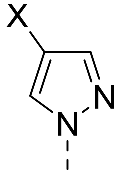 | Bromination | NBS | 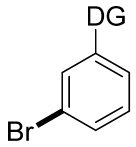 | Substrate (1 equiv, NBS (2 equiv), $[RuCl_2(p\text{-cymene})]_2$ (5 mol%), DMA, 80 °C, 24 h.<br>X = Cl (20%), Br (36%). | 139 |
| 22 | 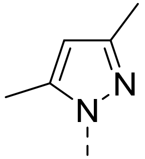 | Carbenoid insertion | 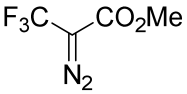 $CF_3$-carbenoid | 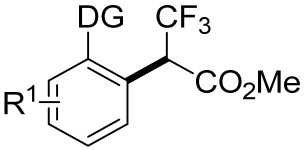 | Substrate (0.2 mmol), reagent (0.24 mmol), $[Cp^*RhCl_2]_2$ (2 mol-%), AgOTf (10 mol-%), DCE (2 mL), 80 °C, 4h.<br>$R^1$ = 4-$CH_3$, 4-MeO, 4-F, H, 5 examples, 93-97% | 143 |
| 23 | 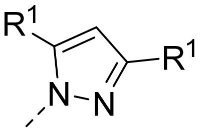 | Hydrovinylation | 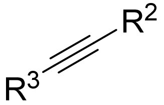 | 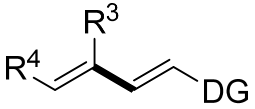 | Substrate (0.2 mmol), alkynes (0.2 mmol), $[Rh(\mu\text{-Cl})(IPr)(\eta^2\text{-coe})]_2$ (0.01 mmol) in $C_6D_6$ (0.5 mL), 70 °C , 2h.<br>The catalytic system is very efficient for the regioselective Markonikov-type head-to-tail dimerization of terminal alkynes to enynes.<br>$R^1$ = H, $CH_3$; $R^2$ = H, alkyl, aryl; $R^3$ = alkyl, aryl; 17 examples, 24-98% | 155 |
| 24 | 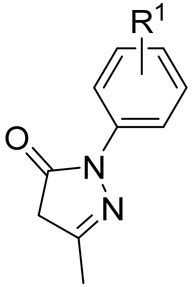 | Indole synthesis | 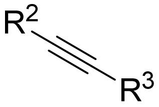 | 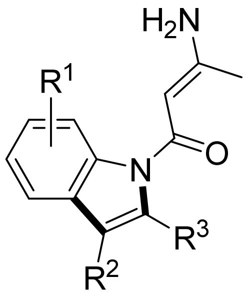 | $[RhCp^*Cl_2]_2$ (2.5 mol%), NaOAc (1 mmol), pyrazolones (0.5 mmol), and alkynes (0.5 mmol) in PhBr (2.5mL) for 2−3 h under 130 °C.<br>1-(3-fluorophenyl)-3-methyl-1*H*-pyrazol-5(4*H*)-one: mixture of regioisomers was observed.<br>Alkyl−alkyl and aryl−alkyl disubstituted alkynes underwent a cyclization process and generated pyrazolo[*1,2-a*]cinnolines and tautomers thereof.<br>23 examples, 30-81% isolated yield<br>$R^1$ = H, $CH_3$, Cl, OMe, Br, F; $R^2$& $R^3$ = Ph, aryl, alkyl | 156 |

| | | | | | | |
|---|---|---|---|---|---|---|
| 25 | 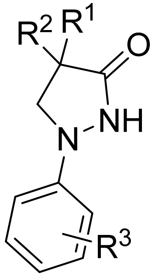 | Indole synthesis | 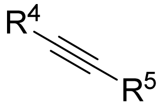 | 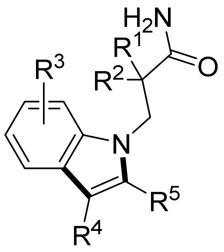 | Substrate (1 equiv), alkyne (1.5 equiv), $[RuCl_2(p\text{-cymene})]_2$ (2.5 mol%), NaOAc (2 equiv), PhCl, 110 °C.<br>$R^1$,$R^2$ = H, Me; $R^3$ = H, Me, F, Cl, Br, CN, $OCF_3$, OMe, $CF_3$, $NO_2$; $R^4$ = Ph, H; $R^5$ = aryl<br>For mono substituted alkynes H always ends up in $R^4$ position.<br>29 examples, 53-94%. | 78 |

## Triazole derivatives in C-H activation chemistry

Even though triazole was used as DG in a silylation reaction already in 2003 (Table 5, Entry 4),[161] it took a while until it was revisited and only in the last three years examples were reported in higher frequency, showing the potential also of this nitrogen heterocycle. Also triazole is potentially a substrate for C-H activation. For example, direct arylation of 4-phenyl-1,2,3-triazole has been reported under Pd catalysis in position 5 of the triazole ring [162-164] which is of course somewhat detrimental for its use as DG, since DG should usually not undergo any side reactions.

Palladium catalyzed acylation using aldehydes as acyl sources have been reported by Kuang and coworkers (Table 5, Entry 1).[165] Triazoles directs the insertion of Pd into the *ortho* C-H bond of the N2-phenyl substituent. The aldehyde is transformed to an acyl radical by reaction with t-butylhydroperoxide, which then gets attached to Pd and after reductive elimination delivers the final products. Both aliphatic and aromatic aldehydes were applied and the transformation showed good functional group tolerance. Using carboxylic acids instead of aldehydes the same group reported also an acyloxylation protocol.

Triazole directed acyloxylation under Pd-catalysis using simple carboxylic acids as coupling partners was reported by the group of Kuang (Table 5, Entry 2).[166] Both, aliphatic and aromatic carboxylic acids were applied and also cinnamic acid derivatives gave high good results.

One of the earliest examples using triazole as DG was reported by Kakiuchi and coworkers (Table 5, Entry 4).[161] The investigated the silylation of arenes carrying different DGs, in one case also 1-methyl-1,2,3-triazole. This $Ru_3(CO)_{12}$ catalyzed transformation used an excess of 5 equivalents $HSiEt_3$ giving a mixture of mono (14%) and bis-silylation products (46%).

The group of Ackermann reported two Ru catalyzed protocols for the direct arylation of arenese directed by 1,2,3-triazole (Table 5, Entry 6).[167] In the first protocol published, the aryl sources which can be applied include aryl bromides, tosylates, and also the cheap and readily available aryl chlorides. However, with triazole directing groups only examples using aryl bromides were disclosed and chlorides and tosylates were only used in combination with oxazoline, pyridine, and pyrazole directing groups. This limitation was soon erased when a slightly modified protocol allowed also the application of aryl chlorides in combination with triazole DGs (Table 5, Entry 7).[168]

Specifically noteworthy is also the iron catalyzed arylation protocol, again developed in the Ackermann lab (Table 5, Entry 8).[169] The attractiveness of iron as catalyst in synthesis does not require any explanation. The catalytic system consisted of simple $FeCl_3$ and dppe as ligand. As aryl source a aryl Grignard reagents were required, which of course leads to certain limitations regarding functional group tolerance. Regarding the substrate scope, C($sp^2$)-H bonds of arenes and alkenes as well as C($sp^3$)-H bonds could be activated. In later work also methylation with MeMgBr was reported using almost the same catalytic system.[170] In this contribution a large diversity of substrates was reacted (arenes, heteroarenes, olefins and even aliphatic ones) and generally high yields were obtained. The need to use Grignard reagents is of course a drawback regarding functional group tolerance. Switching to Ru catalysis, this could be overcome and simple aryl bromides can be applied (Table 5, Entry 10).[171]

Using aryl iodides as aryl source also allowed the development of an C(sp$^3$)-H arylation protocol under Pd-catalysis using a removable triazol based DG (Table 5, Entry 13).[172] The cleavage conditions for the so called TAH group involved heating in presence of $BF_3{\cdot}Et_2O$ in methanol to 100 °C for 10h. Via this protocol, the DG was removed in 86% yield and 0.86g of product was isolated, showing that this can be carried out in gram scale. It has to be mentioned that also the arylation was demonstrated to be scalable and that a stereocenter present in the substrate remained unaffected.

Alkenylations with acrylates are an often applied transformation in C-H activation chemistry. A triazole directed variant has been reported in the group of Shi (Table 5, Entry 12).[173] It is to mention that the DG is relatively remote from the position to be activated. Most importantly, removal of the DG was also demonstrated. Alternative reagents for alkenylations are alkynes, and also here examples with large substrate scope have been reported using triazole as DG.[174]

The group of Shi described two relatively elaborate triazole containing directing groups, which promote actually two different transformations on the same substrate, once a substitution, more specific an acetoxylation reaction (Table, Entry ), and ones an intramolecular cyclization (Table 5, Entry 14).[175] For the acetoxylation, *N*2-pyridine-1,2,3-triazole-4-carboxylic acid is the precursor of the DG, abbreviated TA-Py. The acid functionality is used to attach the substrate to be functionalized by forming an amide bond. Using a system of $Pd(OAc)_2$, $PhI(OAc)_2$, and AgOAc both, C(sp$^2$)-H and C(sp$^3$)-H bonds can be functionalized, the latter requiring somewhat harsher reaction conditions (80 °C sp$^2$ vs 140 °C sp$^3$). Noteworthy, halide substituents in the starting material were well tolerated, which is remarkable in presence of $Pd(OAc)_2$.

In the same contribution N1-aryl-1,2,3-triazole-4-carboxylic acid was introduced as precursor for another DG, abbreviated TAA (Table 5, Entry 15).[175] In this case indoline and azetidine formation was obtained via intramolecular cyclization reactions. Reaction conditions required again of $Pd(OAc)_2$ and $PhI(OAc)_2$ but no AgOAc and halide substituents were once more unaffected and well tolerated. As could be expected, the more strained ring system azetidine required higher temperature for the cyclization to take place (120 °C vs. 80 °C). It is important to note that cleavage of the TAA group has been demonstrated in one example giving indoline in 95% and also the DG precursor was isolated in 82% yield.

So far 1,2,3-triazoles were the DG of choice. Punniyamurthy and coworkers applied also a 1,2,4-triazole derivative as DG in their cupper catalyzed nitration of arenes using simple $Fe(NO_3)_3{\cdot}9H_2O$ as $NO_2$ source (Table 5, Entry 16). [176] Noteworthy, only 35 mol% $Fe(NO_3)_3{\cdot}9H_2O$ were used to get yields >80% in the nitration process indicating that all three $NO_3$ groups are transferable.

**Table 5: Triazole derivatives in C-H activation chemistry**

| Entry | Directing group | Type of transformation | Coupling partner | Typical product structure | Comments | Ref |
|---|---|---|---|---|---|---|
| 1 | 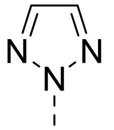 | Acylation | $R^2CHO$ | 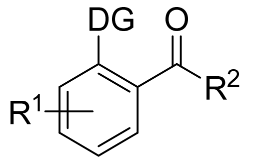 | Substrate (0.4 mmol), aldehyde (0.44 mmol), $Pd(OAc)_2$ (10 mol%), TBHP (0.4 mmol, 70 wt % in water), DCE (2 mL) at 80 °C for 15 h in pressure tubes. $R^1$ = H, Me, Cl, COOMe, OMe; $R^2$ = aryl, alkyl; 26 examples, 25-85% | 165 |
| 2 | 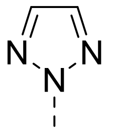 | Acyloxylation | $R^2COOH$ | 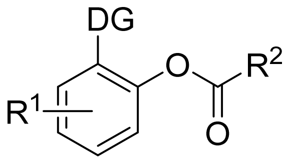 | Substrate (0.4 mmol), carboxylic acid (0.48 mmol), $Pd(OAc)_2$, (10 mol%), $K_2S_2O_8$ (0.8 mmol, 2 equiv), DCE (2 mL) for 20 h in pressure tubes. $R^1$ = H, Cl, COOMe, OMe; $R^2$ = alkyl, aryl, alkenyl; 25 examples, 52-86% The C-H cleavage being the rate-limiting step | 166 |
| 3 | 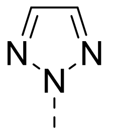 | Arylation | Ar-Br | 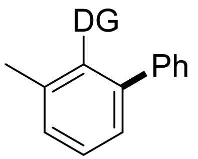 | Substrate (0.5 mmol), PhBr (0.6 mmol), $[RuCl_2(\eta^6\text{-}C_6H_6)]_2$ (0.0125 mmol), $PPh_3$ (0.05mmol), $K_2CO_3$ (1.0 - 2.0 mmol), NMP (1 mL), 120 °C, $N_2$, 20 h. 1 example, 82%. | 157 |
| 4 | 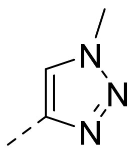 | Silylation | $HSiEt_3$ | 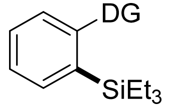 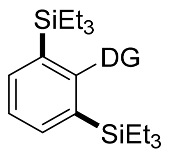 | Substrate (1 mmol), $HSiEt_3$ (5 mmol), norbornene (5 mmol), $Ru_3(CO)_{12}$ (6 mol%), toluene (0.5 ml), reflux, 20h Single example, 14% mono silylation, 46% bis silylation | 161 |

| | | | | | | |
|---|---|---|---|---|---|---|
| 5 | 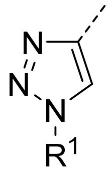 | Alkenylation | 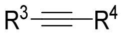 | 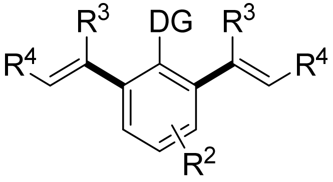 | $[RuCl_2(p\text{-cymene})_2]$ (5 mol %), $Cu(OAc)_2 \cdot H_2O$ (20 mol%), $AgSbF_6$ (20 mol%), toluene, 100 °C, 2.5 h<br>$R^1$ = Bn, *p-subst*-Bn, undecal, cyclohexyl, $CH_3CHPh$, $(CH_3)_2CPh$; $R^2$ = H, Me, Cl, F, $CF_3$, $NO_2$, OMe; in most cases $R^3$ = $R^4$ = Ph resp. 4 subst. phenyl (Me, OMe, Cl, $CF_3$); 2 examples for $R^3$ = alkyl (Me or *n*hex) and $R^4$ = Ph; 25 examples, 52-91% | 174 |
| 6 | 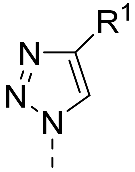 | Arylation | 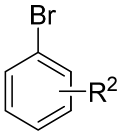 | 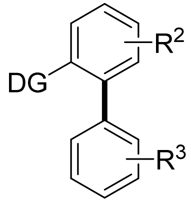 | $[RuCl_2(p\text{-cymene})_2]$ (2.5 mol %), MesCOOH (30 mol %), toluene, $K_2CO_3$, 120 °C, 16-20h<br>$R^1$ = Bu, $CH_2TMS$, hexyl; $R^2$ = 2-Me, 2-MeO; $R^3$ = Cl, Me, OMe, COPh, COMe, COOEt; 8 examples, 63-97%; | 167 |
| 7 | 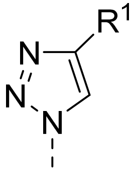 | Arylation | 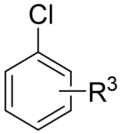 | 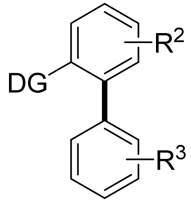 | $[RuCl_2(p\text{-cymene})_2]$ (2.5 mol %), $PCy_3$ (10 mol %), NMP, $K_2CO_3$, 120-135 °C, 20h<br>$R^1$ = Bu, $CH_2TMS$, hexyl; $R^2$ = 2-Me, 3-Me, 2-MeO; $R^3$ = OMe, COPh, COOR, OTs; 12 examples, 50-94%;<br>In 4-chlorobromobenzene the bromine reacted exclusively. | 168 |
| 8 | 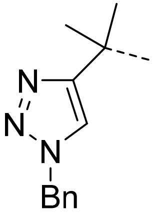<br>(TAM) | Arylation | 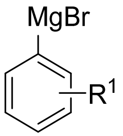 | 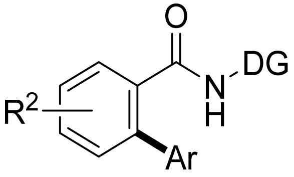 | Substrate (0.3 mmol), ArMgBr (2.1 mmol), $FeCl_3$ (10 mol-%), dppe (10 mol-%), $ZnBr_2$.TMEDA (3 equiv), DCIB (2 equiv), THF (1 mL), 55 °C.<br>$R^1$ = H, Me, F, MeO; $R^2$ = H, Me, MeO, F, Et, Ph;<br>21 examples, 51-93%<br>DG removal in aq. HCl. | 169 |
| 9 | 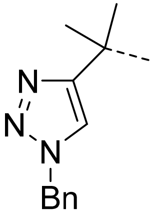 | Arylation | 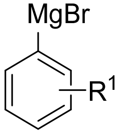 | 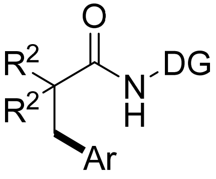 | Substrate (0.2 mmol), ArMgBr (1.4 mmol), $FeCl_3$ (20 mol-%), dppbz (20 mol-%), $ZnBr_2$.TMEDA (3 equiv), DCIB (2 equiv), toluene (1 mL), 80 °C.<br>$R^1$ = H, Me, F, MeO; $R^2$ = Me, cyclohexyl; | 169 |

| | | | | | | |
|---|---|---|---|---|---|---|
| | (TAM) | | | | 9 examples, 52-87% | |
| 10 | 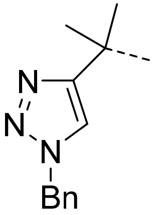<br>(TAM) | Arylation | ArBr | 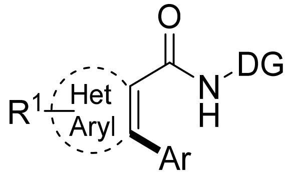 | Substrate (1 equiv), ArBr (1.2 equiv), $Na_2CO_3$ (1.5 equiv), $[RuCl_2(PPh_3)_3]$(5 mol%), *o*-xylene (0.25 M), 22 h, 120–140 °C.<br>Reaction might involve SET-type process.<br>$R^1$ includes H, Me, OMe, $CF_3$, Ph, Cl, F<br>25 examples, 11-99% | [171] |
| 11 | 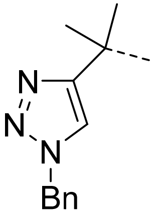<br>(TAM) | Methylation | MeMgBr | 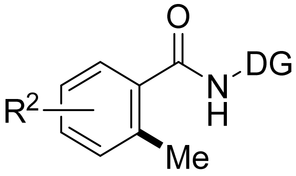 | Substrate (1 equiv), MeMgBr (7 equiv), $FeCl_3$ (20 mol-%), dppe (20 mol-%), $ZnCl_2$.TMEDA (3 equiv), DCIB (2 equiv), THF, 25-55 °C.<br>Functional group tolerance limited as expected for a large excess of Grignard reagent ($R^2$ = Me, OMe); Substrates structurally quite diverse; also heterocyclic substrates applied; also olefins and alkanes instead of arene substrates; 26 examples, 49-99%. | [170] |
| 12 | 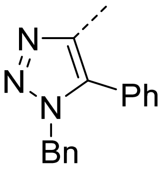<br>TA(Ph) | Alkenylation | 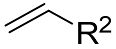 | 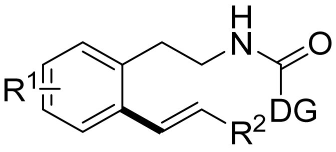 | Substrate (0.2 mmol), alkene (0.8 mmol), HOAc (5.0 mmol), $Pd(OAc)_2$ (7.5-15 mol%), $Cu(OTf)_2$ (10 mol%), 105-115 °C, $O_2$ balloon or 1 atm of $O_2$ in sealed tube, 1,4-dioxane (1.0mL).<br>A primary KIE was observed (KH/KD=2.1) suggesting rather a C−H activation than aLewis acid catalyzed Friedel−Crafts-type mechanism<br>Removal of the Dg using $Boc_2O$ then $LiOH/H_2O_2$ furnished the Boc protected amines.<br>$R^1$ = H, Me, OMe, F, Br; $R^2$ = COOBu, CN, Me, $NMe_2$, $PO(OEt)_2$, Ph, $SO_2Ph$, aryl; 25 examples, 78-95%; in some | [173] |

| | | | | | | |
|---|---|---|---|---|---|---|
| | | | | | cases mixtures of mono and bis alkenylated products were obtained | |
| 13 | 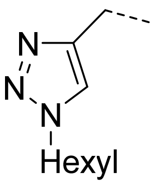 (TAH) | Arylation | 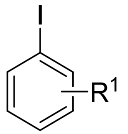 | 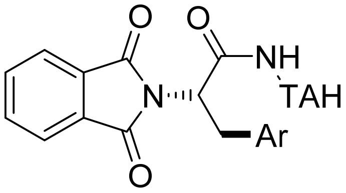 | Substrate (0.40 mmol), $Pd(OAc)_2$ (10.0 mol%), ArI (0.60 mmol, 1.5 equiv), AgOAc (0.60 mmol, 1.5 equiv), HFIP (2 mL), 100 °C, 5 h<br>$R^1$ = MeO, F, Cl, Br, $NO_2$, Me, $CF_3$, COOMe, Ph, COEt; 16 examples, 68-90%<br>DG removal: use $BF_3.Et_2O$, MeOH, 100 °C, 10h | 172 |
| 14 | 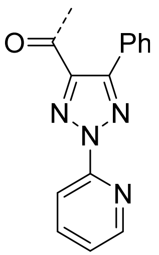 TA-Py | Acetoxylation | $PhI(OAc)_2$ | 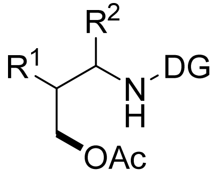 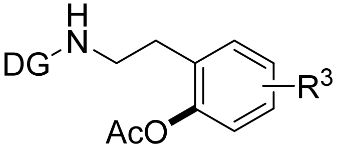 | $C(sp^3)$-H activation: $Pd(OAc)_2$ (10 mol%), $PhI(OAc)_2$ (3.0 equiv), AgOAc (1.5 equiv) in DCE, Ar atmosphere, 140 °C, 24h.<br>$R^1$ = COOMe, $CH_2OAc$; $R^2$ = Me, Et; 4 examples, 58-76%<br>$C(sp^2)$-H activation: $Pd(OAc)_2$ (10 mol%), $PhI(OAc)_2$ (2.5 – 3.0 equiv), AgOAc (0.5 equiv) in DCE, Ar atmosphere, 80 °C, 24h.<br>$R^3$ = H, Me, Br, F, OMe, I; 15 examples 60-85%<br>For both activation types mixtures of mono and bis substitution are sometimes formed. | 175 |
| 15 | 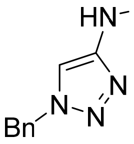 (TAA) | Cyclization | - | 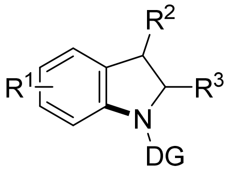 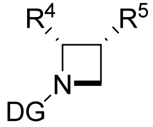 | Indoline formation: $Pd(OAc)_2$ (5 mol%), $PhI(OAc)_2$ (2.0 equiv), in DCE, Ar atmosphere, 80 °C, 24h.<br>$R^1$ = F, Br, I, Me, MeO; $R^2$ = H, Me; $R^3$ = H, COOMe; 12 examples, 53-85%<br>Azetidine formation: $Pd(OAc)_2$ (5 mol%), $PhI(OAc)_2$ (2.5 equiv), in DCE, Ar atmosphere, 120 °C, 24h.<br>$R^4$ = COOMe, $CH_2OAc$, Me; $R^5$ = Me, Et;<br>4 examples, 56-88% | 175 |

| | | | | | | |
|---|---|---|---|---|---|---|
| 16 | 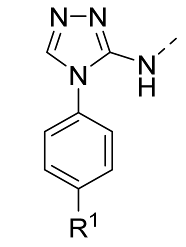 | Nitration | $Fe(NO_3)_3 \cdot 9H_2O$ | 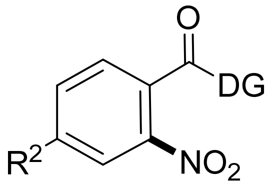 | Substrate (1 mmol), $CuCl_2 \cdot 2H_2O$ (20 mol%), $Fe(NO_3)_3 \cdot 9H_2O$ (35 mol%), DCE (3 mL), rt.<br>$R^1 = R^2$ = H, F, Me; 3 examples, 82-87% | 176 |

## Tetrazole derivatives in C-H activation chemistry

Not surprisingly also tetrazoles are potential DGs. In case they are attached to the substrate via the remaining carbon of the ring, a competitive C-H activation of the DG is not an issue. Still, only few examples have been reported so far. Tetrazoles are of increasing importance in medicinal chemistry due to the fact that they are considered as bioisosters of carboxylic acids.[177-181] Hence, it can be expected that the amount of examples using tetrazoles as DGs will be increasing in the near future. One of the earliest examples using tetrazoles as DGs was reported by Kakiuchi and coworkers.[161] They investigated the silylation of arenes carrying different DGs, in two cases differently methylated (N1 and N2) tetrazoles. This $Ru_3(CO)_{12}$ catalyzed transformation used an excess of 5 equivalents $HSiEt_3$ giving a mixture of mono (22%) and bis-silylation products (71%) in case of N2 methylated tetrazole but only the mono-silylated product in case of N1 metylated tetrazole. Eventually, the DG cannot rotate around the C-C bond to present the $sp^2$ nitrogen to the second ortho position due to steric hindrance between the N1 methyl group and the bulky $SiEt_3$ group. This is actually a general finding since several other examples with this directing group show a selective mono substitution (Table 6, Entries 4-6) for examples where other DGs give also bis-substitution.

Oxidative alkenylations with acrylates [182] (and in one case styrene) and hydroarylative alkenylations with alkynes[183] have been reported using the same Rh species, namely $[RhCp^*(MeCN)_3]SbF_6$ (Table 6, Entries 1 & 2). Both transformations show a broad functional group tolerance, including reactive halides such as Br, important for further elaboration of the products.

The group of Seki has reported tetrazole directed ortho arylation due to their interest of efficient synthesis of angiotensin II receptor blockers (Table 6, Entry 11).[184-187] They explored various N1 substituted tetrazoles under Ru catalysis using bromobenzenes as the aryl source. However, the substrate scope regarding the aryl source was not fully explored and seems to be quite limited.

A much more comprehensive study regarding the aryl bromide substrate scope was reported by Ackermann and coworkers (Table 6, Entry 9) [188]. A bulky carboxylic acid as additive improved the yield significantly. Importantly, also two heterocyclic aryl bromides were successfully coupled. 2-Bromothiophene gave a good yield of product of 63%, whereas 3-bromopyridine gave only 30% yield. due to the N1-benzyl group in the DG, only mono-arylation products were observed.

An interesting quinolone synthesis has been reported by Hua and coworkers (Table 6, Entry 13)[189]. Initially, N1-aryl tetrazoles undergo a cyclization with internal alkynes under Rh-catalysis. The so obtained intermediate fragments and eliminates $N_2$ mediated by $Cu(OAc)_2.H_2O$ (Scheme 4). Interestingly, water free $Cu(OAc)_2$ gives only low yields of the quinolones whereas the mono hydrate gives almost quantitative conversion. In most cases symmetrical diarylalkynes were used. In cases were mixed alkyl-aryl alkynes were used, the alkyl residue ended up in $R^3$ position with high regioselectivity.

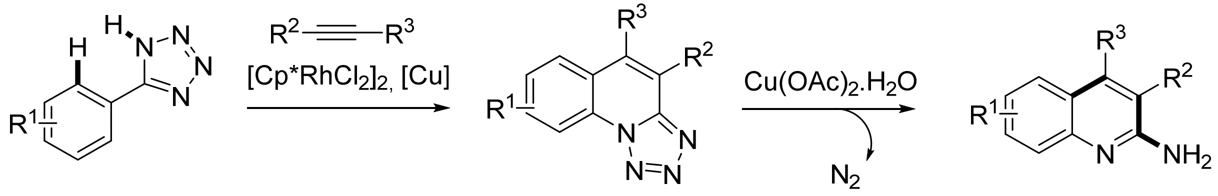


**Scheme 4: Tetrazole directed annulation/$N_2$ extrusion towards 2-aminoquinolines.**

A convenient nitration protocol was reported by Punniyamurthy and coworkers (Table 6, Entry 12).[176] As nitrating agent $Fe(NO_3)_3 \cdot 9H_2O$ was used and cheap $CuCl_2$ could be used as catalyst. Such methods are extremely interesting since the ortho directing group, 1-aryl tetrazoles in this case, can override the directing effects substituents already present in the starting material might have. Overall, 21 examples with high yields have been reported. Since the DG is attached via an NH linker to the substrate, it could also be cleaved afterwards, giving 2-nitro substituted anilines. In one case the transformation was also carried out in gram scale with no decreased yield.

**Table 6: Tetrazole derivatives as directing groups in C-H activation reactions**

| Entry | Directing group | Type of transformation | Coupling partner | Typical product structure | Comments | Ref |
|---|---|---|---|---|---|---|
| 1 | 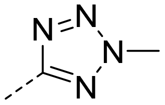 | Alkenylation | 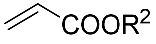 | 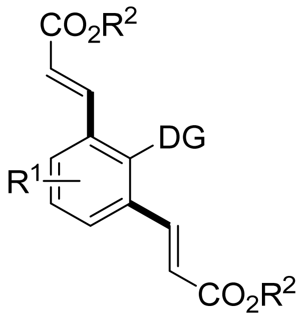 | Substrate (0.1 mmol), acrylates (0.2 mmol), $[RhCp^*(MeCN)_3][SbF_6]_2$ (5 mol-%), $Cu(OAc)_2$ (2 equiv), dioxane (1.5 mL), 110 °C, air, 12 h.<br>Works with styrene but not acrylnitrile.<br>C–H bond cleavage was the rate-determining step<br>$R^1$ = H, Me, OMe, Br, F, $CF_3$, CN, $NO_2$, Cl; $R^2$ = Me, Et, *t*-Bu;<br>16 examples, 22-95% | 182 |
| 2 | 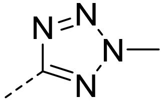 | Alkenylation | 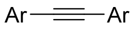 | 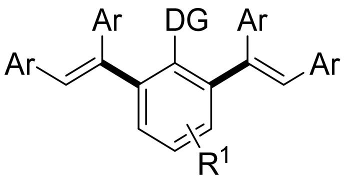 | Substrate (0.2 mmol), reagent (0.6 mmol), $[RhCp^*(MeCN)_3][SbF_6]_2$ (2 mol%), PhCOOH (0.05 mmol), HOAc (2 mL), 80 °C<br>Ar = Ph, 4-$MeC_6H_4$, 4-MeO $C_6H_4$, 4-Br $C_6H_4$, 4-Cl $C_6H_4$, 4-$FC_6H_4$, $R^1$ = H, Me, MeO, Br, F, $CF_3$, COOH, Cl;<br>16 examples, 57-94%. | 183 |
| 3 | 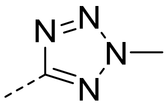 | Silylation | $HSiEt_3$ | 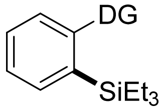<br>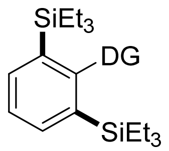 | Substrate (1 mmol), $HSiEt_3$ (5 mmol), norbornene (5 mmol), $Ru_3(CO)_{12}$ (6 mol%), toluene (0.5 ml), reflux, 20h<br>Single example, 22% mono silylation, 71% bis silylation | 161 |
| 4 | 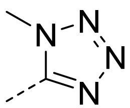 | Alkenylation | 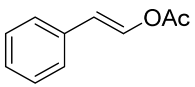 | 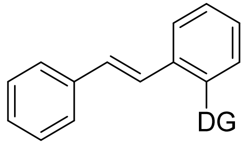 | Substrate (1 mmol), alkene source (3 mmol), 3 (0.05mmol), toluene (1.5 mL), reflux, isolated yield.<br>Mono selective, single example, 52% | 190 |

| | | | | | | |
|---|---|---|---|---|---|---|
| 5 | 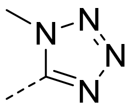 | Arylation | PhBr | 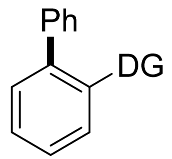 | Substrate (0.5 mmol), PhBr (0.6 mmol), $[RuCl_2(\eta^6\text{-}C_6H_6)]_2$ (0.0125 mmol), $PPh_3$ (0.05mmol), $K_2CO_3$ (1.0 - 2.0 mmol), NMP (1 mL), 120 °C, $N_2$, 20 h.<br>Mono selective, single example, 43% yield | 157 |
| 6 | 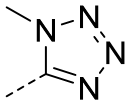 | Iodination | NIS | 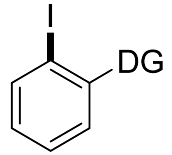 | $Pd(OAc)_2$ (5 mol%), NIS (1.05–2.1 equiv), 100–120 °C, 12 h, MeCN or AcOH.<br>Mono selective, single example, 41% | 160 |
| 7 | 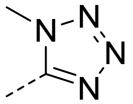 | Silylation | $HSiEt_3$ | 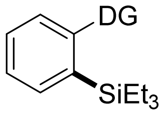 | Substrate (1 mmol), $HSiEt_3$ (5 mmol), norbornene (5 mmol), $Ru_3(CO)_{12}$ (6 mol%), toluene (0.5 ml), reflux, 20h<br>Single example, 70% mono silylation | 161 |
| 8 | 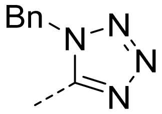 | Arylation | ArI | 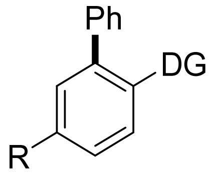 | Substrate (0.2 mmol, 1.0 equiv), reagent (10.0 equiv), $Pd(OAc)_2$ (5 mol%), AgOAc(4.0 equiv), TFA (0.2 mL), 150°C, 24h<br>R = H, Me, MeO, F, Cl, Br; 6 examples, 43-85% | 191 |
| 9 | 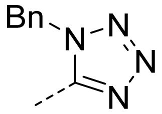 | Arylation | ArBr | 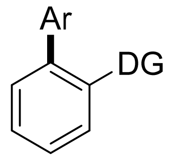 | Substrate (1 equiv), ArBr (1.1 equiv), $[RuCl_2($*p-cymene*$)]_2$ (5 mol%), MesCOOH (30 mol%), $K_2CO_3$ (2 equiv), toluene, 120 °C, 18 h;<br>Selective mono arylation; also heterocyclic bromides could be applied; ArBr carrying ketones, esters, methyl, methoxy, and fluorine were reported; 21 examples, 30-70%. | 188 |
| 10 | 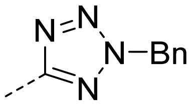 | Arylation | PhI | 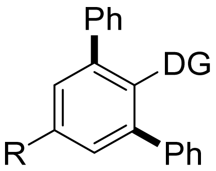 | Substrate (0.2 mmol, 1.0 equiv), reagent (10.0 equiv), $Pd(OAc)_2$ (5 mol%), AgOAc(4.0 equiv), TFA (0.2 mL), 150°C, 24h<br>R = H, Me, OMe, F, Cl, Br; 6 examples, 65-96%.<br>Substrates carrying already a substituent in *ortho* or *meta* position gave high yields of mono arylation: 18 examples, | 191 |

| | | | | | | |
|---|---|---|---|---|---|---|
| | | | | | 59-97%.<br>Also 1-Bn-tetrazole used as DG, then selective mono arylation, same 6 examples 43-88% yield. | |
| 11 | 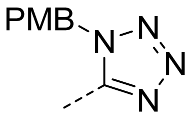 | Arylation | 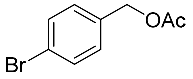 | 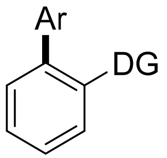 | Substrate (1 equiv), ArBr (1.1. equiv), $K_2CO_3$ (2 equiv), $RuCl_3xH_2O$ (10 mol%), $PPh_3$ (18 mol%), 140 °C, 12 h<br>81% of single example | 187 |
| 12 | 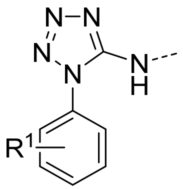 | Nitration | $Fe(NO_3)_3\cdot 9H_2O$ | 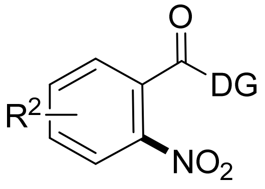 | Substrate (1 mmol), $CuCl_2\cdot 2H_2O$ (20 mol%), $Fe(NO_3)_3\cdot 9H_2O$ (35 mol%), DCE (3 mL), rt.<br>Reaction was scaled up to gram scale<br>DG removal: 7 equiv NaOH in dioxane, 110 °C, 12-22 h.<br>Substrate-binding step is the product-determining step.<br>TEMPO does not inhibit the reaction.<br>$R^1$ = H, Me, Cl, F, OMe, iPr, Et; $R^2$ = H, F, *i*Pr, naphtyl, $NO_2$, Et, Me, CN, NHAc; 22 examples, 77-95% | 176 |
| 13 | 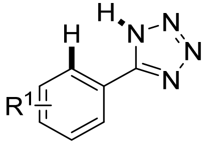 | Quinoline synthesis | 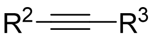 | 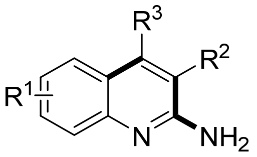 | Substrate (2.5 equiv), alkyne (1 equiv), $[Cp^*RhCl_2]_2$ (2.5 mol%), $Cu(OAc)_2.H_2O$ (2 equiv), KOAc (2 equiv), DMAc, 130 °C, $N_2$, 6 h<br>$R^1$ = H, Me, MeO, Et, *i*Pr, *t*Bu, F, Cl, COOMe, $COCF_3$; $R^2$ = $R^3$ in most cases and aryl, four alkyl-aryl akynes were included and high regioselectivity was observed whereas $R^3$ = alkyl, $R^2$ = aryl. 24 examples overall, 46-79%. | 189 |

## Oxazole based directing groups

Oxazole itself is rarely applied as DG since its C2 and C5 position can be readily C-H activated themselves.[192] The much more frequently applied variant is oxazoline, typically attached via C2 to the substrate to be activated. Even though not shown in many contributions, oxazoline is a cleavable DG since it can be hydrolyzed to a carboxylic acid,[193-195] which in term can be further transformed into other functional groups or eventually removed by decarboxylation. From this point of view, oxazoline is a very attractive DG. The basic $sp^2$ hybridized nitrogen allows similar transformations as with other *N*-heterocycles such as pyridine, pyrimidine, pyrazole, and so on. Not surprising, many contributions which are dedicated to either of these other N-heterocyclic DGs, also show one or the other example in which it is demonstrated that the developed methodology also tolerates oxazoline as a DG. The discussion of examples in this section is however focused on examples which have oxazole derivatives as the main focal point in their research.

Oxazoline was very early established as suitable DG in C-H activation chemistry. Murai and coworkers established $Ru_3(CO)_{12}$ catalyzed carbonylation using CO and an olefine (in most cases ethylene) as coupling partner (Table 7, Entry 17).[196] This is one of the pioneering early examples of C-H activation chemistry. If two ortho positions on the arene substrates were available, mixtures of mono and bis substituted products were obtained. Blocking one ortho position naturally led to selective reactions. Even in presence of one *meta* substituent, the two remaining ortho positions were significantly different in reactivity and carbonylation occurred mainly at the sterically less compromised side, eventually accompanied by bis-carbonylated products. When higher olefins were used, mixtures of linear and branched ketone products were obtained. This study focused on oxazoline, however also several other DGs were reported, amongst them oxazole itself (Table 7, Entry 1), which proved to be significantly less efficient.

Kakiuchi reported $Ru_3(CO)_{12}$ catalyzed silylation with a series of DGs (Table 7, Entry 18).[161] As starting point in their development served 4,4-dimethyloxazoline and high yields of the mono-silylation products were obtained. Twofold silylation, which was often an issue with other DGs was never an issue in the oxazoline directed case.

An interesting alkylation using tetraalkyl tin reagents was reported by the group of Yu (Table 7, Entry 12).[197] It turned out that batch wise addition of the organotin reagent over prolonged reaction times (up to 60h) was necessary in order to get good yields. The reactions could be accelerated significantly when carried out in the microwave.

Nishimura reported a branch-selective alkylation of arenes with vinyl ethers (or hydroarylation of vinyl ethers) using an iridium catalyst (Table 7, Entry 13).[198] Even though only one example with an oxazoline DG has been reported (majority of examples on 2-phenylpyridines), it is worth mentioning since usually only linear alkylation products are obtained.

Alkylation and arylation with alkyl or aryl iodides has been reported by the group of Liu (Table 7, Entries 33 & 34).[199] In this case, an isoxazole containing directing group was used. Remarkable is the functional group tolerance, which was especially demonstrated for the arylation case. Here, it was shown that halides were well tolerated and also a boronic ester, nitro and an azide group gave high yields.

Shi and coworkers reported the remote activation and arylation with aryl iodides of γ-methylene $C(sp^3)$-H bonds (Table 7, Entry 15) and δ-$C(sp^2)$-H bonds (Table 7, Entry 16) using an amide linked oxazoline directing group.[200] A remarkably large functional group tolerance was reported. Additionally, it is a rare example in which cleavage of the oxazoline DG was really tested and successfully carried out.

Bidentate amino oxazoline directing groups, chiral and achiral ones, have been used for the direct arylation of secondary C-H bonds by the group of Shi (Table 7, Entry 24). [201] Reaction optimization started with $Pd(OAc)_2$ as catalyst and carboxylic acids as additives, a typical combination to start a screening in the field. Interestingly, dibenzyl phosphate ($(BnO)_2PO_2H$) proved to be more affective, in the end in combination with $Pd(OPiv)_2$ as palladium source. Iodo arenes had to be used as aryl source and it was shown that many functional groups were well tolerated. Only sterically demanding 2-iodotoluene gave no conversion. Also $C(sp^2)$-H activation was tried using this protocol but no conversion was observed. Additionally, a chiral variant of the DG was applied and three examples with d.r. of 88:12 – 90:10 were reported (Table 7, Entry 25). Alternative aryl sources have been applied as well, e.g. aryl tosylates (Table 7, Entry 6)[202] and aryl bromides (Table 7, Entry 8).[203]

Asymmetric iodination was reported by the group of Yu under mild conditions (Table 7, Entry 23).[204] Four examples with a chiral DG were reported giving d.r. between 91:9 and as high as 99:1. Naturally, the reaction was also carried out in a racemic fashion as well, with typically high yields of up to 97%. In subsequent years the method was further explored in a series of papers[205, 206]and also detailed mechanistic investigations were reported.[207]. The group of Sanford also reported a single example for an isoxazoline directed iodination, this time using NIS as iodination reagent (Table 7, Entry 35).[160]

A copper catalyzed method for the coupling of amides with malonates was reported by Dai and Yu (Table 7, Entry 26).[208] The initial C-H activation and C-C coupling reaction with malonates was followed by an intramolecular oxidative C-N bond formation, ultimately leading to isoindolin-1-ones. The functional group tolerance was good, yields however were often only around 50%.

Besides oxazoline, also benzoxazole can be used as DG since in that case positions 4 and 5 are blocked due to annulation and C2 is typically used to attach the substrate. A comprehensive study of the ortho acylation of 2-arylbenzoxazoles has been reported by the group of Yang and Wu (Table 7, Entry 29-31).[209] Aldehydes were applied as readily available acyl source, which are transformed to acyl radicals by action of the organic oxidant TBHP. Only aromatic aldehydes were applied and in most cases and a large excess of 6 equivalents was required.

Amidation using 3-substituted 1,4,2-dioxazol-5-ones as amide source has been reported by Ackermann and coworkers under Co-catalysis using several different DGs, amongst them oxazoline and 5,6-dihydro-4H-1,3-oxazine (Table 7, Entries 5, 20-22, 36).[15] Such a method leads to regioisomeric amides as compared to the isocyanate method established by Shibata (Table 7, Entry 19)[210].

**Table 7: Oxazole based and related directing groups in C-H activation chemistry**

| Entry | Directing group | Type of transformation | Coupling partner | Typical product structure | Comments | Ref |
|---|---|---|---|---|---|---|
| 1 | 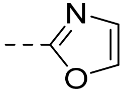 | Carbonylation | CO, $CH_2=CH_2$ | 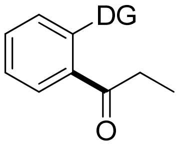 | 5 mol% $Ru_3(CO)_{12}$, ethylene, CO, 20 atm, toluene, 160 °C, 40h<br>Single example, 36% | 196 |
| 2 | 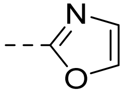 | Arylation | Ar-Br | 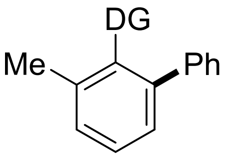 | Substrate (0.5 mmol), PhBr (0.6 mmol), $[RuCl_2(\eta^6\text{-}C_6H_6)]_2$ (0.0125 mmol), $PPh_3$ (0.05mmol), $K_2CO_3$ (1.0 - 2.0 mmol), NMP (1 mL), 120 °C, $N_2$, 20 h.<br>Single example, 66% | 157 |
| 3 | 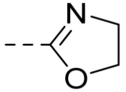 | Alkenylation | 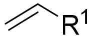 | 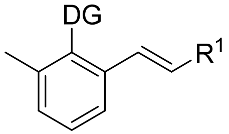 | Substrate (1 equiv), alkene (1.3 equiv), $[RuCl_2(p\text{-cymene})]_2$ (5 mol%), 1,1´-binaphthyl-2,2´-diyl hydrogenphosphate (10 mol%), $Cu(OAc)_2.H_2O$ (0.8 equiv), EtOH (2 mL), 80 °C under air.<br>$R^1$ = various esters, alkyls and aryls, amides; 10 examples, 15-80% | 211 |
| 4 | 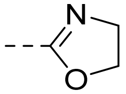 | Alkynylation | 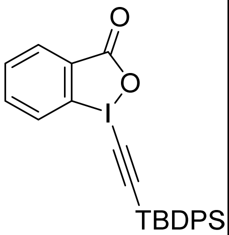 | 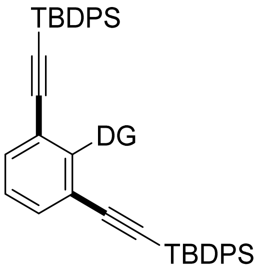 | Substrate (0.2 mmol), R-EBX (0.46 mmol), $[RhCp^*Cl_2]_2$ (2 mol%), $Zn(OTf)_2$ (0.02 mmol, 10 mol%), DCE (2mL), 25 °C, 16 h;<br>1 example, 73% | 28 |
| 5 | 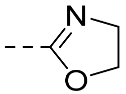 | Amidation | 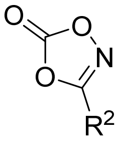 | 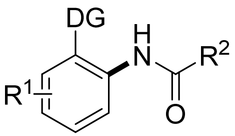 | Substrate (1 equiv), dioxazolone (1.2 equiv), $Cp^*Co(CO)I_2$ (5 mol%), $AgSbF_6$ (20 mol%), NaOAc (20 mol%), DCE, 100 °C, 20 h.<br>$R^1$ = H, Me, Et, $_i$Pr, OMe,O$_t$Bu, Ph, $CF_3$, F, Cl, Br, $CO_2Me$, NHAc, Cl; $R^2$ = Ph, 3-F-$C_6H_4$; 18 examples, 50-75% | 15 |

| | | | | | | |
|---|---|---|---|---|---|---|
| 6 | 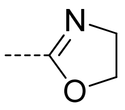 | Arylation | Ar-OTs | 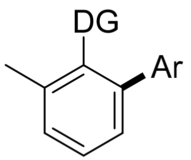 | Substrate 1 equiv, Ar-OTs (1.2 equiv), $[RuCl_2(p\text{-cymene})]_2$ (2.5 mol%), ligand (10 mol%), $K_2CO_3$, NMP, 120 °C, 23 h Ar substituents: OMe, Me, styrene, COOR, CN, $CF_3$, COPh, COMe; 12 examples, 50-96%. | 202 |
| 7 | 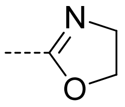 | Arylation | PhCl | 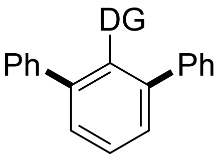 | 2-Phenyloxazoline (0.5 mmol), ArCl (1.25 mmol, 2.5 equiv), $[RuCl_2(p\text{-cymene})]_2$ (2.5 mol%), KOPiv (10 mol%), $K_2CO_3$, (3 equiv), DEC (2 mL); 1 example, 96%. | 158 |
| 8 | 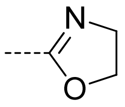 | Arylation | Ar-Br | 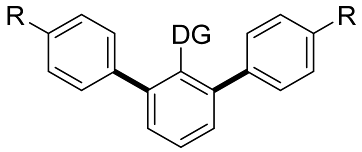 | $[RuCl_2(p\text{-cymene})]_2$ (5 mol%), KOAc (20 mol%), $PPh_3$ (10 mol%), $K_2CO_3$ (4 equiv), $H_2O$ (2 mL), 110 °C, 20 h R = H, Me, F, $CF_3$, OMe, Cl; 6 examples, 12-87% | 203 |
| 9 | 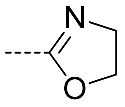 | Cyanation | 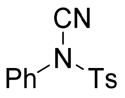 | 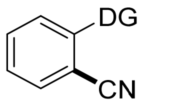 | Substrate (1 equiv), cyanating reagent (2 equiv), $[RhCp^*(CH_3CN)_3](SbF_6)_2$ (5 mol%), $Ag_2CO_3$ (20 mol %), dioxane. Single example, 76% | 212 |
| 10 | 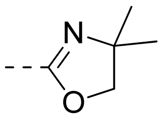 | Alkenylation | 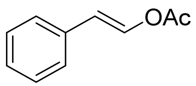 | 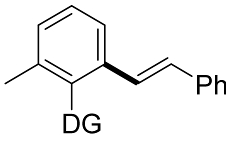 | Substrate (1 mmol), alkene source (3 mmol), Ru(cod)(cot) (0.05mmol), 2,6-lutidine (2 mmol), toluene (1.5 mL), reflux, 40 h. Single example, 69% | 190 |
| 11 | 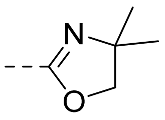 | Alkenylation | 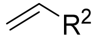 | 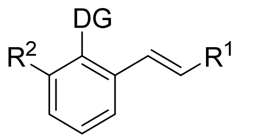 | Substrate (1 equiv), alkene (1.3 equiv), $[RuCl_2(p\text{-cymene})]_2$ (5 mol%), 1,1′-binaphthyl-2,2′-diyl hydrogenphosphate (10 mol%), $Cu(OAc)_2.H_2O$ (0.8 equiv), EtOH (2 mL), 80 °C under air. $R^1$ = various esters, alkyls and aryls, amides; $R^2$ = H, Me; 6 examples, 32-81% | 211 |

| | | | | | | |
|---|---|---|---|---|---|---|
| 12 | 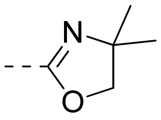 | Alkylation | $R_4Sn$ | 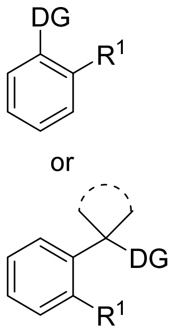 | $Pd(OAc)_2$ (10 mol%), organotin reagent (0.75 equiv in 10 batches a 0.075 equiv or as 20 batches a 0.037 equiv), $Cu(OAc)_2$ (1 equiv), benzoquinone (1 equiv), MeCN, 100 °C, 40-60h<br>$R^1$ = Me, Et, Pr, Bu, Oct; 19 examples, 62-90%.<br>Cyclic substrates included cyclopropyl, cyclopentyl and cyclohexyl<br>Microwave irradiation reduced the reaction time to 10h | 197 |
| 13 | 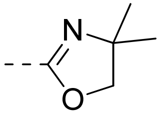 | Alkylation | 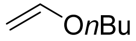 | 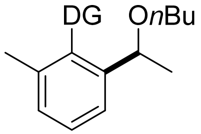 | Substrate (1 equiv), olefin ether (1.5 equiv), [IrCl(cod)]2 (5 mol% Ir), $NaBAr^F{}_4$ (10 mol %), toluene, 80 °C, 48 h.<br>1 example, 94%; gives selectively the branched product! | 198 |
| 14 | 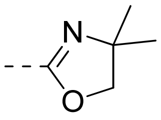 | Arylation | Ar-H | 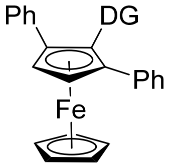 | $Pd(OAc)_2$ (10 mol%), $Cu(OAc)_2$ (2 equiv), benzene, $K_2CO_3$ (2.3 equiv), 120 °C, sealed tube<br>39% bis-arylation, 4% mono-arylation<br>Only example catalytic in Pd | 213 |
| 15 | 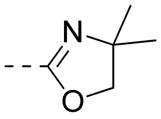 | Arylation | Ar-I | 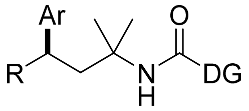 | $Pd(OAc)_2$ (5 mol%), Ar-I (3 equiv), $Ag_2CO_3$ (2 equiv), 1-AdCOOH (0.5 equiv), DCE, $N_2$, 110 °C, 24h<br>R = Et, Pr, *n*Bu, $CH_2Cy$, *n*heptyl, $CH_2CF_3$, *i*Bu, $(CH_2)_2Ph$, and others; Substituents on Ar reported: Et, Ac, OMe, NHAc, F, Cl, OAc, $CF_3$, $CO_2Me$, CN, $NO_2$; 28 examples, 15-83%.<br>Cleavage of DG: 6M HCl reflux, then NaOH, 1 example, 62% | 200 |
| 16 | 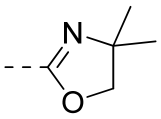 | Arylation | Ar-I | 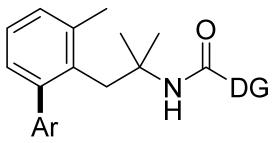 | $Pd(OAc)_2$ (10 mol%), Ar-I (3 equiv), $Ag_2CO_3$ (2 equiv), 1-AdCOOH (0.5 equiv), DCE, $N_2$, 110 °C, 24h<br>Substituents on Ar reported: Ac, OMe, NHAc, F, $CF_3$, $CO_2Me$, $NO_2$; 7 examples, 27-80%. | 200 |

| | | | | | | |
|---|---|---|---|---|---|---|
| 17 | 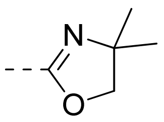 | Carbonylation | CO, $CH_2$=$CH_2$ | 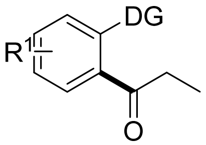 | 5 mol% $Ru_3(CO)_{12}$, ethylene, CO, 20 atm, toluene, 160 °C<br>$R^1$ = Me, $CF_3$, OMe, F, Ph, $Me_3Si$, $CH_2SiMe_3$, OTBDMS, Br, Cl, CN, $NMe_2$; 17 examples, up to 98% yield. | 196 |
| 18 | 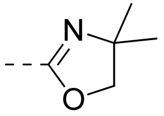 | Silylation | $HSiEt_3$ | 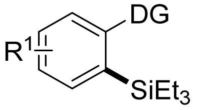 | Substrate (1 mmol), $HSiEt_3$ (5 mmol), norbornene (5 mmol), $Ru_3(CO)_{12}$ (6 mol%), toluene (0.5 ml), reflux, 20h.<br>$R^1$ = H, F, Me, OMe, $CF_3$;<br>8 examples, 26%-quant yield.<br>$CF_3$ in 2 position gave the low yield of 26%, all other examples were significantly better. | 161 |
| 19 | 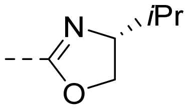 | Amidation | R-NCO | 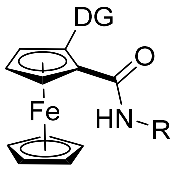 | [RhCp*$(OAc)_2(H_2O)$] (5 mol%), $HBF_4.Et_2O$ (10 mol%), THF, 75 °C;<br>R = Ph, 4-$MeC_6H_4$, 4-$ClC_6H_4$, Bn, *n*Bu, cyclohexyl; 6 examples, 24-69%<br>Single diastereomer. | 210 |
| 20 | 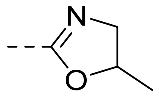 | Amidation | 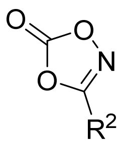 | 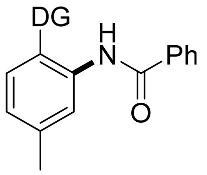 | Substrate (1 equiv), dioxazolone (1.2 equiv), Cp*Co(CO)$I_2$ (5 mol%), $AgSbF_6$ (20 mol%), NaOAc (20 mol%), DCE, 100 °C, 20 h.<br>Single example, 85% | 15 |
| 21 | 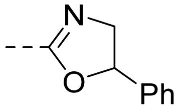 | Amidation | 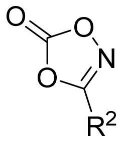 | 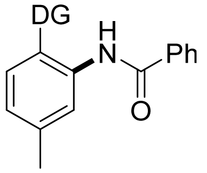 | Substrate (1 equiv), dioxazolone (1.2 equiv), Cp*Co(CO)$I_2$ (5 mol%), $AgSbF_6$ (20 mol%), NaOAc (20 mol%), DCE, 100 °C, 20 h.<br>Single example, 74% | 15 |
| 22 | 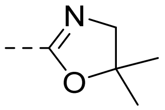 | Amidation | 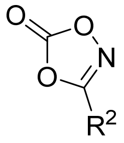 | 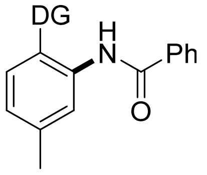 | Substrate (1 equiv), dioxazolone (1.2 equiv), Cp*Co(CO)$I_2$ (5 mol%), $AgSbF_6$ (20 mol%), NaOAc (20 mol%), DCE, 100 °C, 20 h.<br>Single example, 86% | 15 |

| | | | | | | |
|---|---|---|---|---|---|---|
| 23 | 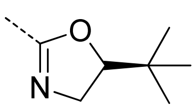 | Iodination | $I_2$ | 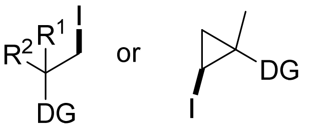 | 10 mol% $Pd(OAc)_2$, $PhI(OAc)_2$ (1 equiv), $I_2$ (1 equiv), DCM, 24 – 50 °C<br>4 examples, 62-98%, 91:9 – 99:1 d.r. | 204 |
| 24 | 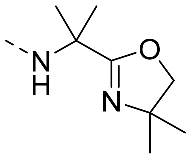 | Arylation | ArI | 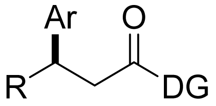 | $Pd(OPiv)_2$ (10 mol%), $Ag_2CO_3$ (1.5 equiv), $(BnO)_2PO_2H$ (1.0 equiv), HFIP/DMSO (9:1) (1.0 mL), 120 °C, $N_2$, 12 h.<br>R = Me: 11 examples with substituted iodobenzenes carrying OPiv, $OCF_3$, OMe, OTs, NHAC, Me, $CF_3$, Br, and COOMe substituents; 42-70% yield.<br>R = (substituted) benzyl or alkyl: 8 examples, 37-68%. | 201 |
| 25 | 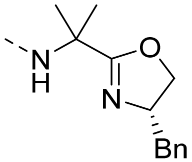 | Arylation | ArI | 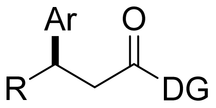 | $Pd(OPiv)_2$ (10 mol%), $Ag_2CO_3$ (1.5 equiv), $(BnO)_2PO_2H$ (1.0 equiv), HFIP/DMSO (9:1) (1.0 mL), 90 °C, $N_2$, 12 h.<br>R = Ph or 4-$BrC_6H_4$; Ar = phenyl, 4-$MeOC_6H_4$, or 4-$BrC_6H_4$; 3 examples, 50-66%, 88:12 – 90:10 d.r. | 201 |
| 26 | 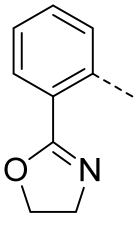 | Alkylation - Amination | 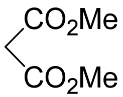 | 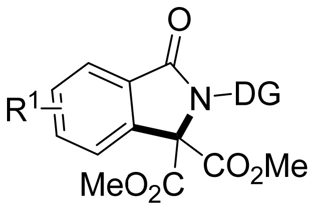 | Substrate (0.10 mmol), malonate (0.2 mmol), $Cu(OAc)_2$ (20 mol%), $Li_2CO_3$ (0.1 mmol), $Ag_2CO_3$ (0.15 mmol), DMSO (4.0 mL), air, 80 °C, 12 h.<br>$R^1$ = H, Me, *t*Bu, MeO, F, Cl, Br, I, Ac, $CF_3$, Ph, $CH_2$=CH; 20 examples, 40-72%.<br>C−H cleavage could potentially be the rate-limiting step | 208 |
| 27 | 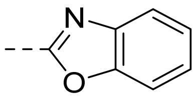 | Arylation | Ar-Br | 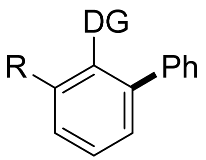 | Substrate (0.5 mmol), PhBr (0.6 mmol), $[RuCl_2(\eta^6\text{-}C_6H_6)]_2$ (0.0125 mmol), $PPh_3$ (0.05mmol), $K_2CO_3$ (1.0 - 2.0 mmol), NMP (1 mL), 120 °C, $N_2$, 20 h.<br>DG = Oxazole: R = Me, 1 example, 66%<br>DG = Benzoxazole: Bisarylation occurred (R = Ph),<br>Single example, 55% | 157 |

| | | | | | | |
|---|---|---|---|---|---|---|
| 28 | 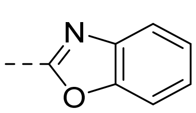 | Fluorination | NFSI | 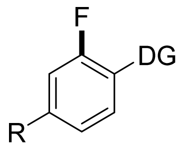 | Substrate (1 equiv), NFSI (1.5 equiv), $Pd(OAc)_2$ (10 mol%), TFA (2 equiv), and a mixed solvent ($CH_3NO_2$/ $CH_3CN$, 2.0 mL), sealed tube, air, 110 °C R = H (64%), Cl (35%), OMe (53%) | 214 |
| 29 | 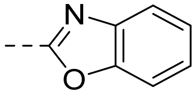 | Acylation | 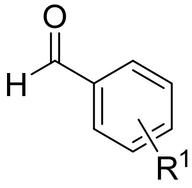 | 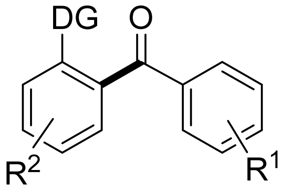 | Substrate (0.4 mmol), aldehyde (2.4 mmol), $Pd(OAc)_2$ (5 mol%), $PPh_3$ (10 mol%), TBHP (2.4 mmol), PhCl (2.0 mL), reflux, 8 h.<br>$R^1$ = H, Br, Cl, F, Me, MeO; $R^2$ = H, Cl, F, Me, MeO;<br>17 examples, 39-85%.<br>Also benzothiazole and benzo[h]quinolone was used as DG successfully, benzimidazole gave no conversion. | 209 |
| 30 | 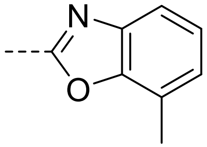 | Acylation | 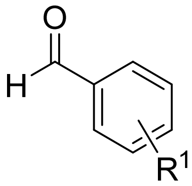 | 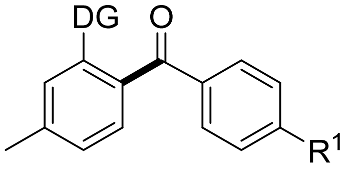 | Substrate (0.4 mmol), aldehyde (2.4 mmol), $Pd(OAc)_2$ (5 mol%), $PPh_3$ (10 mol%), TBHP (2.4 mmol), PhCl (2.0 mL), reflux, 8 h.<br>$R^1$ = H, OMe, Cl; 3 examples, 42-81%.<br>Also benzothiazole and benzo[h]quinolone was used as DG successfully, benzimidazole gave no conversion. | 209 |
| 31 | 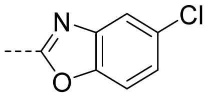 | Acylation | 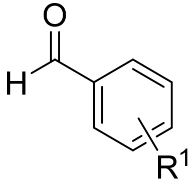 | 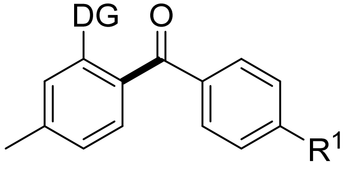 | Substrate (0.4 mmol), aldehyde (2.4 mmol), $Pd(OAc)_2$ (5 mol%), $PPh_3$ (10 mol%), TBHP (2.4 mmol), PhCl (2.0 mL), reflux, 8 h.<br>$R^1$ = H, Br, Cl; 3 examples, 37-55%.<br>Also benzothiazole and benzo[h]quinolone was used as DG successfully, benzimidazole gave no conversion. | 209 |
| 32 | 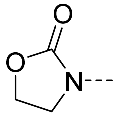 | Arylation | $[Ph_2I]BF_4$ | 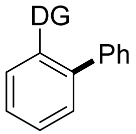 | Substrate (1 equiv), $[Ph_2I]BF_4$ (1.1 – 2.5 equiv), $Pd(OAc)_2$ (5 mol%), $NaHCO_3$ (1.5 - 2.0 equiv), benzene, 100 °C, 12 h.<br>Single example, 83% | 215 |

| | | | | | | |
|---|---|---|---|---|---|---|
| 33 | 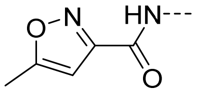 | Alkylation | R-I | 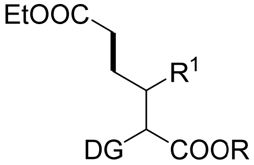 | Substrate (1 equiv), R-I (3 equiv), $Pd(OAc)_2$ (10 mol%), AgOAc (2 equiv), toluene, air, 80 °C, 24 h; $R^1$ = Me, Et, O*t*Bu; 4 examples, 73-91%. | 199 |
| 34 | 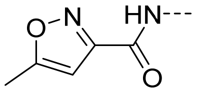 | Arylation | Ar-I | 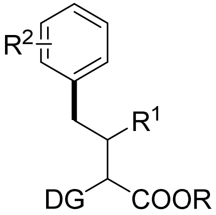 | Substrate (1 equiv), Ar-I (3 equiv), $Pd(OAc)_2$ (10 mol%), AgOAc (2 equiv), toluene, air, 80 °C, 24 h; $R^1$ = H, Me, Et, O*t*Bu; 4 examples, $R^2$ = Me, OMe, COMe, F, Cl, Br, I, $NO_2$, $CF_3$, $B(OR)_2$, $N_3$; 16 examples, 61-93%. | 199 |
| 35 | 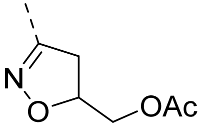 | Iodination | NIS | 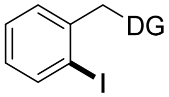 | $Pd(OAc)_2$ (5 mol%), NIS (1.05 equiv), 100-120 °C, 12 h, MeCN or AcOH. 1 example, 54% | 160 |
| 36 | 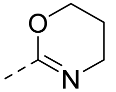 | Amidation | 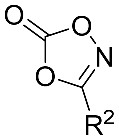 | 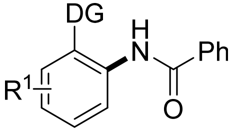 | Substrate (1 equiv), dioxazolone (1.2 equiv), $Cp^*Co(CO)I_2$ (5 mol%), $AgSbF_6$ (20 mol%), NaOAc (20 mol%), DCE, 100 °C, 20 h. 2 examples: $R^1$ = 3-F (71%), 4-Me (66%) | 15 |
| 37 | 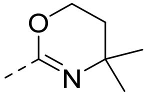 | Carbonylation | CO, $CH_2$=$CH_2$ | 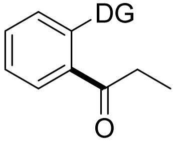 | 5 mol% $Ru_3(CO)_{12}$, ethylene, CO, 20 atm, toluene, 160 °C single example, 37%. | 196 |

## Heterocyclic and related directing groups in C-H activation chemistry

Imidazole derivatives are rarely applied as DGs, since the imidazole ring system is relatively prone to C-H insertions itself. One example has been reported by Kakiuchi and coworkers in their well-known ruthenium catalyzed silylation protocol (Table 8, Entry 2).[161]

Here, amongst series of other heterocyclic DGs, also one example using a imidazole derivative was reported. Another single example in a larger study came from the lab of Inoue. *N*-Methylimidazole proved to be efficient in promoting only mono-arylation, whereas other heterocyclic DGs often also gave bis-arylation (Table 8, Entry 3).[157] Most likely, the *N*-methyl group prevents free rotation around the phenyl-DG bond and the second ortho position cannot be activated anymore.

Thiazole compared to other heterocycles, is underrepresented as DG. Reasons are again that thiazole can be C-H activated itself, primarily in positions 2 and 5. Hence, conditions in which thiazole is applied as DG need to leave the thiazole C-H bonds untouched. The Ru-catalyzed arylation protocol reported by Inoue and coworkers is such an example (Table 8, Entry 3).[157] In order to get selective mono-arylation of arenes, one *ortho* position had to be blocked in advanced, otherwise mixtures of mono- and bis-arylation were obtained. Taking such precautions, the reaction was generally high yielding (up to 98%).

Already in 2000 Murai and coworkers[196] reported one example of thiazoline as DG in a carbonylation reaction with CO and ethylene (Table 8, Entry 7).

The triazene motif was used as 'internally cleavable' directing group in the synthesis of free indoles by Sun et al.[216] The so formed indole derivatives could be further transformed to the corresponding indolo[2,1-*a*]isoquinolines in a 2-step one pot set up (Table 8, Entry 9 & 10). The process is believed to be a triple C-H/N-H/C-H activation cascade. Ghorai and Chouhury[217] showed the application of an *N*-heterocyclic carbene (NHC) as directing group in an intermolecular C-H activation / annulation reaction employing various alkynes as reaction partners. To the best of our knowledge, this represents the only example in the literature where NHCs are used as directing groups rather than as ligands. 7-Azaindoles have been investigated concerning their potential applicability in C-H activation by Qian et al. (Table 8, Entry 13)[218] The attempted chlorination using dichloroethane as halogen-source proceeded smoothly and according to the mechanism proposed, the directing effect is attributed to the pyridine –nitrogen. Phenidones (Table 8, Entry 12) have been selectively *ortho*- aminated by Xue et al.[219] The phenindone motif is readily transformed to other heterocyclic compounds.

In 2015 the group of Zhang presented pyridazinone as DG, which can be used for different direct transformations, like the Pd(II) promoted arylation with aryl iodides or using benzaldehyde derivatives for a regioselective carboxylation. Additionally, the pre-catalyst $[RhCp^*Cl_2]_2$ was used for olefination or naphthylation using diphenylethyne under the same reaction conditions.[21]

An monoselective alkenylation protocol established for 2-aryl-1,3-dithiane derivatives catalyzed by a Rh(III) complex was reported by Unoh et al. This rarely known directing group can be directly removed after the C-H functionalisation by the Dess−Martin periodinane reagent in a $MeCN/CH_2Cl_2/H_2O$ co-solvent system at room temperature resulting in deprotection to furnish aldehydes in an excellent yield. Another stated deprotection protocol including a reductive desulfurization and alkene reduction was achieved by treatment with Raney-Ni in EtOH, rt. for 6 h.[220]

**Table 8: Heterocyclic and related directing groups in C-H activation chemistry**

| Entry | Directing group | Type of transformation | Coupling partner | Typical product structure | Comments | Ref |
|---|---|---|---|---|---|---|
| 1 | 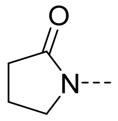 | Arylation | $[Ph_2I]BF_4$ | 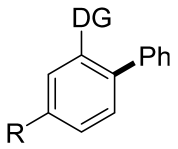 | Substrate (1 equiv), $[Ph_2I]BF_4$ (1.1 – 2.5 equiv), $Pd(OAc)_2$ (5 mol%), $NaHCO_3$ (1.5 - 2.0 equiv), toluene, 100 °C, 12 – 24 h.<br>3 examples, R = H (75%), OMe (84%), Br (78%). | 215 |
| 2 | 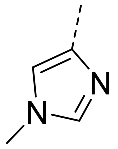 | Silylation | $HSiEt_3$ | 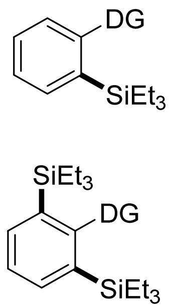 | Substrate (1 mmol), $HSiEt_3$ (5 mmol), norbornene (5 mmol), $Ru_3(CO)_{12}$ (6 mol%), toluene (0.5 ml), reflux, 20h<br>Single example, 13% mono silylation, 56% bis silylation | 161 |
| 3 | 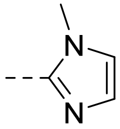 | Arylation | Ar-Br | 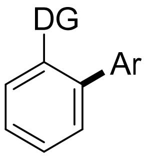 | Substrate (0.5 mmol), PhBr (0.6 mmol), $[RuCl_2(\eta^6\text{-}C_6H_6)]_2$ (0.0125 mmol), $PPh_3$ (0.05mmol), $K_2CO_3$ (1.0 - 2.0 mmol), NMP (1 mL), 120 °C, $N_2$, 20 h.<br>Ar = Ph (84%), Ar = 3-thienyl (83%). | 157 |
| 4 | 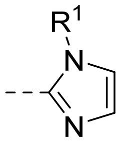 | Alkylation | 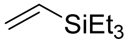 | 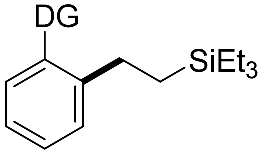 | Substrate (1 equiv), alkene (3 equiv), $[RuCl_2(p\text{-cymene})]_2$ (2.5 - 5 mol%), MesCOOK (30 mol%), toluene, 100 – 120 °C, 18 – 24 h.<br>2 examples, $R^1$ = H (50%), Me (51%). | 24 |

| | | | | | | |
|---|---|---|---|---|---|---|
| 5 | 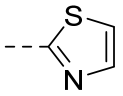 | Arylation | Ar-Br | 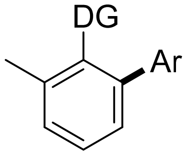 | Substrate (0.5 mmol), PhBr (0.6 mmol), $[RuCl_2(\eta^6\text{-}C_6H_6)]_2$ (0.0125 mmol), $PPh_3$ (0.05mmol), $K_2CO_3$ (1.0 - 2.0 mmol), NMP (1 mL), 120 °C, $N_2$, 20 h.<br>Ar substituents included H, Me, OMe, F, COMe; 8 examples, 63-98% | 157 |
| 6 | 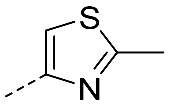 | Alkenylation | 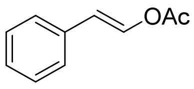 | 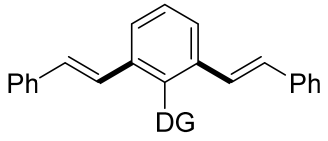 | Substrate (1 mmol), alkene source (3 mmol), 3 (0.05mmol), toluene (1.5 mL), reflux, 50 h.<br>Single example, 60% | 190 |
| 7 | 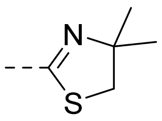 | Carbonylation | CO, $CH_2$=$CH_2$ | 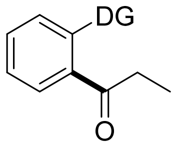 | 5 mol% $Ru_3(CO)_{12}$, ethylene, CO, 20 atm, toluene, 160 °C<br>Single example, 73%. | 196 |
| 8 | 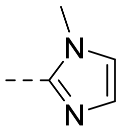 | Alkylation/Alkenylation | Alkene/Alkyne | 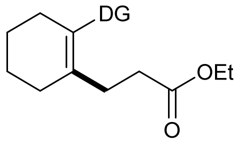 | Olefin (1 equiv), unsaturated coupling partner (1 equiv), $[ReBr(CO)_3(THF)]_2$ (2.5 mol%), toluene, 135 °C, 24 h<br>13 examples, yields generally above 50% | 221 |
| 9 | 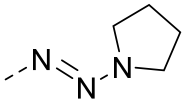 | Synthesis of Indoles | 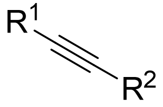 | 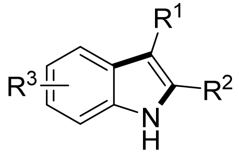 | Substrate (0.3 mmol),alkyne (1.1 equiv), $[RhCp^*Cl_2]_2$ (5 mol%), $AgSbF_6$ (20 mol%), $Cu(OPiv)_2$ (2 equiv), MeOH/$^t$AmOH=1:1, 90 °C, under argon.<br>12 Examples; 47-93%<br>$R^1$ = Aryl, Alkyl<br>$R^2$ = Aryl<br>$R^3$ = CN, COOMe, Ac, Br, OMe, $NO_2$, $CH_2OH$ | 216 |

| | | | | | | |
|---|---|---|---|---|---|---|
| 10 | | Synthesis of Indolo[2,1-*a*]isoquinolines | 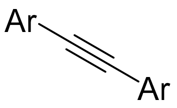 | 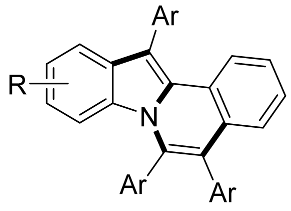 | Method A: Substrate (0.2 mmol),alkyne (3.5 equiv), $[RhCp*Cl_2]_2$ (5 mol%), $AgSbF_6$ (20 mol%), $Cu(OPiv)_2$ (2 equiv), MeOH/$^t$AmOH=1:1, 90°C, under argon.<br>Method B: Substrate (0.2 mmol),alkyne (3.5 equiv), $[RhCp*Cl_2]_2$ (5 mol%), $AgSbF_6$ (20 mol%), $Cu(OAc)_2$▪$H_2O$ (2 equiv), DCE, 90°C, under argon.<br>16 Examples; 35-79%<br>R = Ac, halogen, OMe, CN, $NO_2$ | |
| 11 | 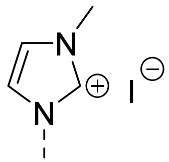 | | 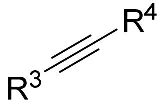 | 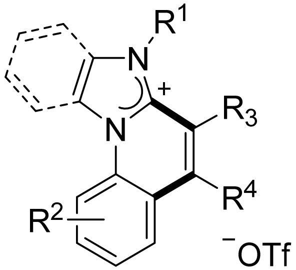 | Substrate (0.1 mmol), reagent (0.12 mmol), $[Cp*RhCl_2]_2$ (5 mol-%), NaOAc (4 equiv), AgOTf (3 equiv), $CH_2Cl_2$ (3.0 mL), $N_2$ atm. rt., 24 h.<br>16 Examples; 29-80% yield<br>$R^1$ = Alkyl<br>$R^2$ = OMe, $NO_2$<br>$R^3$ = Ph, COOMe, alkyl | 217 |
| 12 | 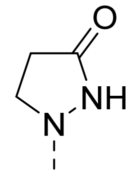 | Amination | 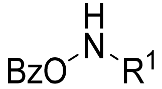 | 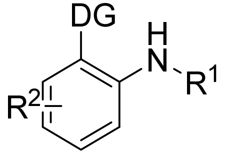 | Substrate (0.5 mmol), reagent (0.75 mmol), $[Cp*RhCl_2]_2$ (4 mol-%), CsOAc (2 equiv), PivOH (0.5 equiv) in toluene (2 mL) under air, 3–5 h.<br>$R^1$ = Alkyl<br>$R^2$ = Me, OMe, $OCF_3$, Br, $NO_2$ | 219 |

| | | | | | | |
|---|---|---|---|---|---|---|
| 13 | 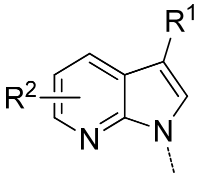 | Chlorination | 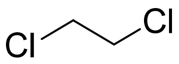 | 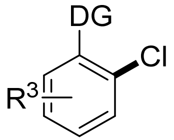 | Substrate (0.3 mmol), Rh[Cp*$Cl_2$]$_2$ (2.0 mol%), Cu$(TFA)_2$ (2.0 equiv), $^{t}$BuNC (2.0 equiv), $Li_2CO_3$ (1.0 equiv), DCE (1.5 mL), 130 °C, air, 23−53 h.<br>31 Examples; 32-85% yield<br>$R^1$ = Halogen, C(O)R, aryl<br>$R^2$ = OMe, halogen, aryl, alkyl<br>$R^3$ = Halogen, OMe, Me, COOMe, C(O)Me, $NO_2$ | 218 |
| 14 | 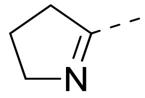 | Arylation | 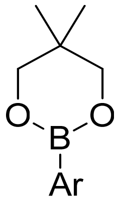 | 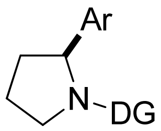 | Substrate (1 mmol), coupling partner (1.2 equiv), $^{t}$BuCOMe (5 equiv), $Ru_3(CO)_{12}$ (3.3 mol%), 150 °C, 4-19 h<br>9 Examples, 38-76% yield | 1 |
| 15 | 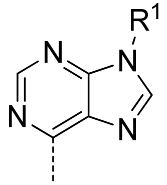 | Amination | $N_3$–Ar | 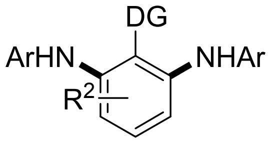 | Condition A: Aryl azide (0.2 mmol), Substrate (2 equiv), [RhCp*$Cl_2$]$_2$ (2mol%), and $AgSbF_6$ (8 mol%) in $ClCH_2$–$CH_2Cl$ for 12 h obtain mono substitution.<br>Condition B: Substrate (0.2 mmol), aryl azide (3 equiv), [RhCp*$Cl_2$]$_2$ (4 mol%), and $AgSbF_6$ (16 mol%) for 24 h obtain double substitution.<br>22 Examples; Yield: 58-95%<br>$R^1$= Alkyl, Bn<br>$R^2$= Me, Cl, $NO_2$, OH, $CF_3$, CHO, COOMe, $SO_2Me$ | 222 |

| | | | | | | |
|---|---|---|---|---|---|---|
| 16 | 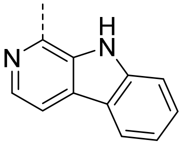 | Arylation | ArBr | 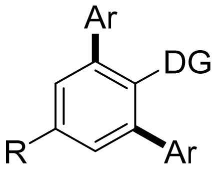 | Substrate (0.2 mmol), ArBr (0.5 mmol), $[RuCl_2(p\text{-cymene})]_2$ (5.0 mol%), $K_2CO_3$ (0.5 mmol), $Ph_2CHCO_2H$ (30 mol%), NMP, 120 °C, 20 h.<br>Monoarylated product was observed in small amount.<br>Mechanism was studied<br>39 Examples; Yield: 40-92%<br>R= Me, OMe, CN, F, $NO_2$<br>Substituted ArBr: Bu, OMe, CN, $NMe_2$,<br>Hetero-bromides tolerated | 223 |
| 17 | 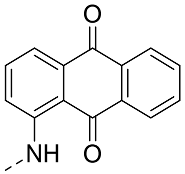 | Acetylation | $PhI(OAc)_2$ | 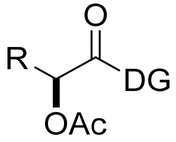 | Substrate (0.1 mmol), $Pd(OAc)_2$ (0.1 equiv), $PhI(OAc)_2$ (5 equiv) and LiOAc (2 equiv) in DCE (0.5 mL) in a sealed tube at 120 °C for 12 h.<br>Removal: NaOH (2 equiv), in MeOH, reflux, 10 min; released 1-amino anthraquinone can be reused.<br>17 Examples; Yield: 0-92%<br>R= Alkyl, cyclopentane, cyclohexane, aryl, vinyl, halogen | 40 |
| 18 | 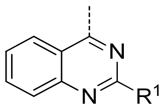 | Arylation oxidation | 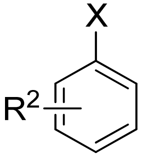 | 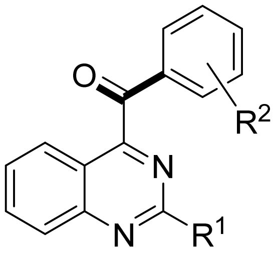 | Substrate (0.3 mmol), ArX (2 equiv), $Pd(PPh_3)_4$ (0.1 equiv), BINAP (0.1 equiv), $Cs_2CO_3$ (2 equiv), air, toluene (3mL), 100 °C.<br>28 Examples, Yield: 0-79%<br>$R^1$= Alkyl, Ph, Cy | 224 |

| | | | | | | |
|---|---|---|---|---|---|---|
| | | | | | $R^2$= Me, OMe, halogen, $NO_2$, $CF_3$, CHO, COOMe<br>X= Cl, Br, I | |
| 19 | 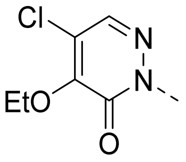<br>(Pyridazinone) | Arylation | ArI | 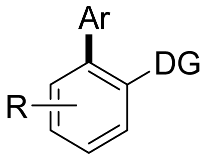 | Substrate 0.1 mmol, reagent (10 equiv), AgOAc (1.5 equiv), $Pd(OAc)_2$ (10 mol%).<br>18 Examples; Yield: 12-84%<br>R= Alkyl, COOMe, OMe, halogen | [21] |
| 20 | | Carboxylation | RCHO | 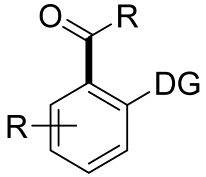 | Substrate (0.28 mmol), aldehyde (1.5 equiv), $Pd(OAc)_2$ (10 mol%), TBHP (2 equiv), DCE, 80 °C, 5 h.<br>6 Examples; Yield: 60-90%<br>R= substituted Ph, Cy, thiophene (Cl, $NO_2$, OMe) | |
| 21 | | Olefination | 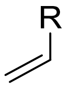 | 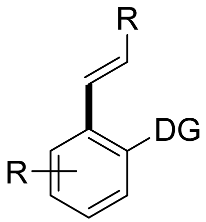 | Substrate (0.28 mmol), alkene (2 equiv), $[RhCp^*Cl_2]_2$(2.5 mol%), $AgSbF_6$(10 mol%), PivOH (2 equiv), $Ag_2CO_3$(2 equiv), MeOH, 100 °C, 2 h.<br>3 Examples; Yield: 91-96%<br>R= $COO^nBn$, $CONH_2$, Ph | |
| 22 | | Naphthylation | 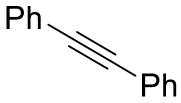 | 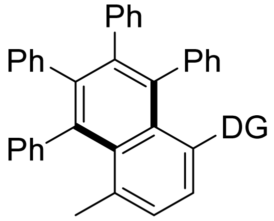 | Substrate (0.26 mmol), diphenylacetylene (2 equiv), $[RhCp^*Cl_2]_2$ (2.5 mol%), $AgSbF_6$(10 mol%), $Ag_2CO_3$(2 equiv), PivOH (2 equiv), MeOH, 100 °C.<br>1 Examples; Yield: 89% | |

| | | | | | | |
|---|---|---|---|---|---|---|
| 23 | 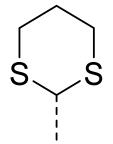 | Alkenylation | 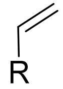 | 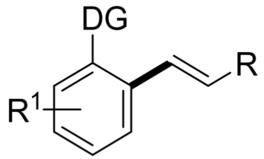 | Substrate (0.25 mmol), reagent (0.5 mmol), $[Cp^*Rh(MeCN)_3][SbF_6]_2$ (0.02 mmol), $Cu(OAc)_2 \cdot H_2O$ (0.5 mmol), in THF (2 mL) at 60 °C under $N_2$ for 24 h.<br>2 removal protocol: (1):- Dess−Martin periodinane reagent in a $MeCN/CH_2Cl_2/H_2O$ co-solvent system at room temperature resulted in deprotection to furnish aldehydes in an excellent yield. (2):- reductive desulfurization/alkene reduction was archived by treatment with Raney-Ni in EtOH, rt. for 6 h.<br>27 Examples; Yield: 0-93%<br>R= $COO^nBu$, $COO^tBu$<br>$R^1$= Me, alkoxy, MeS, NO2, halogene, CF3,<br>Heterocycles tolerated | 220 |

## Amides as directing groups

Substituted as well as unsubstituted amides proved to be extremely valuable as directing groups in C-H activation. The first example utilizing an unsubstituted amide ($CONH_2$) in C-H activation was presented by Li et al. in 2012 (Table 9, Entry 1).[77] Employing AcOH as solvent, facilitates the electrophilic attack of the Pd(II) catalyst to the benzamide coupling partner. Both, electron donating as well as electron withdrawing substituents were tolerated on both reaction partners and a variety of biphenyl-2-carboxamides could be prepared in synthetically useful yields of up to 84%. An extremely diverse set of amide- facilitated *ortho*-modifications is known in the literature amongst which not only arylation reactions are represented but also less classical amidation, amination and alkylation reactions. The Chang group presented the enormously functional group tolerant amination starting from *N-t*-butylbenzamide and an aryl azide as coupling partner (Scheme 5, Table 9, Entry 11).[225] $AgSbF_6$ facilitates the ligand exchange in the catalytic system and thereby the formation of a five-membered rhodacyclic intermediate **(4)**. The azide reaction partner coordinates to this intermediate and subsequent loss of $N_2$ via either pathway b or via formation of a nitrenoid **6** (pathway a) affords the Rh(III) amido spezies **7** which upon protonolysis delivers the ortho-*N*-functionalized aniline**8**.

The same group reported an extremely mild (the reaction is conducted at 45 °C) iridium-catalyzed arylation of benzamides with aryldiazonium tetrafluoroborates as coupling partners (Table 9, Entry 13).[226] With this reaction conditions, also (*Z*)-selective arylation of enamides was accomplished in good yields (40-83%). In 2012 Sharma et al. presented the tandem *ortho*-acylation of *N-i*-propylbenzamides followed by intramolecular cyclization (Table 9, Entry 6).[227] This Rh-catalyzed sequential process allowed for the elegant preparation of substituted 3- hydroxyisoindoles and was later developed further towards an enantioselective transformation utilizing and iridium-based catalyst and a chiral bidentate phosphoramidite ligand[228, 229] employing *N,N*-dimethylamide as directing group (Table 9, Entry 17).

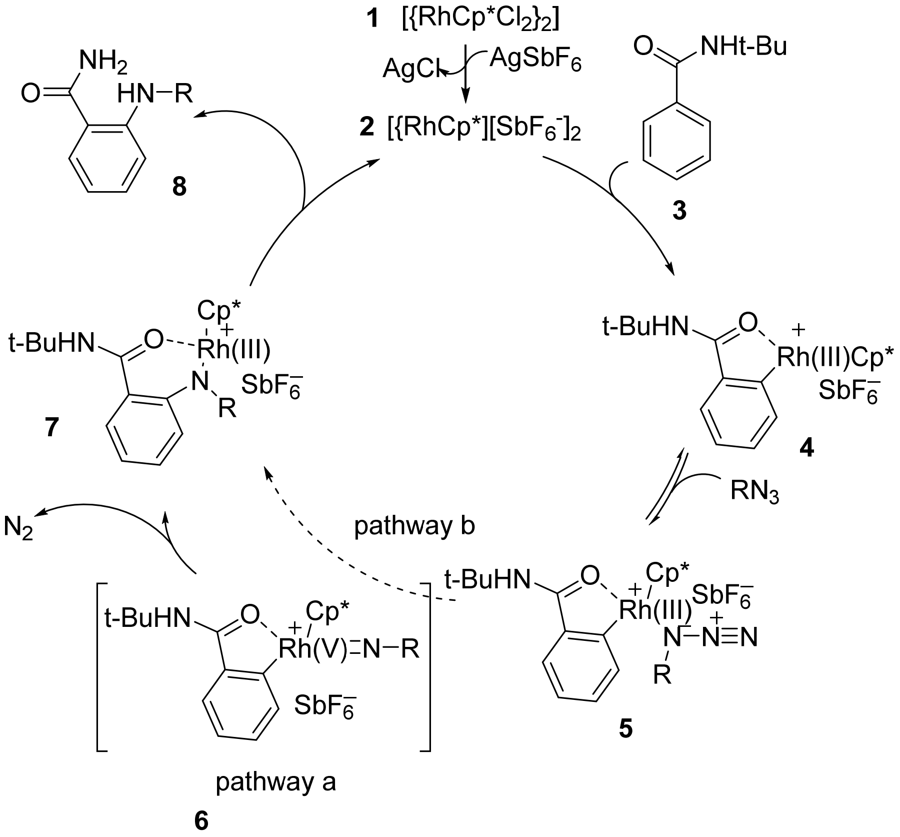


**Scheme 5: Proposed mechanism of the Rh- catalyzed amination of ᵗBu-benzamides.**

The disubstituted *N,N*-diethylamide was successfully applied in the meta-selective borylation of aromatic substrates (Table 9, Entry 19).[230] Although this reactivity was not exclusively shown for the *N,N*-diethylamide (OMOM, $SONEt_2$ and $OCONEt_2$ could also be applied to the reaction conditions), this unique meta-selectivity should be mentioned here. The investigated sequential meta-borylation, Suzuki-coupling is perfectly complementary to directed *ortho*-metalation and Suzuki coupling or an *ortho* functionalization via directed C-H activation. The transformation shows a good functional group tolerance and the meta-selectivity seems to be driven by steric influences rather than electronic effects. A Rh-catalyzed protocol for the Z-selective α-halogenation of alkenes was presented by the Glorius group (Table 9, Entry 27).[231] Halo-acrylamides could be prepared using NXS as the halogen source which in most cases gave the best yields as NIS. The relatively mild conditions (60 °C) gave rise to a broad functional group tolerance allowing the reaction to proceed also in the presence of *p*- or *m*-bromine bringing about attractive synthetic intermediates.

The Glorius group utilized *N,N*-di-*i*-propylamide as directing group in the synthesis of the synthetically challenging [3]dendralene-motif.[232] Allenyl carbinol carbonates were used as reaction partner allowing for the installation of this motif on aromatic as well as olefinic starting materials under mild conditions with excellent functional group tolerance (Table 9, Entry 23).

Amongst aromatic amides, many serve for the alpha selective functionalization of $sp^3$- carbon centers. The enantioselective arylation of very congested cyclobutane is a particularly interesting example (Table 9, Entry 39).[233] The highly electron deficient *N*-(4-cyano-2,3,5,6-tetrafluorophenyl)amide in combination with a modified amino acid as chiral ligand enables the Pd-catalyzed alpha functionalization in good yields and high ees.

The very often challenging *meta*-modification of aromatic substrates was accomplished utilizing a modified norbornene which temporarily blocks the ortho position of the aromatic substrate thereby allowing for selective *meta* substitution with aryl– or alkyl iodides (Scheme 6). The relatively mild reaction conditions allow for a broad functional group tolerance and generally high yields. A second example for the rare meta functionalization was presented by the Yu group.[234] A U-shaped weakly coordinating fully functionalized amide-directing group enables the highly selective meta arylation (Table 9, Entry 40)[234] and olefination[235] of substituted and unsubstituted aromatic substrates. The immensely bulky amide totally shields the ortho position and can be cleaved at room temperature.

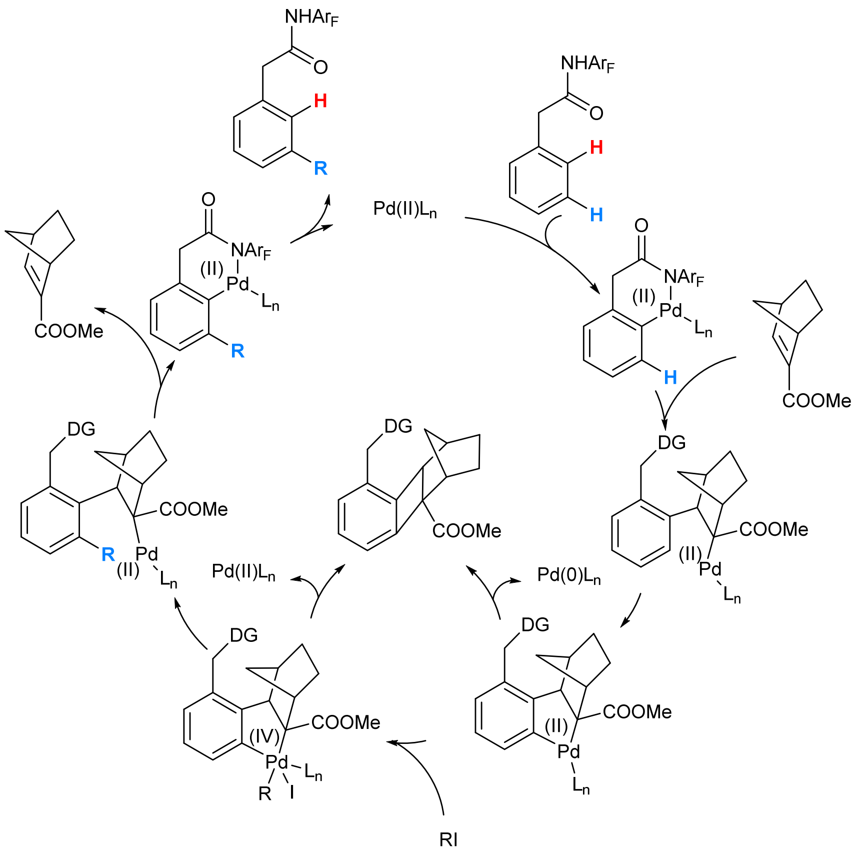


**Scheme 6: *Meta*-selective functionalization; Proposed mechanism.**

**Table 9: Amide- directing groups in C-H activation chemistry**

| Entry | Directing group | Type of transformation | Coupling partner | Typical product structure | Comments | Ref |
|---|---|---|---|---|---|---|
| 1 | 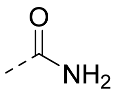 | Arylation | ArI | 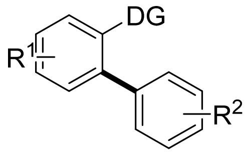 | Substrate (0.5 mmol), aryl iodide (1 mmol), $Pd(OAc)_2$ (0.025 mmol), $Ag_2O$ (1 mmol), AcOH (5 mL), 120 °C, 5-24 h<br>23 Examples; Yield: 33-84% with max. 9% diarylated byproduct<br>$R^1$ = Me, OMe, Halogen, $NO_2$<br>$R^2$ = Me, OMe, Cl<br>First example with unsubstituted amide | 77 |
| 2 | | Arylation | ArI | 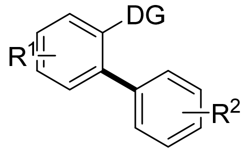 | Substrate (0.5 mmol), iodobenzene (1 mmol), PS-3 catalyst (15 mg), AgOAc (0.75 mmol), acetic acid (5 mL), 120 °C, 15-30 h<br>15 Examples, Yield: 36-74%<br>$R^1$ = Halogen, Me, OMe<br>$R^2$ = Me, OMe, C(O)Me<br>Novel calatyst system; Pd/ mesoporous silica | 236 |
| 3 | | Benzylation | 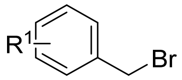 | 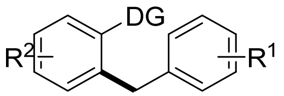 | Substrate (0.2 mmol), benzylbromide (0.2 mmol), $Pd(OAc)_2$ (5 mol%), $PPh_3$ (10 mol%), $Cs_2CO_3$ (0.24 mmol), dioxane (500 mM), 110 °C, 18 h<br>18 Examples; Yield: generally above 50%<br>$R^1$ = Me, OMe, $CF_3$, F<br>$R^2$ = OMe, Br, $CF_3$ | 237 |
| 4 | 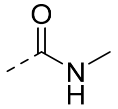 | Alkylation | RCl | 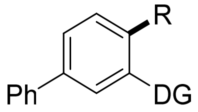 | Substrate (1 equiv), alkyl chloride (1.2 equiv), $Co(acac)_2$ (10 mol%), CyMgCl (3 equiv), DMPU (12 equiv), $Et_2O$, rt, 12 h<br>7 Examples; Yield: 15-73%<br>R = Alkyl | 238 |

| | | | | | | |
|---|---|---|---|---|---|---|
| 5 | | Alkylation | EtMgCl | 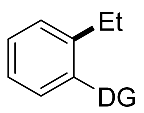 | Substrate (1 equiv), $Co(acac)_2$ (10 mol%), EtMgCl (5.8 equiv), DMPU (30 equiv), air, THF, 25 °C, 12 h<br>Yield: 79% | [239] |
| 6 | 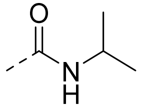 | Acylation / Intramolecular Cyclization | 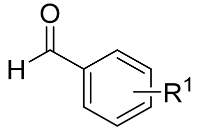 | 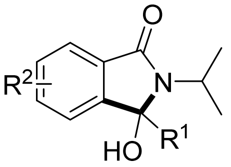 | Substrate (0.3 mmol), aldehyde (0.6 mmol), $[Cp^{*}RhCl_2]_2$ (5 mol%), $AgSbF_6$ (20 mol%), $Ag_2CO_3$ (0.9 mmol), THF (1 mL), 150 °C, 20 h, $N_2$, pressure tube<br>22 Examples; Yield: 30-83%<br>$R^1$ = $CF_3$, COOMe, $NO_2$, C(O)Me, CN, Halogen, OMe<br>$R^2$ = Ph, OR, halogen | [227] |
| 7 | 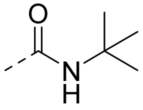 | Alkylation | 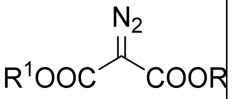 | 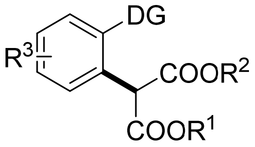 | Substrate (0.2 mmol), diazomalonate (1.2 equiv), $[IrCp^{*}Cl_2]_2$ (2 mol%), $AgNTf_2$ (8 mol%), AgOAc (4 mol%), DCE (1 mL), 90 °C, 10 h<br>14 Examples; Yield: generally above 50%<br>$R^1$ = Me<br>$R^2$ = Me, $^t$Bu, Bn<br>$R^3$ = Halogen, Me, OMe; heterocycles tolerated | [14] |
| 8 | | Amidation | 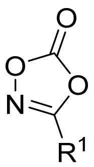 | 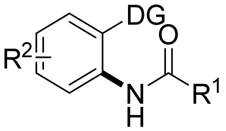 | Substrate (0.2 mmol), dioxazolone (1.1 equiv), $[Cp^{*}CoCl_2]_2$ (1 mol%), $AgSbF_6$ (4 mol%), NaOAc (6 mol%), DCE (0.5 mL), 80 °C, 24h<br>7 Examples; Yield: 60-83%<br>$R^1$ = Ph, Alkyl<br>$R^2$ = Me, $CF_3$, Halogen, OMe<br>Also pyridine and benzamide applicable to reaction conditions | [240] |
| 9 | | Amidation | sulfonyl azide | 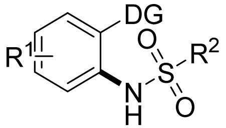 | Substrate (0.2 mmol), sulfonyl azide (1.1 equiv), $[IrCp^{*}Cl_2]_2$ (2 mol%), $AgNTf_2$ (8 mol%), DCE (0.5 mL), 50 °C, 12 h<br>24 Examples; Yield: generally above 60% | [241] |

| | | | | | | |
|---|---|---|---|---|---|---|
| | | | | | $R^1$ = Me, OMe, $CF_3$, $NO_2$, halogen, COOMe, $CH_2OR$<br>$R^2$ = Aryl, alkyl | |
| 10 | | Amination | $N_3R$ | 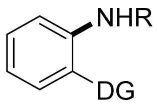 | Substrate (0.25 mmol), azide (0.35 mmol), $[Cp^*MCl_2]_2$ (4 mol%), $AgSbF_6$ (0.04 mmol), TCE (0.5 mL), 1.5 h; M = Ir, Rh, Co<br>4 Examples; Yield: 1- 95% depending on the metal used<br>R = Tosyl, Bn, aryl, C(O)Ar<br>orthogonal reactivity between metal and azide | 242 |
| 11 | | Amination | 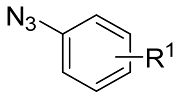 | 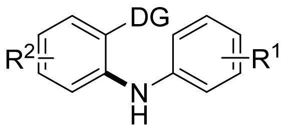 | Substrate (0.36 mmol), azide (0.2 mmol), $[RhCp^*Cl_2]_2$ (2.5 mol%), $AgSbF_6$ (10 mol%), DCE (0.5 mL), 85 °C, 18 h<br>19 Examples; Yield: 45-97%<br>$R^1$ = $NO_2$, $CF_3$, $SO_2Me$, COOR, C(O)Me, Cl<br>$R^2$ = OMe, Me, COOMe, Halogen, $CH_2OH$, $CH_2OAc$ | 225 |
| 12 | | Amination | $N_3R^2$ | 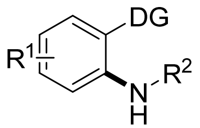 | Substrate (0.2 mmol), azide (0.4 mmol), $[RhCp^*Cl_2]_2$ (4 mol%), $AgSbF_6$ (16 mol%), DCE (0.5 mL), 110 °C, 24 h<br>25 Examples; Yield: 45-94%<br>$R^1$ = $NO_2$, OMe, Me, Halogen, CHO, $CH_2OAc$<br>$R^2$ = Alkyl, Aryl | 243, 244 |
| 13 | | Arylation | 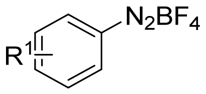 | 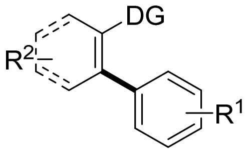 | Substrate (0.3 mmol), aryldiazonium salt (0.2 mmol), $[IrCp^*Cl_2]_2$ (5 mol%), $AgBF_4$ (20 mol%), NaOAc 30 mol%), $CF_3CH_2OH$ (1 mL), 35 °C, 12 h<br>21 Examples; Yield: 43-82%<br>$R^1$ = Halogen, $CF_3$, Me, C(O)Me, COOMe ; F, Cl and Br tolerated<br>$R^2$ = Me, OMe, $CF_3$, Br, OAc, | 226 |

| | | | | | | |
|---|---|---|---|---|---|---|
| 14 | 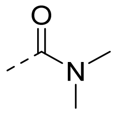 | Alkenylation | 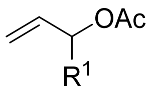 | 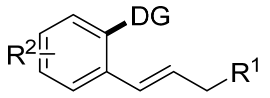 | Substrate (0.2 mmol), alkene (0.24 mmol), $[Cp^*RhCl_2]_2$ (0.0025 mmol), $AgSbF_6$ (0.01 mmol), DCE (1 mL), 16 h<br>22 Examples; Yield: generally above 50%<br>$R^1$ = Alkyl, aryl<br>$R^2$ = Me, OMe, TMS, $CF_3$, Ph, OH, halogen, COOR, C(O)R<br>With $R^1$ other than H, mixtures of isomers are formed | 245 |
| 15 | | Alkenylation | 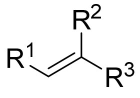 | 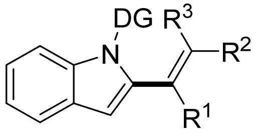 | Substrate (1 equiv), alkene (2 equiv), $[Ru(p\text{-cymene})Cl_2]_2$ (2.5 mol%), $AgSbF_6$ (10 mol%), $Cu(OAc)_2{\cdot}H_2O$ (1 equiv), dioxane (0.15 M), 100 °C, 24 h<br>25 Examples; Yield: generally above 50%,<br>$R^1$ = Alkyl, Ph<br>$R^2$ = Alkyl<br>$R^3$ = Aryl, COOR, $SO_2Ph$, $PO(OEt)_2$, CN<br>Applicable also to monosubstitution of pyrrole<br>Electron rich reaction partners generally preferred | 246 |
| 16 | | Alkenylation | 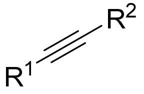 | 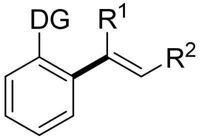 | Substrate (0.25 mmol), alkyne (0.5 mmol), $[RuCl_2(p\text{-}cymene)]_2$ (0.0.125 mmol), $AgSbF_6$ (0.05 mmol), AcOH (1 mmol), dioxane (3 mL), 100 °C, 5 h, $N_2$<br>8 Examples; Yield: 19-47%<br>$R^1$ = Me, Bu, Ph, H<br>$R^2$ = Ph, $Si^iPr_3$ | 247, 248 |
| 17 | | Asymmetric Intramolecular Direct Hydroarylation | 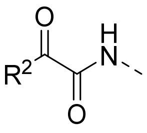 | 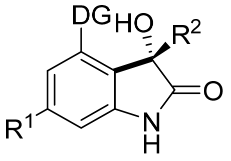 | Substrate (0.25 mmol), $[Ir(cod)_2](BAr^F{}_4)$ (5 mol%), (*R,R*)-Me-BIPAM (1.1 equiv), DMF (1 mL), 135 °C, 16 h<br>24 Examples; Yield: 66-99%<br>ee typically >90%<br>$R^1$ = Me, $CF_3$, Cl<br>$R^2$ = Aryl, alkyl | 228, 229 |

| | | | | | | |
|---|---|---|---|---|---|---|
| 18 | 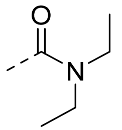 | Arylation | 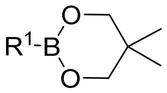 | 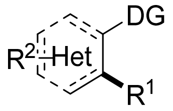 | Substrate (1 equiv), ArBneop (1.5 equiv), $RuH_2(CO)(PPh_3)_3$ (4 mol%), toluene, 125-135 °C, 24-44 h<br>18 Examples; Yield: 18-90%<br>$R^1$ = Aryl, heteroaryl<br>$R^2$ = Alkyl, Ph, OMe<br>Het = O, N, S | 249 |
| 19 | | Borylation | $B_2pin_2$ | 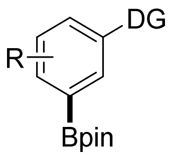 | Substrate (1 equiv), $[Ir(cod)(OMe)]_2$ (2 mol%), dtbpy (4 mol%), $B_2pin_2$ (0.6 equiv), hexanes, 80 °C, 18 h<br>8 Examples; Yield: 32-86%<br>R = TMS, halogen, OMe,<br>Substitution in meta position<br>If a *o*-TMS is present, substitution in para position | 230 |
| 20 | | Trifluoromethylation | 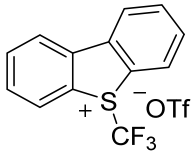 | 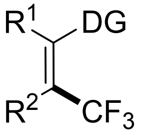 | Substrate (0.3 mmol), CuI (1.1 equiv), TFA (10 equiv), n-methylformamide (15 equiv), DCE (9 mL), air, 120 °C, 16 h<br>8 Examples; Yield: 11 – 69%<br>$R^1$ = Aryl, $^n$Bu<br>$R^2$ = Ph, H | 250 |
| 21 | 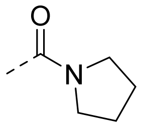 | Alkenylation | 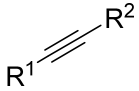 | 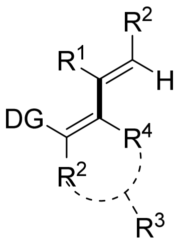 | Substrate (0.5 mmol), alkyne (0.55 mmol), $[Rh(cod)_2]BF_4$/BIPHEP (0.05 mmol), DCM (1 mL), 25 °C, 18-72 h<br>15 Examples; Yield: 20-93%<br>$R^1$, $R^2$ = Alkyne, aryl, alkyl<br>$R^3$ = OMe, $CF_3$<br>$R^2$, $R^4$ = Me, $-(CH_2)_4-$<br>Very good *E*-selectivity | 251 |

| | | | | | | |
|---|---|---|---|---|---|---|
| 22 | | Amination | 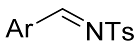 | 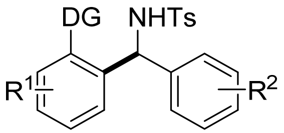 | Substrate (1 equiv), tosylimine (1.5 equiv), $[Cp^*RhCl_2]_2$ (2.5 mol%), $AgB(C_6F_5)_4$ (10 mol%), DCE (0.75 M), 75 °C, 20 h<br>16 Examples; Yield: 36-94%<br>$R^1$ = OMe, $CF_3$, C(O)Me, Br, Me<br>$R^2$ = $NO_2$, COOMe, $CF_3$, CN, halogen, Me; thiophene tolerated | 252 |
| 23 | 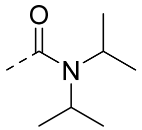 | Alkenylation | 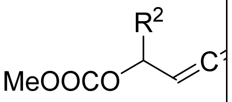 | 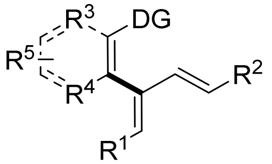 | Substrate (0.4 mmol), allenyl carbinol carbonate (0.8 mmol), $[Cp^*Rh(MeCN)_3](SbF_6)_2$ (5 mol%), $Cu(OAc)_2$ (15 mol%), PivOH (1 equiv), DCM (2 mL), 60 °C, 3-24 h<br>27 Examples; Yield: 30-87%<br>$R^1$ = Alkyl<br>$R^2$ = Alkyl, Ph<br>$R^3$ = CH; Ph, Me<br>$R^4$ = CH; Aryl, Br, COOEt<br>$R^5$ = $CF_3$, halogen, Me, CHO, OMe, COOMe | 232 |
| 24 | | Alkynylation | 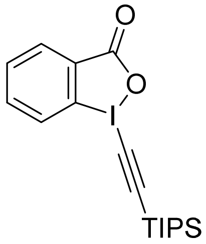 | 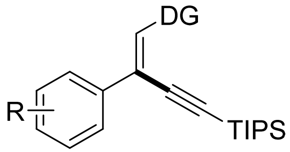 | Substrate (0.2 mmol), hypervalent alkynyl iodine reagent (2 equiv), $RhCp^*(MeCN)_3(SbF_6)_2$ (10 mol%), DCM (1.5 mL), 80 °C, 16 h<br>9 Examples; Yield: 38-92%<br>R = OMe, $NO_2$, Halogen, Me<br>*ortho* alkynylation of benzamides also possible | 253 |
| 25 | | Allylation | allyl carbonate | 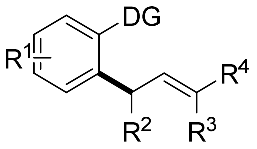 | Substrate (0.4 mmol), allyl carbonate (0.8 mmol), [[254]$_2$] (2.5 mol%), $AgSbF_6$ (30 mol%), PivOH (1 equiv), PhCl (2 mL), 35-50 °C, 18 h<br>20 Examples; Yield: 42-84%<br>$R^1$ = Me, OMe, Br, CHO, COOMe, $CF_3$<br>$R^2$ = $R^3$ = $-(CH_2)_3-$ | 255 |

| | | | | | | |
|---|---|---|---|---|---|---|
| | | | | | $R_2$ = H<br>$R_3$ = $R_4$ = Me<br>Allylation of electron-neutral arenes | |
| 26 | | Cyanation | 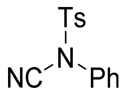 | 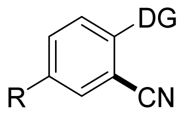 | Substrate (0.5 mmol), *N*-cyano-*N*-phenyl-*p*-toluenesulfonamide (1 mmol), $[RuCl_2(p\text{-cymene})]_2$ (5 mol%), $AgSbF_6$ (20 mol%), NaOAc (20 mol%), DCE (2 mL), 120 °C, 24 h<br>15 Examples; Yield: generally above 50%<br>R = Me, OMe, COOMe, Ph, halogen; thiophene, furan and indole applicable | 93 |
| 27 | | Halogenation | NXS<br>X = I, Br | 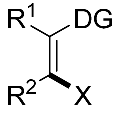 | Substrate (0.4 mmol), NXS (1.1 equiv), $[RhCp^*Cl_2]_2$ (2.5 mol%), $AgSbF_6$ (10 mol%), PivOH (1.1 equiv), DCE, 60 °C, 16 h<br>23 Examples; Yield: 39-93%<br>$R^1$ = Alkyl, Ph, Br<br>$R^2$ = Aryl, Me<br>*Z* selective | 231 |
| 28 | 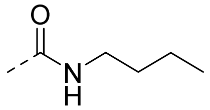 | ß-Acyloxylation | $CF_3COOH$ | 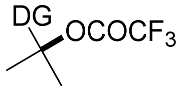 | Substrate (1 mmol), $CF_3COOH$ (5 mmol), $Pd(OAc)_2$ (0.1 mmol), $K_2S_2O_8$ (2 mmol), 80 °C, 20 h<br>1 Example, 91% | 256 |
| 29 | 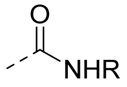 | Alkylation | 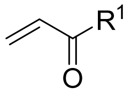 | 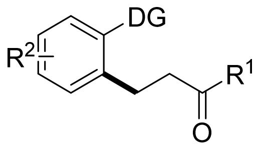 | Substrate (0.5 mmol), alkene (1 mmol), $[RuCl_2(p\text{-cymene})]_2$ (5 mol%), $KO_2CMes$ (30 mol%), $MesCO_2H$ (1 equiv), $H_2O$, $N_2$, 120 °C, 20 h<br>20 Examples; Yield: 45-81%<br>$R^1$ = Alkyl<br>$R^2$ = Halogen, OMe, $CF_3$, Ph<br>Combination with oxidative alkene annulation towards | 257 |

| | | | | | | |
|---|---|---|---|---|---|---|
| | | | | | chinolin derivatives also reportet | |
| 30 | | Alkylation | 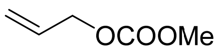 | 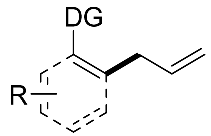 | Substrate(0.4 mmol), alkene (2 equiv), $[Cp^*CoI_2]_2$ (2.5 mol%), $AgBF_4$ (40 mol%), AcOH (40 mol%), TFE, 60 °C, 16 h<br>18 Examples; Yield: 22-81%<br>R = Alkyl, Bn, OMe, $NO_2$, halogen<br>Also alkenylamides as substrates applicable | 258 |
| 31 | 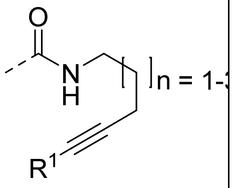 | Intramolecular Annulation | | 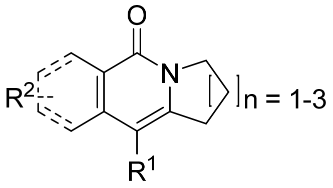 | Substrate (0.25 mmol), $[RhCp^*Cl_2]_2$ (2.5 mol%), $Cu(OAc)_2$ (0.5 mmol), *t*-AmOH (2 mL), 110 °C<br>20 Examples; Yield: generally above 60%<br>$R^1$ = Aryl, Me<br>$R^2$ = OMe, $CF_3$, Br<br>$R^1$ cannot be H | 259 |
| 32 | 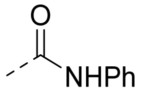 | Heteroarylation | Heteroarene | 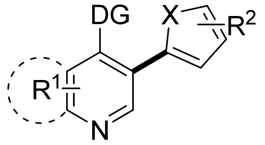 | Substrate (0.2 mmol), heteroarene (0.6 mmol), $[RhCp^*Cl_2]_2$ (1.5 mol%], $Cu(OAc)_2$ (2 equiv), $K_2HPO_4$ (1.5 equiv), dioxane (1 mL), 130 °C, 24 h, $N_2$<br>25 Examples; Yield: 35-91%<br>$R^1$ = Halogen, OMe<br>$R^2$ = Alkyl, halogen, COOEt, CN<br>X = S, O<br>Many alternatives to *N*-Ph investigated with generally very bad yields | 260 |
| 33 | 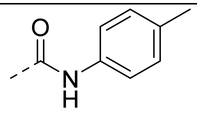 | Alkenylation | 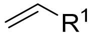 | 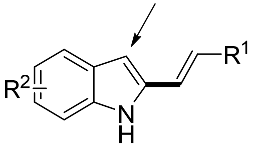 | Substrate (0.3 mmol), olefin (0.6 mmol), $[RhCp^*Cl_2]_2$ (2.5 mol%), $AgSbF_6$ (10 mol%), $Cu(OAc)_2 \cdot H_2O$ (200 mol%), *t*-AmOH, 100 °C, 20 h<br>23 Examples; Yield: 25-84%<br>$R^1$ = COOR, $SO_2Ph$, $PO(OEt)_2$, $CONMe_2$ | 261 |

| | | | | | | |
|---|---|---|---|---|---|---|
| | | | | | $R^2$ = OMe, $NO_2$, CN, halogen, Me | |
| 34 | 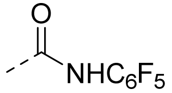 | Alkenylation | 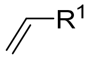 | 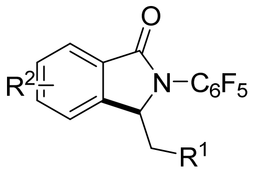<br>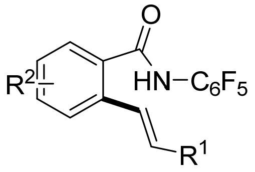 | Substrate (0.2 mmol), alkene (0.5 mmol), $[RhCp^*Cl_2]_2$ (0.01 mmol), NaOPiv (0.2 mmol), MeCN (2 mL), air, 80 °C, 24 h<br>25 Examples; Yield: generally above 50%<br>$R^1$ = Aryl, COOR, CN<br>$R^2$ = Me, OAc, halogen, C(O)Me<br>If $R^1$ = Aryl, no cyclization occurs | [262] |
| 35 | | Arylation | ArI | 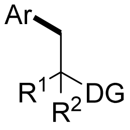 | Substrate (0.2 mmol), aryl iodide (3 equiv), $Pd(OAc)_2$ (10 mol%), ligand (20 mol%), CsF (3 equiv), 3 Å MS (100 mg), toluene (1 mL), 100 °C, $N_2$, 24 h<br>11 Examples; Yield: 30-84% (yield of mono-arylated product)<br>$R^1$ = Me, H<br>$R^2$ = Alkyl, aryl | [263, 264] |
| 36 | 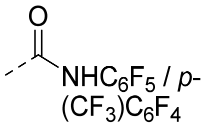 | Alkenylation / Intramolecular Cyclisation | 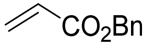 | 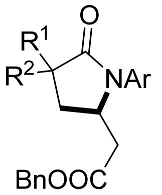 | Substrate (0.2 mmol), benzyl acrylate (0.1 mL), $Pd(OAc)_2$ (10 mol%), LiCl (2 equiv), $Cu(OAc)_2$ (1.1 equiv), AgOAc (1.1 equiv), DMF (1 mL), 120 °C, $N_2$, 12 h<br>16 Examples; Yield: 18-94%<br>$R^1$ = Me, H<br>$R^2$ = Alkyl, $CH_2OR$, $CH_2COOR$, Bn<br>β-C-H alkenylation followed by 1,4-conjugate addition towards lactam | [265] |
| 37 | 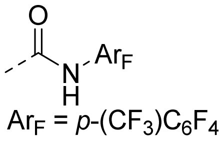 | Alkylation / Arylation | $R^1I$ | 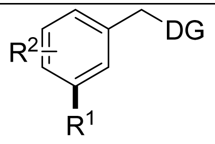 | Substrate (0.1 mmol), iodoarene (3 equiv), $Pd(OAc)_2$ (10 mol%), ligand (20 mol%), norbornene (3 equiv), AgOAc (3 equiv), $PhCF_3$ (1.5 mL), 90 °C, air, 24 h<br>8 Examples; Yield: 57-87% yield | [266, 267] |

| | | | | | | |
|---|---|---|---|---|---|---|
| | | | | | $R^1$ = Alkyl, aryl<br>$R^2$ = OMe, halogen, alkyl<br>Substrate ( 0.1 mmol), alkyliodide (2.5 equiv), $Pd(OAc)_2$ (10 mol%), ligand (10 mol%), norbornene (1.5 equiv), AgOAc (3 equiv), DCE (1.5 mL), 75 °C, air, 16 h<br>31 examples, 52-90% yield<br>$R^1$ = Alkyl, aryl<br>$R^2$ = OMe, halogen, alkyl<br>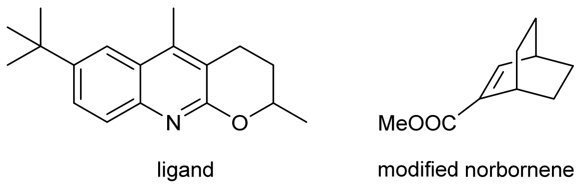<br><br>*meta* directing | |
| 38 | | Arylation (dual) | ArI | 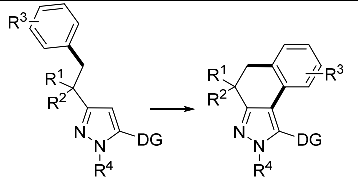<br> | Substrate (0.05 mmol), iodoarene (0.15 mmol), $Pd(OTf)_2(MeCN)_4$ (10 mol%), $Ag_2O$ (0.1 mmol), AcOH (0.5 mL), 120 °C, 24 h<br>27 Examples; Yield: 32-83%<br>$R^1$ = Alkyl<br>$R^2$ = Me<br>$R^3$ = Me, Ph, halogen, $PO(OEt)_2$, COOMe, OMe<br>$R^4$ = Alkyl | 268 |
| 39 | 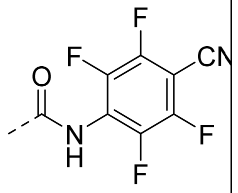<br> | Arylation | ArBPin | 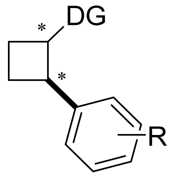<br> | Substrate (0.1 mmol), ArBPin (2 equiv), $Pd(OAc)_2$ (10 mol%), chiral ligand (11 mol%), $Ag_2CO_3$ (2.5 equiv), $Na_2CO_3$ (2 equiv), BQ (0.5 equiv), $H_2O$ (5 equiv), *t*-amylOH (0.5 mL), $N_2$, 70 °C, 24 h<br>18 Examples; Yield: generally above 50%, > 84% ee<br>R = Me, Halogen, OMe, NHAc, COOR | 233 |

| | | | | | | |
|---|---|---|---|---|---|---|
| | | | | | New class of amino acid derived ligands | |
| 40 | 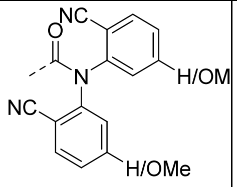  | Arylation | Ar-Bpin | 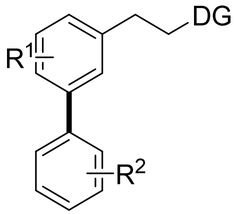  | Substrate (0.1 mmol), ArBpin (0.3 mmol), $Pd(OAc)_2$ (10 mol%), Ac-Gly-OH (20 mol%), $Ag_2CO_3$ (0.2 mmol), $TBAPF_6$ (0.3 mmol), CsF (0.2 mmol), HFIP (1 mL), 70 °C, 24 h<br>24 Examples; Yield: 44-85%<br>$R^1$ = Halogen, CF3, Me, OMe<br>$R^2$ = F, Me, OMe, $CF_3$ | 234 |
| 41 | 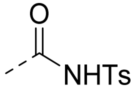  | Alkoxylation | $R^2OH$ | 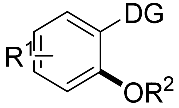  | Substrate (1 equiv), $PhI(OAc)_2$ (1 equiv), $Pd(OAc)_2$ (10 mol%), $R^2OH$, 25 °C<br>15 Examples; 47-95%<br>Methanol, ethanol and *i*-propanol investigated<br>$R^1$ = Halogen, Me, OMe, $NO_2$, $CF_3$, Ph | 174 |
| 42 | | Alkylation | 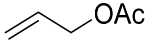  | 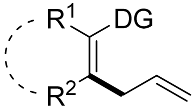  | Substrate (0.1 mmol), hypervalent alkynyl iodine reagent (0.11 mmol), NaOAc (0.1 mmol), $[Cp^*RhCl_2]_2$ (0.002 mmol), DCE (0.5 mL), 16 h, rt<br>18 Examples; Yield: 25-87%<br>$R^1$ = Aryl, Bn, alkyl<br>$R^2$ = Alkyl, Ph | 269 |
| 43 | | Alkynylation | 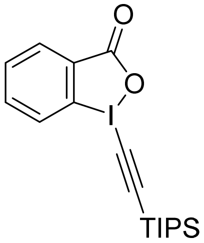  | 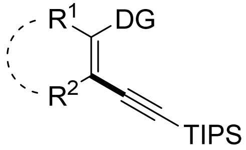  | Substrate (0.1 mmol), hypervalent alkynyl iodine reagent (0.11 mmol), NaOAc (0.1 mmol), $[Cp^*RhCl_2]_2$ (0.002 mmol), DCE (0.5 mL), 16 h, rt<br>R can be aromatic or aliphatic, yields generally above 50%<br>$R^1$ = Aryl, alkyl<br>$R^2$ = Aryl, alkyl | 270 |

| | | | | | | |
|---|---|---|---|---|---|---|
| 44 | | Arylation | ArI | 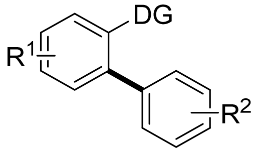 | Substrate (1 equiv), iodobenzene (2 equiv), AgOAc (2 equiv), $Pd(OAc)_2$ (10 mol%), acetic acid, 120 °C, sealed tube<br>23 Examples; Yield: 8-84%<br>$R^1$ = Me, OMe, halogen, $NO_2$, $CF_3$, Ph<br>$R^2$ = Me, $NO_2$, halogen, OMe, $CF_3$<br>Long reaction times (20-720 h) | 271 |
| 45 | | Halogenation | NXS | 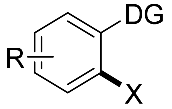 | Substrate (1 equiv), NXS (1.2 equiv), TFA (10 equiv), $Pd(OAc)_2$ (10 mol%), MeOH, 25 °C<br>Iodination, chlorination and bromination possible<br>14 Examples; Yield: generally above 50%<br>R = Halogen, Me, $CF_3$, $NO_2$<br>Alkoxylation as common side reaction | 174 |

### *N*-Methoxy amides as directing group

*N*-Methoxy amides are amongst the most widely used classes in amide based directing groups. A representative of particular interest is the Weinreb amide as it is an inherently valuable functional group. Wang et al. (Table 10, Entry 16)[272] presented a protocol for the direct *ortho* functionalization of aromatic Weinreb amides via Rh-catalysis. They were able to perform high yielding alkenylations in the presence of diverse functional groups including bromine in *meta* or *para*-position. This potentially allows for the orthogonal functionalization of different positions on the aromatic ring.

Li and coworkers[273] presented a protocol for the arylative cyclization of 1,6-enynes towards either tetracyclic isoquinolones or hydrobenzofurans depending on the nature of the directing group used(Table 10, Entry 7 & 13). *N*-(Pivaloyloxy)benzamides might be involved in chelating the Rh(III)-intermediate via the *O*-pivaloyl group thereby promoting C-N bond reductive elimination, in the case of *N*-methoxybenzamides, an additional chelating is not possible resulting in the rapid protonation of the seven-membered rhodacycle and subsequently the formation of the open vinyl-Rh(III) intermediate.

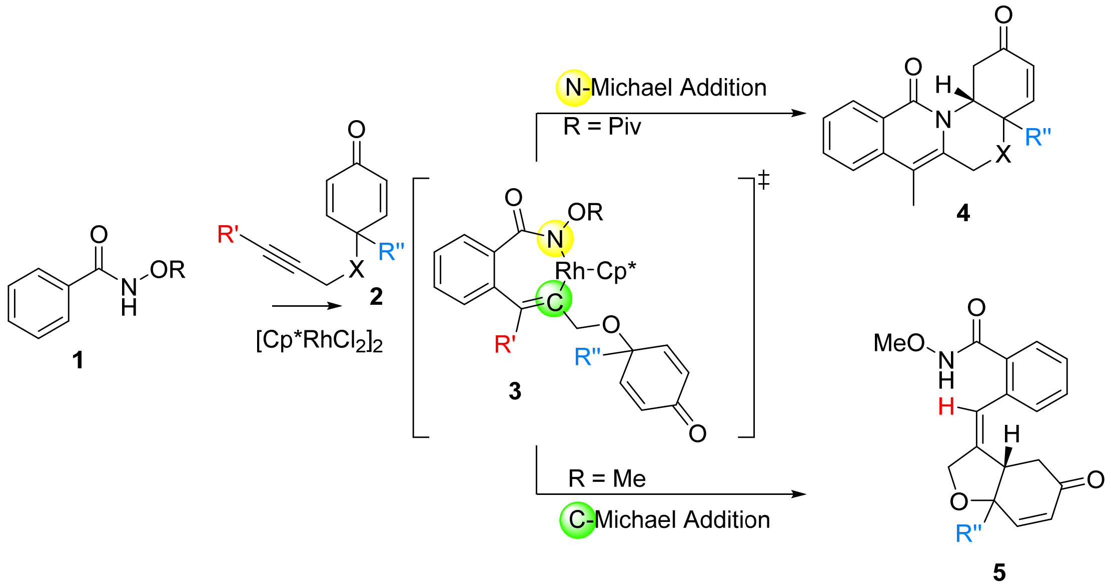


**Scheme 7: Formation of isoquinolines or hydrobenzofurans depending on the used directing group.**

The *N*-methoxybenzamide motif was also used in the intramolecular transformation towards dihydrobenzofurans(Table 10, Entry 8).[274] Starting from substrates of type **1**, two possible positions for C-H insertion can lead to either functionalization at the less hindered α' position or, less likely, at the sterically more demanding α-position. Taking advantage of the reversibility of the insertion-process, the overall equilibrium is driven towards the seven membered rhodacycle **3**. From here, reductive C($sp^3$)-*N* bond

formation or proto-demetalation yielding the desired **4** can occur. In the case of the C(O)-NHOMe directing group[275], the β-elimination towards **5** in usually observed with Rh-catalysis which could be successfully suppressed via addition of PivOH (1 equiv) as additive and good to excellent enantiomeric ratios were reached.

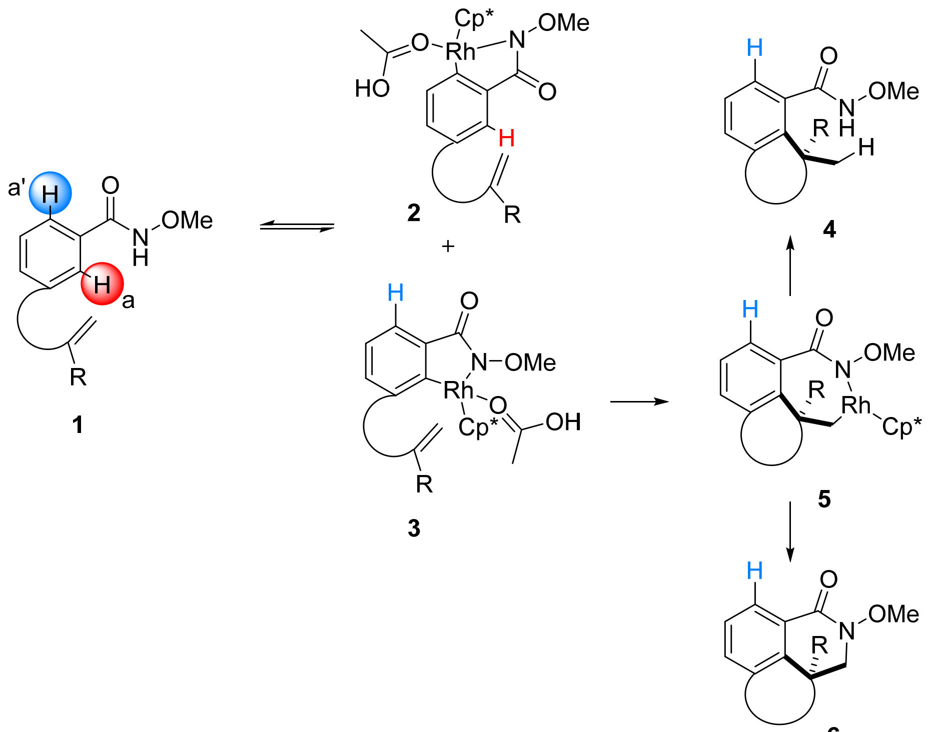


**Scheme 8: Mechanistic rationale for the formation of the cyclized product 6.**

The activation of C($sp^3$) carbon centers is usually a challenging task due to the inherently low activity of these centers. Yu and coworkers[276] investigated the stereoselective β-arylation of modified alanine (Table 10, Entry 10). Via a 2-step Pd-catalyzed protocol with extremely broad substrate scope, the β-position of the protected amino acid could be substituted with 2 different aryl groups. The process could be realized in synthetically useful yields over 2 steps with good diastereomeric ratios.

Geminal disubstituted allenylsilanes have been submitted to Ru-catalyzed aromatic C-H allenylation by Nakanowatari et al.(Table 10, Entry 9)[277]. Allenes are enourmosly versatile functional groups for further modification, their use in C-H activation is however fairly rare. Employing a Ru-catalyst and substituted *N*-methoxybenzamides, terminal allenes with various substituents could be connected to the *ortho* position of the aromatic substrates.

The utilization of diynes as coupling partners in alkenylation followed by intramolecular cyclization gives rise to the synthesis of unsymmetrical heterocyclic products. The Glorius group realized the coupling of diynes including unsymmetrical substrates and could thereby show the preparation of a number of bisheterocyclic compounds (Table 10, Entry 12).[278]

**Table 10: N-Methoxy amides as directing group**

| Entry | Directing group | Type of transformation | Coupling partner | Typical product structure | Comments | Ref |
|---|---|---|---|---|---|---|
| 1 | 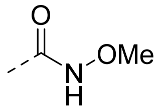 | Acylation | 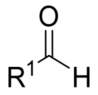 | 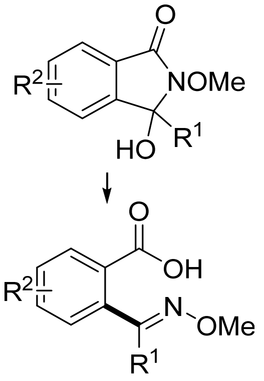 | Substrate (0.3 mmol), aldehyde (1.2 mmol), $Pd(OAc)_2$ (10 mol%), TBHP (70% / $H_2O$; 5 equiv), $BF_3{\cdot}Et_2O$, (0.4 equiv), DMSO/dioxane (4/1; 0.2 M), 130 °C, 1.5-3 h<br>13 Examples; Yield: 51-72%<br>$R^1$ = Aryl<br>$R^2$ = Halogen, OMe, Me | 279 |
| 2 | | Alkenylation | 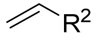 | 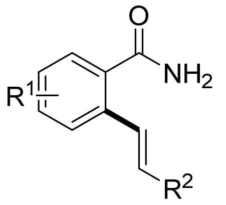 | Substrate (1 equiv), $[Cp*RhCl_2]_2$ (1 mol%), CsOAc (30 mol%), MeOH (0.2 M), 60 °C, 3-16 h<br>35 Examples; Yield: 40-99%<br>$R^1$ = $NO_2$, Ac, COOMe, halogen, Ph, OMe; thiophene tolerated<br>$R^2$ = Aryl, heteroaryl, COOR<br>*N*-Methoxy group cleaved during reaction | 280 |
| 3 | | Alkenylation | 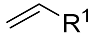 | 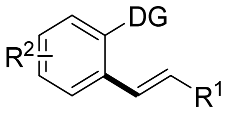 | Substrate (1 equiv), alkene (1.8 equiv), $[RuCl_2(p\text{-cymene})]_2$ (5 mol%), NaOAc (30 mol%), MeOH (0.2 M), 60 °C, 4-24 h<br>19 Examples; Yield: 45-95%<br>$R^1$ = COOR<br>$R^2$ = Alkyl, OMe, halogen, $NO_2$, $CF_3$, OAc, COOMe; thiophene and indole tolerated | 281 |

| | | | | | | |
|---|---|---|---|---|---|---|
| 4 | | Alkenylation / Cyclization | 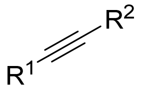  | 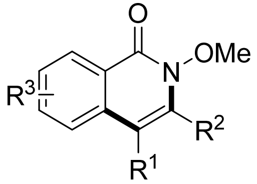  | Substrate (0.3 mmol), alkyne (0.9 mmol), 5% Pd/C (10 mol%), NaI·$2H_2O$ (0.15 mmol), $Na_2CO_3$ (0.3 mmol), DMF (1 mL), 48 h, air<br>17 Examples; Yield: 22-92%<br>$R^1$ = Alkyl, aryl, F<br>$R^2$ = Alkyl, aryl, F; in most cases $R^1$=$R^2$<br>$R^3$ = Alkyl, OMe, Cl | 282 |
| 5 | | Alkenylation and Intramolecular Cyclization | 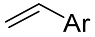  | 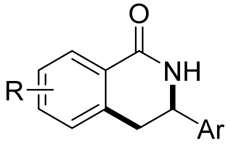  | Substrate (1 equiv), alkene (2 equiv), $[RuCl_2(p\text{-cymene})]_2$ (10 mol%), NaOAc (200 mol%), $CF_3CH_2OH$ (0.25 M), 50 °C, 24-36 h<br>10 Examples; Yield: generally above 50%<br>R = Me, OMe | 281 |
| 6 | | Alkoxylation | Alcohol | 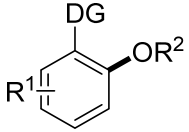  | Substrate (0.25 mmol), $Pd(OAc)_2$ (0.0125 mmol), $K_2S_2O_8$ (0.5 mmol), 4 Ǻ MS (30 mg), alcohol (2 mL), dioxane (2 mL), 55 °C<br>21 Examples, 20-79% yield<br>$R^1$ = Me, Halogen, $NO_2$, COOR<br>$R^2$ = Alkyl | 283 |
| 7 | | Alkynylation/Arylative Cyclization | 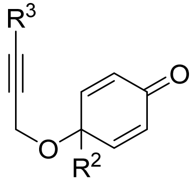  | 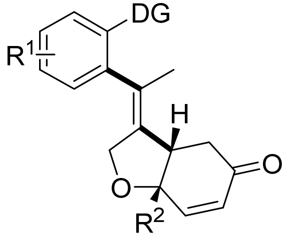  | Substrate (0.3 mmol), 1,6 enyne (0.2 mmol), $[Cp^*RhCl_2]_2$ (0.005 mmol), CsOPiv (2 equiv), PivOH (2 equiv), DCE (2 mL), 60 °C, 12 h<br>20 Examples; Yield: generally above 50%<br>$R^1$ = F, Br, OMe, $CF_3$, $NO_2$, COOMe, Me<br>$R^2$ = Alkyl, | 273 |
| 8 | | Intramolecular Alkylation | 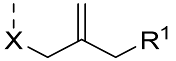  | 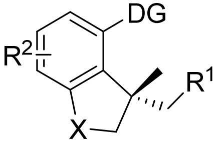  | Substrate (0.1 mmol), PivOH (0.1 mmol), chiral Rh-catalyst (5 µmol), $(Bz)_2O$ (5 µmol), DCM (0.2 M), 23 °C, 12 h<br>15 Examples; Yield: above 50%, e.r. >92:8 | 274, 284 |

| | | | | | | |
|---|---|---|---|---|---|---|
| | | | | | $R^1$ = OR, OH, Ph<br>$R^2$ = OMe, OH, $NO_2$, Alkyl, Br<br>X = O, NMe | |
| 9 | | Allenylation | 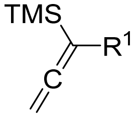 | 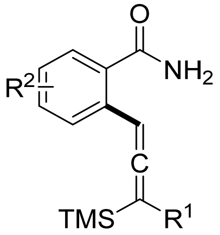 | Substrate (0.5 mmol), Allene (0.53 mmol), $[RuCl_2(p\text{-cymene})]_2$ (5 mol%), NaOAc (30 mol%), MeOH 83 mL), 22 °C, 18 h, $N_2$<br>22 Examples; Yield: 12-75%<br>*N*-OMe cleaved during reaction<br>$R^1$ = Alkyl<br>$R^2$ = Me, OMe, Halogen, $CF_3$, Ph | 277 |
| 10 | | Arylation | ArI | 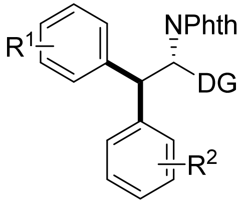 | Sequential process – **$R^1$**<br>Substrate (0.1 mmol), ArI (0.15 mmol), $Pd(OAc)_2$ (10 mol%), AgOAc (0.2 mmol), 2-picoline (20 mol%), HFIP (1 mL), 75 °C, 24 h<br>46 Examples, 42-94%<br>$R^1$ = Halogen, Me, OMe, Ph, C(O)Me, COOR,<br>NHAc, $PO(OEt)_2$, $CH_2OH$<br>Heteroarene iodide susceptible to reaction conditions<br>- **$R^2$**<br>Substituted substrate (0.1 mmol), ArI (0.3 mmol), $Pd(OAc)_2$ (10 mol%), AgOAc (0.2 mmol), 2,6-lutidine (20 mol%), $NaHPO_4 \cdot H_2O$ (0.3 mmol), HFIP (1 mL), 100 °C, 36 h<br>12 Examples, > 50% yield<br>$R^2$ = Me, $CF_3$, COOR | 276 |

| | | | | | | |
|---|---|---|---|---|---|---|
| 11 | | Isocyanade Insertion | $R^1$-NC | 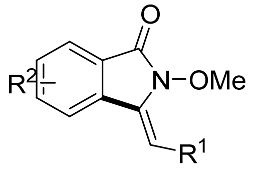<br> | Substrate (0.5 mmol), isocyanide (0.75 mmol), $Pd(OAc)_2$ (0.05 mmol), $O_2$ (balloon), $Cs_2CO_3$ (0.75 mmol), toluene, 90 °C, 16 h<br>17 Examples, 45-91% yield<br>$R^1$ = $^t$Bu, $^i$Pr<br>$R^2$ = Halogen, Me, OMe, COOMe | [285] |
| 12 | 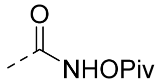<br> | Dual Alkenylation / Cyclization | 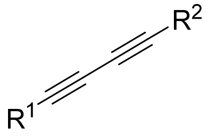<br> | 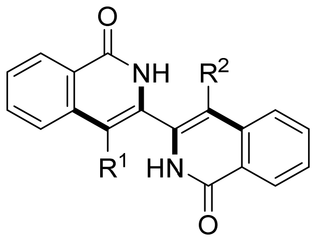<br> | Substrate (0.6 mmol), diyne (0.25 mmol), [${}^{254}{}_{2}$] (2 mol%), NaOAc (0.6 equiv), MeOH, 40 °C, air, 9 h<br>6 Examples, 67-80% yield<br>$R^1$ = Alkyl, aryl, TMS<br>$R^2$ = Alkyl, aryl, TMS<br>Procedure applicable for the synthesis of unsymmetrical compounds | [278] |
| 13 | | Alkynylation/Arylative Cyclization | 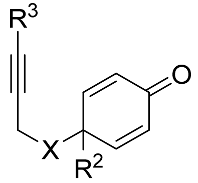<br> | 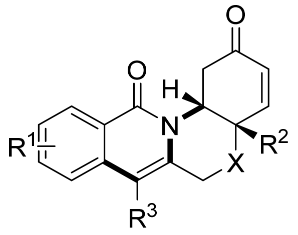<br> | Substrate (1.5 equiv), 1,6 enyne (0.2 mmol), $[Cp^*RhCl_2]_2$ (0.005 mmol), CsOAc (2 equiv), acetone (1 mL), 50 °C<br>20 Examples; Yield: generally above 50%<br>$R^1$ = Me, $CF_3$, OMe, F, Br<br>$R^2$ = Alkyl<br>$R^3$ = Alkyl | [273] |
| 14 | | Alkynylation / Intramolecular Cyclization | 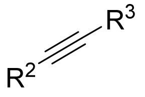<br> | 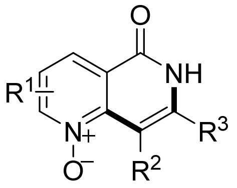<br> | Substrate (1 equiv), alkyne (1.1 equiv), NaOAc (0.5 equiv), $[Cp^*RhCl_2]_2$ (1 mol%), MeOH, 20 °C<br>16 examples, 1 example below 50%<br>$R^1$ = Br, OH, OMEM, $CF_3$, NHAc, COOMe<br>$R^2$ = H, Alkyl<br>$R^3$ = Alkyl | [275] |

| | | | | | | |
|---|---|---|---|---|---|---|
| 15 | | Amination | *N*-Chloroamine | 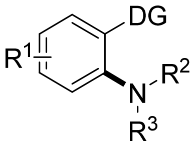 | Substrate (1 mmol), *N*-chloroamine (2 mmol), $[Cp^{*}RhCl_2]_2$ (0.05 mmol), CsOAc (2 mmol), PivOH (0.5 mmol), MeOH (5 mL), 16 h, rt<br>19 examples, only secondary, mostly cyclic amines investigated, yields between 30 and 85%<br>$R^1$ = Alkyl, Ph, COOMe, OMe, halogen, $CF_3$<br>$R_2 – R_3$ = Alkyl | 286 |
| 16 | 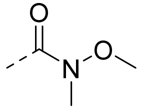 | Alkenylation | 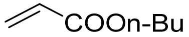 | 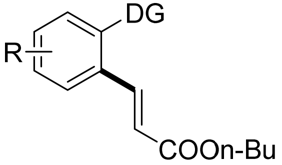 | Weinreb amide (0.2 mmol), alkene (0.3 mmol), $[Cp^{*}RhCl_2]_2$ (1 mol%), $AgSbF_6$ (4 mol%), $Cu(OAc)_2$ (20 mol%), DCE (0.7 mL), 120 °C, 16 h<br>18 Examples, 38-98% yield<br>R = Halogen, Me, $NO_2$, CN, Ph, OAc, OMe | 272 |

**N-Acyl- containing directing groups**

*N*-Acyl –substituents are commonly used as directing groups which is also due to the activating effect of this motif. A protocol for the mild *ortho* acylation of *N*-acetanilides was presented by Szabo et al. (Table 11, Entry 1)[287] Aromatic as well as aliphatic aldehydes could be directly coupled to numerous *N*-acetanilides with good functional group tolerance due to the very mild conditions. The reaction could be conducted under air in aqueous media. Under more forcing conditions (100°C in DMSO), the same class of products could be prepared starting from toluene derivatives by Yin and Sun (Table 11, Entry 2).[288] TBHP (4 equiv) serves as external oxidant producing the reactive acyl radical which adds to the palladacycle formed between Pd(II) and the acetanilide.

The tendency of *N-O* bonds to be cleaved during oxidizing coupling reactions was the rationale in the *ortho* alkenylation of *N*-phenoxyacetamides(Table 11, Entry 3).[289] *Via* the choice of solvent the reaction outcome could be controlled either towards the formation of benzofuran-derivatives or *ortho*-hydroxyphenyl-substituted derivatives (Scheme 9). The transformation showed good selectivity and a broad substrate scope.

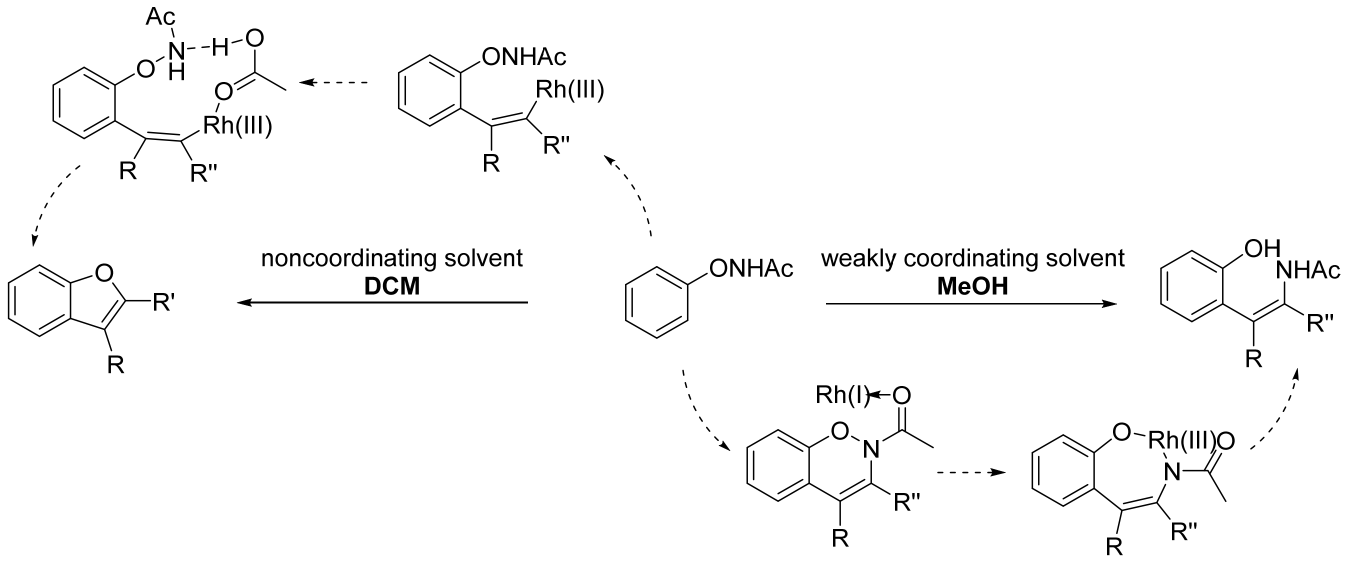


**Scheme: 9: Formation of benzofurane- or *ortho*-hydroxyphenyl- derivatives depending on the applied solvent.**

**Table 11: *N*-Acyl- containing directing groups**

| Entry | Directing group | Type of transformation | Coupling partner | Typical product structure | Comments | Ref |
|---|---|---|---|---|---|---|
| 1 | 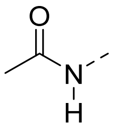  | Acylation | 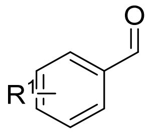  | 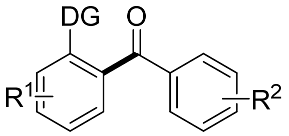  | Substrate (1 mmol), benzaldehyde (2 mmol), $Pd(OAc)_2$ (0.05 mmol), TFA (0.26 mmol), TBHP (2 mmol, 70 w% in water), rt, 24 h<br>20 examples, 32-86% yield<br>$R^1$ = Halogen, alkyl, OMe,<br>$R^2$ = Halogen, OMe<br>- Aqueous conditions<br>- Aliphatic aldehyde applicable | 287 |
| 2 | | Acylation | 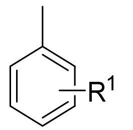  | 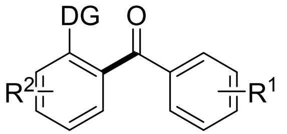  | Substrate (0.5 mmol), toluene derivative (1 mmol), $Pd(OAc)_2$ (5 mol%), TBHP (2 mmol), DMSO (1 mL), 100 °C, air, 20 h<br>28 examples, 9-93% yield<br>$R^1$ = Halogen, Me, OMe<br>$R^2$ = Me, OMe, $NO_2$, halogen<br>Formation of acyl-radical via benzylic oxidation | 288 |
| 3 | | Alkenylation/Intramolecular Cyclization | 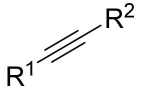  | MeOH 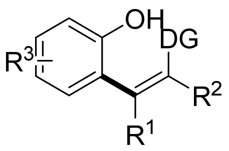  DCM 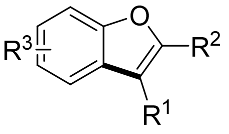  | Substrate (0.24 mmol), alkyne (0.2 mmol), $[(Cp^*RhCl_2)_2]$ (2.5 mol%), CsOAc (0.25 equiv), HOAc (1.2 equiv), MeOH (0.5 mL) or DCM (0.5 mL), 12-48h<br>29 examples in total, 45-90% yield<br>$R^1$ = Aryl, COOR,<br>$R^2$ = Ph, Alkyl<br>$R^3$ = Me, $CF_3$, F<br>- 2 mechanistic pathways proposed according to solvent | 289 |

| | | | | | | |
|---|---|---|---|---|---|---|
| 4 | | Alkenylation / Cyclization | 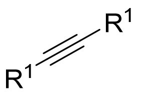 | 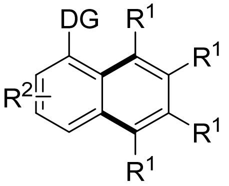 | Substrate (0.3 mmol), alkyne (0.63 mmol), $Pd(OAc)_2$ (5 mol%), TsOH (0.15 mmol), $K_2S_2O_8$ (0.6 mmol), toluene (1.5 mL), 16 h<br>12 Examples, 55-93% yield<br>$R^1$ = Aryl<br>$R^2$ = Me | 290 |
| 5 | | Alkoxylation | ROH | 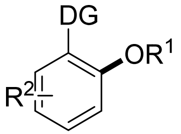 | Substrate (0.3 mmol), $R^1OH$ (10-50 equiv), $Pd(OAc)_2$ (0.03 mmol), $K_2S_2O_8$ (0.6 mmol), $MeSO_3H$ (0.06 mmol), DME (2 mL), rt, 24 h<br>22 Examples, 38-77% yield<br>$R^1$ = Alkyl<br>$R^2$ = Me, Cl, C(O)CH3 | 291 |
| 6 | | Arylation | $ArB(OH)_2$ | 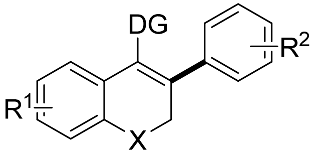 | Substrate (1 equiv), $ArB(OH)_2$ (2 equiv), $Pd(OAc)_2$ (10 mol%, $Cu(OTf)_2$ (2 equiv), $K_2CO_3$ (2 equiv), dioxane, 80 °C, 16 h<br>19 Examples, 57-90% yield<br>$R^1$ = OMe, Me, halogen<br>$R^2$ = Me, Cl, $NO_2$, OMe<br>X = $CH_2$, O | 292, 293 |
| 7 | | Trifluoromethylat ion | 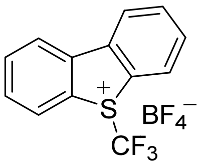Umemoto's reagen | 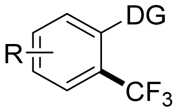 | Substrate (0.1 mmol), Umemoto's reagent (0.15 mmol), $Pd(OAc)_2$ (10 mol%), $Cu(OAc)_2$ (0.22 mmol), PivOH (0.5 mmol), DCE (1 mL), $N_2$, 110 °C, 24 h<br>19 examples, 41-83% yield<br>R = Alkyl, halogen, COOR, C(O)R, OAc, Ph<br>*m*-OMe and *m*-$CF_3$ not tolerated | 294 |

| | | | | | | |
|---|---|---|---|---|---|---|
| 8 | 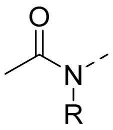 | Acetoxylation | AcOH | 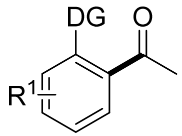 | Substrate (1 mmol), $Pd(OAc)_2$ (0.05 mmol), $K_2S_2O_8$ (2 mmol), AcOH (5 mL), DCE (5 mL), 100 °C, 48 h<br>13 Examples, 22-93% yield<br>$R^1$ = Me, OMe, halogen, $C(O)CH_3$ | 295 |
| 9 | | Alkenylation | 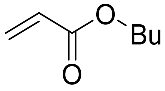 | 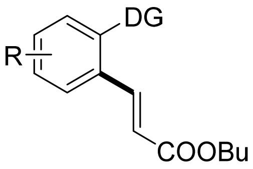 | Substrate (3 mmol), *n*-butyl acrylate (3.3 mmol), $Pd(OAc)_2$ (0.06 mmol), BQ (3 mmol), AcOH/toluene, TsOH (1.5 mmol), 20 °C<br>9 Examples, 30-91% yield<br>R = Me, OMe, $CF_3$ | 296 |
| 10 | | Arylation | $ArB(OH)_2$ | 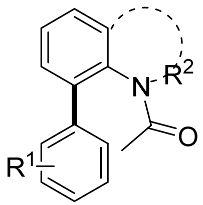 | Substrate (0.2 mmol), boronic acid (0.4 mmol), $Pd(OAc)_2$ (5 mol%), $Cu(OTf)_2$ (1 equiv), $Ag_2O$ (1 equiv), toluene (4 mL), 120 °C<br>24 Examples, 20-92% yield<br>$R^1$ = Me, OMe, Ph, F, $NO_2$<br>$R^2$ = Me,-$(CH_2)_5$-,-$(CH_2)_6$- | 297 |
| 11 | 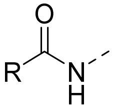 | Oxidative Alkenylation | 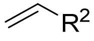 | 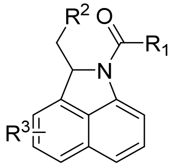 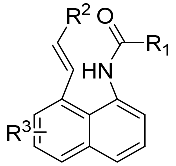 | Substrate (0.54 mmol), acrylate (1.08 mmol), $[RhCp^*Cl_2]_2$ (0.0216 mmol), $Ag_2CO_3$ (1.08 mmol), $CH_3CN$ (3 mL), 115 °C, 16 h, $N_2$<br>16 Examples, 33-89% yield<br>$R^1$ = Alkyl<br>$R^2$ = COOR, aryl<br>$R^3$ = OMe, Br<br>If $R^2$ is an EWG, cyclization occurs | 298 |
| 12 | 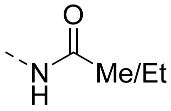 | Arylation | ArI | 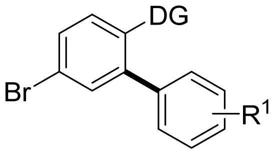 | Substrate (0.2 mmol), iodoarene (1.1 equiv), AgOAc (1.5 equiv), $K_3PO_4$ (3 equiv), TFA (0.1 mmol), DCE (2 mL), 90 °C<br>20 Examples; Protocol for one-pot C-H activation and Suzuki | 299 |

| | | | | | | |
|---|---|---|---|---|---|---|
| | | | | | coupling, 65-97% yield<br>$R^1$ = OMe, Me, Cl | |
| 13 | 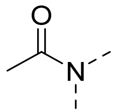 | Arylation | Arene | 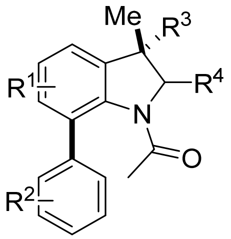 | Substrate (1 equiv), arene (solvent, 0.2 M), $Pd(OAc)_2$ (20 mol%), $Na_2S_2O_8$ (3 equiv), TFA (5 equiv), 100 °C or 120 °C<br>21 examples, yields generally above 50 %<br>$R_1$ = Me, OMe, F<br>$R_2$ = Me, Ph, OMe, OEt<br>$R_3$ = $R_4$ = -(CH2)4-<br>$R_3$ = Me,<br>$R_4$ = Me,<br>- Dehydrogenerative C-H/C-H crosscoupling<br>- Substituent at C2 ($R^4$) required | 300 |
| 14 | 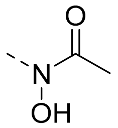 | Alkenylation | 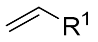 | 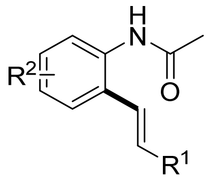 | Substrate (0.237 mmol), alkene (1.2 equiv), $[RhCp^*Cl_2]_2$ (3 mol%), $AgSbF_6$ (12 mol%), NaOAc (2 equiv), dioxane (2 mL), 100 °C, 8 h<br>19 Examples, 48-81% yield<br>$R^1$ = COOR, aryl<br>$R^2$ = Me, OMe, halogen, CN, COOR, CHO, C(O)Me, OH<br>- *N*-OH is removed during reaction | 301 |
| 15 | 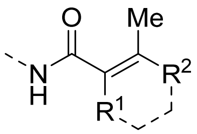 | Intramolecular Cyclization | | 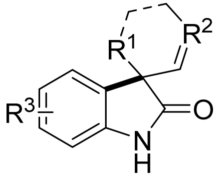 | Substrate (1 equiv), $Pd(OAc)_2$ (2 mol%), methyl nicotinate (8 mol%), mesitylene/*t*BuCOOH (4/1, 0.1 M), $O_2$, 24-230 h<br>12 Examples, 24-68% yield<br>$R^1$ = Me, Ph<br>$R^2$ = Me<br>$R^3$ = OMe, Me, $NMe_2$ | 302 |

| | | | | | | |
|---|---|---|---|---|---|---|
| 16 | 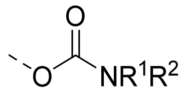 | Amidation | tosyl azide | 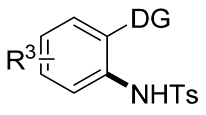 | Substrate (0.36 mmol), tosyl azide (0.2 mmol), $[IrCp^{*}Cl_2]_2$ (4 mol%), $AgNTf_2$ (16 mol%), $Cu(OAc)_2$ (10 mol%), DCE (0.5 mL), 50 °C, 12 h<br>8 Examples, $R_1$ and $R_2$ = Me or Et, yields above 50%<br>$R_3$ = Me, OMe, $CF_3$, $NO_2$, halogen, COOMe, $CH_2OR$, | 241 |
| 17 | 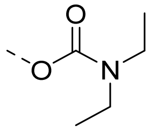 | Borylation | $B_2pin_2$ | 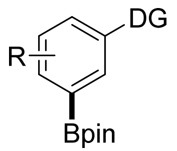 | Substrate (1 equiv), $[Ir(cod)(OMe)]_2$ (2 mol%), dtbpy (4 mol%), $B_2pin_2$ (0.6 equiv), hexanes, 80 °C, 18 h<br>8 Examples, 16-82% yield<br>R = TMS, halogen, OMe,<br>- Substitution in meta position<br>- If a *o*-TMS is present, substitution occurs in para position | 230 |
| 18 | 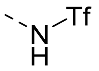 | Acylation | 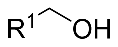 | 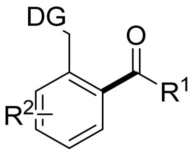 | Substrate (0.3 mmol), Alcohol (1.8 mmol), $Pd(OAc)_2$ (10 mol%), TBHP (1.2 mmol), AcOH (50 mol%), MeCN (1 mL), 120 °C, 40 h<br>18 Examples, 30-84% yield<br>$R^1$ = Aryl, Alkyl<br>$R^2$ = OMe, Halogen<br>- The alcohol reaction partner is oxidized by TBHP to the corresponding acyl radical | 303 |
| 19 | | Acylation | 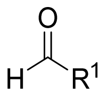 | 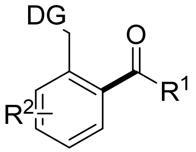 | Substrate (0.3 mmol), aldehyde (0.9 mmol), $Pd(OAc)_2$ (5 mol%), TBHP (3 equiv), AcOH (50 mol%), MeCN/DMF (1/1, 0.6 mL), 100 °C, 20 h<br>24 Examples, 13-75% yield<br>$R^1$ = Aryl, Alkyl<br>$R^2$ = OMe, Me, COOMe, Halogen | 304 |

| | | | | | | |
|---|---|---|---|---|---|---|
| 20 | | Alkenylation/ Cyclization | 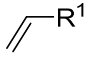 | 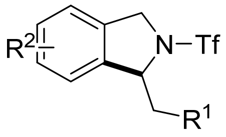 | *N*-Benzyltriflamide (0.3 mmol), Alkene (0.45 mmol), $[RhCp^*Cl_2]_2$ (2.5 mol%), $Cu(OAc)_2 \cdot H_2O$ (200 mol%), DMF-AcOH (3/1, 1 mL), 110 °C, 24 h, sealed tube<br>25 Examples, 21-93%<br>$R^1$ = COOR, CN, C(O)R, $CONMe_2$<br>$R^2$ = Me, OMe, halogen, COOR | 305 |

## Directing groups containing the carbonyl motif

Carbonyl compounds such as esters, ketones and carboxylic acids are essential building blocks for the synthesis of fine chemicals, pharmaceuticals and natural compounds. Form a synthetic point of view, weakly coordinating DGs such as carbonyl or electron rich functional groups (e.g. ethers, hydroxy) show several benefits due to low toxicity, ability for further transformations as well as that they can serve as traceless DGs (e.g. decarboxylation).[306]

## Aldehydes as directing groups in C-H activation

Even though aldehydes are common functional groups in many organic compounds, they have been used as directing groups in only few examples (e.g. alkenylation,[307, 308] annulation,[309, 310]Table 12). The two main reasons are, the low directing ability (Table 12, Entry 4)[310] of aldehydes and additionally, their relatively high tendency to undergo side reactions.. Besides classical imine- directed C-H functionalization, the *in situ* transformation of the aldehyde to the corresponding hydrazone allows an annulation reaction with alkynes at the *ortho* C-H bond of benzaldehyde derivatives. First, the aldehyde is transformed with $NH_2NHAc$ to the corresponding hydrazone and after a reductive elimination, indenones were isolated in good yields after acidic hydrolysis. Finally, the catalyst is regenerated by oxidation of Ag(I) (Table 12, Entry 3).[309]

A highly regioselective alkenylation of indoles with high excess of $Cu(OAc)_2 \cdot H_2O$(50 mol%) for reactivation of the ruthenium catalyst under mild reaction conditions (open flask) has been shown as an straightforward strategy to synthesize 4-substituted indoles (Table 12, Entry 1).[3] This compound class can serves as building blocks for alkaloids and related heterocyclic compounds.[308]

As mentioned in the case of carboxylate- directed C-H activation, the concept of traceless DGs (e.g. decarboxylation) (Table 14, Entry 16)[311] is of great interest especially for a selective and atom efficient synthesis of fine chemicals or natural compounds. A novel rhodium-catalyzed regioselective C-H activation/cyclization of indolyl aldehydes or ketones with alkynes to the corresponding oxindoles in a cascade fashion was reported (Table 12, Entry 4).[310] During optimization studies, tetrahydrofuran and a catalyst loading of 3.5 mol% resulted in improved yields, but interestingly the choice of the oxidant ($Ag_2CO_3$) and $AgSbF_6$ as an additive are crucial for the reaction. The mechanism involves several steps, first a Rh catalyzed C4-H activation followed by a [4+2] cyclization/aromatization and finally a nucleophilic addition of water leads the final motif (Scheme 10, left).

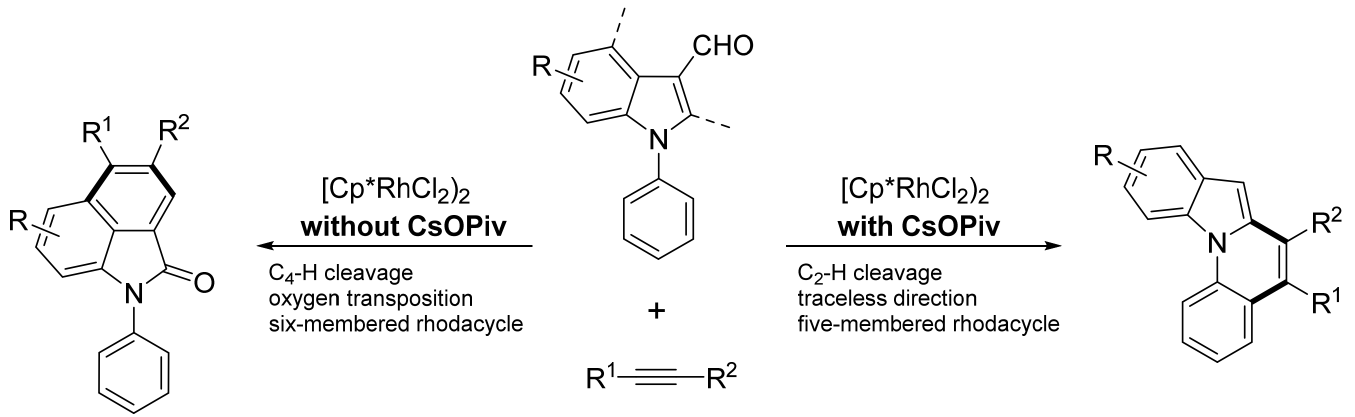


**Scheme 10: Regioselective Rh- catalyzed cyclization depending on the presence of CsOPiv.**

In 2015 the group of You showed the effect of CsOPiv to differentiate between C4-H (Scheme 10, left) and C2-H activation (Scheme 10, right) for similar starting materials. The rhodium catalysed C2-H activation/cyclization requires elevated temperature (140 °C), $Cu(OAc)_2$ as an oxidant in dioxane. This is another example for a traceless aldehyde DG and this procedure leads to bioactive indolo-[1,2-*a*]-quinolone derivatives (Table 12, Entry 5).[312] In the published mechanism, they have stated that coordination of the carbonyl oxygen atom to Rh(III) and pivalate is required for C2−H bond activation to form a five-membered rhodacycle. After the insertion of the alkyne, protonolysis and recyclorhodation to a seven membered rhodacycle was proposed and this intermediate affords after reductive elimination the final product by regeneration of the Rh(III) catalyst.

In 2014, the group of Ackermann reported the first aldehyde directed oxygenation catalysed *via* a ruthenium(II) complex for *ortho*/ *meta* and *para* substituted benzaldehyde derivatives with increased reactivity towards electron rich arenes. The optimized protocol consists of a rate-determining C-H metalation and the hypervalent iodine (III) reagent ($PhI(OTFA)_2$) is required as an oxidant but without further additives.

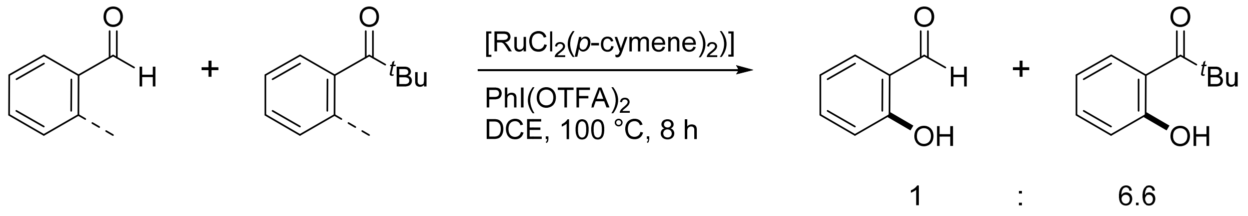


**Scheme 11: Lack of directing power of aldehydes in C-H activation.**

Intermoleculare competition experiments between aldehyde to ketone (1: 6.6) or amide (1:17.5) directed oxygenation (Scheme 11), clearly show the bottleneck of the weakly coordinating aldehydes due to significantly lower directing activity and product formation (Table 12, Entry 6).[313]

**Table 12: Aldehydes as directing groups in C-H activation reactions**

| Entry | Directing group | Type of transformation | Coupling partner | Typical product structure | Comments | Ref |
|---|---|---|---|---|---|---|
| 1 | 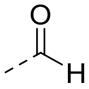 | Alkenylation | 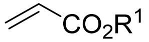 | 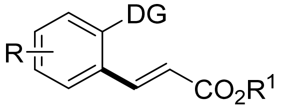 | Substrate (1 mmol), alkene (5- 6 mmol), $[RuCl_2(p\text{-cymene})]_2$ (3 mol%); $AgSbF_6$ (20 mol%); $Cu(OAc)_2{\cdot}H_2O$ (50 mol%), DCE, 100 °C, 16h, under air.<br>17 Examples; Yield: 25- 79%<br>R= OMe, Me, $NMe_2$, Cl<br>$R^1$= Alkyl, OH | 307 |
| 2 | 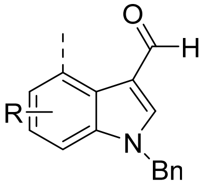 | Alkenylation | 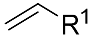 | 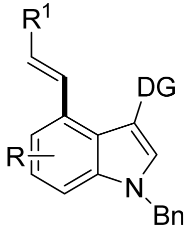 | Substrate (1 equiv.), acrylate (4 equiv.), $[Ru(p\text{-cymene})Cl_2]_2$ (10 mol %), $AbSbF_6$ (20 mol %), $Cu(OAc)_2{\cdot}H_2O$ (1 equiv.), DCE, 120 °C, air.<br>15 Examples; Yield:50- 95%<br>R= Alkyl, alkoxy, halogen<br>$R^1$= COOMe, CHO | 308 |
| 3 | 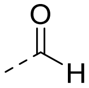 | Annulation | 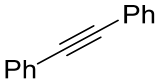 | 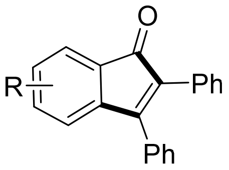 | Substrate (0.2 mmol), alkyne (0.3 mmol), $[Cp^*Rh(MeCN)_3](SbF_6)_2$ (5 mol%), $AgSbF_6$ (20 mol%), $Ag_2CO_3$ (1.0 equiv.), $NH_2NHAc$ (1.1 equiv.), HOAc, 120 °C.<br>12 Examples; Yield: 48-80%<br>R= Alkyl, alkoxy, halogen, $CF_3$ | 309 |
| 4 | 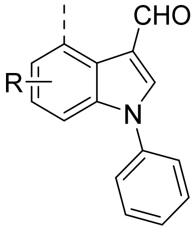 | Annulation,Oxygen transposition | 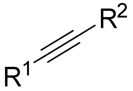 | 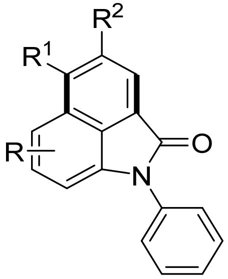 | Substrate (2 mmol), alkyne (0.25 mmol), $[Cp^*RhCl_2]_2$ (3.5 mol%), $AgSbF_6$ (14.0 mol%), $Ag_2CO_3$ (1.2 equiv.), THF, 120 °C, 24h<br>38 Examples; Yield: 30-75%<br>R= Me<br>$R^1$& $R^2$= Substituted aryl (OMe, F, Cl, Br) | 310 |

| | | | | | | |
|---|---|---|---|---|---|---|
| 5 | 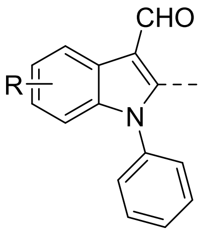 | Cyclization | 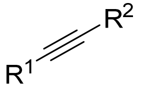 | 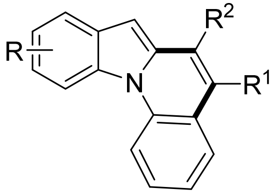 | Substrate (0.375 mmol), alkyne (0.25 mmol), $[Cp^*RhCl_2]_2$ (5 mol%), $Cu(OAc)_2$ (2.1 equiv.), CsOPiv (2.0 equiv.), dioxane, 140 °C, 24h, nitrogen.<br>24 Examples; Yield: 61-95%<br>R= Me, OMe, OBn, COOMe, CN<br>$R^1$& $R^2$= Ph, Me, halogen | 312 |
| 6 | 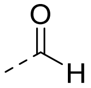 | Hydroxylation | $PhI(OTFA)_2$ | 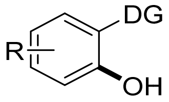 | Substrate (0.5 mmol), $[RuCl_2(p\text{-cymene})_2]$ (2.5 mol%), $PhI(OTFA)_2$ (1.5-2.5 equiv.), DCE, 100 °C, 8h.<br>23 Examples, Yield: 41-72%<br>R= Alkyl, alkyoxy, Ph, halogen | 313 |

**Carboxylic acid- based directing groups**

In contrast to well-studied DG´s such as pyridine, oxazoline, carboxylate-directed C-H activation shows some advantages: cleavage ability, easy to synthesise from other functional groups and availability in organic compounds and a variety of protocols (e.g. alkenylation,[314-316] arylation,[311, 317, 318] hydroxylation[319, 320] etc.) are summarized in the following Table 13.

First published C-H activation on benzoic or naphthoic acids was performed by Miura *et al*. already in 1998 (Table 13, Entry 17).[321] In comparison to the well-studied ruthenium catalysed ketone (Table 15) or carboxylic acid ester (Table 14) directed alkylation reactions, carboxylate directed alkylation reactions (Table 13) promoted by $Pd(OAc)_2$ and $Cu(OAc)_2$ in DMF provided a new access to alkylation reactions. This procedure gave access to phthalides or isocoumarins *via* an *ortho*-vinylation/nucleophilic cyclization or Wacker type oxidative cyclization.

The group of Yu presented several alkenylation reaction (Table 13, Entry 1, 2, 4, 5)[314-316, 322] promoted by $Pd(OAc)_2$ in $^t$Amyl-OH combined with different amino acid ligands. The choice of amino acid ligand effects besides the conversion rate mainly the regioselectivity of the reaction, for example *ortho* or *meta* alkenylation reactions of phenoxyacetic acids (Table 13, Entry 1, 4).[316, 322]

Another major goal for carboxylate-directed C-H functionalisation are one pot processes, containing traceless directing groups that do not require additional steps for DG cleavage. A carboxylate directed *ortho*-alkenylation of benzoic acids with styrene derivatives, catalysed by a rhodium catalyst combined with the cleavage ability of the carboxyl group *via* decarboxylation at 160 °C for 4 h was reported (Table 13 , Entry 6)[323] and for this casethe arrows indicate the former position of the traceless DG. Another example, using a removable carboxylate directing group and a weakly coordinating auxiliaries is the *ortho*- & *meta* selective alkylation of phenol derivatives to synthezise biologically important α-phenoxyacetic acids (Table 13, Entry 4).[316] To control the regioselectivity of this Pd(II) promoted alkylation towards the *meta* position, the carboxylated directing scaffold is exchanged by an CN motif combined with an amino acid ligand system to perform *meta* selective olefination reactions in good yields.

Annulation reaction can be performed *via* Pd, Rh and Ru but nearly all of these procedures requires $CuOAc_2 \cdot H_2O$ as oxidizing agent for cyclization. In general, benzoic acid derivatives serves as substrates, which undergoes annulation reactions with alkenes as well as alkynes. For the second mentioned type of reaction, the regioselectivity of the alkyne insertion is controlled by (Table 13, Entry 14) [324]

Alternatively, lactonization depicts a common cyclization methodology to synthesize different lactones starting for aromatic benzoic acid derivatives. This type of transformation is mainly catalyzed by Pd but also Cu and Rh complexes at high temperatures (80-150 °C) are represented. A novel Pt catalyzed lactonization

procedure in water was developed by the group of Chang (Table 13, Entry 33).[325] This transformation starts with an unusual activation of a $sp^3$ C-H bond and leads to seven and eight membered lactones at 150 °C in moderate to good yields (20-65%).

Also for carboxylic acids as directing group, arylation reactions have been developed using a number of different conditions and aryl sources, mainly aryl iodides and some specific example with $ArBF_3K$, $ArB(OR)_2$ have been used. As can be seen, $Pd(OAc)_2$ is the catalyst precursor of choice and typically no additional ligand was added. Typically high temperatures are required (100-130 °C) and only two examples reported good results already at 80 °C (Table 13, Entry 19 & 20). [326, 327] In contrast to all given examples, the group of Su showed an arylation of benzoic acid with substituted aryl iodides at low temperature. Especially the solvent choice HFIP is responsible to decrease the reaction temperature to 30°C (Table 13, Entry 21).[328] Dastbaravardeh *et al*. presented a very modular carboxylate directed C-H functionalization of mandelic acid and α-phenylglycine mediated by $Pd(OAc)_2$ for arylation, acetoxylation, iodination and olefination reactions (Table 13, Entry 20).[327]The modularity towards the different transformations under identical conditions (AgOAc, KOAc, HFIP, 80 °C) only by adapting the coupling agent gave the desired products in good yields within 24h.A direct *ortho* arylation of benzoic acids as well as a α-arylation of aryl acetic acid derivatives promoted by $Pd(OAc)_2$ are examples for under-represented aryl chlorides or bromides as coupling partner for carboxalyted directed C-H functionalizations (Table 13, Entry 17 & 23).[318, 329]

The group of Yu published a highly selective mono-carboxylation of benzoic acid and phenylic acetic acid derivatives and under optimized conditions. The optimized protocol was also applied to vinylic C-H bond (Table 13, Entry 24).[330] A broad range of phthalic acids were synthesized *via* the generation of six-membered palladacycles[330-332]and the addition of inorganic cations (NaOAc) are crucial for stoichiometric carboxylation with 1 atm CO. Furthermore, the reactivation of Pd(0) to Pd(II) was limited to $Ag_2CO_3$ other oxidants like $Ag_2O$ or $Cu(OAc)_2$ gave less then 10 % conversion.

Besides a $Cu(AcO)_2$ catalyzed hydroxylation on the remote ring system (Table 13, Entry 25)[319] with limiting substrate consumption in the presence of oxygen, a highly selective Pd-catalyzed *ortho* oxygenations of potassium benzoates at 1 atm $O_2$ or air were published in 2009 (Table 13, Entry 26).[320]Based on labelling studies with $O^{18}$ or $H_2{}^{18}O$ the direct $Pd(OAc)_2$ mediated oxygenation was confirmed and the desire target molecules were obtained in good yields using the uncommon solvent (DMA) at 115°C. In contrast to electron rich arenes (yields up to 82 %), electron-withdrawing substituents gave decreased overall yields of around 50%.

**Table 13: Carboxylic acid- based directing groups**

| Entry | Directing group | Type of transformation | Coupling partner | Typical product structure | Comments | Ref |
|---|---|---|---|---|---|---|
| 1 | 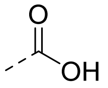 | Alkenylation | 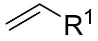 | 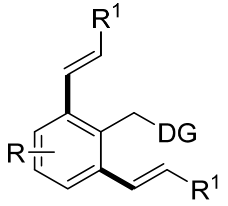 | Substrate (1. equiv.), acrylate (2 equiv.), $Pd(OAc)_2$ (5 mol%), Ac-Val-OH (10 mol%), $KHCO_3$ (2 equiv.), $^{t}$Amyl-OH, 90 °C, 1 atm $O_2$, 6h.<br>24 Examples; Yield: 35-91%<br>R= Me, OMe, halogen, $CF_3$<br>$R^1$= COOR ($^{t}$Bu, Et, Bn) | 322 |
| 2 | 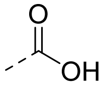 | Alkenylation | 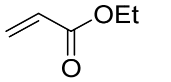 | 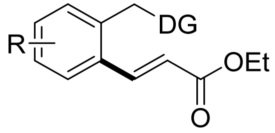 | Substrate (1 equiv.), acrylate (2 equiv.), $Pd(OAc)_2$ (5 mol%), Ac-Ile-OH (10 mol%), $KHCO_3$ (2.0 equiv.), $^{t}$Amyl-OH, 90 °C, 1 atm $O_2$, 48 h.<br>11 Examples; Yield: 72-99%<br>R= Me, OMe, halogen, $NO_2$, $CF_3$ | 315 |
| 3 | 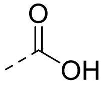 | Alkenylation | 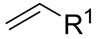 | 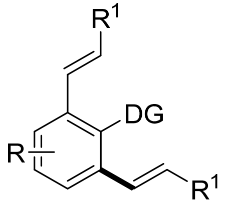 | Substrate (0.5 mmol), alkene (1.5 mmol), $[Cp^*RhCl_2]_2$ (0.005 mmol), Ag(OAc) (2 mmol), DMF, 120 °C, 8-10 h, $N_2$ atm.<br>14 Examples; Yield: 60-84 % | 333 |

| | | | | | | |
|---|---|---|---|---|---|---|
| | | | | | R= Me, OMe, Ph, halogen, $CF_3$<br>$R^1$= Aryl | |
| 4 | 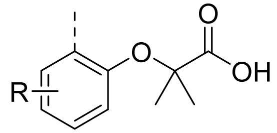 | Alkenylation | 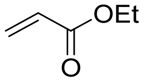 | 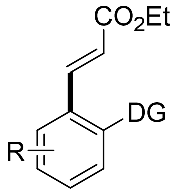 | Substrate (0.1 mmol), acrylate (0.2 mmol), $Pd(OAc)_2$ (5 mol%), Boc-Val-OH (10 mol%), $KHCO_3$ (0.2 mmol), $^t$Amyl-OH, $O_2$, 90 °C, 24 h.<br>15 Examples; Yield: 58-88%<br>R= Me, OMe, halogen, $CF_3$ | 316 |
| 5 | 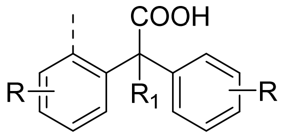 | Alkenylation | 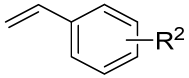 | 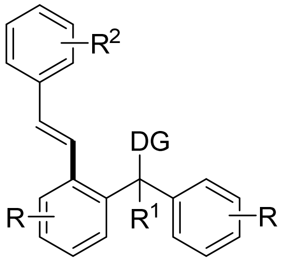 | Substrate (0.5 mmol), $Pd(OAc)_2$ (5 mol%), Boc-Ile-OH·0.5 $H_2O$(10 mol%), BQ (5 mol%), base (e.g. $KHCO_3$) (0.5 equiv.), 1 atm $O_2$, $^t$Amyl-OH, 90 °C, 48 h.<br>19 Examples; Yield: 39-73%<br>R= Alkyl, OMe<br>$R^1$= Alkyl<br>$R^2$= Alkyl and halogen | 314 |

| | | | | | | |
|---|---|---|---|---|---|---|
| 6 | 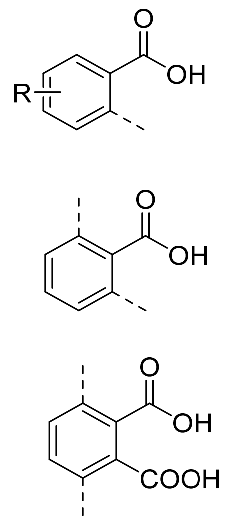 | Alkenylation and Decarboxylation | 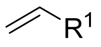 | 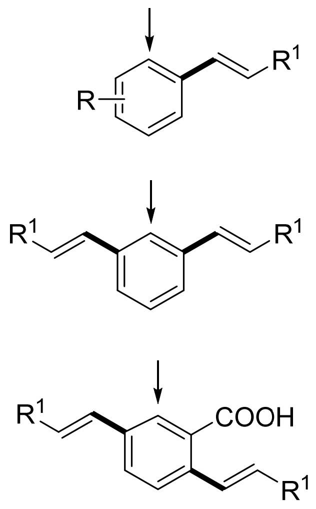 | Substrate (0.5 mmol), alkene (1 mmol), $[Cp^{*}RhCl_2]_2$ (0.005 mmol), AgOAc or $Cu(OAc)_2$ (1-1.5 mmol), DMF or DMAc, 100-120 °C, 10 h.<br>9 Examples; Yield: 55-85%<br>R= OMe, Ph, halogen<br>$R^1$= substituted Ph | 323 |
| 7 | 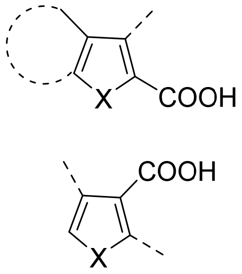 | Alkenylation | 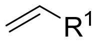 | 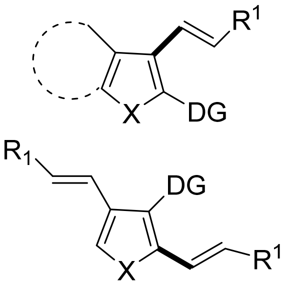 | Substrate (0.25 mmol), acrylate (1.0 mmol), $[Ru(p\text{-cymene-}Cl_2]_2$ (0.005 mmol), $Cu(OAc)_2{\cdot}H_2O$ (0.5 mmol), LiOAc (0.75 mmol), DMF.<br>13 Examples; Yield: 48-94%<br>$R^1$= COOR (alkyl), CONH($^t$Bu), CN<br>X= S, O, NMe | 334 |
| 8 | 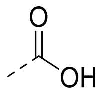 | Alkylation/ Cyclization | 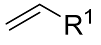 | 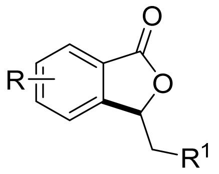 | Substrate (1 mmol), alkene (2.0 mmol), $[RuCl_2(p\text{-cymene})]_2$ (2 mol%), $Cu(OAc)_2{\cdot}H_2O$ (2 mmol), $H_2O$; 80 °C.<br>12 Examples; Yield: 51-95% | 335 |

| | | | | | | |
|---|---|---|---|---|---|---|
| | | | | | R= Me, OMe, halogen<br>$R^1$= COOR (R= alkyl), CN | |
| 9 | 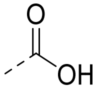 | Annulation | 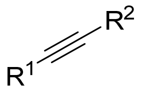 | 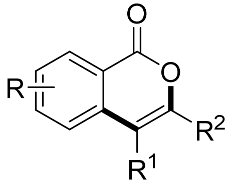 | Substrate (0.5 mmol), alkyne (0.6 mmol), $[Cp^*RhCl_2]_2$ (0.005 mmol), $Cu(OAc)_2·H_2O$ (0.025 mmol), DMF, 120 °C, 2-10 h.<br>22 Examples; Yield: 42-99%<br>R=Me, OMe, OH, $CF_3$<br>$R^1$& $R^2$= Alkyl, Ph | 336 |
| 10 | 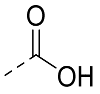 | Annulation | 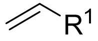 | 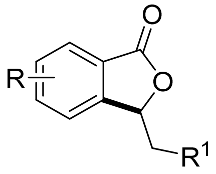 | Substrate (1 mmol), acrylate (3 mmol), $Pd(OAc)_2$ (0.1 mmol), $Cu(OAc)_2·H_2O$ (0.1 mmol), molecular sieve 4A (400 mg), DMF, 6-18 h.<br>5 Examples ; Yield: 34-59 %<br>R= Me, OMe<br>$R^1$= COOR ($^n$Bu, Ph) | 321 |
| 11 | 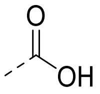 | Annulation | Alkyl halide | 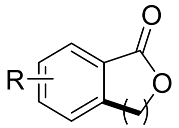 | Substrate (0.5 mmol) $Pd(OAc)_2$ (5 or 10 mol%), base (e.g. $K_2HPO_4$ or $Na_2CO_3$ (3.0 equiv.)), 115-140 °C, 36 h. | 337 |

| | | | | | | |
|---|---|---|---|---|---|---|
| | | | | | 9 Examples; Yield: 26-81 %<br>R= Me, OMe, $CF_3$, COPh<br>Alkyl halide: $ClCH_2CH_2Cl$, $CH_2Br_2$, $C_5H_{11}Cl$ | |
| 12 | 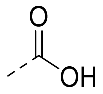 | Annulation | 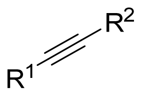 | 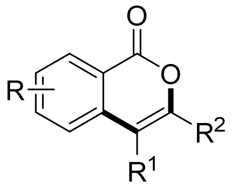 | Substrate (0.5 mmol), alkyne (0.6 mmol), $[Cp^*RhCl_2]_2$ (0.005 mmol), $Cu(OAc)_2\cdot H_2O$ (0.025 mmol), DMF, 120 °C, 2 h, under air.<br>9 Examples; Yield: 83-97%<br>R= Me, OMe, Cl<br>$R^1$& $R^2$= Alkyl, Ph | 338 |
| 13 | 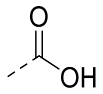 | Annulation | 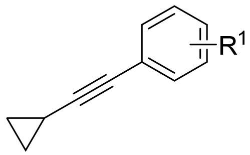 | 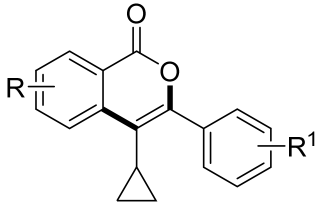 | Substrate (4 equiv.), alkyne (2 equiv.), $[RuCl_2(p\text{-cymene})]_2$ (2.5 mol%), $KPF_6$ (20 mol%), $Cu(OAc)_2\cdot H2O$ , $^t$Amyl-OH, 120 °C.<br>6 Examples; Yield: 21-71%<br>Regioselectivity 7/1<br>R=Me, OMe<br>$R^1$= Me, OMe, COOMe, $CF_3$ | 339 |

| | | | | | | |
|---|---|---|---|---|---|---|
| 14 | 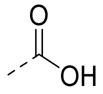 | Annulation | 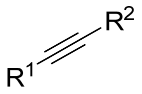 | 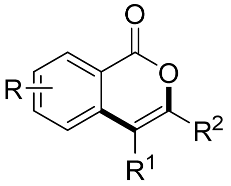 | Substrate (2.0 mmol), alkyne (1 mmol), $[RuCl_2(p\text{-cymene})]_2$ (2.5 mol%), $KPF_6$ (20 mol%), $Cu(OAc)_2 \cdot H2O$, $^{t}$Amyl-OH, 120 °C, 16 h.<br><br>25 Examples; Yield: 60-87%<br><br>$R^1$& $R^2$= Aryl, alkyl | 324 |
| 15 | 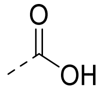 | Arylation | Ar-$BF_3K$ | 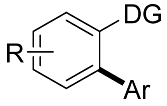 | Substrate (1 eq), aryl trifluoroborate (1.2-1.5 equiv.), $Pd(OAc)_2$ (10 mol%), BQ (0.5 equiv.), $K_2HPO_4$ (1.5 equiv.), 20 atm $O_2$/air, $^{t}$BuOH, 100 °C, 24 h.<br><br>18 Examples; Yield: 41-91%<br><br>R= Me, halogen, $CF_3$, CN, $NMe_2$ | 317 |
| 16 | 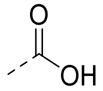 | Arylation | 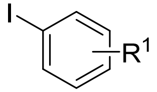 | 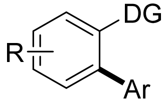 | Substrate (1 equiv.), aryl iodide (3 equiv.), $Pd(OAc)_2$ (2.0 mol%), $Ag_2CO_3$ (1.0 equiv.), AcOH (3.5 equiv.), 130 °C, 16h.<br><br>18 Examples; Yield: 51-83%<br><br>R= Halogen, OMe, $NO_2$, $CF_3$<br><br>$R^1$= Me or halogen (mono or disubstitued) | 311 |

| | | | | | | |
|---|---|---|---|---|---|---|
| 17 | 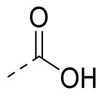 | Arylation | 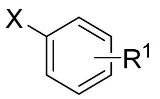 | 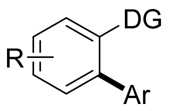 | Substrate (1 mmol), aryl halide (1.5-3 eq), $Pd(OAc)_2$ (5.0 mol%), AgOAc (1.3 equiv.), AcOH (3.5 equiv.), 100-130 °C, 4.5-7 h.<br>20 Examples; Yield: 53-91 %<br>R= Me, OMe, halogen<br>$R^1$= Alkyl, Cl, $CF_3$<br>X= I, Cl | 318 |
| 18 | 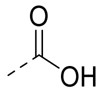 | Arylation | 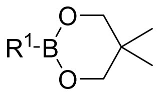 | 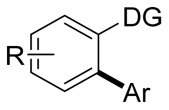 | Substrate (1 equiv.), benzoic acid (1-3 equiv.), $Pd(OAc)_2$ (10 mol%), $Ag_2CO_3$ (1 equiv.), BQ (0.5 equiv.), $^t$BuOH, 100-120 °C, 3 h.<br>6 Examples, Yield: 40-75%<br>R=Me, OMe COOMe<br>$R^1$ = Me, Ph<br>Also for allyl acids | 340 |
| 19 | 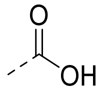 | Arylation | Ar-I | 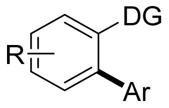 | Substrate (1 equiv.), aryl iodide (2 equiv.), $Pd(OAc)_2$ (5 mol%), AgTFA (1.3 equiv.), Tween 20/$H_2O$ (2 % w/w), 80 °C. | 326 |

| | | | | | | |
|---|---|---|---|---|---|---|
| | | | | | 15 Examples, Yield: 62-92%<br>R= Me, F, $CF_3$, RCO | |
| 20 | 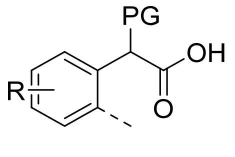 | Arylation | 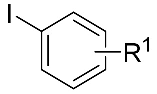 | 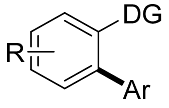 | Substrate (0.1 mmol), aryl iodide (0.002 mmol), $Pd(OAc)_2$ (0.01 mmol), AgOAc (0.2 mmol), KOAc (0.3 mmol), HFIP, 80 °C, 24 h, air.<br>15 Examples, Yield: 38-89%<br>R=Cl, $CF_3$<br>$R^1$= Me, OMe, COOMe, $NO_2$, $CF_3$ | 327 |
| 21 | 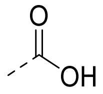 | Arylation | 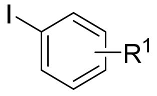 | 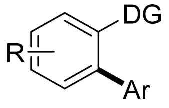 | Substrate (0.2 mmol), aryl iodide (0.4 mmol), $Pd(OAc)_2$ (8 mol%), $Ag_2CO_3$ (0.5 equiv.), $Cs_2CO_3$ (0.5 equiv.), HFIP, 30 °C.<br>35 Examples, Yield: 37-93%<br>R= Me, halogen, $CF_3$, COOR, alkoxy<br>$R^1$=Me, OMe, COOMe, Cl | 328 |
| 22 | 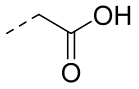 | Arylation | 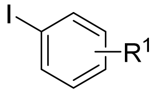 | 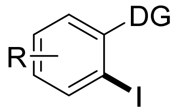 | Substrate (1 equiv.), aryl halide (3 equiv.), $Pd(OAc)_2$ (2 mol%), $Ag_2CO_3$ (0.55 equiv.), $K_2CO_3$ (0.5 equiv.), AcOH (4.5 equiv.), 120 °C, 24 h. | 341 |

| | | | | | | |
|---|---|---|---|---|---|---|
| | | | | | 23<br>18 Examples, Yield: 60-83 %<br>R= Me, OMe, halogen<br>$R^1$= Me, F, $NO_2$, $CF_3$ | |
| 23 | 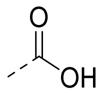 | Arylation | 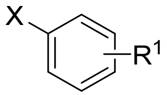 | 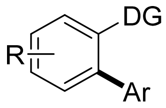 | Substrate (0.1 mmol), aryl halide (1 equiv.), $Pd(OAc)_2$ (5 mol%), NiXantphons (7.5 mol%), $(KNSiMe_3)_2$ (3 equiv.), toluene, 110 °C, 12 h.<br>19 Examples, Yield: 49-82%<br>R= Me, OMe, halogen<br>$R^1$= Me, halogen<br>X= Br, Cl | [329] |
| 24 | 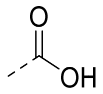 | Carboxylation | CO | 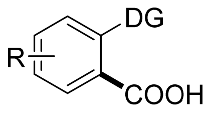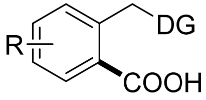 | Substrate (1 equiv.), $Pd(OAc)_2$ (10 mol%), $Ag_2CO_3$ (2.0 equiv.), NaOAc (2.0 equiv.), 130 °C, 18 h, 1 atm CO, 1,4 dioxane.<br>26 Examples; Yield: 45-90%<br>R= Me, Bn, OMe, halogen | [330] |

| | | | | | | |
|---|---|---|---|---|---|---|
| 25 | 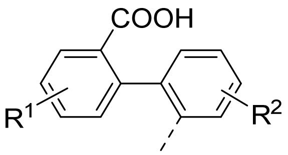 | Hydroxylation | LiOH | 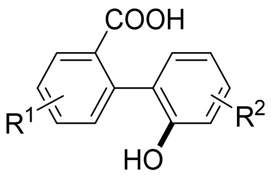 | Substrate (0.5 mmol), $Cu(OAc)_2$ (5.0 mol%), $[PhCO_2]_2$ (1.25 equiv.), HFIP (8 mL/ mmol), 75 °C, 12 h.<br>Hydrolysis: LiOH, MeOH, r.t.<br>25 Examples; Yield: 22-95 %<br>$R^1$& $R^2$= Me, alkoxy | 319 |
| 26 | 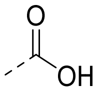 | Hydroxylation | $O_2$ | 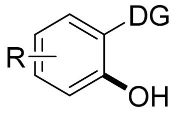 | Substrate (1 equiv.), $Pd(OAc)_2$ (10 mol%), KOAc (2.0 equiv.), BQ (1.0 equiv.), 1 atm $O_2$, DMA, 115 °C, 15 h.<br>20 Examples; Yield: 35-82 %<br>R= Me, OMe, halogen, $CF_3$, $NO_2$, CN, COMe | 320 |
| 27 | 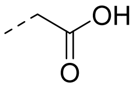 | Iodination | $I_2$ | 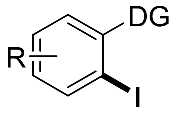 | Substrate (1 mmol), $Pd(OAc)_2$ (2 mol%), $PhI(OAc)_2$ (0.75 equiv.), $I_2$ (0.75 equiv.), DMF, 60 °C, 12h, no light.<br>23 Examples; Yield: 62-82%<br>R= Alkyl, aryl, OPh, halogen, acetyl, $CF_3$ | 342 |

| | | | | | | |
|---|---|---|---|---|---|---|
| 28 | 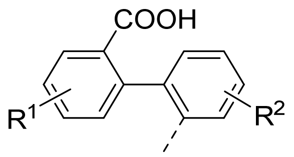 | Lactonization | | 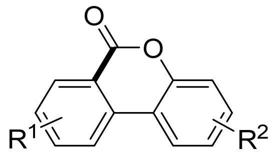 | Substrate (1 equiv.), $Cu(OAc)_2 \cdot H_2O$ (5 mol%), $PhCO_2OtBu$ (3 equiv.), DCE (0.1 M), 85 °C.<br>30 Examples; Yield: 45-97%<br>$R^1$& $R^2$= Alkyl, alkoxy<br>substitution only on one aromatic ring, no example with $R^1$ and $R^2$ together | 343 |
| 29 | 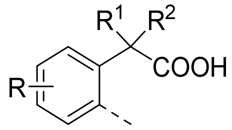 | Lactonization | - | 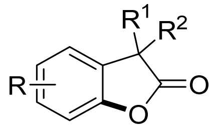 | Substrate (0.2 mmol), $Pd(OAc)_2$ (10 mol%), $PhI(OAc)_2$ (2.0 equiv.), $Ag_2OAc$ (0.5 equiv.), CsOAc/NaOAc (0.5/0.5 equiv.), PhCl/$^t$BuOH (1:1), 100 °C, 12 h.<br>25 Examples; Yield: 35-89%<br>R= Alkyl, alkoxy, aryl, halogen<br>$R^1$& $R^2$=Me, cyclopropyl, cyclohexyl, Bn | 344 |
| 30 | 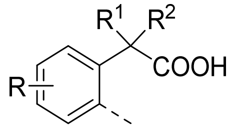 | Lactonization | - | 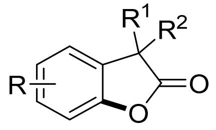 | Substrate (1 equiv.), $Pd(OAc)_2$ (5 mol%), Ac-Gly-OH (30 mol%), $PhI(OAc)_2$ (1.5 equiv.), KOAc (2.0 equiv.), $^t$BuOH, 100 °C, 12 h.<br>22 Examples; Yield: 50-90% | 345 |

| | | | | | | |
|---|---|---|---|---|---|---|
| | | | | | R= Alkyl, alkoxy, aryl, halogen<br>$R^1$& $R^2$= Alkyl, cyclopropyl, cyclobutyl, cyclohexyl | |
| 31 | 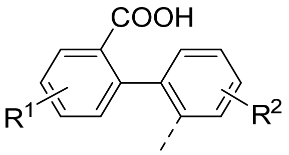 | Lactonization | | 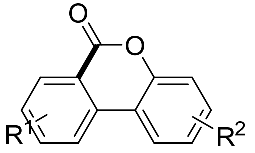 | Substrate (0.2 mmol), $Pd(OAc)_2$ (5.0 mol%), Ac-Gly-OH (15 mol%), $PhI(OAc)_2$ (2.0 equiv.), KOAc (2.0 equiv.), $^tBuOH$, 80 °C, 12 h.<br>28 Examples; Yield: 19-94%<br>$R^1$ & $R^2$= Alkyl, alkoxy, halogen, COOEt, $CF_3$ | 346 |
| 32 | 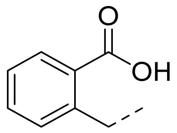 | Lactonization | $R^1CHO$ | 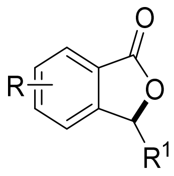 | Substrate (0.1 mmol), aldehyde (0.2 mmol), $[Cp^*RhCl_2]_2$ (8 mol%), AgOTf (40 mol%), $Ag_2CO_3$ (0.4 mmol), dioxane, 150 °C, 48h, argon.<br>19 Examples; Yield: 8-81%<br>R= Me, OMe<br>$R^1$= 3-$NO_2C_6H_4$, 4-& 2-$CF_3C_6H_4$, 3,5-$(CF_3)_2C_6H_4$ | 347 |

| | | | | | | |
|---|---|---|---|---|---|---|
| 33 | 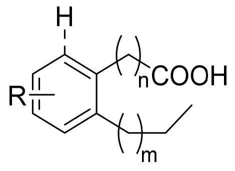 | Lactonization | - | 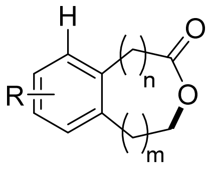 | Substrate (1 equiv.), $K_2PtCl_4$ (10 mol%), $CuCl_2$ (3.0 equiv.), $H_2O$ (0.01 M), 150 °C, 24 h.<br>n= 0, 1<br>m= 0, 1<br>9 Examples; Yield: 20-65%<br>R= Alkyl | 325 |
| 34 | 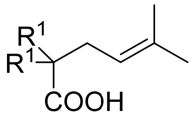 | Intramoleculare lactonization | | 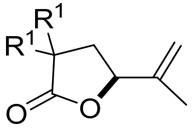 | Substrate (1 equiv.), $Pd(OAc)_2$ (10 mol%), (M,S,S)-*i*-Pr-SPRIX (1 mol%), *p*-benzoquinone (2.0 equiv.), $CH_2Cl_2$ , 25 °C, 60 h.<br>12 Examples; Yield: 56-98 %<br>$R^1$= Ph, Bn, alkyl<br>Some specific example with a different substituted double (e.g. Ph) | 348 |

## Carboxylic Esters

In 1995, first alkylation reactions on aromatic and hetero-aromatic esters were reported by Trost (Table 14, Entry 1)[349] and Kakiuchi (Table 14, Entry 2)[350]*et al.*, using a $RuH_2(CO)(PPH_3)_3$ complex in toluene at reflux conditions for 24. Kakiuchi's procedure was limited to fluoro and tri-fluoro substituted aromatic systems, with exception of two additional presented transformations, using a thiophene carboxylic ester or a lactone. The alkylation established byTrost was demonstrated on different cyclic alkenes containing an ester or ketone directing group combined with alkoxy or alkylsilanes under well established conditions (e.g Murai type reaction) (Table 15, Entry 7).[351] This method was also extended to the addition of styrene (Scheme 12, left) as well as for a regioselective alkenylation with 2 equiv of the depicted silylalkyne (Scheme 12, right) in excellent yields of 82%.

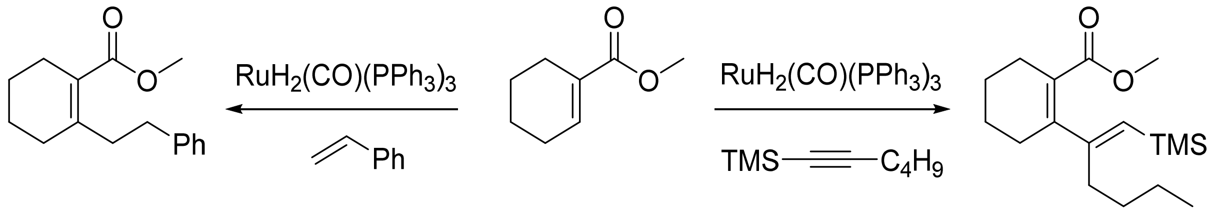


**Scheme 12: Early developments in ester- directed alkylation and alkenylation reactions.**

The weakly coordinating ester group was used by Padala *et al*., for highly chemo and diastereoselective ruthenium catalysed alkenylation. During optimization studies, the effects of additives described and most promising results were obtained with $Cu(OAc)_2$ 3 $H_2O$as oxidant, $AgSbF_6$ under ambient air.[352]

An irdium catalysed amidation under very mild conditions (50 °C) with different sulfonyl azides and a broad substrate scope and good functional group tolerance was demonstrated by Kim et al. This methodology was also adapted for ketone directed amidation reactions and requires besides a Ir(III) precatalyst also $AgNTf_2$, HOAc, $Li_2CO_3$ for generation of the active, positively charged catalyst to promote the regioselective *ortho* amidation (Table 14, Entry 6).[353]

Besides this amidation reaction only a few examples for carbon-heteroatom bond formation reactions (e.g. halogenation[354] or hydroxylation[355]) are published. A highly efficient *ortho* hydroxylation using a mixture of trifluoro-acetic acid and trifluoro-acetic anhydride (TFA/TFAA) and palladium(II) was described for a broad range of starting materials such as aryl ketones, benzoates, benzamides, acetanilides and sulfamides. During optimization studies, the effect of the ratio between TFAA and TFA was evaluated due to reaction speed as well as their role in the catalytic cycle. Most suitable ratio TFA/ TFAA (9:1) showed fast consumption of the starting material and serves also as the required oxygen source (Table 15, Entry 9).[355] Furthermore, the group of Rao demonstrate a hydroxylation procedure for a broad range of easily accessible ethyl benzoates (32 examples) using TFA/ TFAA with similar conditions except the catalyst, in this case $[RuCl_2(p\text{-cymene})]_2$ promotes the oxygenation reaction (Table 14, Entry 10).[356]

In 2012, a novel carboxylic ester directed β-arylation was published by the group of Boudoin. Especially the effect of the aryl-bromide structure on the β/α selectivity for the arylation of tert-butyl isobutyrate should be underlined. Using *ortho* fluoro-aryl bromid, perfect β/α selectivity of 98/2 was obtained (Scheme 13).

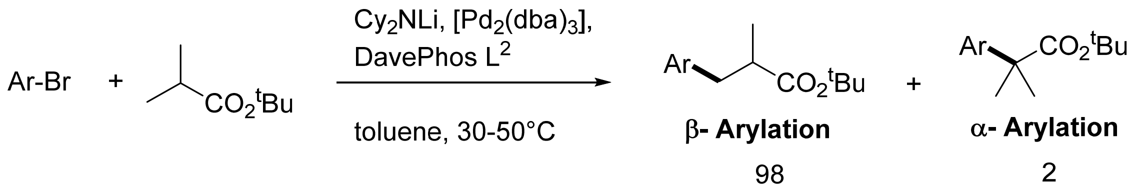


**Scheme 13: Ligand- controlled regioselective aryltion of C(sp$^3$) centers.**

To control the selectivity due to β/α arylations, several different ligands were tested and depending on the structure, the conversions were improved as well as the selectivity. In contrast, *meta*-fluoro or *para*-fluoro substituted aryl bromides showed mixture of both possible products (Table 14, Entry 7).[357, 358]

**Table 14: Ester- directing groups**

| Entry | Directing group | Type of transformation | Coupling partner | Typical product structure | Comments | Ref |
|---|---|---|---|---|---|---|
| 1 | 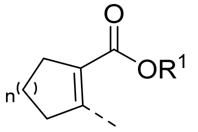  | Alkylation | 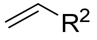  | 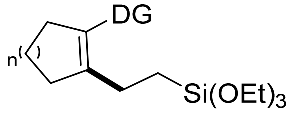  | Substrate (1 equiv.), alkene (1.2-4 equiv.), $RuH_2(CO)(PPh_3)_3$ (5 mol%), toluene, reflux.<br>9 Examples; Yield: 19-92 %<br>$R^1$= Me<br>$R^2$= Ph, $SiR_3$ | 349 |
| 2 | 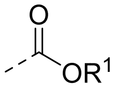  | Alkylation | 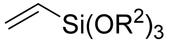  | 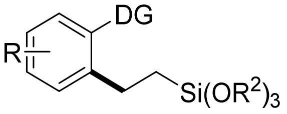  | Substrate (2 mmol), vinylsilane (10 mmol), $RuH_2(CO)(PPH_3)_3$ (0.12 mmol), toluene, 135 °C, 249 Examples, Yield: 42-97%<br>R= F or $CF_3$<br>$R^1$= Me, Et<br>$R^2$= Me, Et | 350 |
| 3 | 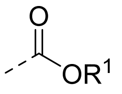  | Alkenylation | 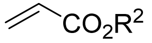  | 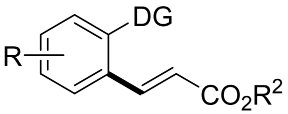  | Substrate (0.2 mmol), acrylate (0.4 mmol), $[RhCp^*Cl_2]_2$ (2.5 mol%), $AgSbF_6$ (10 mol%), Cu(OAc)2*(20 mol%), DCE, 110 °C, 12 h.<br>23 Examples; Yield: 3- 72%<br>R= Me, OMe, OH, halogen<br>$R^1$= Alkyl, Bn<br>$R^2$= Alkyl | 359 |

| | | | | | | |
|---|---|---|---|---|---|---|
| 4 | 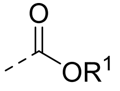 | Alkenylation | 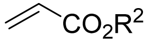 | 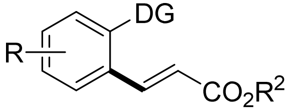 | Substrate (1 equiv.), acrylate (1 equiv.), $[RuCl_2(p\text{-xymene})]_2$ (3 mol%), $AgSbF_6$ (20 mol%), $Cu(OAc)_2$ (30 mol%), DCE, 100 °C, 12 h.<br>15 Examples; Yield: 41-89 %<br>R= OMe, OH, halogen<br>$R^1$= Alkyl<br>$R^2$= Alkyl, halogen | 352 |
| 5 | 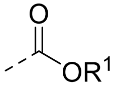 | Alkenylation | 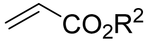 | 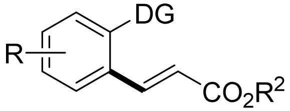 | Substrate (0.5 mmol), acrylate (1.0 mmol), $[RuCl_2(p\text{-cymene})]_2$ (5 mol%), $AgSbF_6$ (40 mol%), $Cu(OAc)_2{\cdot}H_2O$ (1.0 mmol), DCE, 100 °C, air, 16 h.<br>14 Examples; Yield: 48-68 %<br>R= Me, OMe<br>$R^1$= Alkyl<br>$R^2$= Alkyl | 360 |
| 6 | 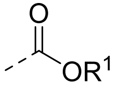 | Amidation | $R_2SO_2N_3$ | 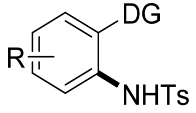 | Substrate (0.1 mmol), azide (1.0 equiv.), $[IrCp^*Cl_2]_2$ (4 mol%), $AgNTf_2$ (16 mol%), HOAc (15 mol%), $Li_2CO_3$ (15 mol%), DCE, 50 °C, 12 h.<br>16 Examples; Yield: 51-99%<br>$R^1$= Alkyl, cyclopropyl, lactones, Bn<br>$R_2SO_2N_3$= NHTs, $NHSO_2Me$, $NHSO_2CH_2C_6H_5$ | 353 |
| 7 | 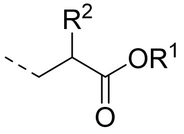 | β Arylation | 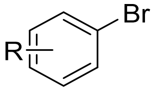 | 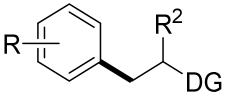 | Substrate (1.6 equiv.), aryl bromide (1 equiv.), $[Pd_2(dba)_3]$ (5 mol%), $Cy_2NLi$ (1.7 equiv.), Davephos (10 mol%) toluene, 110 °C.<br>21 Examples, Yield: 61-81%<br>R= Me, OMe, halogen, $CF_3$<br>$R^1$= Alkyl, Bn<br>$R^2$ = Me, $CF_3$, $NBn_2$ | 357, 358 |

| | | | | | | |
|---|---|---|---|---|---|---|
| 8 | 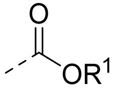 | Halogenation | NCS/ NBS | 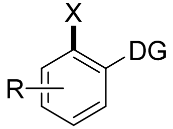 | Substrate (1 equiv.), $Pd(OAc)_2$ (1.1 equiv.), NCS or NBS (1.0-6.0 equiv.), TfOH, DCE, $Na_2S_2O_8$, 60-90 °C.<br>26 Examples; Yield: 36-85%<br>R= Me, halogen, $NO_2$<br>$R^1$= Alkyl | [354] |
| 9 | 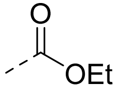 | Hydroxylation | TFA/TFAA | 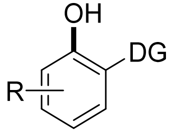 | Substrate (1 equiv.), $Pd(OAc)_2$ (5 mol%), TFA/TFAA (9:1), oxidants (2 equiv.) (e.g. $K_2S_2O_8$), r.t-50 °C.<br>6 Examples; Yield: 36-82 %<br>R= Me, OMe, halogen | [355] |
| 10 | 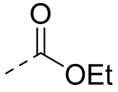 | Hydroxylation | TFA/TFAA | 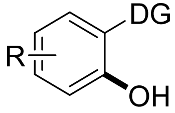 | Substrate (1 equiv.), $[RuCl_2(p\text{-cymene})]_2$ (2.5 mol%), Selectfluor (1.1 equiv.),TFA/TFAA (7:3), 80 °C.<br>32 Examples; Yield: 7-93 %<br>R= Me, OMe, halogen, $CF_3$,COOMe | [356] |

## Ketones

The carbonyl group in ketones was historically amongst the first directing groups to be used in C-H activation chemistry. Over the last years, several ketone directed C-H functionalizations were reported for a broad substrate scope of $sp^2$ C-H bonds[361] except of some specific examples (Table 15, Entry 9).[362, 363] Ruthenium proved to be especially well suited for ketone directed C-H activation reactions. As can be seen in Table 15, most example take advantage of ruthenium catalysts followed by rhodium as the second most frequently applied metal. Palladium[364], has been used successfully only in a handful of examples.

Already in 1993, pioneering work for ketone directed alkylations was published by Murai *et al.* (Table 15, Entry 7)[351] In this landmark contribution, the first highly efficient and selective carbon-hydrogen cleavage with a simultaneous C-C bond formation mediated by a ruthenium complex on different aromatic ketones (e.g. naphtyl, furan, thiophen) with a mono and disubstitued olefines was shown. (Scheme 14).

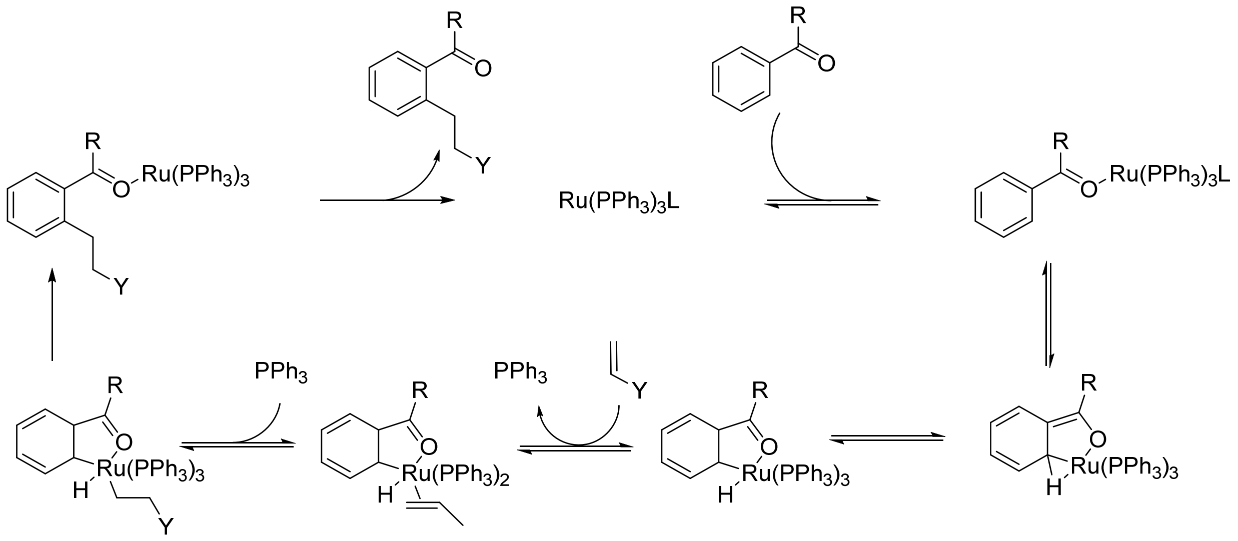


**Scheme 14: Proposed mechansim of carbonyl- directed C-H functionalization.**

The mechanism proposed in this paper served as guideline for many more contributions to come, and can be considered as one of the most important starting points for the field of C-H activation chemistry, as we experience it today.[365, 366] It was proposed, that the carbonyl function first precoordinates the metal catalyst, in this case a ruthenium species, which brings it into a position in close proximity of the a-C-H bond next to the ketone function. This allows C-H insertion of Ru into the C-H bond. Basically, the majority of directed C-H activation reactions rely on this type of strategy. In this early example of Murai, olefin insertion and reductive elimination delivered the α-alkylated ketones, the final products of the reported transformation. The reaction was performed with a$RuH_2(CO)(PPh_3)_3$ pre-catalyst, which was reduced to the active Ru(0) in toluene at 135 °C. Further improvements towards reduced reaction temperature (r.t. to 40 °C) and mechanistic studies were published in 2010.[365]

Independently, the groups of Chaudret (Table 15, Entry 10)[367] and Leitner (Table 15, Entry 9)[363]presented optimized alkylation protocols at room temperature catalysed *via* athermolabile$RuH_2(H_2)(PCy_3)_2$ catalystfor aromatic ketones with ethylene. An Ru(II) promoted *ortho* alkylation with an unusual coupling partner, namely maleimides at high temperature (120 °C) with 4 equiv. of water to generate 3-arylated succinimide derivatives in excellent yields (96%) was published (Table 15, Entry 18).[368]

In 1995, the first catalytic addition of an inactive aromatic C-H bond to a triple bond catalysed by $Ru(H)_2(CO)(PPh_3)_3$ in toluene at 135 °C was reported with moderate to good regioselectivity (E/Z= 5/1- 16/1) (Table 15, Entry 4).[369] Besides different coupling reagents, such as symmetric or asymmetric acetylenes and different vinylsilanes also furan or thiophen was shown as model substrates. An alkenylation protocol to install fluorine scaffolds *via* perfluoro-alkenylation, mediated by 1 mol% $[(RhCp^*Cl_2)_2]$ was presented for cyclic and acyclic aromatic ketones (Table 15, Entry 5).[370]In the proposed mechanism, the final β-hydrogen elimination exclusively gives the E isomer of perfluoroethyl acrylate derivatives. In contrast to the well studied Murai type alkylation, which provides linear products, in 2014 a Ir promoted alkene- hydroarylation to generate disfavored branched compounds was publishied (Table 15, Entry 22).[371] The selectivity towards the C-C bond forming with the internal carbon of styrene is controlled by the ligand (e.g. $d^Fppb^c$) and the styrene loading (coupling reagent) was reduced during optimization studies form 450 mol% to 200 mol% to gave exclusively the branched products.

The effect ofnon-coordinating anions (e.g. $AgSbF_6$, $KPF_6$) for ruthenium catalysed annulation reactions is depicted in Scheme 15 (Table 15, Entry 28-30)**.**[372-374] The addition of $AgSbF_6$ is required, to increase the activity of the rhodium catalyst by removing the chloride ligands $[(RhCp^*Cl_2)_2]$ (Table 15, Entry 28).[68] Furthermore to favour the ring closure reaction, the addition of a $Cu(OAc)_2$ as an oxidant as well as the solvent are crucial, to avoid the well-known alkenylation reaction of aromatic ketones with alkynes.

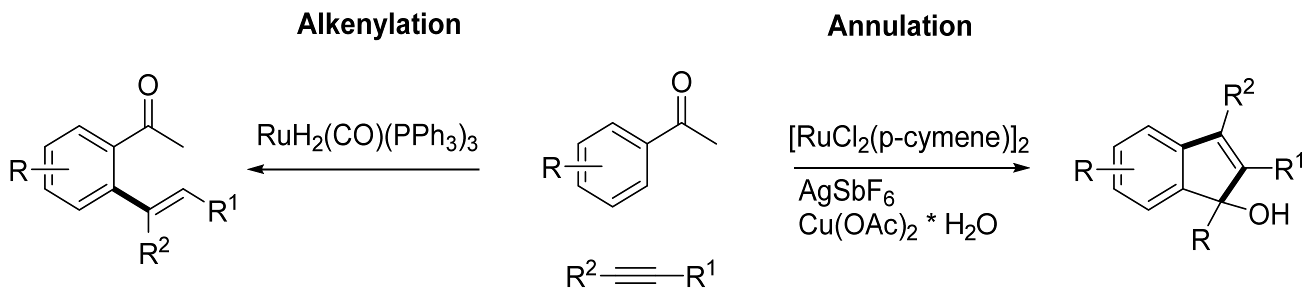


**Scheme 15 Ketone directed alkenylation and annulaion controlled by the catalyst.**

In 2011, Patureau *et al.* presented a novel annulation process to synthesize indenols and fulvenes depending on the substrate structure involved an α, γ dehydration step (loss of a $H_2O$) or non-dehydrative reaction progress (Table 15, Entry 28).**[372]**

In order to avoid several bottlenecks (e.g. regioselectivity) of β-functionalization, a selective Pd- catalyzed arylation using aryl iodides with excellent functional group tolerance at the β-position of cyclic or acyclic ketones was reported (Table 15, Entry 24).[375] The selective arylation in β-position was obtained by a Pd promoted ketone dehydrogenation followed by the formation of an Pd(II)-enlolate, a β-H elimination and finally a reductive elimination for catalyst reactivation are part of the catalytic cycle to end up at the regioselective product molecule. Furthermore, a rhodium catalysed β alkylation of 4-phenyl-3-buten-2-one utilizing diethylamine as an chelation assistant tool to give β, γ unsaturated ketones in 2:1 ratio of E/Z isomers (Table 15, Entry 12).[376] Key step of this amine assisted functionalisation is the formation of an dienamine intermediate by the condensation of α,β- unsaturated ketone and diethylamine. The active rhodium complex is then coordinating and reductive elimination followed by acidic hydrolysis, which yields in the final product.

A ketone directed C-H activation for a enantioselective hydroarylative and hydrovinylative cyclizations were presented by the group Shibata (Table 15, Entry 34).[377] They also stated a possible mechanism for the presented cyclization, which follows a (1) directed C-H activation of an enone, (2) a hydrorhodation of the diyne or enyene and (3) intramolecular carborhodation followed by the generation of the thermodynamically favoured product.

Ketone directing groups have not only been applied in C-C bond forming reactions but also in C-heteroatom bond formations. For example, ruthenium or palladiumcatalysed hydroxylation,[364, 370, 378] amination[379], as well as halogenation.[380]

To install a nitrogen containing functional group on an aromatic system, *ortho* amidation procedures by sulfonyl azides were studied in presence of a $RuCl_2$(*p*-cymene)]$_2$ (Table 15, Entry 19- 21).[381-383] This methodology was adapted to broad substrate scope, do not require external oxidants and only nitrogen is generated as byproduct. Another opportunity for direct C-hetereoatom formation, is described for benzophenones *via* $Pd(OAc)_2$ catalyzed mono- or di-hydroxylation reactions. In 2012, the first ketone directed mono- selective arene oxidation using $PhI(OTFA)_2$ as oxidant in DCE was presented (Table 15, Entry 35).[378] The protocol was extended to substituted benzophenones and the final product is formed after aqueous work up of 2-trifluoro-acetoxylbenzophenone with perfect regioselectivity. Only tolyl-phenylketone was described to generate the dihydroxylated product.

Another direct hydroxylation protocol to synthezise *ortho*- acylphenols by $Pd(TFA)_2$ combined with the oxidant (BTI: bis(trifluoroacetoxy)iodo]benzene) at low temperature was reported by the group of Dong (Table 15, Entry 36).[364] For benzophenone derivatives, both electron neutral and electron rich aromatic systems are di-hydroxylated and for unsymmetrical benzophenones a mono-selectivity trend towards more electron rich aromatic rings is reported.

A ketone or ester directed hydroarlytion catalysed via inexpensive $CoBr_2$ and a bidendate phosphine complex (e.g. dppp or dppe) represents a novel protocol to prepare biologically relevant scaffolds containing an exocyclic double bond. During the catalytic cycle, Co(II) is reduced by Zn dust to Co(I), which promotes the oxidative cyclization of 1,6 enynes. Finally the rate determine step, a reductive elimination gave access to functionalized pyrrolidines and dihydrofurans by an atom efficient synthetic process at 40 °C (Table 15, Entry 33).[384] Additionally the reaction progress is limited to chlorinated solvents (DCM or DCE), only low yields were obtained in dioxane, THF or toluene. Furthermore, $CoI_2$ or$CoCl_2$ combined with different ligands decreasing the activity towards hydroarylatative cyclization.

The groups of Shi and Cheng disclosed independently and simultaneously an identical protocol for the synthesis of fluorenones from benzophenones *via* oxidative dual C-H activation (Table 15, Entry 38 & 39).[385, 386] Both groups also presented an plausible mechanism with the rate determing step, the formation of an six membered palladium complex after double C-H activation. Finally, a reductive elimination leads to the target molecules and the catalyst is recycled with $Ag_2O$.

**Table 15: Ketone directing groups**

| Entry | Directing group | Type of transformation | Coupling partner | Typical product structure | Comments | Ref |
|---|---|---|---|---|---|---|
| 1 | 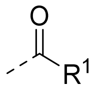 | Alkenylation | 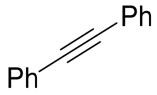 | 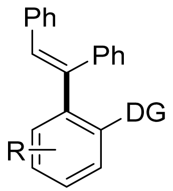 | Substrate (2 equiv.), alkyne (1 equiv.), $[Ir[cod]_2]BF_4$ (5 mol%), rac-BINAP (5 mol%), DCE, reflux, 20 h.<br>8 Examples; Yield: 59-99%<br>R= Me, OMe, $CF_3$<br>$R^1$= Alkyl | 387 |
| 2 | 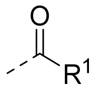 | Alkenylation | 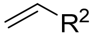 | 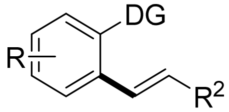 | Substrate (1 mmol), alkene (5-6 mmol), $[RuCl_2(p\text{-cymene})]_2$ (2 mol%), $AgSbF_6$ (10 mol%), $Cu(OAc)_2{\cdot}H2O$ (25 mol%), DCE, 110 °C, 12 h, under air.<br>19 Examples; Yield: 55-89 %<br>R= Me, OMe, halogen, COOMe<br>$R^1$= Alkyl; $R^2$= Me, OEt,alkyl, aryl, COOR (alkyl) | 388 |
| 3 | 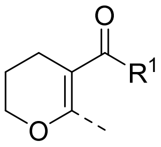 | Alkenylation | 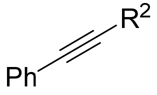 | 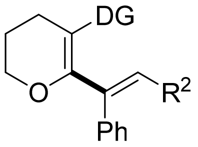 | Substrate (2 mmol), $RuH_2(CO)(PPh_3)_3$ (0.12 mmol), toluene, 135 °C.<br>3 Examples; Yield: 56-96%<br>$R^1$=Alkyl<br>$R^2$= Ph, $SiMe_3$ | 389 |
| 4 | 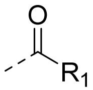 | Alkenylation | 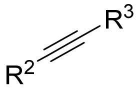 | 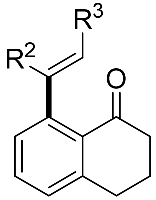 | Substrate (2 mmol), alkyne (4 mmol), $RuH_2(CO)(PPH_3)_3$ (0.12 mmol), toluene, 135 °C.<br>9 Examples; Yield: 20-99%<br>E/Z= 5/1-16/1<br>$R^1$= Alkyl; $R^1$ &$R^2$= Alkyl, $SiMe_3$<br>Heterocycles tolerated | 369 |

| | | | | | | |
|---|---|---|---|---|---|---|
| 5 | 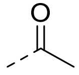 | Alkenylation | 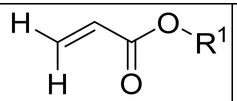 | 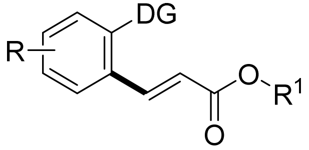 | Substrate (1 equiv.), alkene (1.5 equiv.), $[(RhCp^*Cl_2)_2]$ (1.0 mol%), $AgSbF_6$ (20 mol%), $Cu(OAc)_2$ (1.0 equiv.), $^t$Amyl-OH, 100 °C.<br>16 Examples; Yield: 70-96%<br>R= Alkyl, alkoxy, halogen, di-substituted<br>$R^1$= $CH_2CF_3$, $CH_2CF_2CF_3$ | 370 |
| 6 | 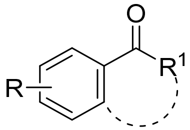 | Alkenylation/ Cyclization | 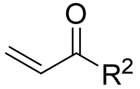 | 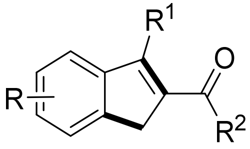 | Substrate (0.2 mmol), acrylate (0.5 mmol), $Cp^*Rh(CH_3CN)_3(SbF_6)_2$ (5 mol%), AgOAc (1.0 equiv.), $H_2O$, DCE, 130 °C, 48 h.<br>20 Examples; Yield: 21-78%<br>R= Me, OMe, halogen; $R^1$= Me; $R^2$= Alkyl | 390 |
| 7 | 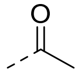 | Alkylation | 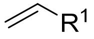 | 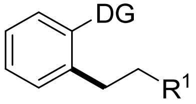 | Substrate (2 mmol), alkene (2-12 mmol), $RuH_2(CO)(PPH_3)_3$ (0.04 mmol), toluene, 125 °C, 0.2-90 h.<br>13 Examples; Yield: 66-99%<br>R= Me<br>$R^1$= $SiMe_3$, $Si(OEt)_3$, aryl, $^t$Bu<br>Heterocycles tolerated | 351 |
| 8 | 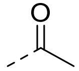 | Alkylation | 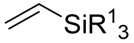 | 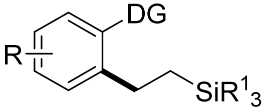 | Substrate (1 mmol), alkene (2 mmol), $RuH_2(CO)(PPH_3)_3$ (2 mol%), toluene, r.t.-40 °C, 48 h.<br>8 Examples; Yield: 74-96%<br>R= Me; $R^1$= Alkyl | 365 |
| 9 | 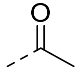 | Alkylation | Ethylene | 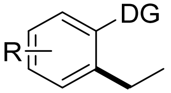 | Substrate (0.4 mmol), ethylene (1 g, 30 bar), $RuH_2(H_2)_2(PCy_3)_2$ (0.04 mmol),toluene, 23 °C.<br>5 Examples; Yield 22-100%<br>R= Me, OMe, Cl, $CF_3$ | 363, 391 |

| | | | | | | |
|---|---|---|---|---|---|---|
| 10 | 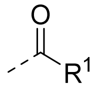 | Alkylation | Ethylene | 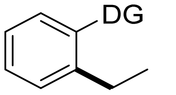 | Substrate (10 equiv.), ethylene (800 equiv.), $RuH_2(H_2)_2(PCy_3)_2$ (1 eq), pentane, 18 °C.<br>7 Examples; Yield: depending on the temperature<br>$R^1$= Me, Ph | [367] |
| 11 | 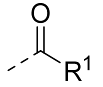 | Alkylation | 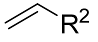 | 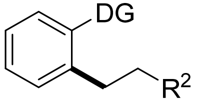 | Substrate (0.324 mmol), alkene (0.972 mmol), $[Rh(PPh_3)_3Cl]$ (5 mol%), $PhCH_2NH_2$ (0.162 mmol), toluene, 150 °C, 6 h.<br>10 Examples; Yield: traces-95%<br>$R^1$ & $R^2$= Alkyl | [392] |
| 12 | 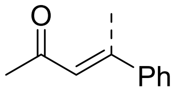 | Alkylation | 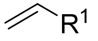 | 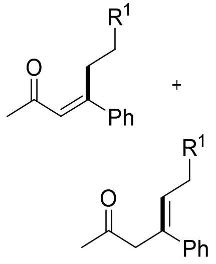 | Substrate (1 mmol), alkene (10 mmol), $RhCl(PPh_3)_3$ (0.05 mol%), $PhCO_2H$ (0.1 mmol), sec-amine (0.5 mmol), toluene, 150 °C.<br>11 Examples; Yield: 6-99%<br>$R^1$= Alkyl, cyclohexyl, Mesilane | [376] |
| 13 | 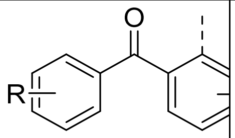 | Alkylation | 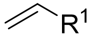 | 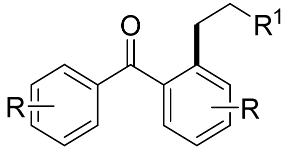 | Substrate (1 equiv.), alkene (1 equiv.), $RuH_2(CO)(PPh_3)_3$ (5 mol%), cyclohexan, 120 °C.<br>10 Examples; Yield: 10-99%<br>5 Examples; Yield: 35-91 % (acetophenone derivatives)<br>R= OMe, $CF_3$; $R^1$= $SiMe_3$, $C_3H_7$, OEt, cyclopentene | [393] |
| 14 | 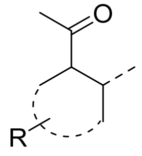 | Alkylation | 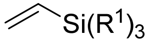 | 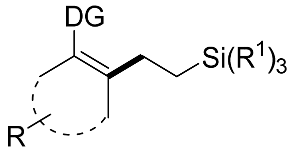 | Substrate (1 mmol), vinyl silane (2 equiv.), $[RuCl_2(p\text{-}cymene)Cl_2]_2$ (2.5 mol%), $NaHCO_2$ (30 mol%), $PPh_3$ (15 mol%), toluene, 140 °C.<br>10 Examples; Yield: 70-100 %<br>R= Me, OMe, halogen; $R^1$= Me, OEt<br>Heterocylces tolerated | [394] |

| | | | | | | |
|---|---|---|---|---|---|---|
| 15 | 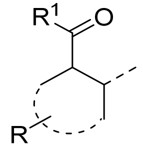 | Alkylation | 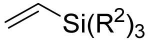 | 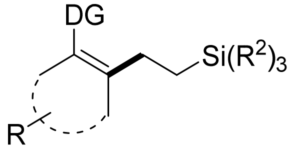 | Substrate (1 mmol), vinyl silane (2 equiv.), $[RuCl_2(p\text{-}cymene)Cl_2]_2$ (2.5 mol%), $NaHCO_2$ (30 mol%), $PPh_3$ (15 mol%), toluene, 140 °C.<br>10 Examples; Yield: 21-100 %<br>R= Me, OMe, halogen; $R^1$= Alkyl, Ph, cyclopropyl<br>$R^2$= Me, OMe, OEt | 395 |
| 16 | 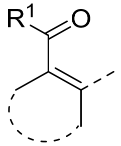 | Alkylation | 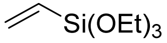 | 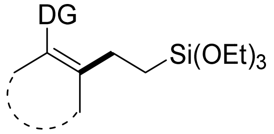 | Substrate (1 mmol), vinyl silane (2 mmol), $[RuCl_2(p\text{-}cymene)]_2$ (5 mol%), $P(Ar)_3$ (10-15 mol%), NaOCOH (30 mol%), $^i$PrOH, co solvent, 80 °C.<br>14 Examples; Yield: 49-91%<br>$R^1$= Cy, Et | 396 |
| 17 | 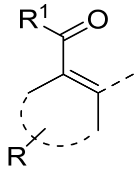 | Alkylation | 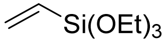 | 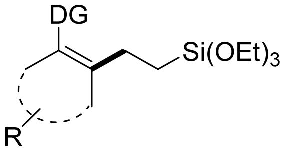 | Substrate (1 mmol), vinyl silane (2 mmol), $RuCl_3{\cdot}H_2O$ (4 mol%), $NaHCO_2$ (30 mol%), $P(4\text{-}CF_3C_6H_4)_3$ (15 mol%), dioxane, 80 °C, 20 h.<br>11 Examples; Yield: 54-92%<br>R= Me, OMe, halogen<br>$R_1$= Me, cyclohexanone, (1-ethoxyethyl)-benzene<br>Heterocycles tolerated | 397 |
| 18 | 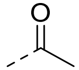 | Alkylation (Maleimides) | 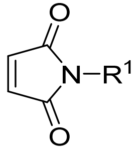 | 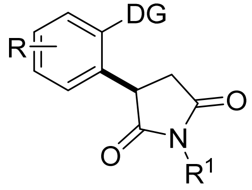 | Substrate (0.3 mmol), maleimide (0.6 mmol), $[Ru(p\text{-}cymene)Cl_2]_2$ (7.5 mol%), $AgSbF_6$ (30 mol%), $Cu(OAc)_2{\cdot}H_2O$ (1.5 equiv.), AcOH (10.0 equiv.), $H_2O$ (5.0 equiv.), DCE, 120 °C, argon.<br>19 Examples; Yield: 54- 90%<br>R= Alkyl, alkoxy, halogen; $R^1$= Bn, Ph, Et | 368 |

| | | | | | | |
|---|---|---|---|---|---|---|
| 19 | 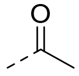 | Amidation | 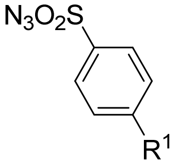 | 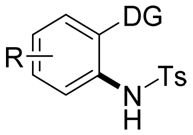 | Substrate (1 mmol), azide (1.5 mmol), $[RuCl_2(p\text{-cymene})]_2$ (5mol%), $AgSbF_6$ (20 mol%), $Cu(OAc)_2{\cdot}H2O$ (50 mol%), DCE, 100 °C.<br>17 Examples; Yield: 8-94%<br>R= Alkyl, alkoxy, halogen<br>$R^1$= Alkyl, $NO_2$, $CF_3$, halogen, Bn | 381 |
| 20 | 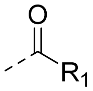 | Amidation | 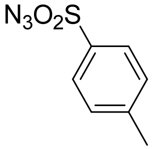 | 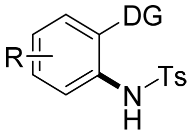 | Substrate (2 equiv.), azide (0.2 mmol), $[RuCl_2(p\text{-cymene})]_2$ (4 mol%), $AgNTf_2$ (16 mol%), NaOAc (20 mol%), DCE, 80 °C, 12 h.<br>20 Examples; Yield: 40-97%:<br>R= Me, OMe, halogen; $R^1$= Alkyl, Ph | 382 |
| 21 | 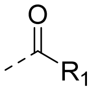 | Amidation | 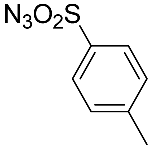 | 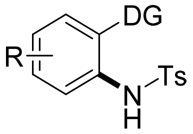 | Substrate (0.3 mmol), azide (0.6 mmol), $[RuCl_2(p\text{-cymene})]_2$ (2.5 mol%), $AgSbF_6$ (10 mol%), $Cu(OAc)_2$ (30 mol%), DCE, 80 °C.<br>20 Examples; Yield: 30-85%<br>R= Me, OMe, halogen<br>$R^1$= Alkyl, cyclopropyl, cyclopentyl, Ph | 383 |
| 22 | 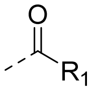 | Arylation | 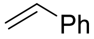 | 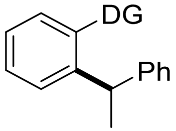 | Substrate (100 mol%), alkene (450 mol%), $[Ir(cod)_2]$BARF (5 mol%), $d^F$ppb (5 mol%), dioxane, 100-120 °C, 24-48 h.<br>5 Examples; Yield: 18-84%<br>$R^1$= Ph, alkyl& amides | 371 |
| 23 | 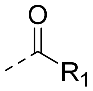 | Arylation | 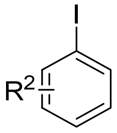 | 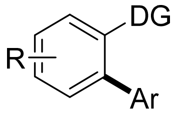 | Substrate (1 mmol), aryl iodide (3 mmol), $Pd(OAc)_2$ (10 mol%), $Ag_2O$ (1.0 equiv.), TFA, 120 °C, 20 h.<br>11 Examples; Yield: 23-92%<br>R= Alkyl, halogen<br>$R^1$= Alkyl, Ph<br>$R^2$= OMe, COOEt, $NO_2$ | 398 |

| | | | | | | |
|---|---|---|---|---|---|---|
| 24 | 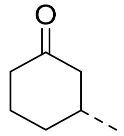 | Arylation | 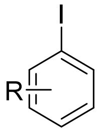 | 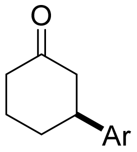 | Substrate (1 mmol), aryl iodide (0.4 mmol), $Pd(TFA)_2$ (0.04 mol%), $P(i\text{-}Pr)_3$ (0.08 mol%), AgTFA (0.8 mmol), HFIP/ dioxane 1:1, 80 °C, 12 h.<br>25 Examples; Yield: 32-91%<br>R= Me, OMe, RCOR, RCOH, halogen | 375 |
| 25 | 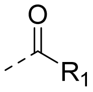 | Arylation | 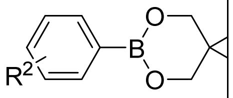 | 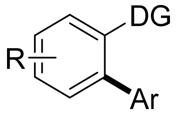 | Substrate (2 mmol), phenylboronate (1 mmol), $RuH_2(CO)(PPh_3)_3$ (0.02 mol%), toluene, reflux.<br>15 Examples; Yield: 56-92%<br>R= OMe, F, $CF_3$; $R^1$= Alkyl; $R^2$= Me, OMe, $NMe_2$ F, $CF_3$ | 399 |
| 26 | 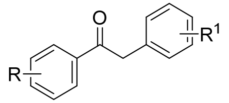 | Arylation | 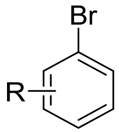 | 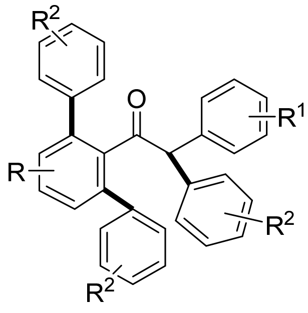 | Substrate (1 mmol), aryl bromide (1-7.5 mmol), $Pd(PPH_3)_3$ (0.01-0.005 mmol), $Cs_2CO_3$ (3-5 mmol), *o*-xylene, $N_2$, 160 °C.<br>25 Examples; Yield: 18-68%<br>R= OMe, Cl<br>$R^1$ & $R^2$= OMe, Cl | 400 |
| 27 | 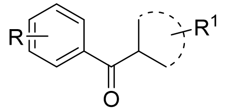 | Arylation and Cyclization | 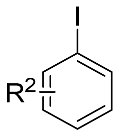 | 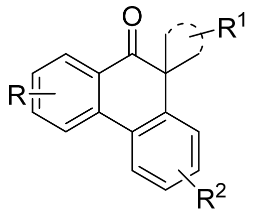 | Substrate (1 mmol), aryl iodide (3 mmol), $Pd(OAc)_2$ (10 mol%), $Ag_2O$ (1.0 equiv.), TFA, 120 °C, 20 h.<br>9 Examples; Yield: 60-78%<br>R= Alkyl, halogen<br>$R^1$= $^i$Pr, cyclohexyl and cyclopentyl<br>$R^2$= COOEt, $NO_2$ | 398 |
| 28 | 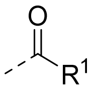 | Cyclization | 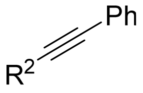 | 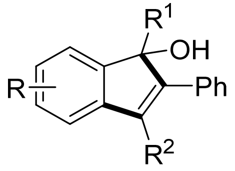 | Substrate (1 mmol), phenome/alkyne (1:1.2 or 1.2:1), $[RhCp^*Cl_2]_2$ (0.5 mol%), $AgSbF_6$ (2 mol%), $Cu(OAc)_2$ (2.1 equiv.), PhCl, 120 °C, 16 h.<br>10 Examples; Yield: 49-90%<br>R= Br, $CF_3$; $R^1$= Alkyl, Ph, 3,5-$(CF_3)C_6H_3$ | 372 |

| | | | | | | |
|---|---|---|---|---|---|---|
| | | | | | $R^2$=Ph, alkyl | |
| 29 | 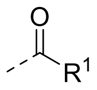 | Cyclization | 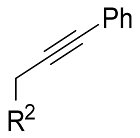 | 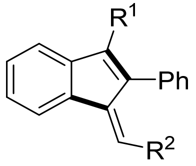 | Substrate (1 mmol), phenome/ alkyne (1:1.2 or 1.2:1), $[RhCp^*Cl_2]_2$ (2.5 mol%), $AgSbF_6$ (10 mol%), $Cu(OAc)_2$ (2.1 equiv.), 1,4-dioxane, 140 °C, 16 h.<br>6 Examples; Yield: 51-80%<br>$R_1$= Ph, anisol<br>$R^2$= Me, Ph | [372] |
| 30 | 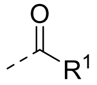 | Cyclization | 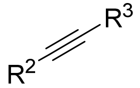 | 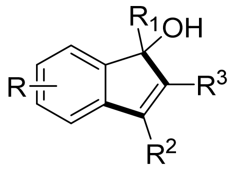 | Substrate (1 mmol), alkyne (1.2 mmol), $[RuCl_2(p\text{-cymene})]_2$ (2 mol%), $AgSbF_6$ (8 or 20 mol%), $Cu(OAc)_2{\cdot}H_2O$ (25 mol%), DCE, 120 °C.<br>21 Examples; Yield: 69-94%<br>R= Me, OMe, halogen; $R^2$= Ph; $R^3$= Alkyl, $SiMe_3$<br>dehydration product with Ag > 8 mol% | [373, 374] |
| 31 | 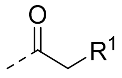 | Cyclization | 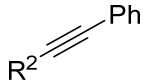 | 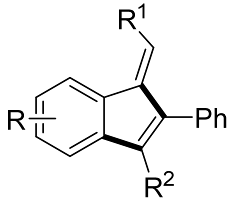 | Substrate (1 mmol), phenome/ alkyne (1:1.2 or 1.2:1), $[RhCp^*Cl_2]_2$ (2.5 mol%), $AgSbF_6$ (10 mol%), $Cu(OAc)_2$ (2.1 equiv.), 1,4-dioxane, 140 °C, 16 h.<br>8 Examples; Yield: 40-70%<br>$R^1$= Me; $R^2$= Ph, alkyl | [372] |
| 32 | 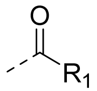 | Halogenation | NXS | 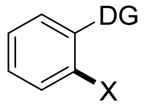 | Substrate (1 mmol), NXS (1.6 equiv.), $[RhCp^*Cl_2]_2$ (2.5 mol%), $AgSbF_6$ (10 mol%), PivOH (1.1 equiv.), 1,2-DCE, 60-120 °C, 16-48 h.<br>7 Examples; Yield: 49-78%<br>$R_1$= Alkyl, OEt; X= Br, I | [380] |

| | | | | | | |
|---|---|---|---|---|---|---|
| 33 | 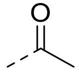 | Hydroarylative Cyclization | 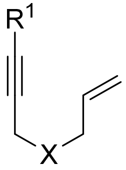 | 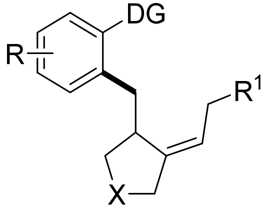 | Substrate (0.32 mmol), enyne (0.30 mmol), $CoBr_2$ (5 mol%), dppp (5 mol%), Zn (10 mol%), $ZnI_2$ (20 mol%), $Ch_2Cl_2$, 40 °C, 2 h.<br>18 Examples; Yield: 71-94%<br>R= Me, OMe, halogen, $CF_3$; $R^1$= Aryl, Ph, thiophen<br>X= O, NTs, $C(CO_2Me)_2$ | 384 |
| 34 | 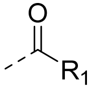 | Hydroarylative Cyclization | 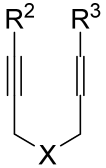 | 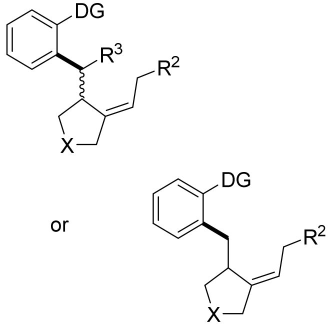 | Substrate (3 equiv.), diyne (1 equiv.), [Rh(biphep)]$BF_4$ (5 mol%), $CH_2Cl_2$, r.t., 30 min.<br>7 Examples; Yield: 55-99%<br>$R^1$= Me, Ph<br>$R^2$& $R^3$ = Me, Ph<br>X= NTs, $C(CO_2Bn)_2$, $[C(CO_2Et)_2]_2$ | 377 |
| 35 | 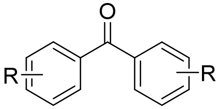 | Hydroxylation | TFA | 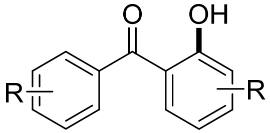 | Substrate (0.5 mmol), $Pd(OAc)_2$ (5 mol%), $PhI(OTFA)_2$ (1 mmol), DCE, 80 °C, 2 h.<br>20 Examples; Yield: 70-86%<br>R= Me, OMe, halogen | 378 |
| 36 | 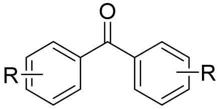 | Hydroxylation | TFA | 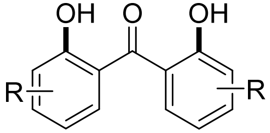 | Substrate (0.4 mmol), $Pd(OAc)_2$ (5 mol%), BTI (2 eq,) or $K_2S_2O_8$ (2 equiv.), TFA , 50 °C.<br>5 Examples; Yield: 21-77%<br>R= Me, OMe | 364 |
| 37 | 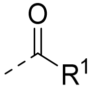 | Hydroxylation | TFA/TFAA | 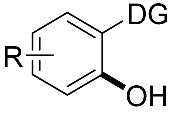 | Substrate (1.0 mmol), $[Ru(O_2CMes)_2(p\text{-cymene})]$ (1-5 mol%), $PhI(OAc)_2$ (1.2 equiv.), TFA/TFAA (3/2), 120 °C.<br>15 Examples; Yield: 57-83%<br>R= Alkyl, alkoxy, halogen<br>$R^1$= $^t$Bu | 379 |

| | | | | | | |
|---|---|---|---|---|---|---|
| 38 | 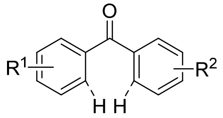 | Oxidative arylation | - | 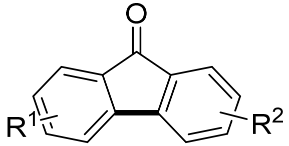 | Substrate (0.2 mmol), $Pd(OAc)_2$ (5.0 mol%), $Ag_2O$ (1.5 equiv.), $K_2CO_3$ (2.5 equiv.), TFA, 140 °C, 24 h.<br>21 Examples; Yield: 28-94%<br>$R^1$& $R^2$= Me, OMe, OH, halogen | 385 |
| 39 | 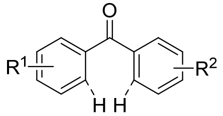 | Oxidative arylation | - | 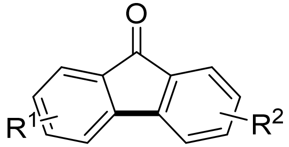 | Substrate (1.0 mmol), $Pd(OAc)_2$ (10 mol%), $Ag_2O$ (1.5 equiv.), TFA, 140 °C, 24 h.<br>15 Examples; Yield: 68-91%<br>$R^1$& $R^2$= Me, OMe, OH, halogen | 386 |

## Hydroxyl- and Phenol- based derivatives

In general hydroxyl directed C-H functionalization is restricted to the *ortho* position due to the electron-donating ability of the oxygen group. To control the regioselectivity for *meta*- alkenylation, a modified phenol molecule containing a hydrolytic removable CN moiety was published by the group of Yu (Table 16, Entry 17).[401]

An asymmetric rhodium catalysed cyclization using several directing groups such as ether, sulfide and sulfoxide groups to synthesize seven or eight membered heterocycles via olefin hydroacylation was reported by the group of Dong (Table 16, Entry 15).**[402]** During mechanistic studies they showed the catalytic process contains several steps (C-H bond activation, olefination insertion, and reductive elimination) to perform intramolecular cyclization reactions with controlled regioselectivity, which is highly depending on the catalyst-ligand and the substrate structure.

The group of Yu presented a catalytic system, which consists of $Pd(OAc)_2$ pre-catalyst, $Li_2CO_3$ as base and $Ph(IOAc)_2$ as oxidantwith a hydroxyl directing moiety to synthesize dihydrobenzofurans (Table 16, Entry 13)**.**[403] A $Pd(OAc)_2$ pre-catalystand similar starting materials combined with amino acid ligands promotes a noval carbonylation reaction for the synthesis of 1-isochromanone scaffolds. The optimization of the reaction conditions was limited by decomposition of the Pd(II) to palladium black in the presence of CO. To overcome this obstacle, different amino acid ligands were tested and (+)-menthyl($O_2C$)-Leu-OH and elevated temperature of 110 °C gave an increased overall yield of 50% (35 to 85%) (Table 16, Entry 12).**[404]**

An enantioselective fluorination reaction for a broad range of acyclic alcohols *via* an *in-situ* generation of a boronic acid monoester, which will act as a removable directing group was presented by the group of Toste (Table 16, Entry 14)**.**[405] After condensation between the boronic acid and the primary alcohol a γ selective fluorination by Selectfluor at r.t. catalysed by S-(AdDIP), a phosphate bearing 4-(1-adamantyl)-2,6-diisopropyl BINOL ligand system gives the final products in high yields (94%) and excellent enantioselectivity (up to 94% ee).

Hydroxy- directed arylation reactions can be divided into three different types (1) an *ortho* arylation catalysed by $[RhCl(PPh)_3]_3$ with a phosphinite co-catalyst (Table 16, Entry 8 & 9)[406, 407] or (2) a regioselective arylation on the remote ring strongly influenced from the reaction conditions (Table 16, Entry 7).[408] Diarylation on the remote ring system was obtained by $PdCl_2$, $Cs_2CO_3$ in DMF at 100 °C. In contrast, the mono-arylated compound requires anhydrous conditions by adding a molecular sieve and is promoted by $Pd(OAc)_2$. (3) An intramolecular arylation *via* $Pd(PPh_3)_3$, by the insertion of Pd into the C-X (X= Br, I) bond followed by the C-C bond formation reactions another procedure to form a new C-C bond is reported (Table 16, Entry 10).[409]

A metal- catalysed oxidative annulation of 2-aryl-3-hydroxy-2-cyclohexenones was reported to provide benzopyrans in good yields up to 78%. During the evaluation of reaction conditions, various pre-catalyst were tested and best results were obtained by $Pd(OAc)_2$ for electron-deficient alkenes including vinyl ketone. In the case of $[RuCl_2(p\text{-}cymene)]_2$ increased yields were obtained with methyl acrylate, *N,N*-dimethylacrylamide and acrylonitrile were determined (Table 16, Entry 1).[410]

A switchable C-H functionalization of substrate molecules, containing different reactive C-H bonds will give access to a variety of products from the same starting material. In this approach 2-aryl cyclic 1,3-dicarbonyl compounds that contains two position for activation were used and the product selectivity was controlled by the catalyst-ligand structure. A palladium−*N*-heterocyclic carbine complex promotes the oxidative annulation with alkynes to spiroindenes in good yields (87%) within 5h. In comparison, $[RuCl_2(p\text{-cymene})_2]$ gave in 22h selectively the benzopyran product using $Cu(OAc)_2 \cdot H_2O$ as oxidant in m-xylene/$H_2O$ (10:1) solvent mixture (Table 16, Entry 5 & 6).[411]

**Table 16: Hydroxyl- and phenol- based directing groups**

| Entry | Directing group | Type of transformation | Coupling partner | Typical product structure | Comments | Ref |
|---|---|---|---|---|---|---|
| 1 | 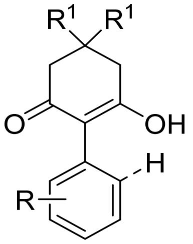 | Alkenylation/ Cyclization | 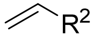 | 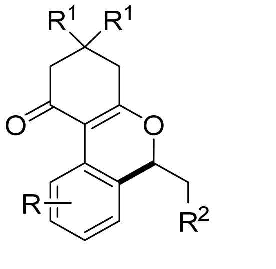 | Substrate (0.5 mmol), alkene (1.5 equiv.), $Pd(OAc)_2$ (5 mol%), $Cu(OAc)_2$ (2.1 equiv.), DMF, 120 °C, 2-5 h.<br>$[RuCl_2(p\text{-cymene})]_2$ (2.5 mol%), $Cu(OAc)_2 \cdot H_2O$ (2.1 equiv.), $K_2CO_3$ (2 equiv.), $^t$Amyl-OH, 90 °C.<br>22 Examples; Yield: 45-76%<br>R= Me, F,<br>$R^1$= Me<br>$R^2$= $SO_2Ph$, COOMe, CN | 410 |
| 2 | 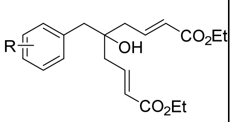 | Alkenylation/ Cyclization | | 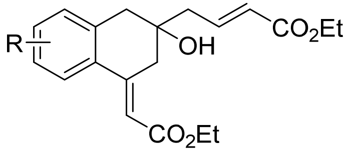 | Substrate (1 equiv.), $Pd(OAc)_2$ (10 mol%), L1 (20 mol%), AgOAc (4.0 equiv.), $Li_2CO_3$ (2.0 equiv.), DCE, 90 °C, 64 h.<br>L1= 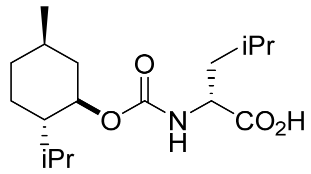<br>11 Examples; Yield: 30-98%<br>dr=91:9<br>R= OMe, Me, $CF_3$ | 412 |
| 3 | 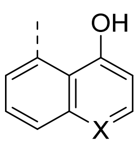 | Annulation | 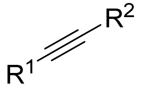 | 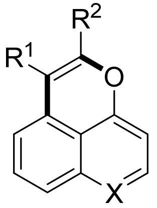 | Substrate (1 mmol), alkyne (0.5 mmol), $[RuCl_2(p\text{-cymene})]_2$ (2.5 mol%), $Cu(OAc)_2 \cdot H_2O$ (1 mmol), m-xylene, 80-110 °C.<br>24 Examples; Yield: 48-81%<br>$R^1$& $R^2$ = Aryl substituted with Me, $CF_3$, OMe, halogen<br>X= O, NMe | 413 |

| | | | | | | |
|---|---|---|---|---|---|---|
| 4 | 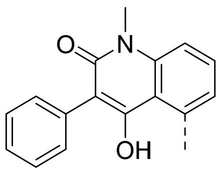 | Annulation | 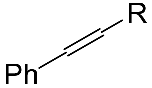 | 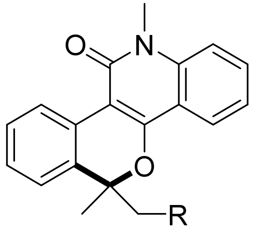 | Substrate (0.5 mmol), Pd $(OAc)_2$ (5 mol%), $Cu(OAc)_2$ (2.1 equiv.), DMF, 120 °C, 3-15 h.<br>5 Examples; Yield: 32-86%<br>R= COOMe, COMe, CN, $SO_2Ph$ | 411 |
| 5 | 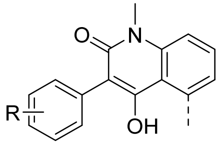 | Annulation | 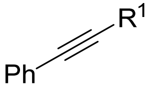 | 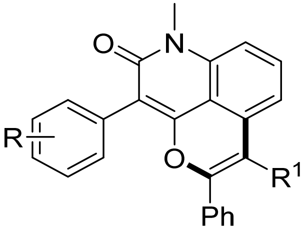 | Substrate (0.5 mmol), $[RuCl_2(p\text{-cymene})]_2$ (5 mol%), $Cu(OAc)_2 \cdot H2O$ (2.1 equiv.), DMF, 90 °C, 1-5 h.<br>9 Examples; Yield: 32-88%<br>R= Me, OMe, COOMe<br>$R^1$= Alkyl, OMe, COOMe | 411 |
| 6 | 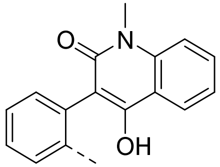 | Annulation | 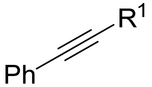 | 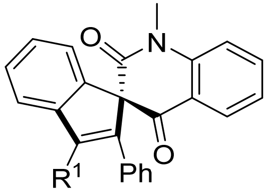 | Substrate (0.5 mmol), alkyne (1.5 equiv.), PEPPSI-IPr (2.5 mol%), $Cu(OAc)_2$ (2.1equiv.), DMF, 120 °C, 2-5 h.<br>9 Examples; Yield: 45-87%<br>$R^1$= Alkyl, Ph, aryl | 411 |
| 7 | 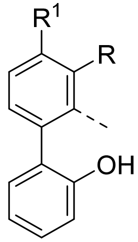 | Arylation | 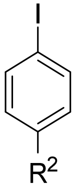 | 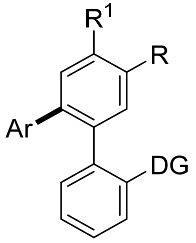 | Substrate (1 mmol), aryl iodide (1.2 mmol), $Pd(OAc)_2$ (0.05 mmol), $Cs_2CO_3$ (1.2 mmol), molecular sieves 4 A (200 mg), DMF (5 mL),<br>100 °C.<br>8 Examples; Yield: 70-88%<br>R= OMe, $NO_2$<br>$R^1$= Me<br>$R^2$= OMe | 408 |

| | | | | | | |
|---|---|---|---|---|---|---|
| 8 | 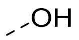 | Arylation | 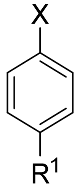 | 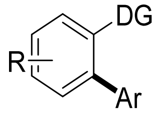 | Substrate (1 mmol), aryl halide (1.5 mmol), $[RhCl(PPH_3)_3]$ (0.05 mmol), $PR_2(OAr)$ (0.15 mmol), $Cs_2CO_3$ (1.7 mmol) toluene, reflux, $N_2$, 18 h.<br>13 Examples, Yield: 21-100%<br>R= Alkyl<br>$R^1$= Me, OMe, carbonyl<br>X= Br, Cl | 406 |
| 9 | 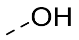 | Arylation | 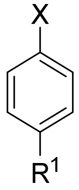 | 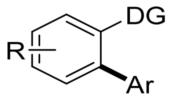 | Substrate (1 mmol), aryl halide (1.5 mmol), $[RhCl(PPh_3)_3]$ (0.05 mmol), $PR_2(OAr)$ (0.15 mmol), $Cs_2CO_3$ (1.7 mmol), toluene, reflux, 18 h, $N_2$.<br>22 Examples, Yield: 15-96%<br>R= Alkyl<br>$R^1$= Me, OMe, RCO, $NMe_2$, halogen<br>X= Br, Cl<br>Hetercyclic halides tolerated | 407 |
| 10 | 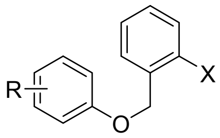 | Intramolecular Arylation | | 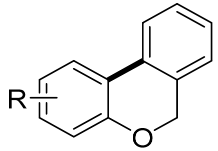 | Substrate (1 equiv.), $Pd(PPh_3)_4$ (0.2 equiv.), $^tBuOK$ (3 equiv.), DMA, 95 °C, 2 d.<br>5 Examples, Yield 90 %<br>R= OH, Me<br>X= Br, Cl | 409 |
| 11 | 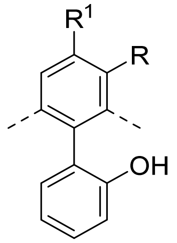 | Diarylation | 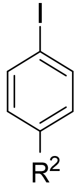 | 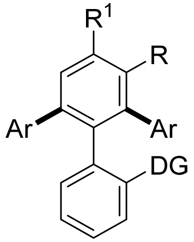 | Substrate (1 mmol), aryl halide (1.2 mmol), $PdCl_2$, (0.05 mmol), $Cs_2CO_3$ (4 mmol),<br>DMF (5 mL), 100 °C.<br>8 Examples; Yield: 57-87%<br>$R^1$= Me<br>$R^2$= OMe | 408 |

| | | | | | | |
|---|---|---|---|---|---|---|
| 12 | 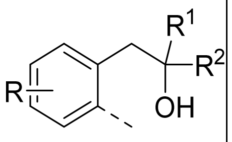 | Carbonylation/ lactonizaiton | CO | 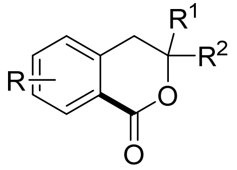 | Substrate (1.0 equiv.), $Pd(OAc)_2$ (10 mol%), (+)-Men-Leu-OH (20 mol%), AgOAc (3.0 equiv.), $Li_2CO_3$ (1.0 equiv.), CO (1 atm), DCM, 110 °C, 48 h.<br>20 Examples; Yield: 51-94%<br>R= Me, OMe, halogen<br>$R^1$& $R^2$= Alkyl | 404 |
| 13 | 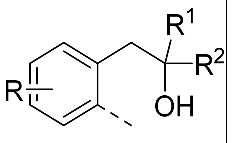 | Cyclization/ lactonization | | 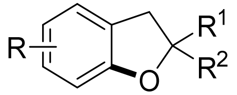 | Substrate (0.2 mmol), $Pd(OAc)_2$ (0.01 mmol), $PhI(OAc)_2$ (0.3 mmol), $Li_2CO_3$ (0.3 mmol), $C_6F_6$, 100 °C, 36 h.<br>20 Examples; Yield: 42-91%<br>R= Me, OMe, halogen<br>$R^1$& $R^2$= Alkyl, Ph, Bn, COOR | 403 |
| 14 | 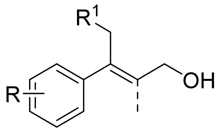 | Fluorination | Selcetfluor | 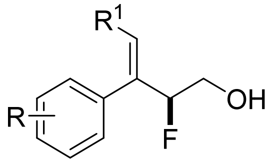 | Substrate (1 equiv.), (S)-AdDIP (10 mol%), Selcetfluor (1.3 equiv.), $Na_2HPO_4$ (4.0 equiv.), *p*-tolylboronic acid (1.0 equiv.), $MgSO_4$ (40mg/ 0.10 mmol), *p*- xylene/ Etcyclohexane (1:1), 0.1 M, r.t., 16-96 h.<br>15 Examples; Yield: 47-85% (ee 94%)<br>R= Me, OMe, halogen, $CF_3$<br>$R^1$= Alkyl | 405 |
| 15 | 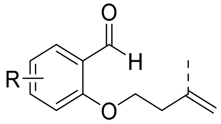 | Intramolecular Olefin Hydroacylation | | 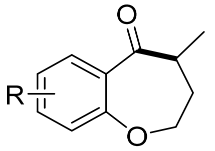 | Substrate (1 equiv.), [RH((R,R)-Me-DuPHOS)]$BF_4$ (5 mol %), $CH_2Cl_2$, r.t., 24 h.<br>8 Examples; Yield: 80-95%<br>R= Me, OMe, halogen | 402 |
| 16 | 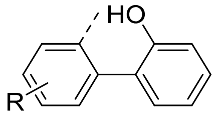 | Carbonylation - lactonizaiton | CO | 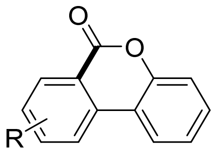 | Substrate (1 equiv.), $[RuCl_2(p\text{-cymene})]_2$ (4 mol%); HIPrCl (12 mol%); PivOH (10 mol%); $Cs_2CO_3$ (3.0 equiv.), mesitylene, 100 °C, CO (ballon), $O_2$ (balloon)<br>15 Examples; Yield: 28-96% | 414 |

| | | | | | | |
|---|---|---|---|---|---|---|
| | | | | | R= Me, OMe, $CF_3$, COOEt, CN, Ac, halogen | |
| 17 | 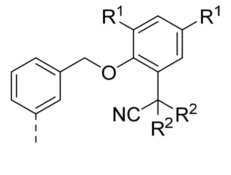 | Alkenylation | 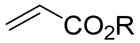 | 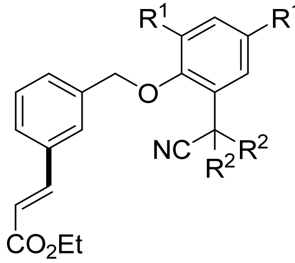 | Substrate (0.05 mmol), acrylate (1.5 equiv.), $Pd(OPiv)_2$ (10 mol%), Ag(OPiv) (3 equiv.), DCE, 90 °C, 18 h.<br>40 Examples; Yield: 4-86 %<br>R= alkyl, Bn<br>$R^1$& $R^2$= Alkyl | 401 |

## Oxime and Oxime derivatives as directing groups in C-H activation chemistry

Oxime containing directing groups are manifold and can generally be subdivided in ketoximes, aldoximes and their ethers and esters. In 2012, one of the first rhodium-cataylzed oxidative *ortho*-acylation of aryl ketoxime ethers with aryl- and alkyl aldehydes via C-H bond activation was demonstrated by Yang et al. Noteworthy; the resulting monoacylated products proceeded to a Rh-catalyzed addition of the second ortho C-H bond to aldehydes, when highly deficient benzaldehydes are employed as coupling partners. In this case, no mono-acylation products were observed and two C-C bonds were generated simultaneously (Table 17, Entry 1 &2).[415]Another example for a Rh(III) catalyzed ketoxime ether directed aromatic C-H bond activation is the oxidative coupling to alkenes reported by Tsai et al. This procedure demonstrated the at this time unknown transformation with nonactivated olefins for Rh catalysts (Table 17, Entry 3).[416] Further, it is also substantial to take advantage of the ubiquity of C-H bonds for C-N coupling. The Li group achieved an amidation of arenes bearing chelating groups by applying *N*-arene sulfonated imides as amidation reagents without the need for the addition of a base (Table 17, Entry 4).[417] Another C-N bond formation is realized by applying 1,4,2-dioxazolone as coupling partner. This C-H amidation was published by Park et al. in 2015 (Table 17, Entry 5).[418] The same coupling partner was used in a protocol of the Li group. In this case the applicability to the late stage functionalization of natural products was demonstrated(Table 17, Entry 2).[419] Further, the Fu group were the first, who reported a Rh-catalyzed directed C−H cyanation as a practical method for the synthesis of aromatic nitriles (Table 17, Entry 6.[212] In 2015 a rhodium(III)-catalyzed coupling of aromatic ketoxime ethers with 2-vinyloxirane via directed C–H activation was describes by Wen et al. Remarkable, this procedure contains an allylation and a concomitant epoxide opening (Table 17, Entry 9).[420] In 2006 a palladium-catalyzed procedure for an intramolecular amidation via cascade C-H activation/nitrene insertion was published by the Che group (Table 17, Entry 10).[421] Lou et al. described a palladium catalyzed mild, versatile nitrate promoted C-H bond fluorination in 2014 (Table 17, Entry 13).[422] However, versatile functionalizations of the ketoxime ether scaffold were demonstrated. For instance a ligand-promoted Pd-catalyzed hydroxylation (Table 17, Entry 15)[423], a chelation assisted, regiospecific nitration (Table 17, Entry 16)[424], a direct selenylation of arenes with electrophilic selenenyl chlorides or diselenides (Table 17, Entry 17)[425], and even a rhodium catalyzed oxime ether directed heteroarylation was presented. This procedure provides a straightforward access to bi(hetero)aryl scaffolds (Table 17, Entry 14).[426] Even the synthesis of highly substituted benzofuranes by activation of a sterically hindered C-H bond has been demonstrated. Benzofurane derivatives, frequently used as building blocks in organic materials, are an important class of heterocycles found in many natural and biologically active molecules. This rhodium(III)-catalyzed C-H activation starts from meta-substituted hydroxybenzenes and alkynes (Table 17, Entry 19).[427]The Sanford group reported in 2004 that unactivated $sp^3$ C-H bonds of oxime substrates undergo highly regio- and chemoselective palladium catalyzed oxygenation under acidic conditions with $PhI(OAc)_2$ as stochiometric oxidant(Table 17, Entry 20).[428] A different example for the application of a hypervalent iodinereagent, is the palladium catalyzed *β*-arylation of oxime ethers using diaryliodonium salts as key arylation reagent described by Peng et al. (Table 17, Entry 21).[429] In 2014 a process applying an unprecious metal was described by the Ellman group. An airstable cationic Co(III) catalyst for a one-step syntheses of furans by C−H bond additions to aldehydes followed by in situ cyclization and aromatization was demonstrated. This protocol is the first examples of Co(III)-catalyzed additions to aldehydes(Table 17, Entry 24).[430] Further, in 2013 a decarboxylative C-H activation in form of an *ortho*-acylation with α-keto acids under ammonium persulfate as a convenient oxidant was described by Kim et al.(Table 17, Entry 1).[431]

**Table 17: Ketoxim ether directing groups**

| Entry | Directing group | Type of transformation | Coupling partner | Typical product structure | Comments | Ref |
|---|---|---|---|---|---|---|
| 1 | 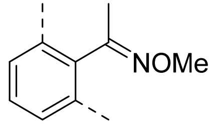 | Acylation | 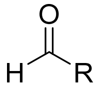 | 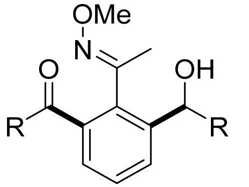 | Substrate (1 eq), aldehyde (4 eq), $[Cp^*RhCl_2]_2$ (0.1 eq), $AgSbF_6$ (0.4 eq), $AgCO_3$ (2.5 eq), DCM (0.2 M), 85 °C, 24 h, highly electron deficient benzaldehyde required<br>3 examples, 41-60 % yield<br>R: PhCOOMe, PhCHO, $PhCH_3CO$ | 415 |
| 2 | 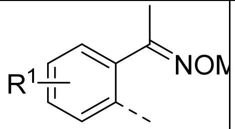 | Acylation | 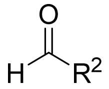 | 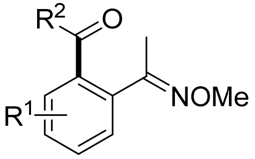 | Substrate (1 eq), aldehyde (2.5 eq), $[Cp^*RhCl_2]_2$ (0.05 eq), $AgSbF_6$ (0.2 eq), $AgCO_3$ (2.5 eq), DCM (0.2 M), 85 °C, 24 h<br>18 examples, 21-82 % yield<br>$R^1$: Me, $OCH_3$, COOMe, $CF_3$, F, COOMe<br>$R^2$: Ph, PhMe, PhOMe, furane, thiophene, cyclohexanone | 415 |
| 3 | | Alkenylation | 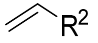 | 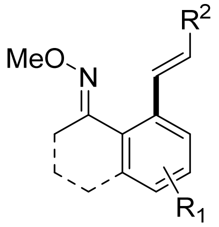 | Substrate (1 equiv), alkene (3 equiv), $[Cp^*RhCl_2]_2$ (5 mol %), $AgSbF_6$ (20 mol%), $Cu(OAc)_2$ (2.1 equiv), and THF (0.1 M) in a sealed vial for 20 h at 75 °C<br>16 examples, 46-96 % yield<br>$R^1$: Alkyl, aryl, COOEt<br>$R^2$: *i*-Propyl, cyclohexanone, (alkyl)halogenide, Ph, COOEt, OAc, Bu | 416 |
| 4 | | Amidation | 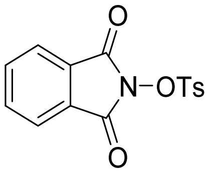 | 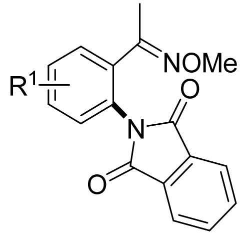 | Substrate (0.2 mmol), N-OTs phthalimide (0.3 mmol), $[RhCp^*Cl_2]_2$(5 mol%), $AgSbF_6$ (40 mol%), DCE (2 mL), 100 °C,20 h, sealed tube under argon<br>23 examples, 56-60 %<br>$R^1$: Ph, OMe | 417 |

| | | | | | | |
|---|---|---|---|---|---|---|
| 5 | | | 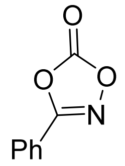 | 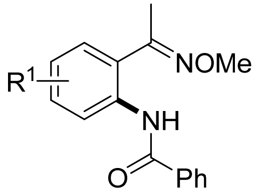 | Substrate (0.2 mmol), coupling partner (0.22 mmol); large scale: substrate (50 mmol), $[RhCp^*Cl_2]_2$ (0.5 mol%), $AgNTf_2$ (2 mol%), ethyl acetate, 60 °C, 18 h<br>2 examples, 51-64 % recrystallization yield (Br)<br>$R^1$: H, Br | 418 |
| 6 | | Cyanation | 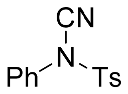 | 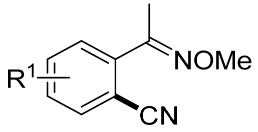 | Substrate (0.2 mmol), $[RhCp^*(CH_3CN)_3](SbF_6)_2$ (5 mol %), and $Ag_2CO_3$ (20 mol%), dioxane, Ar, 24 h, heterocycles tolerated<br>25 examples, 53-94 % yield<br>$R^1$: Me, halogenide, COOMe, OMe, OTs, NHAc, OH, alkoxy, sugar residues | 212 |
| 7 | | Cyclization | 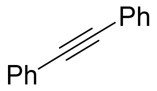 | 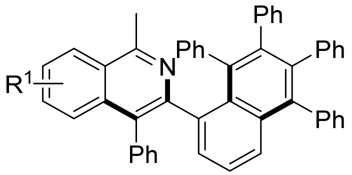 | Substrate (1 eq), alkyne (3 eq), $[Cp^*RhCl_2]_2$ (2.5 mol%), 2 eq. $Cu(OAc)_2$, 0.5 eq. NaOAc, MeOH, 110 °C, 2h, heterocycles tolerated<br>16 examples, 23-96 % yield<br>$R^1$: OH, Me, OMe, NHAc, $CF_3$ | 432 |
| 8 | | Diazo coupling | 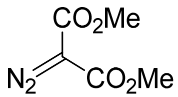 | 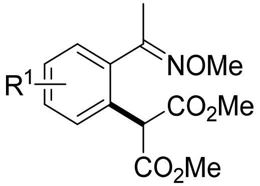 | Substrate (0.4 mmol), diazomalonat (0.2 mmol), $[Cp^*RhCl_2]_2$ (1.25 mol%), AgOAc (7.5 mol %), MeOH, 60 °C, 12 h<br>13 examples, 36-93 % yield<br>$R^1$: OMe, $CF_3$, $SO_2Me$, $CO_2Et$, Br | 153 |
| 9 | 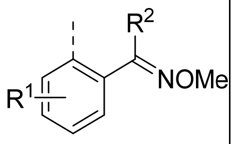 | Allylation | 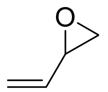 | 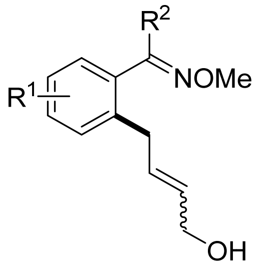 | Substrate (0.2 mmol, 1.0 equiv), vinyloxirane (1.2 equiv), $[RhCp^*Cl_2]_2$(3 mol%), $AgSbF_6$ (12 mol%), THF (2 ml), 50 °C, 12 h, $Cu(OAc)_2$ (0.5 equiv)<br>8 examples,51-84 % yield<br>$R^1$: Me, halogenide, $NO_2$<br>$R^2$: Me, alkyl, (cyclic alkyl) | 420 |

| | | | | | | |
|---|---|---|---|---|---|---|
| 10 | | Amidation | Amide ($H_2NCOR^3$) | 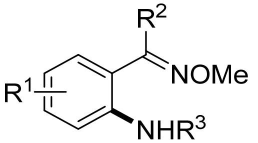 | Substrate (1 eq), amide (1.2 eq), $Pd(OAc)_2$ (5 mol%), $K_2S_2O_8$, DCE, 80 °C, 14-20 h<br>9 examples, 87-96 % yield<br>$R^1$: Me, OMe, halogenide<br>$R^2$: H, Me<br>$R^3$: $CO_2CH_3$, $COCF_3$, $CO^2t$Bu, $SO_2CH_3$, $SO_2$($p$-Cl-$C_6H_4$), COCHC=$CHC_6H_4$ | 421 |
| 11 | | Arylation | 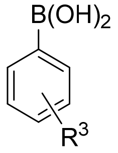 | 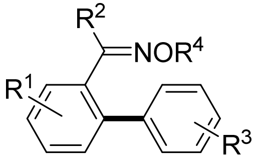 | Substrate (0.2 mmol), boronic acid (0.5 eq every 2 hours)$Pd(OAc)_2$ (10 mol%), $Cu(OTf)_2$ (2 eq), $O_2$ (1 atm), dioxane, 100 °C, 24 h<br>28 examples, 5-87 % yield<br>$R^1$: Me; $R^2$: Me, alkyl (cyclized); $R^3$: H, Me, $t$Bu, halogenide, OMe, $OCF_3$; $R^4$: Me, Bn, Ph, Ac, Bz, Piv | 433 |
| 12 | | Arylation | | 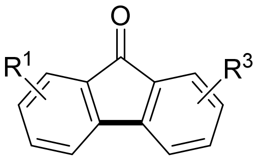 | Substrate (0.2 mmol), boronic acid (0.5 eq every 2 hours)<br>One pot procedure, 1) $Pd(OAc)_2$ (10 mol%), $Cu(OTf)_2$ (2.5 eq), 3 A-MS, dioxane, 90 °C; 2) TfOH (2 eq); 3) HCl (6 M)<br>8 examples, 39-62 % yield<br>$R^1$: Me; $R^3$: Me, $t$Bu, OMe | 433 |
| 13 | | Fluorination | 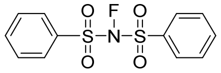 | 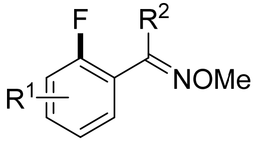 | Substrate (0.3 mmol), $[Pd_2(dba)_3]$ (5 mol%), NFSI (2.0 equiv), $KNO_3$ (30 mol%), 3 ml $CH_3NO_2$,NFSI (N-fluorobenzenesulfonimide)<br>27 examples, 65-87 % yield<br>$R^1$: Me, OMe, OBn, Ph, halogenide, COOMe, $SO_2$Me, CN, $NO_2$, $CF_3$, naphtyl; $R^2$: Alkyl, Ph | 422 |

| | | | | | | |
|---|---|---|---|---|---|---|
| 14 | | Heteroarylation | 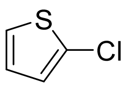 | 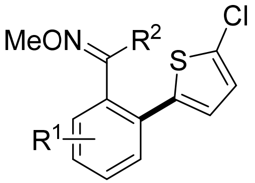 | Substrate (0.25 mmol), 2-chlorothiophene (0.375 mmol), $[Cp^*RhCl_2]_2$ (2.5 mol%), $Ag_2CO_3$ (2.2 equiv.), $Cu(TFA)_2$, $H_2O$ (20 mol%), DCE (0.6 mL) at 150 ° C for 24 hours under an $N_2$atmosphere<br>29 examples, 44-70 % yield<br>$R^1$: COOEt, $NO_2$; $R^2$: Me; Ph | [426] |
| 15 | | Hydroxylation | Oxone | 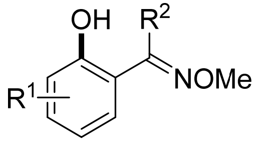 | Substrate (0.3 mmol), $Pd(OAc)_2$ (5 mol%), $PPh_3$ (10 mol%), $KHSO_{5,}$ Oxone (1.2 equiv), $CHCl_2CHCl_2$ (1 mL) was stirred at 100 °C for 24 h under air<br>43 examples, 32-98 % yield<br>$R^1$: H, Me, Ph, naphtyl, OMe, *t*-Bu, F; $R^2$: Aryl, alkyl, cyclized alkyl, COH, $CH_2CH_2COOCH_3$, alkylhalogenide | [423] |
| 16 | | Nitration | $AgNO_2$ | 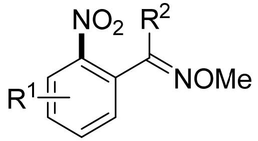 | Substrate (0.3 mmol), $Pd(OCOCF_3)_2$ (10 mol%) (0.03 mmol), $AgNO_2$ (2 eq ) (0.6 mmol), $K_2S_2O_8$ (2 eq) (0.6 mmol) in 3.5 mL of DCE at 110 °C for 48 h<br>Bicycles ($R^2$-) potential substrates<br>23 examples, 42-90 % yield<br>$R^1$: Me, OMe, halogenide, $NO_2$, $SO_2Me$, OBn<br>$R^2$: Me, (cyclized) alkyl, Ph | [424] |
| 17 | | Selenylation | PhSe-Cl<br>PhSe-SePh | 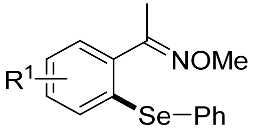 | Substrate (0.2 mmol), coupling partner (0.24 mmol), $[Cp^*RhCl_2]_2$ (4 mol%), $AgSbF_6$ (1.5 equiv), NaOAc (1.2 equiv), THF (3 mL), 60 °C, 20 h, sealed tube under $N_2$.<br>29 examples, 45-94 %<br>$R^1$: OMe, *t*Bu, COOMe, halogenide, alkyl, Ph, $CF_3$ | [425] |

| | | | | | | |
|---|---|---|---|---|---|---|
| 18 | 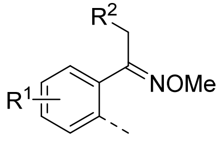 | Amidation, Cyclization | 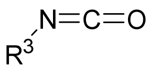 | 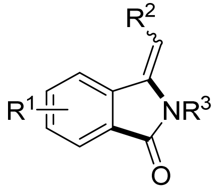 | Substrate (0.2 mmol), isocyanate (0.3 mmol),$[Rh(CH_3CN)_3(Cp^*)][SbF_6]_2$ (5 mol%), DCE (1 mL), 100 °C, 12 h, oxime serves first as DG, then as leaving group<br>17 examples, 74-92 % yield<br>$R^1$: $CF_3$, COOMe, OMe, halogenide; $R^2$: Me, alkyl, aryl<br>$R^3$: PhMe | [434] |
| 19 | 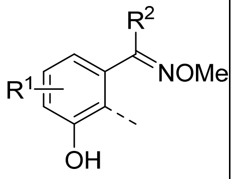 | Cyclization | 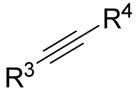 | 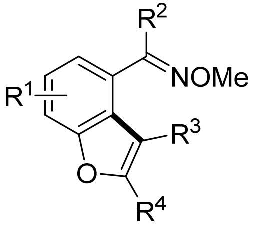 | Substrate (0.20 mmol), alkyne 2 (0.30 mmol) Cu-$(OAc)_2 \cdot H_2O$ (0.70 mmol) and $[RhCp^*Cl_2]_2$ (0.004 mmol) in MeOH (0.2 M) under $N_2$.<br>Route to *meta* and *ortho* substituted benzofurans<br>22 examples, 34-94 % yield<br>$R^1$: OMe, halogenide ; $R^2$: H, Me, cyclyzed alkyl<br>$R^3$: Ph, PhMe, PhMeO, Ph-halogenide | [427] |
| 20 | 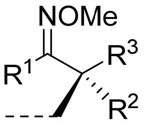 | Acetylation | $PhI(OAc)_2$ | 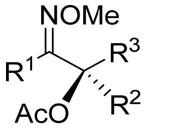 | Substrate (1 eq), 1.1 equiv of $PhI(OAc)_2$,5 mol% $Pd(OAc)_2$, 50% AcOH/50% $Ac_2O$, 100 °C, 1.5-3.5 h.<br>13 examples, 39-68 % yield<br>$R^1$: Alkyl<br>$R^2$, $R^3$: H, OAc | [428] |
| 21 | 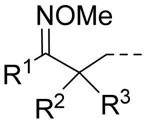 | Arylation | $Ar\text{-}I^+OTf^-$ | 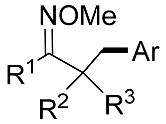 | Substrate (0.25 mmol), diaryliodonium salts (0.25 mmol), $Pd(OAc)_2$ (5 mol%), PivOH (0.6 eq), $Ag_2CO_3$ (2 eq), DCE:HFIP (3:1), 85 °C, 5 h<br>6 examples, 65-83 % yield<br>$R^1$: H, Me, alkyl<br>$R^2$: H, Me, COOEt | [429] |

| | | | | | | |
|---|---|---|---|---|---|---|
| 22 | 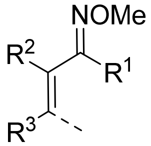 | Amidation, Cyclization | R$^4$—N=C=O | 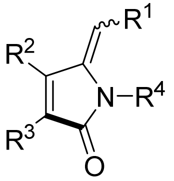 | Substrate (0.2 mmol), isocyanate (0.3 mmol), [RhCp*($CH_3CN$)$_3$]($SbF_6$)$_2$ (5 mol%), DCE, 100 °C, 12 h<br>19 examples, 47-93 % yield<br>R$^1$: H, Me; R$^2$: H, Me; R$^3$: Me, Ph, PhMe, PhMeO, Ph-halogenide, cyclized alkyl, naphtyl<br>R$^4$: PhMe, PhOMe, $PhNO_2$, $PhCF_3$, PhCOOEt, Ph-halogenide, naphtyl, alkyl, cyclohexane | 435 |
| 23 | | Cyclization | 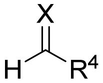 | 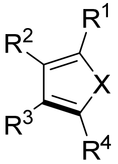 | Substrate (0.2 mmol), aldehyde (0.4 mmol) or imine (0.4 mmol), [Cp*$RhCl_2$]$_2$ / $AgSbF_6$ (5 mol%), $AgBF_4$ (16 mol%), THF, 90 °C, 24 h<br>28 examples, 41-89 % yield<br>R$^1$: Me, alkyl; R$^2$: Me, alkyl, Ph, tolyl; R$^3$: H, Me<br>R$^4$: COOEt, PhMe, PhOMe, $PhNO_2$, $PhCF_3$, PhCOOEt, Ph-halogenide, naphtyl, alkyl, cyclohexane<br>X= O, NTs | 436 |
| 24 | | Cyclization | R$^4$–CHO | 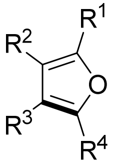 | Co(III) conditions:<br>Substrate (0.20 mmol), aldehyde (0.40 mmol), [Cp*$CoCl_2$]$_2$ (10 mol%), and AcOH (10 mol%) in 1,4-dichloroethane (2.0 M) for 24 h<br>12 examples, 25-84 % yield<br>Rh(III) conditions:<br>Substrate(0.20 mmol), aldehyde (0.40mmol), [Cp*$RhCl_2$]$_2$ (5/10 mol % of Rh dimer), and $AgSbF_6$ (20/ 40 mol %) in tetrahydrofuran (0.3 M) at 90 °C for 24 h<br>12 examples, 41-76 % yield<br>R$^1$: Ph, tolyl, cyclized alkyl; R$^2$: Alkyl, Ph, Ph-halogenide, PhMe, PhCOOMe, $PhCF_3$; R$^4$: Aryl, alkyl | 430 |

| | | | | | | |
|---|---|---|---|---|---|---|
| 1 | 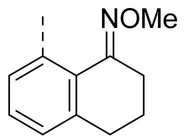<br>or<br>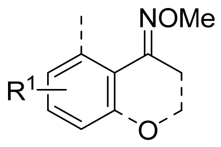 | Acylation | 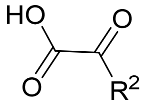 | 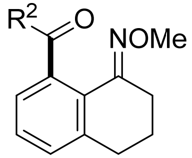<br>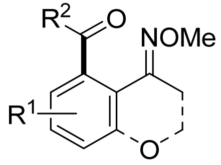 | Substrate (0.3 mmol), α-keto acid (0.45 mmol), $Pd(OAc)_2$ (10 mol%), $(NH_4)_2S_2O_8$ (0.45 mmol), diglyme (1 mL),<br>70 °C in sealed tubes (3 h -10 h)<br>24 examples, 36- 85 % yield<br>$R^1$: Halogenide, MeO, $CF_3$<br>$R^2$ (if $R^1$ present): Ph<br>$R^2$: Ph-halogenide, PhMeO, $PhCF_3$, naphtyl, thiophene | 431 |
| 2 | 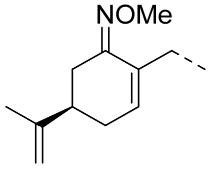 | Amidation | 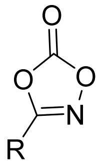 | 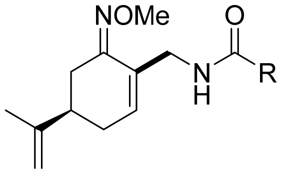 | Substrate (0.2 mmol), dioxazolone (0.21 mmol),<br>AgOAc (8 mol%), $[RhCp^*Cl_2]_2$ (4mol%), $AgSbF_6$ (16 mol%), DCM<br>(3.0 mL), 25 °C, 12 h, sealed tube under nitrogen.<br>11 examples, 21-86 % yield<br>R: Ph, PhMe, Ph-halogenide, $PhCF_3$, Ph-*t*-Bu, thiophen, amide | 419 |

**Ketoxim esters**

Ketoxim esters of various types have been frequently applied in the synthesis of heterocycles such as isoquinolines and pyridines and their oxidized derivatives. The dominant catalyst in this chemistry is $[Cp^*RhCl_2]_2$. The Matsunaga group demonstrated a protocol for the synthesis of multisubstituted isoquinolines by site-selective C-H activation of various unsymmetrically substituted ketoxime esters with terminal and internal alkynes. Notably, this procedure is $Cp^*Co^{III}$ catalyzed and thereby another example for non precious metal catalysis in C-H activation (Table 18, Entry 3).[437] With regard to exceptional metal catalysts in the field, it is important to mention the copper catalyzed coupling of ketoxime esters with sodium sulfinates for the synthesis of sulfone derivatives developed by Tang et al. (Table 18, Entry 4).[438] As already mentioned, ketoxime esters are furthermore utilized for the synthesis of pyridines via C-H activation. In 2013 the Rovis group displays a rhodium catalyzed regioselective pyridine synthesis, starting from alkenes and *α,β*-unsaturated oxime esters. The use of an *O*-pivaloyl ketoxime ester is obligatory since the respective *O*-acetyl ketoxime ester leads to the formation isoxazole instead of the desired pyridine (Table 19, Entry 3).[439]

**Table 18: KetoximEster**

| Entry | Directing group | Type of transformation | Coupling partner | Typical product structure | Comments | Ref |
|---|---|---|---|---|---|---|
| 1 | 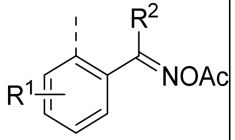 | Cyclization | 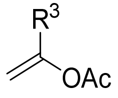 | 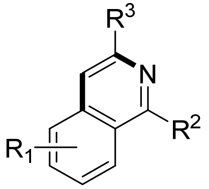 | Substrate (0.4 mmol), vinyl acetate (1.2 mmol), $[Cp^{*}RhCl_2]_2$ (4 mol%), $AgBF_4$ (16 mol%), MeOH (2 mL), 100 °C, 12 h<br>20 examples, 33-87 % yield<br>$R^1$: Me, MeO, alkyl, Ph, Ph-halogenide, $PhCF_3$, $PHNO_2$,<br>$R^2$: Me, alkyl, Ph<br>$R^3$: H, Me, Ph, PhMe, PhCl, thiophene | 440 |
| 2 | | Cyclization | 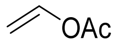 | | Substrate (0.4 mmol), vinyl acetate (1.2 mmol), $[Cp^{*}RhCl_2]_2$ (4 mol%), AgOAc (16 mol%), MeOH (2 mL), 100 °C, 12 h<br>12 examples, 40-90 % yield<br>$R^1$: Me, MeO, alkyl, Ph, Ph-halogenide, $PhCF_3$, $PHNO_2$,<br>$R^2$: Me, alkyl, Ph | 440 |
| 3 | 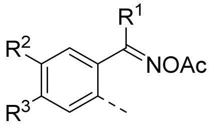 | Cyclization | 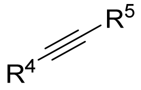 | 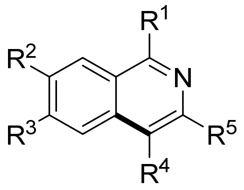 | Substrate (0.15 mmol), alkyne (0.18 mmol), $[Cp^{*}Co(CO)I_2]$ (10 mol%), $AgSbF_6$ (20 mol%), and KOAc (20 mol%) in $ClCH_2CH_2Cl$, 80-120 °C, 24 h, internal and terminal alkynes tolerated<br>56 examples, 45-97 % yield<br>$R^1$: Aryl, alkyl; $R^2$: Alkyl, aryl, halogenide; $R^3$: Alkyl, aryl, halogenide, MeO, $CF_3$; $R^4$: H, aryl, alkyl, ferrocen, pentathrenyl, thienyl; $R^5$: Aryl, alkyl | 437 |

| | | | | | | |
|---|---|---|---|---|---|---|
| 4 | 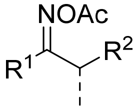 | Sulfonylation | $R^3SO_2Na$ | 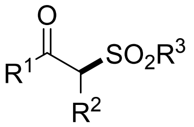 | Substrate (0.5 mmol), sodium sulfinate (0.5 mmol), $Cu(OAc)_2$ (10 mol%), in toluene (2 mL) at 100 °C under $N_2$ stirring in DCM for 6 h, coupling with sodium sulfinates, subsequent hydrolysis; (removal of the DG)<br>30 examples, 70-96 % yield<br>$R^1$: Aryl, thiophene; $R^2$: H; $R^3$: Aryl, alkyl, naphtyl | 438 |

**Table 19: Oxime Ester (Piv)**

| Entry | Directing group | Type of transformation | Coupling partner | Typical product structure | Comments | Ref |
|---|---|---|---|---|---|---|
| 1 | 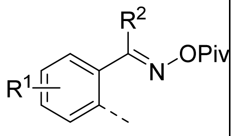 | Cyclization | 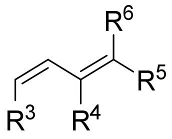 | 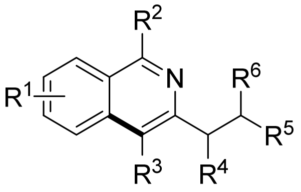 | Substrate (0.2 mmol), dienoate (0.25 mmol), $[Cp^*RhCl_2]_2$ (2.5 mol%), $AgSbF_6$ (15 mol%), PivOH (3.0 equiv.), aromatic o-pivaloylketoxime (0.2 mmol), and (E)-ethyl penta-2,4-dienoate(0.25 mmol) in DCE (1 mL) for 20 h at 100 °C under argon<br>27 examples, 44-91 % yield<br>$R^1$: Me, MeO, $CF_3$, halogenide, $NO_2$, CN; $R^2$: Alkyl; $R^3$: H, alkyl; $R^4$: H<br>$R^{5/6}$: ester, cyanide, aryl, alkyl, ketone, SOOPh, $PO(OEt)_2$ | 441 |
| 2 | | Cyclization | 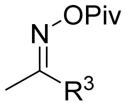 | 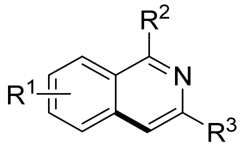 | Substrate (0.125 mmol), aryloxime pivalate (0.375 mmol), 0.05 mmol of $Pd(OAc)_2$, 6 mL of toluene in a sealed tube at 150 °C for 24/48h; selfcoupling (2 eq. oxime ester)<br>34 examples, 20-88 % yield<br>$R^1$: Me, MeO, ester, $CF_3$, halogenide; $R^2$: alkyl, aryl<br>$R^3$: aryl, vinyl, alkoxycarbonyl | 442 |

| | | | | | | |
|---|---|---|---|---|---|---|
| 3 | 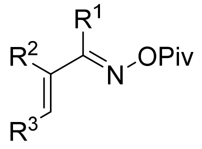 | Cyclization | 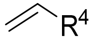 | 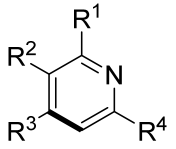 | Substrate (0.21 mmol), alkene (0.25 mmol)$[RhCp^*Cl_2]_2$ (0.005 mmol), and AgOAc (0.44 mmol) in 0.7 mL of 2:1 DCE/AcOH for 14 h.<br>26 examples, 33-96 % yield<br>$R^1$: alkyl; $R^2$: alkyl, aryl; $R^3$: H, alkyl<br>$R^4$; COOEt, Ph, Ph-halogenide, ketone, ester, amide | 439 |
| 4 | 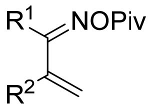 | Cyclization | 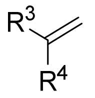 | 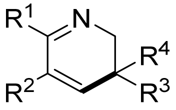 | Substrate ( 1eq), alkene (1.2 eq), Rh (III) catalyst, (cationic tris(acetonitrile) Rh(III) pre-catalyst bearing a trifluoromethyl-substituted Cp* ligand) $[RhCpCF_3^*(CH_3CN)_3](SbF_6)_2$ (2 mol %), CsOAc (2 eq.), HFIP, 50 °C<br>31 examples, 67- 88 % yield<br>$R^1$: alkyl; $R^2$: alkyl, aryl; $R^3$: alkyl; $R^4$: alkyl, ester | 443 |

## Ketoxime

Concerning the ketoxime scaffold as directing group, again rhodium catalyzed processes are dominant. One was published in 2009 by Parthasarathy et al. The procedure describes a highly regioselective synthesis of isoquinoline derivatives from ketoximes and alkynes (Table 20, Entry 1).[444] Thus, a cobalt(III) catalyzed protocol for the production of isochinolins was demonstrated by Sen et al.In this case even oxime containing heterocycles are tolerated (Table 20, Entry 2).[445] Moreover, the Glorius group developed a synthesis of multisubstituted isoquinolines and pyridine *N*-oxides via aryl and vinylic C-H activation (Table 20, Entry 4).[446] Ketoximes can be used as directing groups in the desymmetrization of diazabicycles and thereby provide access to functionalized cyclopentenes (Table 20, Entry 3.[447] The Ackermann group reported the first annulation of redox-active ferrocenylalkynes via catalyzed direct C-H/N-O bond functionalization using a ketoxime as directing group (Table 20, Entry 5).[448] The Jiang group found that the N–OH group of the oximes could serve as a directing group and/or an internal oxidant under different conditions. They demonstrated a palladium catalyzed $C(sp^2)$-H carbonylation of aromatic oximes and thus the access to benzooxazinones and 3-methyleneisoindolin-1-ones (Table 20, Entry 9).[449] Further, a palladium catalyzed direct *ortho* functionalization of aromatic alcohols masked by acetone oxime ethers was developed. Guo et al. achieved as first group a selective alkenylation of aromatic alcohols via a six- or seven-membered *exo*-acetone oxime ether palladacycle (Table 20, Entry 16 & 17).[450] In contrast, a rhodium catalyst would not serve the purpose in this mechanism.

**Table 20: Ketoxime- based directing groups**

| Entry | Directing group | Type of transformation | Coupling partner | Typical product structure | Comments | Ref |
|---|---|---|---|---|---|---|
| 1 | 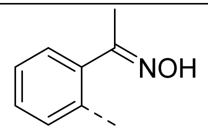 | Cyclization | 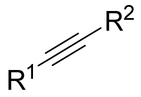 | 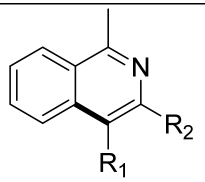 | Substrate (1 mmol), alkyne (1.1 mmol), $Rh(PPh_3)_3Cl$ (3 mol%),<br>130 °C, toluene, 12 h, heterocycles tolerated<br>18 examples, 45-89 %<br>$R^1$, $R^2$: H, alkyl, aryl | 444 |
| 2 | | | 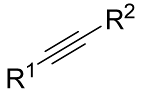 | 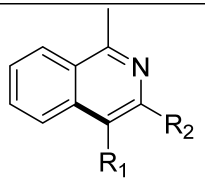 | Substrate (0.2 mmol), alkyne (0.24 mmol), $[Cp^*Co(CO)I_2]$ 10 mol%, NaOAc 20 mol%, $CF_3CH_2OH$, heterocycles tolerated<br>42 examples, 11- 90 % yield<br>$R^1$: Aryl, alkyl<br>$R^2$: Aryl, alkyl | 445 |
| 3 | 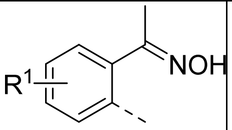 | Desymmetrizatio n | 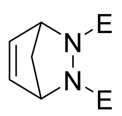 | 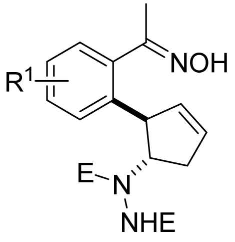 | Substrate (0.2 mmol), diazabicycle (0.22 eq), $[Cp^*RhCl_2]_2$ (2 mol %), AgOAc (8 mol%), MeOH (1.5 mL), 60 °C,<br>6 h, heterocycles tolerated<br>23 examples, 73-95 % yield<br>$R^1$: Alkyl, aryl, OH, ketone, halogenide, $NO_2$, AcHN<br>E: $CO_2Et$, $CO_2tBu$ | 447 |
| 4 | 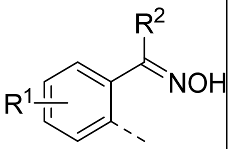 | Cyclization | 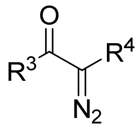 | 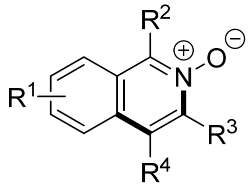 | Substrate (0.2 mmol), diazo compound (0.24 mmol), $[Cp^*RhCl_2]_2$ (2.5 mol%), $AgSbF_6$ (10.0 mol %), MeOH (1.0 mL), 60°C, 12 h, under Ar<br>25 examples, 45-99 % yield<br>$R^1$: H, alkyl; $R^2$: Halogenide, OMe; $R^3$: H, alkyl, aryl | 446 |

| | | | | | | |
|---|---|---|---|---|---|---|
| | | | | | $R^4$: Ester, aryl, $PO(OMe)_2$, $SO_2PhMe$, ketone | |
| 5 | | Cyclization | 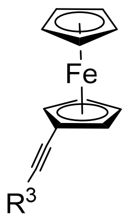 | 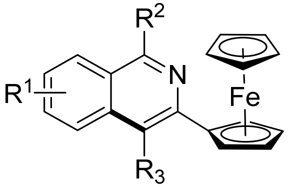 | Substrate (0.5 mmol), alkyne (1 mmol), $[RuCl_2($*p-cymene*$)]_2$ (5 mol%), MeOH, 80 °C, 24 h<br>13 examples, 55-95 % yield<br>$R^1$: Alkyl, OMe<br>$R^2$: Alkyl<br>$R^3$: Alkyl | 448 |
| 6 | | | 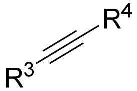 | 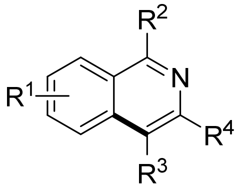 | Substrate (1.0 mmol), alkyne (1.3 mmol), $[RhCp*Cl_2$ (1 mmol%), CsOAc (30 mmol%), methanol (4 mL), 60 °C, 12 h<br>15 examples, 20-95 % yield<br>$R^1$: Halogenide, Me, MeO, naphtyl<br>$R^2$: H, Me, Ph; $R^3$, $R^4$: Alkyl, aryl, MeOH | 451 |
| 7 | 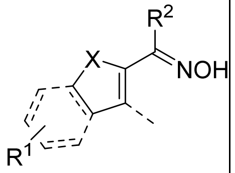 | Cyclization | 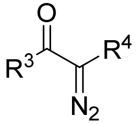 | 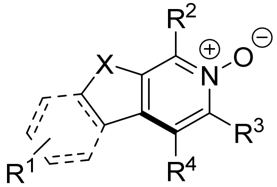 | Substrate (0.5 mmol), diazo compound (1 mmol), $(Cp*RhCl_2)_2$ (2.5 mol%), NaOAc (2 eq), MeOH (2.0 mL) at 80 °C for 12 h under air atmosphere.<br>27 examples, 77-98 % yield<br>X: O, S, N<br>$R^1$: Alkyl, OMe, aryl, halogenide; $R^2$: Alkyl, aryl<br>$R^3$: H, Me, cyclized alkyl; $R^4$: Ester, ketone | 452 |
| 8 | 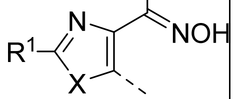 | Cyclization | 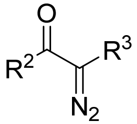 | 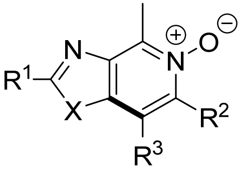 | Substrate (0.5 mmol), diazo compound (1 mmol), $(Cp*RhCl_2)_2$ (2.5 mol%), NaOAc (2 eq), MeOH (2.0 mL) at 70 °C for 12 h under air atmosphere.<br>15 examples, 50-98 % yield<br>X: O, S; $R^1$: Aryl; $R^2$: H, alkyl; $R^3$: Ester | 452 |

| | | | | | | |
|---|---|---|---|---|---|---|
| 9 | 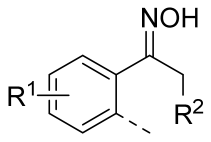 | Carbonylation | CO | 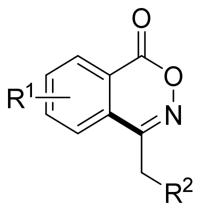 | Substrate (0.5mmol), $PdCl_2$ (0.05mmol), AgOAc (1.0 mmol), CO (balloon), $C_3H_7COOH/(C_3H_7CO)_2O$(2 mL, v/v= 20: 1), 100 °C<br>22 examples, 46-89 % yield<br>$R^1$: alkyl, $CF_3$, OMe, condensed (hetero)cycles<br>$R^2$: H, alkyl | 449 |
| 10 | | | | 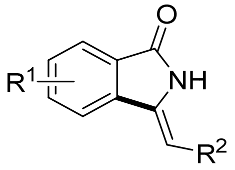 | Substrate (0.5 mmol), $PdCl_2$ (0.05 mmol), $K_2CO_3$ (0.25 mmol), CO balloon, n-$C_3H_7COOH$ (2 mL), 120 °C<br>12 examples, 62-89 % yield<br>$R^1$: Alkyl, OMe, TsO, F, $CF_3$; $R^2$: Alkyl | 449 |
| 11 | 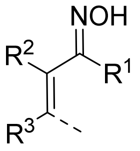 | Cyclization | 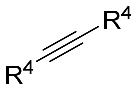 | 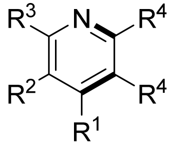 | Substrate (1 mmol), alkyne (1.1 mmol), $Rh(PPh_3)_3Cl$ (3 mol%), and toluene 130 °C, 3h<br>12 examples, 51- 94 % yield<br>$R^1$: H, alkyl; $R^2$: Aryl, alkyl, thiophene<br>$R^3$: H, alkyl, thiophene, aryl; $R^4$: Aryl, alkyl | 453 |
| 12 | 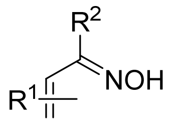 | Cyclization | 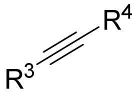 | 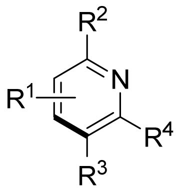 | Substrate, (0.2 mmol), alkyne (0.22 mmol), $[Cp^*RhCl_2]_2$(1.25 mol %) (or $[Cp^tRhCl_2]_2$) $K_2CO_3$ (0.4 mmol) in TFE, 45 °C, 16 h<br>26 examples, 45- 95 %yield<br>$R^1$: Aryl, alkyl, $CF_3$, amide; $R^2$: H, alkyl<br>$R^3$: Alkyl, aryl, ester, $CH_2OTBS$; $R^4$: Alkyl, (hetero)aryl | 454 |
| 13 | 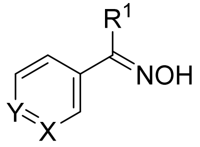 | Cyclization | 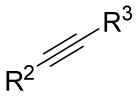 | 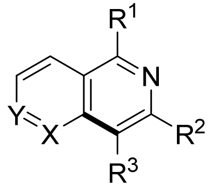 | Substrate (0.2 mmol), alkyne (0.22 mmol), $[Cp^*RhCl_2]_2$(1.25 mol %) (or $[Cp^tRhCl_2]_2$) $K_2CO_3$ (0.4 mmol) in TFE, 45 °C, 16 h<br>13 examples, 70- 96 %yield<br>X/Y: C, S, N, O | 454 |

| | | | | | | |
|---|---|---|---|---|---|---|
| | | | | | $R^1$: Aryl, alkyl<br>$R^2$: Ph<br>$R^3$: Alkyl | |
| 14 | 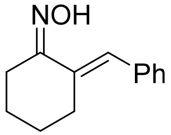 | Cyclization | 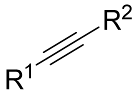 | 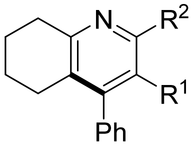 | Substrate (1 mmol), alkyne (1.1 mmol), $Rh(PPh_3)_3Cl$ (3 mol%),<br>130 °C, toluene, 12 h<br>3 examples, 41-83 % yield<br>$R^1$, $R^2$: H, alkyl, aryl | 444 |
| 15 | 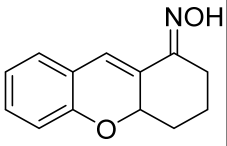 | Cyclization | 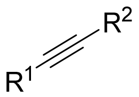 | 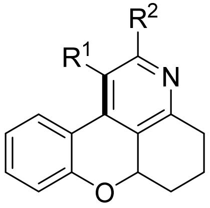 | Substrate (1 mmol), alkyne (1.1 mmol), $Rh(PPh_3)_3Cl$ (3 mol%),<br>130 °C, toluene, 12 h<br>3 examples, 70-76 % yield<br>$R^1$, $R^2$: Alkyl | 444 |
| 16 | 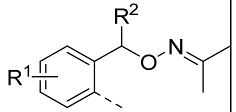 | Alkenylation | 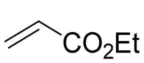 | 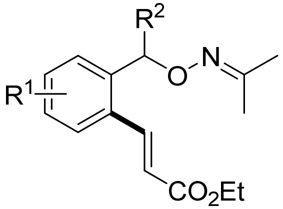 | Substrate (0.2 mmol), alkene (0.3 mmol), $Pd(OAc)_2$<br>(5 mol%), Ac-Val-OH (10 mol%), AgOAc (0.5 mmol), 1,4-dioxane (1 mL), 90 °C, 12 h<br>31 examples, 15 -95 % yield<br>$R^1$: Alkyl, MeS, MeO, halogenide, $NO_2$<br>$R^2$: H, alkyl | 450 |
| 17 | 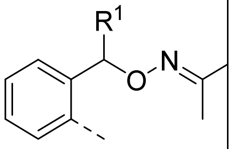 | Alkenylation | 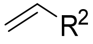 | 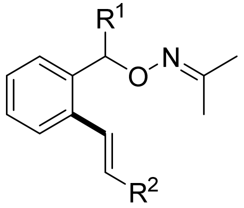 | Substrate (0.2 mmol), alkene (0.3 mmol), $Pd(OAc)_2$<br>(5 mol%), Ac-Val-OH (10 mol %), AgOAc (0.5 mmol), 1,4-dioxane (1 mL), 90 °C, 12 h<br>40 examples, 50 -77 % yield<br>$R^1$: Alkyl<br>$R^2$: H, alcohol, carbonyl, $SO_2Ph$ | 450 |

## Aldoximes and aldoxime ether

In the case of aldoximes only few examples have been reported. The lower stability of the substrates is for sure an important reason for this fact. Still, arylation, alkenylations and even nitration has been reported. Typically, an acidic medium is required since basic conditions would lead to quick hydrolysis of the aldoxime DG, especially at the high temperatures usually required for C-H activation.

**Table 21: Aldoxime- based directing groups**

| Entry | Directing group | Type of transformation | Coupling partner | Typical product structure | Comments | Ref |
|---|---|---|---|---|---|---|
| 1 | 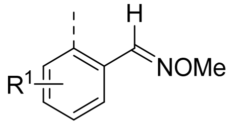<br> | Alkenylation | 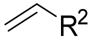<br> | 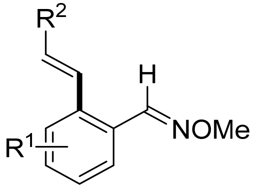<br> | Substrate (0.5 mmol), alkene (1.0 mmol), BQ (0.5 mmol), $Pd(OAc)_2$ (2.24 mg, 0.01 mmol, 2 mol%), and acetic acid (2 mL) were added in a 25 mL sealed tube with a teflon lined cap. The mixture was heated at 80 °C for 6 h.<br>21 examples, 51-93 % yield<br>$R^1$: Alkyl, halogenide, OMe, OH<br>$R^2$: Ester | 455 |
| 2 | | Arylation | 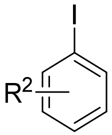<br> | 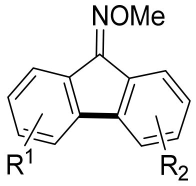<br> | Substrate (1.0 mmol), aryl iodide 2 (5-6 equiv), $Pd(OAc)_2$ (1.0 mmol) and $CF_3CO_2H$ (10 mol%), $Ag_2O$ (2.0 mL), 120 °C, 36 h<br>18 examples, 63-90 % yield<br>$R^1$: Me, halogenide<br>$R^2$: Me, $NO_2$, ester, OMe | 456 |
| 3 | | | 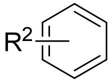<br> | | Substrate 80.7 mmol), arene (2 ml), $Pd(OAc)_2$ (20 mol %), $K_2S_2O_8$ (1.4 mmol), TFA (7.0 mmol), 120 °C, 15h<br>22 examples, 51-91 % yield<br>$R^1$: Me, halogenide<br>$R^2$: Me, $NO_2$, ester, OMe | 457 |

| | | | | | | |
|---|---|---|---|---|---|---|
| 4 | | Nitration | $AgNO_2$ | 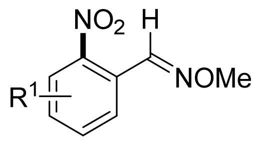 | Substrate (0.3 mmol), $Pd(OCOCF_3)_2$ (0.03 mmol),$AgNO_2$ (0.6 mmol), $K_2S_2O_8$ (0.6 mmol), DCE (3.0 mL), 110 °C, 48 h<br>24 examples, 38- 93 % yield<br>$R^1$: Halogenide, $CF_3$, alkyl, aryl, OMe, naphtyl | [458] |

## Phosphorous-containing directing groups

P-containing directing groups have been added relatively late to the toolbox of C-H activation chemistry. Typically, it is not P-which coordinates to a metal catalyst, but a heteroatom attached to it, most importantly oxygen and to a lesser extent nitrogen. Within the last three years, a series of examples have been disclosed, which demonstrate the high synthetic potential of P-containing functional groups as directing groups in C-H activation chemistry. Phosphinic acid, phosphonic acids, and phosphoric acids as well as the respective ester derivatives have been used so far. Additionally, simple phosphine oxides, phosphonamides, phosphoramidic acids and phosphinic amides have been applied.

Several examples have been disclosed in which the directing group is additionally reacting with a coupling partner in cyclization reactions to give mainly oxaphosphinanes or oxaphospholanes and to a lesser extent azaphosphinanes and azaphospholidines as well as phosphoindoles. The main contributor in this field of cyclizations towards P-containing heterocycles was the group of Lee. They demonstrated the application of phosphor containing directing groups for the synthesis of phosphorous heterocycles under aerobic conditions under ruthenium and rhodium catalysis in an intermolecular fashion using either alkynes or alkenes as coupling partners.[459-462]Additionally, they reported intramolecular cyclization reactions. Interestingly, in these cases, simple $Pd(OAc)_2$ could be applied as the catalyst.[463, 464] The required oxidant for these transformations had to be optimized for each individual case.

The Han group was the first, who employed a phenylphosphinic acid in the sense of a directing group in C-H activation. A new oxapalladacycle was conveniently prepared via direct *ortho* palladation of diphenylphosphinic acid with palladium acetate.[465] In 2014 Zhang et al. demonstrated a novel and efficient Pd-catalyzed C–H acetoxylation, which uses $R_2(O)P$ as a directing group to synthesize various substituted phosphorylbiphenyl-2-OAc compounds (Table 22, Entry 18).[466] The Kim group has focused on the application of organophosphates as directing group in C-H activation. For instance, a palladium(II) catalyzed *ortho*-arylation of aryl phosphates and aryl hydrogen phosphates were demonstrated in 2013 (Table 23, Entry 4).[467] Further, a procedure for Pd-catalyzed acetoxylation ofbenzyl phosphonic and aryl phosphoric monoacids, providing access to various acetoxy benzylic phosphonic acids along with catechol derivatives (Table 22, Entry 12).[468]In contrast to that, the Duan group demonstrated an Ag-mediated C−H/P−H functionalization of arylphosphine oxides with internal alkynes (Table 23, Entry 9).[469] Chary et al. described first phosphoramidate directing group for synthetically useful arylation. This new directing group drives selective C-H bond activation to afford*N*-aryl phosphoramidates in good to excellent yields at room temperature (Table 22, Entry 5). [470] The synthesis of another nitrogen containing scaffold by application of phosphorus containing directing groups was demonstrated by Park et al. The rhodium-

catalyzed oxidative coupling via C–H activationand annulation directed by phosphonamide and phosphinamide group functions under aerobic conditions an yields benzazaphosphole 1-oxides and phosphaisoquinolin-1-oxides (Table 23, Entry 7).[471] Itoh et al. described a ruthenium catalyzed process: a wide range of tri-, di-, and monoarylphosphine oxides efficiently undergo *ortho*-alkenylation through insertion of alkynes, which is environmentally benign because no oxidant such as stoichiometric silver or copper salts is needed(Table 22, Entry 17).[472] The Lee group demonstrated the application of phosphor containing directing groups for the synthesis of phosphorous heterocycles under aerobic conditions under ruthenium and rhodium catalysis (Table 23, Entry 7) and (Table 22, Entry 1&2).[459, 460, 471]The access to diverse P-containing functional frameworks via rhodium(III)-catalyzed oxidative C-H activation of arylphosphonates and phosphonamides with subsequent coupling with alkenes (olefination), internal alkynes (hydroarylation and oxidative cyclization), or arenes was reported by the Glorius group (Table 22, Entry 13).[473]

**Table 22: Phosphonic acid and derivatives as directing groups**

| Entry | Directing group | Type of transformation | Coupling partner | Typical product structure | Comments | Ref |
|---|---|---|---|---|---|---|
| 1 | 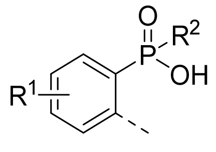 | Cyclization | 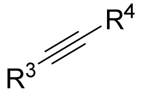 | 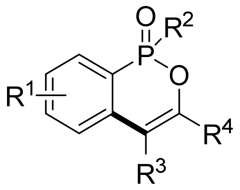 | Substrate (0.2 mmol), alkyne (0.3 mmol), $[RuCl_2(p\text{-cymene})]_2$ (10 mol%), the most common oxidant $Cu(OAc)_2 \cdot H_2O$ for oxidative alkyne annulation reactions was found to be ineffective, and a mixture of silver salts was required as the sacrificial oxidants; $KPF_6$ (20 mol %); $AgCO_3$ (1 eq.); AgOAc(1 eq.); *t*-BuOH; 90°C; under air<br>34 examples, 27- 97 % yield<br>$R^1$:H, alkyl, halogenide, ether, carbonyl, AcMeO, OH, ($OCH_2O$) naphtalenyl, indenyl, thiophenyl, OMe<br>$R^2$: OEt, Ar; $R^3$, $R^4$: Alkyl, aryl | 460 |
| 2 | | | | | Substrate (0.15 mmol), alkyne (0.23 mmol), $[Cp^*RhCl_2]_2$ (2 mol%); $Ag_2CO_3$ (0.15 mmol), AgOAc (0.15 mmol), *t*BuOH (1 mL), 90 °C, under air<br>44 examples, 60-95 % yield<br>$R^1$: Alkyl, halogenide, ether, carbonyl, AcMeO, OH, | 459, 461 |

| | | | | | | |
|---|---|---|---|---|---|---|
| | | | | | $(OCH_2O)$ naphtalenyl, indenyl, thiophenyl, OMe<br>$R^2$: OEt, Ar; $R^3$, $R^4$: Alkyl, aryl | |
| 3 | 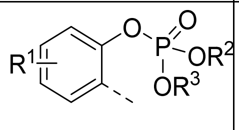 | Arylation | $Ar_2IOTf$ | 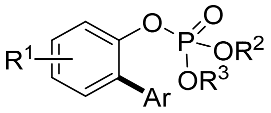 | Substrate (0.15 mmol), $Pd(TFA)_2$ (10 mol%);<br>$Ph_2IOTf$ (2 eq.); 1,2-dichloroethane; 80°C; 15 h<br>12 examples, 29-75 % yield<br>$R^1$: Alkyl, halogenide, OMe<br>$R^2$:H, Me, $CH_2C(CH_3)_2CH_2$, OH; $R^3$: Me | 467<br>474 |
| 4 | 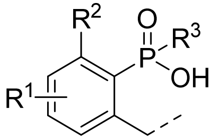 | Cyclization | Intramolecular | 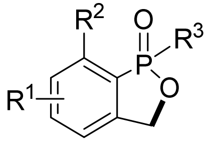 | Substrate (0.2 mmol), $Pd(OAc)_2$ (10 mol%); $(4\text{-MeO-}C_6H_4)_3P$ (0.4 eq.); $Ag_2CO_3$(3 eq.);<br>$K_2HPO_4$ (2.5 eq.); PhCl ; 120°C,12-36 h; phosphinic acid with methylgroup in ortho position<br>16 examples, 43-81 % yield<br>$R^1$: H, Alkyl, aryl , OMe, OPh, halogenide, $CF_3$, TMS<br>$R^2$: Alkyl<br>$R^2$: OMe, OEt, Me, Ph | 463 |
| 5 | 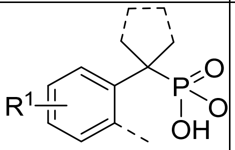 | Cyclization | Intramolecular | 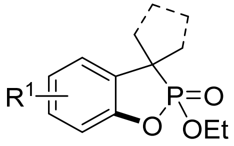 | Substrate (0.2 mmol),$Pd(OAc)_2$ (10 mol%), $PhI(OAc)_2$ (0.3 mmol), NaOAc (0.2 mmol),<br>80 °C, 20 h, DCE (2 mL)<br>20 examples, 55-76 % yield<br>$R^1$: Alkyl, aryl , OMe, halogenide, $CF_3$ | 463 |
| 6 | 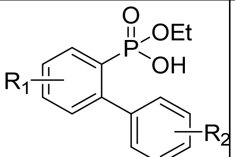 | Cyclization | Intramolecular | 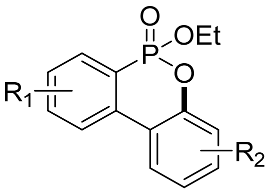 | Substrate (1 eq), $PhI(OAc)_2$ (2.0 equiv.), $Pd(OAc)_2$ (10mol%), KOAc (2 equiv.), *t*BuOH, 30 mol% *N*-acetyl-L-Leucin, 12 h, air atmosphere, 100 °C<br>20 examples, 50-72 % yield<br>$R^1$, $R^2$: Me, OMe, halogenide, naphtyl | 464 |

| | | | | | | |
|---|---|---|---|---|---|---|
| 7 | 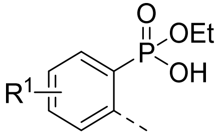 | Cyclization | 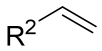 | 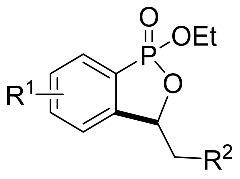 | Substrate (0.2 mmol), alkene (2 eq), $[Cp^*RhCl_2]_2$ (4 mol%); AgOAc (2 eq.), $Na_2HPO_4$ (1 eq.), and $CH_3CN$; 110°C; 16 h<br>16 examples, 61-90 % yield<br>$R^1$: Me<br>$R^2$: CO-alkyl, CN, $CONMe_2$, $SO_2PH$, $PO(OMe)_2$ | 462 |
| 8 | | Cyclization | 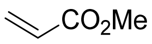 | 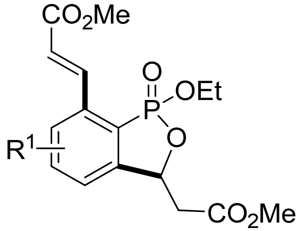 | Substrate (0.2 mmol), alkene (2 eq), $[Cp^*RhCl_2]_2$ (4 mol%); AgOAc (2 eq.), $Na_2HPO_4$ (1 eq.), and $CH_3CN$; 110°C; 24 h<br>8 examples, 59-76 % yield<br>$R^1$: Alkyl, aryl, halogenide, OMe, OAc | |
| 9 | 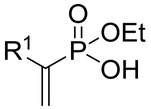 | Cyclization | 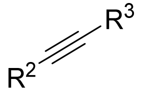 | 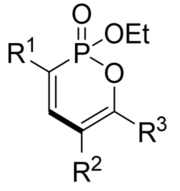 | Substrate (0.15 mmol), alkyne (0.15 mmol), $[(Cp^*RhCl_2)_2]$ (2 mol%), $Ag_2CO_3$ (1 equiv), DMF (1 mL), 120 °C, 10 h under $N_2$.<br>11 examples, 75-91 % yield<br>$R^1$, $R^2$, $R^3$: Aryl, alkyl | 459 |
| 10 | 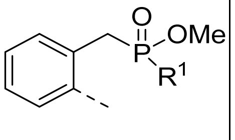 | Alkenylation | 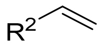 | 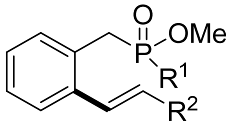 | Substrate (1 eq), alkene (2 eq),<br>1.) $Pd(OAc)_2$ (10 mol%), AgOAc (3 eqiv.), dioxane, 110 °C, 24 h<br>2.) TMS-$CHN_2$, $CH_3OH$, 0.5 h, rt<br>27 examples, 55-96 % yield<br>$R^1$:OMe, OH<br>$R^2$: $CO_2Et$, aryl, alkyl, carbonyl, ester | 475 |
| 11 | 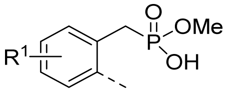 | Arylation | $ArBF_3K$ | 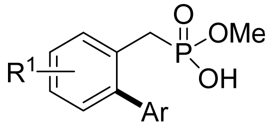 | Substrate (1 eq), $PhBF_3K$ (3 eq), $PdCl_2(PEt_3)_2$ (10 mol %), Ac -Val-OH (20 mol%), $Ag_2O$ (2 equiv), and $KHF_2$ (1 equiv) in *t*-BuOH at 110 °C for 24 h<br>27 examples, 18-95 % yield | 476 |

| | | | | | | |
|---|---|---|---|---|---|---|
| | | | | | $R^1$: Alkyl, naphtyl, $CF_3$, halogenide, OMe | |
| 12 | 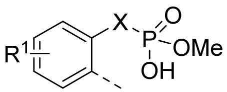 | Acetoxylation | $PhI(OAc)_2$ | 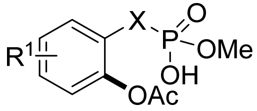 | Substrate (0.15 mmol),<br>1.) 2-3 equiv of $PhI(OAc)_2$, 5 mol% $Pd(OAc)_2$ in 1 mL of 1,2-dichloroethane for 15 h at 110 °C.<br>2.) 5 equiv of $TMSCHN_2$ in 0.5 mL of MeOH at rt for 30 min<br>27 examples, 53-95 % yield<br>X: $CH_2$, O<br>$R^1$: Alkyl,, halogenide, OMe, $CF_3$ | 468 |
| 13 | 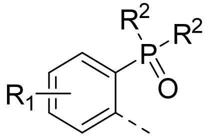 | Arylation | 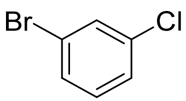 | 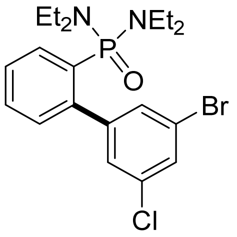 | Substrate (1 eq), arene (40 eq), 2.5 mol% $[RhCp^*Cl_2]_2$, 10 mol% $AgSbF_6$, 2.2 eq $Cu(OAc)_2$, 1 eq PivOH, 20 mol% CsOPiv, 160 °C, 24h<br>1 example, 41 % yield | 473 |
| 14 | | Cyclization | 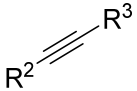 | 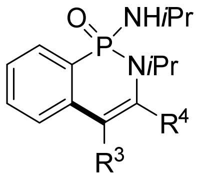 | $RhCp^*(CH_3CN)_3(SbF_6)_2$, 2 eq$Cu(OAc)_2$, DCE, 130 °C, 24 h<br>5 examples,71-77 % yield<br>$R^1$: H<br>$R^2$: OEt, $NEt_2$, NH*i*Pr<br>$R^3$, $R^4$: aryl, alkyl | |
| 15 | | Heck reaction | 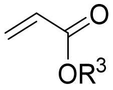 | 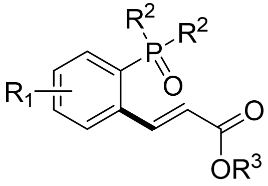 | $RhCp^*(CH_3CN)_3(SbF_6)_2$, $Cu(OAc)_2$, DCE, under air, 130 °C, 24 h<br>9 examples, 77-94 % yield<br>$R^1$: Me, halogenide, OMe, naphtyl; $R^2$: OEt, $NEt_2$<br>$R^3$: $O^nBu$, OEt | |

| | | | | | | |
|---|---|---|---|---|---|---|
| 16 | | Hydroarylation | 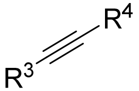 | 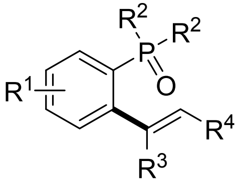 | 2.5 mol% $[RhCp^*Cl_2]_2$, 10 mol % $AgSbF_6$, 10 mol % $Cu(OAc)_2$,<br>1 eq. PivOH, DCE, 110 °C, 24 h<br>6 examples, 72-83 % yield<br>$R^1$: Me, naphtyl; $R^2$: OEt, $NEt_2$; $R^3$, $R^4$: Aryl | |
| 17 | 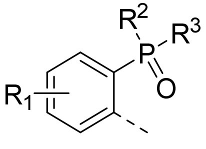 | Alkenylation | 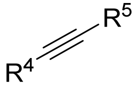 | 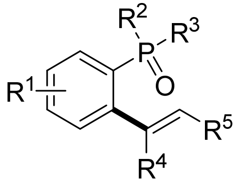 | Substrate (1.25 mmol), alkyne (0.25 mmol), $[RuCl_2(p\text{-cymene})]_2$ (5 mol%); $AgSbF_6$ (20 mol%); AcOH (4 eq.), dioxane; 100 °C; substituted styrene derivatives were obtained in a regio- and stereoselective fashion upon treatment of tri-, di-, or monoarylphosphine oxides with internal alkynes.<br>22 examples, 20-98 % yield<br>$R^1$: Me, OMe, halogenide; $R^2$, $R^3$,: Aryl<br>$R^4$, $R^5$: Aryl, alkyl, thiophenyl | 472 |
| 18 | 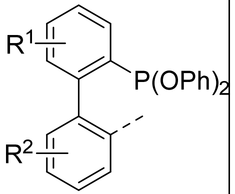 | Acetoxylation | $PhI(OAc)_2$ | 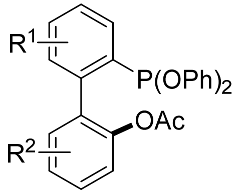 | Substrate (0.2 mmol), $PhI(OAc)_2$ (3.0 equiv.), $Pd(OAc)_2$ (10mol%),$CF_3CH_2OH$ (2.0mL), air atmosphere, 100 °C<br>20 examples, 25-79 % yield<br>$R^1$: Me, $CF_3$; $R^2$, $R^3$: Me, OAc, ester, halogenide | 466 |

**Table 23: Phosphates and derivatives as directing groups**

| Entry | Directing group | Type of transformation | Coupling partner | Typical product structure | Comments | Ref |
|---|---|---|---|---|---|---|

| | | | | | | |
|---|---|---|---|---|---|---|
| 1 | 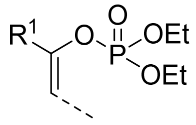 | Alkenylation | 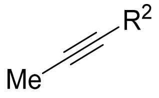 | 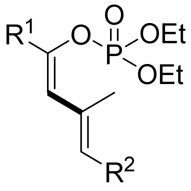 | Substrate (0.15 mmol), alkyne (0.15 mmol), [(Cp*$RhCl_2$)$_2$] (2.5 mol%), $AgSbF_6$ (10 mol%) and $Cu(OAc)_2 \cdot H_2O$ (20 mol %), PivOH (1.1 eqiv.), in THF at 40 °C for 17 h.<br>5 examples, 34-83 % yield<br>$R^1$: Aryl<br>$R^2$: Aryl, ester, ketone | 477 |
| 2 | | **A)**Alkenylation<br>**B)**Hydroalkeny-lation | 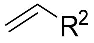 | 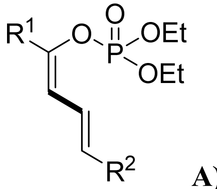 **A)/B)**<br>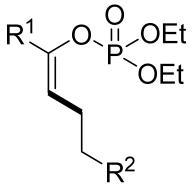 | **A)** Substrate (0.15 mmol), alkene (2 eq), [(Cp*$RhCl_2$)$_2$] (2.5 mol%), $AgSbF_6$ (10 mol%) and $Cu(OAc)_2 \cdot H_2O$ (1.1 eqiv.), in THF at 80 °C for 17 h.<br>41 examples, 20-90 % yield<br>**B)** Substrate (0.15 mmol), enone (2 eq), [{Cp*$RhCl_2$ (2.5 mol%), $AgSbF_6$ (10 mol%) and $Cu(OAc)_2 \cdot H_2O$ (60 mol %), in THF at 80 °C for 17 h.<br>10 examples, 35-89 % yield<br>$R^1$: Aryl<br>$R^2$: **A)** $CO_2R$ (ester)**, B)** COR (ketone) | |
| 3 | 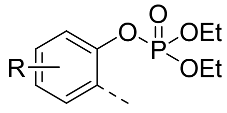 | Arylation | $PhI(OAc)_2$ | 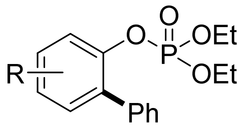 | Substrate (0.25 mmol), diphenyliodonium triflate (0.5 mmol),$Pd(OTf)_2 \cdot 2\ H_2O$ (0.025 mmol), and $Na_2CO_3$ (0.25 mmol) in 1,2-<br>dichloroethane (1.0 mL, 0.25 M) at the designated temperature under Ar for 1 h.<br>20 examples, 27-91 % yield<br>R: OMe, alkyl, halogenide | 474 |

| | | | | | | |
|---|---|---|---|---|---|---|
| 4 | 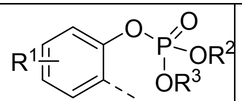 | Arylation | $Ar_2IOTf$ | 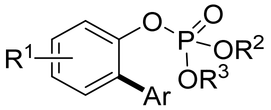 | Substrate (0.15 mmol), $Pd(TFA)_2$ (10 mol%);<br>$Ph_2IOTf$ (2 eq.); 1,2-dichloroethane; 80°C; 15 h<br>12 examples, 29-75 % yield<br>$R^1$: Alkyl, halogenide, OMe<br>$R^2$:H, Me, $CH_2C(CH_3)_2CH_2$, OH<br>$R^3$: Me | 467<br>474 |
| 5 | 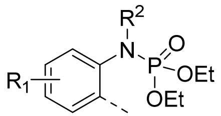 | Arylation | $R^3{}_2IOTf$ | 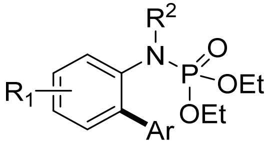 | Substrate (0.3 mmol), $Pd(OAc)_2$ (5 mol%);<br>$Ar_2IOTf$ (1.2 eq.), TfOH, (20 mol %); CuO (3 eq.); 1,4-dioxane; 25 °C<br>24 examples, 68-83 % yield<br>$R^1$: Me, Et, *tert*Bu, Bn, MeO, EtO, PhO, halogenide<br>$R^2$: H, Me, Et, *n*Bu<br>$R^3$: Aryl | 470 |
| 6 | 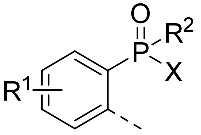 | Arylation | $ArB(OH)_2$ | 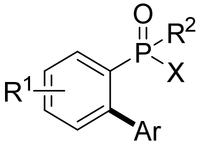 | Substrate (0.25 mmol), boronic acid (0.5 mmol)$Pd(OAc)_2$ (10mol%), BQ (10mol%); CsF (1 eq.); $AgCO_3$ (1.5 eq); DMF; 40°C; 12h; under nitrogen; mixture of mono- and di-orthoarylated product. (BQ plays a critical role in the transmetalation or reductive elimination step. Previous reports have suggested that BQ could improve the reaction through promoting<br>the reductive elimination in C-H activation/C-C bond formations).<br>27 examples, 16-64 % yield<br>X: $NHC_6F_5$, OMe, $NEt_2$, $NHC_3H_7$, N(OMe)Me<br>$R^1$: Alkyl, halogenide<br>$R^2$: Aryl | 478 |

| | | | | | | |
|---|---|---|---|---|---|---|
| 7 | 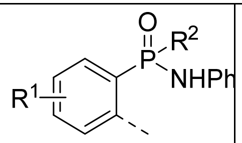 | Cyclization | 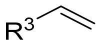 | 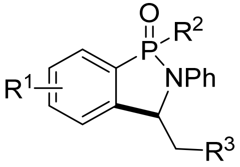 | Substrate (0.2 mmol), alkene (0.4 mmol), $[Cp^*RhCl_2]_2$ (4 mol%), TEMPO (0.5 mmol), CsOPiv (0.15 mmol), xylene (0.8 mL) at 110 °C for 20 h.<br>20 examples, 55-98 % yield<br>$R^1$: Me, OMe, halogenide, $CF_3$, Ac, $NO_2$<br>$R^2$: OEt<br>$R^3$: Ester, CN, $SO_2Ph$ | 471 |
| 8 | | Cyclization | 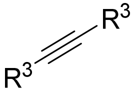 | 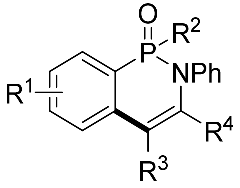 | Substrate (0.15 mmol), alkyne (0.3 mmol), $[Cp^*RhCl_2]_2$ (4 mol%), $AgCO_3$ (0.3 mmol), $KH_2PO_4$ (0.15 mmol), *t*BuOH, 110 °C for 16 h.<br>16 examples, 60-99 % yield<br>$R^1$: Me, OMe, halogenide, $CF_3$<br>$R^2$: Aryl<br>$R^3$: Alkyl, aryl | |
| 9 | 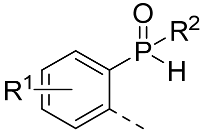 | Cyclization | 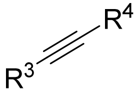 | 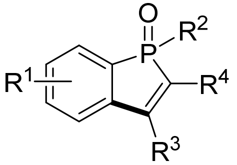 | Substrate (2 eq), alkyne (1 eq), $Ag_2O$ (5 mol% or 2 equiv.), $Zn(NO_3)_2 \cdot 6H_2O$ (1 equiv.), DMF, 100 °C, 12 h<br>17 examples, 22-94 % yield<br>$R^1$: Me, OMe, halogenide<br>$R^2$: Aryl<br>$R^{3,}$ $R^4$: Alkyl, aryl | 469 |

## Si-containing directing groups

Hatanaka et al. demonstrated in 1997 a general approach for the synthesis of functionalized silacycloalkanes, via Rh-catalyzed carbenoid insertion into the $\beta$-C-H bonds of silacycloalkanes (Table 24, Entry 8).[479]

Further, acylsilanes were employed by Becker et al. in a rhodium-catalyzed olefination process for ortho olefinations of aroylsilanes(Table 24, Entry 1).[480] Another palladium catalyzed alkenylation procedure of arenes using silanol as directing group was described by Wang et al. in 2011(Table 24, Entry 2).[481] A cyclization procedure was reported by the Gevorgyan group. In this protocol a Pd-catalyzed benzylsilanol directed *ortho* C-H oxygenation of aromatic rings and further the applicability of silanol as a traceless directing group for Pd-catalyzed *o*-alkenylation of phenols was demonstrated(Table 24, Entry 3).[482] Furthermore an efficient Pd-catalyzed *meta*-directing group based on a silicon tether was developed by Lee et al. The C−H activation was successful for different substitution patterns on the aromatic ring, and the template could be applied to primary and secondary alcohols (Table 24, Entry 4).[483] In general, site-selective C−H functionalization has become an efficient tool regarding the synthesis of complex molecules. Thereby, directing group assisted metallacycle formation serves as an efficient method to ensure promising regioselectivity, as demonstrated by a variety of *ortho*- and *meta*-C−H functionalizations. However, directing group assisted selective *para*-C−H functionalization in arenes has remained uninvestigated, because it includes the formation of a geometrically constrained metallacyclic transition state. Noteworthy, in 2015 Bag et al. reported an easily recyclable, novel Si-containing biphenyl-based template that directs efficient functionalization of the distal *p*-C−H bond of toluene by forming a D-shaped assembly. The complex template morphology enabled a large transition state that favored exquisite site selectivity in performing *para*-olefination and acetoxylation (Table 24, Entry 5).[484]

**Table 24: Si- containing directing groups**

| Entry | Directing group | Type of transformation | Coupling partner | Typical product structure | Comments | Ref |
|---|---|---|---|---|---|---|
| 1 | 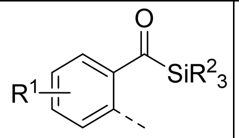 | Alkenylation | 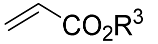 | 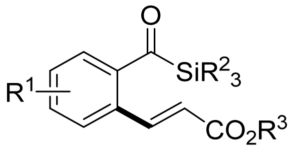 | Substrate (1 eq), acrylat (2 eq), $[(RhCp^*Cl_2)_2]$ (2.5 mol%) AgOTf (10 mol%), $Cu(OAc)_2$ (1.2 equiv) in DCE at 60 °C for 24 h<br>19 examples ,21-90 % yield<br>$R^1$: H, Me, OMe, halogenide<br>$R^2$: Alkyl, aryl<br>$R^3$: Ester | 480 |
| 2 | 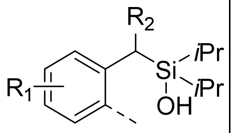 | Alkenylation | 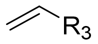 | 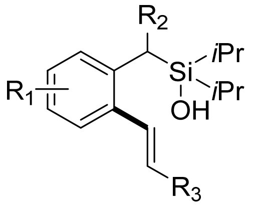 | Substrate(0.3 mmol), alkene (0.6 mmol, 2 equiv), $[Pd(OAc)_2]$ (0.06 mmol, 20 mol%), AgOAc (0.6 mmol, 2 equiv), $KH_2PO_4$ (0.6 mmol, 2 equiv), $CHCl_3$ (3.0 mL), 100 °C, 16 h.<br>25 examples, 36-91 % yield | 481 |

| | | | | | | |
|---|---|---|---|---|---|---|
| | | | | | $R^1$: Me, OMe, halogenide, ester<br>$R^2$: H, Me<br>$R^3$: Ester, ketone, $SO_2Me$, CN, aryl, amide | |
| 3 | 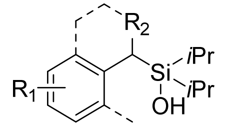 | Oxygenation | $PhI(OAc)_2$ | 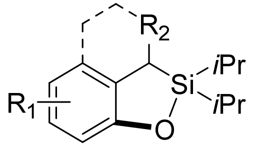 | Substrate (5 mmol), $[Pd(OAc)_2]$ 5 mol%, $PhI(OAc)_2$ (1.2-1.5 equiv), $PhCF_3$ (0.1 M),<br>100°C<br>13 examples, 50-90 % yield<br>$R^1$: Aryl, alkyl, *i*-Pr, naphtyl<br>$R^2$: H, aryl, alkyl | 482 |
| 4 | 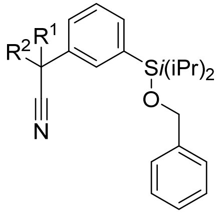 | Alkenylation | 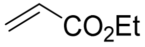 | 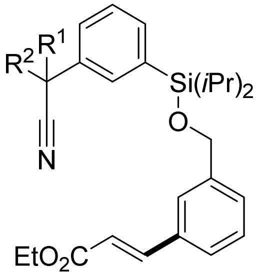 | Substrate (0.1 mmol), 1.5 equivethylacrylate, $Pd(OAc)_2$ (10 mol%),<br>Ac-Gly-OH (20 mol %), AgOAc (2 eq.), DCE, 90 °C, 24 h<br>18 examples, 8-84 % yield<br>$R^1$, $R^2$: Alkyl, *i*-Pr, *c*-Hx | 483 |
| 5 | 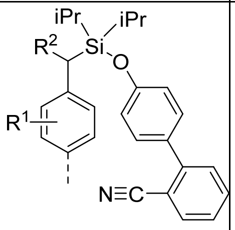 | Acetoxylation | $PhI(OAc)_2$ | 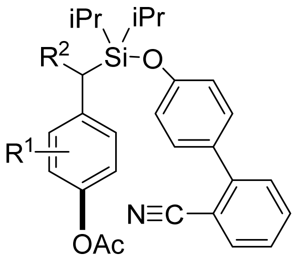 | Substrate (1 eq), $Pd(OAc)_2$ (15 mol%), Piv-Ala-OH (30 mol %), $PhIOAc_2$ (2 eq.); HFIP, 70 °C, 24 h<br>7 examples, 48-68 % yield<br>$R^1$: Me, halogenide<br>$R^2$: H, Me | 484 |

| | | | | | | |
|---|---|---|---|---|---|---|
| 6 | 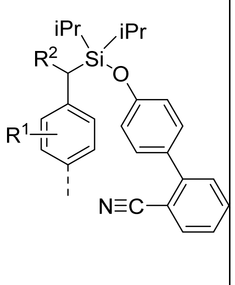 | Alkenylation | 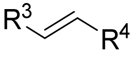 | 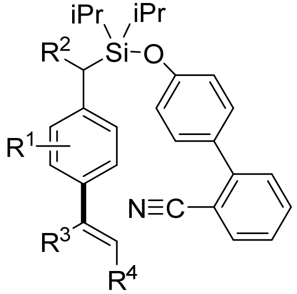 | Substrate (1eq), 2 eq. alkene, $Pd(OAc)_2$ (10 mol%), Ac-Phe-OH (20 mol%), AgOAc (3 eq.); HFIP, 90 °C, 36 h<br>27 examples, 48-76 % yield<br>$R^1$: Me, OMe, halogenide, $CF_3$<br>$R^2$: H, Me, Ph<br>$R^3$, $R^4$: H, ester, aryl | 484 |
| 7 | 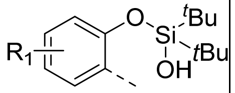 | Alkenylation | 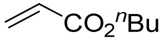 | 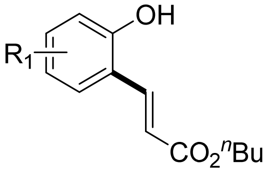 | Substrate (1 eq), alkene (4 equiv), Boc-Val-OH (20 mol %) as the<br>ligand, 110 °C-120 °C, 24 h, 1 eq. $Li_2CO_3$, 4 eq. AgOAc,<br>10 mol% $Pd(OAc)_2$, traceless DG<br>11 examples, 52-97 % yield<br>$R^1$: Me, OMe, halogenide, $OCF_3$, alkyl | 485 |
| 8 | 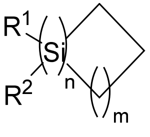<br>n=1,2; m=1,2,3 | Alkylation | 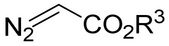 | 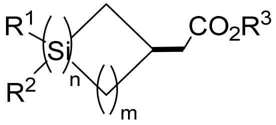<br>n=1,2; m=1,2,3 | Substrate (0.2-0.4 mmol), diazo ester (1.1-4.0 eq), $Rh_2OAc$ (2.5 mol%), DCM, RT<br>7 examples, 62-95 % yield<br>$R^1$, $R^2$: Me,MePh<br>$R^2$: Alkyl | 479 |

## Azo-containing directing groups

In 2013 the Wang group developed a Pd-catalyzed protocol for the synthesis of acylated azobenzenes from aromatic azo compounds and aldehydes via an azo-directed C-H bond activation process and with TBHP as an oxidant (Table 25, Entry 2).[486] Moreover, an unprecedented C−H functionalization ofaryldiazo compounds without a preinstallation of a directinggroup has been performed by Qiu et al. This procedure differs from other reports in its use of diazo compounds as coupling partners in directed C−H activations by application of a rhodium self-relay catalysis. This tandem process includes the in situ formation of a directing groupand a sequential C−H bond activation (Table 25, Entry 1).[487] Song et al. developed a protocol tosynthesize *o*-acylazobenzenes through the Pd(II)-catalyzed C−H bond activation of azobenzenes with toluene derivatives, which are used as acylation reagent in this transformation. Further, diacylazobenzenes were obtained when TBHP loading was increased (Table 25, Entry 5).[488] Moreover, an azo-group directed, highly regioselective synthesis of 2-alkoxy aromatic azo compounds via palladium(II)-catalyzed alkoxylation of azobenzene derivatives using alcohols as the alkoxylation reagents has been demonstrated by the Sum group. This method is applicable to both primary and secondary alcohols(Table 25, Entry 9).[489] In 2015 Zhang et al. and Xia et al. reported a palladium catalyzed direct C−H bond sulfonylation of azobenzenes witharylsulfonyl chlorides (Table 25, Entry 22).[490, 491] The first C–H aminocarbonylation of azoarenes with isocyanates by using rhenium catalysis was developed by Geng et al. This protocol provides a chemo- and regioselective approach to mono-C–H functionalized *o*-azobenzamides (Table 25, Entry 15).[492] The application of a rhodium-catalyst was reported for instance by Wang et al. A regioselective C−N bond formation of azo compounds through C−H bond functionalization using azides as thenitrogen source was developed. Alkyl, aryl, and sulfonyl azides were efficiently assembled in this reaction with excellent functional group tolerance (Table 25, Entry 16).[493] Moreover, a rhodium(III)-catalyzed highly functionalgroup-compatible synthesis of substituted indazoles is reported via C−H bond addition of azobenzenes to aldehydes. The regioselective coupling of unsymmetrical azobenzenes led to the development of a new removable aryl group that enables the preparationof indazoles without *N*-substitution by the Ellman group(Table 25, Entry 20). [494] In 2014, a protocol for the Pd-catalyzed regiospecificortho-nitration of (*E*)-azoarenes has been reported for the first time using $^t$BuONO as a nitrating agent under atmospheric oxygen (Table 25, Entry 21).[495] In 2015 Premi et al. displayed a palladium catalyzed regioselective decarboxylative alkylation of (hetero)arenes with aliphatic carboxylic acids.[496] Another cascaded procedure that also gives access to *ortho*-acyl azoarenes is the palladium catalyzed oxidation/sp$^2$ C-H acylation of azoarenes with ary methanes, which were used as in situ generated acyl sources(Table 25, Entry 1).[497]

**Table 25: Azo- containing directing groups**

| Entry | Directing group | Type of transformation | Coupling partner | Typical product structure | Comments | Ref |
|---|---|---|---|---|---|---|
| 1 | 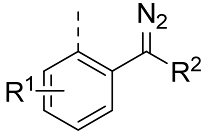 | Cyclization | 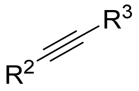 | 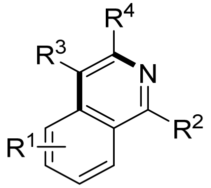 | Substrate (0.2 mmol), alkyne (0.22 mmol), $PhCO_2H$ (0.05 mmol), $[Cp^*RhCl_2]_2$ (0.005 mmol), $CH_2Cl_2$ (1 mL), MeOH (4 mL),<br>80 °C, 12 h<br>26 examples, 68-81 % yield<br>$R^1$: Me, halogenide<br>$R^2$: Aryl, alkyl<br>$R^3$: Aryl, alcohol<br>$R^4$: Aryl | 487 |
| 2 | 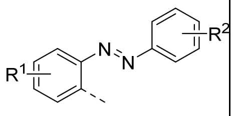 | Acylation | 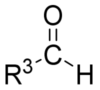 | 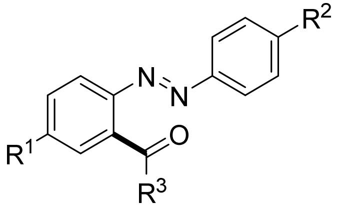 | Substrate (0.20 mmol), aldehyde (0.22 mmol), $Pd(OAc)_2$ (5.0 mol%), TBHP (0.40 mmol), DCE (1.0 mL), 80 °C, sealedtube, $N_2$,<br>12 h<br>24 examples, 54-85 % yield<br>$R^1$, $R^2$: Me, halogenide, OMe<br>$R^3$: Alkyl, aryl, naphtyl, furane, cyclohexyl | 486 |
| 3 | | | $R^3$-$CH_2OH$ | 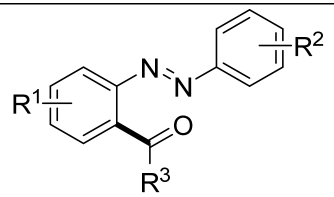 | Substrate (0.15 mmol), alcohol (1.0 equiv.) with $Pd(OAc)_2$ (10 mol%) in the presence of TBHP (4.0 equiv.) in PhCl (2.0 mL) at 80 °C for 30 h under an argon atmosphere<br>30 examples, 20-80 % yield<br>$R^1$, $R^2$: Me, OMe, halogenide, ester<br>$R^3$: Aryl, thiophenyl, alkyl | 498 |

| | | | | | | |
|---|---|---|---|---|---|---|
| 4 | | | Ar-$CH_3$ | 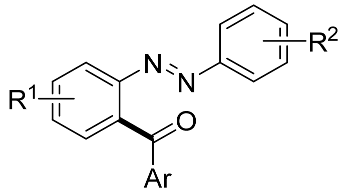 | Substrate (0.15mmol) and toluene (36.0 equiv) with $Pd(OAc)_2$ (10mol%) in the presence of TBHP (12.0 equiv) in $PhCF_3$ (1.0 mL) at 80 °C for 30 h under Ar in a sealed reaction tube; first step: oxidation of toluene<br>34 examples, 15-79 % yield<br>$R^1$, $R^2$: Me, OMe, halogenide, ester, $OCF_3$ | 497 |
| 5 | | | | | Substrate (0.2 mmol), toluene (1.5 mL), $Pd(OAc)_2$ (10 mol%), TBHP (4 equiv), $CH_3CN$ (0.5 mL) at 80 °C<br>Under air atmosphere for 24 h<br>23 examples, 50-91 % yield<br>$R^1$, $R^2$: Me, OMe, halogenide, ester, $OCF_3$ | 488 |
| 6 | | | Ar-$CH_3$ | 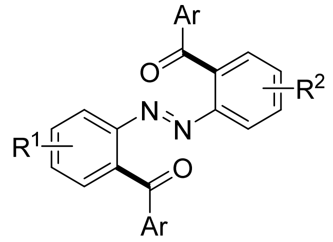 | Substrate (0.2 mmol), toluene derivatives (2 mL), $Pd(OAc)_2$ (10 mol%), TBHP (3 mmol) at 80 °C under air atmosphere for 24 h.<br>10 examples, 53-83 % yield<br>$R^1$, $R^2$: Me, OMe, halogenide | 488 |
| 7 | | | 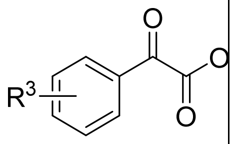 | 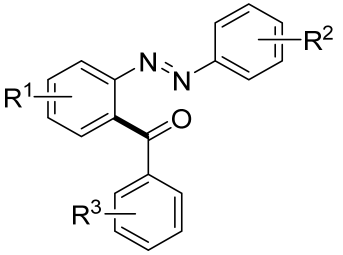 | Substrate (0.3 mmol), α-oxocarboxylicacids (0.33 mmol), $Pd(OAc)_2$ (10 mol%), $K_2S_2O_8$ (2 equiv), dioxane/AcOH/DMSO (7/2/1, 2 mL), 80 °C, 10 h<br>23 examples, 50-88 % yield<br>$R^1$, $R^2$: Me, OMe, OEt, halogenide<br>$R^3$: Me, OMe, halogenide, $CF_3$, aryl | 499 |
| 8 | | Acyloxylation | 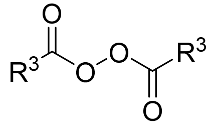 | 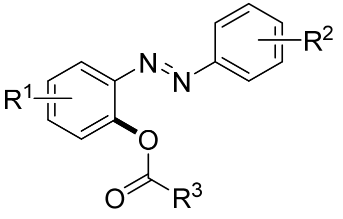 | Substrate (0.15 mmol) and peroxides (2.0 equiv.) was stirred in the presence of $Pd(OAc)_2$ (10 mol%) in $CH_3CN$ (2.0 mL) at 60 °C for 24 h under air conditions in a sealed tube. The ratio | 500 |

| | | | | | | |
|---|---|---|---|---|---|---|
| | | | | | of trans to cis diastereomers was determined by $^1$H NMR spectroscopy<br>20 examples, 29-75 % yield<br>$R^1$, $R^2$: Me, OMe, halogenide, ester; $R^3$: Aryl | |
| 9 | | Alkoxylation | $R^3OH$ | 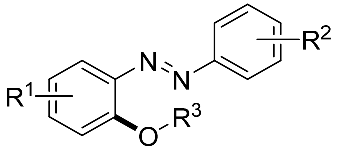 | Substrate (0.5 mmol), alcohol (2 mL), $Pd(OAc)_2$ (10 mol%), and $PhI(OAc)_2$ (1.0 mmol) under an air atmosphere at 80 °C for 24 h<br>24 examples, 35-77 % yield<br>$R^1$, $R^2$: Me, OMe, halogenide, ketone, ester; $R^3$: Alkyl | [489] |
| 10 | | Alkylation | 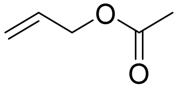 | 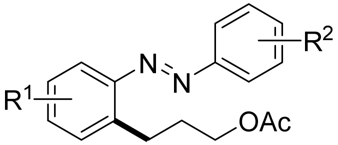 | Substrate (0.40 mmol), allyl acetate (0.30 mmol), $[Cp*RhCl_2]_2$ (3 mol%), $AgSbF_6$ (15 mol%), DCE (1.0mL) at 110 °C in air for 20 h<br>25 examples, 52-80 % yield<br>$R^1$, $R^2$: Me, OMe, halogenide, $CF_3$, *i*-Pr | [501] |
| 11 | | | 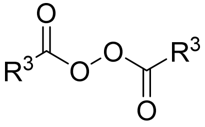 | 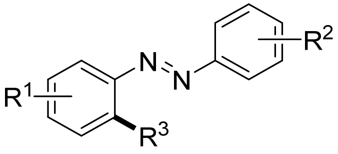 | Substrate (0.15 mmol) and peroxides (2.0 equiv.) was stirred at 130 °C in the presence of $Pd(OAc)_2$ (10 mol%) in PhCl (2.0 mL) for 24 h under air conditions in a sealed tube. The ratio of trans to<br>cis diastereomers was determined by $^1$H NMR spectroscopy<br>18 examples, 23-82 % yield<br>$R^1$, $R^2$: Me, OMe, halogenide, ester<br>$R^3$: Aryl | [500] |
| 12 | | | 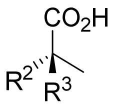 | 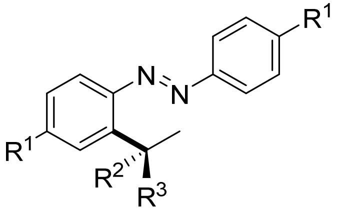 | Substrate (1.0 equiv), carboxylic acid (2.0 equiv), $PhI(OAc)_2$ (2.0equiv), $Pd(OAc)_2$ (10 mol%), 80 °C, 2 h<br>24 examples, 12-77 % yield<br>$R^1$: Me, halogenide<br>$R^2$, $R^3$: Alkyl | [496] |

| | | | | | | |
|---|---|---|---|---|---|---|
| 13 | | | 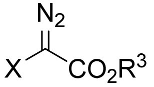 | 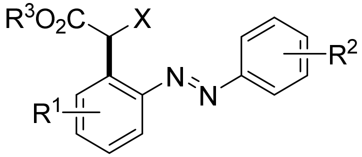 | Substrate (0.2 mmol), $RhCp^*Cl_2]_2$, (2.5 mol %), $AgSbF_6$ (10 mol%), THF (1 mL) under air at 60 °C for 20 h<br>20 examlpes, 23-89 % yield<br>$R^1$, $R^2$: Me, OMe, halogenide, $OCF_3$, ester<br>$R^3$: Alkyl, aryl, *i*-Pr<br>X: $CO_2R$, $SO_2Ph$, $PO(OEt)_2$, COPh | [502] |
| 14 | | Amidation | 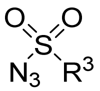 | 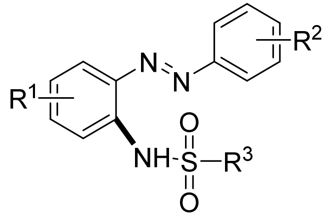 | Substrate (1.5 eq.), $[Cp^*RhCl_2]_2$ (5 mol%); sulfonylazide (1 eq.), $AgNTf_2$ (20 mol%);<br>1,2-dichloroethane; 90 °C; 36 h<br>23 examples, 35-98 % yield<br>$R^1$, $R^2$: Me, OMe, halogenide, $OCF_3$, $NO_2$<br>$R^3$: Alkyl, aryl, thiophenyl, naphtyl | [503] |
| 15 | | Amidation | 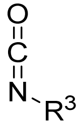 | 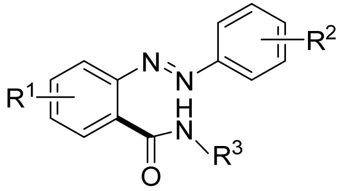 | Substrate (1.25 mmol), isocyanate (0.5 mmol), $Re_2(CO)_{10}$ (0.025 mmol), NaOAc (0.1 mmol), toluene (2.5 mL), 130 °C, 24 h<br>30 examples, 42-86 % yield<br>$R^1$, $R^2$: Me, OMe, $OCF_3$, $OCF_3$, halogenide, aryl<br>$R^3$: Alkyl, aryl, naphtyl | [492] |
| 16 | | Amination | $R^3$-$N_3$ | 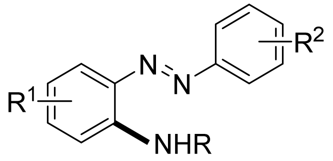 | Substrate (0.5 mmol), $[RhCp^*Cl_2]_2$ (2.5 mol%), $RN_3$ (1.5 eq.); $AgSbF_6$ (10 mol%); DCE; under air; 85 °C;36−40 h; alkyl, aryl, and sulfonylazides could be efficiently assembled in this reaction.<br>35 examples, 46-97 % yield<br>$R^1$, $R^2$: Me, OMe, acetyl, halogenide, ester<br>$R^3$: Aryl, alkyl, sulfonyl | [493] |

| | | | | | | |
|---|---|---|---|---|---|---|
| 17 | | | $TMSN_3$ | 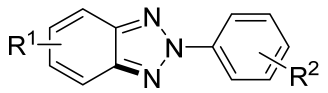 | Substrate (0.5 mmol), $Pd(OAc)_2$, $TMSN_3$ (2 equiv), TBHP-decane (2 equiv) in DMSO (1.0 mL) at 100 °C for 19−50 h<br>18 examples, 8-87 % yield<br>$R^1$, $R^2$: H, Me, OMe, alkyl, halogenide, $CF_3$ | 504 |
| 18 | | | 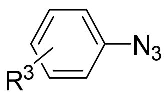 | 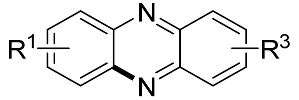 | Substrate (0.2 mmol), azide (0.3 mmol), $[RhCp^*Cl_2]_2$ (5 mol%); $AgB(C_6F_5)_4$ (20 mol %); AcOH; 110 °C; 24 h<br>10 examples, 30-88 % yield<br>$R^1$: Me, halogenide, acetyl, $CF_3$; $R^2$: Me; $R^3$: Me, acetyl, halogenide, $CF_3$ | 505 |
| 19 | | | 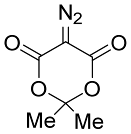 | 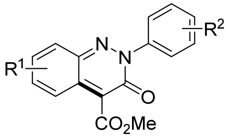 | Substrate (0.2 mmol), diazo derivative of Meldrum's Acid (0.3 mmol), $[RhCp^*Cl_2]_2$ (2.5 mol%), $AgSbF_6$ (10 mol %), MeOH (1 mL), under air at 80 °C for 8 h<br>7 examples, 30-89 % yield<br>$R^1$, $R^2$: Me, halogenide | 502 |
| 20 | | | $R^3CHO$ | 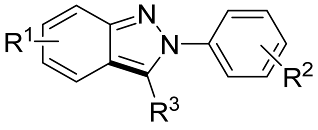 | Substrate (0.20 mmol), aldehyde (0.40 mmol), $[Cp^*RhCl_2]_2$ (5 mol%), $AgSbF_6$, 100mg of $MgSO_4$ in 1.0 mL of THF, 110 °C , 24 h<br>22 examples, 38-81 % yield<br>$R^1$, $R^2$: Me, halogenide, OH, amide, $CF_3$<br>$R^3$: Aryl | 494 |
| 21 | | Nitration | $^tBuONO$ | 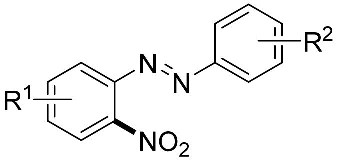 | Substrate (0.5 mmol), *t*BuONO (2.0 mmol), $Pd(CH_3CN)_2Cl_2$ (15 mol%), 1,4-dioxane (3 mL), 90 °C, 20 h, under air<br>19 examples, 40-92 % yield<br>$R^1$, $R^2$: Alkyl, $NO_2$, halogenide, $CF_3$ | 495 |

| | | | | | | |
|---|---|---|---|---|---|---|
| 22 | | Sulfonylation | 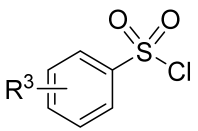 | 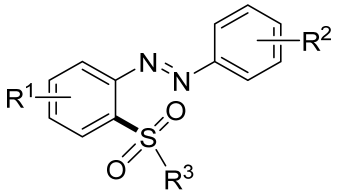 | Substrate (0.5 mmol), arylsulfonyl chlorides (0.6 equiv.), catalyst (10 mol%), base (2.0 equiv.), 4A MS (100 mg) and solvent (2.0 mL) under air at 130 °C for 12 h<br>21 examples, 15-92 % yield<br>$R^1$, $R^2$: Me, OMe, OEt, halogenide<br>$R^3$: Me, OMe, alkyl, halogenide, $NO_2$ | 490 |
| 23 | | | | | Substrate (0.2 mmol), arylsulfonyl chlorides (3.0 equiv), $Pd(TFA)_2$ (5 mol %), and $K_2S_2O_8$ (1.1 equiv) in DCE (2.0 mL) under air at 120 °C for 36 h<br>28 examples, 20-92 % yield<br>$R^1$, $R^2$:  Me, OMe, $OCF_3$, halogenide, ester<br>$R^3$: H, Me, OMe, alkyl, halogenide,CN, $CF_3$ | 491 |

## References


1. S. J. Pastine, D. V. Gribkov and D. Sames, *J. Am. Chem. Soc.*, 2006, **128**, 14220-14221.
2. N. Dastbaravardeh, K. Kirchner, M. Schnuerch and M. D. Mihovilovic, *J. Org. Chem.*, 2013, **78**, 658-672.
3. N. Dastbaravardeh, M. Schnuerch and M. D. Mihovilovic, *Org. Lett.*, 2012, **14**, 1930-1933.
4. V. Smout, A. Peschiulli, S. Verbeeck, E. A. Mitchell, W. Herrebout, P. Bultinck, V. C. M. L. Vande, D. Berthelot, L. Meerpoel and B. U. W. Maes, *J. Org. Chem.*, 2013, **78**, 9803-9814.
5. M. C. Schwarz, N. Dastbaravardeh, K. Kirchner, M. Schnuerch and M. D. Mihovilovic, *Monatsh. Chem.*, 2013, **144**, 539-552.
6. N. Dastbaravardeh, M. Schnuerch and M. D. Mihovilovic, *Eur. J. Org. Chem.*, 2013, **2013**, 2878-2890.
7. N. Dastbaravardeh, M. Schnuerch and M. D. Mihovilovic, *Org. Lett.*, 2012, **14**, 3792-3795.
8. M. Koley, N. Dastbaravardeh, M. Schnürch and M. D. Mihovilovic, *ChemCatChem*, 2012, **4**, 1345-1352.
9. J. Chen, L. He, K. Natte, H. Neumann, M. Beller and X.-F. Wu, *Adv. Synth. Catal.*, 2014, **356**, 2955-2959.
10. W. Song and L. Ackermann, *Angew. Chem., Int. Ed.*, 2012, **51**, 8251-8254.
11. H. Wang, S. Yu, Z. Qi and X. Li, *Org. Lett.*, 2015, **17**, 2812-2815.
12. S. Xu, X. Huang, X. Hong and B. Xu, *Org. Lett.*, 2012, **14**, 4614-4617.
13. Z. Wang, F. Song, Y. Zhao, Y. Huang, L. Yang, D. Zhao, J. Lan and J. You, *Chemistry*, 2012, **18**, 16616-16620.
14. X.-F. Yang, X.-H. Hu, C. Feng and T.-P. Loh, *Chem. Commun.*, 2015, **51**, 2532-2535.
15. R. Mei, J. Loup and L. Ackermann, *ACS Catal.*, 2016, **6**, 793-797.
16. B. Punji, W. Song, G. A. Shevchenko and L. Ackermann, *Chem. - Eur. J.*, 2013, **19**, 10605-10610.
17. X. Cong, J. You, G. Gao and J. Lan, *Chem. Commun.*, 2013, **49**, 662-664.
18. C.-H. Jun, *Chem. Commun.*, 1998, DOI: 10.1039/A801298I, 1405-1406.
19. S. Pan, K. Endo and T. Shibata, *Org. Lett.*, 2011, **13**, 4692-4695.
20. S. Pan, Y. Matsuo, K. Endo and T. Shibata, *Tetrahedron*, 2012, **68**, 9009-9015.
21. L. Shi, X. Zhong, H. She, Z. Lei and F. Li, *Chem. Commun.*, 2015, **51**, 7136-7139.
22. S. D. Bergman, T. E. Storr, H. Prokopcova, K. Aelvoet, G. Diels, L. Meerpoel and B. U. W. Maes, *Chem. - Eur. J.*, 2012, **18**, 10393-10398, S10393/10391-S10393/10363.
23. A. A. Kulago, B. F. Van Steijvoort, E. A. Mitchell, L. Meerpoel and B. U. W. Maes, *Adv. Synth. Catal.*, 2014, **356**, 1610-1618.
24. M. Schinkel, I. Marek and L. Ackermann, *Angew. Chem., Int. Ed.*, 2013, **52**, 3977-3980.

25. X. Li, S. Yu, F. Wang, B. Wan and X. Yu, *Angew. Chem., Int. Ed.*, 2013, **52**, 2577-2580.
26. S. Yu and X. Li, *Org. Lett.*, 2014, **16**, 1200-1203.
27. H. Cheng, W. Dong, C. A. Dannenberg, S. Dong, Q. Guo and C. Bolm, *ACS Catal.*, 2015, **5**, 2770-2773.
28. F. Xie, Z. Qi, S. Yu and X. Li, *J. Am. Chem. Soc.*, 2014, **136**, 4780-4787.
29. H. Xu, X. Qiao, S. Yang and Z. Shen, *J. Org. Chem.*, 2014, **79**, 4414-4422.
30. W. Liu, J. Bang, Y. Zhang and L. Ackermann, *Angew. Chem., Int. Ed.*, 2015, **54**, 14137-14140.
31. S. Gu, C. Chen and W. Chen, *J. Org. Chem.*, 2009, **74**, 7203-7206.
32. H. Prokopcova, S. D. Bergman, K. Aelvoet, V. Smout, W. Herrebout, B. Van der Veken, L. Meerpoel and B. U. W. Maes, *Chem. - Eur. J.*, 2010, **16**, 13063-13067, S13063/13061-S13063/13032.
33. J.-H. Chu, P.-S. Lin, Y.-M. Lee, W.-T. Shen and M.-J. Wu, *Chem. - Eur. J.*, 2011, **17**, 13613-13620.
34. J.-H. Chu, C.-C. Wu, D.-H. Chang, Y.-M. Lee and M.-J. Wu, *Organometallics*, 2013, **32**, 272-282.
35. X. Zhang, F. Wang, Z. Qi, S. Yu and X. Li, *Org. Lett.*, 2014, **16**, 1586-1589.
36. N. Chatani, S. Yorimitsu, T. Asaumi, F. Kakiuchi and S. Murai, *J. Org. Chem.*, 2002, **67**, 7557-7560.
37. K. Parthasarathy, A. R. Azcargorta, Y. Cheng and C. Bolm, *Org. Lett.*, 2014, **16**, 2538-2541.
38. R. Qiu, V. P. Reddy, T. Iwasaki and N. Kambe, *J. Org. Chem.*, 2015, **80**, 367-374.
39. M. Choi, J. Park, S. Sharma, H. Jo, S. Han, M. Jeon, N. K. Mishra, S. H. Han, J. S. Lee and I. S. Kim, *J. Org. Chem.*, 2016, **81**, 4771-4778.
40. M. Wang, Y. Yang, Z. Fan, Z. Cheng, W. Zhu and A. Zhang, *Chem. Commun.*, 2015, **51**, 3219-3222.
41. J.-H. Chu, P.-S. Lin and M.-J. Wu, *Organometallics*, 2010, **29**, 4058-4065.
42. J.-H. Chu, H.-P. Huang, W.-T. Hsu, S.-T. Chen and M.-J. Wu, *Organometallics*, 2014, **33**, 1190-1204.
43. P. L. Alsters, P. F. Engel, M. P. Hogerheide, M. Copijn, A. L. Spek and G. van Koten, *Organometallics*, 1993, **12**, 1831-1844.
44. V. G. Zaitsev, D. Shabashov and O. Daugulis, *J. Am. Chem. Soc.*, 2005, **127**, 13154-13155.
45. Y. Feng and G. Chen, *Angew. Chem., Int. Ed.*, 2010, **49**, 958-961.
46. W. R. Gutekunst, R. Gianatassio and P. S. Baran, *Angew. Chem., Int. Ed.*, 2012, **51**, 7507-7510.
47. Z. Wang, Y. Kuninobu and M. Kanai, *Org. Lett.*, 2014, **16**, 4790-4793.
48. Y. Aihara and N. Chatani, *Chem. Sci.*, 2013, **4**, 664-670.
49. G. Rouquet and N. Chatani, *Chem. Sci.*, 2013, **4**, 2201-2208.
50. Y. Aihara and N. Chatani, *J. Am. Chem. Soc.*, 2013, **135**, 5308-5311.
51. R. Shang, L. Ilies and E. Nakamura, *J. Am. Chem. Soc.*, 2015, **137**, 7660-7663.

52. E. R. Fruchey, B. M. Monks and S. P. Cook, *J. Am. Chem. Soc.*, 2014, **136**, 13130-13133.

53. H. Tang, X.-R. Huang, J. Yao and H. Chen, *J. Org. Chem.*, 2015, **80**, 4672-4682.

54. G. Rouquet and N. Chatani, *Angew. Chem., Int. Ed.*, 2013, **52**, 11726-11743.

55. L. C. M. Castro and N. Chatani, *Chem. Lett.*, 2015, **44**, 410-421.

56. X.-Q. Hao, L.-J. Chen, B. Ren, L.-Y. Li, X.-Y. Yang, J.-F. Gong, J.-L. Niu and M.-P. Song, *Org. Lett.*, 2014, **16**, 1104-1107.

57. L.-B. Zhang, X.-Q. Hao, S.-K. Zhang, K. Liu, B. Ren, J.-F. Gong, J.-L. Niu and M.-P. Song, *J. Org. Chem.*, 2014, **79**, 10399-10409.

58. L.-B. Zhang, X.-Q. Hao, S.-K. Zhang, Z.-J. Liu, X.-X. Zheng, J.-F. Gong, J.-L. Niu and M.-P. Song, *Angew. Chem., Int. Ed.*, 2015, **54**, 272-275.

59. H.-L. Wang, M. Shang, S.-Z. Sun, Z.-L. Zhou, B. N. Laforteza, H.-X. Dai and J.-Q. Yu, *Org. Lett.*, 2015, **17**, 1228-1231.

60. M. Shang, S.-Z. Sun, H.-X. Dai and J.-Q. Yu, *J. Am. Chem. Soc.*, 2014, **136**, 3354-3357.

61. M. Shang, S.-Z. Sun, H.-X. Dai and J.-Q. Yu, *Org. Lett.*, 2014, **16**, 5666-5669.

62. B. V. S. Reddy, G. Revathi, A. S. Reddy and J. S. Yadav, *Tetrahedron Lett.*, 2011, **52**, 5926-5929.

63. B. V. S. Reddy, G. Revathi, A. S. Reddy and J. S. Yadav, *Synlett*, 2011, DOI: 10.1055/s-0030-1260314, 2374-2378.

64. N. Laidaoui, J. Roger, A. Miloudi, D. El Abed and H. Doucet, *Eur. J. Org. Chem.*, 2011, **2011**, 4373-4385.

65. S. Inoue, H. Shiota, Y. Fukumoto and N. Chatani, *J. Am. Chem. Soc.*, 2009, **131**, 6898-6899.

66. K. Shibata, N. Hasegawa, Y. Fukumoto and N. Chatani, *ChemCatChem*, 2012, **4**, 1733-1736.

67. Q. Zhang, K. Chen, W. Rao, Y. Zhang, F.-J. Chen and B.-F. Shi, *Angew. Chem., Int. Ed.*, 2013, **52**, 13588-13592.

68. Q. Zhang, X.-S. Yin, S. Zhao, S.-L. Fang and B.-F. Shi, *Chem. Commun.*, 2014, **50**, 8353-8355.

69. X. Li, Y.-H. Liu, W.-J. Gu, B. Li, F.-J. Chen and B.-F. Shi, *Org. Lett.*, 2014, **16**, 3904-3907.

70. F.-J. Chen, S. Zhao, F. Hu, K. Chen, Q. Zhang, S.-Q. Zhang and B.-F. Shi, *Chem. Sci.*, 2013, **4**, 4187-4192.

71. N. Hasegawa, K. Shibata, V. Charra, S. Inoue, Y. Fukumoto and N. Chatani, *Tetrahedron*, 2013, **69**, 4466-4472.

72. L. Huang, X. Sun, Q. Li and C. Qi, *J. Org. Chem.*, 2014, **79**, 6720-6725.

73. E. T. Nadres, G. I. F. Santos, D. Shabashov and O. Daugulis, *J. Org. Chem.*, 2013, **78**, 9689-9714.

74. G. He, Y. Zhao, S. Zhang, C. Lu and G. Chen, *J. Am. Chem. Soc.*, 2012, **134**, 3-6.

75. Y. F. Zhang, H. W. Zhao, H. Wang, J. B. Wei and Z. J. Shi, *Angew. Chem., Int. Ed.*, 2015, **54**, 13686-13690.

76. V. L. Blair, D. C. Blakemore, D. Hay, E. Hevia and D. C. Pryde, *Tetrahedron Letters*, 2011, **52**, 4590-4594.

77. E. T. Nadres and O. Daugulis, *J. Am. Chem. Soc.*, 2012, **134**, 7-10.

78. C. Lu, S.-Y. Zhang, G. He, W. A. Nack and G. Chen, *Tetrahedron*, 2014, **70**, 4197-4203.

79. S. Allu and K. C. K. Swamy, *J. Org. Chem.*, 2014, **79**, 3963-3972.

80. L. Grigorjeva and O. Daugulis, *Angew. Chem., Int. Ed.*, 2014, **53**, 10209-10212.
81. W. Miura, K. Hirano and M. Miura, *Org. Lett.*, 2015, **17**, 4034-4037.
82. R. Shang, L. Ilies, S. Asako and E. Nakamura, *J. Am. Chem. Soc.*, 2014, **136**, 14349-14352.
83. M. Li, Y. Yang, D. Zhou, D. Wan and J. You, *Org. Lett.*, 2015, **17**, 2546-2549.
84. W. R. Gutekunst and P. S. Baran, *J. Org. Chem.*, 2014, **79**, 2430-2452.
85. L. Ilies, T. Matsubara, S. Ichikawa, S. Asako and E. Nakamura, *J. Am. Chem. Soc.*, 2014, **136**, 13126-13129.
86. L. Ilies, S. Ichikawa, S. Asako, T. Matsubara and E. Nakamura, *Adv. Synth. Catal.*, 2015, **357**, 2175-2179.
87. A. Yokota, Y. Aihara and N. Chatani, *J. Org. Chem.*, 2014, **79**, 11922-11932.
88. S.-Y. Zhang, Q. Li, G. He, W. A. Nack and G. Chen, *J. Am. Chem. Soc.*, 2015, **137**, 531-539.
89. D. Shabashov and O. Daugulis, *J. Am. Chem. Soc.*, 2010, **132**, 3965-3972.
90. K. Shibata, T. Yamaguchi and N. Chatani, *Org. Lett.*, 2015, **17**, 3584-3587.
91. K. Shibata and N. Chatani, *Org. Lett.*, 2014, **16**, 5148-5151.
92. B. M. Monks, E. R. Fruchey and S. P. Cook, *Angew. Chem., Int. Ed.*, 2014, **53**, 11065-11069.
93. J. Liu, Y. Xie, W. Zeng, D. Lin, Y. Deng and X. Lu, *J. Org. Chem.*, 2015, **80**, 4618-4626.
94. Y. Ano, M. Tobisu and N. Chatani, *Org. Lett.*, 2012, **14**, 354-357.
95. M. Al-Amin, M. Arisawa, S. Shuto, Y. Ano, M. Tobisu and N. Chatani, *Adv. Synth. Catal.*, 2014, **356**, 1631-1637.
96. S. Asako, L. Ilies and E. Nakamura, *J. Am. Chem. Soc.*, 2013, **135**, 17755-17757.
97. X. Wu, Y. Zhao and H. Ge, *Chem. - Eur. J.*, 2014, **20**, 9530-9533.
98. G. He, S.-Y. Zhang, W. A. Nack, Q. Li and G. Chen, *Angew. Chem., Int. Ed.*, 2013, **52**, 11124-11128.
99. Z. Wang, J. Ni, Y. Kuninobu and M. Kanai, *Angew. Chem., Int. Ed.*, 2014, **53**, 3496-3499.
100. Q. Yan, Z. Chen, W. Yu, H. Yin, Z. Liu and Y. Zhang, *Org. Lett.*, 2015, **17**, 2482-2485.
101. N. T. T. Tran, Q. H. Tran and T. Truong, *J. Catal.*, 2014, **320**, 9-15.
102. T. Matsubara, S. Asako, L. Ilies and E. Nakamura, *J. Am. Chem. Soc.*, 2014, **136**, 646-649.
103. L. D. Tran, J. Roane and O. Daugulis, *Angew. Chem., Int. Ed.*, 2013, **52**, 6043-6046.
104. W. Zhu, D. Zhang, N. Yang and H. Liu, *Chem. Commun.*, 2014, **50**, 10634-10636.
105. Y. Wei, H. Tang, X. Cong, B. Rao, C. Wu and X. Zeng, *Org. Lett.*, 2014, **16**, 2248-2251.
106. M. Iyanaga, Y. Aihara and N. Chatani, *J. Org. Chem.*, 2014, **79**, 11933-11939.
107. N. Hoshiya, T. Kobayashi, M. Arisawa and S. Shuto, *Org. Lett.*, 2013, **15**, 6202-6205.

108. D. P. Affron, O. A. Davis and J. A. Bull, *Org. Lett.*, 2014, **16**, 4956-4959.
109. R. Feng, B. Wang, Y. Liu, Z. Liu and Y. Zhang, *Eur. J. Org. Chem.*, 2015, **2015**, 142-151.
110. Y. Aihara and N. Chatani, *J. Am. Chem. Soc.*, 2014, **136**, 898-901.
111. B. Gopalakrishnan, S. A. Babu and R. Padmavathi, *Tetrahedron*, 2015, **71**, 8333-8349.
112. Y. Liu, Y. Zhang, M. Huang and J.-P. Wan, *RSC Adv.*, 2015, **5**, 46192-46196.
113. P. Hu and T. Bach, *Synlett*, 2015, **26**, 2853-2857.
114. X. Wu, Y. Zhao and H. Ge, *J. Am. Chem. Soc.*, 2015, **137**, 4924-4927.
115. L. Grigorjeva and O. Daugulis, *Org. Lett.*, 2014, **16**, 4688-4690.
116. J. Roane and O. Daugulis, *Org. Lett.*, 2013, **15**, 5842-5845.
117. T. Truong, K. Klimovica and O. Daugulis, *J. Am. Chem. Soc.*, 2013, **135**, 9342-9345.
118. J. Liu, S. Zhuang, Q. Gui, X. Chen, Z. Yang and Z. Tan, *Adv. Synth. Catal.*, 2015, **357**, 732-738.
119. S. Wang, R. Guo, G. Wang, S.-Y. Chen and X.-Q. Yu, *Chem. Commun.*, 2014, **50**, 12718-12721.
120. K. S. Kanyiva, Y. Kuninobu and M. Kanai, *Org. Lett.*, 2014, **16**, 1968-1971.
121. L. D. Tran, I. Popov and O. Daugulis, *J. Am. Chem. Soc.*, 2012, **134**, 18237-18240.
122. V. P. Reddy, R. Qiu, T. Iwasaki and N. Kambe, *Org. Biomol. Chem.*, 2015, **13**, 6803-6813.
123. H.-Y. Xiong, T. Besset, D. Cahard and X. Pannecoucke, *J. Org. Chem.*, 2015, **80**, 4204-4212.
124. X.-B. Yan, P. Gao, H.-B. Yang, Y.-X. Li, X.-Y. Liu and Y.-M. Liang, *Tetrahedron*, 2014, **70**, 8730-8736.
125. J. Liu, L. Yu, S. Zhuang, Q. Gui, X. Chen, W. Wang and Z. Tan, *Chem. Commun.* , 2015, **51**, 6418-6421.
126. A. Yokota and N. Chatani, *Chem. Lett.*, 2015, **44**, 902-904.
127. Y. Aihara, M. Tobisu, Y. Fukumoto and N. Chatani, *J. Am. Chem. Soc.*, 2014, **136**, 15509-15512.
128. R. Parella, B. Gopalakrishnan and S. A. Babu, *Org. Lett.*, 2013, **15**, 3238-3241.
129. W. R. Gutekunst and P. S. Baran, *J. Am. Chem. Soc.*, 2011, **133**, 19076-19079.
130. M. Moselage, N. Sauermann, S. C. Richter and L. Ackermann, *Angew. Chem., Int. Ed.*, 2015, **54**, 6352-6355.
131. B. Gong, J. Shi, X. Wang, Y. Yan, Q. Li, Y. Meng, H. E. Xu and W. Yi, *Adv. Synth. Catal.*, 2014, **356**, 137-143.
132. Z. Ding and N. Yoshikai, *Beilstein J. Org. Chem.*, 2012, **8**, 1536-1542, No. 1174.
133. C. Premi, A. Dixit and N. Jain, *Org. Lett.*, 2015, **17**, 2598-2601.
134. L. Zhang, X. Xue, C. Xu, Y. Pan, G. Zhang, L. Xu, H. Li and Z. Shi, *ChemCatChem*, 2014, **6**, 3069-3074.
135. J. Atzrodt, V. Derdau, W. J. Kerr, M. Reid, P. Rojahn and R. Weck, *Tetrahedron*, 2015, **71**, 1924-1929.

136. J. Shi, G. Zhao, X. Wang, H. E. Xu and W. Yi, *Org. Biomol. Chem.*, 2014, **12**, 6831-6836.
137. T. Jeong, S. Han, N. K. Mishra, S. Sharma, S.-Y. Lee, J. S. Oh, J. H. Kwak, Y. H. Jung and I. S. Kim, *J. Org. Chem.*, 2015, **80**, 7243-7250.
138. S. Yu and X. Li, *Org. Lett.*, 2014, **16**, 1220-1223.
139. Q. Yu, L.-a. Hu, Y. Wang, S. Zheng and J. Huang, *Angew. Chem., Int. Ed.*, 2015, **54**, 15284-15288.
140. M. Nishino, K. Hirano, T. Satoh and M. Miura, *Angew. Chem., Int. Ed.*, 2012, **51**, 6993-6997.
141. W. Song and L. Ackermann, *Chem. Commun.*, 2013, **49**, 6638-6640.
142. B. Zhou, J. Du, Y. Yang, H. Feng and Y. Li, *Org. Lett.*, 2014, **16**, 592-595.
143. I. E. Iagafarova, D. V. Vorobyeva, A. S. Peregudov and S. N. Osipov, *Eur. J. Org. Chem.*, 2015, **2015**, 4950-4955.
144. X.-G. Liu, S.-S. Zhang, J.-Q. Wu, Q. Li and H. Wang, *Tetrahedron Lett.*, 2015, **56**, 4093-4095.
145. R. Goikhman, T. L. Jacques and D. Sames, *J. Am. Chem. Soc.*, 2009, **131**, 3042-3048.
146. Y. Fall, H. Doucet and M. Santelli, *Synthesis*, 2010, **2010**, 127-135.
147. W. Yang, S. Ye, D. Fanning, T. Coon, Y. Schmidt, P. Krenitsky, D. Stamos and J.-Q. Yu, *Angew. Chem., Int. Ed.*, 2015, **54**, 2501-2504.
148. Y. Hashimoto, K. Hirano, T. Satoh, F. Kakiuchi and M. Miura, *J. Org. Chem.*, 2013, **78**, 638-646.
149. C. Pan, N. Jin, H. Zhang, J. Han and C. Zhu, *J. Org. Chem.*, 2014, **79**, 9427-9432.
150. H. M. L. Davies and T. Hansen, *J. Am. Chem. Soc.*, 1997, **119**, 9075-9076.
151. H. M. L. Davies and R. E. J. Beckwith, *Chem. Rev.* , 2003, **103**, 2861-2903.
152. H. M. L. Davies and J. R. Manning, *Nature*, 2008, **451**, 417-424.
153. W.-W. Chan, S.-F. Lo, Z. Zhou and W.-Y. Yu, *J. Am. Chem. Soc.*, 2012, **134**, 13565-13568.
154. T. Asaumi, N. Chatani, T. Matsuo, F. Kakiuchi and S. Murai, *J. Org. Chem.*, 2003, **68**, 7538-7540.
155. R. Azpiroz, L. Rubio-Perez, A. Di Giuseppe, V. Passarelli, F. J. Lahoz, R. Castarlenas, J. J. Perez-Torrente and L. A. Oro, *ACS Catal.*, 2014, **4**, 4244-4253.
156. Z. Fan, S. Song, W. Li, K. Geng, Y. Xu, Z.-H. Miao and A. Zhang, *Org. Lett.*, 2015, **17**, 310-313.
157. S. Oi, H. Sasamoto, R. Funayama and Y. Inoue, *Chem. Lett.*, 2008, **37**, 994-995.
158. P. Arockiam, V. Poirier, C. Fischmeister, C. Bruneau and P. H. Dixneuf, *Green Chemistry*, 2009, **11**, 1871-1875.
159. G. M. Reddy, N. S. S. Rao, P. Satyanarayana and H. Maheswaran, *RSC Adv.*, 2015, **5**, 105347-105352.
160. D. Kalyani, A. R. Dick, W. Q. Anani and M. S. Sanford, *Tetrahedron*, 2006, **62**, 11483-11498.
161. F. Kakiuchi, M. Matsumoto, K. Tsuchiya, K. Igi, T. Hayamizu, N. Chatani and S. Murai, *Journal of Organometallic Chemistry*, 2003, **686**, 134-144.
162. L. Ackermann, R. Jeyachandran, H. K. Potukuchi, P. Novak and L. Buttner, *Org. Lett.*, 2010, **12**, 2056-2059.
163. L. Ackermann and H. K. Potukuchi, *Org. Biomol. Chem.*, 2010, **8**, 4503-4513.

164. L. Ackermann, R. Vicente and R. Born, *Adv. Synth. Catal.*, 2008, **350**, 741-748.

165. Z. Wang, Q. Tian, X. Yu and C. Kuang, *Adv. Synth. Catal.*, 2014, **356**, 961-966.

166. Z. Wang and C. Kuang, *Adv. Synth. Catal.*, 2014, **356**, 1549-1554.

167. L. Ackermann, R. Vicente and A. Althammer, *Org. Lett.*, 2008, **10**, 2299-2302.

168. L. Ackermann, R. Born and R. Vicente, *ChemSusChem*, 2009, **2**, 546-549.

169. Q. Gu, H. H. Al Mamari, K. Graczyk, E. Diers and L. Ackermann, *Angew. Chem., Int. Ed.*, 2014, **53**, 3868-3871.

170. K. Graczyk, T. Haven and L. Ackermann, *Chem. - Eur. J.*, 2015, **21**, 8812-8815.

171. H. H. Al Mamari, E. Diers and L. Ackermann, *Chem. - Eur. J.*, 2014, **20**, 9739-9743.

172. G. Zhang, X. Xie, J. Zhu, S. Li, C. Ding and P. Ding, *Org. Biomol. Chem.*, 2015, **13**, 5444-5449.

173. X. Ye and X. Shi, *Org. Lett.*, 2014, **16**, 4448-4451.

174. F. Peron, C. Fossey, J. Sopkova-de Oliveira Santos, T. Cailly and F. Fabis, *Chem. - Eur. J.*, 2014, **20**, 7507-7513.

175. X. Ye, Z. He, T. Ahmed, K. Weise, N. G. Akhmedov, J. L. Petersen and X. Shi, *Chem. Sci.*, 2013, **4**, 3712-3716.

176. P. Sadhu, S. K. Alla and T. Punniyamurthy, *J. Org. Chem.*, 2015, **80**, 8245-8253.

177. C. F. Matta, A. A. Arabi and D. F. Weaver, *Eur. J. Med. Chem.*, 2010, **45**, 1868-1872.

178. K. Pegklidou, C. Koukoulitsa, I. Nicolaou and V. J. Demopoulos, *Bioorg. Med. Chem.*, 2010, **18**, 2107-2114.

179. T. Pinter, S. Jana, R. J. M. Courtemanche and F. Hof, *J. Org. Chem.*, 2011, **76**, 3733-3741.

180. F. H. Allen, C. R. Groom, J. W. Liebeschuetz, D. A. Bardwell, T. S. G. Olsson and P. A. Wood, *J. Chem. Inf. Model.*, 2012, **52**, 857-866.

181. W.-H. Song, M.-M. Liu, D.-W. Zhong, Y.-l. Zhu, M. Bosscher, L. Zhou, D.-Y. Ye and Z.-H. Yuan, *Bioorg. Med. Chem. Lett.*, 2013, **23**, 4528-4531.

182. L. Wang, W. Wu, Q. Chen and M. He, *Org. Biomol. Chem.*, 2014, **12**, 7923-7926.

183. B. Chen, Y. Jiang, J. Cheng and J.-T. Yu, *Org. Biomol. Chem.*, 2015, **13**, 2901-2904.

184. M. Seki, *ACS Catalysis*, 2011, **1**, 607-610.

185. M. Seki, *Synthesis*, 2012, **44**, 3231-3237.

186. M. Seki, *ACS Catalysis*, 2014, **4**, 4047-4050.

187. M. Seki and M. Nagahama, *J. Org. Chem.*, 2011, **76**, 10198-10206.

188. E. Diers, N. Y. Phani Kumar, T. Mejuch, I. Marek and L. Ackermann, *Tetrahedron*, 2013, **69**, 4445-4453.

189. L. Zhang, L. Zheng, B. Guo and R. Hua, *J. Org. Chem.*, 2014, **79**, 11541-11548.

190. Y. Matsuura, M. Tamura, T. Kochi, M. Sato, N. Chatani and F. Kakiuchi, *J. Am. Chem. Soc.*, 2007, **129**, 9858-9859.

191. Y.-J. Ding, Y. Li, S.-Y. Dai, Q. Lan and X.-S. Wang, *Org. Biomol. Chem.*, 2015, **13**, 3198-3201.

192. F. Derridj, A. L. Gottumukkala, S. Djebbar and H. Doucet, *Eur. J. Inorg. Chem.*, 2008, DOI: 10.1002/ejic.200800143, 2550-2559.
193. C. Shih and J. S. Swenton, *J. Org. Chem.*, 1982, **47**, 2668-2670.
194. D. P. Phillion and J. K. Pratt, *Synth. Commun.*, 1992, **22**, 13-22.
195. D. Yang, Y.-C. Yip and X.-C. Wang, *Tetrahedron Lett.*, 1997, **38**, 7083-7086.
196. Y. Ie, N. Chatani, T. Ogo, D. R. Marshall, T. Fukuyama, F. Kakiuchi and S. Murai, *J. Org. Chem.*, 2000, **65**, 1475-1488.
197. X. Chen, J.-J. Li, X.-S. Hao, C. E. Goodhue and J.-Q. Yu, *J. Am. Chem. Soc.*, 2006, **128**, 78-79.
198. Y. Ebe and T. Nishimura, *J. Am. Chem. Soc.*, 2015, **137**, 5899-5902.
199. K. K. Pasunooti, B. Banerjee, T. Yap, Y. Jiang and C.-F. Liu, *Org. Lett.*, 2015, **17**, 6094-6097.
200. P.-X. Ling, S.-L. Fang, X.-S. Yin, K. Chen, B.-Z. Sun and B.-F. Shi, *Chemistry*, 2015, **21**, 17503-17507.
201. K. Chen, Z.-W. Li, P.-X. Shen, H.-W. Zhao and Z.-J. Shi, *Chem. - Eur. J.*, 2015, **21**, 7389-7393.
202. L. Ackermann, A. Althammer and R. Born, *Angew. Chem., Int. Ed.*, 2006, **45**, 2619-2622.
203. B. Li, K. Devaraj, C. Darcel and P. H. Dixneuf, *Tetrahedron*, 2012, **68**, 5179-5184.
204. R. Giri, X. Chen and J.-Q. Yu, *Angewandte Chemie*, 2005, **44**, 2112-2115.
205. R. Giri, M. Wasa, S. P. Breazzano and J.-Q. Yu, *Org. Lett.*, 2006, **8**, 5685-5688.
206. R. Giri, X. Chen, X.-S. Hao, J.-J. Li, J. Liang, Z.-P. Fan and J.-Q. Yu, *Tetrahedron: Asymm.*, 2005, **16**, 3502-3505.
207. R. Giri, Y. Lan, P. Liu, K. N. Houk and J.-Q. Yu, *J. Am. Chem. Soc.*, 2012, **134**, 14118-14126.
208. H.-L. Wang, M. Shang, S.-Z. Sun, Z.-L. Zhou, B. N. Laforteza, H.-X. Dai and J.-Q. Yu, *Org. Lett.*, 2015, **17**, 1228-1231.
209. Q. Zhang, C. Li, F. Yang, J. Li and Y. Wu, *Tetrahedron*, 2013, **69**, 320-326.
210. S. Takebayashi, T. Shizuno, T. Otani and T. Shibata, *Beilstein J. Org. Chem.*, 2012, **8**, 1844-1848, No. 1212.
211. B. Li, K. Devaraj, C. Darcel and P. H. Dixneuf, *Green Chem.*, 2012, **14**, 2706-2709.
212. T.-J. Gong, B. Xiao, W.-M. Cheng, W. Su, J. Xu, Z.-J. Liu, L. Liu and Y. Fu, *J. Am. Chem. Soc.*, 2013, **135**, 10630-10633.
213. J.-B. Xia and S.-L. You, *Organometallics*, 2007, **26**, 4869-4871.
214. S.-J. Lou, D.-Q. Xu, A.-B. Xia, Y.-F. Wang, Y.-K. Liu, X.-H. Du and Z.-Y. Xu, *Chem. Commun.*, 2013, **49**, 6218-6220.
215. D. Kalyani, N. R. Deprez, L. V. Desai and M. S. Sanford, *J. Am. Chem. Soc.*, 2005, **127**, 7330-7331.
216. H. Sun, C. Wang, Y.-F. Yang, P. Chen, Y.-D. Wu, X. Zhang and Y. Huang, *J. Org. Chem.*, 2014, **79**, 11863-11872.
217. D. Ghorai and J. Choudhury, *Chem. Commun.* , 2014, **50**, 15159-15162.
218. G. Qian, X. Hong, B. Liu, H. Mao and B. Xu, *Org. Lett.*, 2014, **16**, 5294-5297.
219. Y. Xue, Z. Fan, X. Jiang, K. Wu, M. Wang, C. Ding, Q. Yao and A. Zhang, *Eur. J. Org. Chem.*, 2014, **2014**, 7481-7488.

220. Y. Unoh, K. Hirano, T. Satoh and M. Miura, *Org. Lett.*, 2015, **17**, 704-707.

221. Y. Kuninobu, Y. Fujii, T. Matsuki, Y. Nishina and K. Takai, *Org. Lett.*, 2009, **11**, 2711-2714.

222. H. J. Kim, M. J. Ajitha, Y. Lee, J. Ryu, J. Kim, Y. Lee, Y. Jung and S. Chang, *J. Am. Chem. Soc.*, 2014, **136**, 1132-1140.

223. S. Rajkumar, S. Karthik and T. Gandhi, *J. Org. Chem.*, 2015, **80**, 5532-5545.

224. D. Zhao, M.-X. Zhu, Y. Wang, Q. Shen and J.-X. Li, *Org. Biomol. Chem.*, 2013, **11**, 6246-6249.

225. J. Ryu, K. Shin, S. H. Park, J. Y. Kim and S. Chang, *Angew. Chem., Int. Ed.*, 2012, **51**, 9904-9908.

226. K. Shin, S.-W. Park and S. Chang, *J. Am. Chem. Soc.*, 2015, **137**, 8584-8592.

227. S. Sharma, E. Park, J. Park and I. S. Kim, *Org. Lett.*, 2012, **14**, 906-909.

228. T. Shirai, H. Ito and Y. Yamamoto, *Angew. Chem., Int. Ed.*, 2014, **53**, 2658-2661.

229. T. Shirai and Y. Yamamoto, *Organometallics*, 2015, **34**, 3459-3463.

230. T. E. Hurst, T. K. Macklin, M. Becker, E. Hartmann, W. Kuegel, J.-C. Parisienne-La Salle, A. S. Batsanov, T. B. Marder and V. Snieckus, *Chem. - Eur. J.*, 2010, **16**, 8155-8161.

231. N. Kuhl, N. Schroeder and F. Glorius, *Org. Lett.*, 2013, **15**, 3860-3863.

232. H. Wang, B. Beiring, D.-G. Yu, K. D. Collins and F. Glorius, *Angew. Chem., Int. Ed.*, 2013, **52**, 12430-12434.

233. K.-J. Xiao, D. W. Lin, M. Miura, R.-Y. Zhu, W. Gong, M. Wasa and J.-Q. Yu, *J. Am. Chem. Soc.*, 2014, **136**, 8138-8142.

234. L. Wan, N. Dastbaravardeh, G. Li and J.-Q. Yu, *J. Am. Chem. Soc.*, 2013, **135**, 18056-18059.

235. D. Leow, G. Li, T.-S. Mei and J.-Q. Yu, *Nature*, 2012, **486**, 518-522.

236. T. Parsharamulu, D. Venkanna, M. Lakshmi Kantam, S. K. Bhargava and P. Srinivasu, *Ind. Eng. Chem. Res.*, 2014, **53**, 20075-20084.

237. J. K. Laha, P. U. Shah and K. P. Jethava, *Chem. Commun.* , 2013, **49**, 7623-7625.

238. Q. Chen, L. Ilies and E.-I. Nakamura, *J. Am. Chem. Soc.*, 2011, **133**, 428-429.

239. Q. Chen, L. Ilies, N. Yoshikai and E. Nakamura, *Org. Lett.*, 2011, **13**, 3232-3234.

240. J. Park and S. Chang, *Angew. Chem., Int. Ed.*, 2015, **54**, 14103-14107.

241. D. Lee, Y. Kim and S. Chang, *J. Org. Chem.*, 2013, **78**, 11102-11109.

242. T. M. Figg, S. Park, J. Park, S. Chang and D. G. Musaev, *Organometallics*, 2014, **33**, 4076-4085.

243. K. Shin, Y. Baek and S. Chang, *Angew. Chem., Int. Ed.*, 2013, **52**, 8031-8036.

244. K. Shin, H. Kim and S. Chang, *Acc. Chem. Res.*, 2015, **48**, 1040-1052.

245. C. Feng, D. Feng and T.-P. Loh, *Org. Lett.*, 2013, **15**, 3670-3673.

246. B. Li, J. Ma, W. Xie, H. Song, S. Xu and B. Wang, *J. Org. Chem.*, 2013, **78**, 9345-9353.

247. Y. Hashimoto, K. Hirano, T. Satoh, F. Kakiuchi and M. Miura, *Org. Lett.*, 2012, **14**, 2058-2061.

248. Y. Hashimoto, K. Hirano, T. Satoh, F. Kakiuchi and M. Miura, *J. Org. Chem.*, 2013, **78**, 638-646.

249. Y. Zhao and V. Snieckus, *Adv. Synth. Catal.*, 2014, **356**, 1527-1532.

250. T. Besset, D. Cahard and X. Pannecoucke, *J. Org. Chem.*, 2014, **79**, 413-418.

251. Y. Shibata, Y. Otake, M. Hirano and K. Tanaka, *Org. Lett.*, 2009, **11**, 689-692.

252. K. D. Hesp, R. G. Bergman and J. A. Ellman, *Org. Lett.*, 2012, **14**, 2304-2307.

253. K. D. Collins, F. Lied and F. Glorius, *Chem. Commun.* , 2014, **50**, 4459-4461.

254. R. A. McPherson, M. M. Taylor, E. D. Hershey and T. W. Sturgill, *Oncogene*, 2000, **19**, 3616-3622.

255. H. Wang, N. Schroeder and F. Glorius, *Angew. Chem., Int. Ed.*, 2013, **52**, 5386-5389.

256. L. Zhou and W. Lu, *Org. Lett.*, 2014, **16**, 508-511.

257. J. Li and L. Ackermann, *Org. Chem. Front.*, 2015, **2**, 1035-1039.

258. T. Gensch, S. Vasquez-Cespedes, D.-G. Yu and F. Glorius, *Org. Lett.*, 2015, **17**, 3714-3717.

259. N. Quinones, A. Seoane, R. Garcia-Fandino, J. L. Mascarenas and M. Gulias, *Chem. Sci.*, 2013, **4**, 2874-2879.

260. Y. Shang, X. Jie, H. Zhao, P. Hu and W. Su, *Org. Lett.*, 2014, **16**, 416-419.

261. S. Sharma, S. Han, M. Kim, N. K. Mishra, J. Park, Y. Shin, J. Ha, J. H. Kwak, Y. H. Jung and I. S. Kim, *Org. Biomol. Chem.*, 2014, **12**, 1703-1706.

262. Y. Lu, H.-W. Wang, J. E. Spangler, K. Chen, P.-P. Cui, Y. Zhao, W.-Y. Sun and J.-Q. Yu, *Chem. Sci.*, 2015, **6**, 1923-1927.

263. M. Wasa, K. M. Engle and J.-Q. Yu, *J. Am. Chem. Soc.*, 2009, **131**, 9886-9887.

264. M. Wasa and J.-Q. Yu, *Tetrahedron*, 2010, **66**, 4811-4815.

265. M. Wasa, K. M. Engle and J.-Q. Yu, *J. Am. Chem. Soc.*, 2010, **132**, 3680-3681.

266. P.-X. Shen, X.-C. Wang, P. Wang, R.-Y. Zhu and J.-Q. Yu, *J. Am. Chem. Soc.*, 2015, **137**, 11574-11577.

267. X.-C. Wang, W. Gong, L.-Z. Fang, R.-Y. Zhu, S. Li, K. M. Engle and J.-Q. Yu, *Nature*, 2015, **519**, 334-338.

268. W. Yang, S. Ye, D. Fanning, T. Coon, Y. Schmidt, P. Krenitsky, D. Stamos and J.-Q. Yu, *Angew. Chem., Int. Ed.*, 2015, **54**, 2501-2504.

269. C. Feng, D. Feng and T.-P. Loh, *Chem. Commun.* , 2015, **51**, 342-345.

270. C. Feng, D. Feng, Y. Luo and T.-P. Loh, *Org. Lett.*, 2014, **16**, 5956-5959.

271. F. Peron, C. Fossey, T. Cailly and F. Fabis, *Org. Lett.*, 2012, **14**, 1827-1829.

272. Y. Wang, C. Li, Y. Li, F. Yin and X.-S. Wang, *Adv. Synth. Catal.*, 2013, **355**, 1724-1728.

273. Y. Fukui, P. Liu, Q. Liu, Z.-T. He, N.-Y. Wu, P. Tian and G.-Q. Lin, *J. Am. Chem. Soc.*, 2014, **136**, 15607-15614.

274. B. Ye, P. A. Donets and N. Cramer, *Angew. Chem., Int. Ed.*, 2014, **53**, 507-511.

275. J. R. Huckins, E. A. Bercot, O. R. Thiel, T.-L. Hwang and M. M. Bio, *J. Am. Chem. Soc.*, 2013, **135**, 14492-14495.

276. G. Chen, T. Shigenari, P. Jain, Z. Zhang, Z. Jin, J. He, S. Li, C. Mapelli, M. M. Miller, M. A. Poss, P. M. Scola, K.-S. Yeung and J.-Q. Yu, *J. Am. Chem. Soc.*, 2015, **137**, 3338-3351.

277. S. Nakanowatari and L. Ackermann, *Chemistry*, 2015, **21**, 16246-16251.

278. D.-G. Yu, F. de Azambuja, T. Gensch, C. G. Daniliuc and F. Glorius, *Angew. Chem., Int. Ed.*, 2014, **53**, 9650-9654.

279. N. Zhang, Q. Yu, R. Chen, J. Huang, Y. Xia and K. Zhao, *Chem. Commun.*, 2013, **49**, 9464-9466.

280. S. Rakshit, C. Grohmann, T. Besset and F. Glorius, *J. Am. Chem. Soc.*, 2011, **133**, 2350-2353.

281. B. Li, J. Ma, N. Wang, H. Feng, S. Xu and B. Wang, *Org. Lett.*, 2012, **14**, 736-739.

282. Z. Shu, W. Li and B. Wang, *ChemCatChem*, 2015, **7**, 605-608.

283. G.-W. Wang and T.-T. Yuan, *J. Org. Chem.*, 2010, **75**, 476-479.

284. T. A. Davis, T. K. Hyster and T. Rovis, *Angew. Chem., Int. Ed.*, 2013, **52**, 14181-14185.

285. D. Wang, S. Cai, R. Ben, Y. Zhou, X. Li, J. Zhao, W. Wei and Y. Qian, *Synthesis*, 2014, **46**, 2045-2050.

286. C. Grohmann, H. Wang and F. Glorius, *Org. Lett.*, 2012, **14**, 656-659.

287. F. Szabo, D. Simko and Z. Novak, *RSC Adv.*, 2014, **4**, 3883-3886.

288. Z. Yin and P. Sun, *J. Org. Chem.*, 2012, **77**, 11339-11344.

289. G. Liu, Y. Shen, Z. Zhou and X. Lu, *Angew. Chem., Int. Ed.*, 2013, **52**, 6033-6037.

290. J. Wu, X. Cui, X. Mi, Y. Li and Y. Wu, *Chem. Commun.*, 2010, **46**, 6771-6773.

291. T.-S. Jiang and G.-W. Wang, *J. Org. Chem.*, 2012, **77**, 9504-9509.

292. H. Zhou, W.-J. Chung, Y.-H. Xu and T.-P. Loh, *Chem. Commun.*, 2009, DOI: 10.1039/b903151k, 3472-3474.

293. H. Zhou, Y.-H. Xu, W.-J. Chung and T.-P. Loh, *Angew. Chem., Int. Ed.*, 2009, **48**, 5355-5357.

294. L.-S. Zhang, K. Chen, G. Chen, B.-J. Li, S. Luo, Q.-Y. Guo, J.-B. Wei and Z.-J. Shi, *Org. Lett.*, 2013, **15**, 10-13.

295. G.-W. Wang, T.-T. Yuan and X.-L. Wu, *J. Org. Chem.*, 2008, **73**, 4717-4720.

296. M. D. K. Boele, G. P. F. van Strijdonck, A. H. M. de Vries, P. C. J. Kamer, J. G. de Vries and P. W. N. M. van Leeuwen, *J. Am. Chem. Soc.*, 2002, **124**, 1586-1587.

297. Z. Shi, B. Li, X. Wan, J. Cheng, Z. Fang, B. Cao, C. Qin and Y. Wang, *Angew. Chem., Int. Ed.*, 2007, **46**, 5554-5558.

298. X. Wang, X. Li, J. Xiao, Y. Jiang and X. Li, *Synlett*, 2012, **23**, 1649-1652.

299. L. Ren, W. Chu, D. Guan, Y. Hou, M. Wang, X. Yuan and Z. Sun, *Appl. Organomet. Chem.*, 2014, **28**, 673-677.

300. L.-Y. Jiao and M. Oestreich, *Chem. - Eur. J.*, 2013, **19**, 10845-10848.

301. J. Wen, A. Wu, P. Chen and J. Zhu, *Tetrahedron Lett.*, 2015, **56**, 5282-5286.

302. J. A. Schiffner and M. Oestreich, *Eur. J. Org. Chem.*, 2011, DOI: 10.1002/ejoc.201001526, 1148-1154.

303. J. Park, A. Kim, S. Sharma, M. Kim, E. Park, Y. Jeon, Y. Lee, J. H. Kwak, Y. H. Jung and I. S. Kim, *Org. Biomol. Chem.*, 2013, **11**, 2766-2771.

304. S. Sharma, J. Park, E. Park, A. Kim, M. Kim, J. H. Kwak, Y. H. Jung and I. S. Kim, *Adv. Synth. Catal.*, 2013, **355**, 332-336.

305. N. K. Mishra, J. Park, S. Sharma, S. Han, M. Kim, Y. Shin, J. Jang, J. H. Kwak, Y. H. Jung and I. S. Kim, *Chem. Commun.* , 2014, **50**, 2350-2352.

306. S. De Sarkar, W. Liu, S. I. Kozhushkov and L. Ackermann, *Adv. Synth. Catal.*, 2014, **356**, 1461-1479.

307. K. Padala and M. Jeganmohan, *Org. Lett.*, 2012, **14**, 1134-1137.

308. V. Lanke and K. Ramaiah Prabhu, *Org. Lett.*, 2013, **15**, 6262-6265.

309. S. Chen, J. Yu, Y. Jiang, F. Chen and J. Cheng, *Org. Lett.*, 2013, **15**, 4754-4757.

310. X. Liu, G. Li, F. Song and J. You, *Nat. Commun.*, 2014, **5**.

311. J. Cornella, M. Righi and I. Larrosa, *Angew. Chem., Int. Ed.*, 2011, **50**, 9429-9432.

312. X. Liu, X. Li, H. Liu, Q. Guo, J. Lan, R. Wang and J. You, *Org. Lett.*, 2015, **17**, 2936-2939.

313. C. Zhu, M. Yi, D. Wei, X. Chen, Y. Wu and X. Cui, *Org. Lett.*, 2014, **16**, 1840-1843.

314. B.-F. Shi, Y.-H. Zhang, J. K. Lam, D.-H. Wang and J.-Q. Yu, *J. Am. Chem. Soc.*, 2010, **132**, 460-461.

315. K. M. Engle, D.-H. Wang and J.-Q. Yu, *J. Am. Chem. Soc.*, 2010, **132**, 14137-14151.

316. H.-X. Dai, G. Li, X.-G. Zhang, A. F. Stepan and J.-Q. Yu, *J. Am. Chem. Soc.*, 2013, **135**, 7567-7571.

317. D.-H. Wang, T.-S. Mei and J.-Q. Yu, *J. Am. Chem. Soc.*, 2008, **130**, 17676-17677.

318. H. A. Chiong, Q.-N. Pham and O. Daugulis, *J. Am. Chem. Soc.*, 2007, **129**, 9879-9884.

319. J. Gallardo-Donaire and R. Martin, *J. Am. Chem. Soc.*, 2013, **135**, 9350-9353.

320. Y.-H. Zhang and J.-Q. Yu, *J. Am. Chem. Soc.*, 2009, **131**, 14654-14655.

321. M. Miura, T. Tsuda, T. Satoh, S. Pivsa-Art and M. Nomura, *J. Org. Chem.*, 1998, **63**, 5211-5215.

322. K. M. Engle, D.-H. Wang and J.-Q. Yu, *Angew. Chem.*, 2010, **122**, 6305-6309.

323. S. Mochida, K. Hirano, T. Satoh and M. Miura, *Org. Lett.*, 2010, **12**, 5776-5779.

324. L. Ackermann, J. Pospech, K. Graczyk and K. Rauch, *Org. Lett.*, 2012, **14**, 930-933.

325. J. M. Lee and S. Chang, *Tetrahedron Lett.*, 2006, **47**, 1375-1379.

326. Z. Xu, T. Yang, X. Lin, J. D. Elliott and F. Ren, *Tetrahedron Lett.*, 2015, **56**, 475-477.

327. N. Dastbaravardeh, T. Toba, M. E. Farmer and J.-Q. Yu, *J. Am. Chem. Soc.*, 2015, **137**, 9877-9884.

328. C. Zhu, Y. Zhang, J. Kan, H. Zhao and W. Su, *Org. Lett.*, 2015, **17**, 3418-3421.

329. S.-C. Sha, J. Zhang and P. J. Walsh, *Org. Lett.*, 2015, **17**, 410-413.

330. R. Giri and J.-Q. Yu, *J. Am. Chem. Soc.*, 2008, **130**, 14082-14083.

331. K. M. Engle, T.-S. Mei, M. Wasa and J.-Q. Yu, *Acc. Chem. Res.*, 2012, **45**, 788-802.

332. J. Dupont, C. S. Consorti and J. Spencer, *Chem. Rev.*, 2005, **105**, 2527-2572.

333. S. Mochida, K. Hirano, T. Satoh and M. Miura, *J. Org. Chem.*, 2011, **76**, 3024-3033.

334. T. Ueyama, S. Mochida, T. Fukutani, K. Hirano, T. Satoh and M. Miura, *Org. Lett.*, 2011, **13**, 706-708.

335. L. Ackermann and J. Pospech, *Org. Lett.*, 2011, **13**, 4153-4155.

336. K. Ueura, T. Satoh and M. Miura, *J. Org. Chem.*, 2007, **72**, 5362-5367.

337. Y.-H. Zhang, B.-F. Shi and J.-Q. Yu, *Angew. Chem.*, 2009, **121**, 6213-6216.

338. K. Ueura, T. Satoh and M. Miura, *Org. Lett.*, 2007, **9**, 1407-1409.

339. M. Deponti, S. I. Kozhushkov, D. S. Yufit and L. Ackermann, *Organic & Biomolecular Chemistry*, 2013, **11**, 142-148.

340. R. Giri, N. Maugel, J.-J. Li, D.-H. Wang, S. P. Breazzano, L. B. Saunders and J.-Q. Yu, *J. Am. Chem. Soc.*, 2007, **129**, 3510-3511.

341. C. Arroniz, A. Ironmonger, G. Rassias and I. Larrosa, *Org. Lett.*, 2013, **15**, 910-913.

342. T.-S. Mei, D.-H. Wang and J.-Q. Yu, *Organic letters*, 2010, **12**, 3140-3143.

343. Y. Wang, A. V. Gulevich and V. Gevorgyan, *Chem. - Eur. J.*, 2013, **19**, 15836-15840.

344. M. Yang, X. Jiang, W.-J. Shi, Q.-L. Zhu and Z.-J. Shi, *Org. Lett.*, 2013, **15**, 690-693.

345. X.-F. Cheng, Y. Li, Y.-M. Su, F. Yin, J.-Y. Wang, J. Sheng, H. U. Vora, X.-S. Wang and J.-Q. Yu, *J. Am. Chem. Soc.*, 2013, **135**, 1236-1239.

346. L. D. Tran, J. Roane and O. Daugulis, *Angew. Chem., Int. Ed.*, 2013, **52**, 6043-6046.

347. X. Shi and C.-J. Li, *Adv. Synth. Catal.*, 2012, **354**, 2933-2938.

348. K. Takenaka, M. Akita, Y. Tanigaki, S. Takizawa and H. Sasai, *Org. Lett.*, 2011, **13**, 3506-3509.

349. B. M. Trost, K. Imi and I. W. Davies, *J. Am. Chem. Soc.*, 1995, **117**, 5371-5372.

350. M. Sonoda, F. Kakiuchi, A. Kamatani, N. Chatani and S. Murai, *Chem. Lett.*, 1996, **25**, 109-110.

351. S. Murai, F. Kakiuchi, S. Sekine, Y. Tanaka, A. Kamatani, M. Sonoda and N. Chatani, *Nature*, 1993, **366**, 529-531.

352. K. Padala, S. Pimparkar, P. Madasamy and M. Jeganmohan, *Chem. Commun.*, 2012, **48**, 7140-7142.

353. J. Kim and S. Chang, *Angew. Chem., Int. Ed.*, 2014, **53**, 2203-2207.

354. X. Sun, G. Shan, Y. Sun and Y. Rao, *Angew. Chem., Int. Ed.*, 2013, **52**, 4440-4444.

355. G. Shan, X. Yang, L. Ma and Y. Rao, *Angew. Chem., Int. Ed.*, 2012, **51**, 13070-13074.

356. Y. Yang, Y. Lin and Y. Rao, *Org. Lett.*, 2012, **14**, 2874-2877.

357. A. Renaudat, L. Jean-Gérard, R. Jazzar, C. E. Kefalidis, E. Clot and O. Baudoin, *Angew. Chem., Int. Ed.*, 2010, **49**, 7261-7265.

358. P. Larini, C. E. Kefalidis, R. Jazzar, A. Renaudat, E. Clot and O. Baudoin, *Chem. - Eur. J.*, 2012, **18**, 1932-1944.
359. S. H. Park, J. Y. Kim and S. Chang, *Org. Lett.*, 2011, **13**, 2372-2375.
360. K. Graczyk, W. Ma and L. Ackermann, *Org. Lett.*, 2012, **14**, 4110-4113.
361. Z. Huang, H. N. Lim, F. Mo, M. C. Young and G. Dong, *Chem Soc Rev*, 2015, **44**, 7764-7786.
362. F. Mo and G. Dong, *Science*, 2014, **345**, 68-72.
363. S. Busch and W. Leitner, *Adv. Synth. Catal.*, 2001, **343**, 192-195.
364. F. Mo, L. J. Trzepkowski and G. Dong, *Angew. Chem., Int. Ed.*, 2012, **51**, 13075-13079.
365. F. Kakiuchi, T. Kochi, E. Mizushima and S. Murai, *J. Am. Chem. Soc.*, 2010, **132**, 17741-17750.
366. T. Matsubara, N. Koga, D. G. Musaev and K. Morokuma, *J. Am. Chem. Soc.*, 1998, **120**, 12692-12693.
367. Y. Guari, A. Castellanos, S. Sabo-Etienne and B. Chaudret, *Journal of Molecular Catalysis A: Chemical*, 2004, **212**, 77-82.
368. K. R. Bettadapur, V. Lanke and K. R. Prabhu, *Organic letters*, 2015, **17**, 4658-4661.
369. F. Kakiuchi, Y. Yamamoto, N. Chatani and S. Murai, *Chem. Lett.*, 1995, **24**, 681-682.
370. S. Kathiravan and I. A. Nicholls, *Eur. J. Org. Chem.*, 2014, **2014**, 7211-7219.
371. G. E. M. Crisenza, N. G. McCreanor and J. F. Bower, *J. Am. Chem. Soc.*, 2014, **136**, 10258-10261.
372. F. W. Patureau, T. Besset, N. Kuhl and F. Glorius, *J. Am. Chem. Soc.*, 2011, **133**, 2154-2156.
373. R. K. Chinnagolla and M. Jeganmohan, *Eur. J. Org. Chem.*, 2012, **2012**, 417-423.
374. K. Muralirajan, K. Parthasarathy and C.-H. Cheng, *Angew. Chem., Int. Ed.*, 2011, **50**, 4169-4172.
375. Z. Huang and G. Dong, *J. Am. Chem. Soc.*, 2013, **135**, 17747-17750.
376. C.-H. Jun, C. W. Moon, Y.-M. Kim, H. Lee and J. H. Lee, *Tetrahedron Lett.*, 2002, **43**, 4233-4236.
377. K. Tsuchikama, Y. Kuwata, Y.-k. Tahara, Y. Yoshinami and T. Shibata, *Org. Lett.*, 2007, **9**, 3097-3099.
378. P. Y. Choy and F. Y. Kwong, *Org. Lett.*, 2013, **15**, 270-273.
379. V. S. Thirunavukkarasu and L. Ackermann, *Org. Lett.*, 2012, **14**, 6206-6209.
380. N. Schröder, J. Wencel-Delord and F. Glorius, *Journal of the American Chemical Society*, 2012, **134**, 8298-8301.
381. M. Bhanuchandra, M. Ramu Yadav, R. K. Rit, M. Rao Kuram and A. K. Sahoo, *Chem. Commun.*, 2013, **49**, 5225-5227.
382. J. Kim, J. Kim and S. Chang, *Chem. - Eur. J.*, 2013, **19**, 7328-7333.
383. Q.-Z. Zheng, Y.-F. Liang, C. Qin and N. Jiao, *Chem. Commun.*, 2013, **49**, 5654-5656.
384. R. Santhoshkumar, S. Mannathan and C.-H. Cheng, *Org. Lett.*, 2014, **16**, 4208-4211.
385. H. Li, R.-Y. Zhu, W.-J. Shi, K.-H. He and Z.-J. Shi, *Organic letters*, 2012, **14**, 4850-4853.

386. P. Gandeepan, C.-H. Hung and C.-H. Cheng, *Chem. Commun.*, 2012, **48**, 9379-9381.

387. K. Tsuchikama, M. Kasagawa, Y.-K. Hashimoto, K. Endo and T. Shibata, *J. Organomet. Chem.*, 2008, **693**, 3939-3942.

388. K. Padala and M. Jeganmohan, *Org. Lett.*, 2011, **13**, 6144-6147.

389. F. Kakiuchi, T. Uetsuhara, Y. Tanaka, N. Chatani and S. Murai, *Journal of Molecular Catalysis A: Chemical*, 2002, **182–183**, 511-514.

390. X.-Y. Shi and C.-J. Li, *Organic letters*, 2013, **15**, 1476-1479.

391. S. Busch and W. Leitner, *Chem. Commun.*, 1999, DOI: 10.1039/A906580F, 2305-2306.

392. C.-H. Jun, C. W. Moon, J.-B. Hong, S.-G. Lim, K.-Y. Chung and Y.-H. Kim, *Chem. - Eur. J.*, 2002, **8**, 485-492.

393. C. P. Lenges and M. Brookhart, *J. Am. Chem. Soc.*, 1999, **121**, 6616-6623.

394. R. Martinez, R. Chevalier, S. Darses and J.-P. Genet, *Angew. Chem., Int. Ed.*, 2006, **45**, 8232-8235.

395. R. Martinez, M.-O. Simon, R. Chevalier, C. Pautigny, J.-P. Genet and S. Darses, *J. Am. Chem. Soc.*, 2009, **131**, 7887-7895.

396. M.-O. Simon, R. Martinez, J.-P. Genet and S. Darses, *J. Org. Chem.*, 2010, **75**, 208-210.

397. M.-O. Simon, J.-P. Genet and S. Darses, *Org. Lett.*, 2010, **12**, 3038-3041.

398. P. Gandeepan, K. Parthasarathy and C.-H. Cheng, *J. Am. Chem. Soc.*, 2010, **132**, 8569-8571.

399. F. Kakiuchi, S. Kan, K. Igi, N. Chatani and S. Murai, *J. Am. Chem. Soc.*, 2003, **125**, 1698-1699.

400. Y. Terao, Y. Kametani, H. Wakui, T. Satoh, M. Miura and M. Nomura, *Tetrahedron*, 2001, **57**, 5967-5974.

401. D. Leow, G. Li, T.-S. Mei and J.-Q. Yu, *Nature (London, U. K.)*, 2012, **486**, 518-522.

402. M. M. Coulter, P. K. Dornan and V. M. Dong, *J. Am. Chem. Soc.*, 2009, **131**, 6932-6933.

403. X. Wang, Y. Lu, H.-X. Dai and J.-Q. Yu, *J. Am. Chem. Soc.*, 2010, **132**, 12203-12205.

404. Y. Lu, D. Leow, X. Wang, K. M. Engle and J.-Q. Yu, *Chem. Sci.*, 2011, **2**, 967-971.

405. W. Zi, Y.-M. Wang and F. D. Toste, *J. Am. Chem. Soc.*, 2014, **136**, 12864-12867.

406. R. B. Bedford and M. E. Limmert, *J. Org. Chem.*, 2003, **68**, 8669-8682.

407. R. B. Bedford, S. J. Coles, M. B. Hursthouse and M. E. Limmert, *Angew. Chem., Int. Ed.* , 2003, **42**, 112-114.

408. T. Satoh, Y. Kawamura, M. Miura and M. Nomura, *Angew. Chem., Int. Ed.*, 1997, **36**, 1740-1742.

409. D. D. Hennings, S. Iwasa and V. H. Rawal, *J. Org. Chem.*, 1997, **62**, 2-3.

410. S. Reddy Chidipudi, M. D. Wieczysty, I. Khan and H. W. Lam, *Org. Lett.*, 2013, **15**, 570-573.

411. J. D. Dooley, S. Reddy Chidipudi and H. W. Lam, *J. Am. Chem. Soc.*, 2013, **135**, 10829-10836.

412. S. R. Kandukuri, L.-Y. Jiao, A. B. Machotta and M. Oestreich, *Adv. Synth. Catal.*, 2014, **356**, 1597-1609.

413. V. S. Thirunavukkarasu, M. Donati and L. Ackermann, *Org. Lett.*, 2012, **14**, 3416-3419.

414. K. Inamoto, J. Kadokawa and Y. Kondo, *Org. Lett.*, 2013, **15**, 3962-3965.
415. Y. Yang, B. Zhou and Y. Li, *Adv. Synth. Catal.*, 2012, **354**, 2916-2920.
416. A. S. Tsai, M. Brasse, R. G. Bergman and J. A. Ellman, *Org. Lett.*, 2011, **13**, 540-542.
417. S. Yu, B. Wan and X. Li, *Org. Lett.*, 2013, **15**, 3706-3709.
418. Y. Park, S. Jee, J. G. Kim and S. Chang, *Org. Process Res. Dev.*, 2015, **19**, 1024-1029.
419. H. Wang, G. Tang and X. Li, *Angew. Chem., Int. Ed.*, 2015, **54**, 13049-13052.
420. J. Wen, A. Wu, Y. Miao and J. Zhu, *Tetrahedron Lett.*, 2015, **56**, 5512-5516.
421. H.-Y. Thu, W.-Y. Yu and C.-M. Che, *J. Am. Chem. Soc.*, 2006, **128**, 9048-9049.
422. S.-J. Lou, D.-Q. Xu and Z.-Y. Xu, *Angew. Chem., Int. Ed.*, 2014, **53**, 10330-10335.
423. Y.-F. Liang, X. Wang, Y. Yuan, Y. Liang, X. Li and N. Jiao, *ACS Catal.*, 2015, **5**, 6148-6152.
424. W. Zhang, S. Lou, Y. Liu and Z. Xu, *J. Org. Chem.*, 2013, **78**, 5932-5948.
425. S. Yu, B. Wan and X. Li, *Org. Lett.*, 2015, **17**, 58-61.
426. D. Qin, J. Wang, X. Qin, C. Wang, G. Gao and J. You, *Chem. Commun.*, 2015, **51**, 6190-6193.
427. C.-H. Yeh, W.-C. Chen, P. Gandeepan, Y.-C. Hong, C.-H. Shih and C.-H. Cheng, *Org. Biomol. Chem.*, 2014, **12**, 9105-9108.
428. L. V. Desai, K. L. Hull and M. S. Sanford, *J. Am. Chem. Soc.*, 2004, **126**, 9542-9543.
429. J. Peng, C. Chen and C. Xi, *Chem. Sci.*, 2016, **7**, 1383-1387.
430. J. R. Hummel and J. A. Ellman, *J. Am. Chem. Soc.*, 2015, **137**, 490-498.
431. M. Kim, J. Park, S. Sharma, A. Kim, E. Park, J. H. Kwak, Y. H. Jung and I. S. Kim, *Chem. Commun.*, 2013, **49**, 925-927.
432. B. Liu, F. Hu and B.-F. Shi, *Adv. Synth. Catal.*, 2014, **356**, 2688-2696.
433. C.-L. Sun, N. Liu, B.-J. Li, D.-G. Yu, Y. Wang and Z.-J. Shi, *Org. Lett.*, 2010, **12**, 184-187.
434. B. Zhou, W. Hou, Y. Yang and Y. Li, *Chem. - Eur. J.*, 2013, **19**, 4701-4706.
435. W. Hou, B. Zhou, Y. Yang, H. Feng and Y. Li, *Org. Lett.*, 2013, **15**, 1814-1817.
436. Y. Lian, T. Huber, K. D. Hesp, R. G. Bergman and J. A. Ellman, *Angew. Chem., Int. Ed.*, 2013, **52**, 629-633.
437. B. Sun, T. Yoshino, M. Kanai and S. Matsunaga, *Angew. Chem., Int. Ed.*, 2015, DOI: 10.1002/anie.201507744, Ahead of Print.
438. X. Tang, L. Huang, Y. Xu, J. Yang, W. Wu and H. Jiang, *Angew. Chem., Int. Ed.*, 2014, **53**, 4205-4208.
439. J. M. Neely and T. Rovis, *J. Am. Chem. Soc.*, 2013, **135**, 66-69.
440. H. Chu, S. Sun, J.-T. Yu and J. Cheng, *Chem. Commun.* , 2015, **51**, 13327-13329.
441. D. Zhao, F. Lied and F. Glorius, *Chem. Sci.*, 2014, **5**, 2869-2873.

442. Z.-W. Zhang, A. Lin and J. Yang, *J. Org. Chem.*, 2014, **79**, 7041-7050.
443. F. Romanov-Michailidis, K. F. Sedillo, J. M. Neely and T. Rovis, *J. Am. Chem. Soc.*, 2015, **137**, 8892-8895.
444. K. Parthasarathy and C.-H. Cheng, *J. Org. Chem.*, 2009, **74**, 9359-9364.
445. M. Sen, D. Kalsi and B. Sundararaju, *Chemistry*, 2015, **21**, 15529-15533.
446. Z. Shi, D. C. Koester, M. Boultadakis-Arapinis and F. Glorius, *J. Am. Chem. Soc.*, 2013, **135**, 12204-12207.
447. Y. Zhang, Q. Wu and S. Cui, *Chem. Sci.*, 2014, **5**, 297-302.
448. C. Kornhaass, C. Kuper and L. Ackermann, *Adv. Synth. Catal.*, 2014, **356**, 1619-1624.
449. Y. Xu, W. Hu, X. Tang, J. Zhao, W. Wu and H. Jiang, *Chem. Commun.*, 2015, **51**, 6843-6846.
450. K. Guo, X. Chen, M. Guan and Y. Zhao, *Org. Lett.*, 2015, **17**, 1802-1805.
451. X.-P. Zhang, D. Chen, M. Zhao, J. Zhao, A.-Q. Jia and X.-W. Li, *Adv. Synth. Catal.*, 2011, **353**, 719-723.
452. P. Sun, Y. Wu, T. Yang, X. Wu, J. Xu, A. Lin and H. Yao, *Adv. Synth. Catal.*, 2015, **357**, 2469-2473.
453. K. Parthasarathy, M. Jeganmohan and C.-H. Cheng, *Org. Lett.*, 2008, **10**, 325-328.
454. T. K. Hyster and T. Rovis, *Chem. Commun. (Cambridge, U. K.)*, 2011, **47**, 11846-11848.
455. Z. Xu, B. Xiang and P. Sun, *Eur. J. Org. Chem.*, 2012, **2012**, 3069-3073.
456. V. S. Thirunavukkarasu, K. Parthasarathy and C.-H. Cheng, *Angew. Chem., Int. Ed.*, 2008, **47**, 9462-9465.
457. V. S. Thirunavukkarasu and C.-H. Cheng, *Chem. - Eur. J.*, 2011, **17**, 14723-14726.
458. W. Zhang, D. Wu, J. Zhang and Y. Liu, *Eur. J. Org. Chem.*, 2014, **2014**, 5827-5835.
459. Y. Park, J. Seo, S. Park, E. J. Yoo and P. H. Lee, *Chem. - Eur. J.*, 2013, **19**, 16461-16468.
460. Y. Park, I. Jeon, S. Shin, J. Min and P. H. Lee, *J. Org. Chem.*, 2013, **78**, 10209-10220.
461. J. Seo, Y. Park, I. Jeon, T. Ryu, S. Park and P. H. Lee, *Organic letters*, 2013, **15**, 3358-3361.
462. T. Ryu, J. Kim, Y. Park, S. Kim and P. H. Lee, *Org. Lett.*, 2013, **15**, 3986-3989.
463. D. Eom, Y. Jeong, Y. R. Kim, E. Lee, W. Choi and P. H. Lee, *Org. Lett.*, 2013, **15**, 5210-5213.
464. S. Shin, D. Kang, W. H. Jeon and P. H. Lee, *Beilstein J. Org. Chem.*, 2014, **10**, 1220-1227.
465. Q. Xu, R. Shen, Y. Ono, R. Nagahata, S. Shimada, M. Goto and L.-B. Han, *Chem. Commun.*, 2011, **47**, 2333-2335.
466. H. Zhang, R.-B. Hu, X.-Y. Zhang, S.-X. Li and S.-D. Yang, *Chem. Commun.*, 2014, **50**, 4686-4689.
467. L. Y. Chan, L. Cheong and S. Kim, *Org. Lett.*, 2013, **15**, 2186-2189.
468. L. Y. Chan, X. Meng and S. Kim, *J. Org. Chem.*, 2013, **78**, 8826-8832.
469. Y.-R. Chen and W.-L. Duan, *J. Am. Chem. Soc.*, 2013, **135**, 16754-16757.

470. B. C. Chary, S. Kim, Y. Park, J. Kim and P. H. Lee, *Org. Lett.*, 2013, **15**, 2692-2695.
471. S. Park, B. Seo, S. Shin, J.-Y. Son and P. H. Lee, *Chem. Commun.*, 2013, **49**, 8671-8673.
472. M. Itoh, Y. Hashimoto, K. Hirano, T. Satoh and M. Miura, *J. Org. Chem.*, 2013, **78**, 8098-8104.
473. D. Zhao, C. Nimphius, M. Lindale and F. Glorius, *Org. Lett.*, 2013, **15**, 4504-4507.
474. W. H. Jeon, T. S. Lee, E. J. Kim, B. Moon and J. Kang, *Tetrahedron*, 2013, **69**, 5152-5159.
475. X. Meng and S. Kim, *Org. Lett.*, 2013, **15**, 1910-1913.
476. X. Meng and S. Kim, *J. Org. Chem.*, 2013, **78**, 11247-11254.
477. X. H. Hu, X. F. Yang and T. P. Loh, *Angew. Chem., Int. Ed.*, 2015, **54**, 15535-15539.
478. J. Guan, G.-J. Wu and F.-S. Han, *Chem. - Eur. J.*, 2014, **20**, 3301-3305.
479. Y. Hatanaka, M. Watanabe, S.-y. Onozawa, M. Tanaka and H. Sakurai, *J. Org. Chem.*, 1998, **63**, 422-423.
480. P. Becker, D. L. Priebbenow, R. Pirwerdjan and C. Bolm, *Angew. Chem., Int. Ed.*, 2014, **53**, 269-271.
481. C. Wang and H. Ge, *Chem. - Eur. J.*, 2011, **17**, 14371-14374.
482. C. Huang, N. Ghavtadze, B. Godoi and V. Gevorgyan, *Chem. - Eur. J.*, 2012, **18**, 9789-9792.
483. S. Lee, H. Lee and K. L. Tan, *J. Am. Chem. Soc.*, 2013, **135**, 18778-18781.
484. S. Bag, T. Patra, A. Modak, A. Deb, S. Maity, U. Dutta, A. Dey, R. Kancherla, A. Maji, A. Hazra, M. Bera and D. Maiti, *J. Am. Chem. Soc.*, 2015, **137**, 11888-11891.
485. C.-H. Huang, B. Chattopadhyay and V. Gevorgyan, *J. Am. Chem. Soc.*, 2011, **133**, 12406-12409.
486. H. Li, P. Li and L. Wang, *Org. Lett.*, 2013, **15**, 620-623.
487. L. Qiu, D. Huang, G. Xu, Z. Dai and J. Sun, *Org. Lett.*, 2015, **17**, 1810-1813.
488. H. Song, D. Chen, C. Pi, X. Cui and Y. Wu, *J. Org. Chem.*, 2014, **79**, 2955-2962.
489. Z. Yin, X. Jiang and P. Sun, *J. Org. Chem.*, 2013, **78**, 10002-10007.
490. C. Xia, Z. Wei, C. Shen, J. Xu, Y. Yang, W. Su and P. Zhang, *RSC Adv.*, 2015, **5**, 52588-52594.
491. D. Zhang, X. Cui, Q. Zhang and Y. Wu, *J. Org. Chem.*, 2015, **80**, 1517-1522.
492. X. Geng and C. Wang, *Org. Biomol. Chem.*, 2015, **13**, 7619-7623.
493. H. Wang, Y. Yu, X. Hong, Q. Tan and B. Xu, *J. Org. Chem.*, 2014, **79**, 3279-3288.
494. Y. Lian, R. G. Bergman, L. D. Lavis and J. A. Ellman, *J. Am. Chem. Soc.*, 2013, **135**, 7122-7125.
495. B. Majhi, D. Kundu, S. Ahammed and B. C. Ranu, *Chem. - Eur. J.*, 2014, **20**, 9862-9866.
496. C. Premi, A. Dixit and N. Jain, *Org. Lett.*, 2015, **17**, 2598-2601.
497. F. Xiong, C. Qian, D. Lin, W. Zeng and X. Lu, *Org. Lett.*, 2013, **15**, 5444-5447.

498. H. Tang, C. Qian, D. Lin, H. Jiang and W. Zeng, *Adv. Synth. Catal.*, 2014, **356**, 519-527.
499. Z.-Y. Li, D.-D. Li and G.-W. Wang, *J. Org. Chem.*, 2013, **78**, 10414-10420.
500. C. Qian, D. Lin, Y. Deng, X.-Q. Zhang, H. Jiang, G. Miao, X. Tang and W. Zeng, *Org. Biomol. Chem.*, 2014, **12**, 5866-5875.
501. H. Deng, H. Li and L. Wang, *Org. Lett.*, 2015, **17**, 2450-2453.
502. S. Sharma, S. H. Han, S. Han, W. Ji, J. Oh, S.-Y. Lee, J. S. Oh, Y. H. Jung and I. S. Kim, *Org. Lett.*, 2015, **17**, 2852-2855.
503. X. Jia and J. Han, *J. Org. Chem.*, 2014, **79**, 4180-4185.
504. N. Khatun, A. Modi, W. Ali and B. K. Patel, *J. Org. Chem.*, 2015, **80**, 9662-9670.
505. Y. Lian, J. R. Hummel, R. G. Bergman and J. A. Ellman, *J. Am. Chem. Soc.*, 2013, **135**, 12548-12551.
